# B(C_6_F_5_)_3_‑Catalyzed Coupling of *N*‑Alkyl Arylamines and Alkenes for the Synthesis of Tetrahydroquinolines

**DOI:** 10.1021/acs.orglett.5c03023

**Published:** 2025-09-03

**Authors:** Sophia Moreen Gloria, Laura Winfrey, Yuncong Gao, Maryia Barysevich, Joseph P. Gillions, Lei Yun, Halima Patel, Amy Shah, Ana Alvarez-Montoya, Dean Thomas, Ahmad Khan, Hassan Y. Harb, Alison M. Stuart, Alexander P. Pulis

**Affiliations:** † School of Chemistry, 4488 University of Leicester, Leicester LE1 7RH, U.K.; ‡ Department of Chemistry, 5292 University of Manchester, Manchester M13 9PL, U.K.; § Concept Life Sciences Ltd., Frith Knoll Road, Chapel-en-le-Frith, High Peak SK23 0PG, U.K.

## Abstract

Tetrahydroquinolines are featured in molecules with wide ranging properties, including those with biological activity and materials applications. This report demonstrates that widely accessible *N*-alkyl arylamines can be directly coupled with new classes of alkenes to synthesize tetrahydroquinolines. The reaction is facilitated by a commercially available borane catalyst and enables the creation of novel tetrahydroquinoline scaffolds.

## Graphical abstract

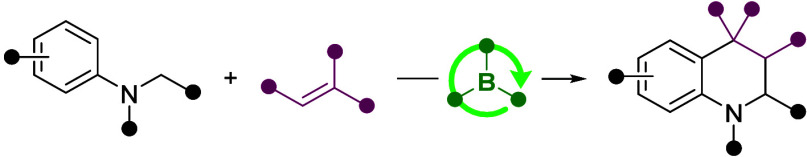

1,2,3,4-Tetrahydroquinolines (THQs) are important nitrogen heterocycles that are found in natural and synthetic products with broad applications (Scheme 1A).^1,2^ THQs are present in dyes^3^ and agrochemicals^4^ and are ligands in coordination chemistry.^5^ There are a wide array of naturally occurring THQs,^2^ many of which display biological activity relevant to human health, including anti-inflammatory,^6^ antiparasitic,^7^ antibiotic,^8^ and cytotoxic effects.^9^ Consequently, THQs are valuable to the pharmaceutical industry and are featured in FDA-approved drugs.^10^

**Scheme 1:**
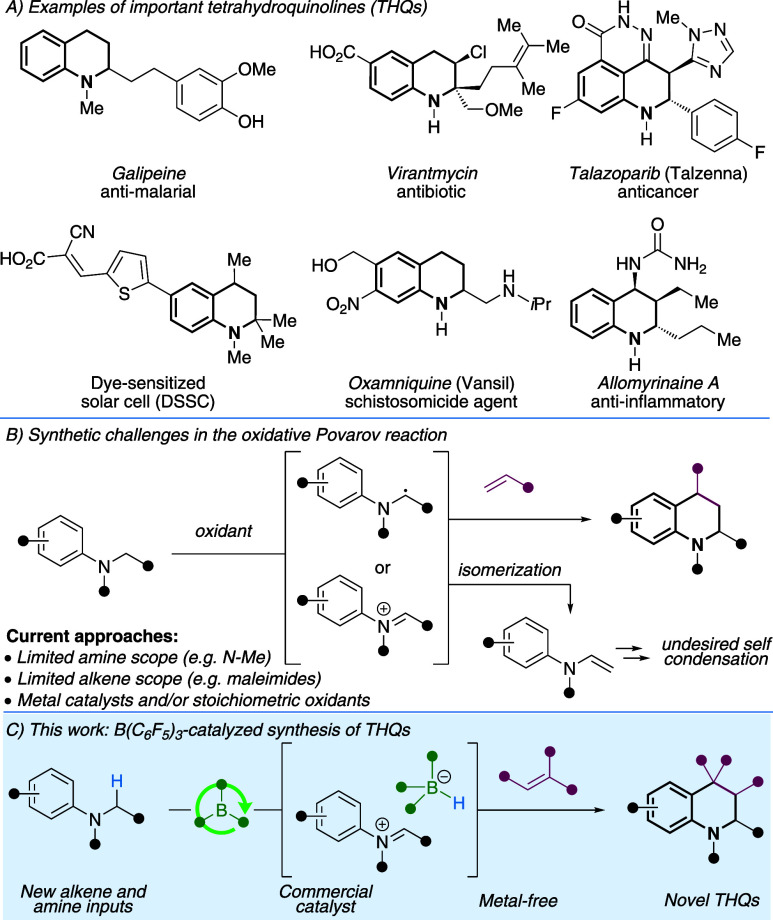
Importance of Tetrahydroquinolines and Their Synthetic Challenges

Given the broad significance of THQs, a variety of methods exist for their synthesis.^1^ One potentially powerful strategy involves the direct coupling of readily accessible *N*-alkyl arylamines and alkenes, via the *in situ* formation of α-aminoalkyl radicals or iminium ions (Scheme 1B). This approach (sometimes termed an oxidative Povarov reaction) was first reported by Roy and Swan in 1968, where benzoyl peroxide was used to mediate the coupling of *N*-methyl arylamines and maleimides.^11^ Since this seminal report, a variety of catalysts and stoichiometric oxidants have been employed.^1,12–15^ However, developments beyond the holotype reported by Swan have been scarce, and most methods employ electron-deficient alkenes (typically maleimides) and *N*-methyl arylamines, greatly limiting the structural diversity possible via this approach. Miura and co-workers reported the coupling of *N*,*N*-dimethyl arylamines with vinyl ethers using oxygen and an Fe­(III) catalyst.^12a^ Minisci discovered that *N*,*N*-dimethyl arylamines and *N*,*N*-diethyl arylamines react with acrylonitrile, *t*BuOOH and a Cu­(OAc)_2_ catalyst to form THQs.^12b^ Rueping reported the reaction between *N*,*N*-dimethyl arylamines and 2-benzylidenemalononitrile derivatives using [Ir­(ppy)_2_bpy]­PF_6_ catalyst and blue LEDs in air.^12c^ Electrochemical oxidation of *N*-methyl arylamines allowed coupling to *N*-acyl enamines, as described by Lei.^12e^ The use of aryl alkenes (i.e., styrene derivatives) as coupling partners is limited to *N*-benzyl arylamines^13^ or *N*-aryl glycine derivatives^14^ and generates quinolines, instead of THQs. A notable exception was reported by Wang and co-workers, where *N*,*N*-dimethyl arylamines were oxidized with diiodomethane, and subsequently coupled with mono and 1,1-disubstituted aryl alkenes to form THQs.^15^

Herein, we show for the first time that a commercially available borane is an effective catalyst for generating novel THQ scaffolds. The B­(C_6_F_5_)_3_ catalyst directly activates a range of readily available *N*-alkyl arylamines, under conditions that are mild so that new alkenes can be employed and deleterious side reactions resulting from iminium-enamine isomerization are avoided. We report a rare example of aryl alkenes being utilized in the oxidative Povarov reaction, and the first-time that allyl silanes have been utilized. The use of new *N*-alkyl arylamines and new classes of alkenes has allowed novel THQ chemical space to be accessed, including those with 4,4-disubstitution, C2 alkyl substituents, polycycles, and spirocycles. The method is metal-free and can be performed with standard techniques, without requiring a glovebox. The novel THQs undergo a variety of further functionalization reactions to allow access to even more complex scaffolds.

Aiming to expand the library of synthetically accessible THQs, we postulated that B­(C_6_F_5_)_3_ could serve as a new catalyst for the oxidative Povarov reaction via its ability to directly generate iminium ions from *N*-alkyl arylamines under mild conditions via α-nitrogen hydride abstraction.^16–18^ B­(C_6_F_5_)_3_-catalyzed coupling of *N*-alkyl arylamines and alkenes to form THQs has not previously been reported. In addition, previous reports of coupling alkenes and iminium ions generated through B­(C_6_F_5_)_3_-mediated hydride abstraction have utilized highly nucleophilic alkenes, such as enolates, and generate Mannich addition-type products.^16b,16f,16h,18h^ B­(C_6_F_5_)_3_ has been used to form THQs in different and mechanistically distinct strategies,^19^ such as in intramolecular cyclizations.^20–23^ For example, Grimme and Paradies,^20^ and Wang^21^ reported the B­(C_6_F_5_)_3_-catalyzed intramolecular cyclization of *ortho*-vinyl-substituted *N*,*N*-dialkyl arylamines. Yang and Ma showed that a B­(C_6_F_5_)_3_/TMSOTf catalyst system mediates a Friedel–Crafts-like reaction between *N*-alkyl arylamines and electron-deficient alkynes to generate enones that cyclize via a [1,5]-hydride shift–intramolecular Mannich addition.^23^

We started with the coupling of arylamine **1a** with allyl silane **2a** with some trepidation as allyl silanes had not previously been used in oxidative Povarov reactions.^24^ In addition, only highly nucleophilic alkenes (e.g., enolates) have been coupled to iminium ions generated from B­(C_6_F_5_)_3_-mediated hydride abstraction.^16b,16f,16h,18h^ Using B­(C_6_F_5_)_3_ (10 mol %) and 1,2-dichloroethane (DCE) as the solvent, we found that product **3a** formed in 59% yield (Table 1, entry 1). Here, and elsewhere in this report, B­(C_6_F_5_)_3_ was formed *in situ* from H_2_O·B­(C_6_F_5_)_3_ (10 mol %) and Et_3_SiH (20 mol %).^25^ This procedure negates the need for a glovebox or separate purification of commercially available B­(C_6_F_5_)_3_. Therefore, B­(C_6_F_5_)_3_ can be used as received from the supplier, and all catalysts/reagents can be weighed in air on the open bench. A solvent screen (Table 1, entries 1–3 and SI) revealed DCE as the best solvent to investigate the use of 10 mol % of a basic additive, where 2,6-dichloropyridine increased the yield of **3a** to 67% (entry 4 and see SI for other additives and loadings). Upon using 3 equiv of alkene **2a** in conjunction with 2,6-dichloropyridine, the yield of **3a** rose to 76% (entry 5), and again to 83% when the temperature was increased to 120 °C using 1,2-dichlorobenzene (*o*-DCB) as the solvent (entry 6).

**Table 1:**
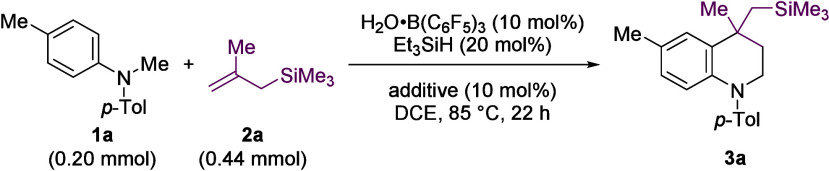
Reaction Optimization^a^

Entry	Modification	**3a** (%)
1	No additive	59
2	No additive, *o*-DCB as solvent	43
3	No additive, Me-THF as solvent	38
4	2,6-dichloropyridine additive	67
5	2,6-dichloropyridine additive, **2a** (3 equiv)	76
6	*o*-DCB as solvent, 2,6-dichloropyridine additive, **2a** (3 equiv), 120 °C	83

a Yields determined via ^1^H NMR analysis with an internal standard. DCE = 1,2-dichloroethane; *o*-DCB = 1,2-dichlorobenzene.

With a set of optimized conditions at hand, we explored the scope of *N*-alkyl arylamines **1** using allyl silane **2a** (Scheme 2). Diaryl methyl amines formed THQs **3a** and **3b**. Diaryl benzyl amines formed C2 aryl substituted THQs **3c**–**h** in approximately 1:1 dr. *N*-Benzyl and *N*-methyl dibenzoazepines formed tricyclic THQs **3h** and **3i**. *N*,*N*-Dimethyl amines were less efficient, for example, in THQs **3j**–**m** (15–37% yield). However, *N*,*N*-dibenzyl aryl amines performed better, and THQs **3n**–**3p** were formed in higher yields (43–56%), including 1-naphthylamine-derived THQ **3p**. In most cases, the 2,6-dichloropyridine additive was not required.

**Scheme 2:**
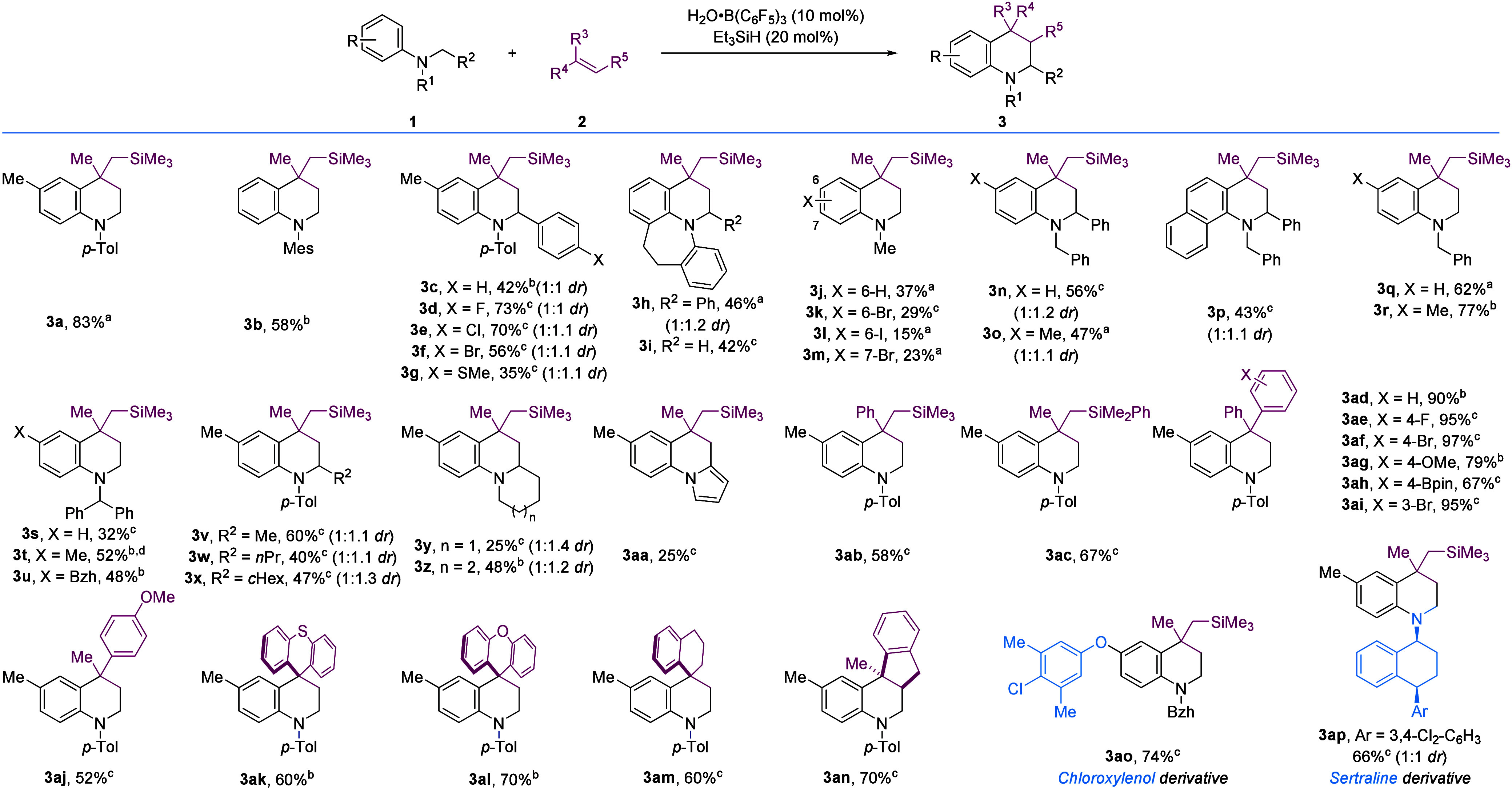
Scope of the B­(C_6_F_5_)_3_-Catalyzed Tetrahydroquinoline Synthesis

a
**1** (0.20 mmol), **2** (0.60 mmol), 2,6-dichloropyridine (10 mol %), *o*-DCB (0.50 mL), 22 h, 120 °C. Yields determined after ^1^H NMR analysis with an internal standard. All compounds were isolated to confirm identity. Diastereomeric ratios (dr) were determined by ^1^H NMR analysis of the crude reaction mixture.
b
**1** (0.20 mmol), **2** (0.44 mmol), DCE (0.50 mL), 22 h, 85 °C.
c
**1** (0.20 mmol), **2** (1.00 mmol), DCE (0.50 mL), 120 h, 85 °C.
d 37% isolated yield, 0.80 g.

Borane-mediated hydride abstraction can occur at a variety of α-nitrogen C–H bonds,^22^ including amino methyl, *n*-alkyl, and benzyl C–H bonds. Mixtures of products may result in substrates with nonequivalent α-nitrogen C–H bonds. However, *N*-methyl *N*-benzyl aryl amines and *N*-methyl *N*-benzhydryl (Bzh) aryl amines formed THQs **3q**–**u** where the methyl substituent was incorporated selectively into the ring. *N*-Methyl-*N*-ethyl- and *N*-methyl-*N*-hexyl arylamines were investigated, but they did not form THQ products (see SI).

We were also interested in going beyond *N*-methyl and *N*-benzyl arylamines so that the diversity in C2 substitution of the THQs could be increased. The generation of THQs bearing C2 alkyl substituents via oxidative (and nonoxidative) Povarov approaches is rare and limited to isolated examples that employ Michael acceptors.^11,12b,12f–12l^ This limitation arises due to the isomerization of aliphatic imines and iminiums that form enamines which in turn undergo side reactions (such as enamine–iminium self-condensation, Scheme 1B).^26^ Using the borane-catalyzed approach with *N*-ethyl, *N*-butyl, and *N*-cyclohexylmethylene amines, THQs with methyl, *n*-propyl, and cyclohexyl groups at the C2 position (**3v**–**3x**) were formed in 60%, 40%, and 47% yield, respectively. Common byproducts of self-condensation between iminium and enamine were not observed. In addition, *N*-aryl piperidine and azepine gave polycyclic THQs **3y** and **3z**. Interestingly, *N*-aryl pyrrolidine successfully underwent cyclization to form the THQ core in **3aa**, with subsequent conversion of the pyrrolidine ring into the corresponding pyrrole, analogous to our previous report of B­(C_6_F_5_)_3_-catalyzed pyrrolidine dehydrogenation.^18a^

1,4-Dihydroquinolines (DHQs) were not formed during the reactions, which was surprising given the efficiency that B­(C_6_F_5_)_3_ catalyzes the dehydrogenation of *N*-heterocycles.^16d,16e,18a^ In some cases, DHQs did form during purification on silica gel. Pure THQs were isolated in all cases, and DHQ formation could be minimized or avoided using short silica columns or purification via precipitation. In addition, conversion of DHQs into THQs is readily achieved using NaBH_4_ (see SI).

We examined different alkenes in the borane-catalyzed synthesis of THQs using amine **1a** (Scheme 2). In addition to trimethyl­(2-methylallyl)­silane (**2a**), trimethyl­(2-phenylallyl)­silane (**2b**), and dimethyl­(2-methylallyl)­(phenyl)­silane (**2c**) were successfully transformed into THQs **3ab** and **3ac**.

Styrene-derived alkenes were also of interest since their use in oxidative Povarov reactions is rare^15^ and limited to *N*-benzyl arylamines^13^ or *N*-aryl glycine derivatives^14^ that generate quinolines instead of THQs. A range of styrene-derived alkenes successfully formed novel THQ structures **3ad**–**3an** in the borane-catalyzed process. For example, 1,1-diaryl ethylenes gave 4,4-diaryl-substituted THQs **3ad**–**3ai** in excellent yields (67–97%). The monoaryl alkene, *para*-methoxy-α-methyl styrene, was converted into THQ **3aj** (52%). Novel spirocyclic THQs **3ak** (60%), **3al** (70%), and **3am** (60%) were formed from alkenes 9-methylenethioxanthene, 9-methylenexanthene, and 1-methylenetetralin, respectively. In addition, novel fused THQ **3an** was formed from 1-methyleneindan.^27^

Enol ethers, including silyl enol ethers, were also investigated but did not form the desired THQ products (see SI).

Across both amine and alkene scopes, a variety of versatile substituents and functional groups were tolerated including fluorine (**3d**, **3ae**), chlorine (**3e**, **3ao**, **3ap**), bromine (**3f**, **3k**, **3m**, **3af**, **3ai**), iodine (**3l**), pinacol borane (Bpin, **3ah**), ethers (**3ag**, **3aj**, **3al**), and thioethers (**3g**, **3ak**).

To further showcase the ability of the borane-catalyzed coupling of *N*-alkyl arylamines and alkenes to generate novel THQs, derivatives of the antiseptic chloroxylenol (*cf*. **3ao**, 74%) and the antidepressant sertraline (*cf*. **3ap**, 66%) were successfully converted into THQs.

With regard to mechanistic aspects, we observed the coupling products **4a** (26%) and **4b** (35%) in the reactions of *ortho*-substituted *N,N*-dimethyl arylamines **1ae** and **1ag** (Scheme 3A). In addition, monitoring reactions via *in situ* ^1^H NMR spectroscopy revealed the formation of alkanes (*cf*. **5**) as byproducts (see SI). Based on our experimental results and the literature,^28^ we propose the following catalytic cycle for the borane-catalyzed synthesis of THQs (Scheme 3B). The B­(C_6_F_5_)_3_ catalyst, formed *in situ*,^25^ abstracts a hydride from the α-nitrogen position of the *N*-alkyl arylamines **1** to give iminium borohydrides **6**.^16–18^

**Scheme 3:**
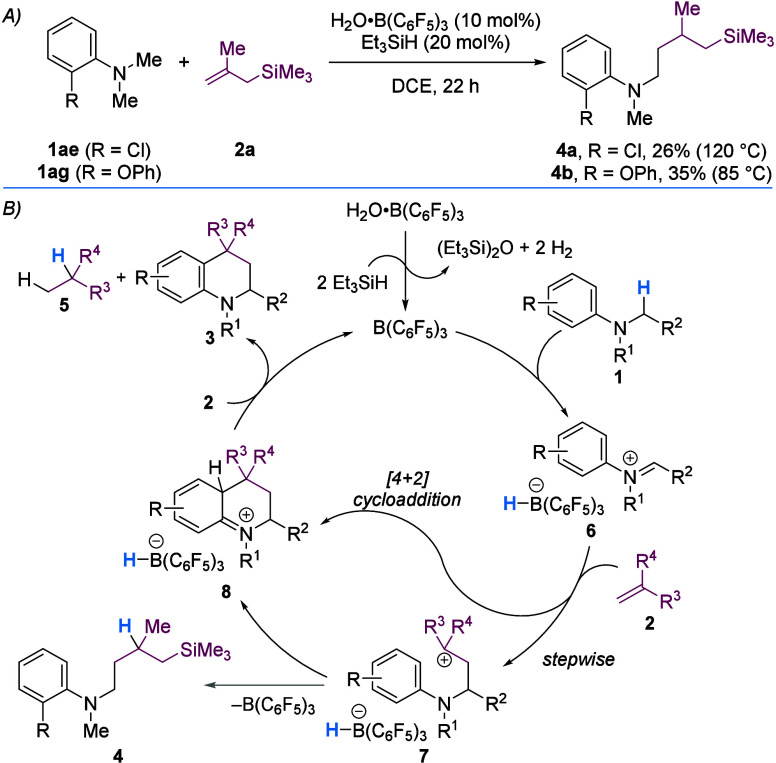
Proposed Catalytic Cycle

Analogous to the classic Povarov reaction, the coupling of the iminium and the alkene could proceed via a concerted or stepwise mechanism.^1,28^ A concerted [4 + 2] cycloaddition (or aza-Diels–Alder reaction) between **6** and alkene **2** would form intermediate **8**. Alternatively, a stepwise mechanism proceeds via alkene addition to electrophilic iminium **6** (Mannich-type addition), forming carbocation **7**, which then undergoes intramolecular Friedel–Crafts alkylation to form intermediate **8**. While neither mechanism has been ruled out, the stepwise process is possible given the formation of product **4**, where, presumably, hydride transfer from the borohydride anion to the carbocation **7** occurs. Analogous structures of **4** were not detected in other substrates. Regarding regeneration of the catalyst, we considered the borohydride anion acting as a base on intermediate **8** during rearomatization, releasing H_2_. However, the formation of alkanes **5** supports the regeneration of B­(C_6_F_5_)_3_ via transfer hydrogenation^16c,18a^ of alkenes **2**.

The role of the substoichiometric 2,6-dichloropyridine additive in the few cases it was used is unclear. A strong adduct does not form between 2,6-dichloropyridine and B­(C_6_F_5_)_3_ as shown by ^1^H, ^19^F, and ^11^B NMR spectroscopy, and there was little change in reaction performance with the loading of the additive (see SI). However, weak and hindered bases such as 2,6-dichloropyridine have been proposed to facilitate turnover in other B­(C_6_F_5_)_3_-catalyzed processes involving [arenium]­[HB­(C_6_F_5_)_3_] intermediates.^29^

To demonstrate the synthetic versatility of the novel THQ products formed herein, THQs **3r** and **3t** were subjected to selective oxidative and reductive processes (Scheme 4). Benzylic oxidation with 2,3-dichloro-5,6-dicyano-1,4-benzoquinone (DDQ) afforded aldehydes **9a** (81%) and **9b** (50%) that bear additional synthetic handles for further functionalization.^30^ In a complementary fashion, DHQ **10** was selectively accessed via BaMnO_4_-mediated oxidation.^31^ Furthermore, selective oxidation at the C2 position^32^ using I_2_ provided lactam **11** that contains a dihydroquinolinone core, a common motif found in drugs and natural products.^33^ Removal of benzyl or benzhydryl (Bzh) groups using Pd/C and HCO_2_NH_4_ gave *NH* THQ **12**.^34^ Finally, hydrogenation over Rh/C gave the saturated heterocycle, *cis*-decahydroquinoline **13** (3:1 dr) (Scheme 4).^35^

**Scheme 4:**
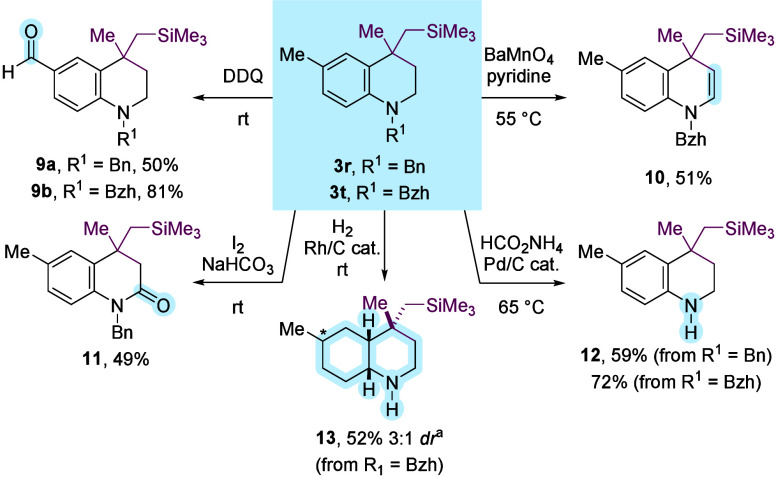
Derivatization of Novel Tetrahydroquinolines

a Ratio of major diastereomer versus all minor diastereomers. *Relative stereochemistry not determined at this position.

We have developed a convenient method for creating novel tetrahydroquinoline structures using an organoborane catalyst, B­(C_6_F_5_)_3_. This commercially available catalyst does not require use of a glovebox and directly activates *N*-alkyl aromatic amines by forming a key iminium intermediate without precious metal catalysts. The method uses common aromatic amines and alkenes, expanding the accessible chemical space of THQs, including 4,4-disubstituted, C2-alkyl, spirocyclic, and polycyclic motifs. It employs styrenes and, for the first time, allyl silanes in an oxidative Povarov reaction. The novel THQ products are amenable to further transformations, including complementary oxidations and reductions.

## Supplementary Material



## Data Availability

The data underlying this study are available in the published article and its Supporting Information.

## Notes

Experimental procedures, optimization reactions, and NMR spectra (PDF)
