# Supplementary material for: B(C6F5)3‑Catalyzed Coupling of N‑Alkyl Arylamines and Alkenes for the Synthesis of Tetrahydroquinolines
Source: Org Lett. 2025 Sep 3;27(36):9993–9. doi: 10.1021/acs.orglett.5c03023 (PMC12442061; doi:10.1021/acs.orglett.5c03023)

Supporting Information

$B(C_6F_5)_3$-Catalyzed coupling of *N*-alkyl arylamines and alkenes for the synthesis of tetrahydroquinolines

Sophia Moreen Gloria,[a] Laura Winfrey,[a] Yuncong Gao,[a] Maryia Barysevich,[a] Joseph P. Gillions,[a] Lei Yun,[a] Halima Patel,[a] Amy Shah,[a] Ana Alvarez-Montoya,[a] Dean Thomas,[b] Ahmad Khan,[b] Hassan Y. Harb,[c] Alison M. Stuart,[a] Alexander P. Pulis[a]*

[a]*School of Chemistry, University of Leicester, Leicester, LE1 7RH, UK.*

[b]*Department of Chemistry, University of Manchester, Manchester, M13 9PL, UK*

[c] Concept Life Sciences Ltd, Frith Knoll Road, Chapel-en-le-Frith, High Peak, SK23 0PG, UK

1 Supplementary Schemes 6

2 General Information 8

3 Experimental and Characterization Data 8

3.1 Synthesis of Starting Materials 8

3.1.1 Synthesis of Tertiary Amines 8

General Procedure 1: *N*-Alkylation of anilines using NaH 8

General Procedure 2: *N*-Alkylation of anilines using K_2CO_3 9

General Procedure 3: Reductive Amination of Diaryl Amines 9

General Procedure 4: *N*-Benzhydryl Protection of *N*-Methylaniline 10

N,4-Dimethyl-*N*-(*p*-tolyl)aniline, 1a 10

N,2,4,6-Tetramethyl-*N*-phenylaniline, 1b 11

N-Benzyl-4-methyl-*N*-(*p*-tolyl)aniline, 1c 11

N-(4-Fluorobenzyl)-4-methyl-*N*-(*p*-tolyl)aniline, 1d 12

N-(4-Chlorobenzyl)-4-methyl-*N*-(p-tolyl)aniline, 1e 12

N-(4-Bromobenzyl)-4-methyl-*N*-(*p*-tolyl)aniline, 1f 13

4-Methyl-*N*-(4-(methylthio)benzyl)-*N*-(*p*-tolyl)aniline, 1g 13

5-Benzyl-10,11-dihydro-5*H*-dibenzo[*b*,*f*]azepine, 1h 14

5-Methyl-10,11-dihydro-5*H*-dibenzo[*b*,*f*]azepine, 1i 14

N,*N*-Dibenzylaniline, 1n 14

N,*N*-Dibenzyl-4-methylaniline, 1o 15

N,*N*-Dibenzylnaphthalen-1-amine, 1p 15

N-Benzyl-*N*-methylaniline, 1q 16

N-Benzyl-*N*,4-dimethylaniline, 1r 16

N-Benzhydryl-*N*-methylaniline, 1s and *N*,4-dibenzhydryl-*N*-methylaniline, 1u 17

N-Benzhydryl-*N*,4-dimethylaniline, 1t 17

N-Ethyl-4-methyl-*N*-(*p*-tolyl)aniline, 1v 18

N-Butyl-4-methyl-*N*-(*p*-tolyl)aniline, 1w 18

N-(Cyclohexylmethyl)-4-methyl-*N*-(*p*-tolyl)aniline, 1x 19

1-(*p*-Tolyl)piperidine, 1y 19

1-(*p*-Tolyl)azepane, 1z ... 20
1-(*p*-Tolyl)pyrrolidine, 1aa ... 20
N-Benzhydryl-4-(4-chloro-3,5-dimethylphenoxy)-*N*-methylaniline, 1ab ... 21
(1*S*,4*S*)-4-(3,4-Dichlorophenyl)-*N*-methyl-*N*-(*p*-tolyl)-1,2,3,4-tetrahydronaphthalen-1-amine, 1ac ... 22
2-Chloro-*N*-methylaniline, 1ad and 2-chloro-*N*,*N*-dimethylaniline, 1ae ... 22
N-Methyl-2-phenoxyaniline, 1af and *N*,*N*-dimethyl-2-phenoxyaniline, 1ag ... 23
N-Ethyl-*N*,4-dimethylaniline, 1ah ... 23
N-Hexyl-*N*-methylaniline, 1ai ... 24

3.1.2 Synthesis of Alkenes ... 24

General Procedure 5: Wittig reactions of acetophenone derivatives ... 24
General Procedure 6: Formation of Alkenes from Ketones using MeLi ... 25
Phenyl trifluoromethanesulfonate ... 25
Trimethyl(2-phenylallyl)silane, 2b ... 26
Dimethyl(2-methylallyl)(phenyl)silane, 2c ... 26
1-Fluoro-4-(1-phenylvinyl)benzene, 2e ... 27
1-Bromo-4-(1-phenylvinyl)benzene, 2f ... 28
1-Methoxy-4-(1-phenylvinyl)benzene, 2g ... 28
4,4,5,5-Tetramethyl-2-(4-(1-phenylvinyl)phenyl)-1,3,2-dioxaborolane, 2h ... 29
1-Bromo-3-(1-phenylvinyl)benzene, 2i ... 29
1-Methoxy-4-(prop-1-en-2-yl)benzene, 2j ... 30
9-Methylene-9*H*-thioxanthene, 2k ... 30
9-Methylene-9*H*-xanthene, 2l ... 30
1-Methylene-1,2,3,4-tetrahydronaphthalene, 2m ... 31
1-Methylene-2,3-dihydro-1*H*-indene, 2n ... 31

3.2 Optimization Studies ... 32

General Procedure 7: Drying $B(C_6F_5)_3$ in situ ... 32
General Procedure 8: $B(C_6F_5)_3$-Catalysed Synthesis of Tetrahydroquinolines from Amines and Alkenes ... 32
General Procedure 9: $B(C_6F_5)_3$-Catalysed Synthesis of Tetrahydroquinolines from Amines and Alkenes with 2,6-Dichloropyridine ... 32

3.2.1 Solvent screen ... 33

Table 1. Solvent screen of tetrahydroquinoline synthesis with *N*-methyl-di-*p*-tolylamine 1a and methylallyltrimethylsilane 2a ... 33

3.2.2 Optimization of Condition B ... 33

Table 2. Screen of basic additives ... 33
Table 3. Screen of 2,6-disubstituted pyridines ... 34
Table 4. Equivalent screen of 2,6-dichloropyridine ... 34
Table 5. Equivalence screen of methylallyltrimethylsilane 2a ... 34
Table 6. Optimization of reaction parameters ... 35

3.2.3 Optimization of Condition C ... 35

Table 7. Equivalent screen of methylallyltrimethylsilane 2a ... 35
Table 8. Optimization of reaction parameters ... 36

3.3 Amine Scope of $B(C_6F_5)_3$-Catalysed THQ synthesis ... 36

4,6-Dimethyl-1-(*p*-tolyl)-4-((trimethylsilyl)methyl)-1,2,3,4-tetrahydroquinoline, 3a and 4,6-dimethyl-1-(*p*-tolyl)-4-((trimethylsilyl)methyl)-1,4-dihydroquinoline, 3a' ... 36
1-Mesityl-4-methyl-4-((trimethylsilyl)methyl)-1,2,3,4-tetrahydroquinoline, 3b ... 37
4,6-Dimethyl-2-phenyl-1-(*p*-tolyl)-4-((trimethylsilyl)methyl)-1,2,3,4-tetrahydroquinoline, 3c ... 38

2-(4-Fluorophenyl)-4,6-dimethyl-1-(*p*-tolyl)-4-((trimethylsilyl)methyl)-1,2,3,4-tetrahydroquinoline, 3d 39
2-(4-Chlorophenyl)-4,6-dimethyl-1-(*p*-tolyl)-4-((trimethylsilyl)methyl)-1,2,3,4-tetrahydroquinoline, 3e 40
2-(4-Bromophenyl)-4,6-dimethyl-1-(*p*-tolyl)-4-((trimethylsilyl)methyl)-1,2,3,4-tetrahydroquinoline, 3f 41
4,6-Dimethyl-2-(4-(methylthio)phenyl)-1-(*p*-tolyl)-4-((trimethylsilyl)methyl)-1,2,3,4-tetrahydroquinoline, 3g 42
3-Methyl-1-phenyl-3-((trimethylsilyl)methyl)-2,3,7,8-tetrahydro-1*H*-benzo[6,7]azepino[3,2,1-ij]quinoline, 3h 43
3-Methyl-3-((trimethylsilyl)methyl)-2,3,7,8-tetrahydro-1*H*-benzo[6,7]azepino[3,2,1-ij]quinoline, 3i 44
1,4-Dimethyl-4-((trimethylsilyl)methyl)-1,2,3,4-tetrahydroquinoline, 3j 44
6-Bromo-1,4-dimethyl-4-((trimethylsilyl)methyl)-1,2,3,4-tetrahydroquinoline, 3k 45
6-Iodo-1,4-dimethyl-4-((trimethylsilyl)methyl)-1,2,3,4-tetrahydroquinoline, 3l 46
7-Bromo-1,4-dimethyl-4-((trimethylsilyl)methyl)-1,2,3,4-tetrahydroquinoline, 3m 46
1-Benzyl-4-methyl-2-phenyl-4-((trimethylsilyl)methyl)-1,2,3,4-tetrahydroquinoline, 3n 47
1-Benzyl-4,6-dimethyl-2-phenyl-4-((trimethylsilyl)methyl)-1,2,3,4-tetrahydroquinoline, 3o 48
1-Benzyl-4-methyl-2-phenyl-4-((trimethylsilyl)methyl)-1,2,3,4-tetrahydrobenzo[*h*]quinoline, 3p 49
1-Benzyl-4-methyl-4-((trimethylsilyl)methyl)-1,2,3,4-tetrahydroquinoline, 3q 50
1-Benzyl-4,6-dimethyl-4-((trimethylsilyl)methyl)-1,2,3,4-tetrahydroquinoline, 3r 50
1-Benzhydryl-4-methyl-4-((trimethylsilyl)methyl)-1,2,3,4-tetrahydroquinoline, 3s 51
1-Benzhydryl-4,6-dimethyl-4-((trimethylsilyl)methyl)-1,2,3,4-tetrahydroquinoline, 3t 51
1,6-Dibenzhydryl-4-methyl-4-((trimethylsilyl)methyl)-1,2,3,4-tetrahydroquinoline, 3u 52
2,4,6-Trimethyl-1-(*p*-tolyl)-4-((trimethylsilyl)methyl)-1,2,3,4-tetrahydroquinoline, 3v 53
4,6-Dimethyl-2-propyl-1-(*p*-tolyl)-4-((trimethylsilyl)methyl)-1,2,3,4-tetrahydroquinoline, 3w 54
2-Cyclohexyl-4,6-dimethyl-1-(*p*-tolyl)-4-((trimethylsilyl)methyl)-1,2,3,4-tetrahydroquinoline, 3x 55
6,8-Dimethyl-6-((trimethylsilyl)methyl)-2,3,4,4a,5,6-hexahydro-1*H*-pyrido[1,2-*a*]quinoline, 3y 56
3,5-Dimethyl-5-((trimethylsilyl)methyl)-5,6,6a,7,8,9,10,11-octahydroazepino[1,2-*a*]quinoline, 3z 57
5,7-Dimethyl-5-((trimethylsilyl)methyl)-4,5-dihydropyrrolo[1,2-*a*]quinoline, 3aa 58

3.4 Alkene Scope of B(C_6F_5)$_3$-Catalysed THQ synthesis 59

6-Methyl-4-phenyl-1-(*p*-tolyl)-4-((trimethylsilyl)methyl)-1,2,3,4-tetrahydroquinoline, 3ab 59
4-((Dimethyl(phenyl)silyl)methyl)-4,6-dimethyl-1-(*p*-tolyl)-1,2,3,4-tetrahydroquinoline, 3ac and 4-((dimethyl(phenyl)silyl)methyl)-4,6-dimethyl-1-(*p*-tolyl)-1,4-dihydroquinoline, 3ac' 60
6-Methyl-4,4-diphenyl-1-(*p*-tolyl)-1,2,3,4-tetrahydroquinoline, 3ad and 6-methyl-4,4-diphenyl-1-(*p*-tolyl)-1,4-dihydroquinoline, 3ad' 61
4-(4-Fluorophenyl)-6-methyl-4-phenyl-1-(*p*-tolyl)-1,2,3,4-tetrahydroquinoline, 3ae and 4-(4-fluorophenyl)-6-methyl-4-phenyl-1-(*p*-tolyl)-1,4-dihydroquinoline, 3ae' 62
4-(4-Bromophenyl)-6-methyl-4-phenyl-1-(*p*-tolyl)-1,2,3,4-tetrahydroquinoline, 3af and 4-(4-bromophenyl)-6-methyl-4-phenyl-1-(*p*-tolyl)-1,4-dihydroquinoline, 3af' 63
4-(4-Methoxyphenyl)-6-methyl-4-phenyl-1-(*p*-tolyl)-1,2,3,4-tetrahydroquinoline, 3ag and 4-(4-methoxyphenyl)-6-methyl-4-phenyl-1-(*p*-tolyl)-1,4-dihydroquinoline, 3ag' 64
6-Methyl-4-phenyl-4-(4-(4,4,5,5-tetramethyl-1,3,2-dioxaborolan-2-yl)phenyl)-1-(*p*-tolyl)-1,2,3,4-tetrahydroquinoline, 3ah 65
4-(3-Bromophenyl)-6-methyl-4-phenyl-1-(*p*-tolyl)-1,2,3,4-tetrahydroquinoline, 3ai and 4-(3-bromophenyl)-6-methyl-4-phenyl-1-(*p*-tolyl)-1,4-dihydroquinoline, 3ai' 66
4-(4-Methoxyphenyl)-4,6-dimethyl-1-(*p*-tolyl)-1,2,3,4-tetrahydroquinoline, 3aj 67
6-Methyl-1-(*p*-tolyl)-2,3-dihydro-1*H*-spiro[quinoline-4,9'-thioxanthene], 3ak 67
6-Methyl-1-(*p*-tolyl)-2,3-dihydro-1*H*-spiro[quinoline-4,9'-xanthene], 3al 68
6'-Methyl-1'-(*p*-tolyl)-2',3,3',4-tetrahydro-1'*H*,2*H*-spiro[naphthalene-1,4'-quinoline], 3am and 6'-methyl-1'-(*p*-tolyl)-3,4-dihydro-1'*H*,2*H*-spiro[naphthalene-1,4'-quinoline], 3am' 69
2,11b-Dimethyl-5-(*p*-tolyl)-6,6a,7,11b-tetrahydro-5*H*-indeno[2,1-c]quinoline, 3an 70

3.5 Drug Derivatives in B(C_6F_5)$_3$-Catalyzed THQ synthesis 70

1-Benzhydryl-6-(4-chloro-3,5-dimethylphenoxy)-4-methyl-4-((trimethylsilyl)methyl)-1,2,3,4-tetrahydroquinoline, 3ao 70
1-((4*S*)-4-(3,4-Dichlorophenyl)-1,2,3,4-tetrahydronaphthalen-1-yl)-4,6-dimethyl-4-((trimethylsilyl)methyl)-1,2,3,4-tetrahydroquinoline, 3ap 71

3.6 Reduction of DHQs 72

General Procedure 10: Reduction of dihydroquinolines (DHQs) with $NaBH_4$ 72

6-Methyl-4,4-diphenyl-1-(*p*-tolyl)-1,2,3,4-tetrahydroquinoline, 3ad 72

4-(4-Bromophenyl)-6-methyl-4-phenyl-1-(*p*-tolyl)-1,2,3,4-tetrahydroquinoline, 3af 72

4-(3-Bromophenyl)-6-methyl-4-phenyl-1-(*p*-tolyl)-1,2,3,4-tetrahydroquinoline, 3ai 73

3.7 Mechanistic studies 73

3.7.1 Uncyclised Products 4 73

2-Chloro-*N*-methyl-*N*-(3-methyl-4-(trimethylsilyl)butyl)aniline, 4a 73

N-Methyl-*N*-(3-methyl-4-(trimethylsilyl)butyl)-2-phenoxyaniline, 4b 74

3.7.2 NMR Experiments 74

In Situ NMR Experiments Observing Transfer Hydrogenation of Alkenes 2a and 2d 74

NMR Experiments of 2,6-Dichloropyridine and $B(C_6F_5)_3$ 76

3.8 Derivatization of Tetrahydroquinolines 3r and 3t 78

1-Benzyl-4-methyl-4-((trimethylsilyl)methyl)-1,2,3,4-tetrahydroquinoline-6-carbaldehyde, 9a 78

1-Benzhydryl-4-methyl-4-((trimethylsilyl)methyl)-1,2,3,4-tetrahydroquinoline-6-carbaldehyde, 9b 79

1-Benzhydryl-4,6-dimethyl-4-((trimethylsilyl)methyl)-1,4-dihydroquinoline, 10 80

1-Benzyl-4,6-dimethyl-4-((trimethylsilyl)methyl)-3,4-dihydroquinolin-2(1*H*)-one, 11 80

4,6-Dimethyl-4-((trimethylsilyl)methyl)-1,2,3,4-tetrahydroquinoline, 12 81

(4*RS*,4a*RS*,8a*RS*)-4,6-Dimethyl-4-((trimethylsilyl)methyl)decahydroquinoline, 13 81

4 References 82

5 NMR Spectra 84

N-(4-Fluorobenzyl)-4-methyl-*N*-(*p*-tolyl)aniline, 1d 84

N-(4-Chlorobenzyl)-4-methyl-*N*-(p-tolyl)aniline, 1e 85

N-(4-Bromobenzyl)-4-methyl-*N*-(*p*-tolyl)aniline, 1f 86

4-Methyl-*N*-(4-(methylthio)benzyl)-*N*-(*p*-tolyl)aniline, 1g 87

N,4-Dibenzhydryl-*N*-methylaniline, 1u 88

N-Benzhydryl-*N*,4-dimethylaniline, 1t 89

N-Ethyl-4-methyl-*N*-(*p*-tolyl)aniline, 1v 91

N-Butyl-4-methyl-*N*-(*p*-tolyl)aniline, 1w 93

N-Benzhydryl-4-(4-chloro-3,5-dimethylphenoxy)-*N*-methylaniline, 1ab 94

(1*S*,4*S*)-4-(3,4-Dichlorophenyl)-*N*-methyl-*N*-(*p*-tolyl)-1,2,3,4-tetrahydronaphthalen-1-amine, 1ac 95

N,*N*-Dimethyl-2-phenoxyaniline, 1ag 97

1-Methylene-2,3-dihydro-1*H*-indene, 2n 98

4,6-Dimethyl-1-(*p*-tolyl)-4-((trimethylsilyl)methyl)-1,2,3,4-tetrahydroquinoline, 3a 100

4,6-Dimethyl-1-(*p*-tolyl)-4-((trimethylsilyl)methyl)-1,4-dihydroquinoline, 3a' 102

1-Mesityl-4-methyl-4-((trimethylsilyl)methyl)-1,2,3,4-tetrahydroquinoline, 3b 104

4,6-Dimethyl-2-phenyl-1-(*p*-tolyl)-4-((trimethylsilyl)methyl)-1,2,3,4-tetrahydroquinoline, 3c 105

2-(4-Fluorophenyl)-4,6-dimethyl-1-(*p*-tolyl)-4-((trimethylsilyl)methyl)-1,2,3,4-tetrahydroquinoline, 3d 1:1.7 *dr* 107

2-(4-Bromophenyl)-4,6-dimethyl-1-(*p*-tolyl)-4-((trimethylsilyl)methyl)-1,2,3,4-tetrahydroquinoline, 3f 1:2 *dr* 111

4,6-Dimethyl-2-(4-(methylthio)phenyl)-1-(*p*-tolyl)-4-((trimethylsilyl)methyl)-1,2,3,4-tetrahydroquinoline, 3g 1:1.1 *dr* 113

3-Methyl-1-phenyl-3-((trimethylsilyl)methyl)-2,3,7,8-tetrahydro-1*H*-benzo[6,7]azepino[3,2,1-ij]quinoline, 3h 115

3-Methyl-3-((trimethylsilyl)methyl)-2,3,7,8-tetrahydro-1*H*-benzo[6,7]azepino[3,2,1-ij]quinoline, 3i 117

1,4-Dimethyl-4-((trimethylsilyl)methyl)-1,2,3,4-tetrahydroquinoline, 3j 119

6-Bromo-1,4-dimethyl-4-((trimethylsilyl)methyl)-1,2,3,4-tetrahydroquinoline, 3k 121

6-Iodo-1,4-dimethyl-4-((trimethylsilyl)methyl)-1,2,3,4-tetrahydroquinoline, 3l 123

7-Bromo-1,4-dimethyl-4-((trimethylsilyl)methyl)-1,2,3,4-tetrahydroquinoline, 3m 125
1-Benzyl-4-methyl-2-phenyl-4-((trimethylsilyl)methyl)-1,2,3,4-tetrahydroquinoline, 3n 1:1.5 *dr* 127
1-Benzyl-4,6-dimethyl-2-phenyl-4-((trimethylsilyl)methyl)-1,2,3,4-tetrahydroquinoline 3o 129
1-Benzyl-4-methyl-2-phenyl-4-((trimethylsilyl)methyl)-1,2,3,4-tetrahydrobenzo[h]quinoline, 3p 1:1.4 *dr* 131
1-Benzyl-4-methyl-4-((trimethylsilyl)methyl)-1,2,3,4-tetrahydroquinoline, 3q 133
1-Benzyl-4,6-dimethyl-4-((trimethylsilyl)methyl)-1,2,3,4-tetrahydroquinoline, 3r 135
1-Benzhydryl-4-methyl-4-((trimethylsilyl)methyl)-1,2,3,4-tetrahydroquinoline, 3s 136
1-Benzhydryl-4,6-dimethyl-4-((trimethylsilyl)methyl)-1,2,3,4-tetrahydroquinoline, 3t 138
1,6-Dibenzhydryl-4-methyl-4-((trimethylsilyl)methyl)-1,2,3,4-tetrahydroquinoline, 3u 140
2,4,6-Trimethyl-1-(*p*-tolyl)-4-((trimethylsilyl)methyl)-1,2,3,4-tetrahydroquinoline, 3v 142
4,6-Dimethyl-2-propyl-1-(*p*-tolyl)-4-((trimethylsilyl)methyl)-1,2,3,4-tetrahydroquinoline, 3w 1:1.3 *dr* 146
2-Cyclohexyl-4,6-dimethyl-1-(*p*-tolyl)-4-((trimethylsilyl)methyl)-1,2,3,4-tetrahydroquinoline, 3x 148
6,8-Dimethyl-6-((trimethylsilyl)methyl)-2,3,4,4a,5,6-hexahydro-1*H*-pyrido[1,2-*a*]quinoline, 3y 150
3,5-Dimethyl-5-((trimethylsilyl)methyl)-5,6,6a,7,8,9,10,11-octahydroazepino[1,2-*a*]quinoline, 3z 154
5,7-Dimethyl-5-((trimethylsilyl)methyl)-4,5-dihydropyrrolo[1,2-*a*]quinoline, 3aa 158
6-Methyl-4-phenyl-1-(*p*-tolyl)-4-((trimethylsilyl)methyl)-1,2,3,4-tetrahydroquinoline, 3ab 160
4-((Dimethyl(phenyl)silyl)methyl)-4,6-dimethyl-1-(*p*-tolyl)-1,2,3,4-tetrahydroquinoline, 3ac 162
4-((Dimethyl(phenyl)silyl)methyl)-4,6-dimethyl-1-(*p*-tolyl)-1,4-dihydroquinoline, 3ac' 163
6-Methyl-4,4-diphenyl-1-(*p*-tolyl)-1,2,3,4-tetrahydroquinoline, 3ad 165
6-Methyl-4,4-diphenyl-1-(*p*-tolyl)-1,4-dihydroquinoline, 3ad' 167
4-(4-Fluorophenyl)-6-methyl-4-phenyl-1-(*p*-tolyl)-1,2,3,4-tetrahydroquinoline, 3ae 169
4-(4-Fluorophenyl)-6-methyl-4-phenyl-1-(*p*-tolyl)-1,4-dihydroquinoline, 3ae' 171
4-(4-Bromophenyl)-6-methyl-4-phenyl-1-(*p*-tolyl)-1,2,3,4-tetrahydroquinoline, 3af 173
4-(4-Bromophenyl)-6-methyl-4-phenyl-1-(*p*-tolyl)-1,4-dihydroquinoline, 3af' 175
4-(4-Methoxyphenyl)-6-methyl-4-phenyl-1-(*p*-tolyl)-1,2,3,4-tetrahydroquinoline, 3ag 177
4-(4-Methoxyphenyl)-6-methyl-4-phenyl-1-(*p*-tolyl)-1,4-dihydroquinoline, 3ag' 179
6-Methyl-4-phenyl-4-(4-(4,4,5,5-tetramethyl-1,3,2-dioxaborolan-2-yl)phenyl)-1-(p-tolyl)-1,2,3,4-tetrahydroquinoline, 3ah 181
4-(3-Bromophenyl)-6-methyl-4-phenyl-1-(*p*-tolyl)-1,2,3,4-tetrahydroquinoline, 3ai 183
4-(3-Bromophenyl)-6-methyl-4-phenyl-1-(*p*-tolyl)-1,4-dihydroquinoline, 3ai' 185
4-(4-Methoxyphenyl)-4,6-dimethyl-1-(*p*-tolyl)-1,2,3,4-tetrahydroquinoline, 3aj 187
6-Methyl-1-(*p*-tolyl)-2,3-dihydro-1*H*-spiro[quinoline-4,9'-thioxanthene], 3ak 189
6-Methyl-1-(*p*-tolyl)-2,3-dihydro-1*H*-spiro[quinoline-4,9'-xanthene], 3al 190
6'-Methyl-1'-(*p*-tolyl)-2',3,3',4-tetrahydro-1'*H*,2*H*-spiro[naphthalene-1,4'-quinoline], 3am 192
6'-Methyl-1'-(*p*-tolyl)-3,4-dihydro-1'*H*,2*H*-spiro[naphthalene-1,4'-quinoline], 3am' 194
2,11b-Dimethyl-5-(*p*-tolyl)-6,6a,7,11b-tetrahydro-5*H*-indeno[2,1-c]quinoline, 3an 195
1-Benzhydryl-6-(4-chloro-3,5-dimethylphenoxy)-4-methyl-4-((trimethylsilyl)methyl)-1,2,3,4-tetrahydroquinoline, 3ao 197
1-((4*S*)-4-(3,4-Dichlorophenyl)-1,2,3,4-tetrahydronaphthalen-1-yl)-4,6-dimethyl-4-((trimethylsilyl)methyl)-1,2,3,4-tetrahydroquinoline, 3ap 1:5 *dr* 199
2-Chloro-*N*-methyl-*N*-(3-methyl-4-(trimethylsilyl)butyl)aniline, 4a 201
N-Methyl-*N*-(3-methyl-4-(trimethylsilyl)butyl)-2-phenoxyaniline, 4b 203
1-Benzyl-4-methyl-4-((trimethylsilyl)methyl)-1,2,3,4-tetrahydroquinoline-6-carbaldehyde, 9a 205
1-Benzhydryl-4-methyl-4-((trimethylsilyl)methyl)-1,2,3,4-tetrahydroquinoline-6-carbaldehyd, 9b 207
1-Benzhydryl-4,6-dimethyl-4-((trimethylsilyl)methyl)-1,4-dihydroquinolin, 10 209
1-Benzyl-4,6-dimethyl-4-((trimethylsilyl)methyl)-3,4-dihydroquinolin-2(1*H*)-one, 11 211
4,6-Dimethyl-4-((trimethylsilyl)methyl)-1,2,3,4-tetrahydroquinoline, 12 213
(4*RS*,4a*RS*,8a*RS*)-4,6-Dimethyl-4-((trimethylsilyl)methyl)decahydroquinoline, 13 215

1 Supplementary Schemes

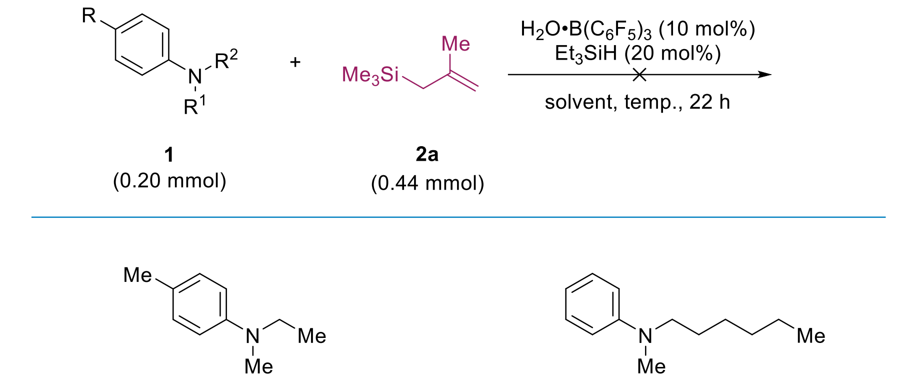

1ah

General procedure 8 (22 h at 85 °C, DCE):
86% **1ah** returned
no THQ products detected by GCMS

General procedure 9 (22 h at 120 °C 0-DCB):
86% **1ah** returned
no THQ products detected by GCMS

1ai

General procedure 8 (22 h at 85 °C, DCE):
42% **1ai** returned
complex mixture
trace THQ products detected by GCMS

Scheme S1. Failed amines in the $B(C_6F_5)_3$-catalyzed coupling of alkenes and *N*-alkyl amines.

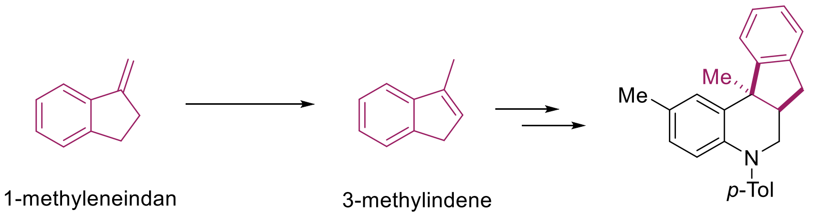

3an

Scheme S2. Proposed isomerisation of 1-methyleneindan to 3-methylindene in the formation of THQ **3an**.

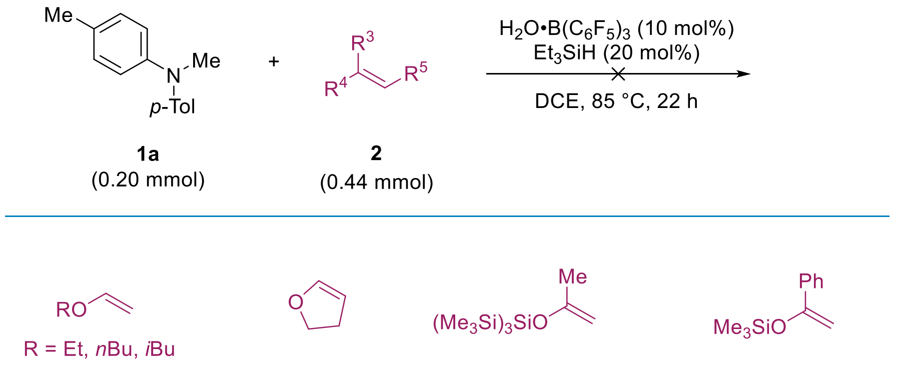

Scheme S3. Failed alkenes in the $B(C_6F_5)_3$-catalyzed coupling of alkenes and *N*-alkyl amines using General Procedure 8. No THQ products were observed, with >90% **1a** returned. Evidence of alkene **2** hydrogenation observed in each case, except for tri(trimethylsilyl) enol ether which formed a complex mixture.

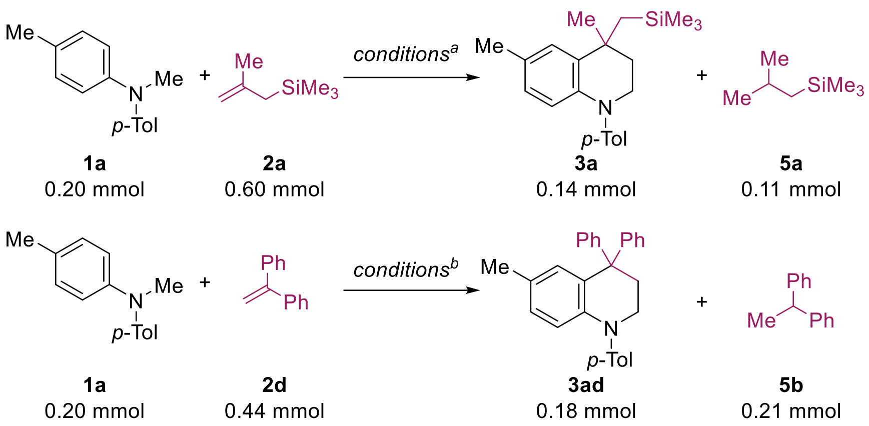

Scheme S4. Observation of hydrogenated alkenes via in situ ^{1}H-NMR analysis of reaction mixtures. Yields determined after ^{1}H-NMR analysis with an internal standard. [a]$B(C_6F_5)_3$ (10 mol%), 2,6-dichloropyridine (10 mol%), *o*-DCB, 120 °C, 22 h. [b]$B(C_6F_5)_3$ (10 mol%), DCE, 85 °C, 22 h.

2 General Information

Reagents were purchased from commercial suppliers and used without further purification unless stated otherwise. $B(C_6F_5)_3$ was purchased from Accela Chembio, supplied in hydrated form, and used without further purification, where the water was removed in situ as described below. Where reactions required an inert atmosphere, a Schlenk line and standard syringe-septa techniques were employed using oven-dried glassware. 1,2-Dichloroethane (DCE) was treated with microwave-activated molecular sieves for 3 days (3Å, 8-12 mesh) then vacuum transferred off the molecular sieves before it was transferred to an oven-dried Schlenk flash *via* typical syringe-septa techniques and stored under nitrogen. 1,2-Dichlorobenzene (*o*-DCB) was treated with microwave-activated molecular sieves for 3 days (3Å, 8-12 mesh) and then freeze-pump-thaw degassed. All other anhydrous solvents were used as received from commercial suppliers with no further drying. Unless otherwise stated, all reactions were heated in a stirred oil bath.

Flash column chromatography was performed using silica gel 60 (Fisher Scientific) and the stated eluent. Where possible, diastereoisomers were separated and independently characterised. In some cases, this was not possible, and they are presented as mixtures. TLC was performed on silica TLC plates (Merck-Millipore 60 F254) with a selected solvent system and spots were observed using UV fluorescence (254 nm) then developed with potassium permanganate or I_2. Mass spectra were obtained from a Micromass Quattro LC Spectrometer (electrospray). High resolution mass spectrometry (HRMS) was carried out on a Waters Acquity XEVO Q ToF spectrometer (electrospray). Peaks are reported in units of mass to charge ratio (m/z). ^{1}H and ^{13}C NMR spectra were obtained using Bruker 400 MHz and Bruker 500 MHz spectrometers at ambient temperatures unless otherwise stated. Chemical shifts (δ) are quoted in parts per million (ppm) and coupling constants (*J*) are in hertz (Hz). Residual solvent peaks were used as the internal reference for proton and carbon chemical shifts. Nitromethane was used as an internal standard to determine spectroscopic yields.

3 Experimental and Characterization Data

3.1 Synthesis of Starting Materials

3.1.1 Synthesis of Tertiary Amines

General Procedure 1: *N*-Alkylation of anilines using NaH

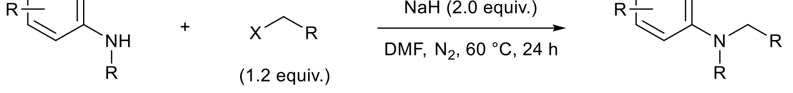

1

Following an adapted literature procedure,[1] an oven-dried RBF equipped with a magnetic stir bar was cooled under vacuum and charged with N_2 gas. NaH (2.0 equiv., 60% in mineral oil) was added to the RBF and the atmosphere cycled three times via vacuum-N_2 backfills. Using standard syringe-septa

techniques, DMF (10 mL) was added and allowed to stir. In a separate vial, aniline (5.0 mmol, 1.0 equiv.) was added, sealed with a septa, and the atmosphere replaced with N_2. Dry DMF (3 mL) was added, and the amine solution was added to the stirred NaH using standard syringe-septa techniques. The vial was washed with further DMF (2 mL) and added to the reaction mixture. The mixture was allowed to stir for 10 mins. Benzyl halide (1.2 equiv.) was dissolved in DMF (5 mL) before it was added to the reaction mixture slowly. The reaction was stirred at 60 °C for 24 h, after which time the reaction was cooled to r.t. before the slow addition of H_2O (30 mL) and EtOAc (20 mL). The phases were separated, and the aqueous phase was extracted with EtOAc (50 mL × 3). The combined organic phases were washed with water (30 mL), brine (30 mL) and dried over $MgSO_4$. After filtration, the solvent was removed *in vacuo* and the crude product was purified via column chromatography on silica gel to obtain the pure alkylated aniline **1**.

General Procedure 2: *N*-Alkylation of anilines using K_2CO_3

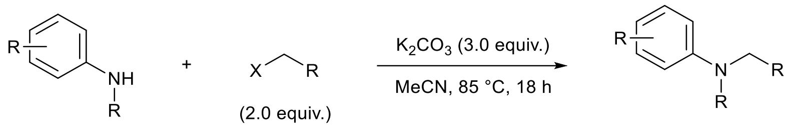

1

Following an adapted literature procedure,[1] to an RBF equipped with a reflux condenser and magnetic stir bar was charged with aniline (15 mmol, 1.0 equiv.), K_2CO_3 (3.0 equiv.), haloalkane (1.5-2.0 equiv.) and MeCN (20 mL), After being heated to 85 °C for 18 h, the reaction was cooled to r.t. and H_2O (30 mL) and EtOAc (20 mL) were added. The phases were separated, and the aqueous phase was extracted with EtOAc (50 mL × 3). The combined organic phases were washed with water (30 mL), brine (30 mL) and dried over $MgSO_4$. After filtration, the solvent was removed *in vacuo* and the crude product was purified via column chromatography on silica gel to obtain the pure alkylated aniline **1**.

General Procedure 3: Reductive Amination of Diaryl Amines

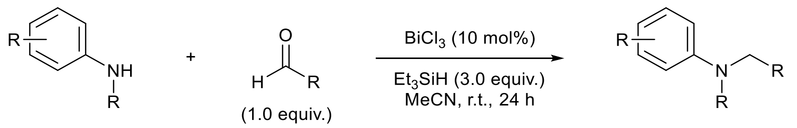

1

Following a literature procedure,[2] to an oven-dried 3-necked RBF equipped with a magnetic stir bar and charged with N_2 gas, was added $BiCl_3$ (10 mol%), Et_3SiH (3.0 equiv.) and MeCN (10 mL). This was allowed to stir for 10 mins. Amine (1.0 mmol, 1.0 equiv.) and aldehyde (1.0 equiv.) were sealed in a vial with a septa and the atmosphere replaced with N_2. MeCN (5 mL) was added and the solution was added to the stirred catalyst using standard syringe-septa techniques. The vial was washed with further MeCN (5 mL) and added to the reaction mixture. The reaction was stirred at r.t. for 24 h before $NaHCO_3$ (20 mL) and DCM (10 mL) were added. The phases were separated, and the aqueous phase was extracted with DCM (50 mL × 3). The combined organic phases were washed with water (20 mL), brine

(20 mL) and dried over $MgSO_4$. After filtration, the solvent was removed *in vacuo* and the crude product was purified via column chromatography on silica gel to obtain the pure *N*-alkylated amine **1**.

General Procedure 4: *N*-Benzhydryl Protection of *N*-Methylaniline

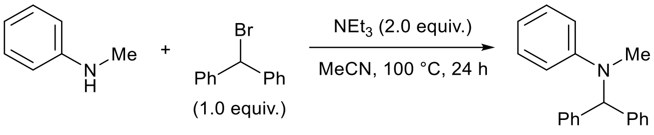

1

To an oven-dried RBF equipped with a magnetic stir bar was cooled under vacuum and charged with N_2 gas. Aniline (9.3 mmol, 1.0 equiv) was added to the RBF and the atmosphere cycled three times via vacuum-N_2 backfills. Using standard syringe-septa techniques, Et_3N (2.0 equiv.) in MeCN (10 mL) was added. This was stirred for 10 mins before benzhydryl bromide (1.0 equiv.) was added. The reaction was stirred at 100 °C for 24 h before H_2O (20 mL) and EtOAc (20 mL) were added. The phases were separated, and the aqueous phase was extracted with EtOAc (50 mL × 3). The combined organic phases were washed with water (30 mL), brine (30 mL) and dried over $MgSO_4$. After filtration, the solvent was removed *in vacuo* and the crude product was purified via column chromatography on silica gel to obtain the pure *N*-benzhydryl protected aniline **1**.

***N*,4-Dimethyl-*N*-(*p*-tolyl)aniline, 1a**

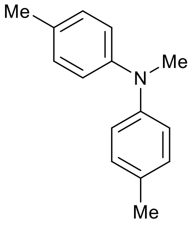

The title compound was prepared according to General Procedure 1, using di-*p*-tolylamine (0.50 g, 2.5 mmol), NaH (0.20 g, 5.1 mmol, 60% in mineral oil), iodomethane (0.38 mL, 3.0 mmol) in DMF (5 mL). Purification by column chromatography on silica gel (eluent = 10% DCM/petroleum ether), gave the title compound **1a** as a white solid (0.43 g, 2.1 mmol, 84%).

R_f = 0.3 (10% DCM/petroleum ether). ^1H NMR (500 MHz, $CDCl_3$) δ_H 7.10-7.06 (4H, m, ArH), 6.94-6.90 (4H, m, ArH), 3.27 (3H, s, NCH_3), 2.31 (6H, s, CH_3); $^{13}C\{^1H\}$ NMR ($CDCl_3$, 101 MHz) δ_C 147.0 (C), 130.4 (C), 129.7 (CH), 120.3 (CH), 40.5 (NCH_3), 20.6 (CH_3). HRMS (ESI$^+$) m/z: calcd for $C_{15}H_{18}N$ $[M+H]^+$ 212.1434; found 212.1436. This data is in agreement with the literature.[1]

N,2,4,6-Tetramethyl-N-phenylaniline, 1b

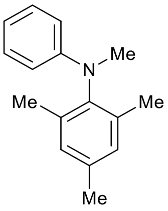

The title compound was prepared according to General Procedure 1, using 2,4,6-trimethyl-*N*-phenylaniline (2.8 g, 13 mmol), NaH (1.1 g, 27 mmol, 60% in mineral oil), iodomethane (3.3 mL, 54 mol) in THF (25 mL). Purification by column chromatography on silica gel (eluent = 3% DCM/petroleum ether), gave the title compound **1b** as a clear oil (1.3 g, 5.9 mmol, 44%).

R_f = 0.2 (3% DCM/petroleum ether). ^{1}H NMR (400 MHz, $CDCl_3$) δ_H H 7.17 (2H, br s, ArH), 6.96 (2H, s, ArH), 6.66 (1H, t, J = 8.3 Hz, ArH), 6.41 (2H, br s, ArH), 3.18 (3H, s, NCH_3), 2.33 (3H, s, CH_3), 2.06 (6H, s, CH_3); ^{13}C{^{1}H} NMR ($CDCl_3$, 101 MHz) δ_C 148.3 (C), 141.5 (C), 137.4 (CH), 136.4 (C), 129.5 (CH), 129.1 (CH), 115.7 (CH), 110.8 (C), 37.1 (NCH_3), 21.0 (CH_3), 17.8 (CH_3). HRMS (ESI$^+$) m/z: calcd for $C_{16}H_{20}N$ $[M+H]^+$ 226.1590; found 226.1594. This data is in agreement with the literature.[3]

***N*-Benzyl-4-methyl-*N*-(*p*-tolyl)aniline, 1c**

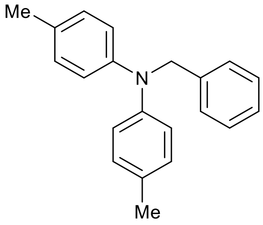

The title compound was prepared according to General Procedure 1, using di-*p*-tolylamine (1.00 g, 5.07 mmol), NaH (0.41 g, 10 mmol, 60% in mineral oil), benzyl bromide (0.72 mL, 6.1 mmol) in DMF (5 mL). Purification by column chromatography on silica gel (eluent = 10% DCM/petroleum ether), gave the title compound **1c** as a white solid (0.78 g, 2.7 mmol, 53%).

R_f = 0.4 (10% DCM/petroleum ether). ^{1}H NMR (400 MHz, $CDCl_3$) δ_H 7.36-7.28 (4H, m, ArH), 7.23-7.20 (1H, m, ArH), 7.04 (4H, d, *J* = 8.4 Hz, ArH), 6.95 (4H, d, *J* = 8.4 Hz, ArH), 4.95 (2H, s, NCH_2), 2.28 (6H, s, CH_3); ^{13}C{^{1}H} NMR ($CDCl_3$, 101 MHz) δ_C 146.0 (C), 139.5 (C), 130.5 (C), 129.8 (CH), 128.5 (CH), 126.7 (CH), 126.5 (CH), 120.6 (CH), 56.5 (NCH_2), 20.6 (CH_3). This data is in agreement with the literature.[4]

N-(4-Fluorobenzyl)-4-methyl-N-(p-tolyl)aniline, 1d

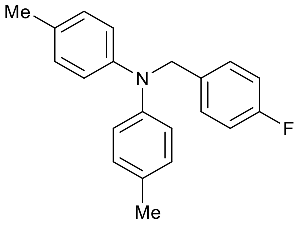

The title compound was prepared according to General Procedure 1, using di-*p*-tolylamine (0.50 g, 2.5 mmol), NaH (0.20 g, 5.1 mmol, 60% in mineral oil), 4-fluorobenzyl bromide (0.38 mL, 3.0 mmol) in DMF (5 mL). Purification by column chromatography on silica gel (eluent = 10% DCM/petroleum ether), gave the title compound **1d** as a clear oil (0.63 g, 2.1 mmol, 81%).

R_f = 0.3 (10% DCM/petroleum ether). ^{1}H NMR (400 MHz, $CDCl_3$) δ_H 7.32-7.29 (2H, m), 7.05 (4H, d, *J* = 8.5 Hz), 6.98 (2H, app t, *J* = 8.6 Hz), 6.93 (4H, d, *J* = 8.5 Hz), 4.90 (2H, s), 2.29 (6H, s); ^{13}C{^{1}H} NMR ($CDCl_3$, 101 MHz) δ_C 161.7 (d, *J* = 244.4 Hz), 145.8, 135.02 (d, *J* = 3.1 Hz), 130.7, 129.8, 128.1 (d, *J* =7.9 Hz), 120.6, 115.3 (d, *J* = 21.2 Hz), 55.8, 20.6. ^{19}F NMR (376 MHz, $CDCl_3$) δ_F -135.1 (CF). HRMS (ESI$^+$) m/z: calcd for $C_{21}H_{21}FN$ [M+H]$^+$ 306.1653; found 306.1659.

N-(4-Chlorobenzyl)-4-methyl-N-(p-tolyl)aniline, 1e

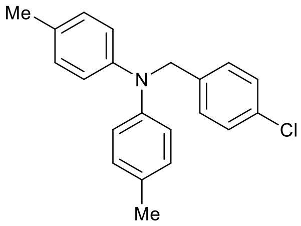

The title compound was prepared according to General Procedure 1, using di-*p*-tolylamine (0.50 g, 2.5 mmol), NaH (0.20 g, 5.1 mmol, 60% in mineral oil), 4-chlorobenzyl chloride (0.49 g, 3.0 mmol) in DMF (5 mL). Purification by column chromatography on silica gel (eluent = 10% DCM/petroleum ether), gave the title compound **1e** as a clear oil (0.63 g, 2.0 mmol, 77%).

R_f = 0.4 (10% DCM/petroleum ether). ^{1}H NMR (400 MHz, $CDCl_3$) δ_H 7.27 (4H, m), 7.05 (4H, d, *J* = 8.50 Hz), 6.92 (4H, d, *J* = 8.50 Hz), 4.90 (2H, s), 2.29 (6H, s); ^{13}C{^{1}H} NMR ($CDCl_3$, 101 MHz) δ_C 145.7, 138.0, 132.3, 130.8, 129.8, 128.6, 138.0, 120.5, 55.9, 20.6. HRMS (ESI$^+$) m/z: calcd for $C_{21}H_{21}{}^{35}ClN$ [M+H]$^+$ 322.1357; found 322.1365; calcd for $C_{21}H_{21}{}^{37}ClN$ [M+H]$^+$ 324.1328; found 324.1342.

N-(4-Bromobenzyl)-4-methyl-N-(p-tolyl)aniline, 1f

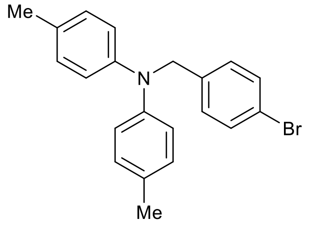

The title compound was prepared according to General Procedure 1, using di-*p*-tolylamine (0.50 g, 2.5 mmol), NaH (0.20 g, 5.1 mmol, 60% in mineral oil), 4-bromobenzyl bromide (0.76 g, 3.0 mmol) in DMF (5 mL). Purification by column chromatography on silica gel (eluent = 10% DCM/petroleum ether), gave the title compound **1f** as a clear oil (0.63 g, 1.7 mmol, 68%).

R_f = 0.4 (10% DCM/petroleum ether). ^{1}H NMR (400 MHz, $CDCl_3$) δ_H 7.42 (2H, d, *J* = 8.4 Hz), 7.22 (2H, d, *J* = 8.4 Hz), 7.05 (4H, d, *J* = 8.5 Hz), 6.92 (4H, d, *J* = 8.5 Hz), 4.88 (2H, s), 2.29 (6H, s); $^{13}C\{^1H\}$ NMR ($CDCl_3$, 101 MHz) δ_C 145.7, 138.6, 131.6, 130.8, 129.8, 128.4, 120.5, 120.4, 55.9, 20.6. HRMS (ESI$^+$) m/z: calcd for $C_{21}H_{21}{}^{79}BrN$ $[M+H]^+$ 366.0852; found 366.0853; calcd for $C_{21}H_{21}{}^{81}BrN$ $[M+H]^+$ 368.0831; found 368.0838.

4-Methyl-N-(4-(methylthio)benzyl)-N-(p-tolyl)aniline, 1g

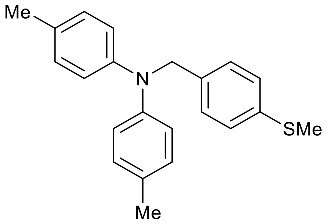

The title compound was prepared according to General Procedure 1, using di-*p*-tolylamine (0.25 g, 1.3 mmol), NaH (0.10 g, 2.5 mmol, 60% in mineral oil), 4-(methylthio)benzyl bromide (0.33 g, 1.5 mmol) in DMF (3 mL). Purification by column chromatography on silica gel (eluent = 3% EtOAc/petroleum ether), gave the title compound **1g** as a yellow oil (0.24 g, 0.72 mmol, 55%).

R_f = 0.5 (3% EtOAc/petroleum ether). ^{1}H NMR (400 MHz, $CDCl_3$) δ_H 7.27 (2H, d, *J* = 8.2 Hz), 7.21 (2H, d, *J* = 8.2 Hz), 7.04 (4H, d, *J* = 8.3 Hz), 6.94 (4H, d, *J* = 8.3 Hz), 4.90 (2H, s), 2.46 (3H, s), 2.29 (6H, s); $^{13}C\{^1H\}$ NMR ($CDCl_3$, 101 MHz) δ_C 145.8, 136.6, 136.4, 130.6, 129.8, 127.1, 127.0, 120.5, 56.0, 20.6, 16.1. HRMS (ESI$^+$) m/z: calcd for $C_{22}H_{23}NS$ $[M]^+$ 333.1546; found 333.1552.

5-Benzyl-10,11-dihydro-5*H*-dibenzo[*b*,*f*]azepine, 1h

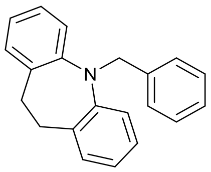

The title compound was prepared according to General Procedure 1, using 10,11-dihydro-5*H*-dibenzo[*b*,*f*]azepine (1.00 g, 5.12 mmol), NaH (0.41 g, 10 mmol, 60% in mineral oil), benzyl bromide (1.20 mL, 20.5 mmol) in THF (10 mL). Purification by column chromatography on silica gel (eluent = 1% EtOAc/petroleum ether), gave the title compound **1h** as a yellow oil (0.82 g, 2.9 mmol, 56%).

R_f = 0.4 (1% EtOAc/petroleum ether). ^{1}H NMR (400 MHz, $CDCl_3$) δ_H 7.41-7.39 (2H, m, ArH), 7.25-7.23 (2H, m, ArH), 7.18-7.05 (7H, m, ArH), 6.91-6.87 (2H, m, ArH), 4.99 (2H, s, NCH_2), 3.26 (4H, s, CH_2); ^{13}C{^{1}H} NMR ($CDCl_3$, 101 MHz) δ_C 148.1 (C), 138.5 (C), 134.0 (C), 129.8 (CH), 128.2 (CH), 128.0 (CH), 126.8 (CH), 126.3 (CH), 122.4 (CH), 120.1 (CH), 55.7 (NCH_2), 32.6 (CH_2). HRMS (ESI$^+$) m/z: calcd for $C_{21}H_{20}N$ $[M+H]^+$ 286.1590; found 286.1592. This data is in agreement with the literature.[5]

5-Methyl-10,11-dihydro-5*H*-dibenzo[*b*,*f*]azepine, 1i

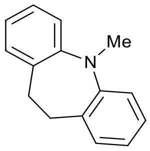

The title compound was prepared according to General Procedure 1, using 10,11-dihydro-5*H*-dibenzo[*b*,*f*]azepine (1.00 g, 5.12 mmol), NaH (0.41 g, 10 mmol, 60% in mineral oil), methyl iodide (1.28 mL, 20.5 mmol) in THF (20 mL). Purification by column chromatography on silica gel (eluent = 3% DCM/hexane), gave the title compound **1i** as a white solid (0.82 g, 3.9 mmol, 77%).

R_f = 0.4 (3% DCM/hexane); ^{1}H NMR (500 MHz, $CDCl_3$) δ_H 7.18-7.14 (2H, m, ArH), 7.11-7.07 (4H, m, ArH), 6.93-6.90 (2H, m, ArH), 3.37 (3H, s, NCH_3), 3.18 (4H, s, CH_2); ^{13}C{^{1}H} NMR ($CDCl_3$, 126 MHz) δ_C 148.7 (C), 133.2 (C), 129.7 (CH), 126.4 (CH), 121.7 (CH), 118.7 (CH), 40.4 (NCH_3), 32.9 (CH_2). This data is in agreement with the literature.[6]

***N*,*N*-Dibenzylaniline, 1n**

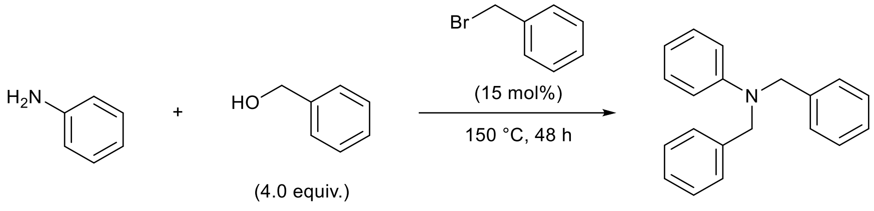

To an oven-dried 3-necked RBF equipped with a magnetic stir bar and charged with N_2 gas, was added benzyl bromide (0.65 mL, 5.5 mmol), aniline (3.30 mL, 36.6 mmol) and benzyl alcohol (15.2 mL, 0.147 mol). This was stirred at 150 °C for 48 h and monitored using TLC. The solvent was removed *in*

vacuo and the crude product was purified via column chromatography on silica gel to obtain the title compound **1n** as an off white solid (1.04 g, 3.80 mmol, 70%).

R_f = 0.2 (15% DCM/petroleum ether). ^{1}H NMR (400 MHz, $CDCl_3$) δ_H 7.37-7.33 (4H, m, ArH), 7.28-7.25 (6H, m, ArH), 7.21-7.17 (2H, m, ArH), 6.78-6.71 (3H, m, ArH), 4.68 (4H, s, NCH_2); $^{13}C\{^1H\}$ NMR ($CDCl_3$, 101 MHz) δ_C 149.2 (C), 138.6 (C), 129.2 (CH), 128.6 (CH), 126.9 (CH), 126.6 (CH), 116.7 (CH), 112.4 (CH), 54.2 (NCH_2). This data is in agreement with the literature.[7]

***N,N*-Dibenzyl-4-methylaniline, 1o**

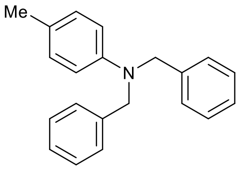

The title compound was prepared according to General Procedure 2, using *p*-toluidine (1.00 g, 9.33 mmol), K_2CO_3 (3.82 g, 28.0 mmol) and benzyl bromide (3.90 mL, 32.7 mmol) in DMF (10 mL). Purification by column chromatography on silica gel (eluent = 10% DCM/petroleum ether), gave the title compound **1o** as a pink solid (1.11 g, 3.86 mmol, 41%).

R_f = 0.3 (10% DCM/petroleum ether). ^{1}H NMR (400 MHz, $CDCl_3$) δ_H 7.35-7.31 (4H, m, ArH), 7.27-7.24 (6H, m, ArH), 7.00 (2H, d, J = 8.7 Hz, ArH), 6.67 (2H, d, J = 8.7 Hz, ArH), 4.64 (4H, s, NCH_2), 2.24 (3H, s, CH_3); $^{13}C\{^1H\}$ NMR ($CDCl_3$, 101 MHz) δ_C 147.0 (C), 138.9 (C), 129.7 (CH), 128.6 (CH), 126.8 (CH), 126.7 (CH), 125.8 (C), 112.6 (CH), 54.2 (NCH_2), 20.2 (CH_3). This data is in agreement with the literature.[8]

***N,N*-Dibenzylnaphthalen-1-amine, 1p**

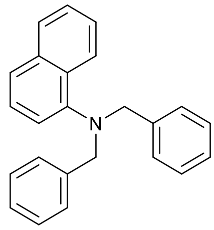

The title compound was prepared according to General Procedure 2, using naphthalen-1-amine (0.50 g, 3.5 mmol), K_2CO_3 (1.45 g, 10.5 mmol) and benzyl bromide (1.04 mL, 8.73 mmol) in MeCN (15 mL). Purification by recrystallisation (hot methanol), gave the title compound **1p** as a pink solid (1.05 g, 3.25 mmol, 93%).

^{1}H NMR (400 MHz, $CDCl_3$) δ_H 8.56 (1H, dd, J = 8.3, 0.7 Hz, ArH), 7.86 (1H, dd, J = 8.3, 1.1 Hz, ArH), 7.58-7.49 (3H, m, ArH), 7.32-7.29 (9H, m, ArH), 7.26-7.21 (2H, m, ArH), 6.95 (1H, dd, J = 7.4, 0.9 Hz, ArH), 4.32 (4H, s, NCH_2); $^{13}C\{^1H\}$ NMR ($CDCl_3$, 101 MHz) δ_C 147.5 (C), 138.2 (C), 134.9 (C), 129.7 (C), 128.5 (CH), 128.4 (CH), 128.2 (CH), 126.9 (CH), 125.7 (CH), 125.5 (CH), 125.4 (CH), 123.7 (CH), 123.5 (CH), 118.4 (CH), 54.1 (NCH_2). This data is in agreement with the literature.[7]

N-Benzyl-N-methylaniline, 1q

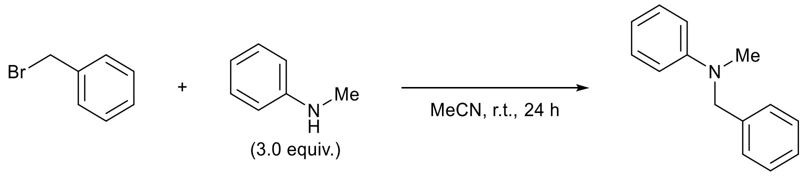

Following a literature procedure,[9] to an oven-dried 3-necked RBF equipped with a magnetic stir bar and charged with N_2 gas, was added benzyl bromide (0.70 mL, 5.9 mmol), *N*-methylaniline (1.90 mL, 17.7 mmol) and MeCN (10 mL). This was stirred at r.t. for 24 h before saturated $NaHCO_3$ (20 mL) and EtOAc (20 mL) were added. The phases were separated, and the aqueous phase was extracted with EtOAc (50 mL × 3). The combined organic phases were washed with water (30 mL), brine (30 mL) and dried over $MgSO_4$. After filtration, the solvent was removed *in vacuo* and the crude product was purified via column chromatography on silica gel to obtain the title compound **1q** as a pale-yellow oil (1.04 g, 5.27 mmol, 90%).

R_f = 0.6 (5% EtOAc/petroleum ether). ^{1}H NMR (400 MHz, $CDCl_3$) δ_H 7.38-7.34 (2H, m, ArH), 7.30-7.25 (5H, m, ArH), 6.81-6.74 (3H, m, ArH), 4.58 (2H, s, NCH_2), 3.06 (3H, s, CH_3); ^{13}C{^{1}H} NMR ($CDCl_3$, 101 MHz) δ_C 149.7 (C), 139.0 (C), 129.2 (CH), 128.5 (CH), 126.8 (CH), 126.7 (CH), 116.5 (CH), 112.3 (CH), 56.6 (NCH_2), 38.5 (CH_3). This data is in agreement with the literature.[10]

N-Benzyl-N,4-dimethylaniline, 1r

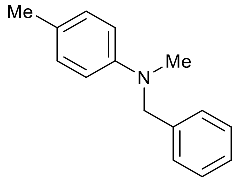

The title compound was prepared according to General Procedure 2, using *N*,4-dimethylaniline (1.00 g, 8.25 mmol), K_2CO_3 (3.42 g, 24.8 mmol) and benzyl bromide (1.20 mL, 9.90 mmol) in EtOH (10 mL). Purification by column chromatography on silica gel (eluent = 10% DCM/petroleum ether), gave the title compound **1r** as a yellow oil (1.29 g, 6.10 mmol, 74%).

R_f = 0.2 (10% DCM /petroleum ether). ^{1}H NMR (400 MHz, $CDCl_3$) δ_H 7.36-7.32 (2H, m, ArH), 7.28-7.24 (3H, m, ArH), 7.06 (2H, d, J = 8.7 Hz, ArH), 6.71 (2H, d, J = 8.7 Hz, ArH), 4.52 (2H, s, NCH_2), 3.00 (3H, s, NCH_3) 2.28 (3H, s, CH_3); ^{13}C{^{1}H} NMR ($CDCl_3$, 101 MHz) δ_C 147.8 (C), 139.2 (C), 129.7 (CH), 128.5 (CH), 126.8 (CH), 126.7 (CH), 125.8 (C), 112.7 (CH), 57.0 (NCH_2), 38.6 (NCH_3), 20.2 (CH_3). This data is in agreement with the literature.[11]

N-Benzhydryl-N-methylaniline, 1s and N,4-dibenzhydryl-N-methylaniline, 1u

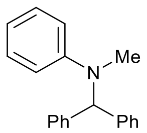

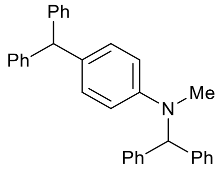

The title compound was prepared according to General Procedure 4, using *N*-methylaniline (1.00 mL, 9.33 mmol), Et_3N (2.60 mL, 18.7 mmol), benzhydryl bromide (2.31 g, 9.33 mmol). Purification by column chromatography on silica gel (eluent = 5% DCM/petroleum ether), *N*-benzhydryl-*N*-methylaniline **1s** as a yellow oil (1.68 g, 6.13 mmol, 66%) and *N*,4-dibenzhydryl-*N*-methylaniline **1u** as a yellow oil (0.39 g, 0.89 mmol, 10%).

N-Benzhydryl-*N*-methylaniline, **1s**: R_f = 0.3 (5% DCM/petroleum ether). ^{1}H NMR (400 MHz, $CDCl_3$) δ_H 7.37-7.21 (12H, m, ArH), 6.82 (2H, d, *J* = 8.1 Hz, ArH), 6.75 (1H, t, *J* = 7.2 Hz, ArH), 6.21 (1H, s, NCH), 2.76 (3H, s, NCH_3); $^{13}C\{^1H\}$ (101 MHz, $CDCl_3$) δ_c 150.1 (C), 140.9 (C), 129.1 (CH), 128.7 (CH), 128.4 (CH), 127.2 (CH), 116.8 (CH), 112.9 (CH), 67.0 (NCH), 34.5 (NCH_3). This data is in agreement with the literature.[12]

N,4-Dibenzhydryl-*N*-methylaniline, **1u**: R_f = 0.2 (5% DCM/petroleum ether). ^{1}H NMR (400 MHz, $CDCl_3$) δ_H 7.40-7.26 (10H, m), 7.21-7.14 (10H, m), 6.95 (2H, d, *J* = 8.6 Hz), 6.71 (2H, d, *J* = 8.6 Hz), 6.15 (1H, s), 5.46 (1H, s), 2.73 (3H, s); $^{13}C\{^1H\}$ (101 MHz, $CDCl_3$) δ_c 148.5, 144.7, 140.8, 132.0, 130.0, 129.4, 128.7, 128.3, 128.2, 127.1, 126.0, 112.6, 67.0, 56.0, 34.5. HRMS (ESI$^+$) m/z: calcd for $C_{33}H_{30}N$ $[M+H]^+$ 440.2373; found 440.2372.

N-Benzhydryl-N,4-dimethylaniline, 1t

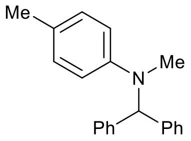

The title compound was prepared according to General Procedure 4, using *N*,4-dimethylaniline (1.18 mL, 9.33 mmol), Et_3N (2.60 mL, 18.7 mmol), benzhydryl bromide (2.29 g, 9.26 mmol). Purification by column chromatography on silica gel (eluent = 5% DCM/petroleum ether), *N*-benzhydryl-*N*,4-dimethylaniline **1t** as a yellow solid (1.72 g, 5.98 mmol, 64%).

R_f = 0.2 (5% DCM/petroleum ether). ^{1}H NMR (400 MHz, $CDCl_3$) δ_H 7.35-7.28 (6H, m, ArH), 7.22-7.20 (4H, m, ArH), 7.04 (2H, d, *J* = 8.4 Hz, ArH), 6.73 (2H, d, *J* = 8.4 Hz, ArH), 6.14 (1H, s, NCH), 2.72 (3H, s, NCH_3), 2.26 (3H, s, CH_3); $^{13}C\{^1H\}$ (101 MHz, $CDCl_3$) δ_c 148.2 (C), 140.8 (C), 129.6 (CH), 128.7 (CH), 128.3 (CH), 127.1 (CH), 126.0 (C), 113.3 (CH), 67.4 (NCH), 34.7 (NCH_3), 20.2 (CH_3). HRMS (ESI$^+$) m/z: calcd for $C_{21}H_{22}N$ $[M+H]^+$ 288.1747; found 288.1756.

_N_-Ethyl-4-methyl-_N_-(_p_-tolyl)aniline, 1v

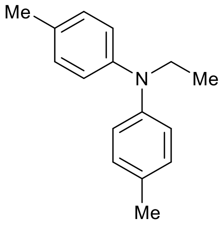

The title compound was prepared according to General Procedure 2, using di-*p*-tolylamine (3.00 g, 15.2 mmol), K_2CO_3 (3.15 g, 23.0 mmol) and iodoethane (1.83 mL, 23.0 mmol) in MeCN (30 mL). Purification by column chromatography on silica gel (eluent = 10% DCM/petroleum ether), gave the title compound **1v** as an orange oil (2.66 g, 11.8 mmol, 78%).

R_f = 0.4 (10% DCM /petroleum ether). ^{1}H NMR (400 MHz, $CDCl_3$) δ_H 7.08 (4H, d, *J* = 8.3 Hz, ArH), 6.89 (4H, d, *J* = 8.3 Hz, ArH), 3.74 (2H, q, *J* = 7.1 Hz, NCH_2), 2.31 (6H, s, CH_3), 1.21 (3H, t, *J* = 7.1 Hz, CH_3); $^{13}C\{^1H\}$ NMR ($CDCl_3$, 101 MHz) δ_C 145.6 (C), 130.2 (C), 129.8 (CH), 120.8 (CH), 46.4 (NCH_2), 20.6 (CH_3), 12.7 (CH_3). HRMS (ESI$^+$) m/z: calcd for $C_{16}H_{20}N$ $[M+H]^+$ 226.1590; found 226.1596.

_N_-Butyl-4-methyl-_N_-(_p_-tolyl)aniline, 1w

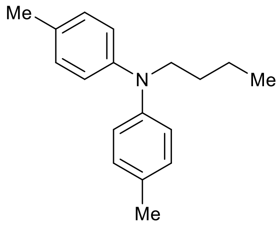

The title compound was prepared according to General Procedure 1, using di-*p*-tolylamine (0.99 g, 5.0 mmol), NaH (0.36 g, 9.0 mmol, 60% in mineral oil), 1-chlorobutane (0.46 mL, 4.5 mmol) in THF (10 mL). Purification by column chromatography on silica gel (eluent = 5% EtOAc/petroleum ether), gave the title compound **1w** as a pale brown oil (0.51 g, 2.0 mmol, 40%).

R_f = 0.9 (5% EtOAc/petroleum ether). ^{1}H NMR (400 MHz, $CDCl_3$) δ_H 7.08 (4H, d, *J* = 8.3 Hz), 6.89 (4H, d, *J* = 8.3 Hz), 3.67-3.63 (2H, m), 2.32 (6H, s), 1.69-1.61 (2H, m), 1.38 (2H, dq, *J* = 15.0, 7.3 Hz), 0.94 (3H, t, *J* = 7.3 Hz); $^{13}C\{^1H\}$ NMR ($CDCl_3$, 101 MHz) δ_C 146.0, 130.2, 129.7, 120.8, 52.2, 29.6, 20.6, 20.3, 13.9. HRMS (ESI$^+$) m/z: calcd for $C_{18}H_{24}N$ $[M+H]^+$ 254.1903; found 254.1900.

N-(Cyclohexylmethyl)-4-methyl-N-(p-tolyl)aniline, 1x

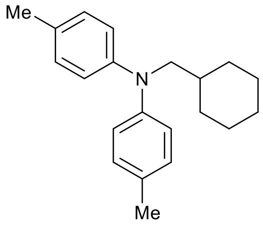

The title compound was prepared according to General Procedure 3, using di-*p*-tolylamine (0.20 g, 1.0 mmol), $BiCl_3$ (0.032 g, 0.10 mmol), cyclohexanecarboxaldehyde (0.11 g, 1.0 mmol) in MeCN (10 mL). Purification by column chromatography on silica gel (eluent = 1.5% EtOAc/petroleum ether), gave the title compound **1x** as a pale-yellow oil (297 mg, 1.00 mmol, quant.).

R_f = 0.7 (10% EtOAc/petroleum ether). ^{1}H NMR (400 MHz, $CDCl_3$) δ_H 7.09-7.06 (4 H, m, ArH), 6.92-6.88 (4 H, m, ArH), 3.49 (2 H, d, *J* = 7.1 Hz, NCH_2), 2.32 (6 H, s, CH_3), 1.90-1.84 (2 H, m, CH_2), 1.80-1.70 (3 H, m, CH, CH_2), 1.70-1.64 (1 H, m, CH_2), 1.27-1.13 (3 H, m, CH_2), 1.03-0.89 (2 H, m, CH_2); ^{13}C{^{1}H} NMR ($CDCl_3$, 101 MHz) δ_C 146.9 (C), 130.1 (C), 129.7 (CH), 121.0 (CH), 59.4 (NCH_2), 36.6 (CH), 31.4 (CH_2), 26.6 (CH_2), 26.0 (CH_2), 20.6 (CH_3). HRMS (ESI$^+$) m/z: calcd for $C_{21}H_{28}N$ $[M+H]^+$ 294.2216; found 294.2215. This data is in agreement with the literature.[13]

1-(p-Tolyl)piperidine, 1y

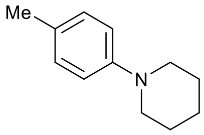

The title compound was prepared according to General Procedure 2, using *p*-toluidine (0.51 mL, 4.7 mmol), K_2CO_3 (1.93 g, 13.9 mmol) and 1,5-dibromobutane (0.95 mL, 7.0 mmol) in MeCN (5 mL). Purification by column chromatography on silica gel (eluent = 5% EtOAc/petroleum ether), gave the title compound **1y** as a yellow oil (0.30 g, 1.7 mmol, 36%).

R_f = 0.6 (5% EtOAc/petroleum ether). ^{1}H NMR (400 MHz, $CDCl_3$) δ_H 7.13-7.06 (2H, m, ArH), 6.93-6.87 (2H, m, ArH), 3.16-3.09 (4H, m, NCH_2), 2.30 (3 H, s, CH_3), 1.78-1.71 (4H, m, CH_2), 1.63-1.56 (2H, m, CH_2); ^{13}C{^{1}H} NMR ($CDCl_3$, 101 MHz) δ_C 150.3 (C), 129.5 (CH), 128.7 (C), 116.9 (CH), 51.3 (NCH_2), 25.9 (CH_2), 24.3 (CH_2), 20.4 (CH_3). This data is in agreement with the literature.[14]

1-(*p*-Tolyl)azepane, 1z

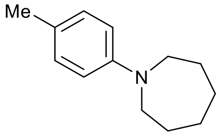

The title compound was prepared according to General Procedure 2, using *p*-toluidine (0.55 mL, 5.0 mmol), K_2CO_3 (2.07 g, 15.0 mmol) and 1,6-dibromohexane (1.15 mL, 7.50 mmol) in MeCN (20 mL). Purification by column chromatography on silica gel (eluent = 5% EtOAc/petroleum ether), gave the title compound **1z** as a yellow oil (0.47 g, 2.5 mmol, 49%).

R_f = 0.6 (5% EtOAc/petroleum ether). ^{1}H NMR (400 MHz, $CDCl_3$) δ_H 7.03 (2H, d, *J* = 8.6 Hz, ArH), 6.62 (2H, d, *J* = 8.6 Hz, ArH), 3.46-3.43 (4H, m, NCH_2), 2.25 (3H, s, CH_3), 1.80-1.78 (4H, m, CH_2), 1.58-1.52 (4H, m, CH_2); $^{13}C\{^1H\}$ NMR ($CDCl_3$, 101 MHz) δ_C 146.8 (C), 129.8 (CH), 124.1 (C), 111.2 (CH), 49.2 (NCH_2), 27.8 (CH_2), 27.2 (CH_2), 20.1 (CH_3). HRMS (ESI^+) m/z: calcd for $C_{13}H_{20}N$ $[M+H]^+$ 190.1590; found 190.1595. This data is in agreement with the literature.[15]

1-(*p*-Tolyl)pyrrolidine, 1aa

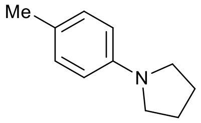

The title compound was prepared according to General Procedure 2, using *p*-toluidine (2.06 mL, 18.7 mmol), K_2CO_3 (7.74 g, 56.0 mmol) and 1,4-dibromobutane (3.34 mL, 28.0 mmol) in MeCN (20 mL). Purification by column chromatography on silica gel (eluent = 5% EtOAc/petroleum ether), gave the title compound **1aa** as a white solid (1.87 g, 11.6 mmol, 62%).

R_f = 0.6 (5% EtOAc/petroleum ether). ^{1}H NMR (400 MHz, $CDCl_3$) δ_H 7.05 (2H, d, *J* = 8.3 Hz, ArH), 6.51 (2H, d, *J* = 8.3 Hz, ArH), 3.24 (4H, m, NCH_2), 2.26 (3H, s, CH_3), 2.00 (4H, m, CH_2); $^{13}C\{^1H\}$ NMR ($CDCl_3$, 101 MHz) δ_C 146.1 (C), 129.6 (CH), 124.4 (C), 111.8 (CH), 47.8 (NCH_2), 25.4 (CH_2), 20.3 (CH_3). This data is in agreement with the literature.[16]

N-Benzhydryl-4-(4-chloro-3,5-dimethylphenoxy)-N-methylaniline, 1ab

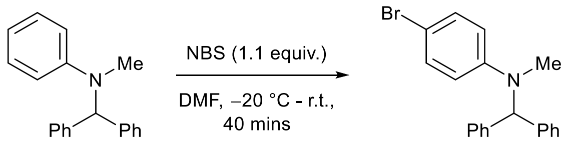

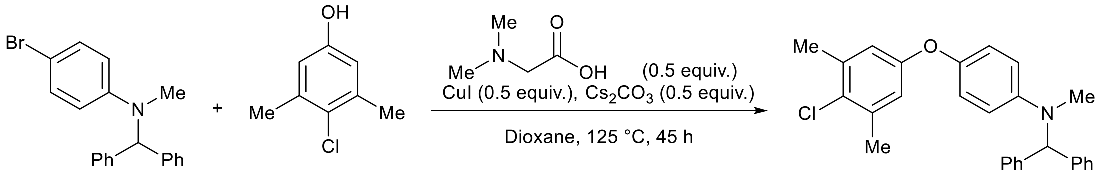

An oven-dried J. Youngs flask equipped with a magnetic stir bar was cooled under vacuum and charged with N_2 gas. Amine **1s** (0.58 g, 2.0 mmol) was added to the J. Youngs flask and the atmosphere cycled three times via vacuum-N_2 backfills. Using standard syringe-septa techniques, DMF (6 mL) was added and the reaction cooled to −20 °C before a solution of NBS (0.37 g, 2.1 mmol) in DMF (2 mL) was slowly added. This was left to stir at −20 °C for 40 mins. After which time, the reaction was allowed to warm to r.t. before it was then diluted with brine (15 mL), extracted with EtOAc (15 mL × 3), and the combined organic layers were dried over Na_2SO_4, and evaporated. A suspension of the residue in Et_2O (15 mL) was filtered, and the filtrate was washed with 3 M NaOH (30 mL), dried over Na_2SO_4, and evaporated to give brominated product without further purification.

An oven-dried RBF equipped with a magnetic stir bar was cooled under vacuum and charged with N_2 gas. Crude brominated amine (0.18 g, 0.500 mmol), CuI (23.8 mg, 0.125 mmol), Cs_2CO_3 (195 mg, 0.600 mmol), chloroxylenol (149 mg, 1.00 mmol) and dimethyl amino acetic acid (11.1 mg, 0.125 mmol) was added to the RBF and the atmosphere cycled three times via vacuum-N_2 backfills. Using standard syringe-septa techniques, 1,4-dioxane (8 mL) was added and the reaction was stirred at 125 °C for 48 h before H_2O (15 mL) and EtOAc (10 mL) were added. The phases were separated, and the aqueous phase was extracted with EtOAc (20 mL × 3). The combined organic phases were washed with water (15 mL), brine (15 mL) and dried over $MgSO_4$. After filtration, the solvent was removed *in vacuo* and the crude product was purified via column chromatography (30% DCM/petroleum ether) on silica gel to obtain the amine **1ab** as a white solid (0.14 g, 0.32 mmol, 16% over two steps).

R_f = 0.4 (30% DCM/petroleum ether). ^{1}H NMR (500 MHz, $CDCl_3$) δ_H 7.37-7.29 (6H, m), 7.23-7.20 (4H, m), 6.92-6.88 (2H, m), 6.79-6.75 (2H, m), 6.68 (2H, s), 6.12 (1H, s), 2.74 (3H, s), 2.32 (6H, s); ^{13}C{^{1}H} NMR ($CDCl_3$, 126 MHz) δ_C 156.6, 147.4, 147.1, 140.7, 137.3, 128.7, 128.4, 127.6, 127.2, 120.6, 117.2, 114.3, 67.8, 35.0, 20.9. HRMS (ASAP$^+$) m/z: calcd for $C_{28}H_{27}{}^{35}ClNO$ $[M+H]^+$ 428.1776; found 428.1780; calcd for $C_{28}H_{27}{}^{37}ClNO$ $[M+H]^+$ 430.1746; found 430.1764.

(1*S*,4*S*)-4-(3,4-Dichlorophenyl)-*N*-methyl-*N*-(*p*-tolyl)-1,2,3,4-tetrahydronaphthalen-1-amine, 1ac

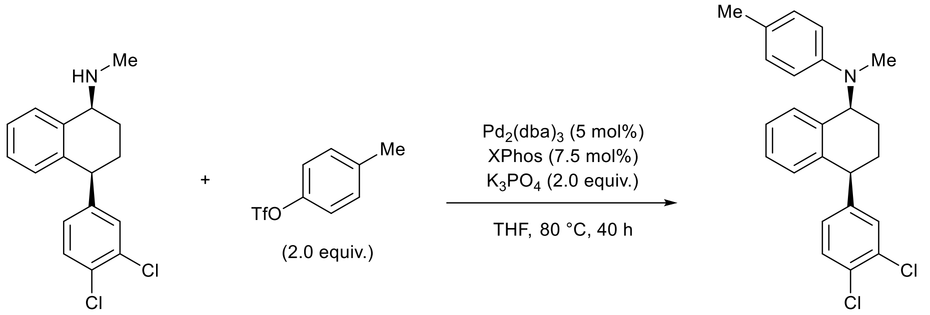

Following an adapted literature procedure,[17] $Pd_2(dba)_3$ (30 mg, 33 μmol), XPhos (23 mg, 48 μmol), K_3PO_4 (276 mg, 1.30 mmol), and sertraline (0.20 g, 0.65 mmol) were added to a J. Youngs flask. The vial was back-filled with N_2 gas three times before THF (1.0 mL) and *p*-tolyl trifluoromethanesulfonate (0.23 mL, 1.3 mmol) were added. The reaction mixture was allowed to stir at 80 °C for 40 h. After completion, the reaction mixture was quenched with water (3.0 mL) and extracted with DCM (3 mL × 3). The organic phase was washed with saturated $NaHCO_3$ and brine, dried with Mg_2SO_4, filtered, and concentrated *in vacuo*. Purification by flash column chromatography on silica gel (eluent = 8% DCM/petroleum ether) gave the title compound **1ac** as a white solid (0.12 g, 0.30 mmol, 47%, 12:1 *dr*).

R_f = 0.4 (15% DCM/petroleum ether). ^{1}H NMR (400 MHz, $CDCl_3$) δ_H 7.44 (1H, d, *J* = 7.6 Hz, ArH) 7.36 (1H, d, *J* = 8.3 Hz, ArH), 7.27-7.17 (2H, m, ArH), 7.16 (1H, d, *J* = 1.9 Hz, ArH), 7.10 (2H, d, *J* = 8.4 Hz, ArH) 6.97 (1H, d, *J* = 7.3 Hz, ArH) 6.88 (1H, dd, *J* = 8.3, 1.9 Hz, ArH) 6.83 (2H, d, *J* = 8.4 Hz, ArH) 5.08 (1H, dd, *J* = 9.9, 6.1 Hz, NCH) 4.21 (1H, dd, *J* = 5.4, 3.5 Hz, CH) 2.70 (3H, s, NCH_3) 2.33-2.25 (1H, m, CH_2), 2.29 (3H, s, CH_3) 2.03 (1H, ddt, *J* = 13.5, 5.4, 3.0 Hz , CH_2) 1.90-1.73 (2H, m, CH_2); $^{13}C\{^1H\}$ NMR ($CDCl_3$, 101 MHz) δ_C 148.3 (C), 147.3 (C), 138.5 (C), 138.2 (C), 132.3 (C), 130.7 (CH), 130.6 (CH), 130.1 (CH), 130.0 (C), 129.8 (CH), 128.1 (CH), 127.9 (CH), 127.3 (CH), 127.2 (CH), 126.0 (C), 113.1 (CH), 58.5 (NCH), 43.4 (CH), 33.1 (NCH_3), 30.5 (CH_2), 20.8 (CH_2), 20.2 (CH_3). HRMS (ESI^+) m/z: calcd for $C_{24}H_{23}{}^{35}Cl_2N$ $[M]^+$ 395.1202; found 395.1206.

2-Chloro-*N*-methylaniline, 1ad and 2-chloro-*N*,*N*-dimethylaniline, 1ae

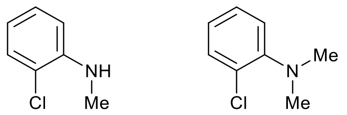

The title compound was prepared according to General Procedure 1, using 2-chloroaniline (3.00 g, 23.5 mmol), NaH (2.82 g, 70.5 mmol, 60% in mineral oil), MeI (5.90 mL, 94.0 mmol). Purification by column chromatography on silica gel (eluent = 1% EtOAc/petroleum ether), 2-chloro-*N*-methylaniline **1ad** as a yellow oil (0.78 g, 5.5 mmol, 23%) and 2-chloro-*N*,*N*-dimethylaniline **1ae** as a yellow oil (0.14 g, 0.90 mmol, 4%).

2-Chloro-*N*-methylaniline, **1ad**: R_f = 0.5 (1% EtOAc/petroleum ether). ^{1}H NMR (400 MHz, $CDCl_3$) δ_H 7.29-7.26 (1H, m, ArH), 7.22-7.18 (1H, m, ArH), 6.68-6.64 (2H, m, ArH), 4.37 (1H, br s, NH), 2.93

(3H, d, J = 5.2 Hz, CH_3); $^{13}C\{^1H\}$ NMR ($CDCl_3$, 101 MHz) δ_C 145.0 (C), 128.9 (CH), 127.8 (CH), 119.0 (C), 117.0 (CH), 110.6 (CH), 30.4 (CH_3). This data is in agreement with the literature.[18]

2-Chloro-*N*,*N*-dimethylaniline, **1ae**: R_f = 0.4 (1% EtOAc/petroleum ether). ^1H NMR (400 MHz, $CDCl_3$) δ_H 7.36 (1H, dd, J = 7.9 and 1.6 Hz, ArH), 7.22 (1H, ddd, J = 8.1, 7.3 and 1.6 Hz, ArH), 7.08 (1H, ddd, J = 8.9, 8.1 and 1.6 Hz, ArH), 6.95 (1H, ddd, J = 8.9, 7.9 and 1.6 Hz, ArH), 2.83 (6H, s, CH_3); $^{13}C\{^1H\}$ NMR ($CDCl_3$, 101 MHz) δ_C 150.4 (C), 130.7 (CH), 128.3 (C), 127.4 (CH), 123.2 (CH), 120.0 (CH), 43.8 (CH_3). This data is in agreement with the literature.[19]

***N*-Methyl-2-phenoxyaniline, 1af and *N*,*N*-dimethyl-2-phenoxyaniline, 1ag**

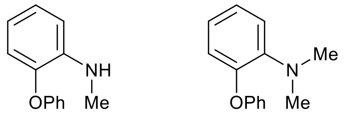

The title compound was prepared according to General Procedure 1, using 2-phenoxyaniline (2.00 g, 10.8 mmol), NaH (1.30 g, 32.4 mmol, 60% in mineral oil), MeI (2.00 mL, 32.4 mmol). Purification by column chromatography on silica gel (eluent = 1% EtOAc/petroleum ether), *N*-methyl-2-phenoxyaniline **1af** as a yellow oil (0.06 g, 0.3 mmol, 3%) and *N*,*N*-dimethyl-2-phenoxyaniline **1ag** as a yellow oil (0.60 g, 2.8 mmol, 26%).

N-Methyl-2-phenoxyaniline, **1af**: R_f = 0.3 (1% EtOAc/petroleum ether). ^1H NMR (400 MHz, $CDCl_3$) δ_H 7.34-7.29 (2H, m, ArH), 7.12-7.04 (2H, m, ArH), 6.99-6.96 (2H, m, ArH), 6.86 (1H, dd, J = 7.9 and 1.5 Hz, ArH), 6.75 (1H, dd, J = 7.9 and 1.5 Hz, ArH), 6.67 (1H, dt, J = 7.9 and 1.5 Hz, ArH), 4.20 (1H, br s, NH), 2.87 (3H, s, CH_3); $^{13}C\{^1H\}$ NMR ($CDCl_3$, 101 MHz) δ_C 157.6 (C), 142.8 (C), 141.6 (C), 129.7 (CH), 125.1 (CH), 122.6 (CH), 119.2 (CH), 117.2 (CH), 116.5 (CH), 110.9 (CH), 30.3 (CH_3). This data is in agreement with the literature.[20]

N,*N*-Dimethyl-2-phenoxyaniline, **1ag**: R_f = 0.2 (1% EtOAc/petroleum ether). ^1H NMR (400 MHz, $CDCl_3$) δ_H 7.35-7.30 (2H, m), 7.13-6.99 (5H, m), 6.92-6.91 (2H, m), 2.85 (6H, s); $^{13}C\{^1H\}$ NMR ($CDCl_3$, 101 MHz) δ_C 157.5, 148.4, 144.9, 129.5, 124.3, 122.5, 121.6, 120.6, 118.5, 117.7, 43.0. HRMS (ESI^+) m/z: calcd for $C_{14}H_{16}NO$ $[M+H]^+$ 214.1226; found 214.1224.

***N*-Ethyl-*N*,4-dimethylaniline, 1ah**

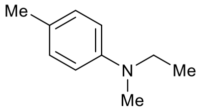

The title compound was prepared according to General Procedure 2, using *N*,4-dimethylaniline (1.8 g, 15 mmol), K_2CO_3 (2.30 g, 16.5 mmol) and bromoethane (2.2 mL, 30 mmol) in DMF (15 mL). Purification by column chromatography on silica gel (eluent = 2% EtOAc/petroleum ether), gave the title compound **1ah** as a yellow oil (0.79 g, 5.3 mmol, 35%).

R_f = 0.5 (5% EtOAc/petroleum ether). ^{1}H NMR (400 MHz, $CDCl_3$) δ_H 7.10-7.04 (2H, m), 6.72-6.66 (2H, m), 3.39 (2H, q, J = 7.1 Hz), 2.90 (3H, s), 2.28 (3H, s), 1.12 (3H, q, J = 7.1 Hz); ^{13}C{1H} NMR ($CDCl_3$, 101 MHz) δ_C 147.2, 129.7, 125.4, 113.0, 47.1, 37.6, 20.2, 11.0. This data is in agreement with the literature.[21]

_N_-Hexyl-_N_-methylaniline, 1ai

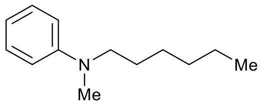

The title compound was prepared according to General Procedure 2, using *N*-methylaniline (1.0 g, 9.3 mmol), K_2CO_3 (4.50 g, 32.6 mmol) and 1-bromohexane (1.60 mL, 11.2 mmol) in MeCN (8 mL). Purification by column chromatography on silica gel (eluent = 2% EtOAc/petroleum ether), gave the title compound **1ai** as a yellow oil (1.56 g, 8.15 mmol, 88%).

R_f = 0.5 (5% EtOAc/petroleum ether). ^{1}H NMR (400 MHz, $CDCl_3$) δ_H 7.27 (2H, t, J = 8.7 Hz), 6.78-6.69 (3H, m), 3.39-3.30 (2H, m), 2.97 (3H, s), 1.68-1.55 (2H, m), 1.42-1.31 (6H, m), 1.00-0.90 (3H, m); ^{13}C{1H} NMR ($CDCl_3$, 101 MHz) δ_C 149.4, 129.1, 115.8, 112.1, 52.8, 38.2, 31.7, 26.9, 26.6, 22.7, 14.0. This data is in agreement with the literature.[22]

3.1.2 Synthesis of Alkenes

General Procedure 5: Wittig reactions of acetophenone derivatives

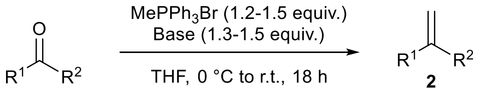

Following an adapted literature procedure,[23] an oven-dried 3-necked RBF equipped with a magnetic stir bar was cooled under vacuum and charged with N_2 gas. $MePPh_3Br$ (1.2-1.5 equiv.) was added to the RBF and the atmosphere cycled three times via vacuum-N_2 backfills. Using standard syringe-septa techniques, THF (15 mL) was added and allowed to stir. This was cooled to 0 °C before the base (1.3-1.6 equiv.) was added slowly. The reaction was stirred at 0 °C for 1 h before ketone (2.5 mmol, 1.0 equiv.) was added. The reaction was stirred until completion, monitored through TLC. H_2O (15 mL) and EtOAc (10 mL) were added, before the phases were separated, and the aqueous phase extracted with EtOAc (25 mL × 3). The combined organic phases were washed with water (15 mL), brine (15 mL) and dried over $MgSO_4$. After filtration, the solvent was removed *in vacuo* and the crude product was purified via column chromatography on silica gel to obtain the pure alkene **2**.

General Procedure 6: Formation of Alkenes from Ketones using MeLi

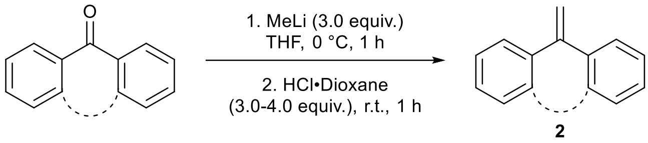

Following an adapted literature procedure,[24] an oven-dried 3-necked RBF equipped with a magnetic stir bar was cooled under vacuum and charged with N_2 gas. Ketone (5.0 mmol, 1.0 equiv.) was added to the RBF and the atmosphere cycled three times via vacuum-N_2 backfills. Using standard syringe-septa techniques, THF (10 mL) was added and allowed to stir. This was cooled to 0 °C before MeLi (3.0 equiv., 1.6 M in Et_2O) was added slowly. The reaction was stirred at 0 °C for 1 h before HCl (3.0 equiv., 4.0 M in Dioxane) was added. The reaction was stirred until completion as determined by TLC. H_2O (30 mL) and EtOAc (20 mL) were added, the phases were separated, and the aqueous phase was extracted with EtOAc (50 mL × 3). The combined organic phases were washed with water (30 mL), brine (30 mL) and dried over $MgSO_4$. After filtration, the solvent was removed *in vacuo* and the crude product was purified via column chromatography on silica gel to obtain the pure alkene **2**.

Phenyl trifluoromethanesulfonate

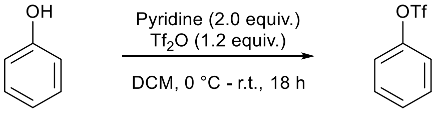

Following an adapted literature procedure,[17] an oven-dried RBF equipped with a magnetic stir bar was cooled under vacuum and charged with N_2 gas. Phenol (1.00 g, 10.6 mmol) was added to the RBF and the atmosphere cycled three times via vacuum-N_2 backfills. Using standard syringe-septa techniques, DCM (10 mL), followed by pyridine (2.50 mL, 21.2 mmol) was added, before being stirred at 0 °C for 10 mins. Triflic anhydride (2.10 mL, 12.7 mmol) was added dropwise to the reaction. The reaction was allowed to warm to r.t. and left to stir for 18 h. Upon completion, the reaction was diluted with Et_2O (40 mL) quenched with HCl (30 mL, 1.0 M). The organic phase was washed with saturated $NaHCO_3$ (30 mL) and brine (30 mL), dried with Na_2SO_4, filtered, and concentrated *in vacuo*. Purification by flash column chromatography on silica gel (eluent = 1% EtOAc/petroleum ether) gave the title compound as a colourless oil (1.68 g, 7.43 mmol, 70%).

R_f = 0.7 (1% EtOAc/petroleum ether). ^{1}H NMR (400 MHz, $CDCl_3$) δ_H 7.50–7.45 (2H, m, ArH), 7.43-7.39 (1H, m, ArH), 7.31-7.28 (2H, m, ArH); ^{13}C{^{1}H} NMR (101 MHz, $CDCl_3$) δ_C = 149.7 (C), 130.3 (CH), 128.4 (CH), 121.3 (CH). ^{19}F NMR (376 MHz, $CDCl_3$) δ_F -72.89 (3F, s). This data is in agreement with the literature.[17]

Trimethyl(2-phenylallyl)silane, 2b

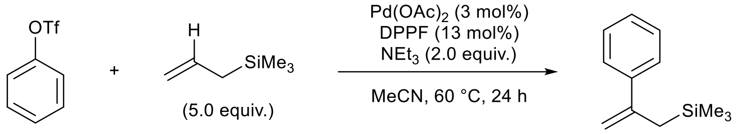

Following an adapted literature procedure,[17] an oven-dried J. Youngs flask equipped with a magnetic stir bar was cooled under vacuum and charged with N_2 gas. $Pd(OAc)_2$ (21 mg, 94 µmol) and 1,1'-bis(diphenylphosphino)ferrocene (0.22 g, 0.40 mmol), was added to the J. Youngs flask and the atmosphere cycled three times via vacuum-N_2 backfills. Using standard syringe-septa techniques, MeCN (12 mL) was added, before being stirred at r.t. for 10 mins. After this time, phenyl triflate (0.49 mL, 3.0 mmol), allyltrimethylsilane (2.40 mL, 15.0 mmol) and Et_3N (0.90 mL, 6.0 mmol) were added, before the flask was sealed and the reaction stirred at 60 °C for 24 h. Upon completion, the reaction was quenched with water (15 mL), and extracted with Et_2O (20 mL × 3). The combined organics were dried with Na_2SO_4, filtered, and concentrated *in vacuo*. Purification by flash column chromatography on silica gel (eluent = petroleum ether) gave the title compound **2b** as a colourless oil (0.29 g, 1.50 mmol, 50%).

R_f = 0.4 (petroleum ether). ^{1}H NMR (500 MHz, $CDCl_3$) δ_H 7.43-7.39 (2H, m, ArH), 7.33-7.28 (2H, m, ArH), 7.27-7.23 (1H, m, ArH), 5.14 (1H, d, J = 1.7 Hz, CH), 4.88 (1H, dt, J = 1.7, 1.0 Hz, CH), 2.04 (2H, d, J = 1.0 Hz, CH_2), -0.08 (9H, s, $SiMe_3$); $^{13}C\{^1H\}$ NMR (101 MHz, $CDCl_3$) δ_C = 146.6 (C), 142.8 (C), 128.1 (CH), 127.2 (CH), 126.3 (CH), 110.0 (CH_2), 26.1 (CH_2), -1.44 (CH_3); ^{29}Si NMR (79 MHz, $CDCl_3$) δ_{Si} = 0.9 (1Si). This data is in agreement with the literature.[25]

Dimethyl(2-methylallyl)(phenyl)silane, 2c

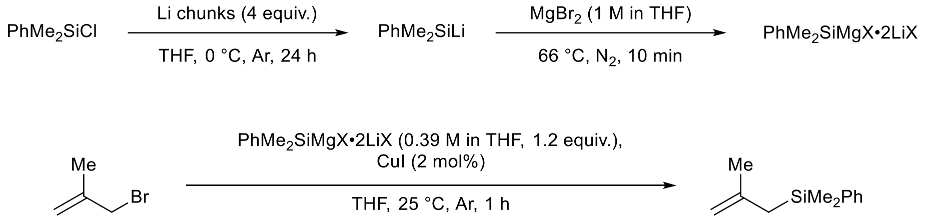

2c

Following a literature procedure,[26] an oven-dried J. Youngs RBF equipped with a magnetic stir bar was cooled under vacuum and charged with Ar gas. The flask was then placed in a glove box and charged with Li chunks (662 mg, 96.0 mmol). After the flask was removed, THF (20 mL) was added, and the suspension cooled to 0 °C, followed by the addition of $PhMe_2SiCl$ (4.0 mL, 24 mmol). The resulting mixture was stirred at this temperature under an Ar atmosphere for 24 h. The concentration of $PhMe_2SiLi$ (0.92 M) was determined by titration against diphenylacetic acid (Kofron's method).[27] An oven-dried two-necked RBF charged with a magnetic stir bar and equipped with a water condenser was connected to a Schlenk line and purged with N_2. The flask was charged with Mg turnings (292 mg, 12.0 mmol) and THF (10 mL) and was then heated to 65 °C followed by the addition of 1,2-

dibromoethane (1.88 g, 10.0 mmol) via syringe-septa techniques. The reaction mixture was refluxed for 3 h to give $MgBr_2$ (1.0 M in THF at 65 °C). The solution of $PhMe_2SiLi$ (10.9 mL, 10.0 mmol) was then added dropwise to the $MgBr_2$ solution over 10 mins at 65 °C. Obtained $PhMe_2SiMgX$•2LiX solution was cooled to r.t. and the concentration of $PhMe_2SiMgX$•2LiX (0.39 M) determined by titration against I_2 (Knochel's method).[28]

An oven-dried J. Youngs RBF equipped with a magnetic stir bar was cooled under vacuum and charged with N_2 gas. CuI (23 mg, 0.12 mmol) was added to the J. Youngs flask, and the atmosphere cycled three times via vacuum-N_2 backfills, followed by the addition of THF (12 mL). After stirring for 10 mins at r.t., 3-bromo-2-methylprop-1-ene (0.61 mL, 6.0 mmol) was added, and the mixture was cooled to 0 °C. The solution of $PhMe_2SiMgX$•2LiX (18.5 mL, 7.2 mmol) was then added over 5 mins. After stirring for 1 h at this temperature, the reaction mixture was quenched with saturated aqueous NH_4Cl (5 mL) and extracted with DCM (10 mL × 3). The organic layers were washed with brine (20 mL), dried with Na_2SO_4, filtered, and concentrated *in vacuo*. Purification by flash column chromatography on silica gel (eluent = hexane) gave the title compound **2c** as a colourless oil (0.76 g, 4.00 mmol, 67%).

R_f = 0.6 (petroleum ether). ^{1}H NMR (400 MHz, $CDCl_3$) δ_H 7.56-7.52 (2H, m, ArH), 7.39-7.34 (3H, m, ArH), 4.63-4.60 (1H, m, CH), 4.51-4.48 (1H, m, CH), 1.79 (2H, d, *J* = 0.92 Hz, CH_2), 1.38 (3H, dd, *J* = 1.4, 0.8 Hz, CH_3), 0.33 (6H, s, $SiMe_2$); $^{13}C\{^1H\}$ NMR (126 MHz, $CDCl_3$) δ_c 143.2 (C), 139.1 (C), 133.6 (CH), 128.9 (CH), 127.7 (CH), 108.8 (CH_2), 27.7 (CH_2), 25.2 (CH_3), -2.9 ($SiCH_3$). This data is in agreement with the literature.[26]

1-Fluoro-4-(1-phenylvinyl)benzene, 2e

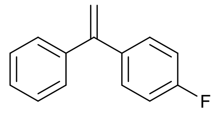

The title compound was prepared according to General Procedure 5, using (4-fluorophenyl)(phenyl)methanone (0.50 g, 2.5 mmol), $MePPh_3Br$ (1.97 g, 5.50 mmol), NaH (1.00 g, 25.0 mmol, 60% in mineral oil) in THF (15 mL). Purification by column chromatography on silica gel (eluent = petroleum ether), gave the title compound **2e** as a colourless oil (0.37 g, 1.9 mmol, 74%).

R_f = 0.3 (petroleum ether). ^{1}H NMR (400 MHz, $CDCl_3$) δ_H 7.38-7.30 (7H, m, ArH), 7.06-7.00 (2H, m, ArH), 5.46 (1H, d, *J* = 1.1 Hz, CH), 5.43 (1H, d, *J* = 1.1 Hz, CH); $^{13}C\{^1H\}$ NMR (126 MHz, $CDCl_3$) δ_c 162.5 (C, d, *J* = 246.6 Hz), 149.1 (C), 141.3 (C), 137.5 (C, d, *J* = 3.2), 129.9 (CH, d, *J* = 8.0 Hz), 128.23 (CH), 128.18 (CH), 127.9 (CH), 115.0 (CH, d, *J* = 21.4 Hz), 114.2 (CH_2); ^{19}F NMR (376 MHz, $CDCl_3$) δ_F -114.76 (1F, app. tt, *J* = 9.1, 4.9 Hz). This data is in agreement with the literature.[29]

1-Bromo-4-(1-phenylvinyl)benzene, 2f

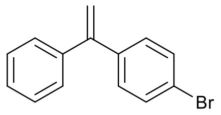

The title compound was prepared according to General Procedure 5, using (4-bromophenyl)(phenyl)methanone (1.30 g, 5.00 mmol), $MePPh_3Br$ (3.03 g, 8.50 mmol), *n*-BuLi (4.7 mL, 7.5 mmol, 1.6 M in Hexane) in THF (20 mL). Purification by column chromatography on silica gel (eluent = 0.6% DCM/petroleum ether), gave the title compound **2f** as a white solid (1.14 g, 4.38 mmol, 88%).

R_f = 0.5 (1% DCM/petroleum ether). ^{1}H NMR (400 MHz, $CDCl_3$) δ_H 7.49-7.46 (2H, m, ArH), 7.38-7.31 (5H, m, ArH), 7.24-7.21 (2H, m ArH), 5.49-5.47 (2H, m, CH_2); ^{13}C{^{1}H} NMR (101 MHz, $CDCl_3$) δ_c 148.9 (C), 140.9 (C), 140.4 (C), 131.3 (CH), 129.9 (CH), 128.3 (CH), 128.2 (CH), 127.9 (CH), 121.7 (CH_2), 114.8 (CH_2). This data is in agreement with the literature.[29]

1-Methoxy-4-(1-phenylvinyl)benzene, 2g

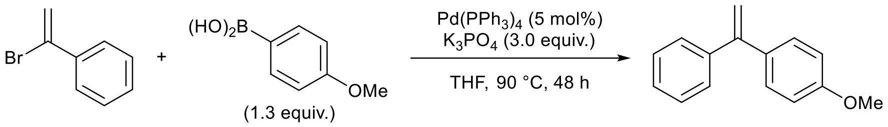

Following an adapted literature procedure,[30] an oven-dried J Young's flask equipped with a magnetic stir bar was cooled under vacuum and charged with N_2 gas. $Pd(PPh_3)_4$ (0.17 g, 0.15 mmol), K_3PO_4 (1.91 g, 9.00 mmol) and 4-methoxyphenylboronic acid (0.59 g, 3.9 mmol) was added to the J. Youngs flask and the atmosphere cycled three times via vacuum-N_2 backfills. Using standard syringe-septa techniques, α-bromostyrene (0.39 mL, 3.0 mmol) and THF (11 mL) were added. The reaction was allowed to stir at 90 °C for 24 h. Water (20 mL) and EtOAc (20 mL) were added. The phases were separated, and the aqueous phase was extracted with EtOAc (20 mL × 3). The combined organic phases were washed with water (15 mL), brine (15 mL) and dried over $MgSO_4$. After filtration, the solvent was removed *in vacuo* and the crude product was purified via column chromatography (eluent = 5% DCM/petroleum ether) on silica gel to give title compound **2g** as a white solid (0.59 g, 2.8 mmol, 94%).

R_f = 0.2 (5% DCM/petroleum ether). ^{1}H NMR (400 MHz, $CDCl_3$) δ_H 7.37-7.32 (5H, m, ArH), 7.29 (2H, m, ArH), 6.88 (2H, m, ArH), 5.41 (1H, d, J = 1.3 Hz, CH_2), 5.37 (1H, d, J = 1.3 Hz, CH_2), 3.84 (3H, s, CH_3); ^{13}C{^{1}H} NMR (101 MHz, $CDCl_3$) δ_c 159.3 (C), 149.5 (C), 141.8 (C), 134.0 (C), 129.4 (CH), 128.3 (CH), 128.1 (CH), 127.6 (CH), 113.5 (CH), 112.9 (CH_2), 55.3 (CH_3). This data is in agreement with the literature.[22]

4,4,5,5-Tetramethyl-2-(4-(1-phenylvinyl)phenyl)-1,3,2-dioxaborolane, 2h

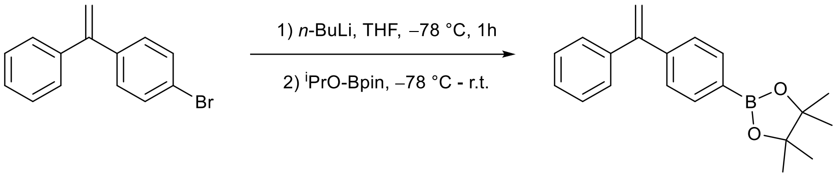

Following an adapted literature procedure,[31] an oven-dried J. Young Flask equipped with a magnetic stir bar was cooled under vacuum and charged with N_2 gas. 1-Bromo-4-(1-phenylvinyl)benzene **2f** (538 mg, 3.00 mmol) was added and the atmosphere cycled three times via vacuum-N_2 backfills. Using standard syringe-septa techniques, THF (6 mL) was added and the reaction allowed to stir. This was cooled to –78 °C before *n*-BuLi (2.3 mL, 3.6 mmol, 1.6 M in Hexane) was added dropwise. The reaction was stirred at –78 °C for 1 h before isopropyl pinacol borate (1.65 mL, 7.50 mmol) was added. The cooling bath was removed, and the reaction was stirred at room temperature. After completion showed by TLC, H_2O (30 mL) and EtOAc (20 mL) were added. The phases were separated, and the aqueous phase was extracted with EtOAc (50 mL × 3). The combined organic phases were washed with water (30 mL), brine (30 mL) and dried over $MgSO_4$. After filtration, the solvent was removed *in vacuo* and the crude product was purified via column chromatography on silica gel (eluent = 2% EtOAc/petroleum ether) to obtain the pure alkene **2h** as a white solid (500 mg, 1.63 mmol, 54%).

R_f = 0.3 (2% EtOAc/petroleum ether). ^{1}H NMR (400 MHz, $CDCl_3$) δ_H 7.81-7.78 (2 H, m, ArH), 7.38-7.32 (7 H, m, ArH), 5.50 (2 H, q, J = 1.3 Hz, CH2), 1.37 (12 H, s, CH3); $^{13}C\{^1H\}$ NMR (101 MHz, $CDCl_3$) δ_c 150.0 (C), 144.3 (C), 141.3 (C), 134.6 (CH), 128.3 (CH), 128.2 (CH), 127.7 (C), 127.6 (CH), 114.8 (CH_2), 83.8 (C), 24.9 (CH_3); ^{11}B NMR (376 MHz, $CDCl_3$) δ_B 30.7. This data is in agreement with the literature.[32]

1-Bromo-3-(1-phenylvinyl)benzene, 2i

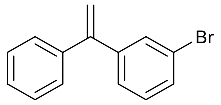

The title compound was prepared according to General Procedure 5, using (3-bromophenyl)(phenyl)methanone (0.79 g, 3.0 mmol), $MePPh_3Br$ (1.82 g, 5.10 mmol), *n*-BuLi (2.8 mL, 4.5 mmol, 1.6 M in Hexane) in THF (12 mL). Purification by column chromatography on silica gel (eluent = 1% DCM/petroleum ether), gave the title compound **2i** as a white solid (0.24 g, 0.91 mmol, 30%).

R_f = 0.5 (1% DCM/petroleum ether). ^{1}H NMR (400 MHz, $CDCl_3$) δ_H 7.53-7.52 (H, m, ArH), 7.49-7.46 (1H, m, ArH), 7.40-7.33 (5H, m, ArH), 7.30-7.27 (1H, m, ArH), 7.52-5.48 (2H, m, CH_2); $^{13}C\{^1H\}$ NMR (101 MHz, $CDCl_3$) δ_c 148.8 (C), 143.7 (C), 140.7 (C), 131.2 (CH), 130.7 (CH), 129.7 (CH), 128.3 (CH),

128.1 (CH), 128.0 (CH), 126.9 (CH), 122.4 (C), 115.3 (CH_2). This data is in agreement with the literature.[29]

1-Methoxy-4-(prop-1-en-2-yl)benzene, 2j

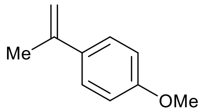

The title compound was prepared according to General Procedure 5, using 1-(4-methoxyphenyl)ethan-1-one (1.00 g, 6.66 mmol), $MePPh_3Br$ (2.85 g, 7.99 mmol), *n*-BuLi (2.8 mL, 4.5 mmol, 1.6 M in Hexane) in THF (20 mL). Purification by column chromatography on silica gel (eluent = 10% DCM/petroleum ether), gave the title compound **2j** as a white solid (0.99 g, 6.6 mmol, quant.).

R_f = 0.2 (10% DCM/petroleum ether). ^{1}H NMR (500 MHz, $CDCl_3$) δ_H 7.45-7.42 (2H, m, ArH), 7.90-7.87 (2H, m, ArH), 5.30 (1H, m CH_2), 5.00 (1H, m, CH_2), 3.83 (1H, s, CH_3), 2.14 (1H, m, CH_3); $^{13}C\{^1H\}$ NMR (126 MHz, $CDCl_3$) δ_c 159.0 (C), 142.5 (C), 133.6 (C), 126.6 (CH), 113.5 (CH), 110.6 (CH_2), 55.3 (CH_3), 21.9 (CH_3). This data is in agreement with the literature.[33]

9-Methylene-9*H*-thioxanthene, 2k

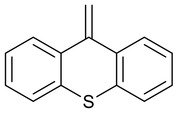

The title compound was prepared according to General Procedure 6, using thioxanthone (1.00 g, 4.71 mmol), MeLi (5.9 mL, 9.4 mmol, 1.6 M in Et_2O), HCl (3.5 mL, 14 mmol, 4.0 M in Dioxane) in THF (10 mL). Purification by column chromatography on silica gel (eluent = 10% DCM/petroleum ether), gave the title compound **2k** as a yellow oil (0.50 g, 2.4 mmol, 50%).

R_f = 0.5 (10% DCM/petroleum ether). ^{1}H NMR (400 MHz, $CDCl_3$) δ_H 7.58-7.54 (2H, m, ArH), 7.31-7.27 (2H, m, ArH), 7.20-7.17 (4H, m ArH), 5.48 (2H, s, CH_2); $^{13}C\{^1H\}$ NMR (101 MHz, $CDCl_3$) δ_c 142.3 (C), 134.5 (C), 131.1 (C), 127.6 (CH), 126.7 (CH), 126.0 (CH), 125.8 (CH), 113.7 (CH_2). This data is in agreement with the literature.[34]

9-Methylene-9*H*-xanthene, 2l

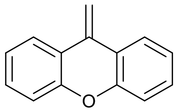

The title compound was prepared according to General Procedure 6, using xanthone (0.98 g, 5.0 mmol), MeLi (3.8 mL, 6.0 mmol, 1.6 M in Et_2O), HCl (2.2 mL, 9.0 mmol, 4.0 M in Dioxane) in THF (10 mL). Purification by flash chromatography on silica gel (eluent = 10% EtOAc/petroleum ether), gave the title compound **2l** as a yellow solid (0.72 g, 3.7 mmol, 74%).

R_f = 0.6 (10% EtOAc/petroleum ether). ^{1}H NMR (400 MHz, $CDCl_3$) δ_H 7.75 (2H, dd, *J* = 8.0, 1.6 Hz, ArH), 7.35-7.30 (2H, m, ArH), 7.15-7.11 (4H, m ArH), 5.53 (2H, s, CH_2); $^{13}C\{^1H\}$ NMR (101 MHz, $CDCl_3$) δ_c 150.6 (C), 132.5 (C), 129.5 (CH), 123.8 (CH), 123.3 (CH), 121.3 (C), 117.3 (CH), 101.1 (CH_2). This data is in agreement with the literature.[35]

1-Methylene-1,2,3,4-tetrahydronaphthalene, 2m

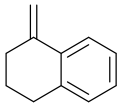

The title compound was prepared according to General Procedure 5, using 3,4-dihydronaphthalen-1(2*H*)-one (1.33 mL, 10.0 mmol), $MePPh_3Br$ (4.28 g, 12.0 mmol), *n*-BuLi (8.0 mL, 13 mmol, 1.6 M) in THF (20 mL). Purification by column chromatography on silica gel (eluent = 6% DCM/petroleum ether), gave the title compound **2m** as a clear oil (1.00 g, 6.93 mmol, 69%).

R_f = 0.7 (10% DCM/petroleum ether). ^{1}H NMR (400 MHz, $CDCl_3$) δ_H 7.71-7.67 (1H, m, ArH), 7.23-7.13 (3H, m, ArH), 5.51 (1H, s, CH_2), 4.99 (1H, s, CH_2), 2.89 (2H, t, J = 6.24, CH_2), 2.59 (2H, m, CH_2), 1.93 (2H, m, CH_2); $^{13}C\{^1H\}$ NMR (101 MHz, $CDCl_3$) δ_c 143.4 (C), 137.3 (C), 134.7 (C), 129.2 (CH), 127.6 (CH), 125.9 (CH), 124.2 (CH), 107.8 (CH_2), 33.2 (CH_2), 30.4 (CH_2), 23.8 (CH_2). This data is in agreement with the literature.[36]

1-Methylene-2,3-dihydro-1*H*-indene, 2n

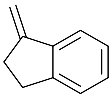

The title compound was prepared according to General Procedure 5, using 2,3-dihydro-1*H*-inden-1-one (1.00 g, 7.56 mmol), $MePPh_3Br$ (3.24 g, 9.10 mmol), ^{t}BuOK (1.02 g, 9.80 mmol) in THF (20 mL). Purification by column chromatography on silica gel (eluent = 8% DCM/petroleum ether), gave the title compound **2n** as a yellow oil (0.52 g, 4.0 mmol, 53%).

R_f = 0.6 (10% DCM/petroleum ether). ^{1}H NMR (500 MHz, $CDCl_3$) δ_H 7.53-7.50 (1H, m, ArH), 7.29-7.20 (3H, m, ArH), 7.54 (1H, t, J=2.52, CH_2), 5.48 (1H, t, J=2.17, CH_2), 3.02-2.99 (2H, m, CH_2), 2.84-2.80 (2H, m, CH_2); $^{13}C\{^1H\}$ NMR (126 MHz, $CDCl_3$) δ_c 150.6 (C), 146.7 (C), 141.1 (C), 128.2 (CH), 126.4 (CH), 125.3 (CH), 120.6 (CH), 102.4 (CH_2), 31.2 (CH_2), 30.1 (CH_2). HRMS (ESI^+) m/z: calcd for $C_{10}H_{11}$ $[M+H]^+$ 131.0855; found 131.0859.

3.2 Optimization Studies

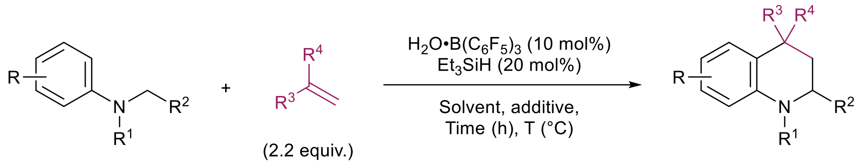

General Procedure 7: Drying $B(C_6F_5)_3$ in situ

An oven-dried J. Youngs flask equipped with a magnetic stir bar was cooled under vacuum and charged with H_2O•$B(C_6F_5)_3$ (n × 10.6 mg, 20.0 µmol) (used as received from the supplier) sealed, and the atmosphere cycled three times via vacuum-N_2 backfills. Using standard syringe-septa techniques, the desired solvent (n × 0.15 mL) was added and allowed to stir. Et_3SiH (n × 0.04 mmol) was added to the solution and allowed to stir for 10 mins, over which time effervescence was observed. (Where n = no. of reactions + 1)

General Procedure 8: $B(C_6F_5)_3$-Catalysed Synthesis of Tetrahydroquinolines from Amines and Alkenes

$B(C_6F_5)_3$ was prepared as in General Procedure 7. To an oven-dried J. Youngs flask (equipped with a magnetic stir bar, cooled under vacuum and charged with N_2, sealed with a septa) was added an aliquot of the $B(C_6F_5)_3$ solution (0.15 mL, 10.6 mg, 0.13 M, 10 mol%). Amine **1** (0.20 mmol) was weighed into a vial and sealed with a septa. The atmosphere in the vial was cycled three times via vacuum-N_2 backfills and the amine was dissolved in the reaction solvent (0.20 mL). The amine solution was transferred to the J. Youngs flask containing $B(C_6F_5)_3$ using standard syringe-septa techniques. The vial was washed with the reaction solvent (0.15 mL) and the washings were transferred to the reaction flask. The alkene **2** (0.44 - 1.00 mmol) was added before the J. Youngs flask was sealed with a Teflon tap, and the mixture was stirred at 85 °C or 120 °C for 22 - 120 h. After cooling to ambient temperature, sat. $NaHCO_3$ (1.50 mL) was added, and the mixture vigorously stirred for 10 minutes. The aqueous phase was separated and extracted with CH_2Cl_2 (3 × 2 mL). The combined organic phases were dried over $MgSO_4$ and the solvent removed *in vacuo*. A spectroscopic yield was obtained using nitromethane as an internal standard and ^{1}H-NMR spectroscopy. The crude material was purified via flash column chromatography on silica gel to obtain the pure tetrahydroquinolines **3**.

General Procedure 9: $B(C_6F_5)_3$-Catalysed Synthesis of Tetrahydroquinolines from Amines and Alkenes with 2,6-Dichloropyridine

As for General Procedure 8, except alkene **2** (0.6 mmol) and 2,6-dichloropyridine (10 mol%) was added to the reaction flask before cycling the flask with N_2 and the addition of the amine solution.

3.2.1 Solvent screen

Table 1. Solvent screen of tetrahydroquinoline synthesis with *N*-methyl-di-*p*-tolylamine 1a and methylallyltrimethylsilane 2a

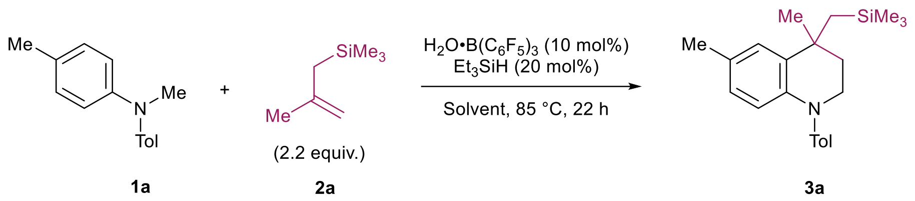

Entry[a]	Solvent	Yield (%)[b]
1	DCE	59
2	*o*-DCB	43
3	PCE	32
4	Me-THF	38
5	*p*-Xylene	14
6	*t*BuOMe	6
7	PhF	4
8	DMSO	0

[a]Following General Procedure 8, reactions were performed using 0.2 mmol of amine **1a** in solvent (0.5 mL) under an N_2 atmosphere. [b]Yields were determined by ^1H NMR analysis of the crude reaction mixture with nitromethane as the internal standard. DCE = 1,2-Dichloroethane; *o*-DCB = 1,2-dichlorobenzene; PCE = perchloroethylene; Me-THF = 2-methyltetrahydrofuran; *t*BuOMe = methyl tert-butyl ether; PhF = fluorobenzene; DMSO = dimethyl sulfoxide.

3.2.2 Optimization of Condition B

Table 2. Screen of basic additives

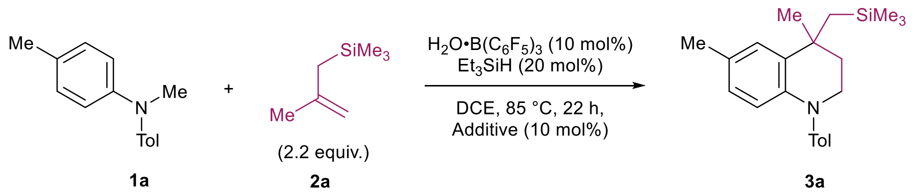

Entry[a]	Additive	$pK_{aH}(H_2O)$[b]	Yield (%)[c]
1	PPh_3		4
2	$PMes_3$		48
3	BINAP		40
4	$P(C_6F_5)_3$		56
5	TMP	11	65
6	DBU	~13	0
7	DABCO	8.8	0

[a]Following General Procedure 8, reactions were performed using 0.2 mmol of amine **1a** in solvent (0.5 mL) under an N_2 atmosphere. [b]from *from http://ibond.nankai.edu.cn/. [c]Yields were determined by ^1H NMR analysis of the crude reaction mixture with nitromethane as the internal standard. $PMes_3$ = Tris(2,4,6-trimethylphenyl)phosphine; BINAP = 2,2′-bis(diphenylphosphino)-1,1′-binaphthyl; TMP = 2,2,6,6-tetramethylpiperidine; DBU = 1,8-diazabicyclo(5.4.0)undec-7-ene; DABCO = 1,4-diazabicyclo[2.2. 2]octane.

Table 3. Screen of 2,6-disubstituted pyridines

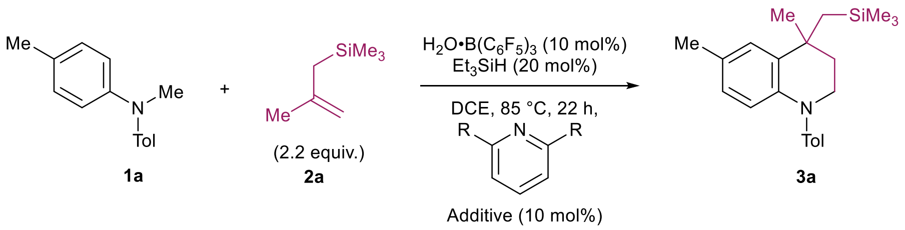

Entry[a]	Additive R group	pK_{aH}(H_2O)[b]	Yield (%)[c]
1	*t*Bu	5	63
2	Me	6.8	59
3	F	−6[d]	64
4	Cl	−3	67
5	NH_2	6	6

[a]Following General Procedure 8, reactions were performed using 0.2 mmol of amine **1a** in solvent (0.5 mL) under an N_2 atmosphere. [b]From *from http://ibond.nankai.edu.cn/. [c]Yields were determined by ^{1}H NMR analysis of the crude reaction mixture with nitromethane as the internal standard. [d]Predicted.

Table 4. Equivalent screen of 2,6-dichloropyridine

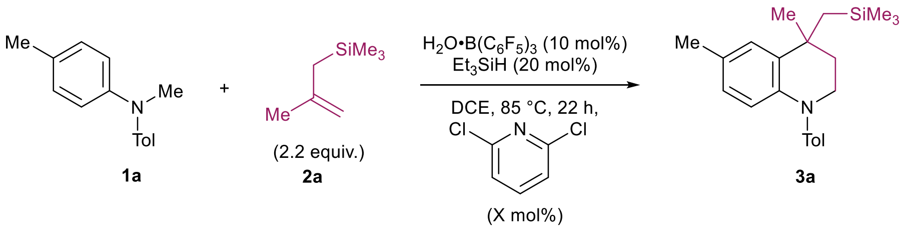

Entry[a]	Additive (mol%)	Yield (%)[b]
1	5	51
2	10	67
3	20	65
4	30	63
5	40	65
6	50	64

[a]Following General Procedure 8, reactions were performed using 0.2 mmol of amine **1a** in solvent (0.5 mL) under an N_2 atmosphere. [b]Yields were determined by ^{1}H NMR analysis of the crude reaction mixture with nitromethane as the internal standard.

Table 5. Equivalence screen of methylallyltrimethylsilane 2a

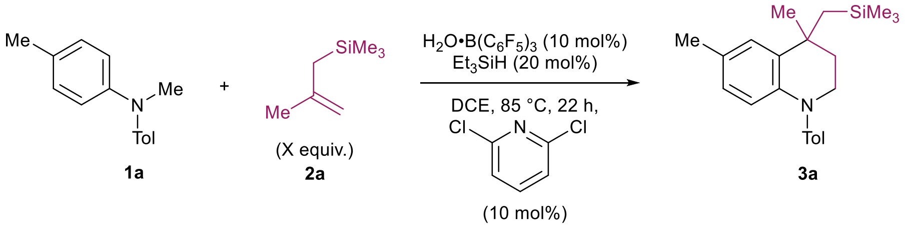

Entry[a]	Alkene (equiv.)	Yield (%)[b]
1	1.1	36
2	2.2	66
3	3.0	76

[a]Following General Procedure 8, reactions were performed using 0.2 mmol of amine **1a** in solvent (0.5 mL) under an N_2 atmosphere. [b]Yields were determined by ^1H NMR analysis of the crude reaction mixture with nitromethane as the internal standard.

Table 6. Optimization of reaction parameters

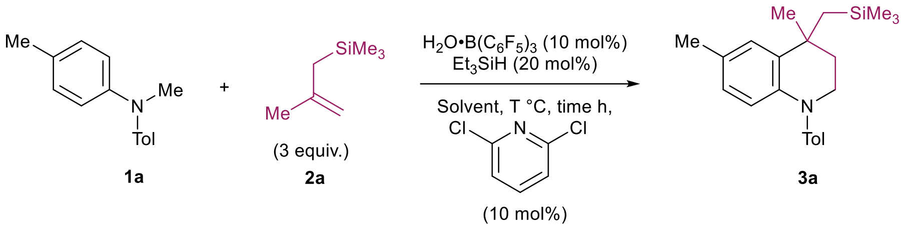

Entry[a]	Solvent	Conc	T (°C)	Time (h)	Yield (%)[b]
1	DCE	0.4[c]	85	22	69
2	DCE	0.4[c]	85	48	74
3	*o*-DCB	0.4[c]	85	22	65
4	*o*-DCB	0.4[c]	120	22	83
5	*o*-DCB	0.8[d]	120	22	60
6	*o*-DCB	2.0[e]	120	22	83

[a]Following General Procedure 8, reactions were performed using 0.2 mmol of amine **1a** in solvent (0.5 mL) under an N_2 atmosphere. [b]Yields were determined by ^1H NMR analysis of the crude reaction mixture with nitromethane as the internal standard. [c]0.5 mL solvent. [d]0.25 mL solvent. [e]0.1 mL solvent. DCE = 1,2-Dichloroethane; *o*-DCB = 1,2-dichlorobenzene.

3.2.3 Optimization of Condition C

Table 7. Equivalent screen of methylallyltrimethylsilane 2a

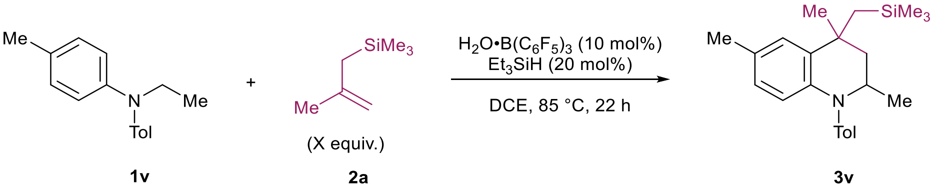

Entry[a]	Alkene (equiv.)	Yield (%)[b]
1	0.5	8
2	0.75	12
3	0.9	20
4	2.2	29
5	3.5	33
6	5.0	37

[a]Following General Procedure 8, reactions were performed using 0.2 mmol of amine **1k** in solvent (0.5 mL) under an N_2 atmosphere. [b]Yields were determined by ^1H NMR analysis of the crude reaction mixture with nitromethane as the internal standard.

Table 8. Optimization of reaction parameters

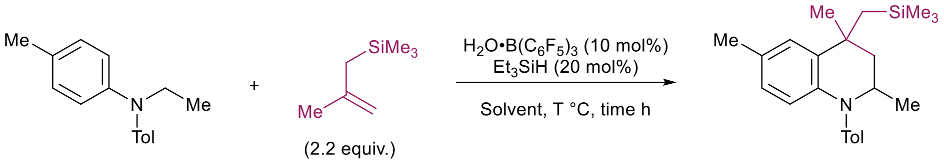

1v **2a** **3v**

Entry[a]	Solvent	T (°C)	Time (h)	Yield (%)[b]
1	*o*-DCB	85	22	30
2	*o*-DCB	110	22	33
3	*o*-DCB	140	22	29
4	DCE	85	22	29
5	DCE	85	48	36
6	DCE	85	72	38
7	DCE	85	96	42
8	DCE	85	120	46
9[c]	DCE	85	22	30
10[d]	DCE	85	120	60

[a]Following General Procedure 8, reactions were performed using 0.2 mmol of amine **1k** in solvent (0.5 mL) under an N_2 atmosphere. [b]Yields were determined by ^1H NMR analysis of the crude reaction mixture with nitromethane as the internal standard. [p]Pure $B(C_6F_5)_3$ used, i.e. the in situ drying procedure using $H_2O.B(C_6F_5)_3$ and Et_3SiH was not used. [d]5.0 equiv. of methylallyltrimethylsilane **2a**. DCE = 1,2-Dichloroethane; *o*-DCB = 1,2-dichlorobenzene.

3.3 Amine Scope of $B(C_6F_5)_3$-Catalysed THQ synthesis

4,6-Dimethyl-1-(*p*-tolyl)-4-((trimethylsilyl)methyl)-1,2,3,4-tetrahydroquinoline, 3a and 4,6-dimethyl-1-(*p*-tolyl)-4-((trimethylsilyl)methyl)-1,4-dihydroquinoline, 3a'

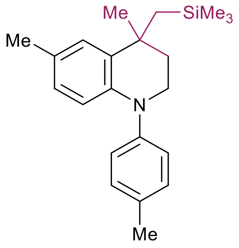

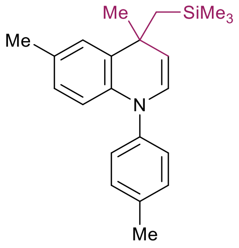

Following General Procedure 8, using *N*-methyl-di-*p*-tolylamine **1a** (42.3 mg, 0.200 mmol), methylallyltrimethylsilane **2a** (80 µL, 0.44 mmol) in DCE (0.50 mL) for 22 h at 85 °C, gave the compound **3a** (63% spectroscopic yield). Purification by column chromatography on silica gel (eluent = 8% DCM/petroleum ether) gave the dihydroquinoline **3a'** as a white solid (36 mg, 0.11 mmol, 54%).

Following General Procedure 8, using *N*-methyl-di-*p*-tolylamine **1a** (84.6 mg, 0.400 mmol), methylallyltrimethylsilane **2a** (0.16 mL, 0.88 mmol) in DCE (1.00 mL) for 22 h at 85 °C, gave the compound **3a** (57% spectroscopic yield). Purification by flash column chromatography on silica gel (through a short silica plug) (eluent = 10% DCM/petroleum ether) gave the compound **3a** as a colourless oil (58 mg, 0.17 mmol, 43%).

Following General Procedure 9, using *N*-methyl-di-*p*-tolylamine **1a** (42.3 mg, 0.200 mmol), methylallyltrimethylsilane **2a** (0.10 mL, 0.60 mmol), 2,6-dichloropyridine (3.00 mg, 20.0 µmol) in *o*-DCB (1.00 mL) for 22 h at 120 °C, gave the compound **3a** (83% spectroscopic yield).

Following General Procedure 8, using *N*-methyl-di-*p*-tolylamine **1a** (42.3 mg, 0.200 mmol), methylallyltrimethylsilane **2a** (0.18 mL, 1.0 mmol) in DCE (0.50 mL) for 120 h at 85 °C, gave the compound **3a** (71% spectroscopic yield).

4,6-Dimethyl-1-(*p*-tolyl)-4-((trimethylsilyl)methyl)-1,2,3,4-tetrahydroquinoline **3a**: R_f = 0.4 (10% DCM/petroleum ether). ^{1}H NMR (500 MHz, $CDCl_3$) δ_H 7.22-7.16 (4H, m, ArH), 7.13 (1H, d, *J* = 2.0 Hz, ArH), 6.79 (1H, ddd, *J* = 8.3, 2.1, 0.6 Hz, ArH), 6.74 (1H, d, *J* = 7.8 Hz, ArH), 3.75-3.67 (1H, m, NCH_2), 3.64-3.57 (1H, m, NCH_2), 2.40 (3H, s, CH_3), 2.32 (3H, s, CH_3), 2.11-2.03 (1H, m, CH_2), 1.89-1.81 (1H, m, CH_2), 1.48 (3H, s, CH_3), 1.25 (2H, s, $SiCH_2$), -0.04 (9H, s, $SiCH_3$); ^{13}C{^{1}H} NMR ($CDCl_3$, 126 MHz) δ_C 146.2 (C), 140.6 (C), 134.7 (C), 132.3 (C), 129.8 (CH), 127.2 (C), 127.0 (CH), 126.6 (CH), 123.6 (CH), 116.4 (CH), 47.1 (NCH_2), 37.9 (CH_2), 35.0 (C), 32.1 (CH_3), 32.0 (CH_2), 20.8 (CH_3), 20.7 (CH_3), 0.8 ($SiCH_3$). HRMS (ESI$^+$) m/z: calcd for $C_{22}H_{31}NSi$ $[M]^+$ 337.2220; found 337.2223.

4,6-Dimethyl-1-(*p*-tolyl)-4-((trimethylsilyl)methyl)-1,4-dihydroquinoline **3a'**: R_f = 0.4 (10% DCM/petroleum ether). ^{1}H NMR (400 MHz, $CDCl_3$) δ_H 7.25 (2H, d, *J* = 8.1 Hz, ArH), 7.18 (2H, d, *J* = 8.1 Hz, ArH), 7.08 (1H, d, *J* = 1.6 Hz, ArH), 6.72 (1H, dd, *J* = 8.3, 1.7 Hz, ArH), 6.39 (1H, d, *J* = 8.3 Hz, ArH), 6.11 (1H, d, *J* = 8.1 Hz, NCH), 4.44 (1H, d, *J* = 8.1 Hz, CH), 2.41 (3H, s, CH_3), 2.27 (3H, s, CH_3), 1.54 (3H, s, CH_3), 1.39 (1H, d, *J* = 14.6 Hz, $SiCH_2$), 1.01 (1H, d, *J* = 14.6 Hz, $SiCH_2$), -0.07 (9H, s, $SiCH_3$); ^{13}C{^{1}H} NMR ($CDCl_3$, 101 MHz) δ_C 142.1 (C), 137.5 (C), 136.0 (C), 130.5 (C), 130.3 (CH), 129.8 (C), 128.5 (CH), 127.9 (CH), 127.4 (CH), 126.6 (CH), 113.2 (CH), 108.3 (CH), 37.8 (CH_3), 37.2 ($SiCH_2$), 35.7 (C), 21.1 (CH_3), 20.7 (CH_3), 0.2 ($SiCH_3$). HRMS (ESI$^+$) m/z: calcd for $C_{22}H_{30}NSi$ $[M + H]^+$ 336.2142; found 336.2136.

1-Mesityl-4-methyl-4-((trimethylsilyl)methyl)-1,2,3,4-tetrahydroquinoline, 3b

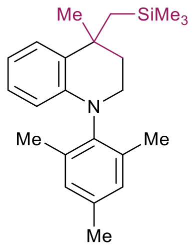

Following General Procedure 8, using *N*,2,4,6-tetramethyl-*N*-phenylaniline **1b** (45.6 mg, 0.200 mmol), methylallyltrimethylsilane **2a** (80 µL, 0.44 mmol) in DCE (0.50 mL) for 22 h at 85 °C, gave the compound **3b** (58% spectroscopic yield). Purification via flash column chromatography on silica gel (eluent = 5% DCM/petroleum ether) gave the title compound **3b** as a clear oil (12.9 mg, 36.7 µmol, 18%).

R_f = 0.4 (5% DCM/petroleum ether). ^{1}H NMR (400 MHz, $CDCl_3$) δ_H 7.25 (1H, d, *J* = 7.1 Hz), 6.99 (2H, s), 6.87-6.79 (1H, m), 6.61-6.53 (1H, m), 5.88 (1H, d, *J* = 8.2 Hz), 3.46 (2H, m), 2.34 (3H, s), 2.13 (3H, s), 2.10 (3H, s), 2.03-1.98 (1H, m), 1.91-1.86 (1H, m), 1.45 (3H, s), 1.26 (1H, d, *J* = 14.8 Hz), 1.20 (1H, d, *J* = 14.8 Hz), 0.01 (9H, s); $^{13}C\{^1H\}$ NMR ($CDCl_3$, 101 MHz) δ_C 142.6, 140.3, 137.5, 137.4, 136.5, 130.1, 129.8, 129.7, 126.7, 125.7, 115.1, 110.7, 44.6, 36.4, 34.8, 32.2, 31.4, 21.0, 17.9, 17.8, 0.83. HRMS (ESI$^+$) m/z: calcd for $C_{23}H_{34}NSi$ $[M+H]^+$ 352.2455; found 352.2443.

4,6-Dimethyl-2-phenyl-1-(*p*-tolyl)-4-((trimethylsilyl)methyl)-1,2,3,4-tetrahydroquinoline, 3c

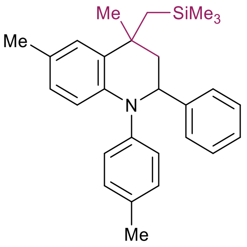

Following General Procedure 8, using *N*-benzyl-4-methyl-*N*-(*p*-tolyl)aniline **1c** (144 mg, 0.500 mmol), methylallyltrimethylsilane **2a** (0.20 mL,1.10 mmol) in DCE (1.00 mL) for 22 h at 85 °C, gave the compound **3c** (42% spectroscopic yield, 1:1 *dr*). Purification by column chromatography on silica gel (eluent = 10% DCM/petroleum ether) gave the title compound **3c** as a brown oil (74 mg, 0.18 mmol, 36%, 1:1.1 *dr*).

Following General Procedure 8, using *N*-benzyl-4-methyl-*N*-(*p*-tolyl)aniline **1c** (144 mg, 0.500 mmol), methylallyltrimethylsilane **2a** (0.35 mL, 2.00 mmol) in DCE (1.00 mL) for 22 h at 85 °C, gave the compound **3c** (63% spectroscopic yield, 1:1 *dr*).

Following General Procedure 9, using *N*-benzyl-4-methyl-*N*-(*p*-tolyl)aniline **1c** (57.4 mg, 0.200 mmol), methylallyltrimethylsilane **2a** (0.10 mL, 0.60 mmol), 2,6-dichloropyridine (3.1 mg, 20.0 µmol) in *o*-DCB (1.00 mL) for 22 h at 120 °C, gave the title compound **3c** (51% spectroscopic yield, 1:1 *dr*).

Mixture of diastereoisomers (1:1.1 dr): R_f = 0.3 (3% DCM/petroleum ether). ^{1}H NMR (400 MHz, $CDCl_3$, asterisk indicate resolved minor diastereoisomer peaks) δ_H 7.40-7.20 (10H, m, ArH, ArH*), 7.15-7.13 (1H, m, ArH*), 7.10-7.07 (1H, m, ArH), 7.00-6.94 (5H, m, ArH), 6.91 (1H, dd, *J* = 8.6, 1.2 Hz, ArH), 6.88-6.83 (4H, m, ArH, ArH*), 6.81 (1H, dd, *J* = 8.6, 1.2 Hz, ArH*), 6.61 (1H, d, *J* = 8.3 Hz, ArH*), 4.71 (1H, dd, *J* = 10.8, 6.4 Hz, NCH), 4.63 (1H, dd, *J* = 10.5, 5.9 Hz, NCH*), 2.33 (3H, s, CH_3), 2.31 (3H, s, CH_3*), 2.30-2.26 (1H, s, CH_2), 2.25 (3H, s, CH_3), 2.24 (3H, s, CH_3*), 2.19-2.10 (2H, m, CH_2*), 1.90 (1H, dd, *J* = 13.3, 10.8 Hz, CH_2), 1.45 (3H, s, CH_3*), 1.39 (3H, s, CH_3), 1.28-1.24 (2H, m, CH_2), 1.23 (1H, d, *J* = 14.2 Hz, $SiCH_2$*), 1.08 (1H, d, *J* = 14.2 Hz, $SiCH_2$*), 0.02 (9H, s, $SiCH_3$*), 0.01 (9H, s, $SiCH_3$); $^{13}C\{^1H\}$ NMR ($CDCl_3$, 101 MHz, asterisk indicate resolved minor diastereoisomer peaks) δ_C 146.7 (C), 146.2 (C*), 146.1 (C), 145.2 (C*), 142.3 (C*), 140.6 (C), 138.9 (C), 136.4 (C*), 132.6 (C*), 130.8 (C), 129.5 (CH*), 129.4 (CH), 129.2 (C), 128.6 (CH), 128.3 (CH*), 128.1 (C*), 127.1 (CH), 127.1 (CH*), 126.9 (CH*), 126.7 (CH*), 126.5 (CH), 126.4 (CH*), 126.3 (CH), 125.4 (CH),

124.7 (CH*), 121.2 (CH), 121.1 (CH), 119.4 (CH*), 61.6 (NCH*), 61.5 (NCH), 51.4 (CH_2), 48.7 (CH_2*), 35.7 (C*), 35.5 (C), 32.0 (CH_3*), 31.5 (CH_2*), 29.5 (CH_2), 28.5 (CH_3), 20.9 (CH_3), 20.8 (CH_3*), 20.8 (CH_3*), 20.6 (CH_3), 1.0 ($SiCH_3$*), 0.8 ($SiCH_3$). HRMS (ESI^+) m/z: calcd for $C_{28}H_{36}NSi$ $[M+H]^+$ 414.2612; found 414.2615.

2-(4-Fluorophenyl)-4,6-dimethyl-1-(*p*-tolyl)-4-((trimethylsilyl)methyl)-1,2,3,4-tetrahydroquinoline, 3d

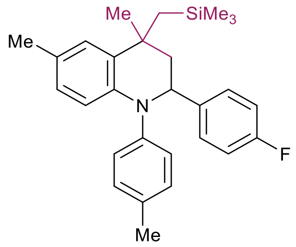

Following General Procedure 8, using *N*-(4-fluorobenzyl)-4-methyl-*N*-(*p*-tolyl)aniline **1d** (61.4 mg, 0.200 mmol), methylallyltrimethylsilane **2a** (80 µL, 0.44 mmol) in DCE (0.50 mL) for 22 h at 85 °C, gave the compound **3d** (42% spectroscopic yield, 1:1 *dr*). Purification via flash column chromatography on silica gel (eluent = 5% DCM/petroleum ether) gave the title compound **3d** as a clear oil (20 mg, 46 µmol, 23%).

Following General Procedure 8, using *N*-(4-fluorobenzyl)-4-methyl-*N*-(*p*-tolyl)aniline **1d** (61.1 mg, 0.200 mmol), methylallyltrimethylsilane **2a** (0.18 mL, 1.0 mmol) in DCE (0.50 mL) for 120 h at 85 °C, gave the title compound **3d** (73% spectroscopic yield, 1:1 *dr*). Purification via flash column chromatography on silica gel (eluent = 5% DCM/petroleum ether) gave the title compound **3d** as a clear oil (42.5 mg, 98.4 µmol, 49%, 1:1.7 *dr*).

Mixture of diastereoisomers (1:1.7 dr): R_f = 0.2 (5% DCM/petroleum ether). ^{1}H NMR (400 MHz, $CDCl_3$, asterisk indicate resolved minor diastereoisomer peaks) δ_H 7.28-7.22 (5H, m, ArH), 7.12 (1H, s, ArH*), 7.07 (1H, s, ArH), 6.99-6.94 (7H, m, ArH), 6.87 (3H, s, ArH), 6.82-6.76 (4H, m, ArH), 6.50 (1H, d, *J* = 8.3 Hz, ArH*), 4.69 (1H, dd, *J* = 10.9, 6.2 Hz, NCH), 4.60 (1H, dd, *J* = 11.3, 4.6 Hz, NCH*), 2.31 (3H, s, CH_3), 2.29 (3H, s, CH_3*), 2.24 (6H, s, CH_3, CH_3*), 2.15 (1H, app t, *J* = 11.3 Hz, CH_2*), 2.05 (1H, dd, *J* = 13.4, 4.6 Hz, CH_2*), 1.88 (1H, app t, *J* = 10.9 Hz, CH_2), 1.44 (3H, s, CH_3*), 1.38 (3H, s, CH_3), 1.31-1.23 (2H, m, CH_2*), 1.20 (1H, d, *J* = 14.8 Hz, $SiCH_2$), 1.07 (1H, d, *J* = 14.8 Hz, $SiCH_2$), 0.00 (9H, s, $SiCH_3$*), -0.02 (9H, s, $SiCH_3$); $^{13}C\{^1H\}$ NMR ($CDCl_3$, 101 MHz, asterisk indicate resolved minor diastereoisomer peaks) δ_C 162.8 (1C, d, *J* = 244.3 Hz, CF), 160.4 (1C, d, *J* = 244.3 Hz, CF), 146.5 (C), 146.0 (C), 142.5 (C), 141.6 (1C, d, *J* = 3.1 Hz, C), 140.7 (1C, d, *J* = 3.1 Hz, C), 140.6 (C), 138.4 (C), 135.7 (C), 133.2 (C), 131.3 (C), 129.6 (CH), 129.5 (CH), 129.2 (CH), 128.7 (1C, d, *J* = 7.8 Hz, CH), 127.9 (1C, d, *J* = 8.0 Hz, CH), 127.1 (CH), 127.0 (CH), 126.5 (CH), 125.5 (CH), 125.4 (CH), 121.7 (CH), 120.9 (CH), 118.9 (CH), 115.5 (CH), 115.3 (CH), 115.0 (CH), 61.0 (NCH*), 60.9 (NCH), 51.1 (CH_2), 48.6 (CH_2*), 35.7 (C*), 35.5 (C), 32.6 (CH_3*), 31.7 (CH_2*), 29.9 (CH_2), 28.6 (CH_3), 20.9

(CH_3), 20.8 (CH_3*), 20.7 (CH_3*), 20.6 (CH_3), 1.0 ($SiCH_3$*), 0.9 ($SiCH_3$). $^{19}F\{^1H\}$ NMR (376 MHz, $CDCl_3$) δ_F –116.5, –116.2 Hz. HRMS (ESI^+) m/z: calcd for $C_{28}H_{35}FNSi$ $[M+H]^+$ 432.2517; found 432.2522.

2-(4-Chlorophenyl)-4,6-dimethyl-1-(*p*-tolyl)-4-((trimethylsilyl)methyl)-1,2,3,4-tetrahydroquinoline, 3e

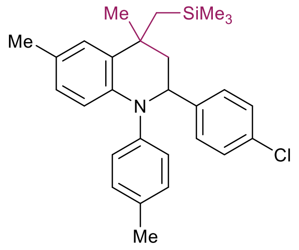

Following General Procedure 8, using *N*-(4-chlorobenzyl)-4-methyl-*N*-(*p*-tolyl)aniline **1e** (64.0 mg, 0.200 mmol), methylallyltrimethylsilane **2a** (80 µL, 0.44 mmol) in DCE (0.50 mL) for 22 h at 85 °C, gave the compound **3e** (42% spectroscopic yield, 1:1.1 *dr*). Purification via flash column chromatography on silica gel (eluent = 5% DCM/petroleum ether) gave the title compound **3e** as a clear oil (25.5 mg, 56.9 µmol, 28%, 1:1.1 *dr*).

Following General Procedure 8, using *N*-(4-chlorobenzyl)-4-methyl-*N*-(*p*-tolyl)aniline **1e** (161 mg, 0.500 mmol), methylallyltrimethylsilane **2a** (0.44 mL, 2.5 mmol) in DCE (1.00 mL) for 120 h at 85 °C, gave the compound **3e** (70% spectroscopic yield, 1:1.1 *dr*). Purification via flash column chromatography on silica gel (eluent = 5% DCM/petroleum ether) gave the title compound **3e** as a white solid (0.11 g, 0.24 mmol, 49%, 1:1.1 *dr*).

The diastereoisomers were isolated separately but were combined to give stronger ^{13}C NMR data.

Major isomer (1:1.1 dr): Rf = 0.2 (5% DCM/petroleum ether). ^1H NMR (400 MHz, $CDCl_3$) δ_H 7.30 (4H, s, ArH), 7.12 (1H, s, ArH), 7.0 (2H, d, *J* = 8.3 Hz, ArH), 6.93 (2H, s, ArH), 6.86 (2H, d, *J* = 8.3 Hz, ArH), 4.73 (1H, dd, *J* = 10.9, 6.3 Hz, NCH), 2.36 (3H, s, CH_3), 2.32-2.27 (1H, m, CH_2), 2.29 (3H, s, CH_3), 1.91 (1H, dd, *J* = 13.4, 11.0 Hz, CH_2), 1.43 (3H, s, CH_3), 1.20 (1H, d, *J* = 14.7 Hz, $SiCH_2$), 1.06 (1H, d, *J* = 14.7 Hz, $SiCH_2$), 0.03 (9H, s, $SiCH_3$); $^{13}C\{^1H\}$ NMR ($CDCl_3$, 101 MHz) δ_C 146.4 (C), 144.6 (C), 140.5 (C), 138.4 (C), 132.1 (C), 131.3 (C), 129.5 (CH), 129.3 (C), 128.7 (CH), 127.8 (CH), 127.1 (CH), 125.5 (CH), 121.5 (CH), 120.9 (CH), 61.0 (NCH), 51.1 (CH_2), 35.5 (CCH_2), 29.8 (CH_3), 28.6 (C), 20.9 (CH_3), 20.6 (CH_3), 0.84 ($SiCH_3$).

Minor isomer (1:1.1 dr): Rf = 0.2 (5% DCM/petroleum ether). ^1H NMR (400 MHz, $CDCl_3$) δ_H 7.30-7.25 (4H, m, ArH), 7.19-7.16 (1H, m, ArH), 7.02 (2H, d, *J* = 8.2 Hz, ArH), 6.87 (2H, d, *J* = 8.2 Hz, ArH), 6.83 (1H, m, ArH), 6.58 (1H, d, *J* = 8.3 Hz, ArH), 4.65 (1H, dd, *J* = 11.2, 5.1 Hz NCH), 2.33 (3H, s, CH_3), 2.28 (3H, s, CH_3), 2.18 (1H, dd, *J* = 13.4, 11.2 Hz, CH_2), 2.11 (1H, dd, *J* = 13.4, 5.05 Hz, CH_2), 1.48 (3H, s, CH_3), 1.26 (1H, d, *J* = 14.6 Hz, $SiCH_2$), 1.23 (1H, d, *J* = 14.6 Hz, $SiCH_2$), 0.05 (9H, s,

$SiCH_3$); $^{13}C\{^1H\}$ NMR ($CDCl_3$, 101 MHz) δ_C 146.0 (C), 143.7 (C), 142.4 (C), 135.8 (C), 133.2 (C), 132.2 (C), 129.7 (CH), 128.5 (CH), 128.5 (CH), 128.1 (C), 127.0 (CH), 126.5 (CH), 125.2 (CH), 119.1 (CH), 61.1 (NCH), 48.6 (CH_2), 35.7 (C), 32.5 (CH_3), 31.6 (CH_2C), 20.8 (CH_3), 20.7 (CH_3), 0.99 ($SiCH_3$).

HRMS (ESI^+) m/z: calcd for $C_{28}H_{35}{}^{35}ClNSi$ $[M+H]^+$ 448.2222; found 448.2212; calcd for $C_{28}H_{35}{}^{37}ClNSi$ $[M+H]^+$ 450.2192; found 450.2210.

2-(4-Bromophenyl)-4,6-dimethyl-1-(*p*-tolyl)-4-((trimethylsilyl)methyl)-1,2,3,4-tetrahydroquinoline, 3f

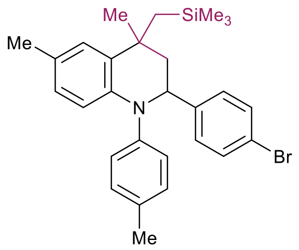

Following General Procedure 8, using *N*-(4-bromobenzyl)-4-methyl-*N*-(*p*-tolyl)aniline **1f** (73.4 mg, 0.200 mmol), methylallyltrimethylsilane **2a** (80 µL, 0.44 mmol) in DCE (0.50 mL) for 22 h at 85 °C, gave the compound **3f** (42% spectroscopic yield, 1:1.1 *dr*). Purification via flash column chromatography on silica gel (eluent = 5% DCM/petroleum ether) gave the title compound **3f** as a clear oil (14.2 mg, 28.8 µmol, 14%, 1:1.1 *dr*).

Following General Procedure 8, using *N*-(4-bromobenzyl)-4-methyl-*N*-(*p*-tolyl)aniline **1f** (183 mg, 0.500 mmol), methylallyltrimethylsilane **2a** (0.44 mL, 2.5 mmol) in DCE (1.00 mL) for 120 h at 85 °C, gave the compound **3f** (56% spectroscopic yield, 1:1.1 *dr*). Purification via flash column chromatography on silica gel (eluent = 5% DCM/petroleum ether) gave the title compound **3f** as a pale-yellow solid (64 mg, 0.13 mmol, 26%, 1:1.1 *dr*).

The diastereoisomers were isolated separately but were combined to give stronger ^{13}C NMR data.

Major isomer (1:1.1 dr): R_f = 0.3 (5% DCM/petroleum ether). ^1H NMR (400 MHz, $CDCl_3$) δ_H 7.44 (2H, d, *J* = 8.4 Hz, ArH), 7.24 (2H, d, *J* = 8.4 Hz, ArH), 7.11 (1H, s, ArH), 7.00 (2H, d, *J* = 8.3 Hz, ArH), 6.93 (2H, s, ArH), 6.86 (2H, d, *J* = 8.3 Hz, ArH), 4.71 (1H, dd, *J* = 10.8, 6.2 Hz, NCH), 2.35 (3H, s, CH_3), 2.31-2.26 (1H, m, CH_2), 2.28 (3H, s, CH_3), 1.89 (1H, dd, *J* = 13.3, 10.9 Hz, CH_2), 1.42 (3H, s, CH_3), 1.24 (1H, d, *J* = 14.7 Hz, $SiCH_2$), 1.09 (1H, d, *J* = 14.7 Hz, $SiCH_2$), 0.02 (9H, s, $SiCH_3$); $^{13}C\{^1H\}$ NMR ($CDCl_3$, 101 MHz) δ_C 146.4 (C), 145.1 (C), 140.4 (C), 138.4 (C), 131.7 (CH), 131.3 (C), 129.5 (CH), 129.3 (C), 128.2 (CH), 127.1 (CH), 125.5 (CH), 121.5 (CH), 121.0 (CH), 120.2 (C), 61.1 (NCH), 51.1 (CH_2), 35.5 (C), 29.8 (CH_2), 28.6 (CH_3), 20.9 (CH_3), 20.6 (CH_3), 0.84 ($SiCH_3$).

Minor isomer (1:1.1 dr): R_f = 0.3 (5% DCM/petroleum ether). ^1H NMR (400 MHz, $CDCl_3$) δ_H 7.40 (2H, d, *J* = 8.4 Hz, ArH), 7.21 (2H, d, *J* = 8.4 Hz, ArH), 7.16 (1H, m, ArH), 7.00 (2H, d, *J* = 8.4 Hz, ArH), 6.86 (2H, d, *J* = 8.4 Hz, ArH), 6.82 (1H, m, ArH), 6.57 (1H, d, *J* = 8.2 Hz, ArH), 4.62 (1H, dd, *J* = 11.0,

5.0 Hz, NCH), 2.33 (3H, s, CH_3), 2.28 (3H, s, CH_3), 2.19-2.07 (2H, m, CH_2), 1.47 (3H, s, CH_3), 1.31 (1H, d, J = 14.7 Hz, $SiCH_2$), 1.27 (1H, d, J = 14.7 Hz, $SiCH_2$), 0.04 (9H, s, $SiCH_3$); $^{13}C\{^1H\}$ NMR ($CDCl_3$, 101 MHz) δ_C 146.0 (C), 144.2 (C), 142.3 (C), 135.9 (C), 133.2 (C), 131.5 (CH), 129.7 (CH), 129.3 (CH), 128.9 (CH), 128.2 (C), 127.0 (CH), 126.5 (CH), 125.2 (CH), 120.3 (C), 61.2 (NCH), 48.6 (CH_2), 35.7 (C), 32.4 (CH_3), 31.6 (CH_2), 20.8 (CH_3), 20.7 (CH_3), 0.99 ($SiCH_3$).

HRMS (ESI^+) m/z: calcd for $C_{28}H_{35}{}^{79}BrNSi$ $[M+H]^+$ 492.1717; found 492.1712; calcd for $C_{28}H_{35}{}^{81}BrNSi$ $[M+H]^+$ 494.1696; found 494.1699.

4,6-Dimethyl-2-(4-(methylthio)phenyl)-1-(*p*-tolyl)-4-((trimethylsilyl)methyl)-1,2,3,4-tetrahydroquinoline, 3g

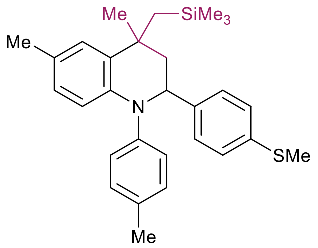

Following General Procedure 8, using 4-methyl-*N*-(4-(methylthio)benzyl)-*N*-(*p*-tolyl)aniline **1g** (66.4 mg, 0.200 mmol), methylallyltrimethylsilane **2a** (80 µL, 0.44 mmol) in DCE (0.50 mL) for 22 h at 85 °C, gave the compound **3g** (8% spectroscopic yield, 1:1.1 *dr*).

Following General Procedure 8, using 4-methyl-*N*-(4-(methylthio)benzyl)-*N*-(*p*-tolyl)aniline **1g** (66.7 mg, 0.200 mmol), methylallyltrimethylsilane **2a** (0.18 mL, 1.0 mmol) in DCE (0.50 mL) for 120 h at 85 °C, gave the compound **3g** (35% spectroscopic yield, 1:1.1 *dr*). Purification via flash column chromatography on silica gel (eluent = 10% DCM/petroleum ether) gave the title compound **3g** as a clear oil (31.5 mg, 68.5 µmol, 34%, 1:1.1 *dr*).

Mixture of diastereoisomers (1:1.1 dr): R_f = 0.1 (10% DCM/petroleum ether). ^1H NMR (500 MHz, $CDCl_3$, asterisk indicate resolved minor diastereoisomer peaks) δ_H 7.26 (2H, d, J = 8.4 Hz, ArH), 7.23 (2H, d, J = 8.4 Hz, ArH), 7.20 (2H, d, J = 8.4 Hz, ArH), 7.16 (2H, d, J = 8.4 Hz, ArH), 7.13 (1H, d, J = 1.6 Hz, ArH), 7.08 (1H, d, J = 1.1 Hz, ArH), 6.97-6.95 (4H, m, ArH), 6.92 (1H, d, J = 8.1 Hz, ArH), 6.89 (1H, dd, J = 8.2, 1.6 Hz, ArH), 6.86-6.83 (4H, m, ArH), 6.80 (1H, dd, J = 8.4, 1.6 Hz, ArH), 6.57 (1H, d, J = 8.2 Hz, ArH), 4.68 (1H, dd, J = 10.9, 6.4 Hz, NCH), 4.59 (1H, dd, J = 11.0, 5.0 Hz, NCH*), 2.48 (3H, s, SCH_3), 2.47 (3H, s, SCH_3*), 2.32 (3H, s, CH_3), 2.30 (3H, s, CH_3*), 2.29-2.27 (1H, m, CH_2), 2.26 (3H, s, CH_3), 2.25 (3H, s, CH_3*), 2.14 (1H, dd, J = 13.4, 11.0 Hz, CH_2), 2.08 (1H, dd, J = 13.4, 5.2 Hz, CH_2), 1.89 (1H, dd, J = 13.4, 11.0 Hz, CH_2), 1.44 (3H, s, CH_3), 1.39 (3H, s, CH_3*), 1.28 (1H, d, J = 14.8 Hz, $SiCH_2$*), 1.24 (1H, d, J = 14.8 Hz, $SiCH_2$*), 1.22 (1H, d, J = 14.7 Hz, $SiCH_2$), 1.08 (1H, d, J = 14.7 Hz, $SiCH_2$), 0.01 (9H, s, $SiCH_3$*), -0.01 (9H, s, $SiCH_3$); $^{13}C\{^1H\}$ NMR ($CDCl_3$, 101 MHz, asterisk indicate resolved minor diastereoisomer peaks) δ_C 146.6 (C), 146.1 (C), 143.1 (C), 142.4 (C), 142.2 (C), 140.6 (C), 138.6 (C), 136.2 (C), 136.2 (C), 136.1 (C), 132.8 (C), 131.0 (C), 129.6 (CH), 129.4

(CH), 129.1 (C), 128.0 (C), 127.6 (CH), 127.1 (CH), 127.0 (CH), 127.0 (CH), 126.9 (CH), 126.7 (CH), 126.4 (CH), 125.4 (CH), 125.0 (CH), 121.4 (CH), 121.0 (CH), 119.2 (CH), 61.2 (NCH*), 61.1 (NCH), 51.2 (CH_2), 48.6 (CH_2), 35.7 (C), 35.5 (C), 32.2 (CH_3*), 31.6 (CH_2*), 29.7 (CH_2), 28.6 (CH_3), 20.9 (CH_3), 20.8 (CH_3), 20.7 (CH_3), 20.6 (CH_3), 16.0 (SCH_3), 15.9 (SCH_3), 0.99 ($SiCH_3$*), 0.83 ($SiCH_3$). HRMS (ESI^+) m/z: calcd for $C_{29}H_{38}NSSi$ $[M+H]^+$ 460.2489; found 460.2477.

3-Methyl-1-phenyl-3-((trimethylsilyl)methyl)-2,3,7,8-tetrahydro-1*H*-benzo[6,7]azepino[3,2,1-ij]quinoline, 3h

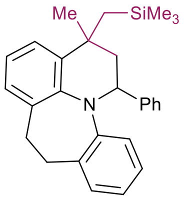

Following General Procedure 8, using 5-benzyl-10,11-dihydro-5*H*-dibenzo[*b*,*f*]azepine **1h** (56.9 mg, 0.200 mmol), methylallyltrimethylsilane **2a** (80 µL, 0.44 mmol) in DCE (0.50 mL) for 22 h at 85 °C, gave the compound **3h** (39% spectroscopic yield, 1:1.2 *dr*). Purification via flash column chromatography on silica gel (eluent = 10% DCM/petroleum ether) gave the title compound **3h** as a clear oil (14.4 mg, 0.03 mmol, 17%, 1:1.2 *dr*).

Following General Procedure 9, using 5-benzyl-10,11-dihydro-5*H*-dibenzo[*b*,*f*]azepine **1h** (57.1 mg, 0.200 mmol), methylallyltrimethylsilane **2a** (0.10 mL, 0.60 mmol), 2,6-dichloropyridine (3.00 mg, 20.0 µmol) in *o*-DCB (1.00 mL) for 22 h at 120 °C, gave the compound **3h** (46% spectroscopic yield, 1:1.2 *dr*).

Following General Procedure 8, using 5-benzyl-10,11-dihydro-5*H*-dibenzo[*b*,*f*]azepine **1h** (57.1 mg, 0.200 mmol), methylallyltrimethylsilane **2a** (0.18 mL, 1.0 mmol) in DCE (0.50 mL) for 120 h at 85 °C, gave the compound **3h** (73% spectroscopic yield). Note, *dr* could not be accurately determined from this reaction mixture due to unresolved signals.

One diastereoisomer isolated: R_f = 0.3 (10% DCM/petroleum ether). ^{1}H NMR (400 MHz, $CDCl_3$) δ_H 7.48 (1H, d, J = 8.0 Hz, ArH), 7.29-7.26 (2H, m, ArH), 7.20-7.09 (5H, m, ArH), 6.99-6.89 (3H, m, ArH), 6.75 (1H, app t, J = 7.5 Hz, ArH), 5.33-5.28 (1H, m, NCH), 3.31-3.22 (2H, m, CH_2), 3.00-2.96 (1H, m, CH_2), 2.48-2.42 (1H, m, CH_2), 2.16 (1H, dd, J = 13.5, 6.4 Hz, CH_2), 2.03 (1H, dd, J = 13.5, 12.3 Hz, CH_2), 1.48 (3H, s, CH_3), 1.39 (2H, s, $SiCH_2$), 0.06 (9H, s, $SiCH_3$); $^{13}C\{^1H\}$ NMR ($CDCl_3$, 101 MHz) δ_C 147.9 (C), 143.9 (C), 141.4 (C), 140.8 (C), 140.0 (C), 130.1 (CH), 128.0 (CH), 127.9 (CH), 127.6 (C), 127.2 (CH), 126.9 (CH), 126.4 (CH), 124.4 (CH), 124.1 (CH), 121.6 (CH), 119.0 (CH), 62.1 (NCH), 48.4 (CH_2), 36.0 (CH_2), 35.4 (CH_2), 31.1 (CH_2), 29.2 (C), 28.9 (CH_3), 0.8 ($SiCH_3$).

HRMS (ESI^+) m/z: calcd for $C_{28}H_{33}NSi$ $[M]^+$ 411.2377; found 411.2382.

3-Methyl-3-((trimethylsilyl)methyl)-2,3,7,8-tetrahydro-1*H*-benzo[6,7]azepino[3,2,1-ij]quinoline, 3i

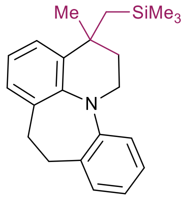

Following General Procedure 8, using 5-methyl-10,11-dihydro-5*H*-dibenzo[*b*,*f*]azepine **1i** (41.3 mg, 0.200 mmol), methylallyltrimethylsilane **2a** (80 µL, 0.44 mmol) in DCE (0.50 mL) for 22 h at 85 °C, gave the compound **3i** (26% spectroscopic yield). Purification via flash column chromatography on silica gel (eluent = 10% DCM/petroleum ether) gave the title compound **3i** as a clear oil (13 mg, 40 µmol, 19%).

Following General Procedure 8, using 5-methyl-10,11-dihydro-5*H*-dibenzo[*b*,*f*]azepine **1i** (41.9 mg, 0.200 mmol), methylallyltrimethylsilane **2a** (0.18 mL, 1.0 mmol) in DCE (0.50 mL) for 120 h at 85 °C, gave the compound **3i** (42% spectroscopic yield). Purification via flash column chromatography on silica gel (eluent = 10% DCM/petroleum ether) gave the title compound **3i** as a clear oil (22 mg, 66 µmol, 33%).

R_f = 0.3 (10% DCM/petroleum ether). ^{1}H NMR (400 MHz, $CDCl_3$) δ_H 7.22-7.15 (3H, m, ArH), 7.10 (1H, d, *J* = 7.2 Hz, ArH), 6.94-6.85 (3H, m, ArH), 3.86 (1H, ddd, *J* = 13.9, 5.6, 3.2 Hz, NCH_2), 3.59 (1H, ddd, *J* = 13.9, 10.4, 2.5 Hz, NCH_2), 3.33-3.24 (2H, m, CH_2), 3.08-2.96 (2H, m, CH_2), 2.28 (1H, ddd, *J* = 13.2, 10.4, 3.2 Hz, CH_2), 1.86 (1H, ddd, *J* = 13.2, 5.7, 2.5 Hz, CH_2), 1.44 (3H, s, CH_3), 1.29 (1H, d, *J* = 14.9 Hz, $SiCH_2$), 1.20 (1H, d, *J* = 14.9 Hz, $SiCH_2$), -0.14 (9H, s, $SiCH_3$); ^{13}C{^{1}H} NMR ($CDCl_3$, 101 MHz) δ_C 147.8 (C), 143.6 (C), 139.0 (C), 133.3 (C), 133.1 (C), 130.0 (CH), 127.1 (CH), 126.5 (CH), 125.6 (CH), 121.3 (CH), 121.2 (CH), 118.7 (CH), 46.9 (NCH_2), 36.8 (CH_2), 36.2 (C), 34.6 (CH_3), 34.3 (CH_2), 33.7 (CH_2), 33.4 (CH_2), 0.55 ($SiCH_3$). HRMS (ASAP$^+$) m/z: calcd for $C_{22}H_{30}NSi$ $[M+H]^+$ 336.2142; found 336.2158.

1,4-Dimethyl-4-((trimethylsilyl)methyl)-1,2,3,4-tetrahydroquinoline, 3j

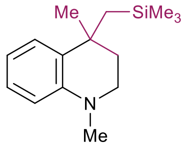

Following General Procedure 8, using *N*,*N*-dimethylaniline **1j** (24.3 mg, 0.200 mmol), methylallyltrimethylsilane **2a** (80 µL, 0.44 mmol) in DCE (0.50 mL) for 22 h at 85 °C, gave the compound **3j** (21% spectroscopic yield).

Following General Procedure 9, using *N*,*N*-dimethylaniline **1j** (24.6 mg, 0.200 mmol), methylallyltrimethylsilane **2a** (0.10 mL, 0.60 mmol), 2,6-dichloropyridine (2.90 mg, 20.0 µmol) in *o*-DCB (0.50 mL) for 22 h at 120 °C, gave the compound **3j** (37% spectroscopic yield).

Following General Procedure 8, using *N*,*N*-dimethylaniline **1j** (23.8 mg, 0.200 mmol), methylallyltrimethylsilane **2a** (0.18 mL, 1.0 mmol) in DCE (0.50 mL) for 120 h at 85 °C, gave the compound **3j** (24% spectroscopic yield). Purification via flash column chromatography on silica gel (eluent = 20% DCM/petroleum ether) gave the title compound **3j** as a colourless oil (8.8 mg, 36 µmol, 18%).

R_f = 0.4 (20% DCM/petroleum ether). ^{1}H NMR (500 MHz, $CDCl_3$) δ_H 7.19 (1H, dd, *J* = 7.7, 1.6 Hz, ArH), 7.09-7.03 (1H, m, ArH), 6.65 (1H, td, *J* = 7.4, 1.4 Hz, ArH), 6.58 (1H, dd, *J* = 8.2, 1.1 Hz, ArH), 3.27-3.18 (2H, m, CH_2), 2.90 (3H, s, CH_3), 1.98-1.93 (1H, m, CH_2), 1.77-1.72 (1H, m, CH_2), 1.35 (3H, s, CH_3), 1.19 (1H, d, *J* = 14.9 Hz, $SiCH_2$), 1.10 (1H, d, *J* = 14.9 Hz, $SiCH_2$), -0.05 (9H, s, $SiCH_3$); ^{13}C{^{1}H} NMR ($CDCl_3$, 101 MHz) δ_C 145.3 (C), 133.1 (C), 126.6 (CH), 126.0 (CH), 116.0 (CH), 110.9 (CH), 47.8 (NCH_2), 39.3 (CH_3), 37.0 (CH_2), 34.9 (C), 32.7 (CH_2), 32.5 (CH_3), 0.7 ($SiCH_3$). HRMS (ESI$^+$) m/z: calcd for $C_{15}H_{26}NSi$ $[M+H]^+$ 248.1829; found 248.1836.

6-Bromo-1,4-dimethyl-4-((trimethylsilyl)methyl)-1,2,3,4-tetrahydroquinoline, 3k

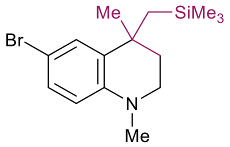

Following General Procedure 8, using 4-bromo-*N*,*N*-dimethylaniline **1k** (40.5 mg, 0.200 mmol), methylallyltrimethylsilane **2a** (80 µL, 0.44 mmol) in DCE (0.50 mL) for 22 h at 85 °C, gave the compound **3k** (26% spectroscopic yield).

Following General Procedure 9, using 4-bromo-*N*,*N*-dimethylaniline **1k** (40.4 mg, 0.200 mmol), methylallyltrimethylsilane **2a** (0.10 mL, 0.60 mmol), 2,6-dichloropyridine (3.40 mg, 20.0 µmol) in *o*-DCB (0.50 mL) for 22 h at 120 °C, gave the compound **3k** (26% spectroscopic yield).

Following General Procedure 8, using 4-bromo-*N*,*N*-dimethylaniline **1k** (40.5 mg, 0.200 mmol), methylallyltrimethylsilane **2a** (0.18 mL, 1.0 mmol) in DCE (0.50 mL) for 120 h at 85 °C, gave the compound **3k** (29% spectroscopic yield). Purification via flash column chromatography on silica gel (eluent = 20% DCM/petroleum ether) gave the title compound **3k** as a colourless oil (18.6 mg, 57.0 µmol, 29%).

R_f = 0.3 (20% DCM/petroleum ether). ^{1}H NMR (400 MHz, $CDCl_3$) δ_H 7.24 (1H, d, *J* = 2.4 Hz, ArH), 7.12 (1H, dd, *J* = 8.8, 2.4 Hz, ArH), 6.42 (1H, d, *J* = 8.8 Hz, ArH), 3.27-3.16 (2H, m, CH_2), 2.87 (3H, s, CH_3), 1.95-1.88 (1H, m, CH_2), 1.74-1.68 (1H, m, CH_2), 1.33 (3H, s, CH_3), 1.14 (1H, d, *J* = 14.9 Hz, $SiCH_2$), 1.07 (1H, d, *J* = 14.9 Hz, $SiCH_2$), -0.03 (9H, s, $SiCH_3$); ^{13}C{^{1}H} NMR ($CDCl_3$, 101 MHz) δ_C

144.1 (C), 135.1 (C), 129.2 (CH), 128.8 (CH), 112.4 (CH), 107.9 (C), 47.6 (NCH_2), 39.2 (CH_3), 36.6 (CH_2), 35.1 (C), 32.5 (CH_2), 32.2 (CH_3), 0.7 ($SiCH_3$). HRMS (ESI^+) m/z: calcd for $C_{15}H_{25}{}^{79}BrNSi$ $[M+H]^+$ 326.0934; found 326.0939; calcd for $C_{15}H_{25}{}^{81}BrNSi$ $[M+H]^+$ 328.0914; found 328.0925.

6-Iodo-1,4-dimethyl-4-((trimethylsilyl)methyl)-1,2,3,4-tetrahydroquinoline, 3l

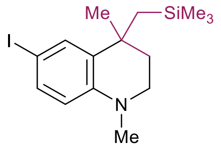

Following General Procedure 8, using 4-iodo-*N*,*N*-dimethylaniline **1l** (98.8 mg, 0.400 mmol), methylallyltrimethylsilane **2a** (0.16 mL, 0.88 mmol) in DCE (0.50 mL) for 22 h at 85 °C, gave the compound **3l** (13% spectroscopic yield). Purification via flash column chromatography on silica gel (eluent = 20% DCM/petroleum ether) gave the title compound **3l** as a colourless oil (18.1 mg, 48.5 µmol, 12%).

Following General Procedure 9, using 4-iodo-*N*,*N*-dimethylaniline **1l** (49.4 mg, 0.200 mmol), methylallyltrimethylsilane **2a** (0.10 mL, 0.60 mmol), 2,6-dichloropyridine (3.40 mg, 20.0 µmol) in *o*-DCB (0.50 mL) for 22 h at 120 °C, gave the compound **3l** (15% spectroscopic yield).

Following General Procedure 8, using 4-iodo-*N*,*N*-dimethylaniline **1l** (49.4 mg, 0.200 mmol), methylallyltrimethylsilane **2a** (0.18 mL, 1.0 mmol) in DCE (0.50 mL) for 120 h at 85 °C, gave the compound **3l** (14% spectroscopic yield).

R_f = 0.3 (20% DCM/petroleum ether). ^1H NMR (400 MHz, $CDCl_3$) δ_H 7.38 (1H, d, J = 2.2 Hz, ArH), 7.29 (1H, dd, J = 8.7, 2.2 Hz, ArH), 6.32 (1H, d, J = 8.7 Hz, ArH), 3.28-3.17 (2H, m, CH_2), 2.87 (3H, s, CH_3), 1.93-1.87 (1H, m, CH_2), 1.73-1.67 (1H, m, CH_2), 1.32 (3H, s, CH_3), 1.13 (1H, d, J = 14.8 Hz, $SiCH_2$), 1.05 (1H, d, J = 14.8 Hz, $SiCH_2$), -0.04 (9H, s, $SiCH_3$); $^{13}C\{^1H\}$ NMR ($CDCl_3$, 101 MHz) δ_C 144.7 (C), 135.7 (C), 135.2 (CH), 134.5 (CH), 113.1 (CH), 77.0 (C), 47.5 (NCH_2), 39.1 (CH_3), 36.5 (CH_2), 35.0 (C), 32.5 (CH_2), 32.2 (CH_3), 0.76 ($SiCH_3$). HRMS (ESI^+) m/z: calcd for $C_{15}H_{25}INSi$ $[M+H]^+$ 374.0795; found 374.0802.

7-Bromo-1,4-dimethyl-4-((trimethylsilyl)methyl)-1,2,3,4-tetrahydroquinoline, 3m

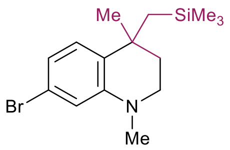

Following General Procedure 8, using 3-bromo-*N*,*N*-dimethylaniline **1m** (40.3 mg, 0.200 mmol), methylallyltrimethylsilane **2a** (80 µL, 0.44 mmol) in DCE (0.50 mL) for 22 h at 85 °C, gave the compound **3m** (21% spectroscopic yield).

Following General Procedure 9, using 3-bromo-*N*,*N*-dimethylaniline **1m** (40.0 mg, 0.200 mmol), methylallyltrimethylsilane **2a** (0.10 mL, 0.60 mmol), 2,6-dichloropyridine (3.40 mg, 20.0 µmol) in *o*-DCB (0.50 mL) for 22 h at 120 °C, gave the compound **3m** (23% spectroscopic yield).

Following General Procedure 8, using 3-bromo-*N*,*N*-dimethylaniline **1m** (39.5 mg, 0.200 mmol), methylallyltrimethylsilane **2a** (0.18 mL, 1.0 mmol) in DCE (0.50 mL) for 120 h at 85 °C, gave the compound **3m** (21% spectroscopic yield). Purification via flash column chromatography on silica gel (eluent = 20% DCM/petroleum ether) gave the title compound **3m** as a colourless oil (11 mg, 34 µmol, 17%).

R_f = 0.4 (20% DCM/petroleum ether). ^{1}H NMR (500 MHz, $CDCl_3$) δ_H 7.01 (1H, d, *J* = 8.1 Hz, ArH), 6.73 (1H, dd, *J* = 8.1, 2.1 Hz, ArH), 6.65 (1H, d, *J* = 2.1 Hz, ArH), 3.29-3.20 (2H, m, CH_2), 2.89 (3H, s, CH_3), 1.93-1.88 (1H, m, CH_2), 1.73-1.68 (1H, m, CH_2), 1.31 (3H, s, CH_3), 1.13 (1H, d, *J* = 14.9 Hz, $SiCH_2$), 1.05 (1H, d, *J* = 14.9 Hz, $SiCH_2$), -0.05 (9H, s, $SiCH_3$); $^{13}C\{^1H\}$ NMR ($CDCl_3$, 101 MHz) δ_C 146.4 (C), 131.7 (C), 127.2 (CH), 120.4 (C), 118.3 (CH), 113.3 (CH), 47.6 (NCH_2), 39.1 (CH_3), 36.6 (CH_2), 34.7 (C), 32.2 (CH_2), 32.1 (CH_3), 0.74 ($SiCH_3$). HRMS (ESI$^+$) m/z: calcd for $C_{15}H_{25}{}^{79}BrNSi$ $[M+H]^+$ 326.0934; found 326.0942; calcd for $C_{15}H_{25}{}^{81}BrNSi$ $[M+H]^+$ 328.0914; found 328.0923.

1-Benzyl-4-methyl-2-phenyl-4-((trimethylsilyl)methyl)-1,2,3,4-tetrahydroquinoline, 3n

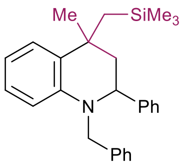

Following General Procedure 8, using *N*,*N*-dibenzylaniline **1n** (54.9 mg, 0.200 mmol), methylallyltrimethylsilane **2a** (80 µL, 0.44 mmol) in DCE (0.50 mL) for 22 h at 85 °C, gave the compound **3n** (30% spectroscopic yield, 1:1.2 *dr*).

Following General Procedure 8, using *N*,*N*-dibenzylaniline **1n** (55.1 mg, 0.200 mmol), methylallyltrimethylsilane **2a** (0.18 mL, 1.0 mmol) in DCE (0.50 mL) for 120 h at 85 °C, gave the compound **3n** (56% spectroscopic yield, 1:1.2 *dr*). Purification via flash column chromatography on silica gel (eluent = 10% DCM/petroleum ether) gave the title compound **3n** as a colourless oil (35 mg, 90 µmol, 44%, 1:1.5 *dr*).

Mixture of diastereoisomers (1:1.5 dr): R_f = 0.2 (10% DCM/petroleum ether). ^{1}H NMR (400 MHz, $CDCl_3$, asterisk indicate resolved minor diastereoisomer peaks) δ_H 7.41-7.19 (17 H, m, ArH), 7.15-7.09 (6H, m, ArH), 7.06-7.02 (1H, m, ArH), 6.87 (1H, d, *J* = 8.2 Hz, ArH), 6.76-6.71 (3H, m, ArH), 4.77 (1H, d, *J* = 15.9 Hz, NCH_2), 4.67 (1H, d, *J* = 17.0 Hz, NCH_2*), 4.52 (1H, dd, *J* = 11.6, 5.3 Hz, NCH), 4.47 (1H, dd, *J* = 11.5, 3.9 Hz, NCH*), 4.05 (1H, d, *J* = 17.0 Hz, NCH_2*), 3.97 (1H, d, *J* = 15.9 Hz, NCH_2), 2.23 (1H, app t, *J* = 13.0, 11.9 Hz, CH_2*), 2.04 (1H, dd, *J* = 13.5, 5.4 Hz, CH_2), 1.95-1.86 (2H, m, CH_2, CH_2*), 1.40 (6H, s, CH_3, CH_3*), 1.36 (1H, d, *J* = 14.8 Hz, $SiCH_2$*), 1.19 (1H, d, *J* = 14.8 Hz,

$SiCH_2$*), 1.12 (1H, d, *J* = 14.7 Hz, $SiCH_2$), 1.02 (1H, d, *J* = 14.7 Hz, $SiCH_2$), -0.03 (18H, s, $SiCH_3$, $SiCH_3$*); $^{13}C\{^1H\}$ NMR ($CDCl_3$, 101 MHz, asterisk indicate resolved minor diastereoisomer peaks) δ_C 145.3 (C), 144.6 (C), 144.3 (C), 144.0 (C), 139.0 (C), 138.4 (C), 134.7 (C), 133.1 (C), 128.5 (CH), 128.2 (CH), 128.1 (CH), 128.0 (CH), 127.4 (CH), 127.2 (CH), 127.1 (CH), 127.0 (CH), 126.9 (CH), 126.8 (CH), 126.7 (CH), 126.4 (CH), 126.1 (CH), 124.5 (CH), 116.7 (CH), 116.5 (CH), 113.6 (CH), 113.2 (CH), 59.9 (NCH*), 59.1 (NCH), 52.9 (NCH_2*), 52.2 (NCH_2), 49.0 (CH_2*), 47.2 (CH_2), 35.4 (C*), 35.2 (C), 33.6 (CH_3), 32.2 (CH_2*), 30.5 (CH_2), 28.7 (CH_3), 0.90 ($SiCH_3$*), 0.67 ($SiCH_3$). HRMS (ESI$^+$) m/z: calcd for $C_{27}H_{34}NSi$ $[M+H]^+$ 400.2455; found 400.2459.

1-Benzyl-4,6-dimethyl-2-phenyl-4-((trimethylsilyl)methyl)-1,2,3,4-tetrahydroquinoline, 3o

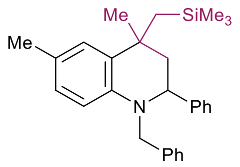

Following General Procedure 8, using *N,N*-dibenzyl-4-methylaniline **1o** (287 mg, 1.00 mmol), methylallyltrimethylsilane **2a** (0.40 mL, 2.2 mmol) in DCE (2.50 mL) for 22 h at 85 °C, gave the compound **3o** (20% spectroscopic yield, 1:1.1 *dr*). Purification via flash column chromatography on silica gel (eluent = 10% DCM/petroleum ether) gave the title compound **3o** as a clear oil (30 mg, 70 µmol, 7%, 1:1.3 *dr*).

Following General Procedure 9, using *N,N*-dibenzyl-4-methylaniline **1o** (57.5 mg, 0.200 mmol), methylallyltrimethylsilane **2a** (0.10 mL, 0.60 mmol), 2,6-dichloropyridine (3.00 mg, 20.0 µmol) in *o*-DCB (1.00 mL) for 22 h at 120 °C, gave the compound **3o** (47% spectroscopic yield, 1:1.1 *dr*).

The diastereoisomers were isolated separately but were combined to give stronger ^{13}C NMR data.

Major isomer (1:1.3 dr): R_f = 0.3 (10% DCM/petroleum ether). ^1H NMR (400 MHz, $CDCl_3$) δ_H 7.34-7.16 (8H, m, ArH), 7.14-7.10 (2H, m, ArH), 7.02 (1H, d, *J* = 2.0 Hz, ArH), 6.90 (1H, dd, *J* = 8.3, 2.0 Hz, ArH), 6.77 (1H, d, *J* = 8.3 Hz, ArH), 4.73 (1H, d, *J* = 15.9 Hz, NCH_2), 4.47 (1H, dd, *J* = 11.5, 5.2 Hz, NCH), 3.94 (1H, d, *J* = 15.9 Hz, NCH_2), 2.29 (3H, s, CH_3), 2.01 (1H, dd, *J* = 13.5, 5.2 Hz, CH_2), 1.86 (1H, dd, *J* = 13.5, 11.5 Hz, CH_2), 1.38 (3H, s, CH_3), 1.06 (1H, d, *J* = 14.8 Hz, $SiCH_2$), 1.01 (1H, d, *J* = 14.8 Hz, $SiCH_2$), -0.04 (9H, s, $SiCH_3$); $^{13}C\{^1H\}$ NMR ($CDCl_3$, 101 MHz) δ_C 144.5 (C), 142.2 (C), 138.6 (C), 134.8 (C), 128.5 (CH), 128.1 (CH), 128.0 (CH), 127.3 (CH), 127.3 (CH), 127.0 (CH), 126.8 (CH), 125.4 (C), 125.4 (CH), 113.3 (CH), 59.1 (NCH), 52.4 (NCH_2), 48.9 (CH_2), 35.2 (C), 30.9 (CH_2), 28.7 (CH_3), 20.6 (CH_3), 0.73 ($SiCH_3$).

Minor isomer (1:1.3 dr): R_f = 0.3 (10% DCM/petroleum ether). ^1H NMR (400 MHz, $CDCl_3$) δ_H 7.34-7.16 (8H, m, ArH), 7.14-7.10 (2H, m, ArH), 7.09 (1H, d, *J* = 1.8 Hz, ArH), 6.83 (1H, dd, *J* = 8.4, 1.8 Hz, ArH), 6.63 (1H, d, *J* = 8.4 Hz, ArH), 4.60 (1H, d, *J* = 16.8 Hz, NCH_2), 4.41 (1H, dd, *J* = 11.6, 3.9 Hz, NCH), 4.00 (1H, d, *J* = 16.8 Hz, NCH_2), 2.26 (3H, s, CH_3), 2.21 (1H, dd, *J* = 13.4, 11.6 Hz, CH_2),

1.86 (1H, dd, J = 13.4, 3.9 Hz, CH_2), 1.38 (3H, s, CH_3), 1.32 (1H, d, J = 14.8 Hz, $SiCH_2$), 1.17 (1H, d, J = 14.8 Hz, $SiCH_2$), -0.05 (9H, s, $SiCH_3$); $^{13}C\{^1H\}$ NMR ($CDCl_3$, 101 MHz) δ_C 144.2 (C), 143.1 (C), 139.3 (C), 133.1 (C), 128.1 (CH), 127.4 (CH), 127.2 (CH), 127.2 (CH), 127.1 (CH), 127.0 (C), 126.5 (CH), 126.3 (CH), 127.0 (CH), 126.4 (CH), 125.4 (CH), 125.6 (CH), 113.7 (CH), 60.0 (NCH), 53.2 (CH_2), 47.5 (CH_2), 35.4 (C), 33.7 (CH_3), 32.3 (CH_2), 20.5 (CH_3), 0.92 ($SiCH_3$).

HRMS (ESI^+) m/z: calcd for $C_{28}H_{36}NSi$ $[M+H]^+$ 414.2612; found 414.2611.

1-Benzyl-4-methyl-2-phenyl-4-((trimethylsilyl)methyl)-1,2,3,4-tetrahydrobenzo[*h*]quinoline, 3p

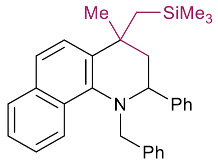

Following General Procedure 8, using *N*,*N*-dibenzylnaphthalen-1-amine **1p** (65.1 mg, 0.200 mmol), methylallyltrimethylsilane **2a** (80 µL, 0.44 mmol) in DCE (0.50 mL) for 22 h at 85 °C, gave the compound **3p** (14% spectroscopic yield, 1:1 *dr*).

Following General Procedure 8, using *N*,*N*-dibenzylnaphthalen-1-amine **1p** (64.3 mg, 0.200 mmol), methylallyltrimethylsilane **2a** (0.18 mL, 1.0 mmol) in DCE (0.50 mL) for 120 h at 85 °C, gave the compound **3p** (43% spectroscopic yield). Purification via flash column chromatography on silica gel (eluent = 10% DCM/petroleum ether) gave the title compound **3p** as a colourless oil (39 mg, 86 µmol, 43%, 1:1.4 *dr*).

Mixture of diastereoisomers (1:1.4 dr): R_f = 0.2 (10% DCM/petroleum ether). ^1H NMR (400 MHz, $CDCl_3$, asterisk indicate resolved minor diastereoisomer peaks) δ_H 8.30-8.26 (2H, m, ArH), 7.85-7.82 (2H, m, ArH), 7.54-7.52 (4H, m, ArH), 7.49-7.35 (12H, m, ArH), 7.32-7.28 (2H, m, ArH), 7.23-7.12 (6H, m, ArH), 6.84-6.82 (2H, m, ArH), 6.72-6.70 (2H, m, ArH), 4.93 (1H, d, J = 14.8 Hz, NCH_2), 4.84 (1H, d, J = 15.0 Hz, NCH_2*), 4.41 (1H, dd, J = 11.0, 4.9 Hz, NCH), 4.34 (1H, dd, J = 10.6, 4.8 Hz, NCH*), 4.19-4.09 (2H, m, NCH_2, NCH_2*), 2.06 (1H, dd, J = 13.5, 4.9 Hz, CH_2), 2.00 (1H, dd, J = 13.4, 10.7 Hz, CH_2*), 1.92 (1H, dd, J = 13.4, 4.9 Hz, CH_2*), 1.84 (1H, dd, J = 13.5, 11.1 Hz, CH_2), 1.18 (3H, s, CH_3), 1.06 (1H, d, J = 15.0 Hz, $SiCH_2$*), 0.98-0.91 (4H, m, CH_3*, $SiCH_2$*), 0.72 (1H, d, J = 15.0 Hz, $SiCH_2$), 0.49 (1H, d, J = 15.0 Hz, $SiCH_2$), -0.03 (9H, s, $SiCH_3$), -0.16 (9H, s, $SiCH_3$*); $^{13}C\{^1H\}$ NMR ($CDCl_3$, 101 MHz, asterisk indicate resolved minor diastereoisomer peaks) δ_C 144.9 (C), 144.7 (C*), 141.5 (C*), 140.8 (C), 137.7 (C*), 137.6 (C), 136.8 (C), 135.3 (C*), 134.0 (C), 133.9 (C*), 129.3 (CH), 129.0 (CH), 128.5 (CH), 128.3 (CH), 128.2 (CH), 128.1 (CH), 127.9 (CH), 127.4 (CH), 127.3 (CH), 127.2 (CH), 127.1 (CH), 127.0 (C), 126.9 (C*) 126.8 (CH), 124.8 (CH), 124.7 (CH), 124.5 (CH), 124.4 (CH), 124.2 (CH), 123.5 (CH), 120.1 (CH), 120.0 (CH), 57.8 (NCH_2), 57.7 (NCH_2*), 57.4 (NCH*), 57.2 (NCH), 46.2 (CH_2), 45.1 (CH_2*), 35.5 (C*), 35.4 (C), 32.1 (CH_2*), 31.8 (CH_2), 31.3 (CH_3*), 30.0

(CH_3), 1.01 ($SiCH_3$), 0.83 ($SiCH_3$*). HRMS (ESI$^+$) m/z: calcd for $C_{31}H_{36}NSi$ [M+H]$^+$ 450.2612; found 450.2617.

1-Benzyl-4-methyl-4-((trimethylsilyl)methyl)-1,2,3,4-tetrahydroquinoline, 3q

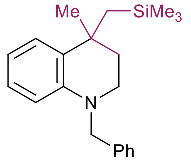

Following General Procedure 8, using *N*-benzyl-*N*-methylaniline **1q** (39.1 mg, 0.200 mmol), methylallyltrimethylsilane **2a** (80 µL, 0.44 mmol) in DCE (0.50 mL) for 22 h at 85 °C, gave the compound **3q** (55% spectroscopic yield).

Following General Procedure 9, using *N*-benzyl-*N*-methylaniline **1q** (39.5 mg, 0.200 mmol), methylallyltrimethylsilane **2a** (0.10 mL, 0.60 mmol), 2,6-dichloropyridine (3.10 mg, 20.0 µmol) in *o*-DCB (1.00 mL) for 22 h at 120 °C, gave the compound **3q** (62% spectroscopic yield). Purification via flash column chromatography on silica gel (eluent = 10% DCM/petroleum ether) gave the title compound **3q** as a clear oil (21.4 mg, 66.1 µmol, 33%).

R_f = 0.3 (10% DCM/petroleum ether). ^{1}H NMR (400 MHz, $CDCl_3$) δ_H 7.35-7.32 (2H, m, ArH), 7.29-7.25 (3H, m, ArH), 7.23 (1H, dd, *J* = 7.7, 1.6 Hz, ArH), 6.99-6.04 (1H, m, ArH), 6.63 (1H, app t, *J* = 7.4 Hz, ArH), 6.50 (1H, d, *J* = 8.3 Hz, ArH), 4.52 (2H, s, NCH_2), 3.39 (2H, t, *J* = 6.0 Hz, NCH_2), 1.96 (1H, dt, *J* = 13.0, 6.0 Hz, CH_2), 1.81 (1H, dt, *J* = 13.0, 6.0 Hz, CH_2), 1.41 (3H, s, CH_3), 1.21 (1H, d, *J* = 14.8 Hz, $SiCH_2$), 1.15 (1H, d, *J* = 14.8 Hz, $SiCH_2$), -0.02 (9H, s, $SiCH_3$); $^{13}C\{^1H\}$ NMR ($CDCl_3$, 101 MHz) δ_C 144.0 (C), 138.9 (C), 132.3 (C), 128.6 (CH), 126.7 (CH), 126.6 (CH), 125.8 (CH), 115.7 (CH), 111.1 (CH), 55.2 (NCH_2), 46.1 (NCH_2), 36.8 (CH_2), 34.8 (C), 32.2 (CH_2), 31.7 (CH_3), 0.76 ($SiCH_3$). HRMS (ESI$^+$) m/z: calcd for $C_{21}H_{30}NSi$ [M+H]$^+$ 324.2142; found 324.2140.

1-Benzyl-4,6-dimethyl-4-((trimethylsilyl)methyl)-1,2,3,4-tetrahydroquinoline, 3r

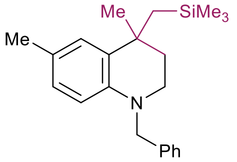

Following General Procedure 8, using *N*-benzyl-*N*,4-dimethylaniline **1r** (42.3 mg, 0.200 mmol), methylallyltrimethylsilane **2a** (80 µL, 0.44 mmol) in DCE (0.50 mL) for 22 h at 85 °C, gave the compound **3r** (77% spectroscopic yield). Purification via flash column chromatography on silica gel (eluent = 15% DCM/petroleum ether) gave the title compound **3r** as a brown oil (22 mg, 65 µmol, 33%).

Following General Procedure 9, using *N*-benzyl-*N*,4-dimethylaniline **1r** (42.3 mg, 0.200 mmol), methylallyltrimethylsilane **2a** (0.10 mL, 0.60 mmol), 2,6-dichloropyridine (3.20 mg, 20.0 µmol) in *o*-DCB (1.00 mL) for 22 h at 120 °C, gave the compound **3r** (70% spectroscopic yield).

R_f = 0.3 (15% DCM/petroleum ether). ^{1}H NMR (400 MHz, $CDCl_3$) δ_H 7.40-7.20 (5H, m), 7.07 (1H, d, *J* =2.2 Hz), 6.80 (1H, ddd, *J* = 8.7, 2.2, 0.8 Hz), 6.45 (1H, d, *J* = 8.7 Hz), 4.51 (2H, s), 3.37 (2H, t, *J* = 6.0 Hz), 2.27 (3H, s) 1.98 (1H, dt, *J* = 13.0, 5.8 Hz), 1.83 (1H, dt, *J* = 13.0, 5.8 Hz), 1.43 (3H, s), 1.24 (1H, d, *J* = 14.8 Hz), 1.17 (1H, d, *J* = 14.8 Hz), 0.02 (9H, s); ^{13}C{^{1}H} NMR ($CDCl_3$, 101 MHz) δ_C 141.9, 139.2, 132.5, 128.5, 127.1, 126.8, 126.7, 126.6, 124.6, 111.2, 55.5), 46.2, 37.0, 34.8), 32.5, 31.9, 20.5, 0.81. HRMS (ESI$^+$) m/z: calcd for $C_{22}H_{31}NSi$ [M]$^+$ 337.2220; found 337.2221.

1-Benzhydryl-4-methyl-4-((trimethylsilyl)methyl)-1,2,3,4-tetrahydroquinoline, 3s

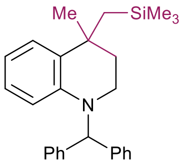

Following General Procedure 8, using *N*-benzhydryl-*N*-methylaniline **1s** (54.5 mg, 0.200 mmol), methylallyltrimethylsilane **2a** (80 µL, 0.44 mmol) in DCE (0.50 mL) for 22 h at 85 °C, gave the compound **3s** (24% spectroscopic yield).

Following General Procedure 8, using *N*-benzhydryl-*N*-methylaniline **1s** (54.3 mg, 0.200 mmol), methylallyltrimethylsilane **2a** (0.18 mL, 1.0 mmol) in DCE (0.50 mL) for 120 h at 85 °C, gave the compound **3s** (32% spectroscopic yield). Purification via flash column chromatography on silica gel (eluent = 10% DCM/petroleum ether) gave the title compound **3s** as a colourless oil (25 mg, 63 µmol, 31%).

R_f = 0.3 (10% DCM/petroleum ether). ^{1}H NMR (400 MHz, $CDCl_3$) δ_H 7.37-7.29 (6H, m, ArH), 7.25-7.22 (5H, m, ArH), 7.02- 6.94 (1H, m, ArH), 6.65 (1H, app t, *J* = 7.4 Hz, ArH), 6.55 (1H, d, *J* = 8.2 Hz, ArH), 6.19 (1H, s, NCH), 3.1-3.03 (1H, m, NCH_2), 3.00-2.90 (1H, m, NCH_2), 1.83-1.75 (1H, m, CH_2), 1.75-1.67 (1H, m, CH_2), 1.37 (3H, s, CH_3), 1.08 (2H, m, $SiCH_2$), -0.06 (9H, s, $SiCH_3$); ^{13}C{^{1}H} NMR ($CDCl_3$, 101 MHz) δ_C 144.2 (C), 140.7 (C), 140.0 (C), 133.0 (C), 129.3 (CH), 128.8 (CH), 128.4 (CH), 128.3 (CH), 127.2 (CH), 127.1 (CH), 126.8 (CH), 125.3 (CH), 115.7 (CH), 111.5 (CH), 65.9 (NCH), 41.3 (NCH_2), 36.9 (CH_2), 34.9 (C), 31.2 (CH_2), 30.9 (CH_3), 0.67 ($SiCH_3$). HRMS (ASAP$^+$) m/z: calcd for $C_{27}H_{34}NSi$ [M+H]$^+$ 400.2455; found 400.2444.

1-Benzhydryl-4,6-dimethyl-4-((trimethylsilyl)methyl)-1,2,3,4-tetrahydroquinoline, 3t

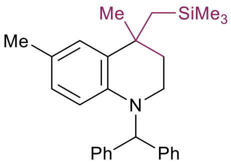

Following General Procedure 8, using *N*-benzhydryl-*N*,4-dimethylaniline **1t** (57.3 mg, 0.200 mmol), methylallyltrimethylsilane **2a** (80 µL, 0.44 mmol) in DCE (0.50 mL) for 22 h at 85 °C, gave the compound **3t** (58% spectroscopic yield). Purification via flash column chromatography on silica gel

(eluent = 10% DCM/petroleum ether) gave the title compound **3t** as a yellow oil (48 mg, 0.11 mmol, 57%).

Large scale procedure:

An oven-dried J. Youngs flask equipped with a magnetic stir bar was cooled under vacuum and charged with N_2 gas. $H_2O{\cdot}B(C_6F_5)_3$ (277 mg, 0.520 mmol) was added to the J. Youngs flask and the atmosphere cycled three times via vacuum-N_2 backfills. Using standard syringe-septa techniques, DCE (4.0 mL) was added and allowed to stir. Et_3SiH (170 µL, 1.06 mmol) was added to the solution and allowed to stir for 10 mins. *N*-Benzhydryl-*N*,4-dimethylaniline **1t** (1.5 g, 5.2 mmol) was weighed into a vial and sealed with a septa. The atmosphere in the vial was cycled three times via vacuum-N_2 backfills and the amine was subsequently dissolved in DCE (8.0 mL). The solution was transferred to the J. Youngs flask containing dried $B(C_6F_5)_3$ using standard syringe-septa techniques. The vial was washed with DCE (1.0 mL) and the washings were transferred to the reaction flask. Methylallyltrimethylsilane **2a** (2.02 mL, 11.5 mmol) was added before the J. Youngs flask was sealed, and the mixture was stirred for 22 h at 85 °C. Saturated $NaHCO_3$ (40 mL) was added, and the mixture vigorously stirred. The aqueous phase was separated and extracted with CH_2Cl_2 (3 × 10 mL). The combined organic phases were dried over $MgSO_4$ and the solvent removed in *vacuo* giving the compound **3t** in 52% spectroscopic yield. The crude material was purified via flash column chromatography on silica gel (eluent = 10% DCM/petroleum ether) to obtain the pure tetrahydroquinoline **3t** as a yellow oil (0.80 g, 1.9 mmol, 37%).

R_f = 0.3 (10% DCM/petroleum ether). ^{1}H NMR (400 MHz, $CDCl_3$) δ_H 7.38-7.29 (6H, m, ArH), 7.25-7.22 (4H, m, ArH), 7.06 (1H, d, *J* = 1.9 Hz, ArH), 6.80 (1H, dd, *J* = 8.3, 1.9 Hz, ArH), 6.47 (1H, d, *J* = 8.3 Hz, ArH), 6.16 (1H, s, NCH), 3.10-3.01 (1H, m, NCH_2), 3.00-2.91 (1H, m, NCH_2), 2.26 (3H, s, CH_3), 1.86-1.75 (1H, m, CH_2), 1.75-1.65 (1H, m, CH_2), 1.37 (3H, s, CH_3), 1.11 (1H, d, *J* = 14.9 Hz, $SiCH_2$), 1.07 (1H, d, *J* = 14.9 Hz, $SiCH_2$), -0.05 (9H, s, $SiCH_3$); ^{13}C{^{1}H} NMR ($CDCl_3$, 101 MHz) δ_C 141.9 (C), 140.9 (C), 140.3 (C), 133.1 (C), 129.2 (CH), 128.8 (CH), 128.3 (CH), 128.2 (CH), 127.2 (CH), 127.0 (CH), 126.2 (CH), 124.5 (C), 111.5 (CH), 65.9 (NCH), 41.2 (NCH_2), 37.0 (CH_2), 34.8 (C), 31.5 (CH_2), 31.0 (CH_3), 20.5 (CH_3), 0.73 ($SiCH_3$). HRMS (ESI$^+$) m/z: calcd for $C_{28}H_{35}NSi$ [M]$^+$ 413.2533; found 413.2539.

1,6-Dibenzhydryl-4-methyl-4-((trimethylsilyl)methyl)-1,2,3,4-tetrahydroquinoline, 3u

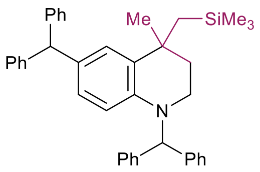

Following General Procedure 8, using *N*,4-dibenzhydryl-*N*-methylaniline **1u** (87.4 mg, 0.200 mmol), methylallyltrimethylsilane **2a** (80 µL, 0.44 mmol) in DCE (0.50 mL) for 22 h at 85 °C, gave the

compound **3u** (48% spectroscopic yield). Purification via flash column chromatography on silica gel (eluent = 20% DCM/petroleum ether) gave the title compound **3u** as a red oil (51 mg, 90 µmol, 45%).

Following General Procedure 9, using *N*,4-dibenzhydryl-*N*-methylaniline **1u** (87.9 mg, 0.200 mmol), methylallyltrimethylsilane **2a** (0.10 mL, 0.60 mmol), 2,6-dichloropyridine (3.00 mg, 20.0 µmol) in *o*-DCB (0.50 mL) for 22 h at 120 °C, gave the compound **3u** (32% spectroscopic yield).

R_f = 0.3 (20% DCM/petroleum ether). ^{1}H NMR (400 MHz, $CDCl_3$) δ_H 7.40-7.29 (8H, m, ArH), 7.23-7.15 (12H, m, ArH), 6.98 (1H, d, *J* = 1.9 Hz, ArH), 6.65 (1H, dd, *J* = 8.4, 1.9 Hz, ArH), 6.44 (1H, d, *J* = 8.4 Hz, ArH), 6.15 (1H, s, NCH), 5.44 (1H, s, CH), 3.07 (1H, ddd, *J* = 11.8, 7.0, 4.9 Hz, NCH_2), 2.96 (1H, ddd, *J* = 11.8, 8.0, 4.9 Hz, NCH_2), 1.77 (1H, ddd, *J* = 12.6, 7.0, 4.9 Hz, NCH_2), 1.69 (1H, ddd, *J* = 12.6, 8.0, 4.9 Hz, NCH_2), 1.28 (3H, s, CH_3), 1.00 (1H, d, *J* = 14.9 Hz, $SiCH_2$), 0.95 (1H, d, *J* = 14.9 Hz, $SiCH_2$), -0.17 (9H, s, $SiCH_3$); $^{13}C\{^1H\}$ NMR ($CDCl_3$, 101 MHz) δ_C 144.9 (C), 144.8 (C), 142.7 (C), 140.9 (C), 140.3 (C), 132.2 (C), 130.6 (C), 129.5 (CH), 129.4 (CH), 129.2 (CH), 128.8 (CH), 128.3 (CH), 128.2 (CH), 128.1 (CH), 128.0 (CH), 127.5 (CH), 127.2 (CH), 127.1 (CH), 127.0 (CH), 126.7 (CH), 125.9 (CH), 111.2 (CH), 66.0 (NCH), 56.4 (CH), 41.3 (NCH_2), 37.3 (CH_2), 34.8 (C), 31.4 (CH_3), 30.9 (CH_2), 0.54 ($SiCH_3$). HRMS ($ASAP^+$) m/z: calcd for $C_{40}H_{44}NSi$ $[M+H]^+$ 566.3238; found 566.3242.

2,4,6-Trimethyl-1-(*p*-tolyl)-4-((trimethylsilyl)methyl)-1,2,3,4-tetrahydroquinoline, 3v

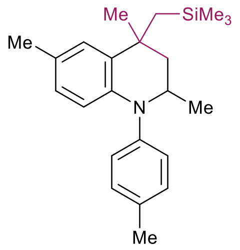

Following General Procedure 8, using *N*-ethyl-4-methyl-*N*-(*p*-tolyl)aniline **1v** (45.0 mg, 0.200 mmol), methylallyltrimethylsilane **2a** (80 µL, 0.44 mmol) in DCE (0.50 mL) for 22 h at 85 °C, gave the compound **3v** (29% spectroscopic yield, 1:1.1 *dr*).

Following General Procedure 9, using *N*-ethyl-4-methyl-*N*-(*p*-tolyl)aniline **1v** (44.6 mg, 0.200 mmol), methylallyltrimethylsilane **2a** (0.10 mL, 0.60 mmol), 2,6-dichloropyridine (2.90 mg, 20.0 µmol) in *o*-DCB (0.50 mL) for 22 h at 120 °C, gave the compound **3v** (26% spectroscopic yield, 1:1.1 *dr*).

Following General Procedure 8, using *N*-ethyl-4-methyl-*N*-(*p*-tolyl)aniline **1v** (45.2 mg, 0.200 mmol), methylallyltrimethylsilane **2a** (0.18 mL, 1.0 mmol) in DCE (0.50 mL) for 120 h at 85 °C, gave the compound **3v** (60% spectroscopic yield, 1:1.1 *dr*). Purification via flash column chromatography on silica gel (eluent = 5% DCM/petroleum ether) gave the title compound **3v** as a colourless oil (major isomer: 14 mg, 40 µmol, 20% and minor isomer: 9.7 mg, 30 µmol, 14%).

Major isomer (1:1.1 dr): R_f = 0.2 (5% DCM/petroleum ether). ^{1}H NMR (400 MHz, $CDCl_3$) δ_H 7.21 (2H, d, *J* = 7.9 Hz, ArH), 7.07-7.01 (3H, m, ArH), 6.63 (1H, d, *J* = 8.3 Hz, ArH), 6.03 (1H, d, *J* = 8.3 Hz, ArH), 3.66-3.58 (1H, m, NCH), 2.39 (3H, s, CH_3), 2.22 (3H, s, CH_3), 1.94 (1H, app t, *J* = 12.5, 11.8 Hz, ArH), 1.80 (1H, dd, *J* = 13.1, 2.4 Hz, CH_2), 1.43 (3H, s, CH_3), 1.31 (1H, d, *J* = 14.8 Hz, $SiCH_2$), 1.22 (1H, d, *J* = 14.8 Hz, $SiCH_2$), 0.99 (3H, d, *J* = 6.1 Hz, CH_3), -0.01 (9H, s, $SiCH_3$); ^{13}C{^{1}H} NMR ($CDCl_3$, 101 MHz) δ_C 144.5 (C), 144.1 (C), 135.6 (C), 132.7 (C), 130.2 (CH), 129.5 (CH), 127.3 (CH), 126.6 (CH), 126.0 (C), 116.0 (CH), 50.2 (NCH), 46.9 (CH_2), 35.3 (CH_3), 35.1 (C), 33.1 (CH_2), 22.5 (CH_3), 21.1 (CH_3), 20.5 (CH_3), 0.89 ($SiCH_3$). HRMS (ESI$^+$) m/z: calcd for $C_{23}H_{34}NSi$ [M+H]$^+$ 352.2455; found 352.2463.

Minor isomer (1:1.1 dr): R_f = 0.1 (5% DCM/petroleum ether). ^{1}H NMR (400 MHz, $CDCl_3$) δ_H 7.21 (2H, d, *J* = 7.8 Hz, ArH), 7.06 (2H, d, *J* = 7.8 Hz, ArH), 7.03 (1H, s, ArH), 6.68 (1H, d, *J* = 8.3 Hz, ArH), 6.21 (1H, d, *J* = 8.3 Hz, ArH), 3.78-3.72 (1H, m, NCH), 2.39 (3H, s, CH_3), 2.24 (3H, s, CH_3), 2.00 (1H, dd, *J* = 13.2, 2.9 Hz, CH_2), 1.77 (1H, app t, *J* = 12.7, 11.6 Hz, ArH), 1.46 (3H, s, CH_3), 1.24 (1H, d, *J* = 15.0 Hz, $SiCH_2$), 1.15 (1H, d, *J* = 15.0 Hz, $SiCH_2$), 1.07 (3H, d, *J* = 6.1 Hz, CH_3), 0.04 (9H, s, $SiCH_3$); ^{13}C{^{1}H} NMR ($CDCl_3$, 101 MHz) δ_C 144.7 (C), 142.4 (C), 135.1 (C), 134.7 (C), 130.2 (CH), 128.0 (CH), 126.7 (CH), 126.6 (C), 126.0 (CH), 116.9 (CH), 50.8 (NCH), 47.0 (CH_2), 35.0 (C), 34.5 (CH_2), 29.9 (CH_3), 22.2 (CH_3), 21.0 (CH_3), 20.6 (CH_3), 0.88 ($SiCH_3$). HRMS (ESI$^+$) m/z: calcd for $C_{23}H_{34}NSi$ [M+H]$^+$ 352.2455; found 352.2465.

4,6-Dimethyl-2-propyl-1-(*p*-tolyl)-4-((trimethylsilyl)methyl)-1,2,3,4-tetrahydroquinoline, 3w

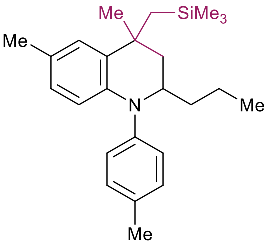

Following General Procedure 8, using *N*-butyl-4-methyl-*N*-(*p*-tolyl)aniline **1w** (50.7 mg, 0.200 mmol), methylallyltrimethylsilane **2a** (80 µL, 0.44 mmol) in DCE (0.50 mL) for 22 h at 85 °C, gave the compound **3w** (21% spectroscopic yield, 1:1.1 *dr*).

Following General Procedure 9, using *N*-butyl-4-methyl-*N*-(*p*-tolyl)aniline **1w** (50.7 mg, 0.200 mmol), methylallyltrimethylsilane **2a** (0.10 mL, 0.60 mmol), 2,6-dichloropyridine (3.30 mg, 20.0 µmol) in *o*-DCB (0.50 mL) for 22 h at 120 °C, gave the compound **3w** (13% spectroscopic yield, 1:1.1 *dr*).

Following General Procedure 8, using *N*-butyl-4-methyl-*N*-(*p*-tolyl)aniline **1w** (50.7 mg, 0.200 mmol), methylallyltrimethylsilane **2a** (0.18 mL, 1.0 mmol) in DCE (0.50 mL) for 120 h at 85 °C, gave the compound **3w** (40% spectroscopic yield, 1:1.1 *dr*). Purification via flash column chromatography on silica gel (eluent = 5% DCM/petroleum ether) gave the title compound **3w** as a colourless oil (14 mg, 38 µmol, 19%, 1:1.3 *dr*).

Mixture of diastereoisomers (1:1.3 dr): R_f = 0.5 (5% DCM/petroleum ether). ^{1}H NMR (400 MHz, $CDCl_3$, asterisk indicate resolved minor diastereoisomer peaks) δ_H 7.21 -7.17 (2H, m, ArH*), 7.17 – 7.12 (2H, m, ArH), 7.07 – 6.99 (6H, m, ArH, ArH*), 6.71 (1H, ddd, *J* = 8.3, 2.1, 0.6 Hz, ArH, major), 6.67 – 6.61 (1H, m, ArH*), 6.39 (1H, d, *J* = 8.3 Hz, ArH), 6.10 (1H, d, *J* = 8.2 Hz, ArH*), 3.70 – 3.60 (1H, m, NCH), 3.57 – 3.45 (1H, m, NCH*), 2.38 (3H, s, CH_3*), 2.35 (3H, s, CH_3), 2.24 (3H, s, CH_3), 2.22 (3H, s, CH_3*), 2.06 (1H, dd, *J* = 13.4, 4.8 Hz, CH_2), 1.92 – 1.82 (2H, m, CH_2*), 1.69 (1H, dd, *J* = 13.4, 10.1 Hz, CH_2), 1.61 – 1.53 (1H, m, CH_2), 1.45 (3H, s, CH_3), 1.44 – 1.39 (3H, m, CH_2*, CH_2), 1.38 (3H, s, CH_3*), 1.37 – 1.31 (1H, m, CH_2), 1.31 – 1.15 (5H, m, CH_2*, CH_2), 1.13 (1H, d , *J* = 15.1 Hz, $SiCH_2$), 1.09 (1H, d , *J* = 15.1 Hz, $SiCH_2$), 0.85 (3H, t, *J* = 7.2 Hz, CH_3), 0.81 (3H, t, *J* = 6.9 Hz, CH_3*), 0.00 (9H, s, $SiCH_3$*), -0.02 (9H, s, $SiCH_3$); $^{13}C\{^1H\}$ NMR ($CDCl_3$, 101 MHz, asterisk indicate resolved minor diastereoisomer peaks) δ_C 145.3 (C), 144.9 (C*), 143.9 (C), 141.7 (C), 136.5 (C), 134.9 (C), 133.7 (C*), 133.1 (C), 130.1 (CH*), 130.0 (CH), 128.5 (CH), 127.5 (C), 126.9 (CH), 126.7 (CH), 126.6 (CH*), 126.4 (C), 125.8 (CH), 125.3 (CH), 118.7 (CH), 117.0 (CH*), 55.4 (NCH), 54.7 (NCH*), 44.3 (CH_2), 43.2 (CH_2*), 37.6 (CH_2), 37.1 (CH_2*), 34.9 (C*), 34.8 (C), 34.4 (CH_3*), 33.2 ($SiCH_2$), 32.8, ($SiCH_2$*) 29.9 (CH_3), 21.0 (CH_3*), 20.9 (CH_3), 20.7 (CH_3), 20.5 (CH_3*), 18.4 (CH_2), 18.1 (CH_2*), 14.4 (CH_3), 14.3 (CH_3*), 1.0 ($SiCH_3$*), 0.9 ($SiCH_3$). HRMS (ESI$^+$) m/z: calcd for $C_{25}H_{38}NSi$ $[M+H]^+$ 380.2768; found 380.2776.

2-Cyclohexyl-4,6-dimethyl-1-(*p*-tolyl)-4-((trimethylsilyl)methyl)-1,2,3,4-tetrahydroquinoline, 3x

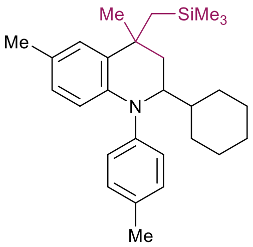

Following General Procedure 8, using *N*-(cyclohexylmethyl)-4-methyl-*N*-(*p*-tolyl)aniline **1x** (58.7 mg, 0.200 mmol), methylallyltrimethylsilane **2a** (80 μL, 0.44 mmol) in DCE (0.50 mL) for 22 h at 85 °C, gave the compound **3x** (30% spectroscopic yield, 1:1.2 *dr*).

Following General Procedure 8, using *N*-(cyclohexylmethyl)-4-methyl-*N*-(*p*-tolyl)aniline **1x** (58.7 mg, 0.200 mmol), methylallyltrimethylsilane **2a** (0.18 mL, 1.0 mmol) in DCE (0.50 mL) for 120 h at 85 °C, gave the compound **3x** (47% spectroscopic yield, 1:1.3 *dr*). Purification via flash column chromatography on silica gel (eluent = 100% petroleum ether) gave the title compound **3x** (major diastereoisomer) as a colourless oil (14 mg, 33 μmol, 17%). Minor diastereoisomer could not be separated from starting amine **1x**.

Major isomer (1:1.3 dr): R_f = 0.5 (15% DCM/petroleum ether). ^{1}H NMR (400 MHz, $CDCl_3$) δ_H 7.20-7.15 (2H, m, ArH), 7.05 (1H, d, *J* = 1.9 Hz, ArH, ArH), 7.04 - 7.00 (2H, m, ArH), 6.66 (1H, d, *J* = 8.3, 1.6 Hz, ArH), 6.20 (1H, d, *J* = 8.3 Hz, ArH), 3.42 (1H, dt, *J* = 11.3, 3.5 Hz, NCH), 2.37 (3H, s, CH_3),

2.23 (3H, s, CH_3), 1.95 -1.86 (2H, m, CH_2), 1.78 - 1.68 (3H, m, CH_2), 1.66 - 1.60 (1H, m, CH_2), 1.48 - 1.42 (2H, m, CH, CH_2), 1.34 (3H, s, CH_3), 1.31 (1H, d, *J* = 14.9 Hz, $SiCH_2$), 1.25 (1H, d, *J* = 14.9 Hz, $SiCH_2$) 1.16 - 1.08 (3H, m, CH_2), 1.03 - 0.95 (2H, m, CH_2), 0.02 (9H, s, $SiCH_3$); $^{13}C\{^1H\}$ NMR ($CDCl_3$, 101 MHz) δ_C 145.6 (C), 144.1 (C), 134.8 (C), 134.3 (C), 130.1 (CH), 127.2 (CH), 126.8 (C), 126.6 (CH), 118.3 (CH), 59.9 (NCH), 40.2 (CH), 38.2 (CH_2), 34.7 (C), 33.6 (CH_3), 32.7 (CH_2), 30.4 (CH_2), 27.0 (CH_2), 26.9 (CH_2), 26.4 (CH_2), 26.2 (CH_2), 21.0 (CH_3), 20.6 (CH_3), 1.0 ($SiCH_3$). HRMS (ESI+) m/z: calcd for $C_{28}H_{42}NSi$ $[M+H]^+$ 420.3081; found 420.3089.

6,8-Dimethyl-6-((trimethylsilyl)methyl)-2,3,4,4a,5,6-hexahydro-1*H*-pyrido[1,2-*a*]quinoline, 3y

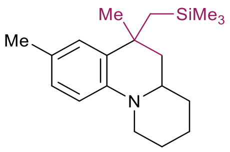

Following General Procedure 8, using 1-(*p*-tolyl)piperidine **1y** (35.4 mg, 0.200 mmol), methylallyltrimethylsilane **2a** (80 µL, 0.44 mmol) in DCE (0.50 mL) for 22 h at 85 °C, gave the compound **3y** (21% spectroscopic yield, 1:1.4 *dr*).

Following General Procedure 9, using 1-(*p*-tolyl)piperidine **1y** (35.1 mg, 0.200 mmol), methylallyltrimethylsilane **2a** (80 µL, 0.44 mmol), 2,6-dichloropyridine (3.00 mg, 20.0 µmol) in *o*-DCB (0.50 mL) for 22 h at 120 °C, gave the compound **3y** (25% spectroscopic yield, 1:1.3 *dr*).

Following General Procedure 8, using 1-(*p*-tolyl)piperidine **1y** (87.6 mg, 0.500 mmol), methylallyltrimethylsilane **2a** (0.31 mL, 1.8 mmol) in DCE (0.50 mL) for 120 h at 85 °C, gave the compound **3y** (25% spectroscopic yield, 1:1.3 *dr*). Purification via flash column chromatography on silica gel (eluent = 20% DCM/petroleum ether) gave the title compound **3y** as a colourless oil (major isomer: 17 mg, 60 µmol, 11% and minor isomer: 12 mg, 40 µmol, 8%).

Major isomer (1:1.4 dr): R_f = 0.3 (20% DCM/petroleum ether). ^1H NMR (400 MHz, $CDCl_3$) δ_H 7.03 (1H, d, *J* = 1.9 Hz, ArH), 6.87 (1H, dd, *J* = 8.4, 1.9 Hz, ArH), 6.74 (1H, d, *J* = 8.4 Hz, ArH), 3.90-3.83 (1H, m, NCH_2), 2.74 (1H, tt, *J* = 10.8, 2.7 Hz, NCH), 2.45 (1H, td, *J* = 12.2, 2.3 Hz, NCH_2), 2.24 (3H, s, CH_3), 1.87-1.81 (3H, m, CH_2), 1.72-1.59 (3H, m, CH_2), 1.43-1.35 (2H, m, CH_2), 1.33 (3H, s, CH_3), 1.22 (1H, d, *J* = 14.7 Hz, $SiCH_2$), 1.15 (1H, d, *J* = 14.7 Hz, $SiCH_2$), -0.08 (9H, s, $SiCH_3$); $^{13}C\{^1H\}$ NMR ($CDCl_3$, 101 MHz) δ_C 144.2 (C), 134.4 (C), 128.0 (CH), 127.0 (CH), 126.4 (C), 113.0 (CH), 53.2 (NCH), 48.3 (CH_2), 45.8 (CH_2), 35.8 (CH_3), 34.9 (C), 34.1 (CH_2), 33.5 (CH_2), 26.3 (CH_2), 24.0 (CH_2), 20.4 (CH_3), 0.80 ($SiCH_3$). HRMS (ESI^+) m/z: calcd for $C_{19}H_{32}NSi$ $[M+H]^+$ 302.2299; found 302.2312.

Minor isomer (1:1.4 dr): R_f = 0.2 (20% DCM/petroleum ether). ^1H NMR (400 MHz, $CDCl_3$) δ_H 7.00 (1H, d, *J* = 1.8 Hz, ArH), 6.86 (1H, dd, *J* = 8.4, 1.8 Hz, ArH), 6.72 (1H, d, *J* = 8.4 Hz, ArH), 3.96-3.89 (1H, m, NCH), 2.91 (1H, tt, *J* = 11.0, 2.7 Hz, CH_2), 2.58 (1H, td, *J* = 12.4, 2.7 Hz, CH_2), 2.24 (3H, s, CH_3), 1.85-1.77 (3H, m, CH_2), 1.71-1.60 (3H, m, CH_2), 1.48-1.39 (1H, m, CH_2), 1.38 (3H, s, CH_3), 1.34-

1.30 (1H, m, CH_2), 1.17 (1H, d, *J* = 15.0 Hz, $SiCH_2$), 1.05 (1H, d, *J* = 15.0 Hz, $SiCH_2$), 0.04 (9H, s, $SiCH_3$); $^{13}C\{^1H\}$ NMR ($CDCl_3$, 101 MHz) δ_C 142.7 (C), 136.0 (C), 127.1 (CH), 126.8 (CH), 126.2 (C), 112.9 (CH), 53.3 (NCH), 48.1 (CH_2), 45.3 (CH_2), 35.5 (CH_2), 35.0 (C), 33.6 (CH_2), 30.4 (CH_3), 26.1 (CH_2), 24.4 (CH_2), 20.4 (CH_3), 0.89 ($SiCH_3$). HRMS (ESI$^+$) m/z: calcd for $C_{19}H_{32}NSi$ $[M+H]^+$ 302.2299; found 302.2309.

3,5-Dimethyl-5-((trimethylsilyl)methyl)-5,6,6a,7,8,9,10,11-octahydroazepino[1,2-*a*]quinoline, 3z

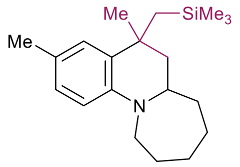

Following General Procedure 8, using 1-(*p*-tolyl)azepane **1z** (38 mg, 0.200 mmol), methylallyltrimethylsilane **2a** (80 µL, 0.44 mmol) in DCE (0.50 mL) for 22 h at 85 °C, gave the compound **3z** (48% spectroscopic yield, 1:1.2 *dr*). Purification via flash column chromatography on silica gel (eluent = 10% DCM/petroleum ether) gave the title compound **3z** as a colourless oil (major isomer: 12 mg, 40 µmol, 19% and minor isomer: 10 mg, 30 µmol, 16%).

Major isomer (1:1.2 dr): R_f = 0.4 (10% DCM/petroleum ether). ^1H NMR (400 MHz, $CDCl_3$) δ_H 6.95 (1H, d, *J* = 1.9 Hz, ArH), 6.85 (1H, dd, *J* = 8.3, 1.9 Hz, ArH), 6.47 (1H, d, *J* = 8.3 Hz, ArH), 3.66-3.61 (1H, m, NCH), 3.56-3.49 (1H, m, CH_2), 3.24 (1H, ddd, *J* = 14.9, 11.3, 2. Hz, CH_2), 2.23 (3H, s, CH_3), 1.93-1.45 (10H, m, CH_2), 1.39 (3H, s, CH_3), 1.07 (1H, d, *J* = 14.9 Hz, $SiCH_2$), 0.95 (1H, d, *J* = 14.9 Hz, $SiCH_2$), -0.01 (9H, s, $SiCH_3$); $^{13}C\{^1H\}$ NMR ($CDCl_3$, 101 MHz) δ_C 142.3 (C), 133.1 (C), 127.2 (CH), 125.2 (CH), 123.4 (C), 110.3 (CH), 53.5 (NCH), 49.0 (CH_2), 45.3 (CH_2), 36.7 (CH_2), 34.8 (C), 32.1 (CH_2), 29.4 (CH_2), 29.1 (CH_3), 27.6 (CH_2), 23.3 (CH_2), 20.4 (CH_3), 0.78 ($SiCH_3$). HRMS (ESI$^+$) m/z: calcd for $C_{20}H_{34}NSi$ $[M+H]^+$ 316.2455; found 316.2462.

Minor isomer (1:1.2 dr): R_f = 0.2 (10% DCM/petroleum ether). ^1H NMR (400 MHz, $CDCl_3$) δ_H 6.99 (1H, d, *J* = 2.0 Hz, ArH), 6.84 (1H, dd, *J* = 8.3, 2.0 Hz, ArH), 6.49 (1H, d, *J* = 8.3 Hz, ArH), 3.56-3.46 (2H, m, NCH, CH_2), 3.25-3.18 (1H, m, CH_2), 2.23 (3H, s, CH_3), 1.94-1.86 (1H, m, CH_2), 1.81-1.45 (9H, m, CH_2), 1.25 (3H, s, CH_3), 1.21 (1H, d, *J* = 14.6 Hz, $SiCH_2$), 1.18 (1H, d, *J* = 14.6 Hz, $SiCH_2$), -0.03 (9H, s, $SiCH_3$); $^{13}C\{^1H\}$ NMR ($CDCl_3$, 101 MHz) δ_C 143.1 (C), 131.7 (C), 127.1 (CH), 126.5 (CH), 123.5 (C), 110.8 (CH), 53.0 (NCH), 48.8 (CH_2), 44.3 (CH_2), 36.6 (CH_2), 35.1 (C), 33.0 (CH_3), 32.2 (CH_2), 29.7 (CH_2), 28.4 (CH_2), 23.4 (CH_2), 20.4 (CH_3), 0.98 ($SiCH_3$). HRMS (ESI$^+$) m/z: calcd for $C_{20}H_{34}NSi$ $[M+H]^+$ 316.2455; found 316.2456.

5,7-Dimethyl-5-((trimethylsilyl)methyl)-4,5-dihydropyrrolo[1,2-*a*]quinoline, 3aa

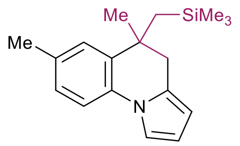

Following General Procedure 8, using 1-(*p*-tolyl)pyrrolidine **1aa** (32.4 mg, 0.200 mmol), methylallyltrimethylsilane **2a** (80 µL, 0.44 mmol) in DCE (0.50 mL) for 22 h at 85 °C, gave the compound **3aa** (17% spectroscopic yield).

Following General Procedure 9, using 1-(*p*-tolyl)pyrrolidine **1aa** (32.0 mg, 0.200 mmol), methylallyltrimethylsilane **2a** (0.10 mL, 0.60 mmol), 2,6-dichloropyridine (3.20 mg, 20.0 µmol) in *o*-DCB (0.50 mL) for 22 h at 120 °C, gave the compound **3aa** (14% spectroscopic yield).

Following General Procedure 8, using 1-(*p*-tolyl)pyrrolidine **1aa** (80.2 mg, 0.50 mmol), methylallyltrimethylsilane **2a** (0.31 mL, 1.8 mmol) in DCE (0.50 mL) for 120 h at 85 °C, gave the compound **3aa** (25% spectroscopic yield). Purification via flash column chromatography on silica gel (eluent = 10% DCM/petroleum ether) gave the title compound **3aa** as a colourless oil (32 mg, 0.11 mmol, 23%).

R_f = 0.3 (10% DCM/petroleum ether). ^{1}H NMR (400 MHz, $CDCl_3$) δ_H 7.25 (1H, d, *J* = 8.1 Hz, ArH), 7.21 (1H, d, *J* = 1.5 Hz, ArH), 7.15 (1H, dd, *J* = 2.8, 1.5 Hz, ArH), 7.07 (1H, dd, *J* = 8.1, 1.3 Hz, ArH), 6.28 (1H, t, *J* = 3.1 Hz, ArH), 6.05-6.00 (1H, m, ArH), 2.86 (1H, d, *J* = 14.8 Hz, CH_2), 2.76 (1H, d, *J* = 14.8 Hz, CH_2), 2.38 (3H, s, CH_3), 1.44 (3H, s, CH_3), 0.97 (1H, d, *J* = 14.8 Hz, $SiCH_2$), 0.93 (1H, d, *J* = 14.8 Hz, $SiCH_2$), -0.08 (9H, s, $SiCH_3$); ^{13}C{^{1}H} NMR ($CDCl_3$, 101 MHz) δ_C 136.6 (C), 133.3 (C), 133.0 (C), 128.6 (C), 127.4 (CH), 126.2 (CH), 115.3 (CH), 113.9 (CH), 109.0 (CH), 106.3 (CH), 38.0 (CH_2), 36.3 (C), 29.6 (CH_2), 28.3 (CH_3), 21.1 (CH_3), 0.41 ($SiCH_3$). HRMS (ESI$^+$) m/z: calcd for $C_{18}H_{26}NSi$ $[M+H]^+$ 284.1829; found 284.1840.

3.4 Alkene Scope of $B(C_6F_5)_3$-Catalysed THQ synthesis

6-Methyl-4-phenyl-1-(*p*-tolyl)-4-((trimethylsilyl)methyl)-1,2,3,4-tetrahydroquinoline, 3ab

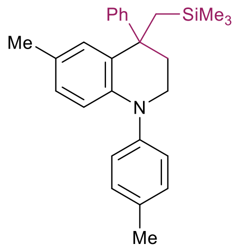

Following General Procedure 8, using *N*-methyl-di-*p*-tolylamine **1a** (42.3 mg, 0.200 mmol), phenylallyltrimethylsilane **2b** (83.8 mg, 0.440 mmol) in DCE (0.5 mL) for 22 h at 85 °C, gave the title compound **3ab** (15% spectroscopic yield).

Following General Procedure 8, using *N*-methyl-di-*p*-tolylamine **1a** (42.0 mg, 0.200 mmol), phenylallyltrimethylsilane **2b** (191 mg, 1.00 mmol) in DCE (0.5 mL) for 120 h at 85 °C, gave the title compound **3ab** (58% spectroscopic yield).

Following General Procedure 8, using *N*-methyl-di-*p*-tolylamine **1a** (42.3 mg, 0.200 mmol), phenylallyltrimethylsilane **2b** (83.8 mg, 0.440 mmol) in DCE (0.5 mL) for 44 h at 85 °C, gave the title compound **3ab** (16% spectroscopic yield). Purification via flash column chromatography on silica gel (eluent = 15% DCM/petroleum ether) gave the title compound **3ab** as a clear oil (13 mg, 30 µmol, 16%).

R_f = 0.4 (15% DCM/petroleum ether). ^{1}H NMR (400 MHz, $CDCl_3$) δ_H 7.28-7.21 (4H, m, ArH), 7.16-7.10 (3H, m, ArH), 7.06-7.05 (2H, m, ArH), 6.95 (1H, d, *J* = 1.8 Hz, ArH), 6.81 (1H, dd, *J* = 8.4, 1.8 Hz, ArH), 6.70 (1H, d, *J* = 8.4 Hz, ArH), 3.42 (1H, dt, *J* = 11.7, 3.6 Hz, NCH_2), 3.20 (1H, dt, *J* = 11.7, 3.6 Hz, NCH_2), 2.38-2.34 (1H, m, CH_2), 2.33 (3H, s, CH_3), 2.24 (3H, s, CH_3), 2.17 (1H, m, CH_2), 1.74 (1H, d, *J* = 14.8 Hz, $SiCH_2$), 1.70 (1H, d, *J* = 14.8 Hz, $SiCH_2$), -0.07 (9H, s, $SiCH_3$); $^{13}C\{^1H\}$ NMR ($CDCl_3$, 101 MHz) δ_C 152.4 (C), 146.3 (C), 142.4 (C), 132.7 (C), 130.1 (CH), 129.8 (CH), 129.6 (C), 127.8 (CH), 127.4 (CH), 127.3 (CH), 126.7 (C), 125.4 (CH), 124.3 (CH), 116.2 (CH), 47.3 (NCH_2), 43.9 (C), 38.4 (CH_2), 32.1 (CH_2), 20.8 (CH_3), 20.6 (CH_3), 0.87 ($SiCH_3$). HRMS (ESI$^+$) m/z: calcd for $C_{27}H_{33}NSi$ $[M]^+$ 399.2377; found 399.2385; calcd for $C_{27}H_{34}NSi$ $[M+H]^+$ 400.2455; found 400.2458.

4-((Dimethyl(phenyl)silyl)methyl)-4,6-dimethyl-1-(*p*-tolyl)-1,2,3,4-tetrahydroquinoline, 3ac and 4-((dimethyl(phenyl)silyl)methyl)-4,6-dimethyl-1-(*p*-tolyl)-1,4-dihydroquinoline, 3ac'

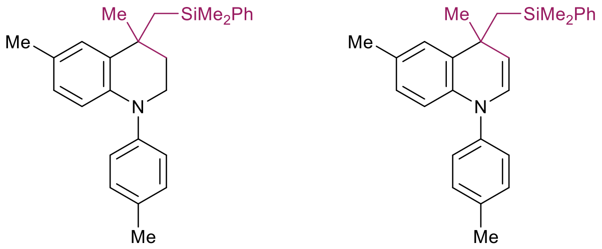

Following General Procedure 8, using *N*-methyl-di-*p*-tolylamine **1a** (42.3 mg, 0.200 mmol), dimethyl(2-methylallyl)(phenyl)silane **2c** (100 µL, 0.440 mmol) in DCE (0.5 mL) for 22 h at 85 °C, gave the compound **3ac** (37% spectroscopic yield).

Following General Procedure 8, using *N*-methyl-di-*p*-tolylamine **1a** (42.3 mg, 0.200 mmol), dimethyl(2-methylallyl)(phenyl)silane **2c** (215 µL, 1.00 mmol) in DCE (0.5 mL) for 120 h at 85 °C, gave the compound **3ac** (67% spectroscopic yield). Purification via flash column chromatography on silica gel neutralised with Et_3N (1.5 mL per 100 mL of silica gel) (eluent = 5% DCM/petroleum ether) gave the tetrahydroquinoline **3ac** with 10% as inseparable mixture with starting amine **1a** (45.6 mg, 0.11 mmol, 57%).

Following General Procedure 8, using *N*-methyl-di-*p*-tolylamine **1a** (42.3 mg, 0.200 mmol), dimethyl(2-methylallyl)(phenyl)silane **2c** (215 µL, 1.00 mmol) in DCE (0.5 mL) for 192 h at 85 °C, gave the compound **3ac** (60% spectroscopic yield). Purification via flash column chromatography on silica gel (eluent = 5% DCM/petroleum ether) gave the dihydroquinoline **3ac'** as a clear oil (23 mg, 60 µmol, 29%).

4-((Dimethyl(phenyl)silyl)methyl)-4,6-dimethyl-1-(*p*-tolyl)-1,2,3,4-tetrahydroquinoline, **3ac**: R_f = 0.5 (10% DCM/petroleum ether). ^{1}H NMR (500 MHz, $CDCl_3$) δ_H 7.51-7.47 (2H, m), 7.36-7.33 (3H, m), 7.17-7.13 (2H, m), 7.11-7.07 (2H, m), 7.01 (1H, d, *J* = 1.9 Hz), 6.76-6.71 (1H, m), 6.66 (1H, d, *J* = 8.3 Hz), 3.60 (1H, ddd, *J* = 11.7, 7.7, 4.3 Hz), 3.48 (1H, ddd, *J* = 11.7, 7.4, 4.7 Hz), 2.35 (3H, s), 2.23 (3H, s), 1.97 (1H, ddd, *J* = 13.1, 7.7, 4.7 Hz), 1.73 (1H, ddd, *J* = 13.1, 7.4, 4.3 Hz), 1.44 (2H, s), 1.37 (3H, s), 0.24 (3H, s), 0.23 (3H, s); ^{13}C{^{1}H} NMR ($CDCl_3$, 126 MHz) δ_C 146.1, 140.7, 140.5, 134.3, 133.5, 132.4, 129.8, 128.6, 127.7, 127.2, 127.1, 126.7, 123.6, 116.3, 46.9, 37.9, 35.1, 32.2, 31.1, 20.8, 20.6, -0.8 ($SiCH_3$), -1.0. HRMS (ESI$^+$) m/z: calcd for $C_{27}H_{34}NSi$ $[M+H]^+$ 400.2455; found 400.2462.

4-((Dimethyl(phenyl)silyl)methyl)-4,6-dimethyl-1-(*p*-tolyl)-1,4-dihydroquinoline, **3ac'**: R_f = 0.6 (10% DCM/petroleum ether). ^{1}H NMR (500 MHz, $CDCl_3$) δ_H 7.48-7.43 (2H, m, ArH), 7.33-7.29 (3H, m, ArH), 7.27-7.23 (2H, m, ArH), 7.18-7.14 (2H, m, ArH), 7.02 (1H, d, *J* = 1.8 Hz ArH), 6.73-6.66 (1H, m, ArH), 6.37 (1H, d, *J* = 8.3 Hz, ArH), 6.04 (1H, d, *J* = 8.0 Hz, ArH), 4.38 (1H, d, *J* = 8.0 Hz, ArH), 2.41 (3H, s, CH_3), 2.21 (3H, s, CH_3), 1.62 (1H, d, *J* = 14.7 Hz, $SiCH_2$), 1.51 (3H, s, CH_3), 1.25 (1H, d, *J* = 14.7 Hz, $SiCH_2$), 0.29 (3H, s, $SiCH_3$), 0.09 (3H, s, $SiCH_3$); ^{13}C{^{1}H} NMR ($CDCl_3$, 126 MHz) δ_C

142.0 (C), 141.1 (C), 137.4 (C), 136.0 (C), 133.5 (CH), 130.3 (CH), 130.2 (C), 129.9 (C), 128.6 (CH), 128.4 (CH), 128.0 (CH), 127.5 (CH), 127.4 (CH), 126.7 (CH), 113.3 (CH), 108.1 (CH), 37.9 (CH_3), 36.2 (CH_2), 35.8 (C), 21.1 (CH_3), 20.6 (CH_3), -1.2 ($SiCH_3$), -1.8 ($SiCH_3$). HRMS (ESI^+) m/z: calcd for $C_{27}H_{32}NSi$ $[M+H]^+$ 398.2299; found 398.2305.

6-Methyl-4,4-diphenyl-1-(*p*-tolyl)-1,2,3,4-tetrahydroquinoline, 3ad and 6-methyl-4,4-diphenyl-1-(*p*-tolyl)-1,4-dihydroquinoline, 3ad'

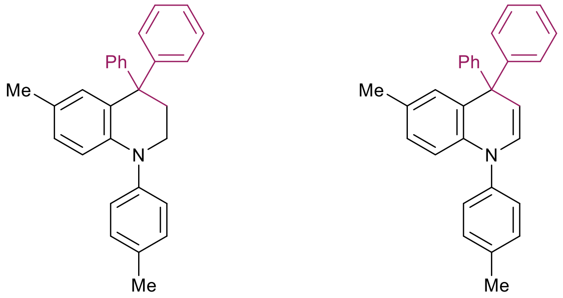

Following General Procedure 8, using *N*-methyl-di-*p*-tolylamine **1a** (42.3 mg, 0.200 mmol), 1,1-diphenylethylene **2d** (80 µL, 0.44 mmol) in DCE (0.5 mL) for 22 h at 85 °C, gave the compound **3ad** (73% spectroscopic yield). Purification via flash column chromatography on silica gel (eluent = 1% DCM/petroleum ether) gave the dihydroquinoline **3ad'** as a white solid (27.7 mg, 70.0 µmol, 36%).

Following General Procedure 8, using *N*-methyl-di-*p*-tolylamine **1a** (42.3 mg, 0.200 mmol), 1,1-diphenylethylene **2d** (80 µL, 0.44 mmol) in DCE (0.5 mL) for 22 h at 85 °C, gave the compound **3ad** (90% spectroscopic yield). The reaction crude was dissolved in HCl solution (2mL, 2 M in Et_2O) and stirred overnight. Amorphous white solid precipitated out and was filtrated, washed with more methanol to give the tetrahydroquinoline **3ad** as a white solid (32 mg, 80 µmol, 41%).

Following General Procedure 8, using *N*-methyl-di-*p*-tolylamine **1a** (42.3 mg, 0.200 mmol), 1,1-diphenylethylene **2d** (0.18 mL, 1.0 mmol) in DCE (0.5 mL) for 120 h at 85 °C, gave the compound **3ad** (94% spectroscopic yield).

6-Methyl-4,4-diphenyl-1-(*p*-tolyl)-1,2,3,4-tetrahydroquinoline, **3ad**: R_f = 0.1 (1% DCM/petroleum ether). ^1H NMR (500 MHz, $CDCl_3$) δ_H 7.33-7.30 (4H, m, ArH), 7.26-7.23 (2H, m, ArH), 7.17-7.16 (4H, m, ArH), 7.11-7.09 (2H, m, ArH), 7.06-7.04 (2H, m, ArH), 6.83 (1H, dd, *J* = 8.3 Hz, *J* = 1.8 Hz, ArH), 6.71 (1H, d, *J* = 8.3 Hz, ArH), 6.34 (1H, d, *J* = 1.8 Hz, ArH), 3.37 (2H, t, *J* = 6.1 Hz, NCH_2), 2.84 (2H, t, *J* = 6.1 Hz, CH_2), 2.32 (3H, s, CH_3), 2.11 (3H, s, CH_3); $^{13}C\{^1H\}$ NMR ($CDCl_3$, 126 MHz) δ_C 147.1 (C), 145.9 (C), 142.2 (C), 132.9 (C), 130.8 (C), 130.7 (CH), 129.9 (CH), 129.3 (CH), 127.9 (CH), 127.7 (CH), 126.5 (C), 126.2 (CH), 124.0 (CH), 116.5 (CH), 51.7 (C), 47.2 (NCH_2), 36.5 (CH_2), 20.8 (CH_3), 20.6 (CH_3). HRMS (ESI^+) m/z: calcd for $C_{29}H_{28}N$ $[M+H]^+$ 390.2216; found 390.2208.

6-Methyl-4,4-diphenyl-1-(*p*-tolyl)-1,4-dihydroquinoline, **3ad'**: Rf = 0.1 (1% DCM/petroleum ether). ^1H NMR (400 MHz, $CDCl_3$) δ_H 7.36-7.30 (7H, m, ArH), 7.29-7.21 (6H, m, ArH), 7.19-7.16 (2H, m, ArH),

6.80 (1H, dd, J = 8.5, 1.9 Hz, ArH), 6.70 (1H, d, J = 1.9 Hz, ArH), 6.55 (1H, d, J = 8.5 Hz, ArH), 6.47 (1H, d, J = 7.8 Hz, NCH), 5.08 (1H, d, J = 7.8 Hz, NCH), 2.41 (3H, s, CH_3), 2.15 (3H, s, CH_3); $^{13}C\{^1H\}$ NMR ($CDCl_3$, 101 MHz) δ_C 150.2 (C), 141.5 (C), 138.4 (C), 136.3 (C), 131.6 (CH), 130.3 (CH), 130.0 (C), 129.4 (NCH), 129.1 (CH), 127.8 (CH), 127.3 (CH), 127.2 (CH), 125.9 (C), 125.6 (CH), 120.3 (CH), 113.7 (CH), 107.2 (CH), 51.8 (C), 21.1 (CH_3), 20.7 (CH_3). HRMS (ESI$^+$) m/z: calcd for $C_{29}H_{26}N$ [M+H]$^+$ 388.2060; found 388.2075.

4-(4-Fluorophenyl)-6-methyl-4-phenyl-1-(*p*-tolyl)-1,2,3,4-tetrahydroquinoline, 3ae and 4-(4-fluorophenyl)-6-methyl-4-phenyl-1-(*p*-tolyl)-1,4-dihydroquinoline, 3ae'

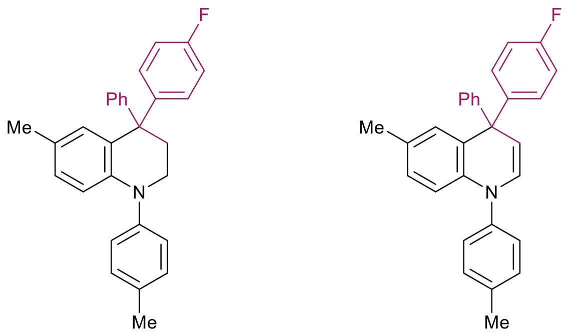

Following General Procedure 8, using *N*-methyl-di-*p*-tolylamine **1a** (84.6 mg, 0.400 mmol), 1-fluoro-4-(1-phenylvinyl)benzene **2e** (175 mg, 0.880 mmol) in DCE (1.0 mL) for 22 h at 85 °C, gave the compound **3ae** (58% spectroscopic yield). Purification via flash column chromatography on silica gel (eluent = 10% DCM/petroleum ether) gave the dihydroquinoline **3ae'** as a clear oil (60 mg, 0.15 mmol, 37%).

Following General Procedure 8, using *N*-methyl-di-*p*-tolylamine **1a** (84.6 mg, 0.400 mmol), 1-fluoro-4-(1-phenylvinyl)benzene **2e** (397 mg, 2.00 mmol) in *o*-DCB (1.0 mL) for 44 h at 120 °C, gave the compound **3ae** (95% spectroscopic yield). Purification by recrystallisation (petroleum ether) gave the tetrahydroquinoline **3ae** as a white solid (13 mg, 30 µmol, 8%).

4-(4-Fluorophenyl)-6-methyl-4-phenyl-1-(*p*-tolyl)-1,2,3,4-tetrahydroquinoline, **3ae**: R_f = 0.5 (20% DCM/petroleum ether). ^1H NMR (400 MHz, $CDCl_3$) δ_H 7.36-7.21 (3H, m, ArH), 7.14-7.09 (6H, m, ArH), 7.04-6.96 (4H, m, ArH), 6.85-6.80 (1H, m, ArH), 6.7 (1H, d, J = 8.3 Hz, ArH), 6.30 (1H, m, ArH), 3.40-3.30 (2H, m, NCH_2), 2.86-2.75 (2H, m, CH_2), 2.31 (3H, s, CH_3), 2.11 (3H, s, CH_3); $^{13}C\{^1H\}$ NMR ($CDCl_3$, 101 MHz) δ_C 162.5 (C), 160.1 (C), 146.8 (C), 145.7 (C), 142.7 (1C, d, J = 3.4 Hz, C), 142.1 (C), 133.0 (C), 130.8 (2C, d, J = 7.6 Hz, CH), 130.5 (CH), 129.9 (CH), 129.1 (CH), 128.0 (CH), 127.9 (CH), 126.6 (C), 126.4 (CH), 123.9 (CH), 116.6 (CH), 114.7 (2C, d, J = 21.0 Hz, CH), 51.2 (C), 47.1 (NCH_2), 36.7 (CH_2), 20.8 (CH_3), 20.6 (CH_3). $^{19}F\{^1H\}$ NMR (376 MHz, $CDCl_3$) δ_F -117.0. HRMS (ESI$^+$) m/z: calcd for $C_{29}H_{27}FN$ [M+H]$^+$ 408.2122; found 408.2126; calc. for $C_{29}H_{26}FN$ [M]$^+$ 407.2044; found 407.2046.

4-(4-Fluorophenyl)-6-methyl-4-phenyl-1-(*p*-tolyl)-1,4-dihydroquinoline, **3ae'**: R_f = 0.3 (10% DCM/petroleum ether). ^{1}H NMR (400 MHz, $CDCl_3$) δ_H 7.36-7.32 (2H, m, ArH), 7.26-7.21 (7H, m, ArH), 7.16 (2H, m, ArH), 7.01 (2H, m, ArH), 6.82 (1H, dd, *J* = 8.4, 1.7 Hz, ArH), 6.66 (1H, d, *J* = 1.7 Hz, ArH), 6.55 (1H, d, *J* = 8.4 Hz, ArH), 6.46 (1H, d, *J* = 7.7 Hz, NCH), 5.05 (1H, d, *J* = 7.7 Hz, CH), 2.41 (3H, s, CH_3), 2.16 (3H, s, CH_3); ^{13}C{^{1}H} NMR ($CDCl_3$, 101 MHz) δ_C 162.2 (C), 159.7 (C), 150.0 (C), 146.0 (1C, d, *J* = 3.43 Hz, C), 141.4 (C), 138.3 (C), 136.4 (C), 131.4 (CH), 130.6 (2C, d, *J* = 7.8 Hz, CH), 130.4 (CH), 130.1 (CH), 129.6 (NCH), 129.0 (CH), 127.9 (CH), 127.4 (CH), 127.3 (CH), 125.8 (C), 114.4 (2C, d, *J* = 21.0 Hz, CH), 113.8 (CH), 107.0 (CH), 51.3 (C), 21.0 (CH_3), 20.7 (CH_3). ^{19}F{^{1}H} NMR (376 MHz, $CDCl_3$) δ_F -117.8. HRMS (ESI$^+$) m/z: calcd for $C_{29}H_{25}FN$ [M+H]$^+$ 406.1966; found 406.1971.

4-(4-Bromophenyl)-6-methyl-4-phenyl-1-(*p*-tolyl)-1,2,3,4-tetrahydroquinoline, 3af and 4-(4-bromophenyl)-6-methyl-4-phenyl-1-(*p*-tolyl)-1,4-dihydroquinoline, 3af'

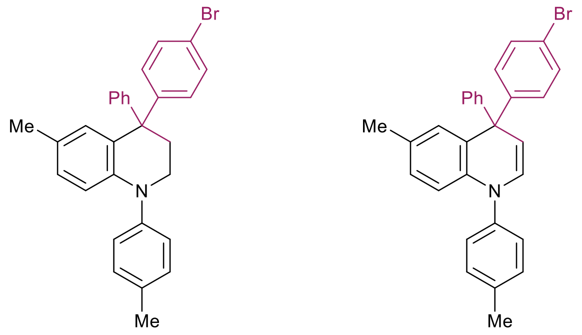

Following General Procedure 8, using *N*-methyl-di-*p*-tolylamine **1a** (42.3 mg, 0.200 mmol), 1-bromo-4-(1-phenylvinyl)benzene **2f** (259 mg, 1.00 mmol) in *o*-DCB (0.5 mL) for 44 h at 120 °C, gave the compound **3af** (97% spectroscopic yield). Purification via recrystallisation (methanol) gave the tetrahydroquinoline **3af** as a white solid (36 mg, 80 µmol, 41%).

Following General Procedure 8, using *N*-methyl-di-*p*-tolylamine **1a** (42.3 mg, 0.200 mmol), 1-bromo-4-(1-phenylvinyl)benzene **2f** (259 mg, 1.00 mmol) in DCE (0.5 mL) for 120 h at 85 °C, gave the compound **3af** (92% spectroscopic yield). Purification via flash column chromatography on silica gel (eluent = 1% EtOAc/petroleum ether) gave the dihydroquinoline **3af'** as a white solid (40 mg, 86 µmol, 43%).

4-(4-Bromophenyl)-6-methyl-4-phenyl-1-(*p*-tolyl)-1,4-tetrahydroquinoline, **3af**: R_f = 0.6 (2% EtOAc/petroleum ether). ^{1}H NMR (400 MHz, $CDCl_3$) δ_H 7.44-7.39 (2H, m, ArH), 7.34-7.28 (2H, m, ArH), 7.27-7.22 (1H, m, ArH), 7.15-7.07 (4H, m, ArH), 7.06-7.00 (4H, m, ArH), 6.83 (1H, dd, *J* = 8.3, 1.7 Hz, ArH), 6.69 (1H, d, *J* = 8.3 Hz, ArH), 6.30 (1H, d, *J* = 1.7 Hz, ArH), 3.40-3.30 (2H, m, NCH_2), 2.87-2.72 (2H, m, CH_2), 2.32 (3H, s, CH_3), 2.11 (3H, s, CH_3); ^{13}C{^{1}H} NMR ($CDCl_3$, 101 MHz) δ_C 146.5 (C), 146.3 (C), 145.7 (C), 142.1 (C), 133.1 (C), 131.1 (CH), 131.0 (CH), 130.5 (CH), 130.1 (C), 130.0 (CH), 129.1 (CH), 128.1 (CH), 128.0 (CH), 126.6 (C), 126.5 (CH), 124.0 (CH), 120.3 (C), 116.6

(CH), 51.4 (C), 47.0 (NCH_2), 36.5 (CH_2), 20.8 (CH_3), 20.6 (CH_3). HRMS (ESI$^+$) m/z: calcd for $C_{29}H_{26}{}^{79}BrN$ $[M]^+$ 467.1243; found 467.1249.

4-(4-Bromophenyl)-6-methyl-4-phenyl-1-(*p*-tolyl)-1,4-dihydroquinoline, **3af'**: R_f = 0.6 (2% EtOAc/petroleum ether). ^1H NMR (400 MHz, $CDCl_3$) δ_H 7.46-7.42 (2H, m, ArH), 7.36-7.31 (2H, m, ArH), 7.27-7.22 (5H, m, ArH), 7.18-7.14 (4H, m, ArH), 6.82 (1H, ddd, *J* = 8.4, 2.1, 0.6 Hz, ArH), 6.65-6.64 (1H, m, ArH), 6.54 (1H, d, *J* = 8.4 Hz, ArH), 6.46 (1H, d, *J* = 7.8 Hz, NCH), 5.01 (1H, d, *J* = 7.8 Hz, CH), 2.41 (3H, s, CH_3), 2.15 (3H, s, CH_3); $^{13}C\{^1H\}$ NMR ($CDCl_3$, 101 MHz) δ_C 149.6 (C), 149.3 (C), 141.3 (C), 138.3 (C), 136.4 (C), 131.4 (CH), 130.9 (CH), 130.8 (CH), 130.4 (CH), 130.2 (C), 129.8 (NCH), 129.0 (CH), 127.9 (CH), 127.4 (CH), 127.3 (CH), 125.8 (CH), 125.3 (C), 119.7 (C), 113.8 (CH), 106.6 (CH), 51.5 (C), 21.1 (CH_3), 20.7 (CH_3). HRMS (ASAP$^+$) m/z: calcd for $C_{29}H_{25}{}^{79}BrN$ $[M+H]^+$ 466.1165; found 466.1171; calc. for $C_{29}H_{25}{}^{81}BrN$ $[M+H]^+$ 468.1144; found 468.1150.

4-(4-Methoxyphenyl)-6-methyl-4-phenyl-1-(*p*-tolyl)-1,2,3,4-tetrahydroquinoline, 3ag and 4-(4-methoxyphenyl)-6-methyl-4-phenyl-1-(*p*-tolyl)-1,4-dihydroquinoline, 3ag'

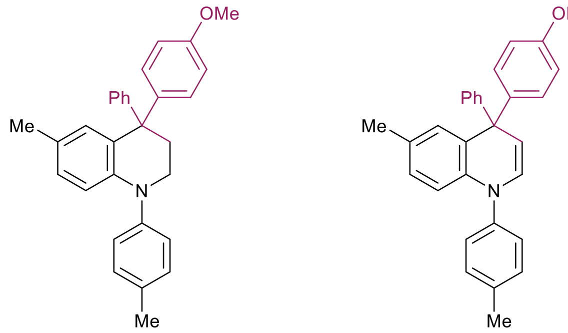

Following General Procedure 8, using *N*-methyl-di-*p*-tolylamine **1a** (42.3 mg, 0.200 mmol), 1-methoxy-4-(1-phenylvinyl)benzene **2g** (92.5 mg, 0.440 mmol) in DCE (0.5 mL) for 22 h at 85 °C, gave the title compound **3ag** (79% spectroscopic yield). Purification via flash column chromatography on silica gel (eluent = 1% EtOAc/petroleum ether) gave the dihydroquinoline **3ag'** as a white solid (4.6 mg, 10 µmol, 5%).

Following General Procedure 8, using *N*-methyl-di-*p*-tolylamine **1a** (84.6 mg, 0.400 mmol), 1-methoxy-4-(1-phenylvinyl)benzene **2g** (185 mg, 0.880 mmol) in DCE (0.5 mL) for 22 h at 85 °C, gave the title compound **3ag** (20% spectroscopic yield). Purification by flash column chromatography on silica gel (through a short silica plug) (eluent = 20% DCM/petroleum ether) gave the tetrahydroquinoline **3ag** as a white solid (24 mg, 57 µmol, 14%).

4-(4-Methoxyphenyl)-6-methyl-4-phenyl-1-(*p*-tolyl)-1,2,3,4-tetrahydroquinoline, **3ag**: R_f = 0.2 (20% DCM/petroleum ether). ^1H NMR (500 MHz, $CDCl_3$) δ_H 7.32-7.29 (2H, m, ArH), 7.25-7.21 (1H, m, ArH), 7.16-7.14 (2H, m, ArH), 7.11-7.04 (6H, m, ArH), 6.86-6.84 (2H, m, ArH), 6.83-6.81 (1H, m, ArH), 6.70 (1H, d, *J* = 8.3 Hz, ArH), 6.34 (1H, d, J = 1.9 Hz, ArH), 3.82 (3H, s, OCH_3), 3.41-3.32 (2H, m, NCH_2), 2.84-2.76 (2H, m, CH_2), 2.32 (3H, s, CH_3), 2.11 (3H, s, CH_3); $^{13}C\{^1H\}$ NMR ($CDCl_3$, 126

MHz) δ_C 157.8 (C), 147.3 (C), 145.9 (C), 142.1 (C), 139.1 (C), 132.9 (C), 131.1 (C), 130.6 (CH), 130.2 (CH), 129.9 (CH), 129.2 (CH), 127.3 (CH), 127.7 (CH), 126.5 (C), 126.2 (CH), 124.0 (CH), 116.5 (CH), 113.2 (CH), 55.2 (OCH_3), 51.0 (C), 47.2 (NCH_2), 36.6 (CH_2), 20.8 (CH_3), 20.6 (CH_3). HRMS (ESI$^+$) m/z: calcd for $C_{30}H_{30}NO$ $[M+H]^+$ 420.2322; found 420.2327; calcd for $C_{30}H_{29}NO$ $[M]^+$ 419.2244; found 419.2246.

4-(4-Methoxyphenyl)-6-methyl-4-phenyl-1-(*p*-tolyl)-1,4-dihydroquinoline, **3ag'**: R_f = 0.3 (1% EtOAc/petroleum ether). ^{1}H NMR (400 MHz, $CDCl_3$) δ_H 7.32-7.29 (2H, m, ArH), 7.26-7.20 (5H, m, ArH), 7.19-7.16 (2H, m, ArH), 7.16-7.13 (2H, m, ArH), 6.87-6.84 (2H, m, ArH), 6.78 (1H, ddd, *J* = 8.3, 2.6, 0.7 Hz ArH), 6.65 (1H, m, ArH), 6.50 (1H, d, *J* = 8.3 Hz, ArH), 6.42 (1H, d, *J* = 7.7 Hz, NCH), 5.03 (1H, d, *J* = 7.7 Hz, CH), 3.82 (3H, s, OCH_3), 2.39 (3H, s, CH_3), 2.13 (3H, s, CH_3); $^{13}C\{^1H\}$ NMR ($CDCl_3$, 101 MHz) δ_C 157.5 (C), 150.5 (C), 142.7 (C), 141.6 (C), 138.4 (C), 136.2 (C), 131.6 (CH), 130.3 (CH), 130.2 (CH), 130.0 (C), 129.3 (CH), 129.1 (CH), 127.8 (CH), 127.3 (CH), 127.2 (CH), 126.2 (C), 125.6 (CH), 113.7 (NCH), 113.1 (CH), 107.4 (CH), 55.2 (OCH_3), 51.1 (C), 21.1 (CH_3), 20.7 (CH_3). HRMS (ESI$^+$) m/z: calcd for $C_{30}H_{28}NO$ $[M+H]^+$ 418.2165; found 418.2165.

6-Methyl-4-phenyl-4-(4-(4,4,5,5-tetramethyl-1,3,2-dioxaborolan-2-yl)phenyl)-1-(*p*-tolyl)-1,2,3,4-tetrahydroquinoline, 3ah

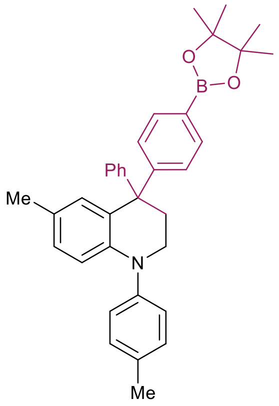

Following General Procedure 8, using *N*-methyl-di-*p*-tolylamine **1a** (42.3 mg, 0.200 mmol), 4,4,5,5-tetramethyl-2-(4-(1-phenylvinyl)phenyl)-1,3,2-dioxaborolane **2h** (306 mg, 1.00 mmol) in DCE (0.5 mL) for 120 h at 85 °C, gave the title compound **3ah** (67% spectroscopic yield). Purification via flash column chromatography on silica gel impregnated with boric acid[37] (eluent = 1% EtOAc/petroleum ether) gave the title compound **3ah** as a colourless oil (58 mg, 0.11 mmol, 56%).

R_f = 0.5 (4% EtOAc/petroleum ether). ^{1}H NMR (400 MHz, $CDCl_3$) δ_H 7.74-7.72 (2H, m, ArH), 7.30-7.25 (2H, m, ArH), 7.24-7.19 (1H, m, ArH), 7.17-7.07 (6H, m, ArH), 7.04-7.01 (2H, m, ArH), 6.81 (1H, ddd, *J* = 8.3, 2.2, 0.6 Hz, ArH), 6.68 (1H, d, *J* = 8.3 Hz, ArH), 6.31 (1H, d, *J* = 2.0 Hz, ArH), 3.33 (2H, t, *J* = 6.1 Hz, NCH_2), 2.87-2.76 (2H, m, CH_2), 2.30 (3H, s, CH_3), 2.08 (3H, s, CH_3), 1.36 (6H, s, CH_3), 1.35 (6H, s, CH_3); $^{13}C\{^1H\}$ NMR ($CDCl_3$, 101 MHz) δ_C 150.4 (C), 146.9 (C), 145.8 (C), 142.2 (C), 134.4 (CH), 132.9 (C), 130.7 (CH), 130.5 (C), 129.9 (CH), 129.3 (CH), 128.7 (CH), 127.9 (CH), 127.8

(CH), 126.5 (C), 126.3 (CH), 124.1 (CH), 116.4 (CH), 83.7 (C), 51.9 (C), 47.1 (NCH_2), 36.5 (CH_2), 25.0 (CH_3), 24.8 (CH_3), 20.8 (CH_3), 20.6 (CH_3); ^{11}B NMR (376 MHz, $CDCl_3$) δ_B 30.7. HRMS (ESI$^+$) m/z: calcd for $C_{35}H_{39}{}^{11}BNO_2$ $[M+H]^+$ 516.3068; found 516.3074.

4-(3-Bromophenyl)-6-methyl-4-phenyl-1-(*p*-tolyl)-1,2,3,4-tetrahydroquinoline, 3ai and 4-(3-bromophenyl)-6-methyl-4-phenyl-1-(*p*-tolyl)-1,4-dihydroquinoline, 3ai'

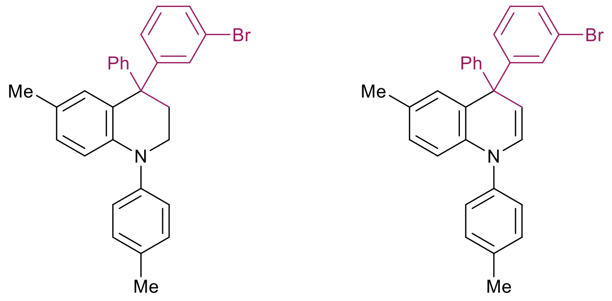

Following General Procedure 8, using *N*-methyl-di-*p*-tolylamine **1a** (42.3 mg, 0.200 mmol), 1-bromo-3-(1-phenylvinyl)benzene **2i** (259.2 mg, 1.00 mmol) in DCE (0.5 mL) for 120 h at 85 °C, gave the compound **3ai** (95% spectroscopic yield). Purification via flash column chromatography on silica gel (eluent = 20% DCM/petroleum ether) gave the dihydroquinoline **3ai'** as a white solid (67 mg, 0.14 mmol, 72%).

Following General Procedure 8, using *N*-methyl-di-*p*-tolylamine **1a** (42.3 mg, 0.200 mmol), 1-bromo-3-(1-phenylvinyl)benzene **2i** (259.2 mg, 1.00 mmol) in *o*-DCB (0.5 mL) for 44 h at 120 °C, gave the compound **3ai** (92% spectroscopic yield). The reaction crude was dissolved in HCl solution (2 mL, 2 M in Et_2O) and stirred overnight. Amorphous white solid precipitated out and was filtrated, washed with more methanol to give the tetrahydroquinoline **3ai** as a white solid (20 mg, 43 µmol, 21%).

4-(3-Bromophenyl)-6-methyl-4-phenyl-1-(*p*-tolyl)-1,2,3,4-tetrahydroquinoline, **3ai**: Rf = 0.4 (20% DCM/petroleum ether). ^{1}H NMR (400 MHz, $CDCl_3$) δ_H 7.38-7.30 (4H, m, ArH), 7.28-7.24 (1H, m, ArH), 7.18-7.02 (8H, m, ArH), 6.84 (1H, dd, *J* = 8.3, 1.6 Hz, ArH), 6.71 (1H, d, *J* = 8.3 Hz, ArH), 6.30 (1H, d, *J* = 1.6 Hz, ArH), 3.42-3.28 (2H, m, NCH_2), 2.86-2.75 (2H, m, CH_2), 2.32 (3H, s, CH_3), 2.11 (3H, s, CH_3); ^{13}C{^{1}H} NMR ($CDCl_3$, 101 MHz) δ_C 149.6 (C), 146.2 (C), 145.6 (C), 142.0 (C), 133.0 (C), 132.1 (CH), 130.4 (CH), 130.2 (C), 129.9 (CH), 129.5 (CH), 129.4 (CH), 129.1 (CH), 128.2 (CH), 128.1 (CH), 128.0 (CH), 126.8 (C), 126.5 (CH), 123.9 (CH), 122.3 (C), 116.8 (CH), 106.5 (CH), 51.6 (C), 47.0 (NCH_2), 36.6 (CH_2), 20.8 (CH_3), 20.6 (CH_3). HRMS (ESI+) m/z: calcd for $C_{29}H_{26}{}^{79}BrN$ $[M]^+$ 467.1243; found 467.1247.

4-(3-Bromophenyl)-6-methyl-4-phenyl-1-(*p*-tolyl)-1,4-dihydroquinoline, **3ai'**: Rf = 0.4 (20% DCM/petroleum ether). ^{1}H NMR (400 MHz, $CDCl_3$) δ_H 7.49-7.48 (1H, m, ArH), 7.38-7.33 (3H, m, ArH), 7.27-7.23 (5H, m, ArH), 7.19-7.16 (4H, m, ArH), 6.83 (1H, dd, *J* = 8.4, 1.7 Hz, ArH), 6.66 (1H, d, *J* = 1.7 Hz, ArH), 6.55 (1H, d, *J* = 8.4 Hz, ArH), 6.47 (1H, d, *J* = 7.8 Hz, NCH), 5.02 (1H, d, *J* = 7.8

Hz, CH), 2.42 (3H, s, CH_3), 2.16 (3H, s, CH_3); $^{13}C\{^1H\}$ NMR ($CDCl_3$, 101 MHz) δ_C 152.6 (C), 149.5 (C), 141.3 (C), 138.3 (C), 136.4 (C), 132.2 (CH), 131.4 (CH), 130.4 (CH), 130.3 (CH), 129.9 (NCH), 129.2 (CH), 129.0 (C), 128.8 (CH), 127.9 (CH), 127.8 (CH), 127.5 (CH), 127.3 (CH), 125.9 (CH), 125.1 (C), 122.2 (C), 113.9 (CH), 106.5 (CH), 51.8 (C), 21.1 (CH_3), 20.7 (CH_3). HRMS (ESI+) m/z: calcd for $C_{29}H_{25}{}^{79}BrN$ $[M+H]^+$ 466.1165; found 466.1168.

4-(4-Methoxyphenyl)-4,6-dimethyl-1-(*p*-tolyl)-1,2,3,4-tetrahydroquinoline, 3aj

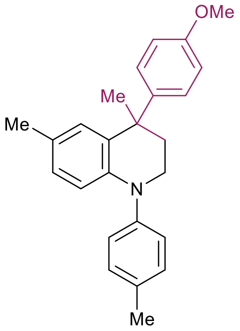

Following General Procedure 8, using *N*-methyl-di-*p*-tolylamine **1a** (42.3 mg, 0.200 mmol), 1-Methoxy-4-(prop-1-en-2-yl)benzene **2j** (148 mg, 1.00 mmol) in DCE (0.5 mL) for 120 h at 85 °C, gave the compound **3aj** (52% spectroscopic yield). Purification via flash column chromatography on silica gel (eluent = 30% DCM/petroleum ether) gave the title compound **3aj** as a clear oil (30 mg, 84 µmol, 42%).

R_f = 0.4 (40% DCM/petroleum ether). ^1H NMR (500 MHz, $CDCl_3$) δ_H 7.15-7.07 (6H, m, ArH), 6.88 (1H, d, *J* = 1.7 Hz, ArH), 6.83-6.79 (3H, m, ArH), 6.67 (1H, d, *J* = 8.4 Hz, ArH), 3.79 (3H, s, OCH_3), 3.56-3.51 (1H, m, NCH_2), 3.27-3.22 (1H, m, NCH_2), 2.33 (3H, s, CH_3), 2.24-2.19 (1H, m, CH_2), 2.21 (3H, s, CH_3), 2.11-2.05 (1H, m, CH_2), 1.76 (3H, s, CH_3); $^{13}C\{^1H\}$ NMR ($CDCl_3$, 126 MHz) δ_C 157.5 (C), 146.4 (C), 142.2 (C), 142.1 (C), 133.1 (C), 131.4 (C), 129.9 (CH), 128.8 (CH), 128.3 (CH), 127.3 (CH), 127.1 (C), 124.6 (CH), 116.4 (CH), 113.3 (CH), 55.2 (OCH_3), 47.5 (NCH_2), 40.5 (C), 39.2 (CH_2), 29.7 (CH_3), 20.8 (CH_3), 20.6 (CH_3). HRMS (ESI^+) m/z: calcd for $C_{25}H_{27}NO$ $[M]^+$ 357.2087; found 357.2093.

6-Methyl-1-(*p*-tolyl)-2,3-dihydro-1*H*-spiro[quinoline-4,9'-thioxanthene], 3ak

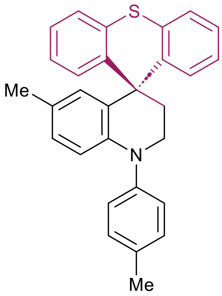

Following General Procedure 8, using *N*-methyl-di-*p*-tolylamine **1a** (42.4 mg, 0.200 mmol), 9-methylene-9H-thioxanthene **2k** (92.0 mg, 0.440 mmol) in DCE (0.5 mL) for 22 h at 85 °C, gave the title

compound **3ak** (60% spectroscopic yield). Purification via flash column chromatography on silica gel (eluent = 3% DCM/petroleum ether) gave the title compound **3ak** as a white solid (36 mg, 86 μmol, 43%).

R_f = 0.3 (3% DCM/petroleum ether). ^{1}H NMR (400 MHz, $CDCl_3$) δ_H 7.41 (2H, dd, *J* = 7.7, 1.4 Hz), 7.20 (2H, ddd, *J* = 7.7, 7.3, 1.4 Hz), 7.17-7.12 (6H, m), 7.05 (2H, dd, *J* = 7.9, 1.4 Hz), 6.93 (1H, dd, *J* = 8.5, 1.8 Hz), 6.86 (1H, d, *J* = 8.5 Hz), 6.78 (1H, d, *J* = 1.8 Hz), 3.33-3.31 (2H, m), 2.35 (3H, s), 2.27-2.25 (2H, m), 2.17 (3H, s). ^{13}C{^{1}H} NMR ($CDCl_3$, 101 MHz) δ_C 146.4, 144.9, 142.5, 133.2, 131.1, 130.3, 130.0, 128.3, 127.9, 126.8, 126.4, 125.9, 125.8, 124.7, 120.3, 117.3, 49.4, 47.1, 32.8, 20.8, 20.5. HRMS (ESI$^+$) m/z: calcd for $C_{29}H_{26}NS$ [M+H]$^+$ 420.1780; found 420.1782.

6-Methyl-1-(*p*-tolyl)-2,3-dihydro-1*H*-spiro[quinoline-4,9'-xanthene], 3al

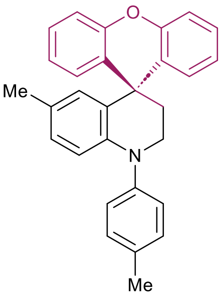

Following General Procedure 8, using *N*-methyl-di-*p*-tolylamine **1a** (42.2 mg, 0.200 mmol), 9-methylene-9H-xanthene **2l** (85.4 mg, 0.440 mmol) in DCE (0.5 mL) for 22 h at 85 °C, gave the title compound **3al** (70% spectroscopic yield). Purification via flash column chromatography on silica gel (eluent = 10% DCM/petroleum ether) gave the title compound **3al** as a white solid (36 mg, 88 μmol, 44%).

R_f = 0.3 (10% DCM/petroleum ether). ^{1}H NMR (400 MHz, $CDCl_3$) δ_H 7.26-7.23 (2H, m, ArH), 7.20-7.16 (6H, m, ArH), 7.04 (4H, m, ArH), 6.85 (1H, dd, *J* = 8.4, 1.7 Hz, ArH), 6.77 (1H, d, *J* = 8.4 Hz, ArH), 6.75 (1H, d, *J* = 1.7 Hz, ArH), 3.50 (2H, m, NCH_2), 2.37 (3H, s, CH_3), 2.13 (2H, m, CH_2), 2.10 (3H, s, CH_3). ^{13}C{^{1}H} NMR ($CDCl_3$, 101 MHz) δ_C 151.4 (C), 146.4 (C), 144.5 (C), 133.6 (C), 132.4 (CH), 131.5 (C), 130.1 (CH), 129.4 (CH), 128.2 (CH), 127.9 (C), 127.4 (CH), 127.1 (C), 125.1 (CH), 122.9 (CH), 116.3 (CH), 116.1 (CH), 46.8 (NCH_2), 42.5 (C), 40.9 (CH_2), 20.9 (CH_3), 20.4 (CH_3). HRMS (ESI$^+$) m/z: calcd for $C_{29}H_{26}NO$ [M+H]$^+$ 404.2009; found 404.2010; calcd for $C_{29}H_{25}NO$ [M]$^+$ 403.1931; found 403.1938.

6'-Methyl-1'-(*p*-tolyl)-2',3,3',4-tetrahydro-1'*H*,2*H*-spiro[naphthalene-1,4'-quinoline], 3am and 6'-methyl-1'-(*p*-tolyl)-3,4-dihydro-1'*H*,2*H*-spiro[naphthalene-1,4'-quinoline], 3am'

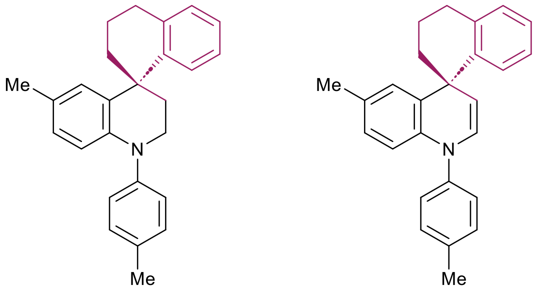

Following General Procedure 8, using *N*-methyl-di-*p*-tolylamine **1a** (42.3 mg, 0.200 mmol), 1-methylene-1,2,3,4-tetrahydronaphthalene **2m** (144.2 mg, 1.00 mmol) in DCE (0.5 mL) for 120 h at 85 °C, gave the compound **3am** (60% spectroscopic yield). Purification via flash column chromatography on silica gel (eluent = 10% DCM/petroleum ether) gave the dihydroquinoline **3am'** as a clear oil (11 mg, 31 µmol, 16%).

Following General Procedure 8, using *N*-methyl-di-*p*-tolylamine **1a** (42.3 mg, 0.200 mmol), 1-methylene-1,2,3,4-tetrahydronaphthalene **2m** (144 mg, 1.00 mmol) in DCE (0.5 mL) for 120 h at 85 °C, gave the compound **3am** (40% spectroscopic yield). Purification via flash column chromatography on silica gel (eluent = 20% DCM/petroleum ether) gave the title tetrahydroquinoline **3am** as a clear oil (17 mg, 48 µmol, 24%).

6'-Methyl-1'-(*p*-tolyl)-2',3,3',4-tetrahydro-1'*H*,2*H*-spiro[naphthalene-1,4'-quinoline], **3am**: Rf = 0.4 (20% DCM/petroleum ether). ^{1}H NMR (400 MHz, $CDCl_3$) δ_H 7.18-7.03 (8H, m, ArH), 6.75-6.73 (1H, m, ArH), 6.68-6.66 (1H, m, ArH), 6.52-6.49 (1H, m, ArH), 3.71-3.63 (2H, m, NCH_2), 3.02-2.94 (1H, m, CH_2), 2.91-2.84 (1H, m, CH_2), 2.36 (3H, m, CH_3), 2.35-2.28 (1H, m, CH_2), 2.12-2.06 (3H, m, CH_2), 2.10 (3H, s, CH_3), 1.95-1.83 (2H, m, CH_2); ^{13}C{^{1}H} NMR ($CDCl_3$, 101 MHz) δ_C 146.4 (C), 144.9 (C), 142.1 (C), 137.5 (C), 134.0 (C), 133.0 (C), 130.4 (CH), 130.0 (CH), 129.8 (CH), 129.8 (CH), 128.7 (CH), 127.2 (C), 127.0 (CH), 125.9 (CH), 125.6 (CH), 124.4 (CH), 116.3 (CH), 47.3 (NCH_2), 41.0 (C), 38.3 (CH_2), 37.6 (CH_2), 30.4 (CH_2), 20.9 (CH_3), 20.5 (CH_3), 19.0 (CH_2). HRMS (ESI$^+$) m/z: calcd for $C_{26}H_{28}N$ [M+H]$^+$ 354.2216; found 354.2222; calc. for $C_{26}H_{27}N$ [M]$^+$ 353.2138; found 353.2144.

6'-Methyl-1'-(*p*-tolyl)-3,4-dihydro-1'*H*,2*H*-spiro[naphthalene-1,4'-quinoline], **3am'**: Rf = 0.3 (10% DCM/petroleum ether). ^{1}H NMR (500 MHz, $CDCl_3$) δ_H 7.34-7.21 (2H, m), 7.18-7.09 (7H, m), 6.74 (1H, ddd, J = 8.4, 2.0, 0.3 Hz), 6.50 (1H, d, J = 2.0 Hz), 6.48 (1H, d, J = 8.4 Hz), 6.31 (1H, d, J = 7.8), 4.77 (1H, d, J = 7.8 Hz), 2.98-2.92 (1H, m), 2.91-2.84 (1H, m), 2.42 (3H, s), 2.12 (3H, s), 2.09 (2H, t, J = 6.1 Hz), 1.92-1.82 (2H, m); ^{13}C{^{1}H} NMR ($CDCl_3$, 126 MHz) δ_C 145.9, 141.9, 138.1, 136.5, 136.1, 136.5, 136.1, 132.2, 130.6, 129.8, 128.3, 128.1, 127.3, 126.7, 126.1, 125.5, 113.3, 108.1, 41.9, 41.9, 30.0, 21.1, 20.6, 18.26. HRMS (ESI$^+$) m/z: calcd for $C_{26}H_{26}N$ [M+H]$^+$ 352.2060; found 352.2048.

2,11b-Dimethyl-5-(*p*-tolyl)-6,6a,7,11b-tetrahydro-5*H*-indeno[2,1-c]quinoline, 3an

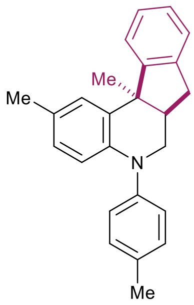

Following General Procedure 8, using *N*-methyl-di-*p*-tolylamine **1a** (42.3 mg, 0.200 mmol), 1-Methylene-2,3-dihydro-1H-indene **2n** (130 mg, 1.00 mmol) in DCE (0.5 mL) for 120 h at 85 °C, gave the compound **3an** (70% spectroscopic yield). Purification via flash column chromatography on silica gel (eluent = 12% DCM/petroleum ether) gave the title compound **3an** as a clear oil (22 mg, 65 µmol, 32%).

R_f = 0.21 (10% DCM/petroleum ether) ^{1}H NMR (500 MHz, $CDCl_3$) δ_H 7.55 (1H, d, J = 7.6 Hz, ArH), 7.26-7.21 (2H, m, ArH), 7.17-7.11 (6H, m, ArH), 6.71-6.69 (1H, m, ArH), 6.65 (1H, d, J = 8.4 Hz, ArH), 3.84 (1H, dd, *J* = 12.3 Hz, 4.0 Hz, NCH_2), 3.51 (1H, dd, *J* = 12.3 Hz, 4.0 Hz, NCH_2), 3.11-3.06 (1H, m, CH_2), 2.97-2.93 (1H, m, CH_2), 2.66 (1H, m, CH), 2.36 (3H, s, CH_3), 2.24 (3H, s, CH_3), 1.74 (3H, s, CH_3); $^{13}C\{^1H\}$ NMR ($CDCl_3$, 126 MHz) δ_C 150.7 (C), 146.0 (C), 141.7 (C), 141.0 (C), 133.0 (C), 130.0 (CH), 129.5 (CH), 128.0 (C), 127.1 (CH), 126.7 (CH), 126.5 (CH), 124.8 (CH), 124.2 (CH), 123.8 (CH), 115.9 (CH), 49.7 (NCH_2), 48.0 (CH), 47.8 (C), 34.0 (CH_2), 30.2 (CH_3), 20.9 (CH_3), 20.7 (CH_3). HRMS (ESI^+) m/z: calcd for $C_{25}H_{26}N$ $[M+H]^+$ 340.2060; found 340.2066.

3.5 Drug Derivatives in $B(C_6F_5)_3$-Catalyzed THQ synthesis

1-Benzhydryl-6-(4-chloro-3,5-dimethylphenoxy)-4-methyl-4-((trimethylsilyl)methyl)-1,2,3,4-tetrahydroquinoline, 3ao

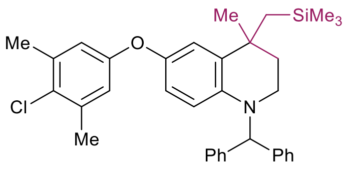

Following General Procedure 8, using *N*-benzhydryl-4-(4-chloro-3,5-dimethylphenoxy)-*N*-methylaniline **1ab** (85.6 mg, 0.200 mmol), methylallyltrimethylsilane **2a** (0.18 mL, 1.0 mmol) in DCE (0.50 mL) for 120 h at 85 °C, gave the compound **3ao** (74% spectroscopic yield). Purification via flash column chromatography on silica gel (eluent = 13% DCM/petroleum ether) gave the title compound **3ao** as a colourless oil (51 mg, 90 µmol, 46%).

R_f = 0.4 (20% DCM/petroleum ether). ^{1}H NMR (500 MHz, $CDCl_3$) δ_H 7.39-7.35 (4H, m, ArH), 7.34-7.30 (2H, m, ArH), 7.28-7.22 (4H, m, ArH), 6.96 (1H, d, *J* = 2.8 Hz, ArH), 6.71 (2H, s, ArH), 6.65 (1H, dd, *J* = 9.0 Hz, 2.8 Hz, ArH), 6.50 (1H, d, *J* = 9.0 Hz, CH), 6.15 (1H, s, NCH), 3.09 (1H, ddd, *J* = 12.0,

7.1, 4.7 Hz, NCH_2), 2.98 (1H, ddd, *J* = 12.0, 8.4, 5.0 Hz, NCH_2), 2.34 (6H, s, CH_3), 1.80 (1H, ddd, *J* = 12.7, 7.1, 4.7 Hz, CH_2), 1.70 (1H, ddd, *J* = 12.7, 8.4, 5.0 Hz, CH_2), 1.33 (3H, s, CH_3), 1.05 (2H, s, $SiCH_2$), -0.06 (9H, s, $SiCH_3$); $^{13}C\{^1H\}$ NMR ($CDCl_3$, 126 MHz) δ_C 156.9 (C), 146.1 (C), 141.1 (C), 140.8 (C), 140.1 (C), 137.1 (C), 134.4 (C), 129.2 (CH), 128.8 (CH), 128.4 (CH), 128.3 (CH), 127.3 (CH), 127.2 (C), 127.1 (CH), 118.2 (CH), 117.7 (CH), 117.0 (CH), 112.2 (CH), 66.3 (NCH), 41.3 (NCH_2), 37.0 (CH_2), 35.2 (C), 31.2 (CH_3), 31.0 (CH_2), 20.8 (CH_3), 0.7 ($SiCH_3$). HRMS (ESI^+) m/z: calcd for $C_{35}H_{41}{}^{35}ClNOSi$ $[M+H]^+$ 554.2460; found 554.2477.

1-((4*S*)-4-(3,4-Dichlorophenyl)-1,2,3,4-tetrahydronaphthalen-1-yl)-4,6-dimethyl-4-((trimethylsilyl)methyl)-1,2,3,4-tetrahydroquinoline, 3ap

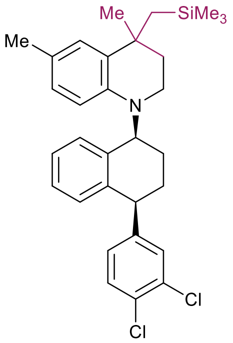

Following General Procedure 8, using (1*S*,4*S*)-4-(3,4-dichlorophenyl)-*N*-methyl-*N*-(*p*-tolyl)-1,2,3,4-tetrahydronaphthalen-1-amine **1ac** (79.3 mg, 0.200 mmol), methylallyltrimethylsilane **2a** (0.18 mL, 0.44 mmol) in DCE (0.50 mL) for 120 h at 85 °C, gave the compound **3ap** (66% spectroscopic yield, *dr* = 1:1).

Following General Procedure 8, using (1*S*,4*S*)-4-(3,4-dichlorophenyl)-*N*-methyl-*N*-(*p*-tolyl)-1,2,3,4-tetrahydronaphthalen-1-amine **1ac** (79.3 mg, 0.200 mmol), methylallyltrimethylsilane **2a** (0.18 mL, 1.0 mmol) in DCE (0.50 mL) for 44 h at 120 °C, gave the compound **3ap** (50% spectroscopic yield, 1:1 *dr*). Purification via flash column chromatography on silica gel (eluent = 8% DCM/petroleum ether) gave the title compound **3ap** as a clear oil (8.0 mg, 15 µmol, 8%, 6:1 *dr*).

One diastereoisomer: R_f = 0.4 (20% DCM/petroleum ether). ^1H NMR (400 MHz, $CDCl_3$) δ_H 7.48 (1H, d, *J* = 7.70 Hz, ArH), 7.37 (1H, d, *J* = 8.34 Hz, ArH), 7.30-7.24 (1H, m, ArH), 7.23-7.16 (2H, m, ArH), 7.07 (1H, m, ArH), 6.97 (1H, d, *J* = 7.34 Hz, ArH), 6.89 (2H, br dd, *J* = 8.30, 1.79 Hz, ArH), 6.66 (1H, br s, ArH), 5.27-5.01 (1H, m, CH), 4.20 (1H, t, *J* = 4.43 Hz, CH), 3.12-2.71 (2H, m, CH_2), 2.33-2.23 (1H, m, CH_2), 2.27 (3H, s, CH_3), 2.08-2.00 (1H, m, CH_2), 1.98-1.79 (3H, m, CH_2), 1.70-1.95 (1H, m, CH_2), 1.34 (3H, s, CH_3), 1.26 (1H, d, *J* = 14.68 Hz, CH_2), 1.13 (1 H, d, *J* = 14.68 Hz, CH_2), -0.02 (9 H, s, $SiCH_3$); $^{13}C\{^1H\}$ NMR ($CDCl_3$, 101 MHz) δ_C 147.2 (C), 142.3 (C), 138.6 (C), 138.1 (C), 133.2 (C), 132.3 (C), 130.7 (CH), 130.5 (CH), 130.1 (CH), 130.0 (C), 128.1 (CH), 128.0 (CH), 127.9 (CH), 127.2 (CH), 127.2 (CH), 127.1 (CH), 124.8 (C), 110.2 (CH), 55.4 (CH), 43.6 (CH), 39.2 (NCH_2), 37.2 (CH_2),

35.0 (C), 33.6 (CH_3), 32.8 (CH_2), 30.5 (CH_2), 20.4 (CH_3), 19.7 (CH_2), 0.8 (CH_3). HRMS (ESI$^+$) m/z: calcd for $C_{31}H_{37}{}^{35}Cl_2NSi$ $[M]^+$ 521.2067; found 521.2075.

3.6 Reduction of DHQs

General Procedure 10: Reduction of dihydroquinolines (DHQs) with $NaBH_4$

The dihydroquinoline **3’** (0.05 mmol) was dissolved in THF (1 mL) in an open vial and stirred at room temperature. $NaBH_4$ (10-30 equiv., 2.00 mmol, 75.6 mg) was added to the solution. After 30 min, a 25% v/v solution of AcOH in THF (0.5 mL) was added dropwise with vigorous stirring. After 24 h, to the mixture was added 5 mL saturated $NaHCO_3$, extracted with Et_2O (5 mL × 2). The combined organic phases were dried over $MgSO_4$ and the solvent removed *in vacuo*. A spectroscopic yield was obtained using nitromethane as an internal standard and ^{1}H-NMR spectroscopy.[38]

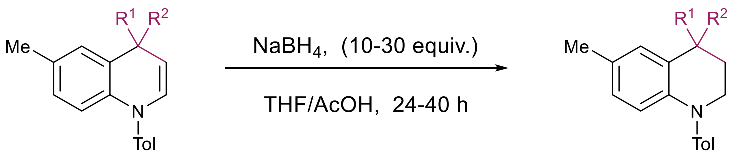

3' → **3**

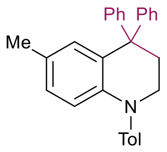

3ad, 100%

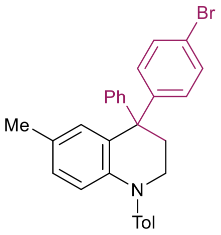

3af, 64%

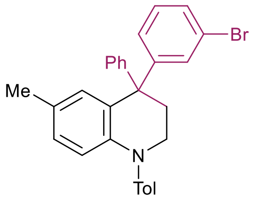

3ai, 72%

6-Methyl-4,4-diphenyl-1-(*p*-tolyl)-1,2,3,4-tetrahydroquinoline, 3ad

Following General Procedure 10, using 6-methyl-4,4-diphenyl-1-(p-tolyl)-1,4-dihydroquinoline, **3ad'** (19.4 mg, 50.0 µmol), $NaBH_4$ (18.2 mg, 0.500 mmol) in AcOH solution (8% in THF, 1.5 mL) for 24 h at rt gave the compound **3ad** (quant. spectroscopic yield).

4-(4-Bromophenyl)-6-methyl-4-phenyl-1-(*p*-tolyl)-1,2,3,4-tetrahydroquinoline, 3af

Following General Procedure 10, using 4-(4-bromophenyl)-6-methyl-4-phenyl-1-(*p*-tolyl)-1,4-dihydroquinoline, **3af'** (34.0 mg, 73.0 µmol), $NaBH_4$ (27.6 mg, 0.730 mmol) in AcOH solution (8% in THF, 2.3 mL) for 24 h at rt gave the compound **3af** (64% spectroscopic yield).

4-(3-Bromophenyl)-6-methyl-4-phenyl-1-(*p*-tolyl)-1,2,3,4-tetrahydroquinoline, 3ai

Following General Procedure 10, using 4-(3-bromophenyl)-6-methyl-4-phenyl-1-(p-tolyl)-1,4-dihydroquinoline, **3ai'** (36.0 mg, 77.0 μmol), $NaBH_4$ (87.0 mg, 2.77 mmol) in AcOH solution (8% in THF, 7.2 mL) for 40 h at rt gave the compound **3ai** (72% spectroscopic yield).

3.7 Mechanistic studies

3.7.1 Uncyclised Products 4

2-Chloro-*N*-methyl-*N*-(3-methyl-4-(trimethylsilyl)butyl)aniline, 4a

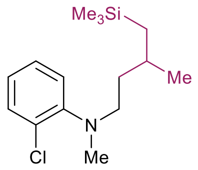

Following General Procedure 8, using 2-chloro-*N*,*N*-dimethylaniline **1ae** (31.6 mg, 0.200 mmol), methylallyltrimethylsilane **2a** (80 μL, 0.44 mmol) in DCE (0.5 mL) for 22 h at 85 °C, gave the compound **4a** (16% spectroscopic yield).

Following General Procedure 9, using 2-chloro-*N*,*N*-dimethylaniline **1ae** (30.9 mg, 0.200 mmol), methylallyltrimethylsilane **2a** (0.11 mL, 0.60 mmol), 2,6-dichloropyridine (3.50 mg, 20.0 μmol) in *o*-DCB (0.50 mL) for 22 h at 120 °C, gave the compound **4a** (25% spectroscopic yield).

Following General Procedure 8, using 2-chloro-*N*,*N*-dimethylaniline **1ae** (77.7 mg, 0.500 mmol), methylallyltrimethylsilane **2a** (0.31 mL, 1.8 mmol) in DCE (1.00 mL) for 120 h at 85 °C, gave the compound **4a** (26% spectroscopic yield). Purification via flash column chromatography on silica gel (eluent = 20% DCM/petroleum ether) gave the title compound **4a** as a colourless oil (29.6 mg, 104 μmol, 21%).

R_f = 0.3 (20% DCM/petroleum ether). ^{1}H NMR (400 MHz, $CDCl_3$) δ_H 7.36 (1H, dd, *J* = 7.9, 1.2 Hz, ArH), 7.20 (1H, m, ArH), 7.07 (1H, m, ArH), 6.94 (1H, m, ArH), 3.09-2.97 (2H, m, NCH_2), 2.78 (3H, s, NCH_3), 1.67-1.57 (2H, m, CH_2, CH), 1.49-1.40 (1H, m, CH_2), 0.92 (3H, d, *J* = 6.5 Hz, CH_3), 0.63 (1H, dd, *J* = 14.6, 5.0 Hz, $SiCH_2$), 0.42 (1H, dd, *J* = 14.6, 5.0 Hz, $SiCH_2$), -0.01 (9H, s, $SiCH_3$); ^{13}C{^{1}H} NMR ($CDCl_3$, 101 MHz) δ_C 150.1 (C), 130.6 (CH), 129.0 (C), 127.2 (CH), 123.1 (CH), 121.3 (CH), 54.2 (NCH_2), 40.9 (NCH_3), 37.7 (CH_2), 27.9 (CH), 25.4 (CH_2), 23.0 (CH_3), -0.63 ($SiCH_3$). HRMS (ESI$^+$) m/z: calcd for $C_{15}H_{27}{}^{35}ClNSi$ $[M+H]^+$ 284.1596; found 284.1606; calcd for $C_{15}H_{27}{}^{37}ClNSi$ $[M+H]^+$ 286.1566; found 286.1577.

N-Methyl-N-(3-methyl-4-(trimethylsilyl)butyl)-2-phenoxyaniline, 4b

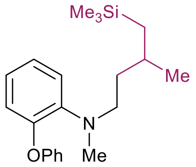

Following General Procedure 8, using *N*,*N*-dimethyl-2-phenoxyaniline **1ag** (42.5 mg, 0.200 mmol), methylallyltrimethylsilane **2a** (80 µL, 0.44 mmol) in DCE (0.5 mL) for 22 h at 85 °C, gave the title compound **4b** (35% spectroscopic yield). Purification via flash column chromatography on silica gel (eluent = 1% EtOAc/petroleum ether) gave the title compound **4b** as a clear oil (7.8 mg, 20 µmol, 11%).

R_f = 0.2 (1% EtOAc/petroleum ether). ^{1}H NMR (400 MHz, $CDCl_3$) δ_H 7.31-7.29 (2H, m, ArH), 7.11-7.06 (1H, m, ArH), 7.05-6.99 (2H, m, ArH), 6.96-6.93 (2H, m, ArH), 6.90-6.88 (2H, m, ArH), 3.12-3.08 (2H, m, NCH_2), 2.80 (3H, s, NCH_3), 1.52-1.45 (2H, m, CH_2, CH), 1.38-1.33 (1H, m, CH_2), 0.83 (3H, d, *J* = 6.4 Hz, CH_3), 0.54 (1H, dd, *J* = 14.7, 4.6 Hz, $SiCH_2$), 0.34 (1H, dd, *J* = 14.7, 8.4 Hz, $SiCH_2$), -0.05 (9H, s, $SiCH_3$); ^{13}C{^{1}H} NMR ($CDCl_3$, 101 MHz) δ_C 157.7 (C), 148.3 (C), 144.5 (C), 129.4 (CH), 124.3 (CH), 122.2 (CH), 121.3 (CH), 121.1 (CH), 119.5 (CH), 117.4 (CH), 53.5 (NCH_2), 40.0 (NCH_3), 37.5 (CH_2), 27.8 (CH), 25.3 (CH_2), 22.9 (CH_3), -0.65 ($SiCH_3$). HRMS (ESI$^+$) m/z: calcd for $C_{21}H_{32}NOSi$ $[M+H]^+$ 342.2248; found 342.2253.

3.7.2 NMR Experiments

In Situ NMR Experiments Observing Transfer Hydrogenation of Alkenes 2a and 2d

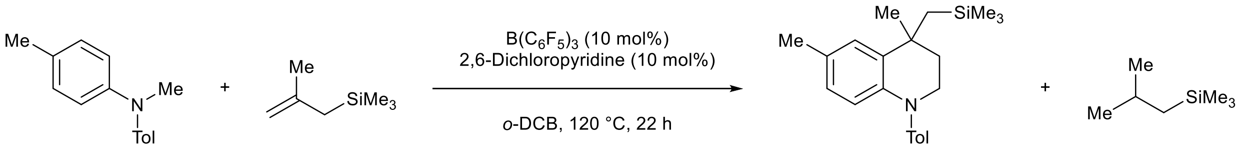

An oven-dried J. Young NMR tube was cooled under vacuum and then placed in the glove box. $B(C_6F_5)_3$ (10.2 mg, 20.0 µmol) was added to the J. Young NMR tube. The NMR tube was sealed and removed from the glovebox, placed in the Schlenk flask and the atmosphere was cycled three times via vacuum-N_2 backfills. *N*,4-dimethyl-*N*-(*p*-tolyl)aniline **1a** (42.3 mg, 0.200 mmol) and 2,6-dichloropyridine (2.9 mg, 20 µmol) were weighed into a vial and sealed with a septa. The atmosphere in the vial was cycled three times via vacuum-N_2 backfills and the amine and pyridine were subsequently dissolved in *o*-DCB (0.3 mL). The solution was transferred to the J. Young NMR tube containing $B(C_6F_5)_3$ using standard syringe-septa techniques. The vial was washed with *o*-DCB (0.2 mL) and the washings were transferred to the J. Young NMR tube. Methylallyltrimethylsilane **2a** (0.1 mL, 0.6 mmol) was added before the J. Young NMR tube was sealed, and the mixture was heated at 120 °C for 22 h. The reaction was allowed to cool to room temperature and a spectroscopic yield of compound **3a** and **5a** were

determined using ^{1}H-NMR spectroscopy and nitromethane as an internal standard. The NMR signals of the known compound **5a** were assigned based on reported data.[39]

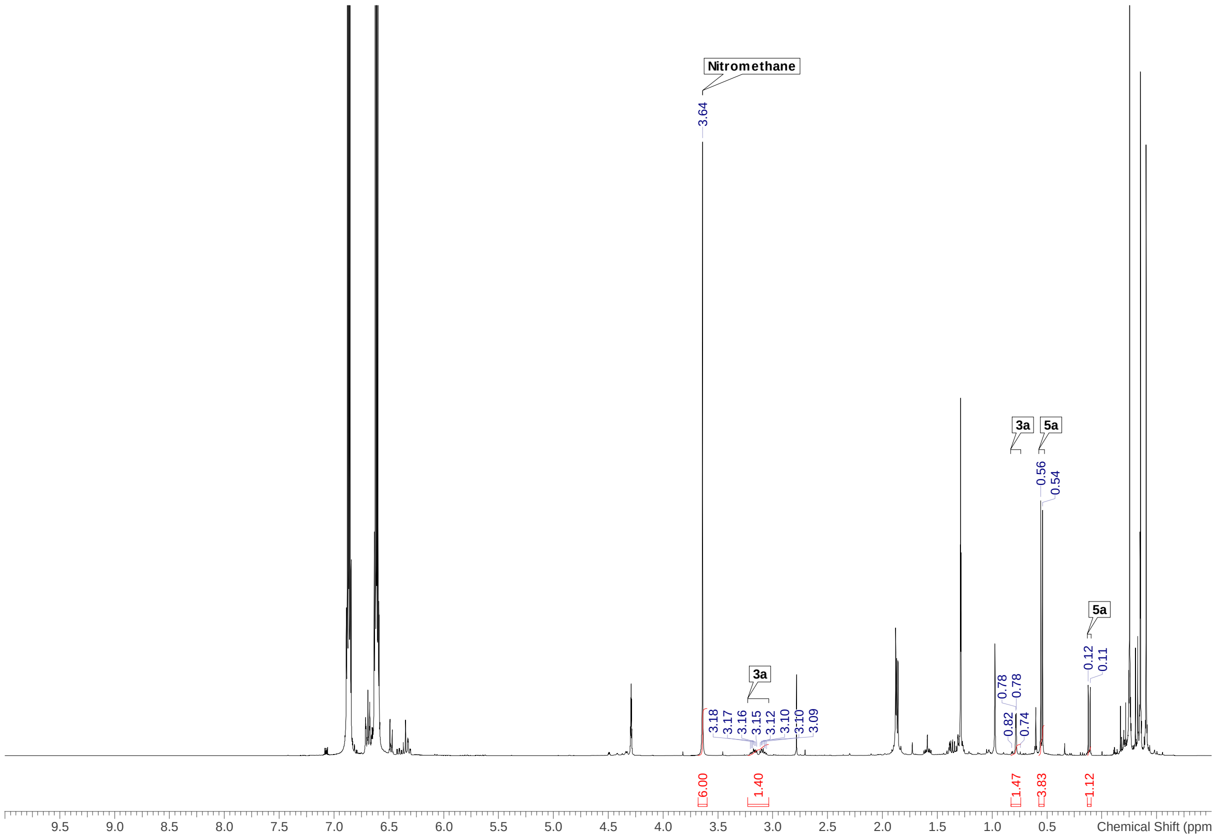

Figure S1. ^{1}H-NMR (400 MHz) of the reaction mixture of **1a** with **2a**.

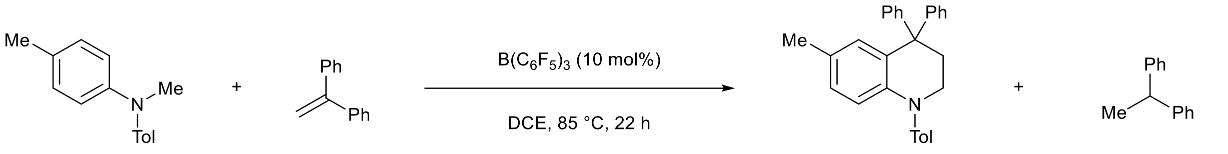

1a 0.20 mmol

2d 0.44 mmol

3ad 0.18 mmol (89%)

5b 0.21 mmol (105%)

An oven-dried J. Young NMR tube was cooled under vacuum and then placed in the glove box. $B(C_6F_5)_3$ (10.2 mg, 20.0 µmol) was added to the J. Young NMR tube. The NMR tube was sealed and removed from the glovebox, placed in the Schlenk flask and the atmosphere was cycled three times via vacuum-N_2 backfills. *N*,4-dimethyl-*N*-(*p*-tolyl)aniline **1a** (42.3 mg, 0.200 mmol) was weighed into a vial and sealed with a septa. The atmosphere in the vial was cycled three times via vacuum-N_2 backfills and the amine was subsequently dissolved in DCE (0.3 mL). The solution was transferred to the J. Young NMR tube containing $B(C_6F_5)_3$ using standard syringe-septa techniques. The vial was washed with DCE (0.2 mL) and the washings were transferred to the J. Young NMR tube. 1,1-Diphenylethylene **2d** (77 µL, 0.44 mmol) was added before the J. Young NMR tube was sealed, and the mixture was heated at 85 °C for 22 h. The reaction was allowed to cool to room temperature and a spectroscopic yield of compound

3ad and **5b** were determined using ^{1}H-NMR spectroscopy and dibromomethane as an internal standard. The NMR signals of the known compound **5b** were assigned based on reported data.[40]

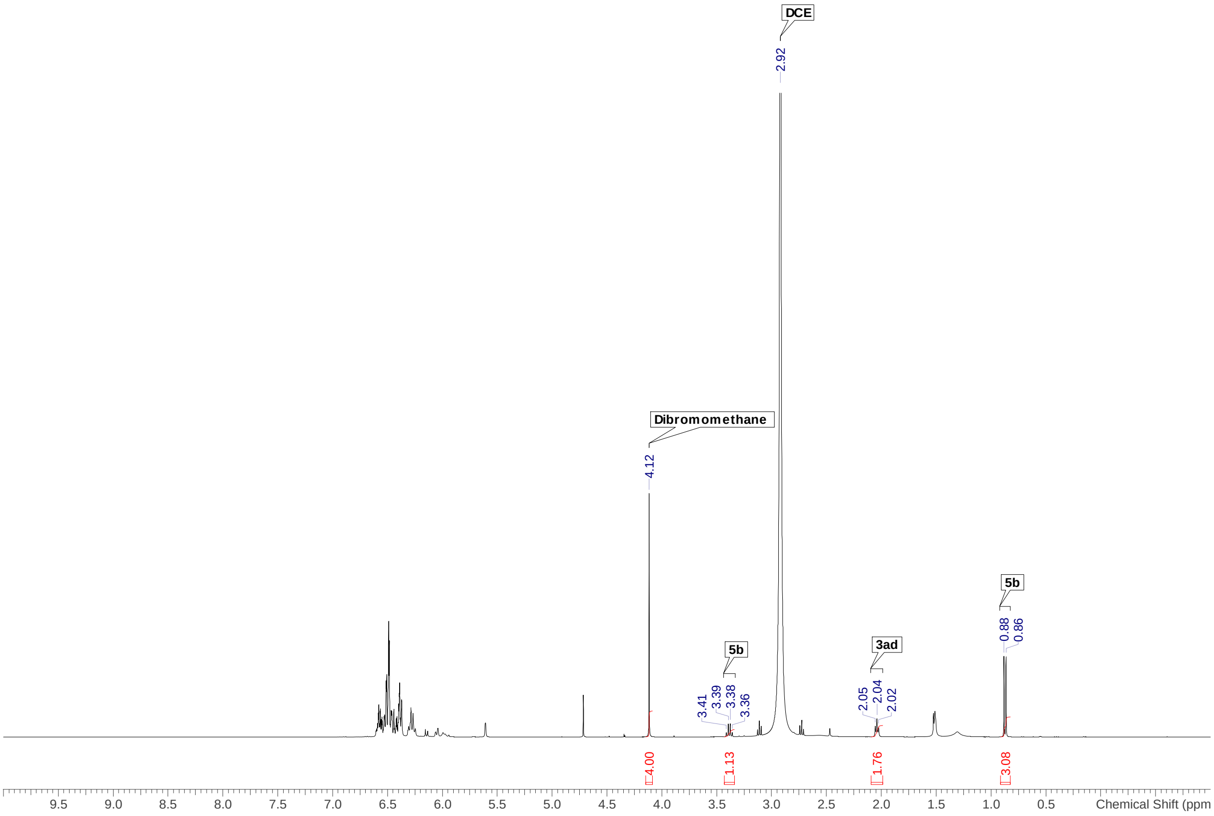

Figure S2. ^{1}H-NMR (400 MHz) of the reaction mixture of **1a** with **2d**.

NMR Experiments of 2,6-Dichloropyridine and $B(C_6F_5)_3$

An oven-dried J. Young NMR tube was cooled under vacuum and then placed in an Ar filled glove box. $B(C_6F_5)_3$ (25.5 mg, 50.0 µmol) was added to the J. Young NMR tube. The NMR tube was sealed and removed from the glovebox, placed in the Schlenk flask and the atmosphere was cycled three times via vacuum-N_2 backfills. 2,6-Dichloropyridine (7.4 mg, 50 µmol) was weighed into a vial and sealed with a septa. The atmosphere in the vial was cycled three times via vacuum-N_2 backfills, and 2,6-dichloropyridine was subsequently dissolved in anhydrous C_6D_6 (0.3 mL). The solution was transferred to the J. Young NMR tube containing $B(C_6F_5)_3$ using standard syringe-septa techniques. The vial was washed with C_6D_6 (0.2 mL) and the washings were transferred to the J. Young NMR tube. The NMR tube was sealed and ^{1}H, ^{11}B and ^{19}F NMR spectra were recorded shortly after.

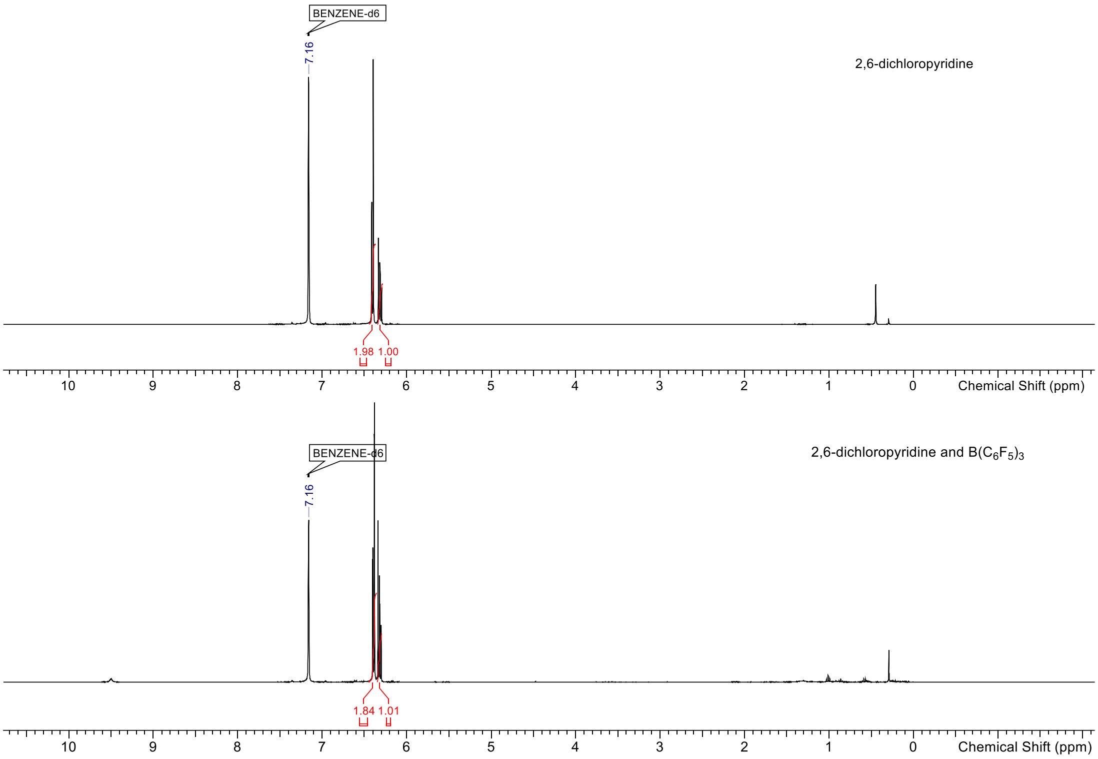

Figure S3. ^{1}H-NMR (C_6D_6, 400 MHz) spectra of 2,6-dichloropyridine compared to $B(C_6F_5)_3$ and 2,6-dichloropyridine.

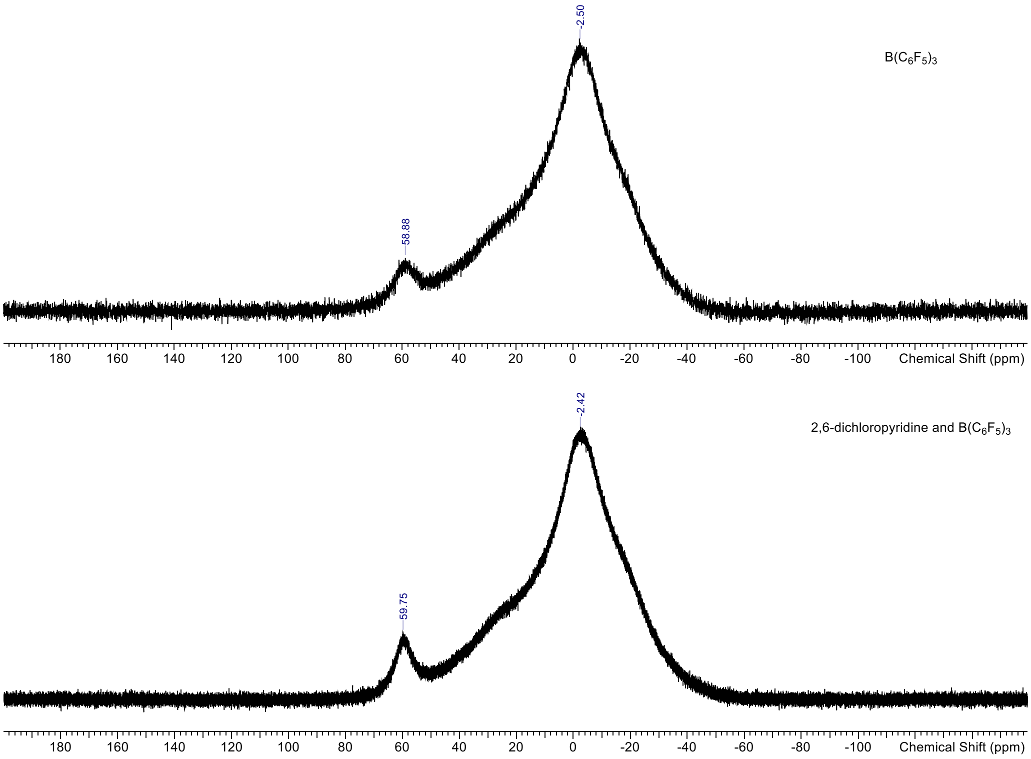

Figure S4. ^{11}B-NMR (C_6D_6, 128 MHz) spectra of $B(C_6F_5)_3$ compared to $B(C_6F_5)_3$ and 2,6-dichloropyridine.

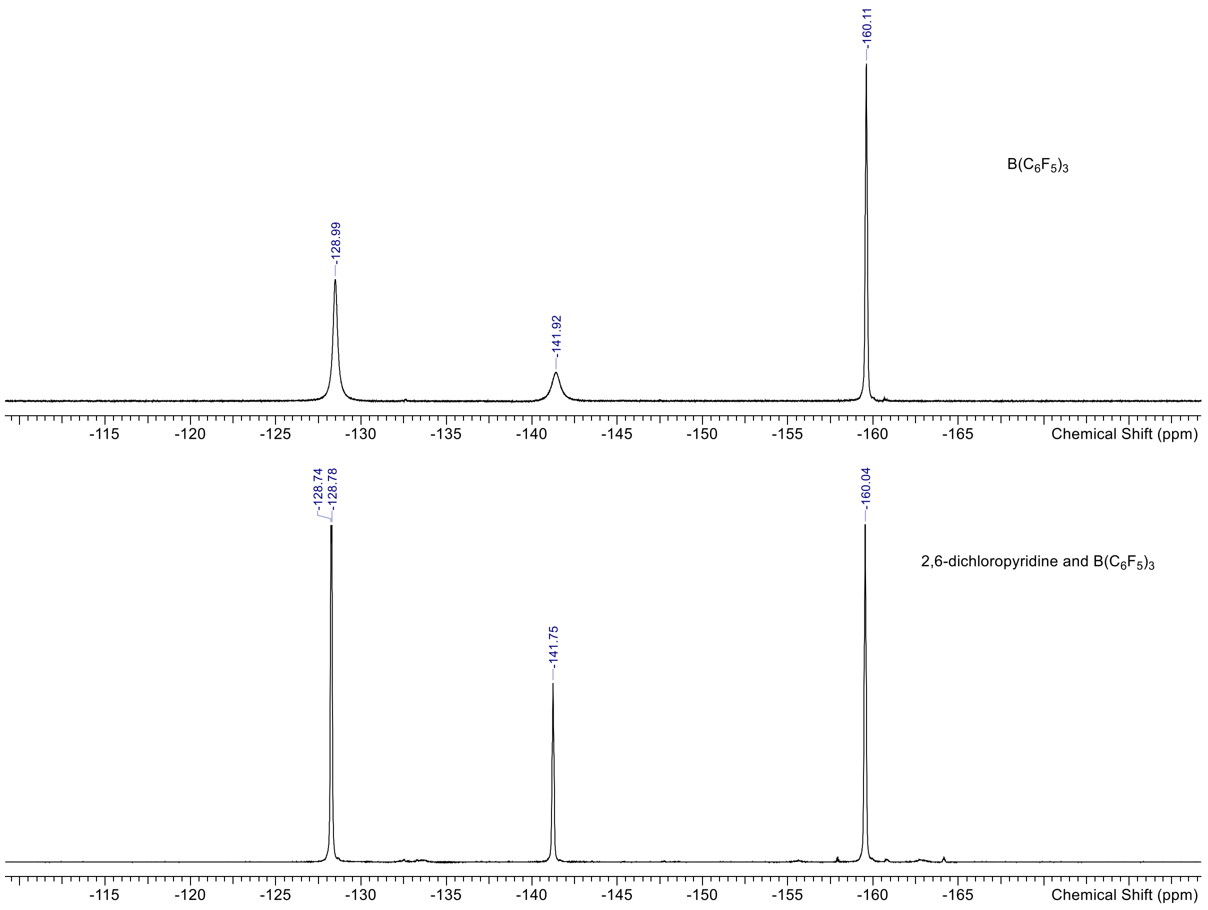

Figure S5. ^{19}F-NMR (C_6D_6, 376 MHz) spectra of $B(C_6F_5)_3$ compared to $B(C_6F_5)_3$ and 2,6-dichloropyridine.

3.8 Derivatization of Tetrahydroquinolines 3r and 3t

1-Benzyl-4-methyl-4-((trimethylsilyl)methyl)-1,2,3,4-tetrahydroquinoline-6-carbaldehyde, 9a

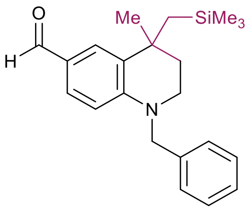

Following an adapted literature procedure,[41] to a stirred solution of THQ **3r** (20 mg, 60 µmol) in THF/H_2O (1.2 mL, 1:1), DDQ (27.2 mg, 0.120 mmol, 2.00 equiv.) was added and the mixture was stirred at r.t. for 40 mins. An aqueous sat. $NaHCO_3$ solution (1 mL) was added, and the resulting mixture was extracted with DCM (2 mL × 3). The combined organic phases were dried over $MgSO_4$, filtered and concentrated *in vacuo*. The crude product was purified via column chromatography on silica gel (eluent = 2.5% EtOAc/petroleum ether) to obtain the title compound **9a** as a pale-yellow oil (10.5 mg, 30.0 µmol, 50%).

R_f = 0.50 (10% EtOAc/petroleum ether). ^{1}H NMR (400 MHz, $CDCl_3$) δ_H 9.69 (1H, s, CHO), 7.77 (1H, d, *J* = 2.0 Hz, ArH), 7.45 (1H, dd, *J* = 8.6, 2.0 Hz, ArH), 7.37-7.33 (2H, m, ArH), 7.30-7.27 (1H, m, ArH), 7.22-7.20 (2H, m, ArH), 6.54 (1H, d, *J* = 8.6 Hz, ArH), 4.63 (2H, s, NCH_2Ph), 3.55-3.46 (2H, m, NCH_2), 1.97-1.91 (1H, m, CH_2), 1.86-1.79 (1H, m, CH_2), 1.43 (3H, s, CH_3), 1.19 (1H, d, *J* = 14.8 Hz, $SiCH_2$), 1.15 (1H, d, *J* = 14.8 Hz, $SiCH_2$), -0.01 (9H, s, $SiCH_3$); $^{13}C\{^1H\}$ NMR ($CDCl_3$, 101 MHz) δ_C 190.3 (CHO), 149.2 (C), 137.0 (C), 132.1 (C), 131.1 (CH), 128.9 (CH), 127.3 (CH), 127.1 (CH), 126.3 (CH), 125.1 (C), 110.3 (CH), 54.9 (NCH_2), 46.4 (NCH_2), 35.8 (CH_2), 34.8 (C), 31.6 ($SiCH_2$), 30.7 (CH_3), 0.8 ($SiCH_3$). IR (neat)/cm^{-1} 2808, 2723, 1670, 1246, 835. HRMS (ESI+) m/z: calcd for $C_{22}H_{30}NOSi$ $[M+H]^+$ 352.2091; found 352.2097.

1-Benzhydryl-4-methyl-4-((trimethylsilyl)methyl)-1,2,3,4-tetrahydroquinoline-6-carbaldehyde, 9b

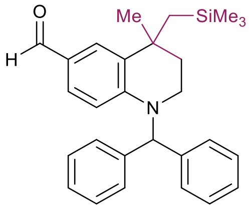

Following a literature procedure,[41] to a stirred solution of THQ **3t** (82.7 mg, 0.200 mmol) in $CHCl_3$ (3 mL), DDQ (90.8 mg, 0.400 mmol, 2.00 equiv.) was added and the mixture was stirred at r.t. for 24 h. An aqueous saturated $NaHCO_3$ solution (1 mL) was added, and the resulting mixture was extracted with DCM (2 mL × 3). The combined organic phases were dried over $MgSO_4$, filtered and concentrated *in vacuo*. The crude product was purified via column chromatography on silica gel (eluent = 10% EtOAc/petroleum ether) to obtain the title compound **9b** as a pale-yellow oil (69 mg, 0.16 mmol, 81%).

R_f = 0.2 (10% EtOAc/petroleum ether). ^{1}H NMR (400 MHz, $CDCl_3$) δ_H 9.71 (1H, s, CHO), 7.80 (1H, d, *J* = 2.0 Hz, ArH), 7.47 (1H, dd, *J* = 8.6, 2.0 Hz, ArH), 7.41-7.34 (6H, m, ArH), 7.21-7.19 (4H, m, ArH), 6.61 (1H, d, *J* = 9.0 Hz, ArH), 6.29 (1H, s, NCH), 3.12-2.98 (2H, m, NCH_2), 1.85-1.73 (2H, m, CH_2), 1.39 (3H, s, CH_3), 1.11 (1H, d, *J* = 15.3 Hz, $SiCH_2$), 1.07 (1H, d, *J* = 15.3 Hz, $SiCH_2$), -0.05 (9H, s, $SiCH_3$); $^{13}C\{^1H\}$ NMR ($CDCl_3$, 101 MHz) δ_C 190.4 (CHO), 149.6 (C), 139.2 (C), 138.7 (C), 133.1 (C), 129.0 (CH), 128.7 (CH), 128.6 (CH), 127.8 (CH), 127.6 (CH), 125.1 (C), 111.0 (CH), 66.2 (NCH), 42.1 (NCH_2), 35.8 (CH_2), 34.8 (C), 30.8 ($SiCH_2$), 30.2 (CH_3), 0.69 ($SiCH_3$). HRMS (ESI+) m/z: calcd for $C_{28}H_{34}NOSi$ $[M+H]^+$ 428.2404; found 428.2413.

1-Benzhydryl-4,6-dimethyl-4-((trimethylsilyl)methyl)-1,4-dihydroquinoline, 10

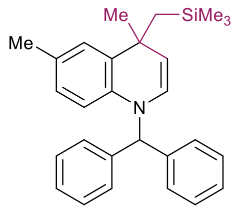

Following an adapted literature procedure,[41] a mixture of THQ **3t** (200 mg, 0.480 mmol), pyridine (0.21 mL, 2.4 mmol, 5.0 equiv.) and $BaMnO_4$ (2.5 g, 9.7 mmol, 20 equiv.) in cyclohexane (25 mL) was stirred in an open vessel for 24 h at 55 °C. The crude mixture was allowed to reach r.t. and filtered through a Celite 545, and the filtrate was concentrated *in vacuo*. The reaction mixture was purified via column chromatography on silica gel (eluent = 5% DCM/petroleum ether) to obtain the title compound **10** as a pale-yellow oil (100 mg, 240 µmol, 51 %).

R_f = 0.40 (15% DCM/petroleum ether). ^{1}H NMR (400 MHz, $CDCl_3$) δ_H 7.37-7.28 (6H, m, ArH), 7.24-7.21 (4H, m, ArH), 7.07 (1H, d, *J* = 2.0 Hz, ArH), 6.80 (1H, ddd, *J* = 8.4, 2.0, 0.6 Hz), 6.61 (1H, d, *J* = 8.4 Hz), 6.27 (1H, s, NCH), 5.84 (1H, d, *J* = 8.1 Hz, NCH), 4.31 (1H, d, *J* = 8.1 Hz, CH), 2.26 (3H, s, CH_3), 1.46 (3H, s, CH_3), 1.35 (1H, d, *J*=14.6 Hz, $SiCH_2$), 0.93 (1H, d, *J*=14.6 Hz, $SiCH_2$), -0.11 (9H, s, $SiCH_3$); $^{13}C\{^1H\}$ NMR ($CDCl_3$, 101 MHz) δ_C 140.5 (C), 140.3 (C), 137.4 (C), 132.1 (C), 129.3 (C), 129.1 (CH), 128.6 (CH), 128.6 (CH), 128.5 (CH), 127.6 (CH), 127.5 (CH), 127.3 (CH), 126.9 (CH), 125.7 (CH), 111.7 (CH), 107.9 (CH), 64.3 (NCH), 37.1 (CH_3), 36.5 ($SiCH_2$), 35.6 (C), 20.6 (CH_3), 0.4 ($SiCH_3$). HRMS (ESI$^+$) m/z: calcd for $C_{28}H_{34}NSi$ $[M+H]^+$ 412.2455; found 412.2457.

1-Benzyl-4,6-dimethyl-4-((trimethylsilyl)methyl)-3,4-dihydroquinolin-2(1*H*)-one, 11

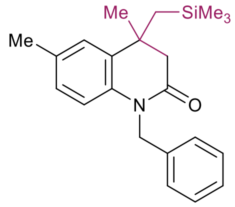

Following a literature procedure,[42] to a stirred mixture of THQ **3r** (50 mg, 0.15 mmol) and $NaHCO_3$ (125 mg, 1.48 mmol, 10.0 equiv.) in THF/H_2O (6 mL, 2.5:1), iodine (282 mg, 1.11 mmol, 7.50 equiv.) was added. The reaction mixture was stirred at r.t. for 20 mins and then quenched with sat. aqueous $Na_2S_2O_3$ (2 mL) and sat. $NaHCO_3$ (2 mL) solutions. The reaction mixture was extracted with EtOAc (3 mL × 3) and the combined organic phases were dried over $MgSO_4$. After filtration, the solvent was removed *in vacuo* and the crude product was purified via column chromatography on silica gel (eluent = 5% EtOAc/petroleum ether) to obtain the title compound **11** as a pale-yellow oil (25.5 mg, 72.6 µmol, 49%).

R_f = 0.40 (10% EtOAc/petroleum ether). ^{1}H NMR (400 MHz, $CDCl_3$) δ_H 7.31- 7.26 (4H, m, ArH), 7.24-7.21 (1H, m, ArH), 7.11 (1H, d, *J* = 1.7 Hz, ArH), 6.93-6.91 (1H, m, ArH), 6.83 (1H, d, *J* = 8.2 Hz,

ArH), 5.20 (2H, s, NCH_2Ph), 2.68 (1H, d, J = 15.3 Hz, CH_2), 2.65 (1H, d, J = 15.3 Hz, CH_2), 2.30 (3H, s, CH_3), 1.43 (3H, s, CH_3), 1.11 (1H, d, J = 14.8 Hz, $SiCH_2$), 0.98 (1H, d, J = 14.8 Hz, $SiCH_2$), -0.12 (9H, s, $SiCH_3$); $^{13}C\{^1H\}$ NMR ($CDCl_3$, 101 MHz) δ_C 169.8 (C), 137.3 (C), 135.9 (C), 135.4 (C), 132.5 (C), 128.6 (CH), 127.5 (CH), 127.1 (CH), 126.9 (CH), 125.4 (CH), 115.8 (CH), 47.6 (CH_2), 45.5 (NCH_2), 35.7 (C), 29.0 ($SiCH_2$), 27.6 (CH_3), 20.8 (CH_3), 0.2 ($SiCH_3$). IR (neat)/cm^{-1} 1670, 1247, 834. HRMS (ESI^+) m/z: calcd for $C_{22}H_{30}NOSi$ $[M+H]^+$ 352.2091; found 352.2097.

4,6-Dimethyl-4-((trimethylsilyl)methyl)-1,2,3,4-tetrahydroquinoline, 12

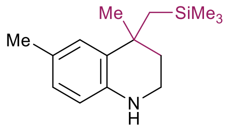

Following a literature procedure,[43] to a stirred solution of THQ **3r**/**3t** (20.0 mg/24.8 mg, 60.0 µmol) and 10% Pd/C (12.0 mg) in MeOH (0.75 mL), $HCO_2NH_4\cdot5H_2O$ (37.8 mg, 0.600 mmol, 10.0 equiv.) was added in a single portion. The reaction mixture was stirred at 65 °C for 1 h 20 mins. After completion, as determined by TLC, the mixture was filtered through Celite 545 and concentrated under reduced pressure. The residue was dissolved in DCM (2 mL), washed with H_2O (2 mL) and dried over $MgSO_4$. After filtration, the solvent was removed *in vacuo* and the crude product was purified via column chromatography on silica gel (eluent = 5% EtOAc/petroleum ether) to obtain the product **12** as a yellow oil (8.6 mg, 35 µmol, 59% from THQ **3r**; 10.7 mg, 43.3 µmol, 72% from THQ **3t**).

R_f = 0.45 (10% EtOAc/petroleum ether). ^1H NMR (400 MHz, $CDCl_3$) δ_H 6.99 (1H, d, J = 1.7 Hz, ArH), 6.77-6.75 (1H, m, ArH), 6.39 (1H, d, J = 8.0 Hz, ArH), 3.73 (1H, br s, NH), 3.31-3.26 (2H, m, NCH_2), 2.23 (3H, s, CH_3), 1.96-1.89 (1H, m, CH_2), 1.76-1.70 (1H, m, CH_2), 1.36 (3H, s, CH_3), 1.22 (1H, d, J = 15.1 Hz, $SiCH_2$), 1.12 (1H, d, J = 15.1 Hz, $SiCH_2$), -0.03 (9H, s, $SiCH_3$); $^{13}C\{^1H\}$ NMR ($CDCl_3$, 101 MHz) δ_C 141.0 (C), 132.0 (C), 127.4 (CH), 127.0 (CH), 126.0 (C), 114.4 (CH), 38.7 (NCH_2), 37.2 (CH_2), 34.5 (C), 33.2 ($SiCH_2$), 32.9 (CH_3), 20.7 (CH_3), 0.8 ($SiCH_3$). IR (neat)/cm^{-1} 3388, 1246, 832. HRMS (ESI^+) m/z: calcd for $C_{15}H_{26}NSi$ $[M+H]^+$ 248.1829; found 248.1839.

(4*RS*,4a*RS*,8a*RS*)-4,6-Dimethyl-4-((trimethylsilyl)methyl)decahydroquinoline, 13

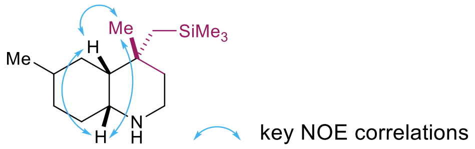

Following a literature procedure,[44] a mixture of THQ **3t** (150 mg, 0.362 mmol), Rh/C (45 mg, 30 wt%, 5 wt% Rh) and *i*-PrOH (3 mL) were stirred in an autoclave under a hydrogen atmosphere (10 atm) at 25 °C for 72 h. The reaction mixture was filtered through Celite 545 and the solvent was removed *in vacuo*. The crude mixture was purified via flash column chromatography on silica gel (eluent = 2:2:96 v.v. MeOH:Et_3N:$CHCl_3$) to obtain the decahydroquinoline **13** as an oil (48 mg, 0.19 mmol, 52% yield; 3:1 *dr* [major diastereoisomer:all minor diastereoisomers]). Repurification by flash column

chromatography on silica gel (eluent = 2:1:97 v.v. MeOH:Et_3N:$CHCl_3$) gave the major diastereoisomer **13** as a pale-yellow oil (17 mg, 67 μmol, 19%).

R_f = 0.40 (10% MeOH/ $CHCl_3$). ^{1}H NMR (400 MHz, $CDCl_3$) δ_H 3.16 - 3.08 (1H, m, CH), 2.96 - 2.83 (2H, m, CH_2), 2.30 (1H, br s, NH), 1.74 (1H, qd, *J* = 3.0, 13.8 Hz, CH_2), 1.57 - 1.35 (4H, m, CH_2), 1.34 - 1.06 (5H, m, CH, CH_2) 1.10 (3H, s, CH_3), 0.93 (3H, d, *J* = 6.1 Hz, CH_3), 0.71 (1H, d, *J* = 14.7 Hz, $SiCH_2$), 0.56 (1H, d, *J* = 14.7 Hz, $SiCH_2$), 0.04 (9H, s, $SiCH_3$); $^{13}C\{^1H\}$ NMR ($CDCl_3$, 101 MHz) δ_C 49.7 (CH), 46.1 (CH), 43.1 (CH_2), 36.5 (CH_2), 35.5 (C), 32.9 (CH), 32.7 (CH_2), 31.8 ($SiCH_2$), 30.8 (CH_2), 28.8 (CH_2), 25.9 (CH_3), 22.9 (CH_3), 1.2 (CH_3). HRMS (ESI^+) m/z: calcd for $C_{15}H_{32}NSi$ $[M+H]^+$ 254.2299; found 254.2304.

4 References

1. T. Iwamoto, A. Hosokawa and M. Nakamura, *Chem. Commun.*, 2019, **55**, 11683–11686.
2. T. Matsumura and M. Nakada, *Tetrahedron Lett.*, 2014, **55**, 1829–1834.
3. A. Chartoire, A. Boreux, A. R. Martin and S. P. Nolan, *RSC Adv.*, 2013, **3**, 3840.
4. M. Sai, *Adv. Synth. Catal.*, 2021, **363**, 5422–5428.
5. K. Nakaguchi, S. Ohtani, K. Kato and T. Ogoshi, *Chem. – Eur. J.*, 2025, **31**, e202500755.
6. Y. Si, Y. Zhao, W. Dai, S. Cui, P. Sun, J. Shi, B. Tong, Z. Cai and Y. Dong, *Chin. J. Chem.*, 2023, **41**, 1575–1582.
7. Y. Miki, K. Hirano, T. Satoh and M. Miura, *Org. Lett.*, 2013, **15**, 172–175.
8. C. Li, K. Wan, F. Guo, Q. Wu, M. Yuan, R. Li, H. Fu, X. Zheng and H. Chen, *J. Org. Chem.*, 2019, **84**, 2158–2168.
9. N. S. Keddie, P. A. Champagne, J. Desroches, J.-F. Paquin and D. O'Hagan, *Beilstein J. Org. Chem.*, 2018, **14**, 106–113.
10. Y. Pan, Z. Luo, J. Han, X. Xu, C. Chen, H. Zhao, L. Xu, Q. Fan and J. Xiao, *Adv. Synth. Catal.*, 2019, **361**, 2301–2308.
11. Z. Luo, Y. Pan, Z. Yao, J. Yang, X. Zhang, X. Liu, L. Xu and Q.-H. Fan, *Green Chem.*, 2021, **23**, 5205–5211.
12. F. He, C. Empel and R. M. Koenigs, *Org. Lett.*, 2021, **23**, 6719–6723.
13. K. Sun, T. Su, G. Lu, R. Franke, H. Neumann and M. Beller, *Angew. Chem. Int. Ed.*, 2025, **64**, e202419370.
14. V. H. Tran and H.-K. Kim, *Org. Biomol. Chem.*, 2022, **20**, 2881–2888.
15. X. Cui, X. Dai, Y. Deng and F. Shi, *Chem. – Eur. J.*, 2013, **19**, 3665–3675.
16. V. H. Tran, W. P. Hong and H. Kim, *Bull. Korean Chem. Soc.*, 2022, **43**, 777–783.
17. T. Taeufer and J. Pospech, *J. Org. Chem.*, 2020, **85**, 7097–7111.
18. K. Li, J. Li, B. Yin and F. Zeng, *ChemCatChem*, 2022, **14**, e202101630.
19. S. Gupta, P. Sureshbabu, A. K. Singh, S. Sabiah and J. Kandasamy, *Tetrahedron Lett.*, 2017, **58**, 909–913.
20. T. Wang, M. Hoffmann, A. Dreuw, E. Hasagić, C. Hu, P. M. Stein, S. Witzel, H. Shi, Y. Yang, M. Rudolph, F. Stuck, F. Rominger, M. Kerscher, P. Comba and A. S. K. Hashmi, *Adv. Synth. Catal.*, 2021, **363**, 2783–2795.

21. Y. Wei, Q. Xuan, Y. Zhou and Q. Song, *Org. Chem. Front.*, 2018, **5**, 3510–3514.

22. A. K. Mandal, S. Sreejith, T. He, S. K. Maji, X.-J. Wang, S. L. Ong, J. Joseph, H. Sun and Y. Zhao, *ACS Nano*, 2015, **9**, 4796–4805.

23. J. Huang, G. Hu, S. An, D. Chen, M. Li and P. Li, *J. Org. Chem.*, 2019, **84**, 9758–9769.

24. L.-H. Chen, Q. Zhang, Y.-F. Xiao, Y.-C. Fang, X. Xie and F.-J. Nan, *J. Med. Chem.*, 2022, **65**, 3991–4006.

25. H. Zhang, W. Pu, T. Xiong, Y. Li, X. Zhou, K. Sun, Q. Liu and Q. Zhang, *Angew. Chem. Int. Ed.*, 2013, **52**, 2529–2533.

26. W. Xue and M. Oestreich, *Synthesis*, 2019, **51**, 233–239.

27. W. G. Kofron and L. M. Baclawski, *J. Org. Chem.*, 1976, **41**, 1879–1880.

28. A. Krasovskiy and P. Knochel, *Synthesis*, 2006, **2006**, 0890–0891.

29. T. vom Stein, M. Peréz, R. Dobrovetsky, D. Winkelhaus, C. B. Caputo and D. W. Stephan, *Angew. Chem. Int. Ed.*, 2015, **54**, 10178–10182.

30. G. Lu, B. Lin, Y. Gao, J. Ying, G. Tang and Y. Zhao, *Synlett*, 2016, **28**, 724–728.

31. T. Torigoe, T. Ohmura and M. Suginome, *Angew. Chem. Int. Ed.*, 2017, **56**, 14272–14276.

32. C. G. Santana, Y. S. Teoh, M. M. Evarts, J. Z. Shezaf and M. J. Krische, *Org. Lett.*, 2024, **26**, 7055–7059.

33. P. K. Pandey, M. Patra, P. Ranjan, N. Kumar Pal, S. Choudhary and J. K. Bera, *Chem. – Eur. J.*, 2024, **30**, e202400337.

34. K. Tanaka, M. Kishimoto, Y. Asada, Y. Tanaka, Y. Hoshino and K. Honda, *J. Org. Chem.*, 2019, **84**, 13858–13870.

35. K. Okuma, A. Nojima, N. Matsunaga and K. Shioji, *Org. Lett.*, 2009, **11**, 169–171.

36. P. Yuan, Z. Yang, S. Zhang, C. Zhu, X. Yang and Q. Meng, *Angew. Chem. Int. Ed.*, 2024, **63**, e202313030.

37. S. Hitosugi, D. Tanimoto, W. Nakanishi and H. Isobe, *Chem. Lett.*, 2012, **41**, 972–973.

38. J. Xu, S. Zheng, J. Zhang, X. Liu and B. Tan, *Angew. Chem. Int. Ed.*, 2016, **55**, 11834–11839.

39. A. Alvarez-Montoya, J. P. Gillions, L. Winfrey, R. R. Hawker, K. Singh, F. Ortu, Y. Fu, Y. Li and A. P. Pulis, *ACS Catal.*, 2024, 4856–4864.

40. I. Chatterjee, Z. Qu, S. Grimme and M. Oestreich, *Angew. Chem. Int. Ed.*, 2015, **54**, 12158–12162.

41. E. Vicente-García, R. Ramón, S. Preciado and R. Lavilla, *Beilstein J. Org. Chem.*, 2011, **7**, 980–987.

42. R. J. Griffiths, G. A. Burley and E. P. A. Talbot, *Org. Lett.*, 2017, **19**, 870–873.

43. T. Kiguchi, N. Kuninobu, Y. Takahashi, Y. Yoshida, T. Naito and I. Ninomiya, *Synthesis*, 1989, **1989**, 778–781.

44. L.-R. Wang, D. Chang, Y. Feng, Y.-M. He, G.-J. Deng and Q.-H. Fan, *Org. Lett.*, 2020, **22**, 2251–2255.

5 NMR Spectra

N-(4-Fluorobenzyl)-4-methyl-*N*-(*p*-tolyl)aniline, 1d

^{1}H NMR (400 MHz, $CDCl_3$)

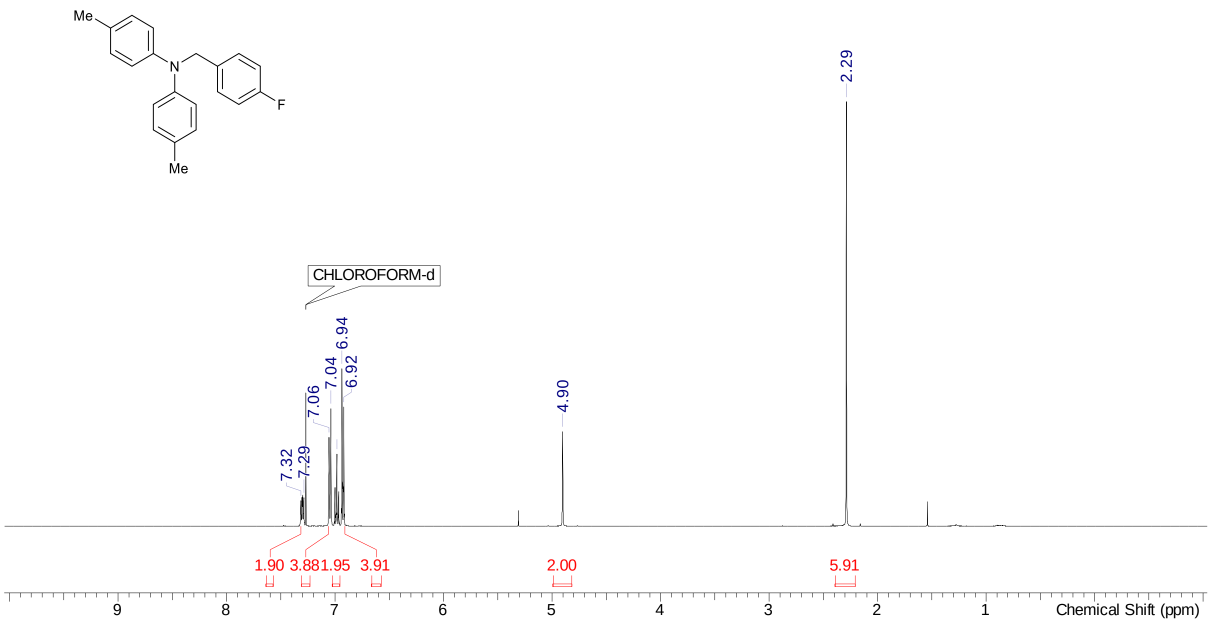

^{13}C{^{1}H} NMR ($CDCl_3$, 101 MHz)

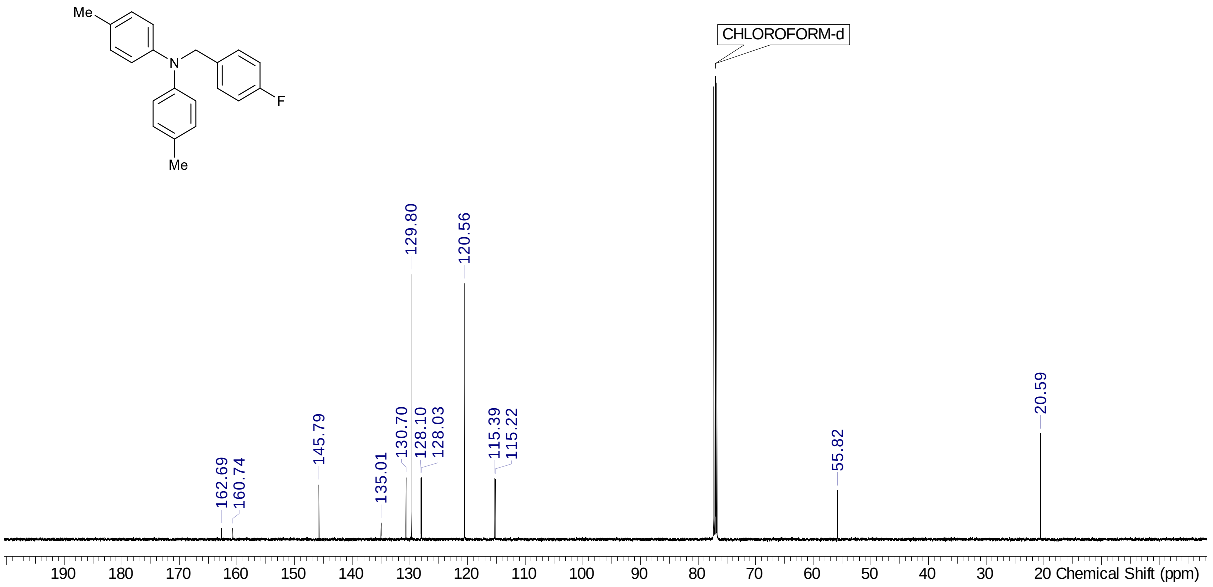

N-(4-Chlorobenzyl)-4-methyl-N-(p-tolyl)aniline, 1e

^{1}H NMR (400 MHz, $CDCl_3$)

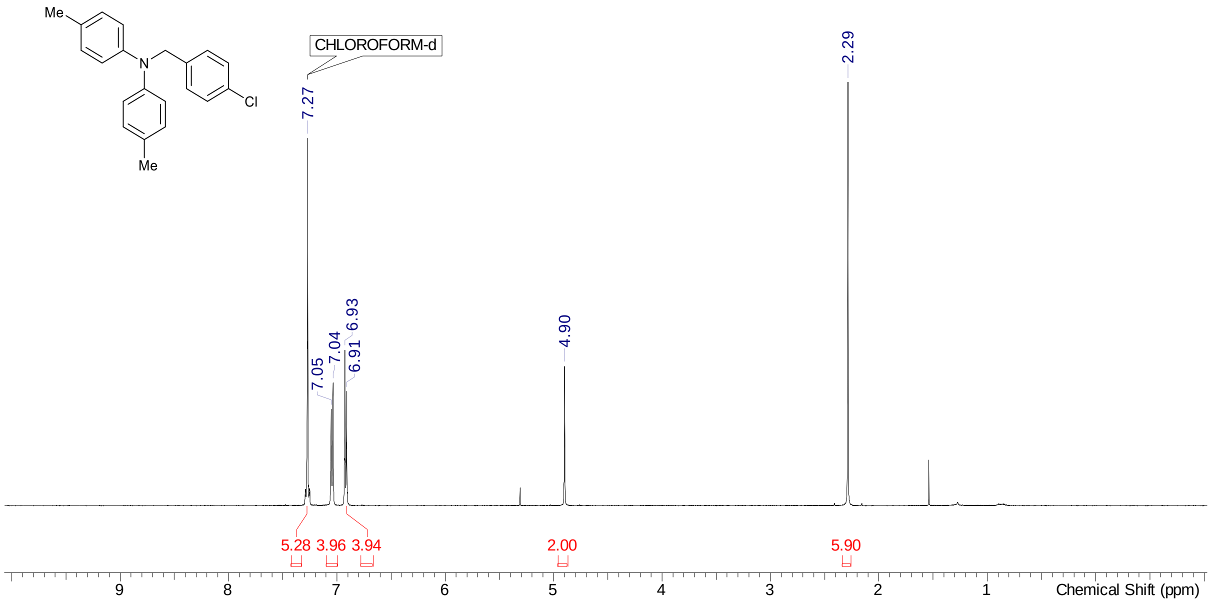

$^{13}C\{^{1}H\}$ NMR ($CDCl_3$, 101 MHz)

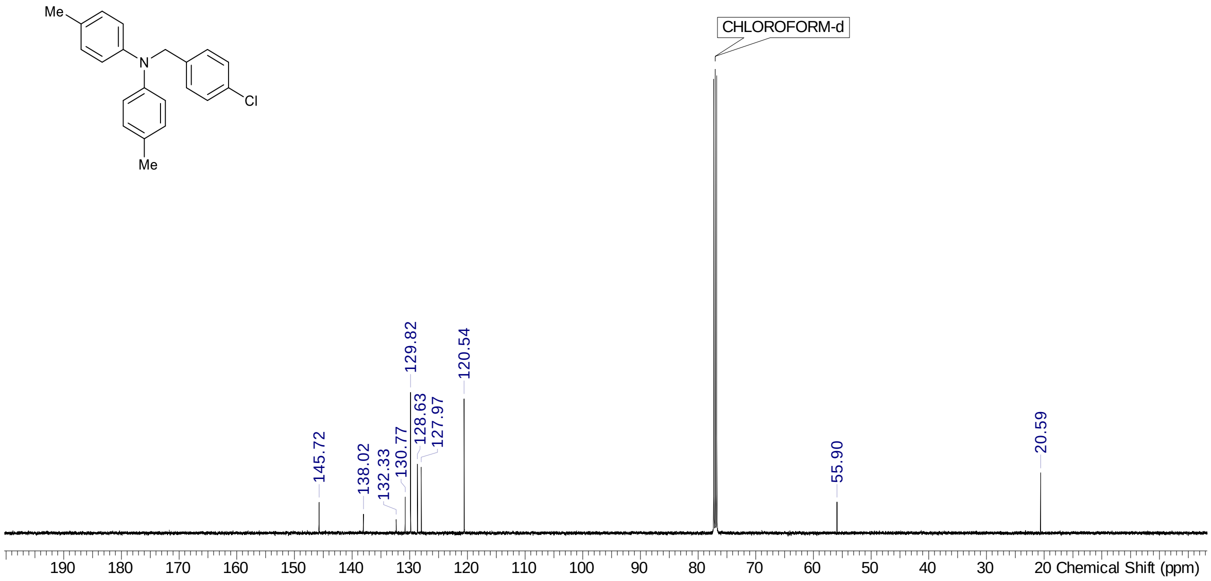

_N_-(4-Bromobenzyl)-4-methyl-_N_-(_p_-tolyl)aniline, 1f

^{1}H NMR (400 MHz, $CDCl_3$)

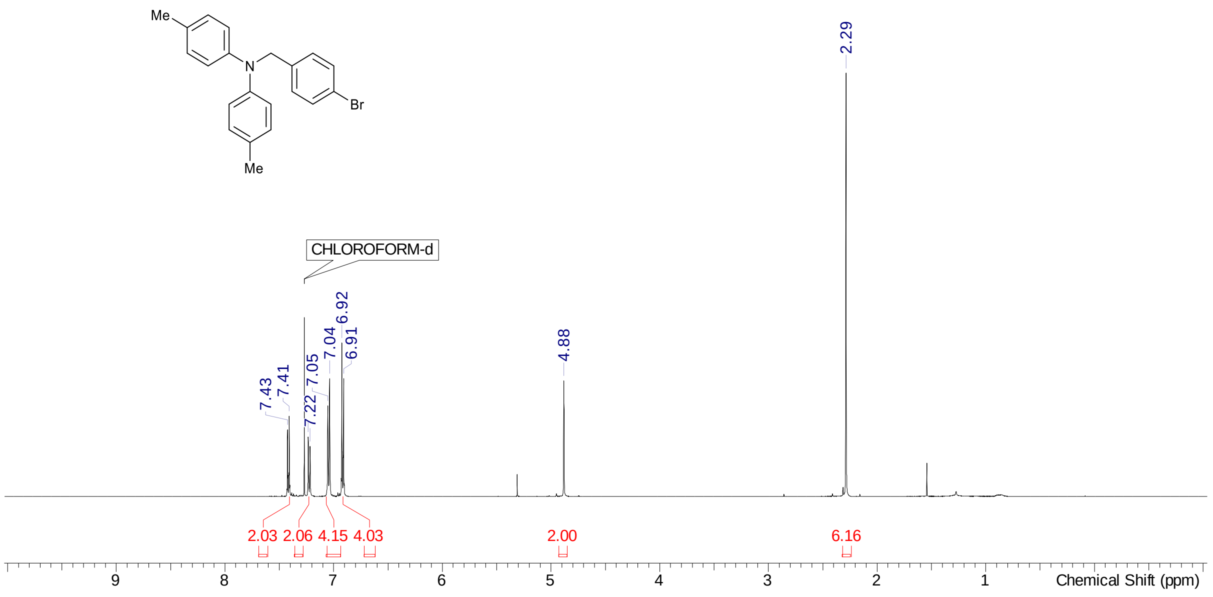

$^{13}C\{^{1}H\}$ NMR ($CDCl_3$, 101 MHz)

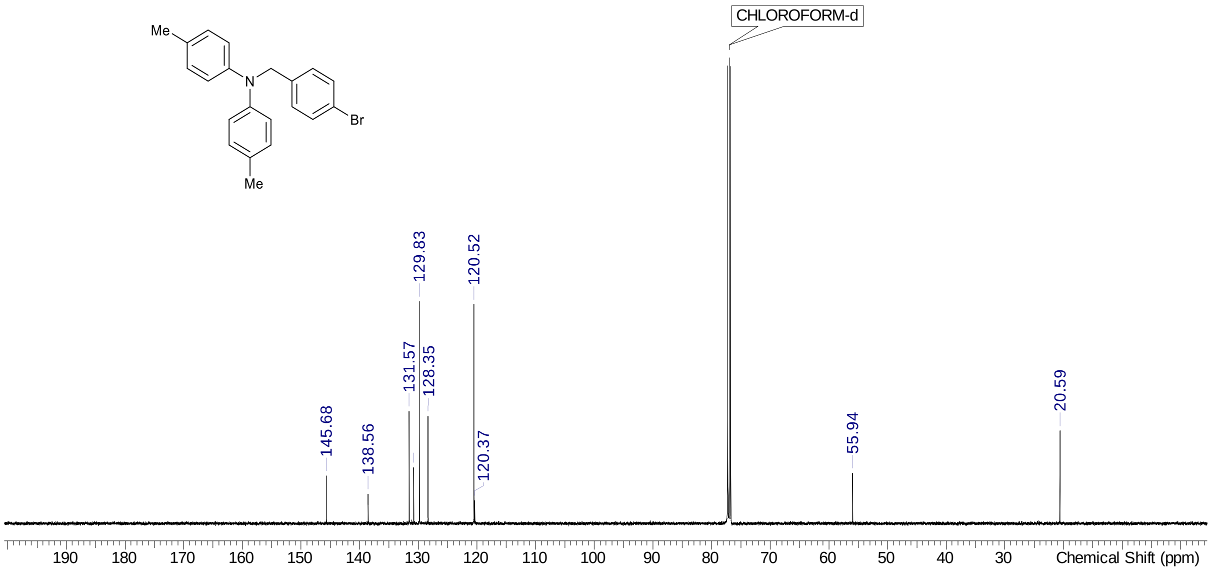

4-Methyl-*N*-(4-(methylthio)benzyl)-*N*-(*p*-tolyl)aniline, 1g

^{1}H NMR (400 MHz, $CDCl_3$)

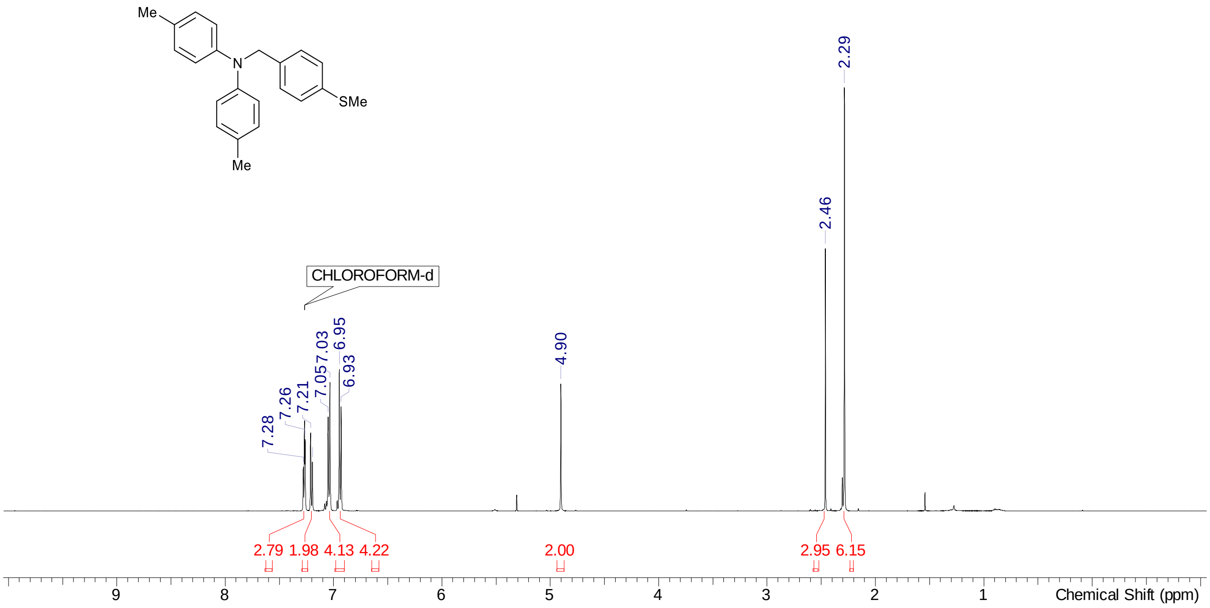

$^{13}C\{^{1}H\}$ NMR ($CDCl_3$, 101 MHz)

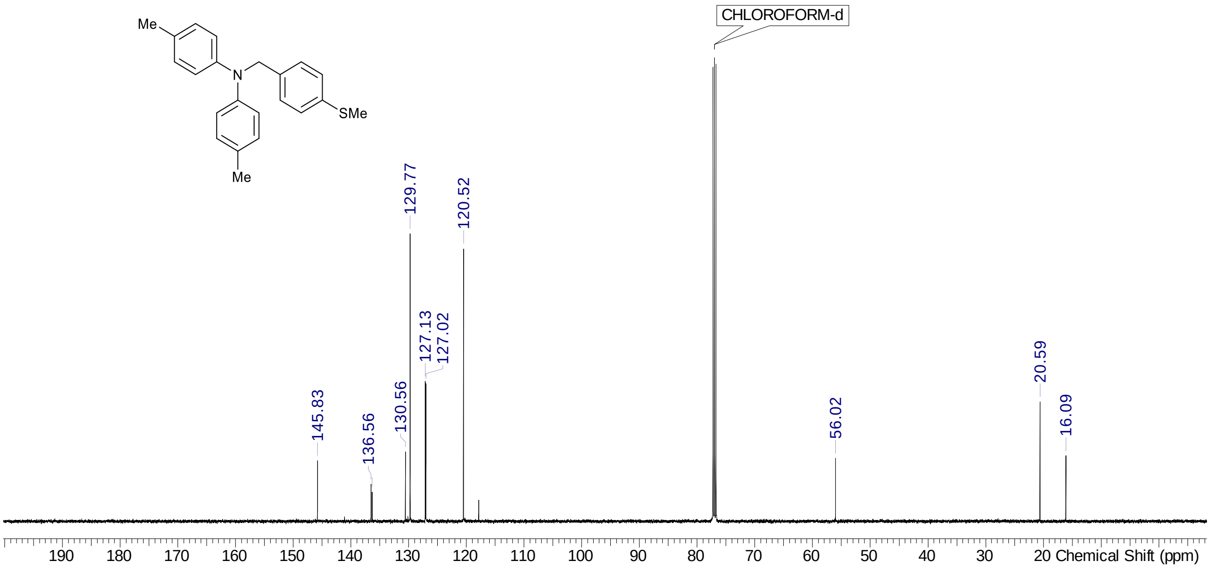

N,4-Dibenzhydryl-N-methylaniline, 1u

^{1}H NMR (400 MHz, $CDCl_3$)

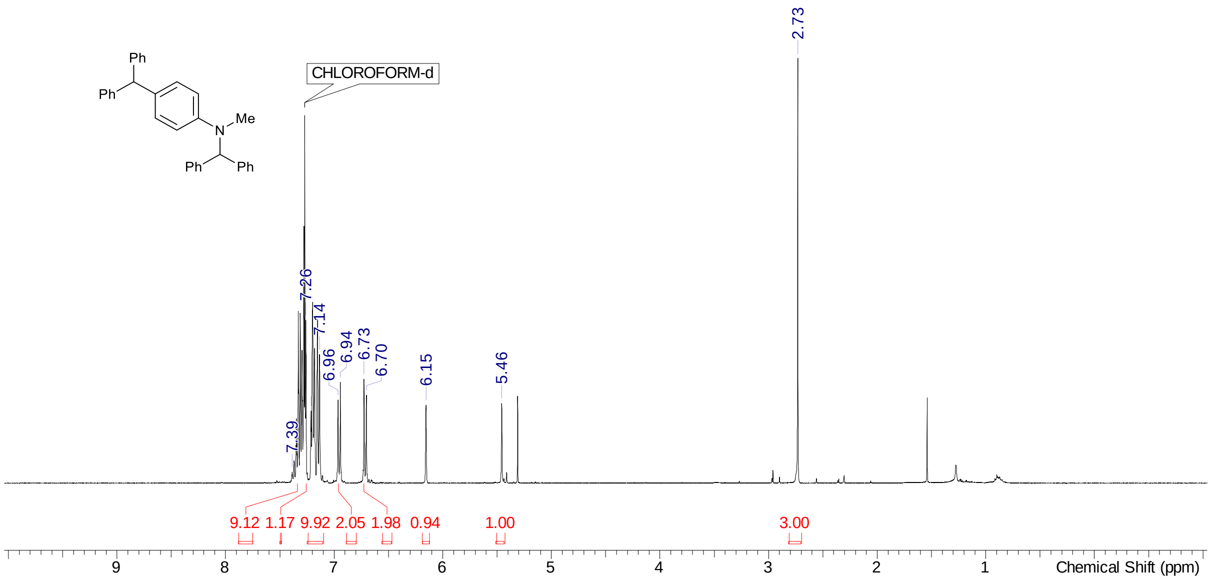

^{13}C{^{1}H} NMR (101 MHz, $CDCl_3$)

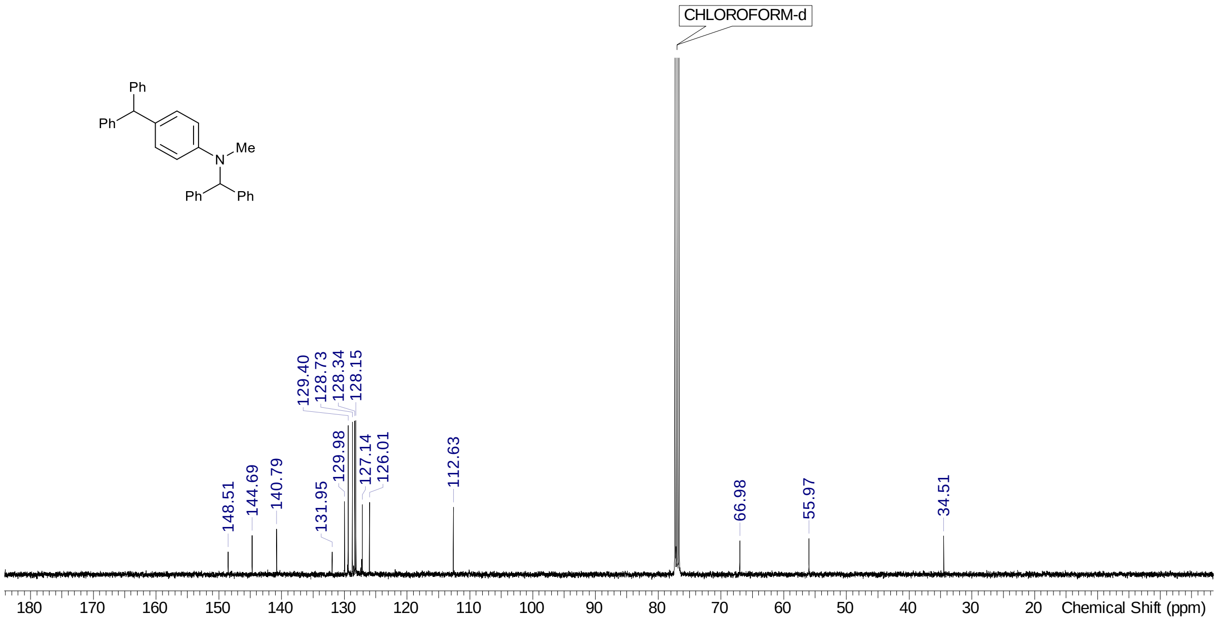

***N*-Benzhydryl-*N*,4-dimethylaniline, 1t**

^{1}H NMR (400 MHz, $CDCl_3$)

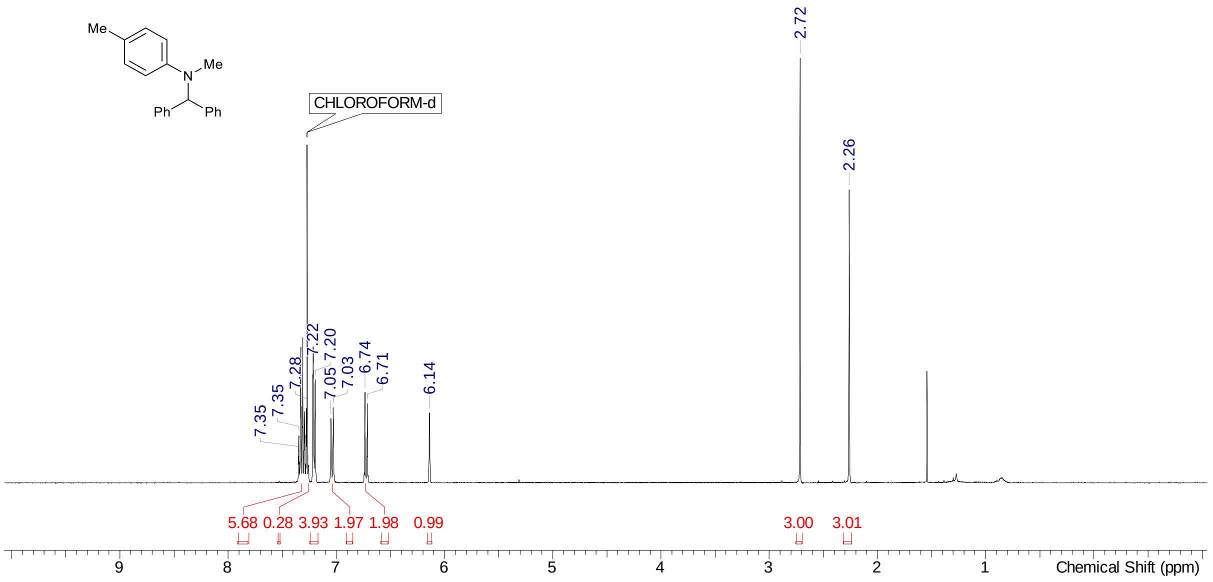

$^{13}C\{^{1}H\}$ NMR (101 MHz, $CDCl_3$)

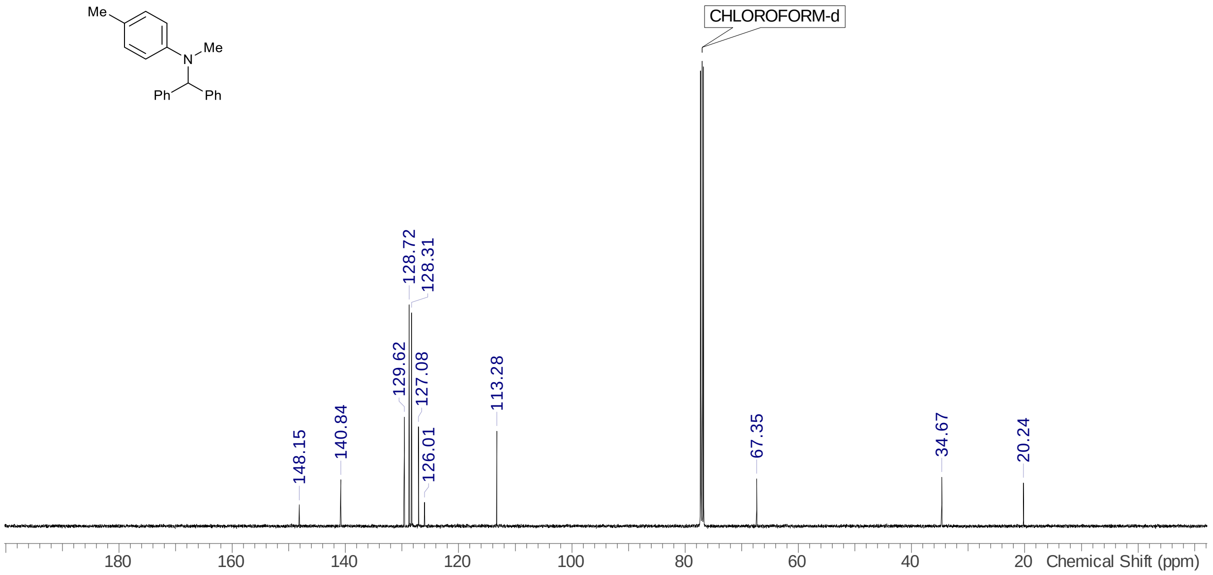

^{1}H, $^{13}C\{^{1}H\}$-HSQC NMR ($CDCl_3$, 400, 101 MHz)

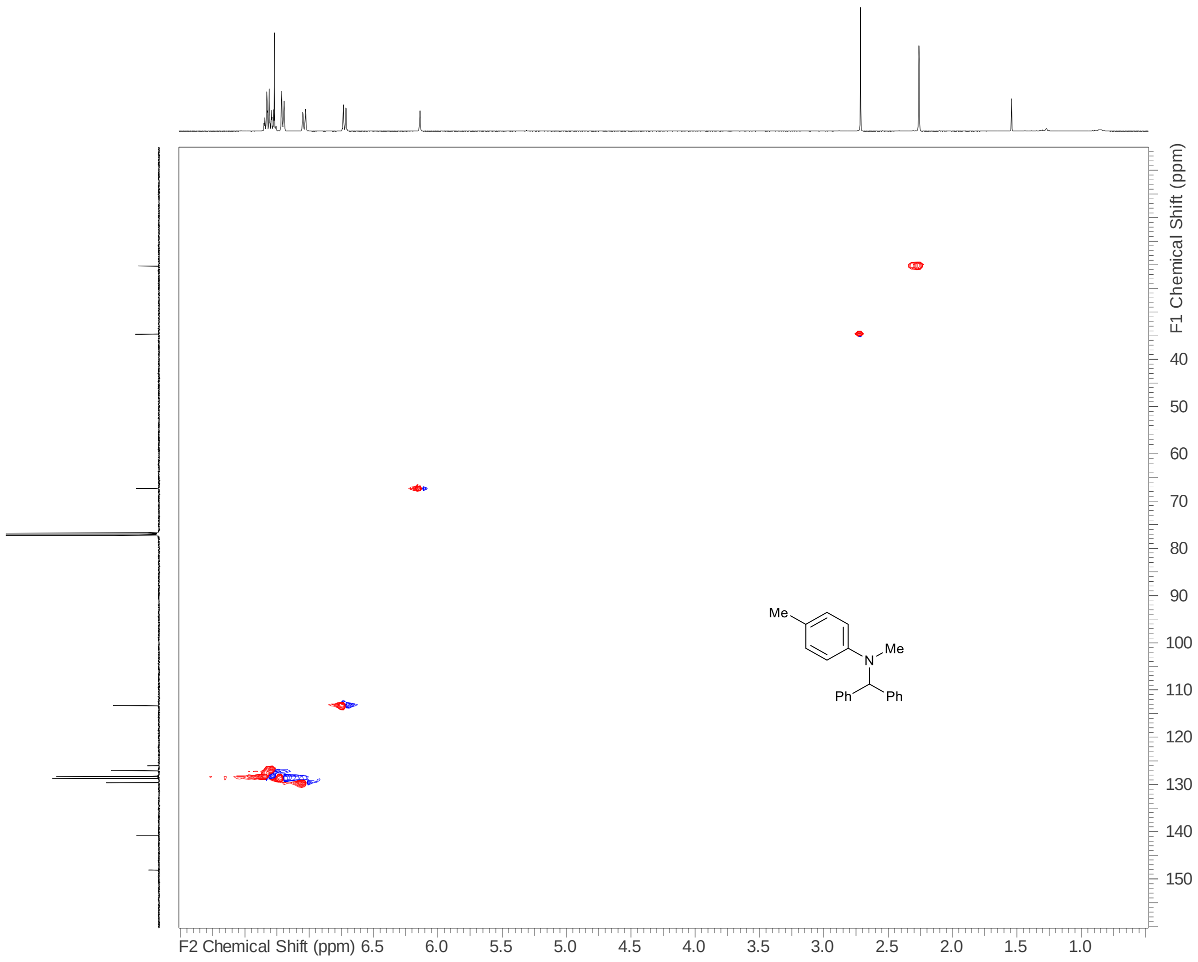

_N_-Ethyl-4-methyl-_N_-(_p_-tolyl)aniline, 1v

^{1}H NMR (400 MHz, $CDCl_3$)

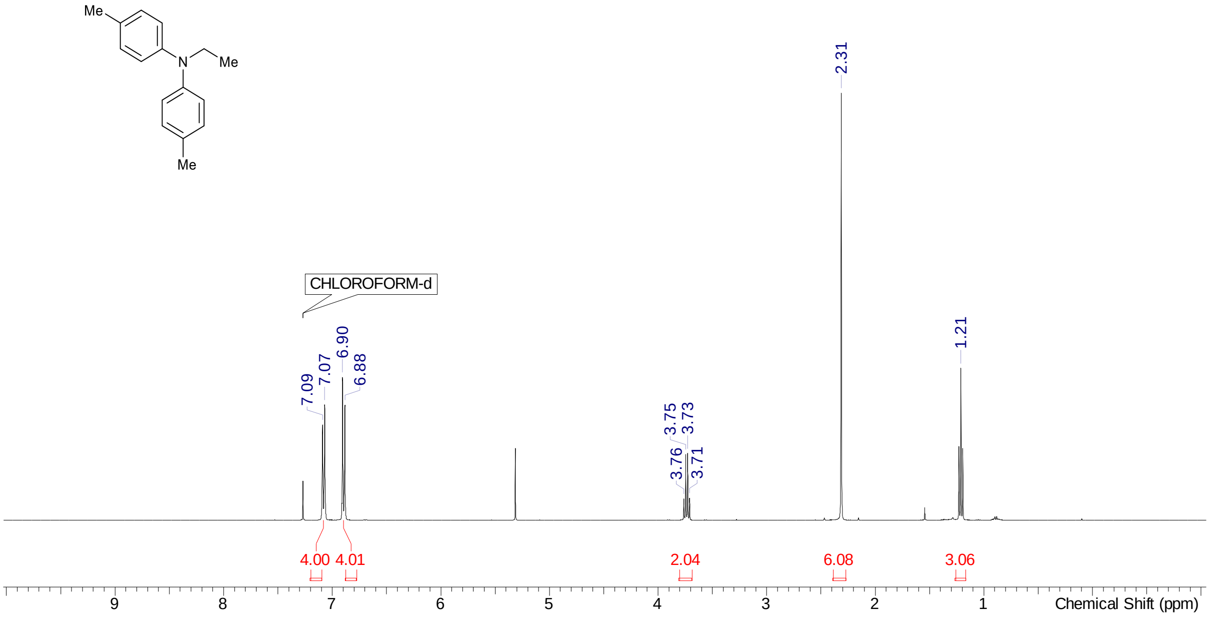

$^{13}C\{^1H\}$ NMR ($CDCl_3$, 101 MHz)

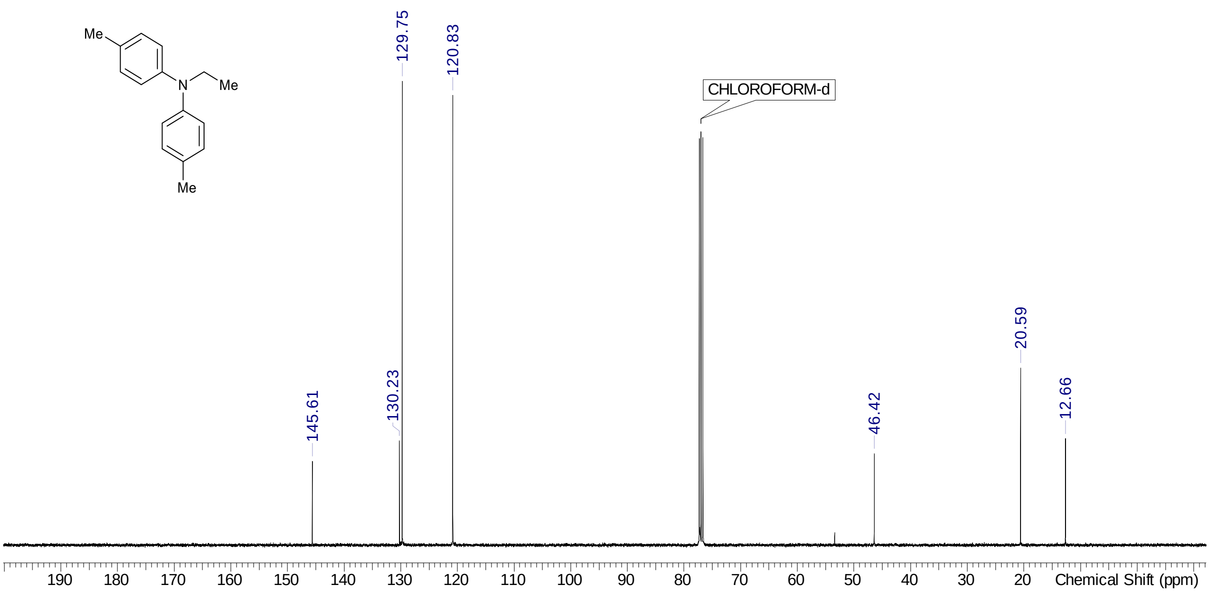

^{1}H, $^{13}C\{^{1}H\}$-HSQC NMR ($CDCl_3$, 400, 101 MHz)

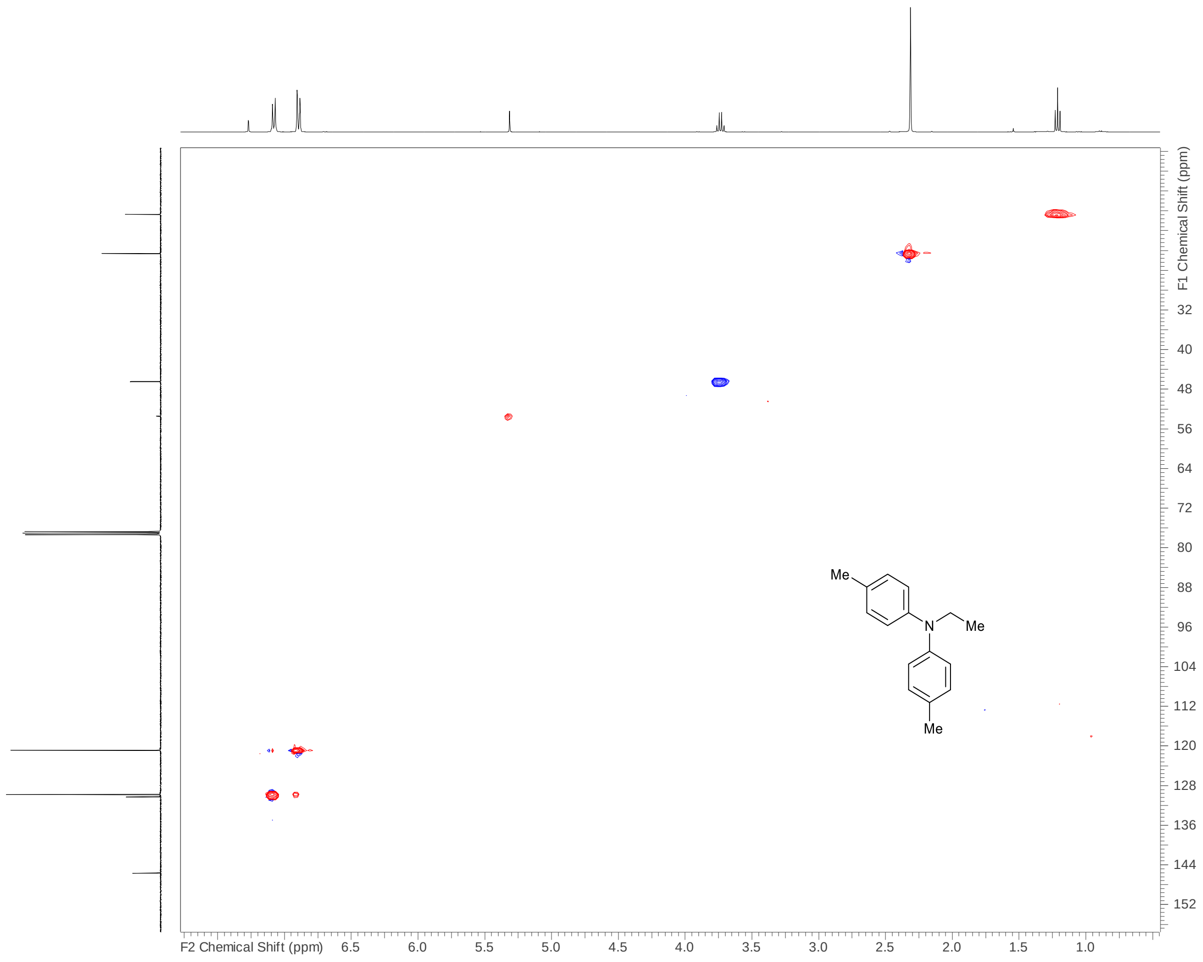

***N*-Butyl-4-methyl-*N*-(*p*-tolyl)aniline, 1w**

^{1}H NMR (400 MHz, $CDCl_3$)

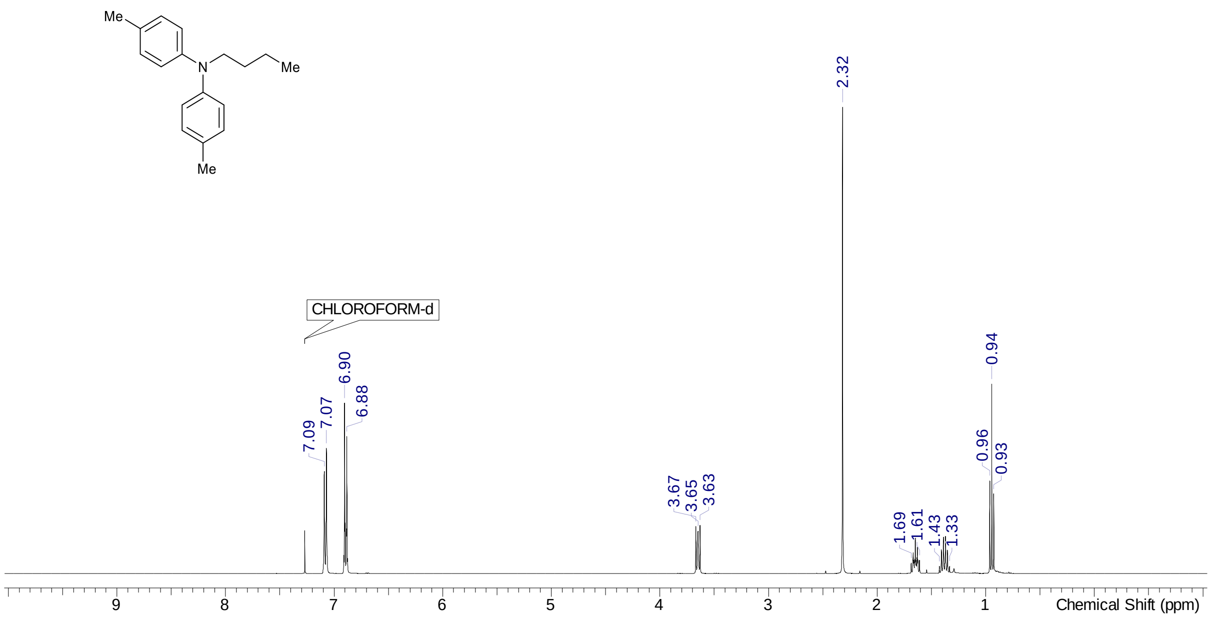

^{13}C{^{1}H} NMR ($CDCl_3$, 101 MHz)

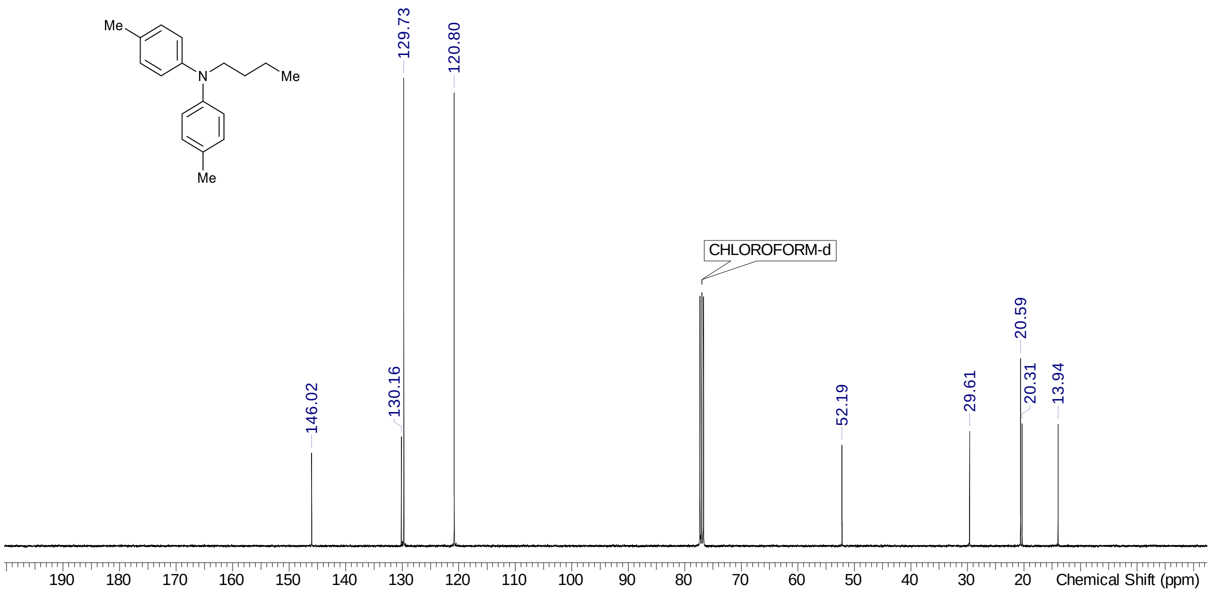

N-Benzhydryl-4-(4-chloro-3,5-dimethylphenoxy)-N-methylaniline, 1ab

^{1}H NMR (500 MHz, $CDCl_3$)

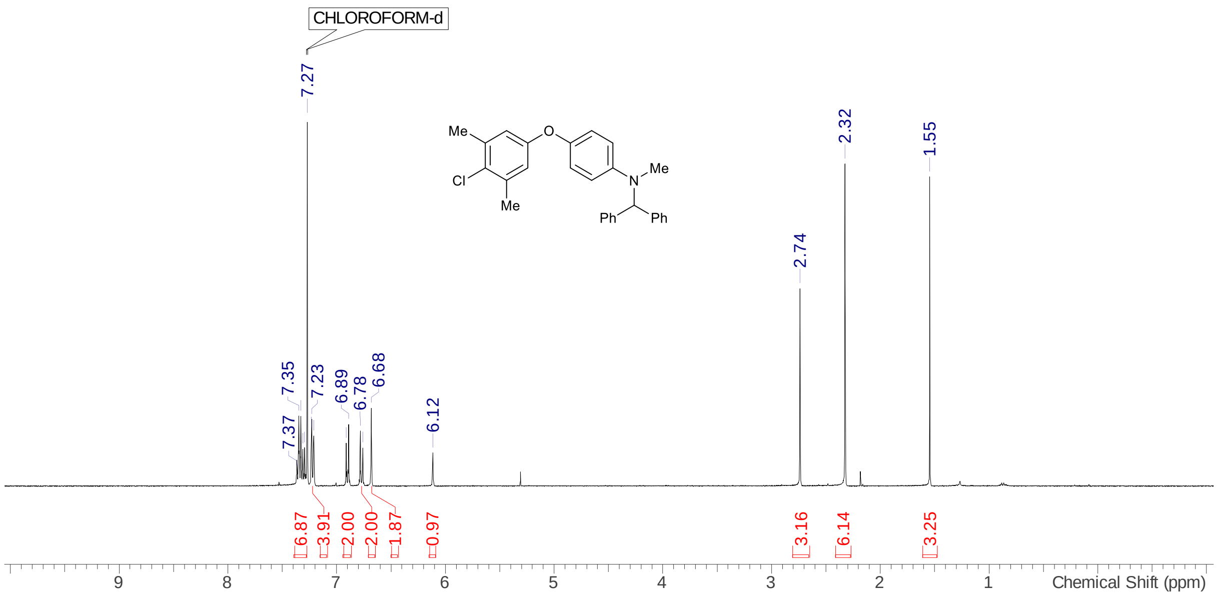

$^{13}C\{^{1}H\}$ NMR ($CDCl_3$, 126 MHz)

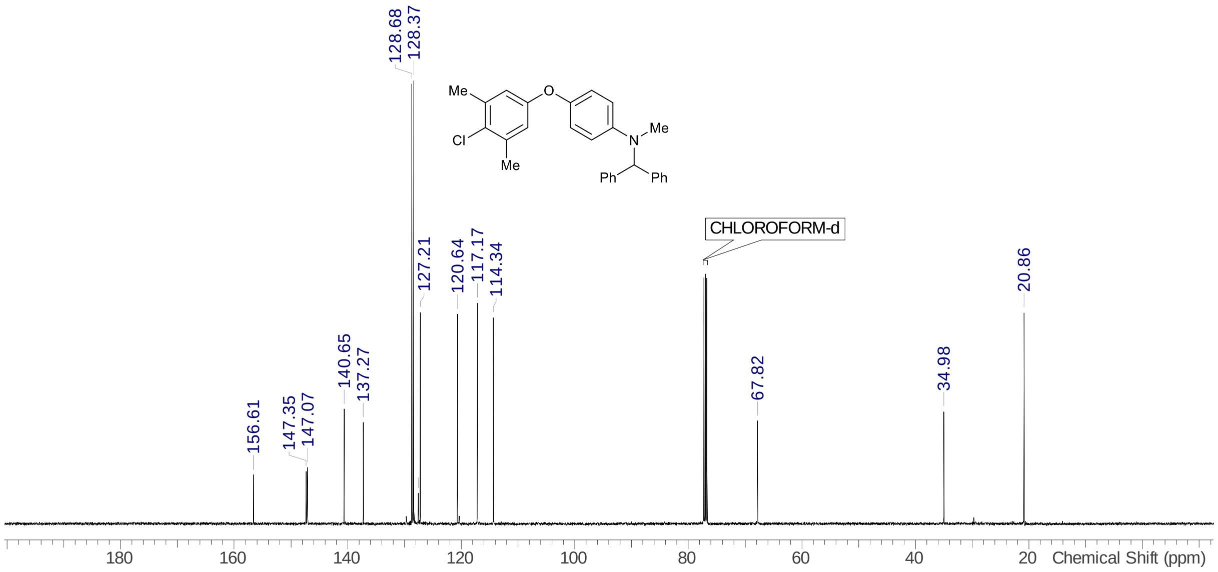

(1*S*,4*S*)-4-(3,4-Dichlorophenyl)-*N*-methyl-*N*-(*p*-tolyl)-1,2,3,4-tetrahydronaphthalen-1-amine, 1ac

^{1}H NMR (400 MHz, $CDCl_3$)

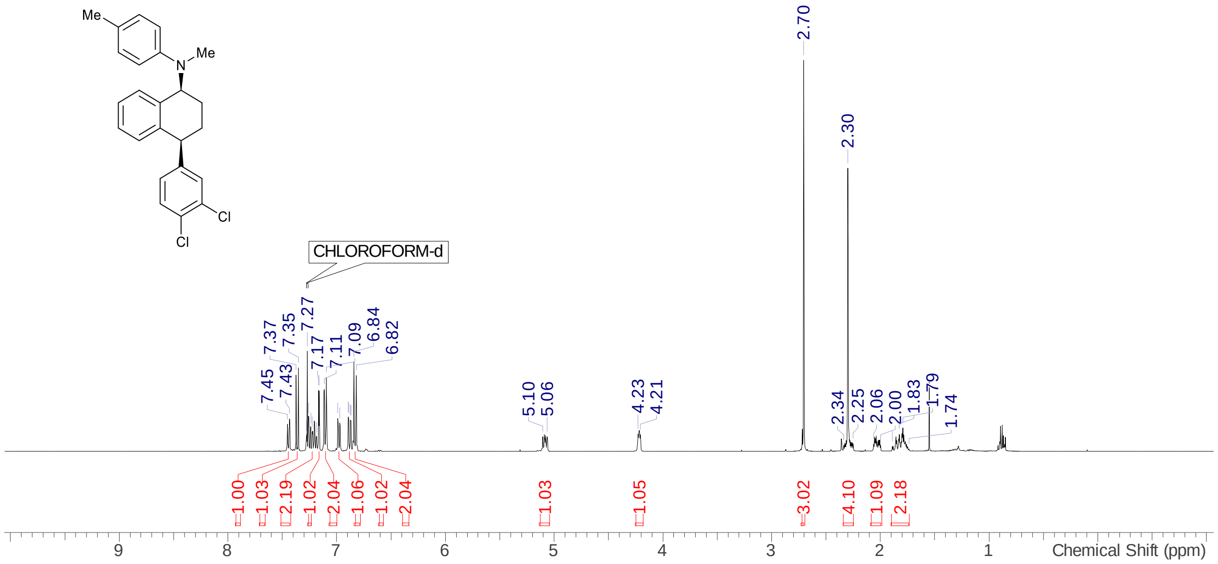

^{13}C{^{1}H} NMR ($CDCl_3$, 101 MHz)

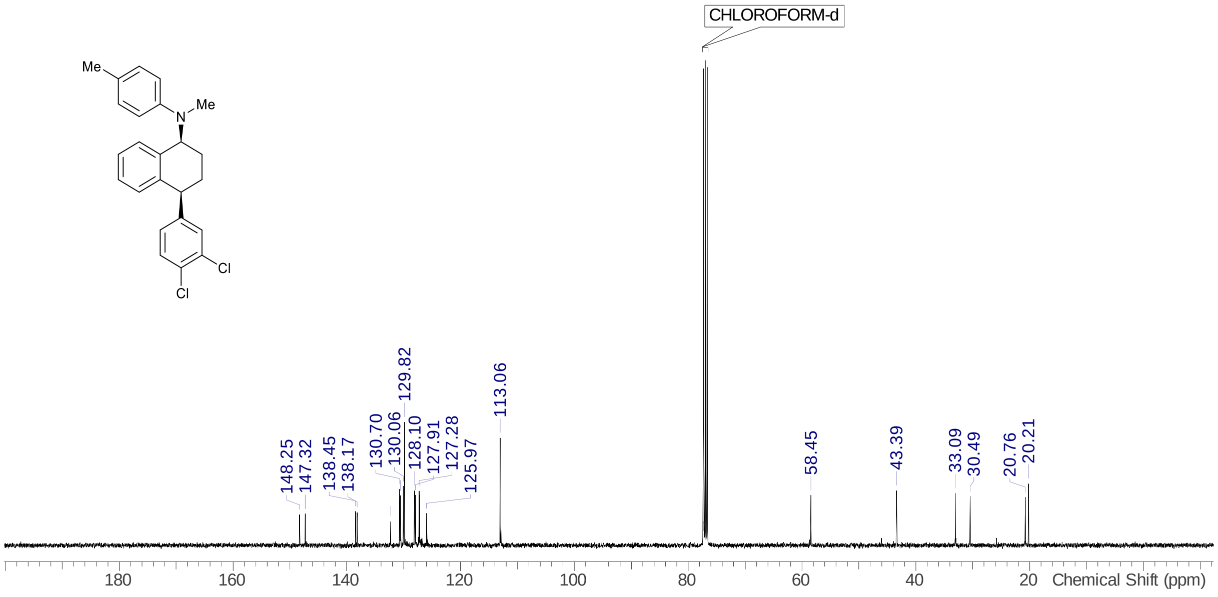

^{1}H, ^{13}C{^{1}H}-HSQC NMR ($CDCl_3$, 400, 101 MHz)

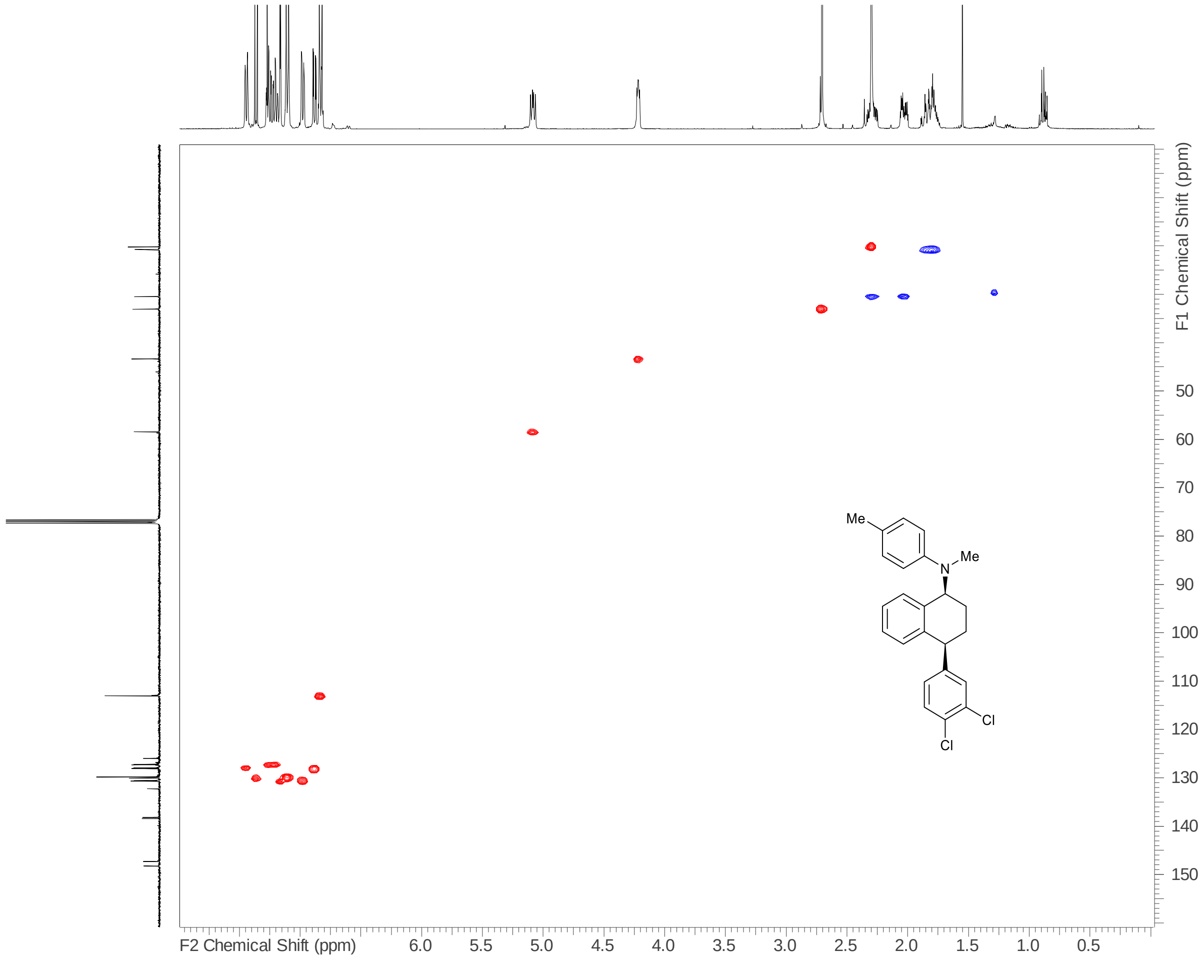

_N,N_-Dimethyl-2-phenoxyaniline, 1ag

^{1}H NMR (400 MHz, $CDCl_3$)

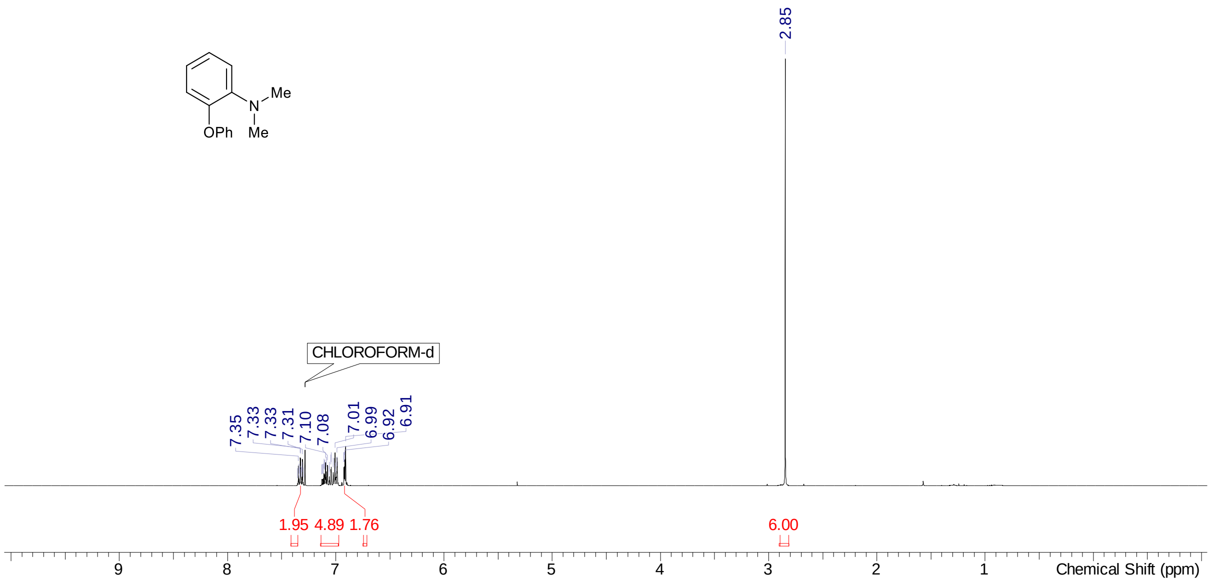

$^{13}C\{^{1}H\}$ NMR ($CDCl_3$, 101 MHz)

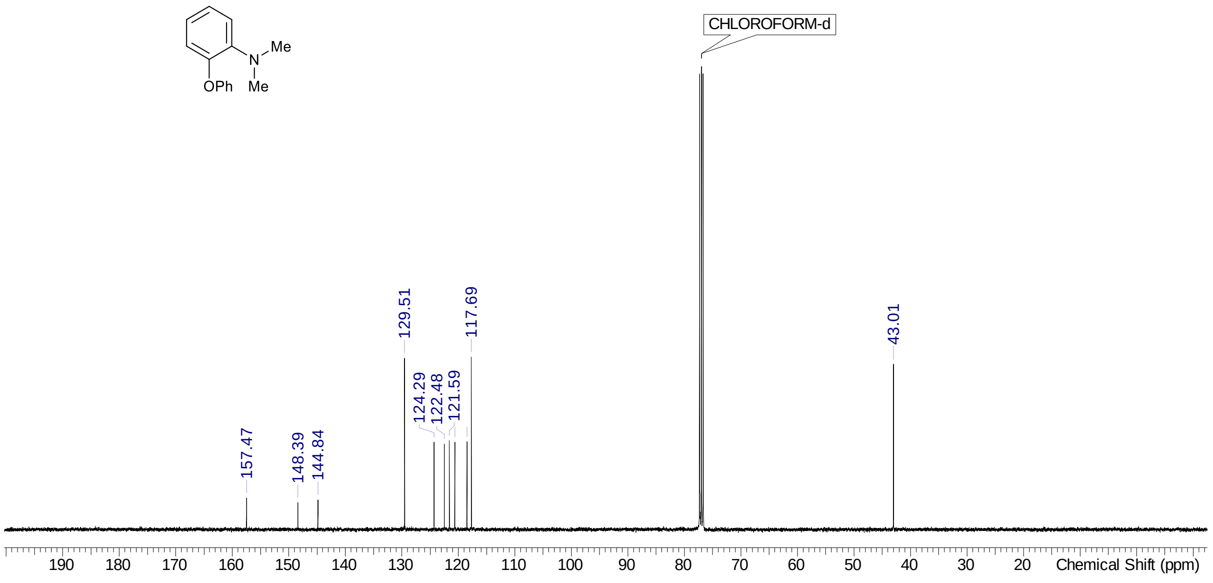

1-Methylene-2,3-dihydro-1*H*-indene, 2n

^{1}H NMR (500 MHz, $CDCl_3$)

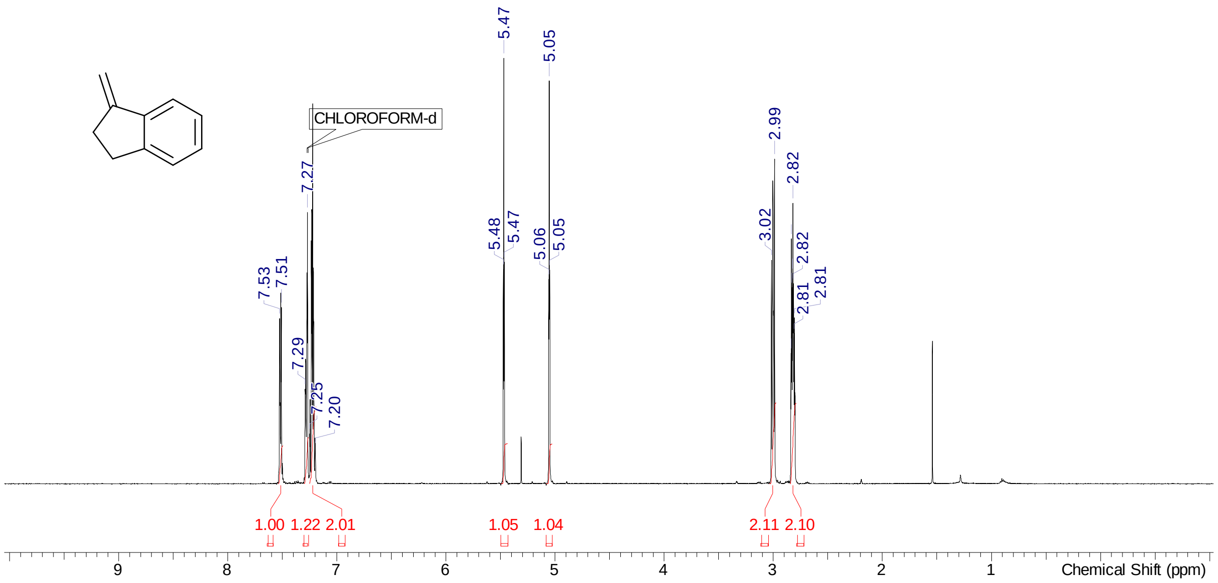

$^{13}C\{^{1}H\}$ NMR (126 MHz, $CDCl_3$)

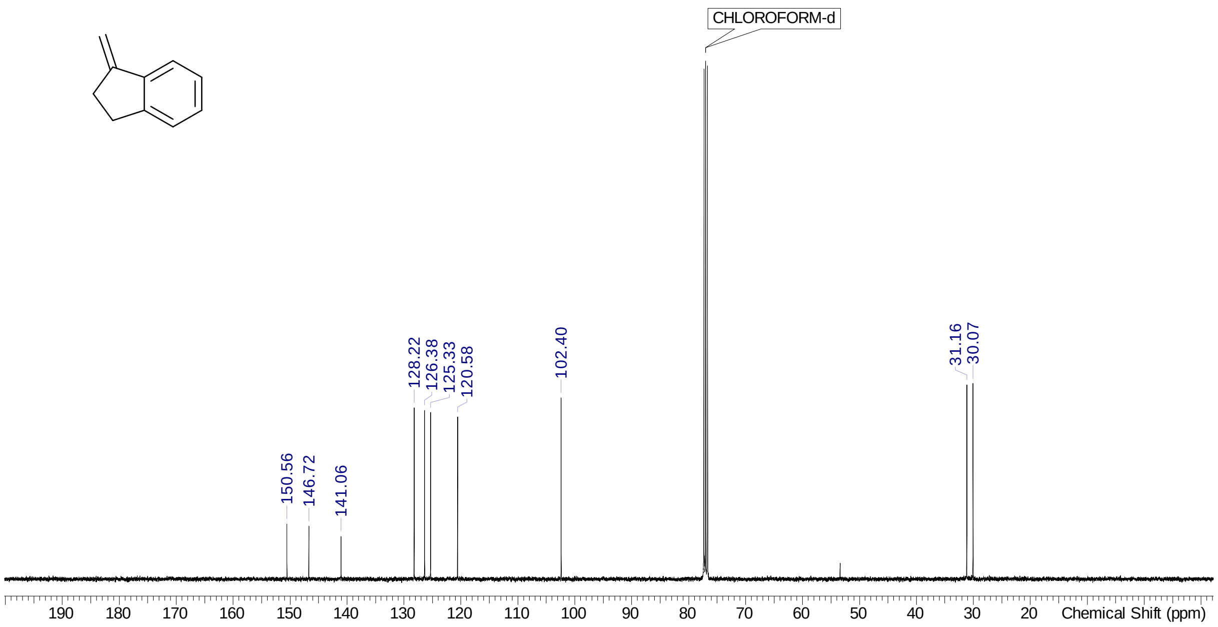

^{1}H, ^{13}C{^{1}H}-HSQC NMR ($CDCl_3$, 500, 126 MHz)

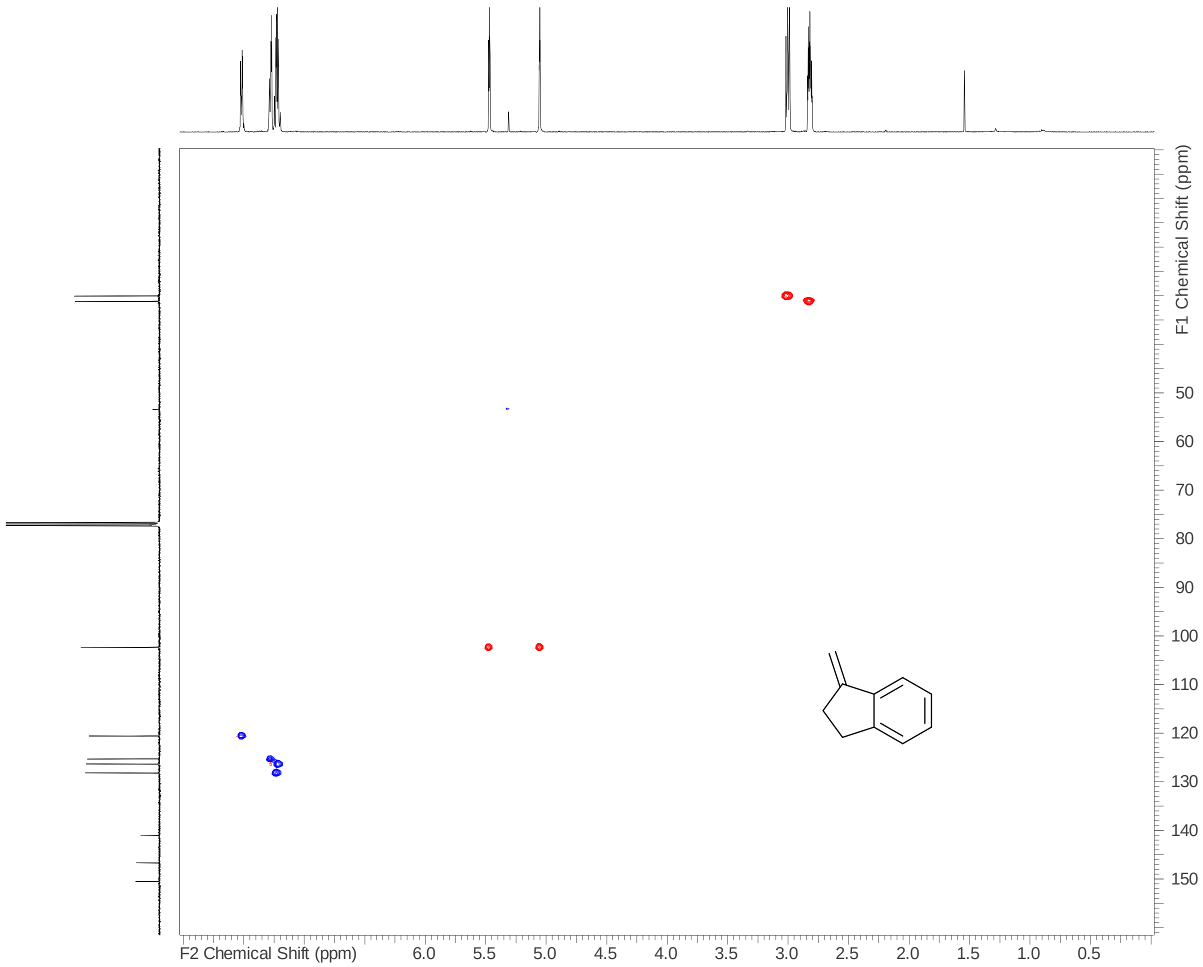

4,6-Dimethyl-1-(*p*-tolyl)-4-((trimethylsilyl)methyl)-1,2,3,4-tetrahydroquinoline, 3a

^{1}H NMR (500 MHz, $CDCl_3$)

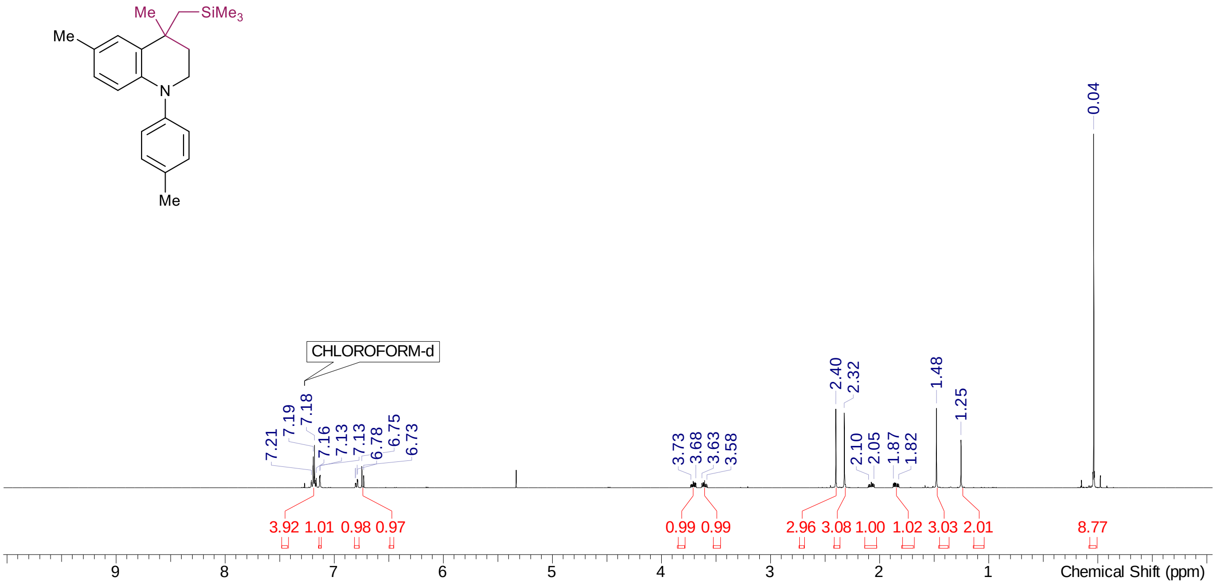

^{13}C{^{1}H} NMR ($CDCl_3$, 126 MHz)

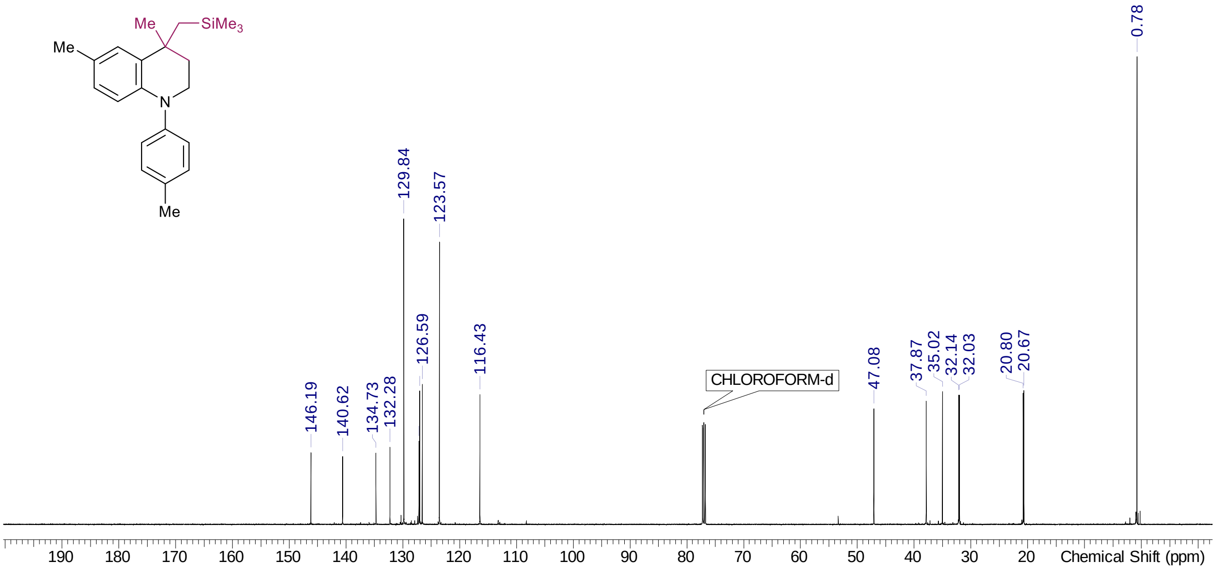

^{1}H, $^{13}C\{^{1}H\}$-HSQC NMR ($CDCl_3$, 500, 126 MHz)

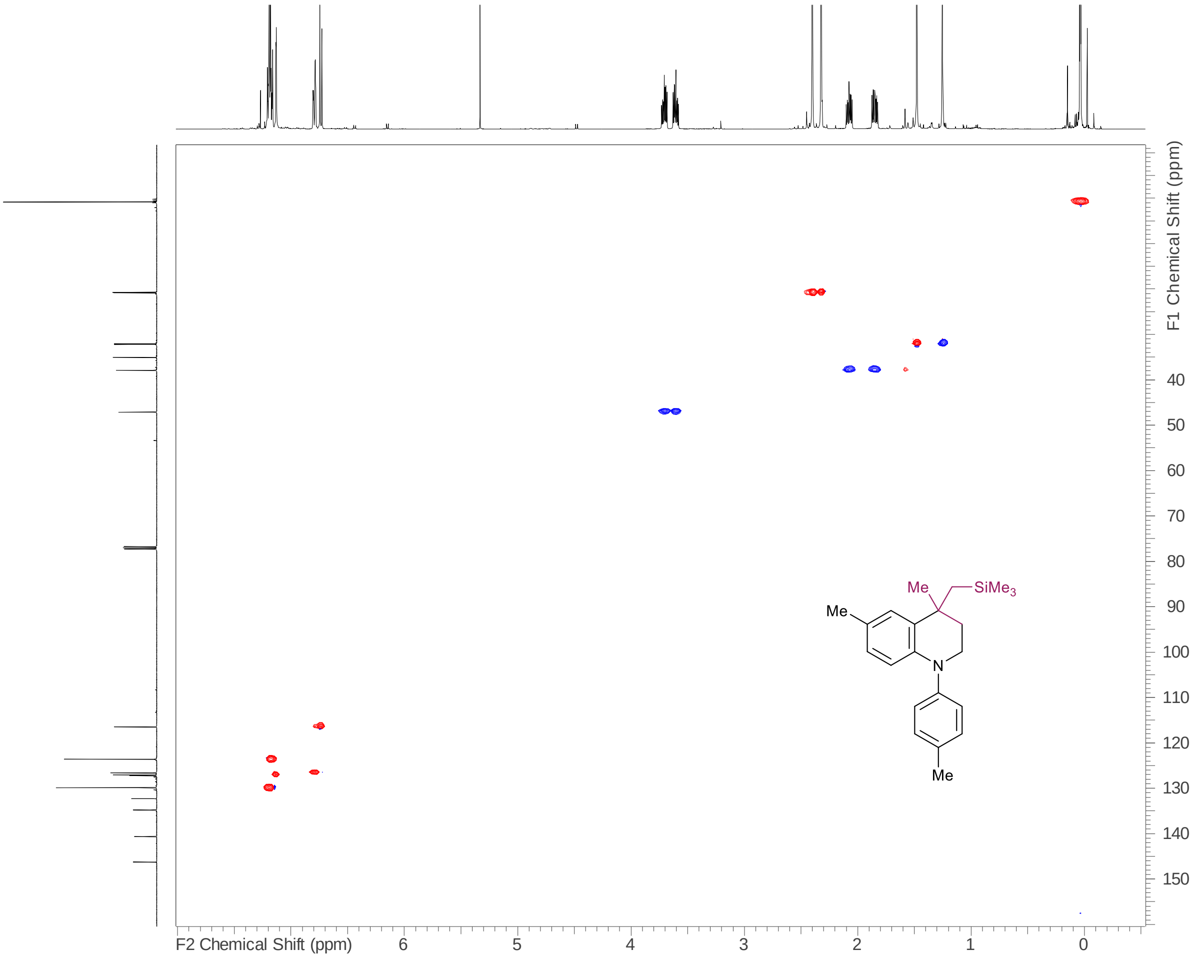

4,6-Dimethyl-1-(*p*-tolyl)-4-((trimethylsilyl)methyl)-1,4-dihydroquinoline, 3a'

^{1}H NMR (400 MHz, $CDCl_3$)

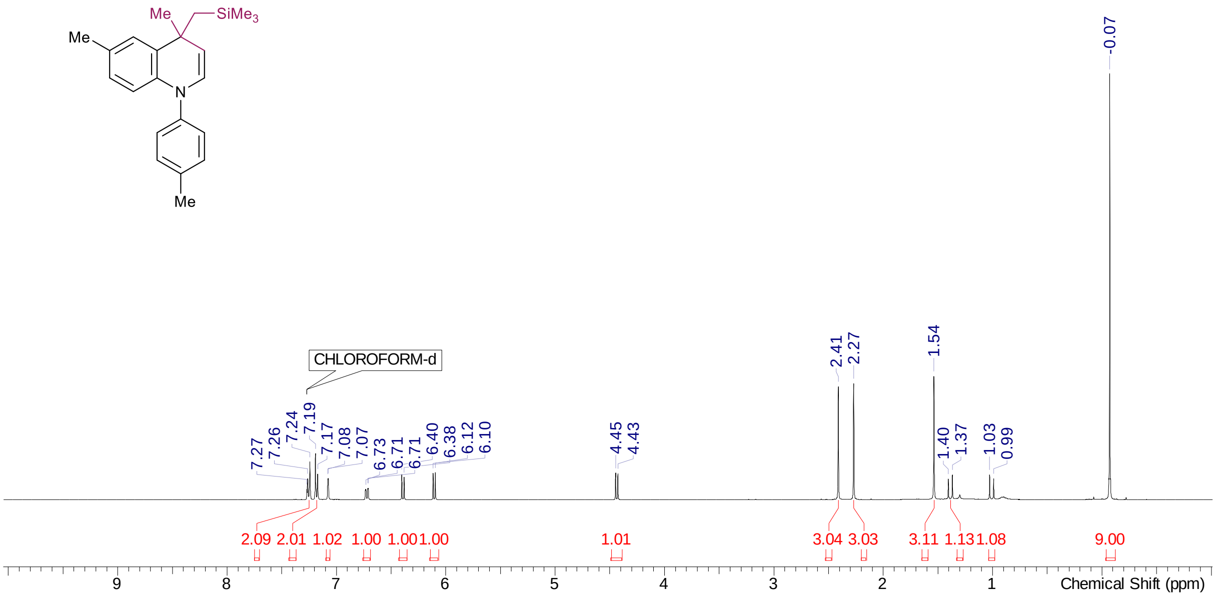

$^{13}C\{^{1}H\}$ NMR ($CDCl_3$, 101 MHz)

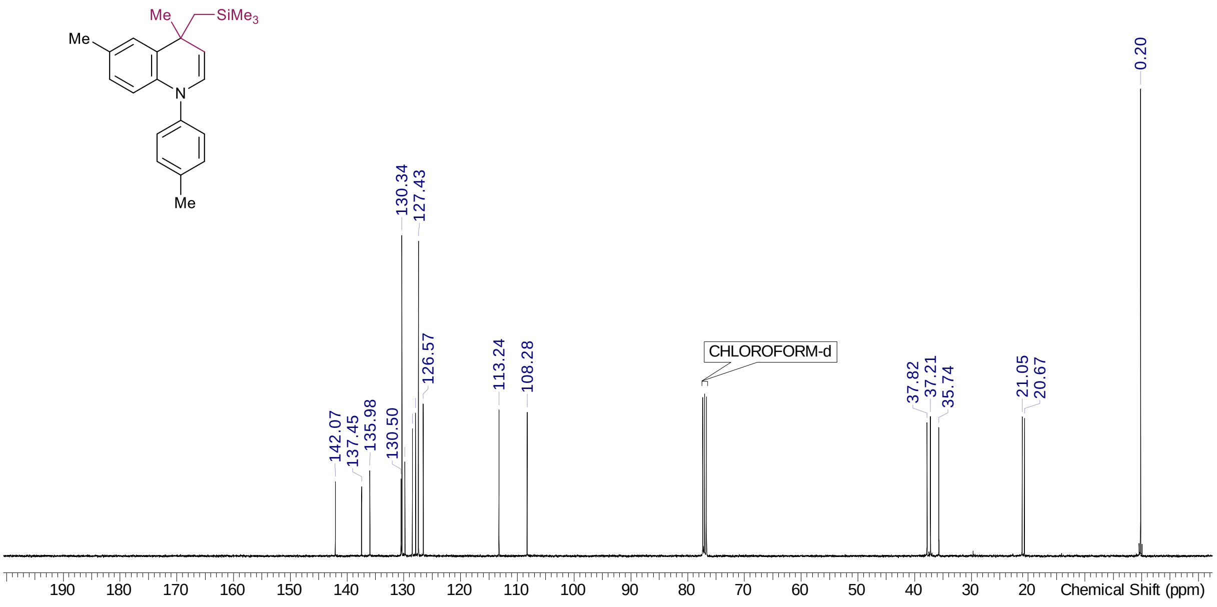

^{1}H, ^{13}C{^{1}H}-HSQC NMR ($CDCl_3$, 400, 101 MHz)

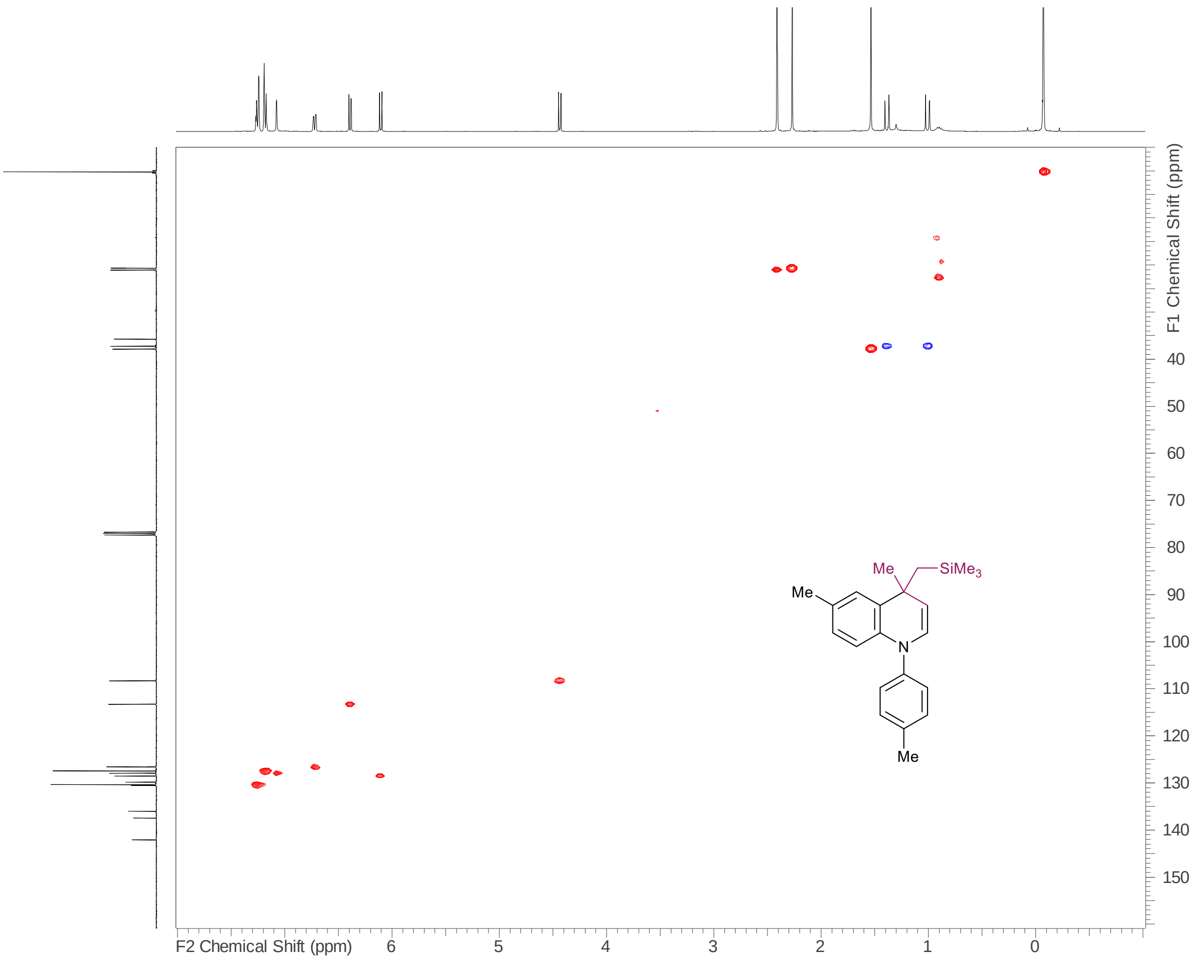

1-Mesityl-4-methyl-4-((trimethylsilyl)methyl)-1,2,3,4-tetrahydroquinoline, 3b

^{1}H NMR (400 MHz, $CDCl_3$)

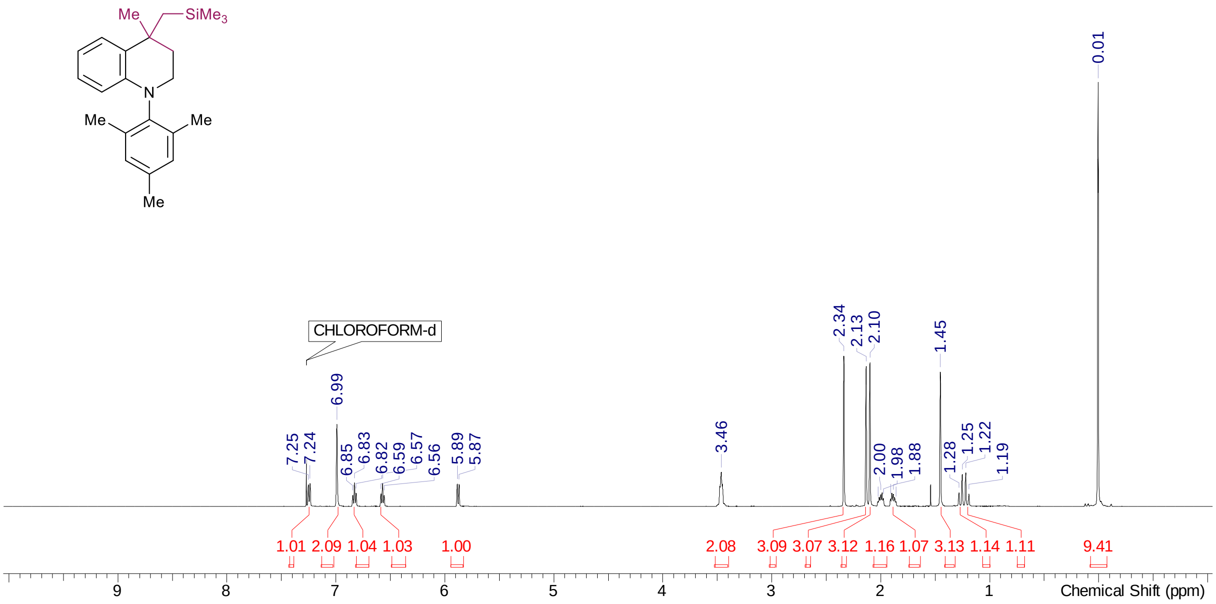

^{13}C{^{1}H} NMR ($CDCl_3$, 101 MHz)

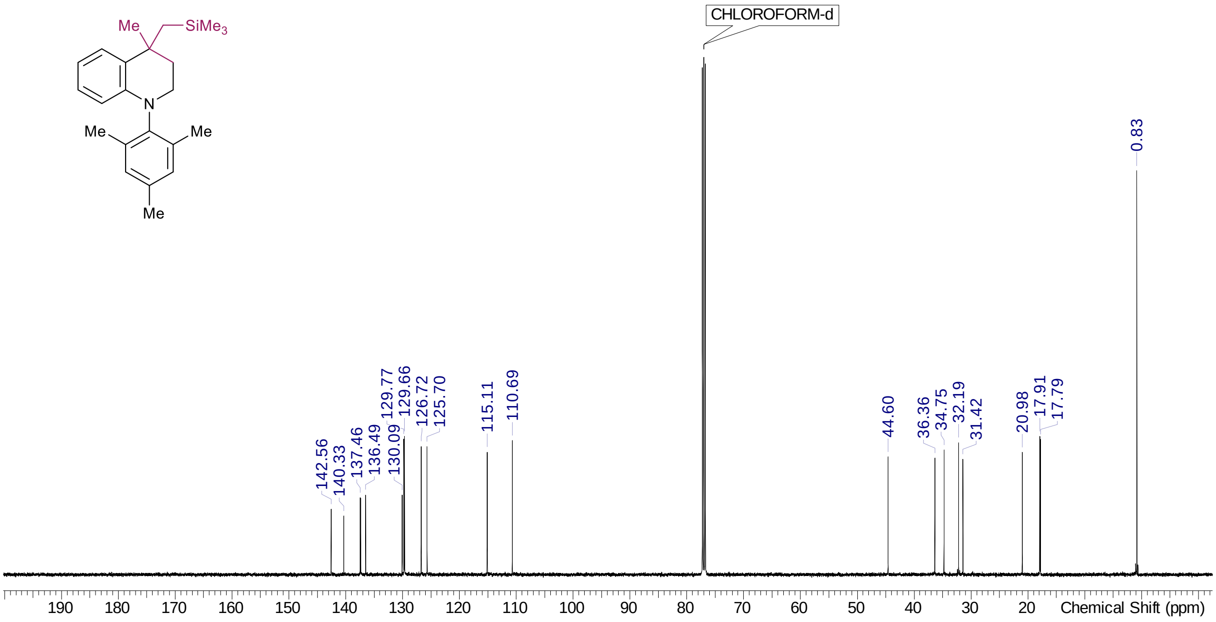

4,6-Dimethyl-2-phenyl-1-(*p*-tolyl)-4-((trimethylsilyl)methyl)-1,2,3,4-tetrahydroquinoline, 3c

1:1.1 *dr*

^{1}H NMR (400 MHz, $CDCl_3$)

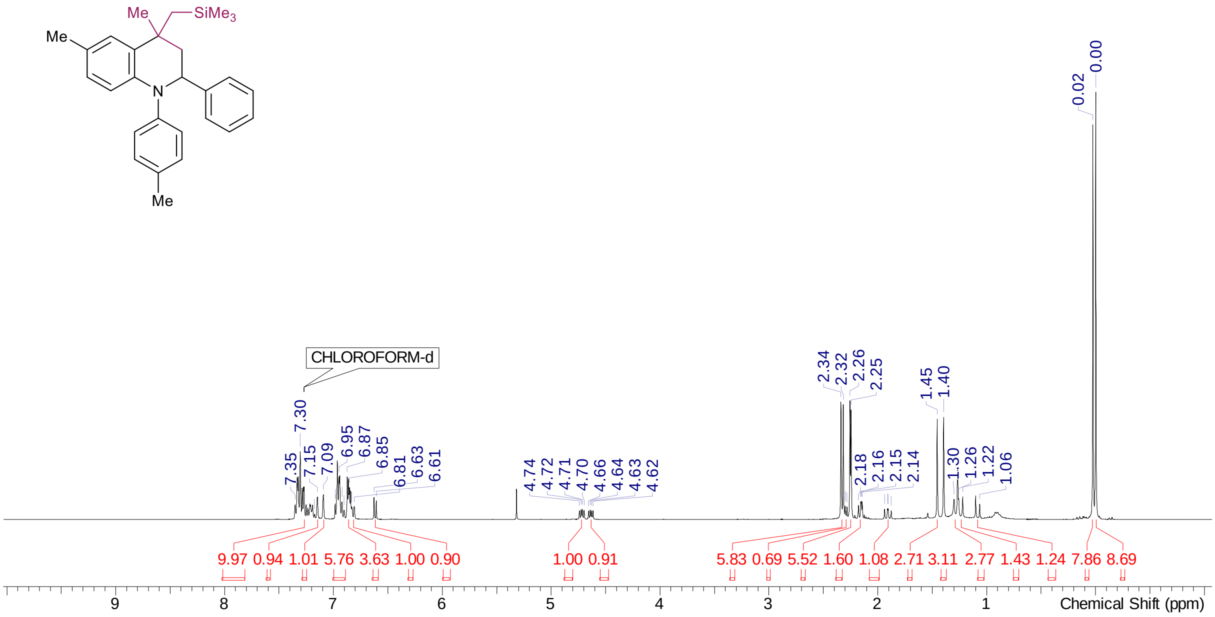

$^{13}C\{^{1}H\}$ NMR ($CDCl_3$, 101 MHz)

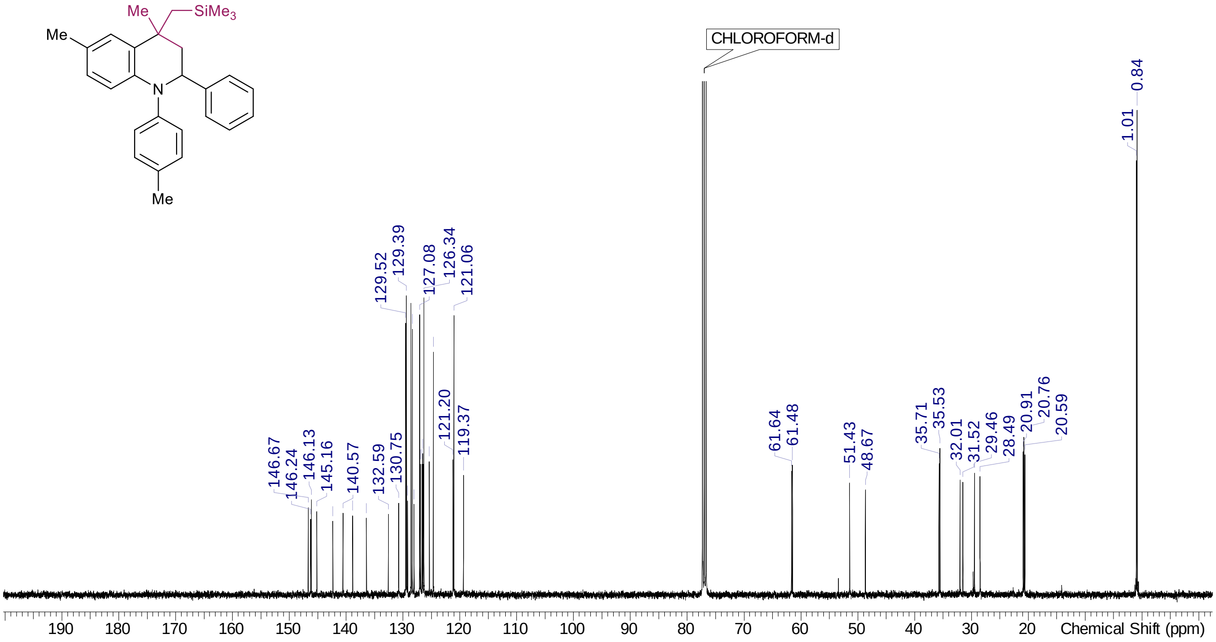

^{1}H, ^{13}C{^{1}H}-HSQC NMR ($CDCl_3$, 400, 101 MHz)

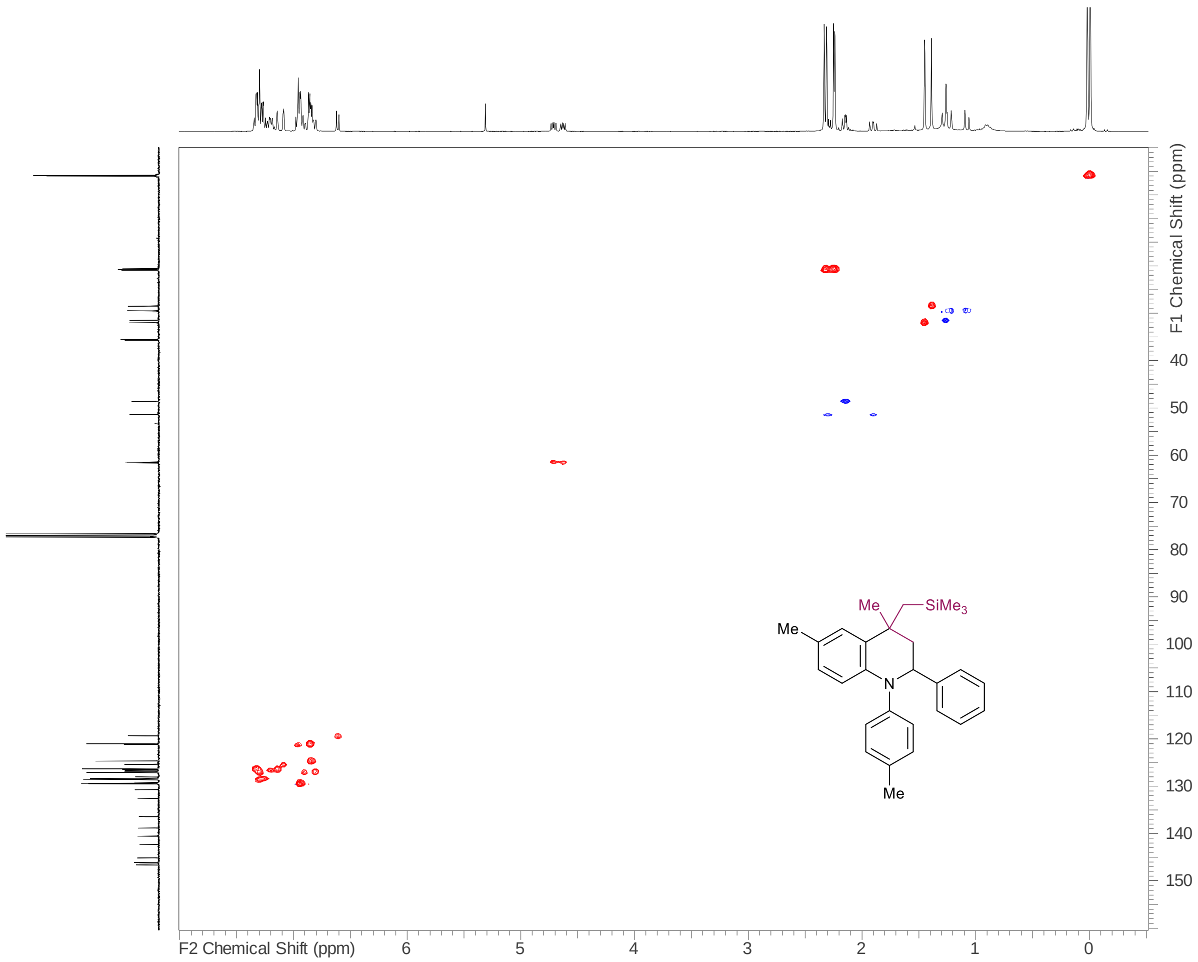

2-(4-Fluorophenyl)-4,6-dimethyl-1-(*p*-tolyl)-4-((trimethylsilyl)methyl)-1,2,3,4-tetrahydroquinoline, 3d 1:1.7 *dr*

^{1}H NMR (400 MHz, $CDCl_3$)

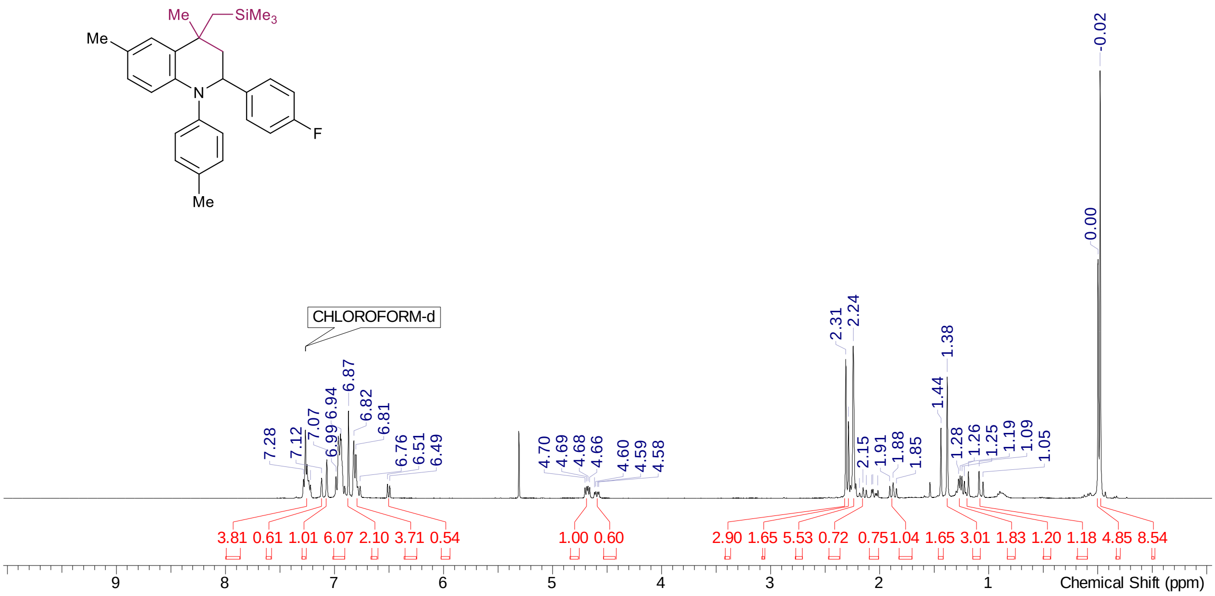

$^{13}C\{^{1}H\}$ NMR ($CDCl_3$, 101 MHz)

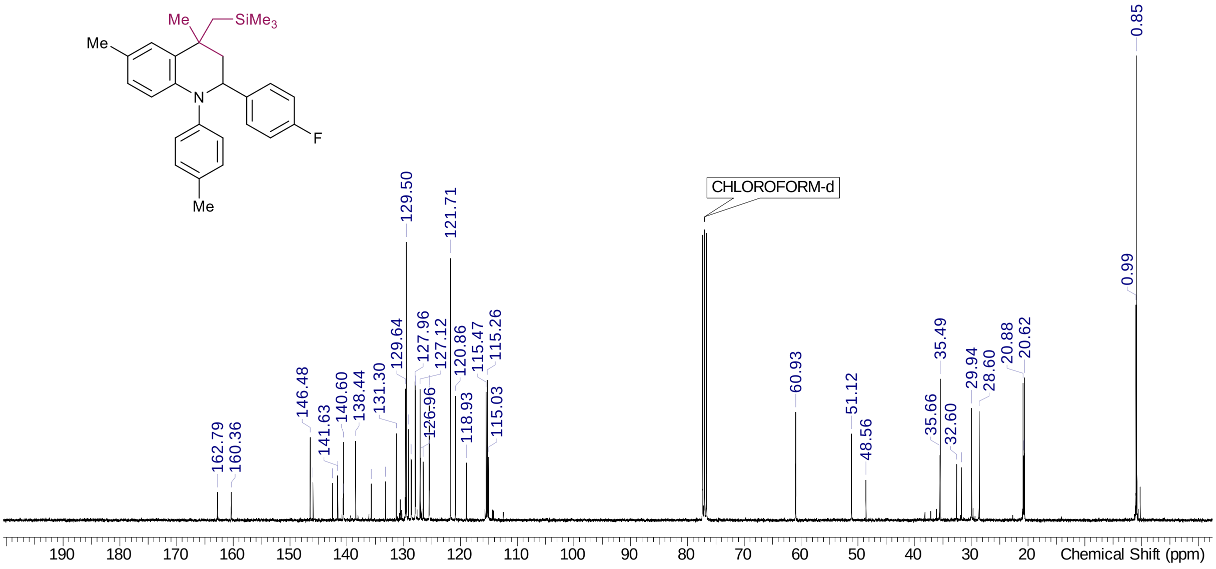

^{1}H, $^{13}C\{^{1}H\}$-HSQC NMR ($CDCl_3$, 400, 101 MHz)

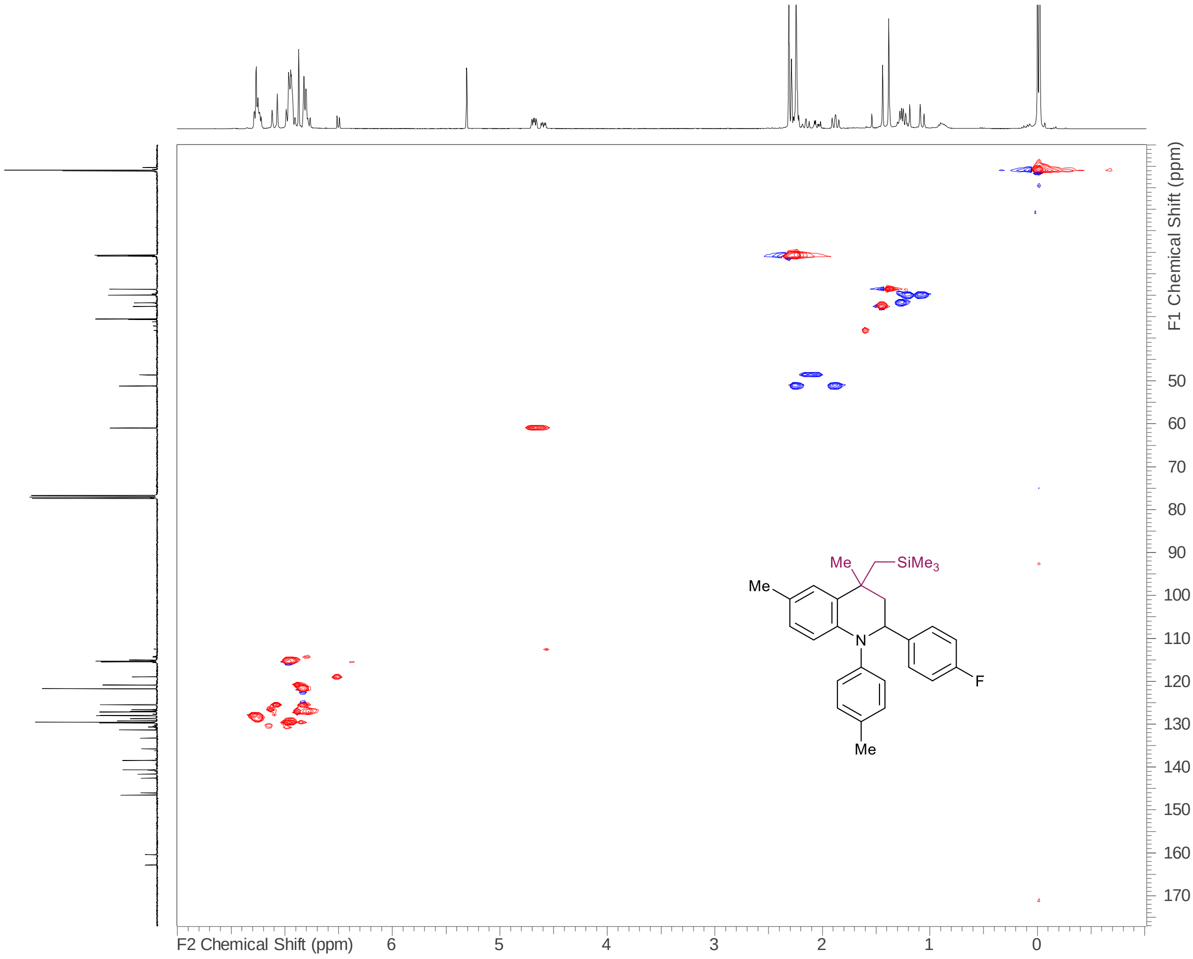

2-(4-Chlorophenyl)-4,6-dimethyl-1-(*p*-tolyl)-4-((trimethylsilyl)methyl)-1,2,3,4-tetrahydroquinoline, 3e 1:1.7 *dr*

^{1}H NMR (400 MHz, $CDCl_3$)

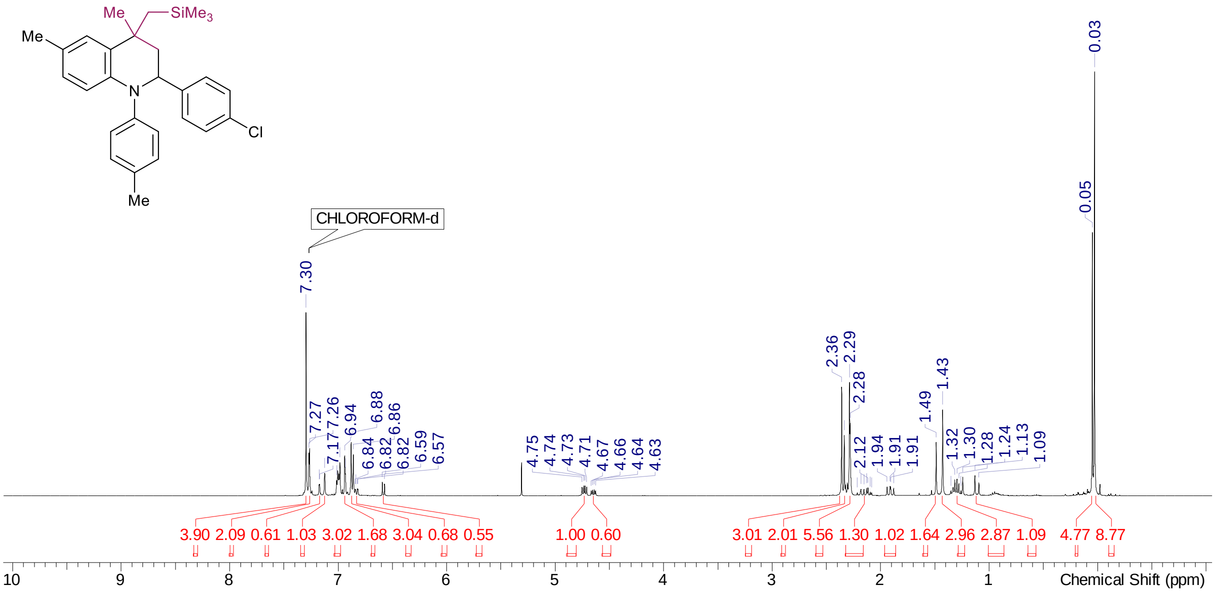

$^{13}C\{^{1}H\}$ NMR ($CDCl_3$, 101 MHz)

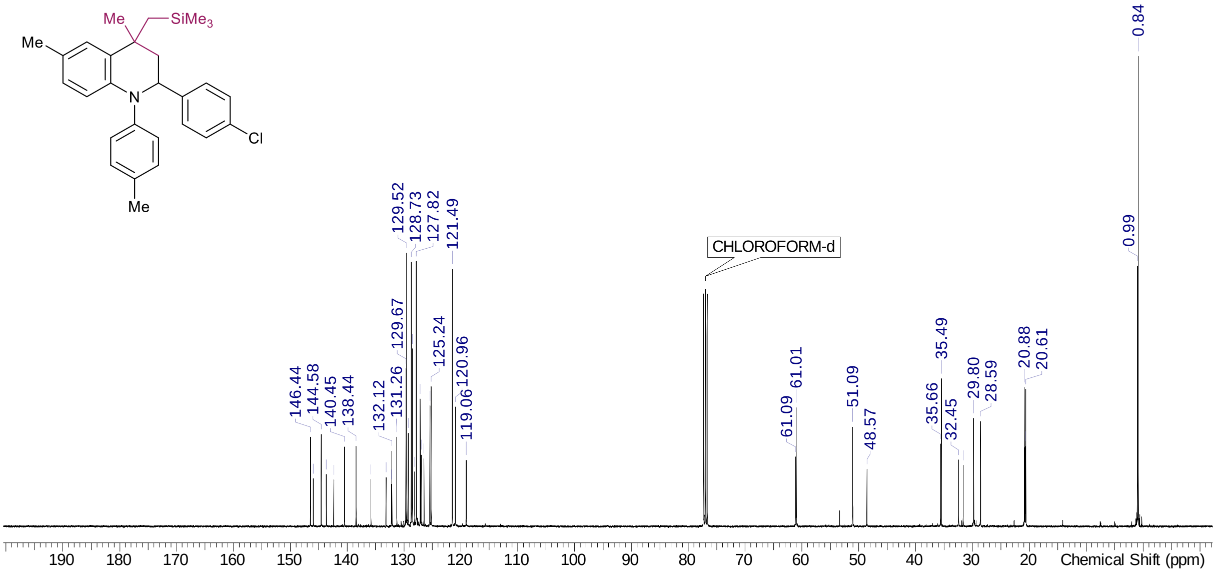

^{1}H, ^{13}C{^{1}H}-HSQC NMR ($CDCl_3$, 400, 101 MHz)

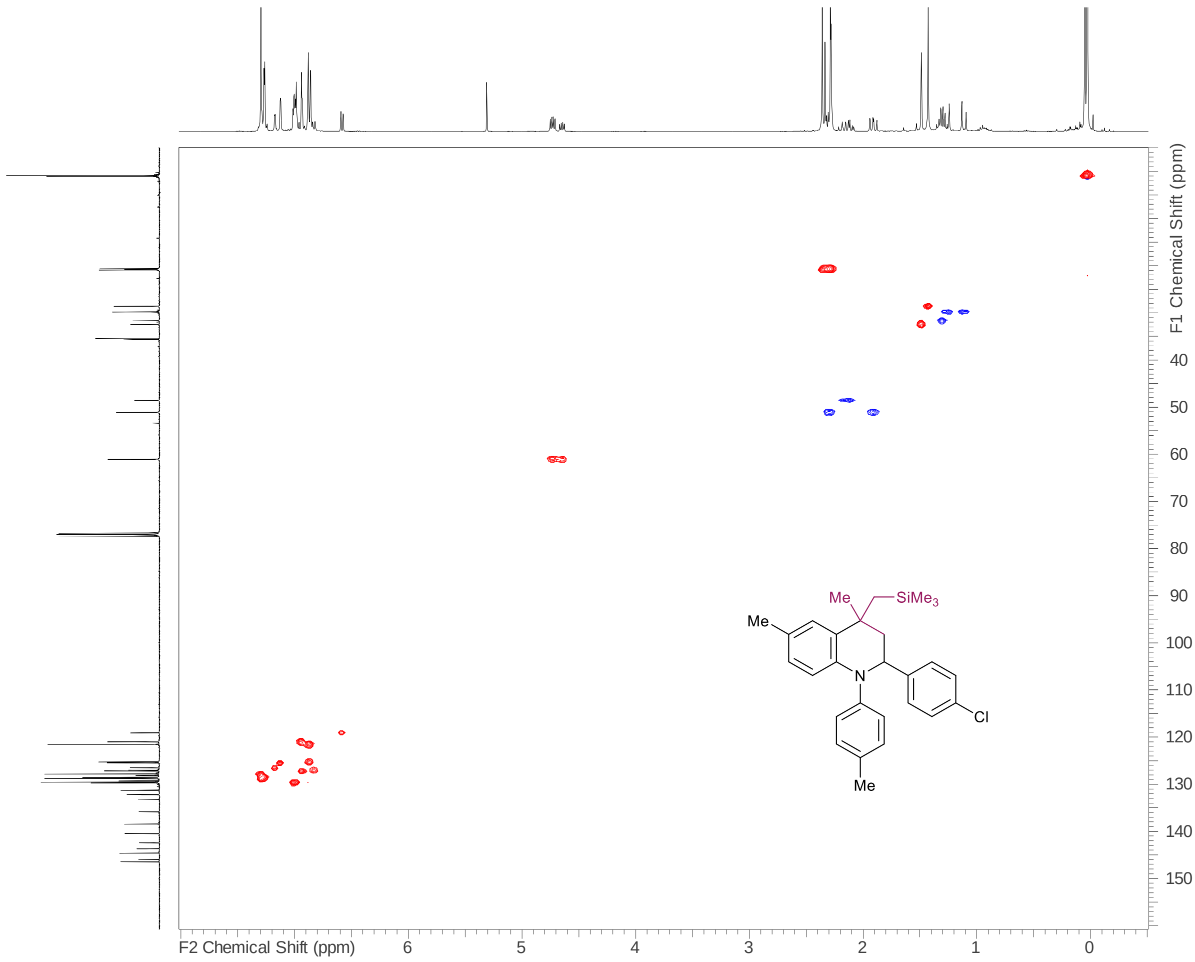

2-(4-Bromophenyl)-4,6-dimethyl-1-(*p*-tolyl)-4-((trimethylsilyl)methyl)-1,2,3,4-tetrahydroquinoline, 3f 1:2 *dr*

^{1}H NMR (400 MHz, $CDCl_3$)

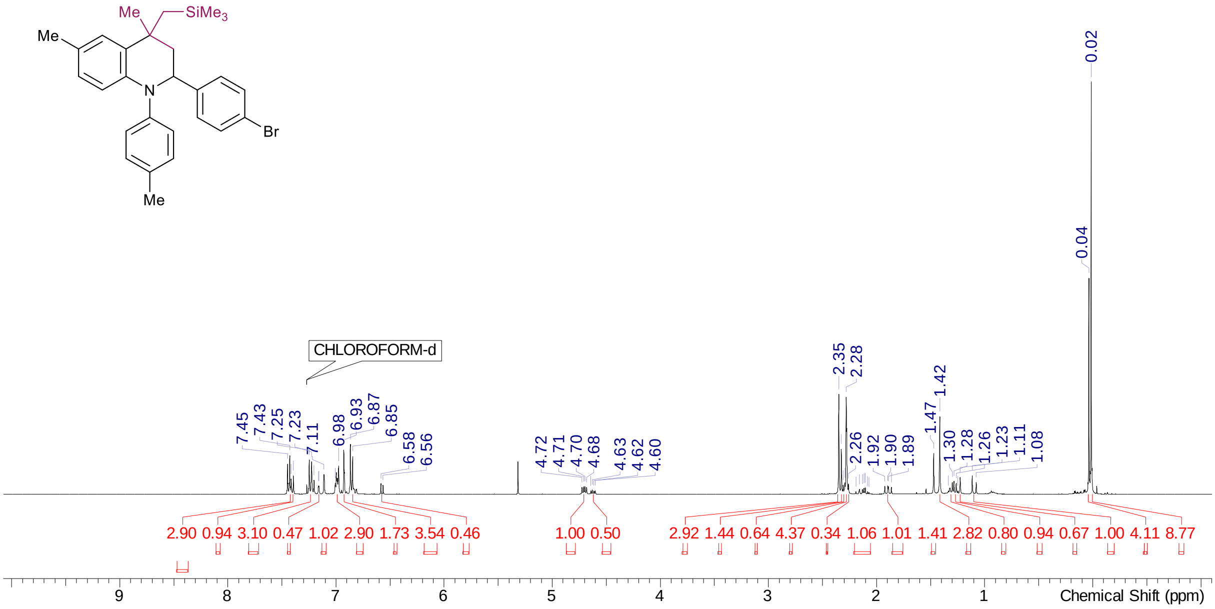

$^{13}C\{^{1}H\}$ NMR ($CDCl_3$, 101 MHz)

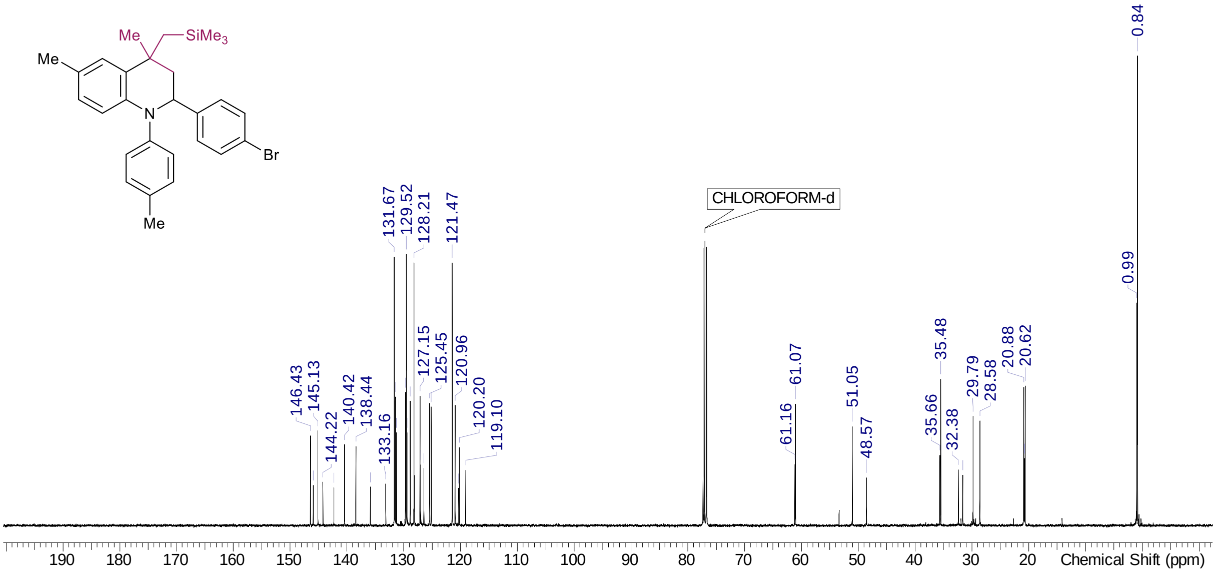

^1H, $^{13}C\{^1H\}$-HSQC NMR ($CDCl_3$, 400, 101 MHz)

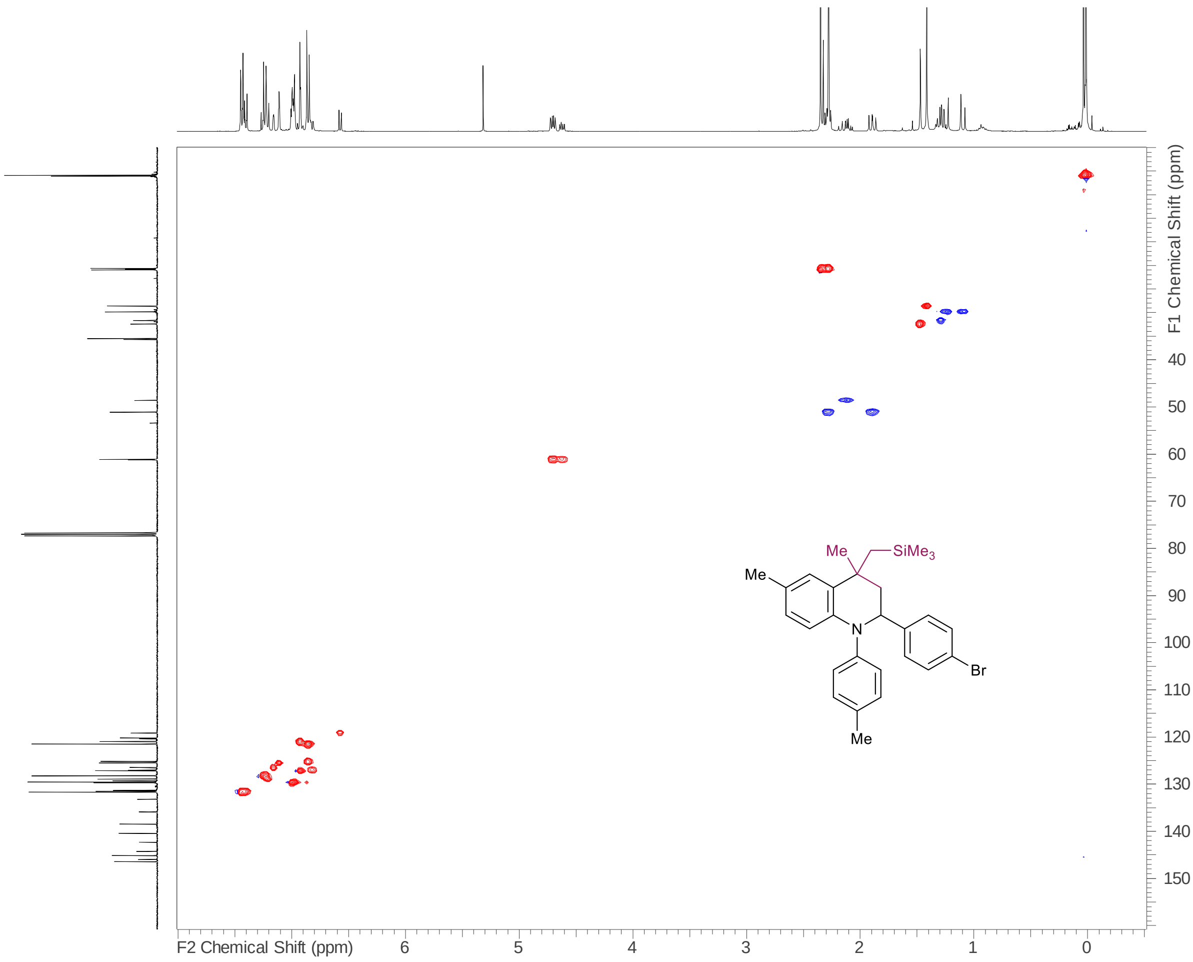

4,6-Dimethyl-2-(4-(methylthio)phenyl)-1-(*p*-tolyl)-4-((trimethylsilyl)methyl)-1,2,3,4-tetrahydroquinoline, 3g 1:1.1 *dr*

^{1}H NMR (500 MHz, $CDCl_3$)

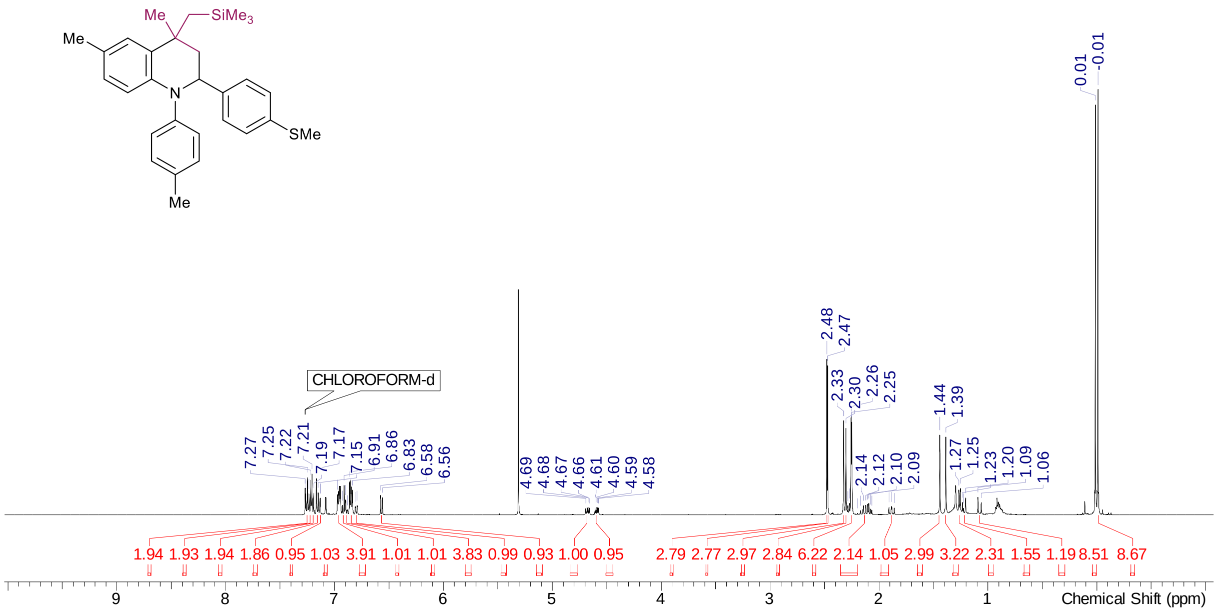

$^{13}C\{^{1}H\}$ NMR ($CDCl_3$, 126 MHz)

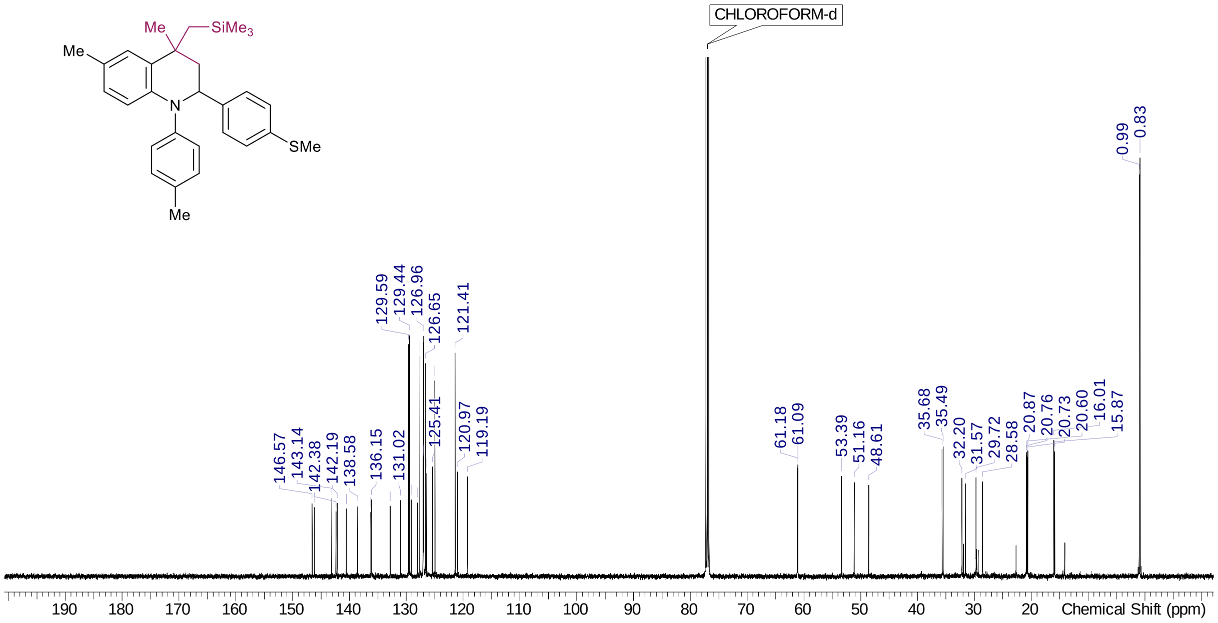

^{1}H, $^{13}C\{^{1}H\}$-HSQC NMR ($CDCl_3$, 500, 126 MHz)

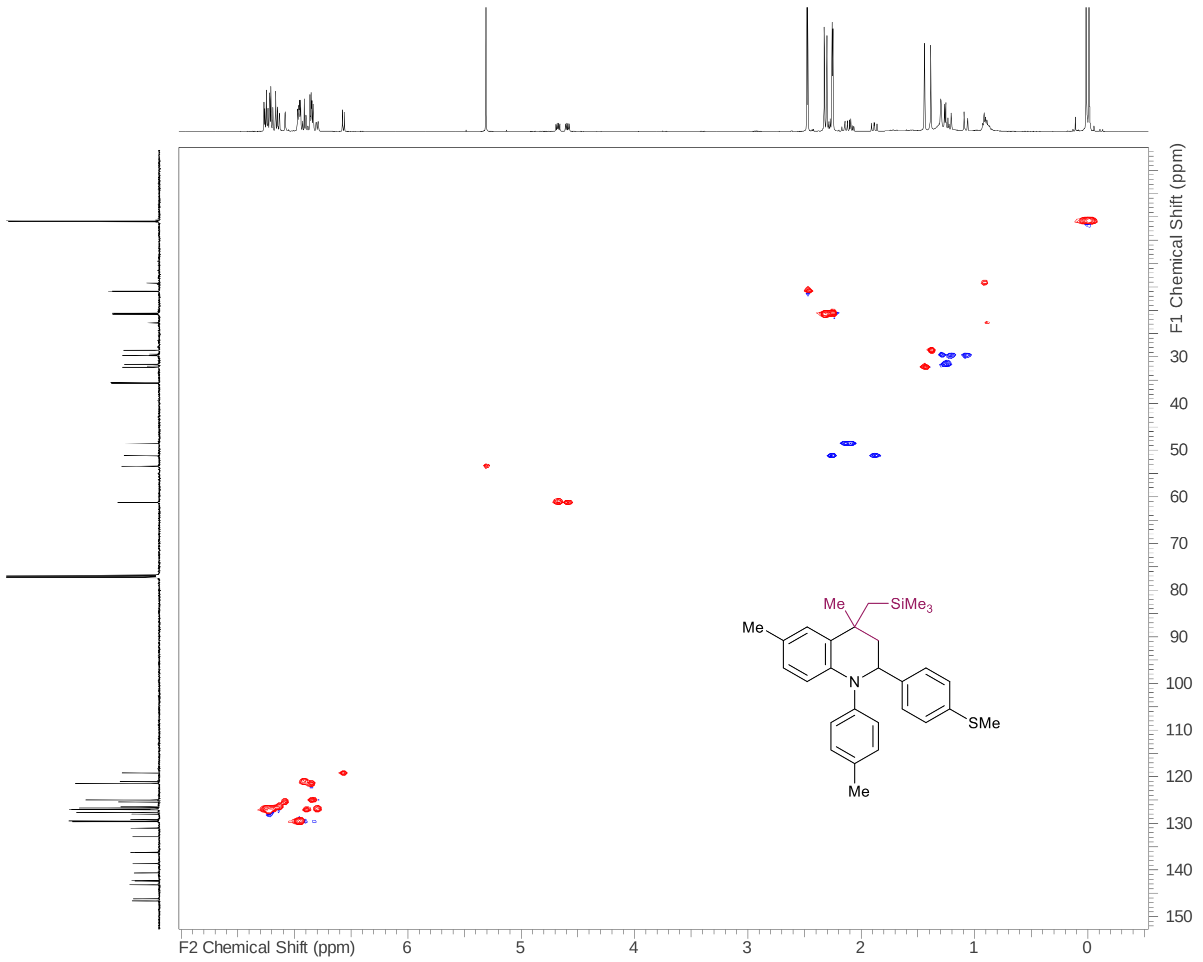

3-Methyl-1-phenyl-3-((trimethylsilyl)methyl)-2,3,7,8-tetrahydro-1*H*-benzo[6,7]azepino[3,2,1-ij]quinoline, 3h

^{1}H NMR (400 MHz, $CDCl_3$)

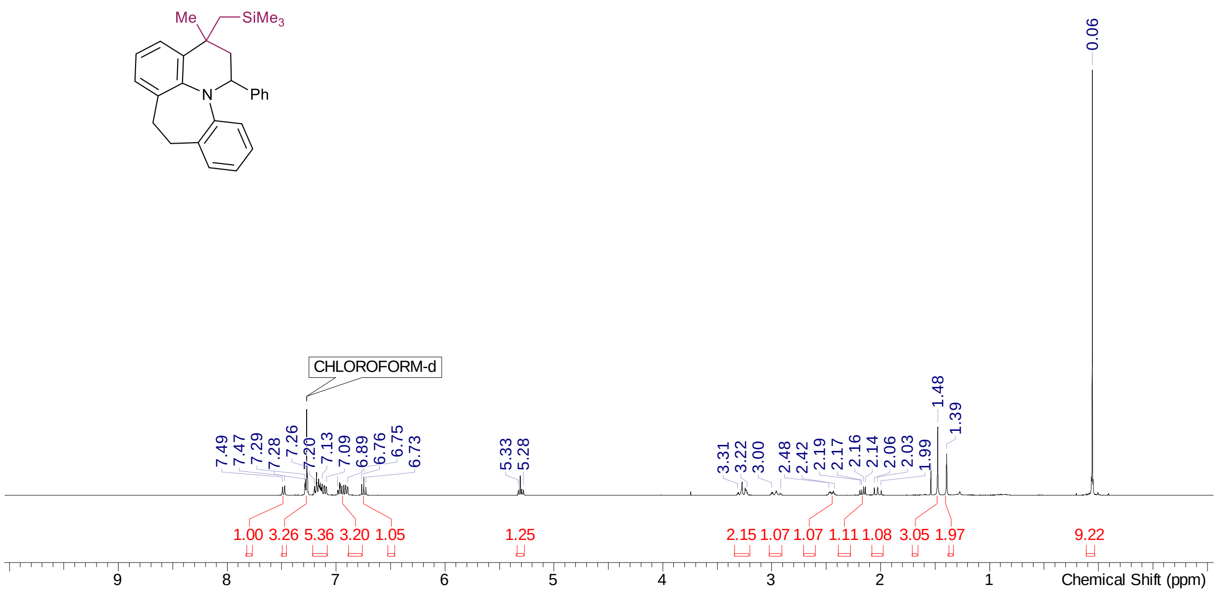

$^{13}C\{^{1}H\}$ NMR ($CDCl_3$, 101 MHz)

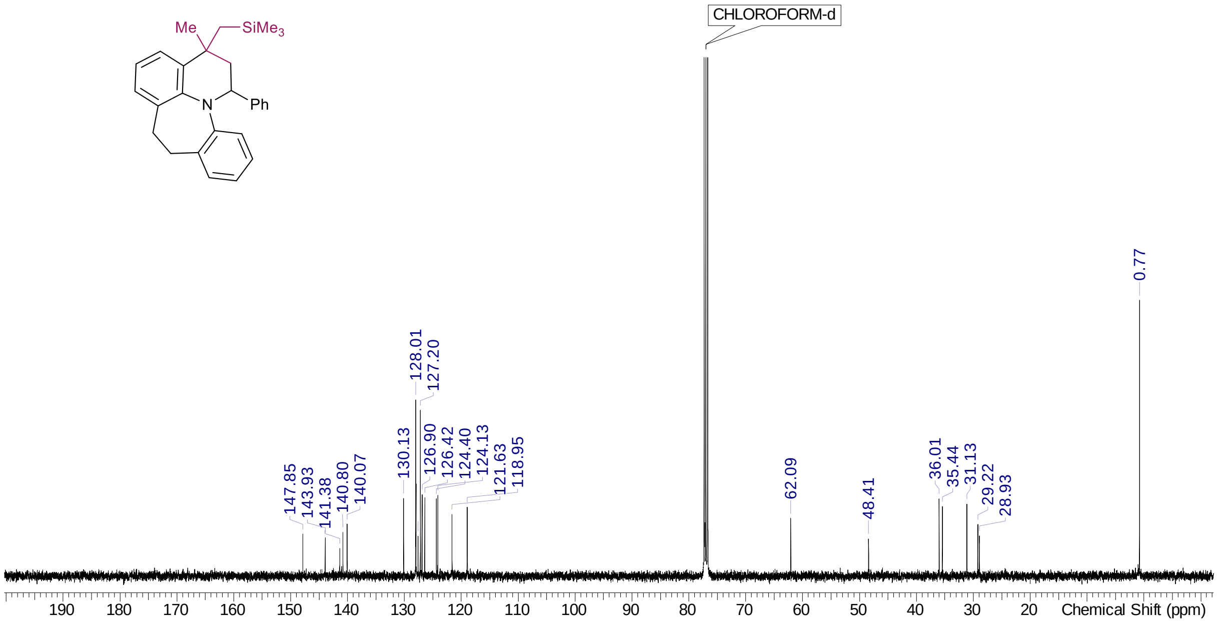

^{1}H, ^{13}C{^{1}H}-HSQC NMR ($CDCl_3$, 400, 101 MHz)

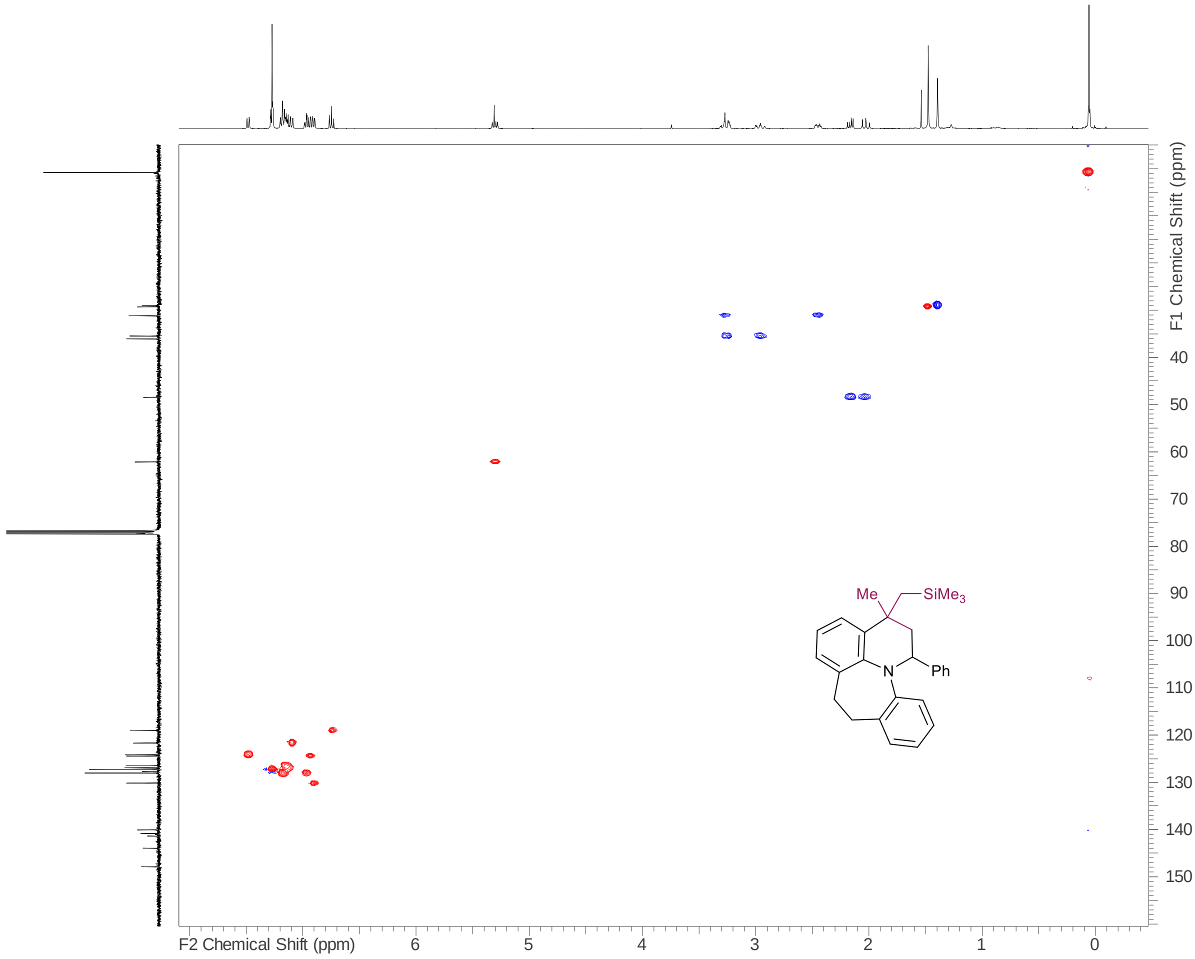

3-Methyl-3-((trimethylsilyl)methyl)-2,3,7,8-tetrahydro-1*H*-benzo[6,7]azepino[3,2,1-ij]quinoline, 3i

^{1}H NMR (400 MHz, $CDCl_3$)

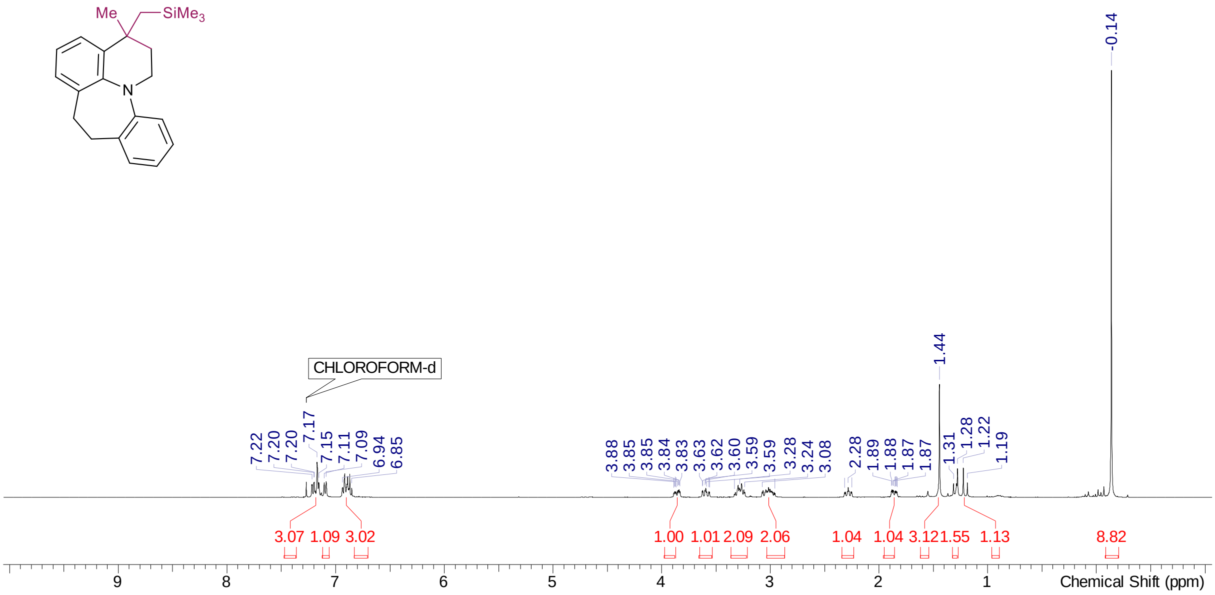

^{13}C{^{1}H} NMR ($CDCl_3$, 101 MHz)

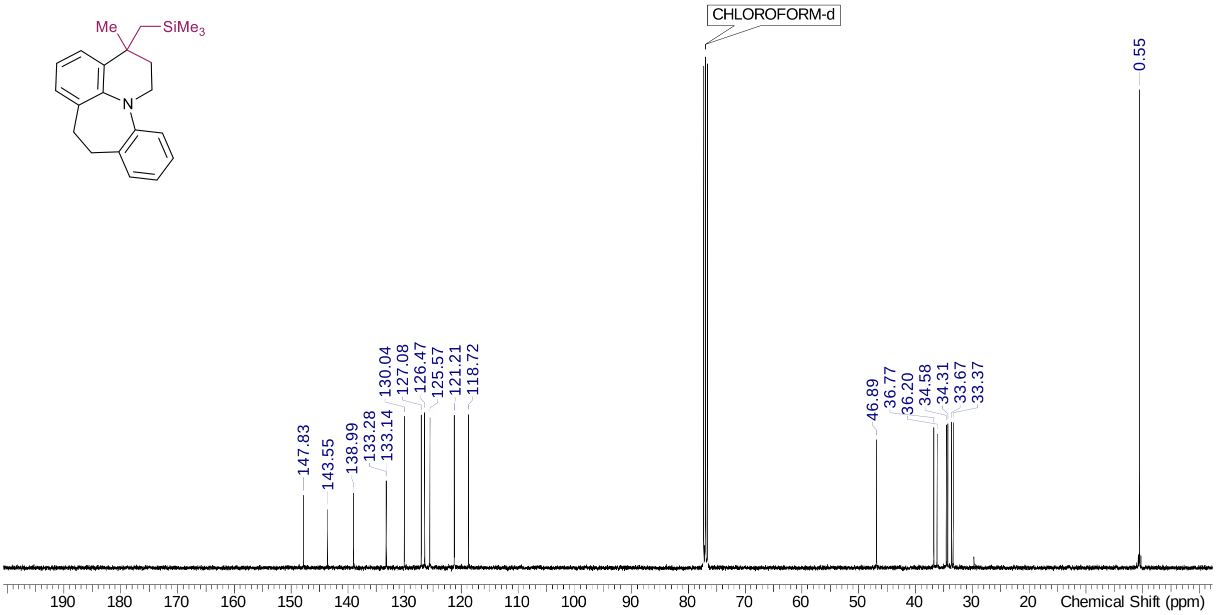

^{1}H, $^{13}C\{^{1}H\}$-HSQC NMR ($CDCl_3$, 400, 101 MHz)

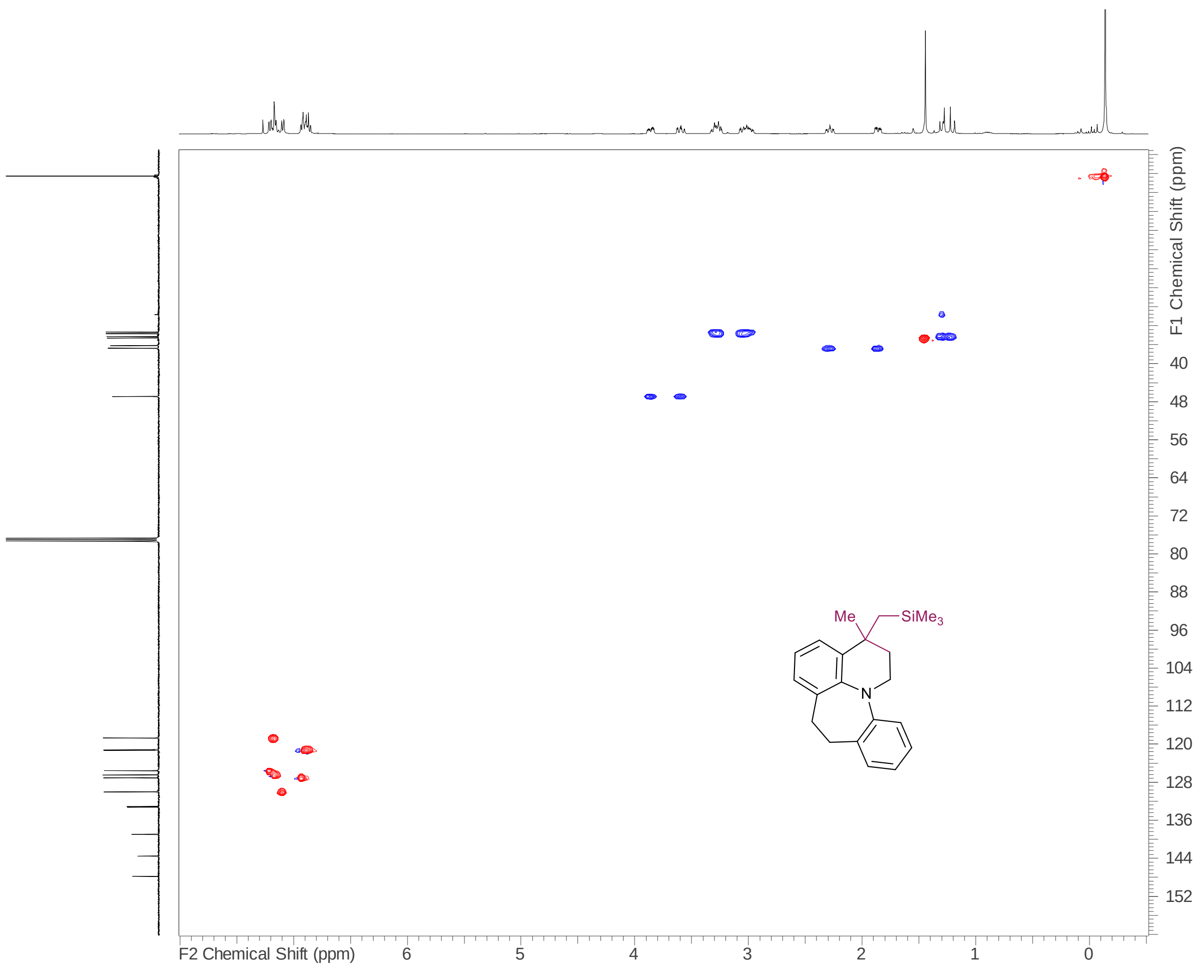

1,4-Dimethyl-4-((trimethylsilyl)methyl)-1,2,3,4-tetrahydroquinoline, 3j

^{1}H NMR (500 MHz, $CDCl_3$)

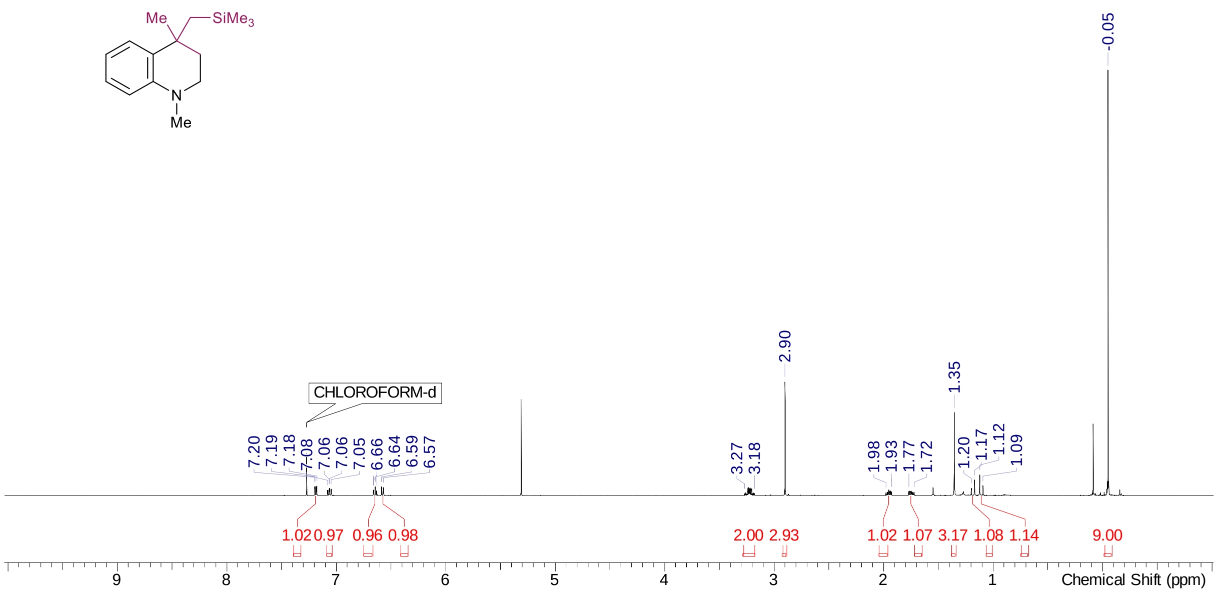

$^{13}C\{^{1}H\}$ NMR ($CDCl_3$, 126 MHz)

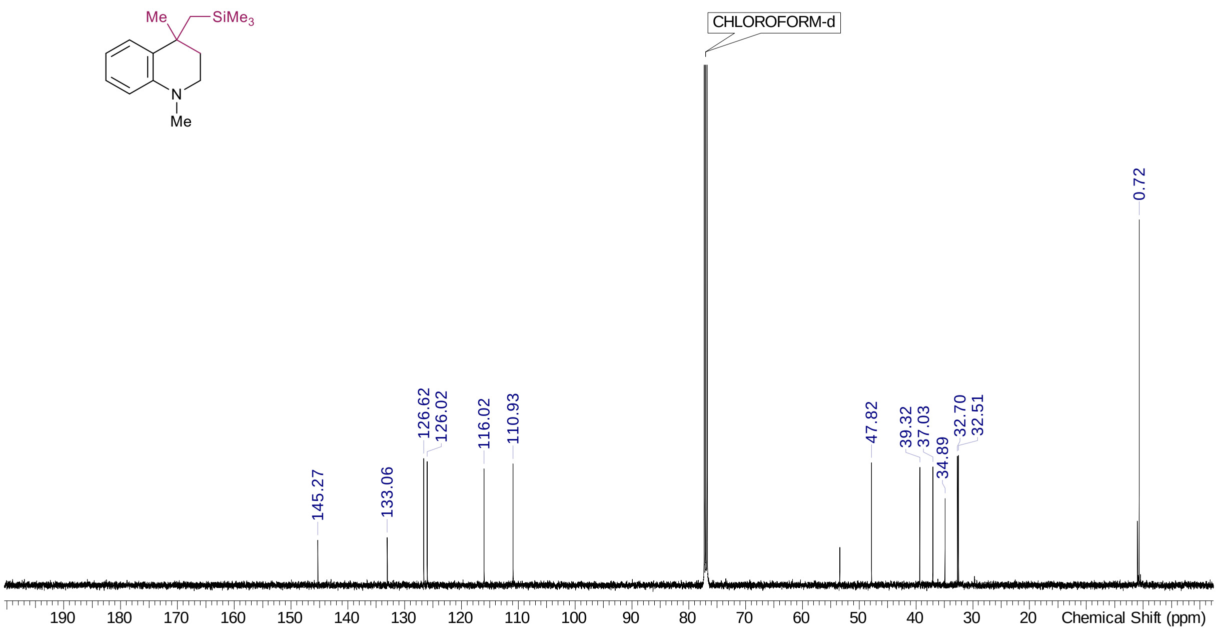

^{1}H, $^{13}C\{^{1}H\}$-HSQC NMR ($CDCl_3$, 500, 126 MHz)

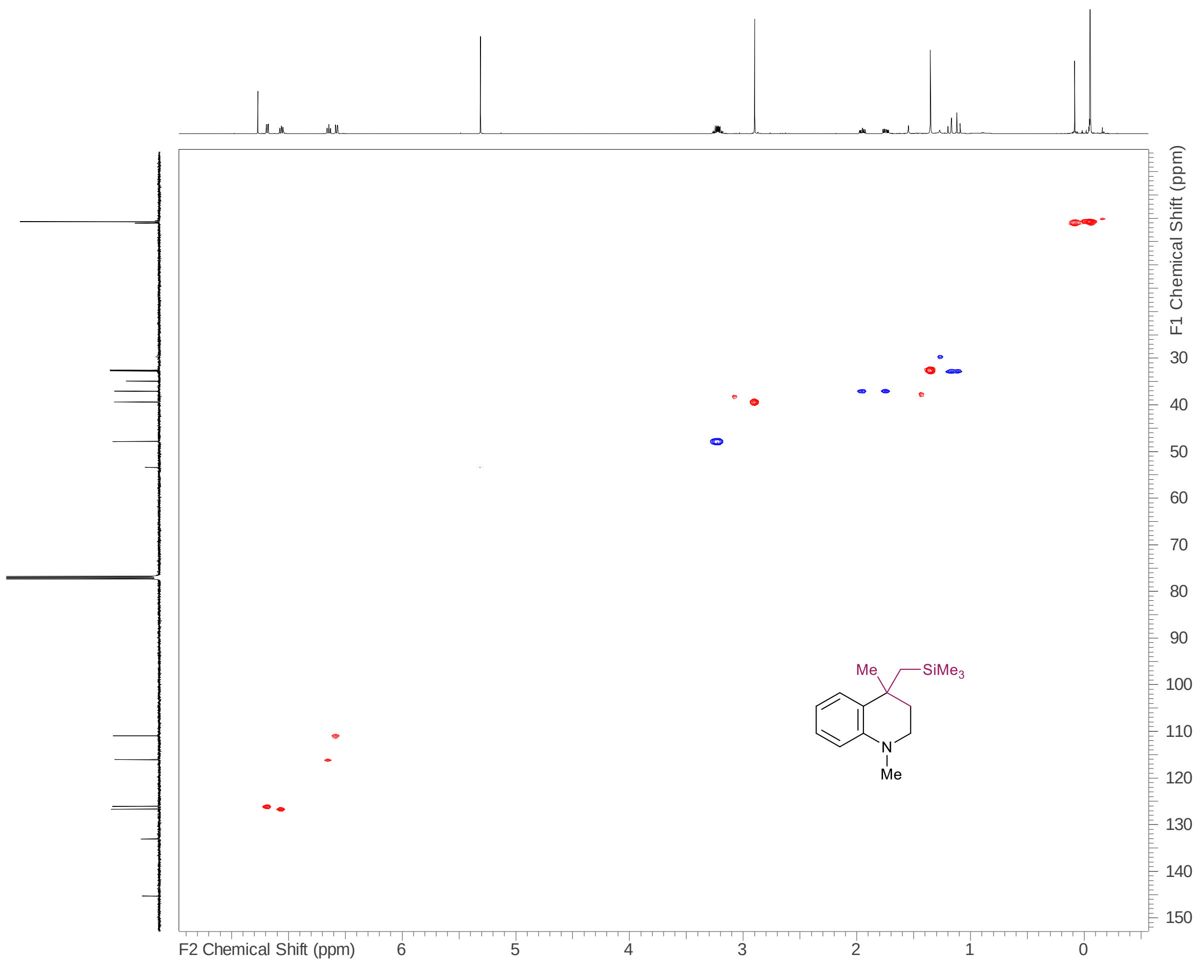

6-Bromo-1,4-dimethyl-4-((trimethylsilyl)methyl)-1,2,3,4-tetrahydroquinoline, 3k

^{1}H NMR (400 MHz, $CDCl_3$)

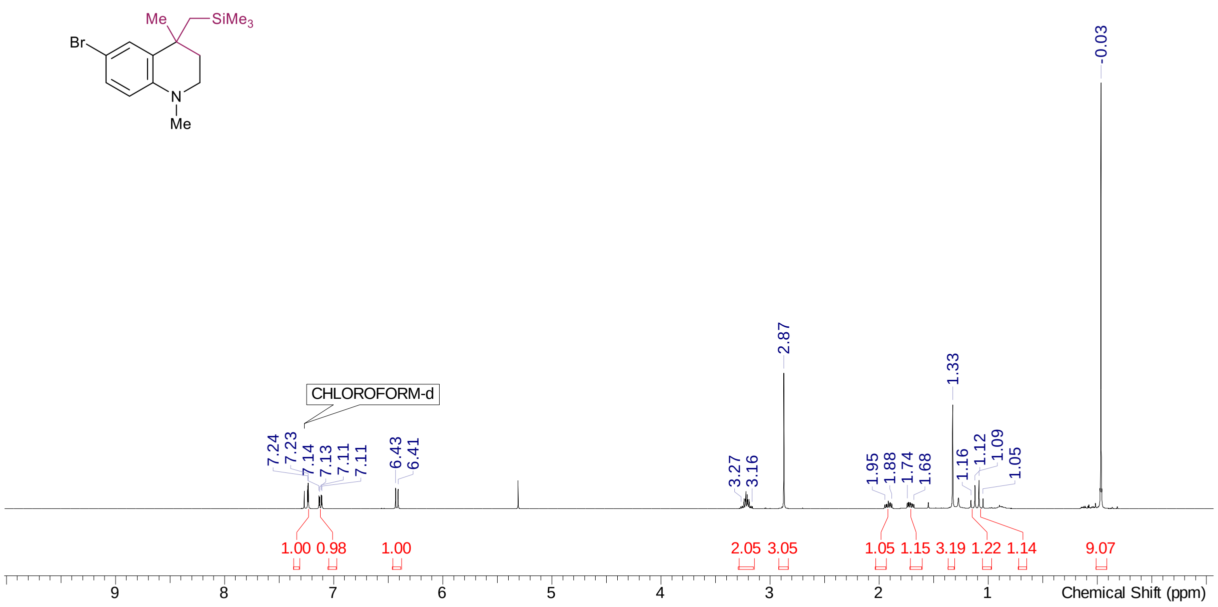

^{13}C{^{1}H} NMR ($CDCl_3$, 101 MHz)

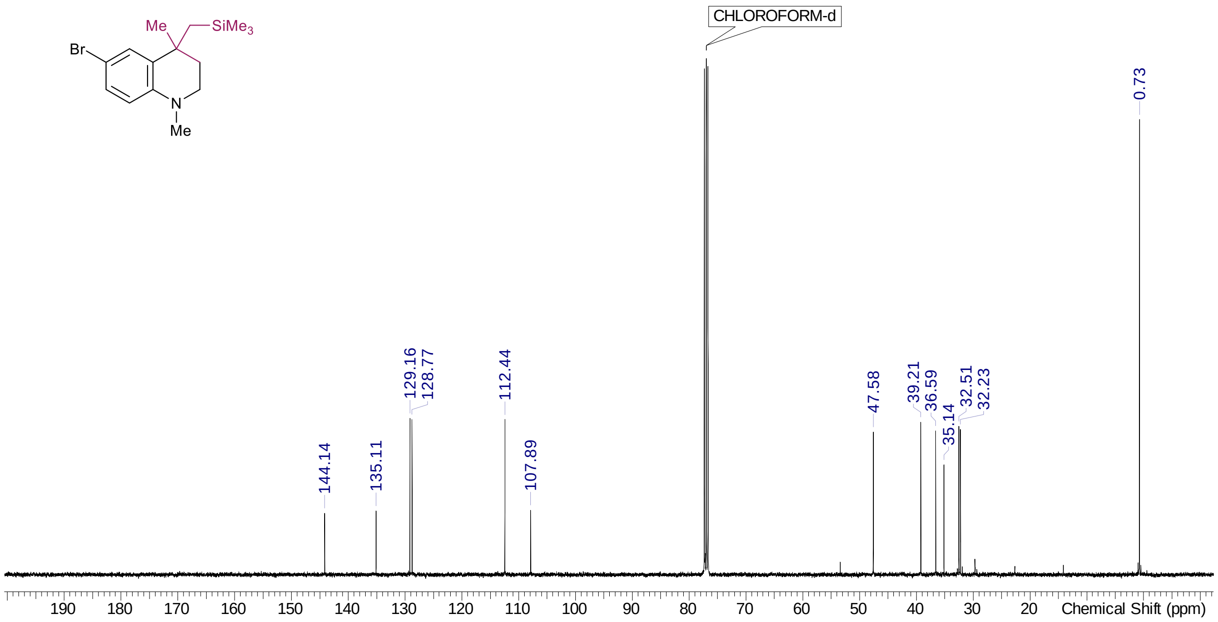

^{1}H, ^{13}C{^{1}H}-HSQC NMR ($CDCl_3$, 400, 101 MHz)

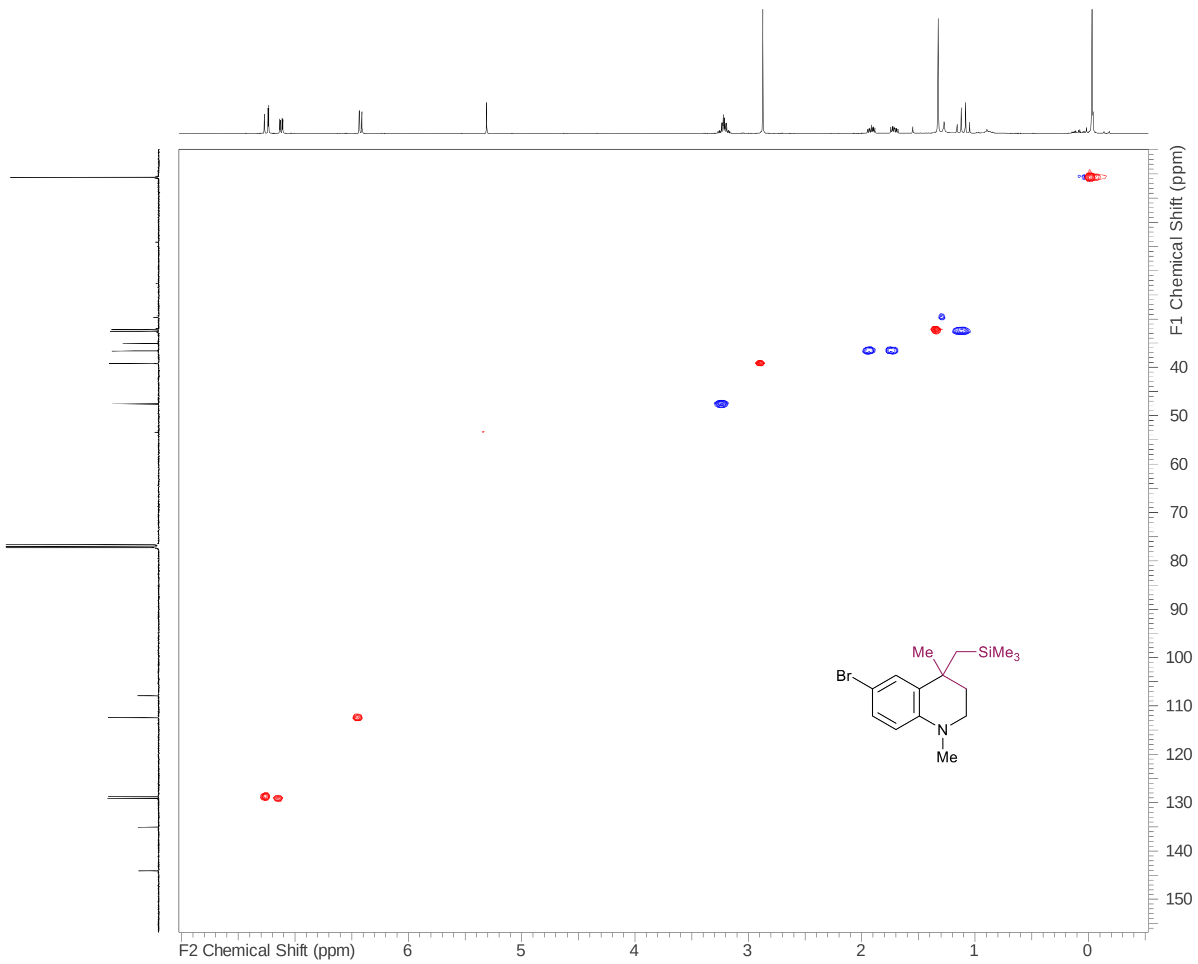

6-Iodo-1,4-dimethyl-4-((trimethylsilyl)methyl)-1,2,3,4-tetrahydroquinoline, 3l

^{1}H NMR (400 MHz, $CDCl_3$)

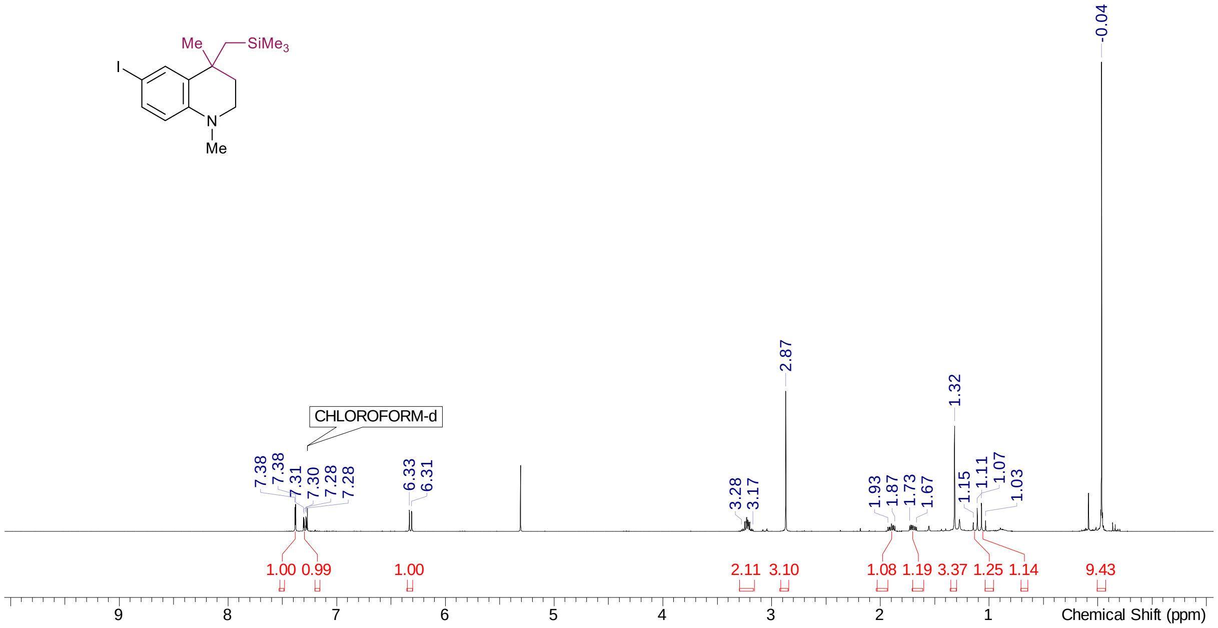

$^{13}C\{^1H\}$ NMR ($CDCl_3$, 101 MHz)

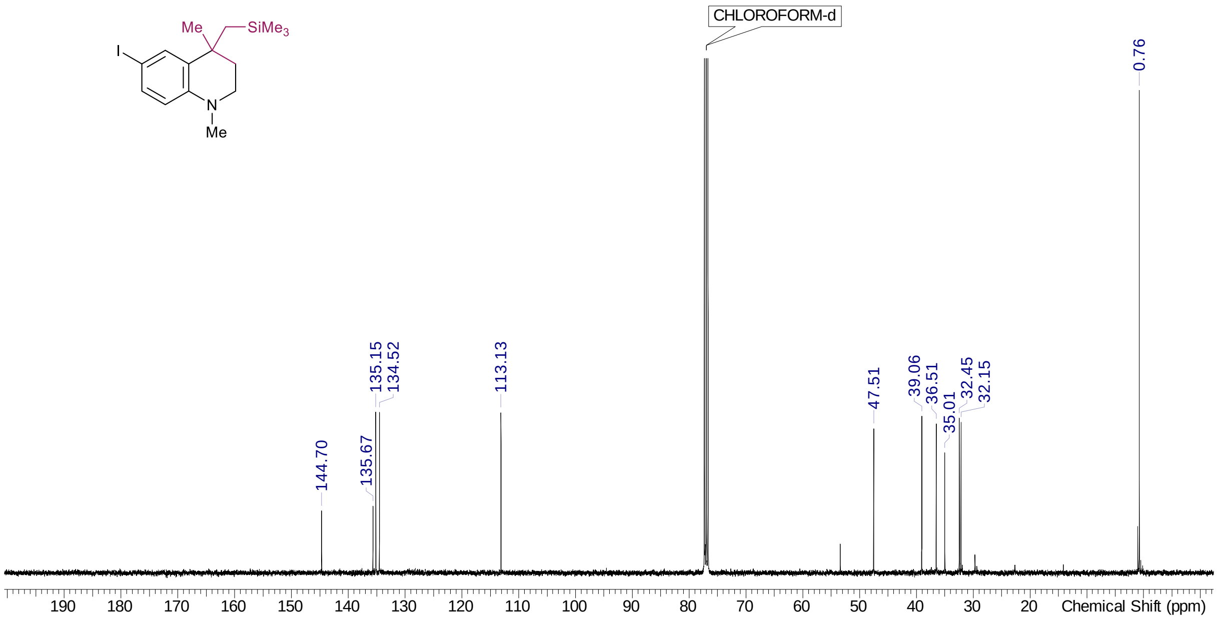

^{1}H, ^{13}C{^{1}H}-HSQC NMR ($CDCl_3$, 400, 101 MHz)

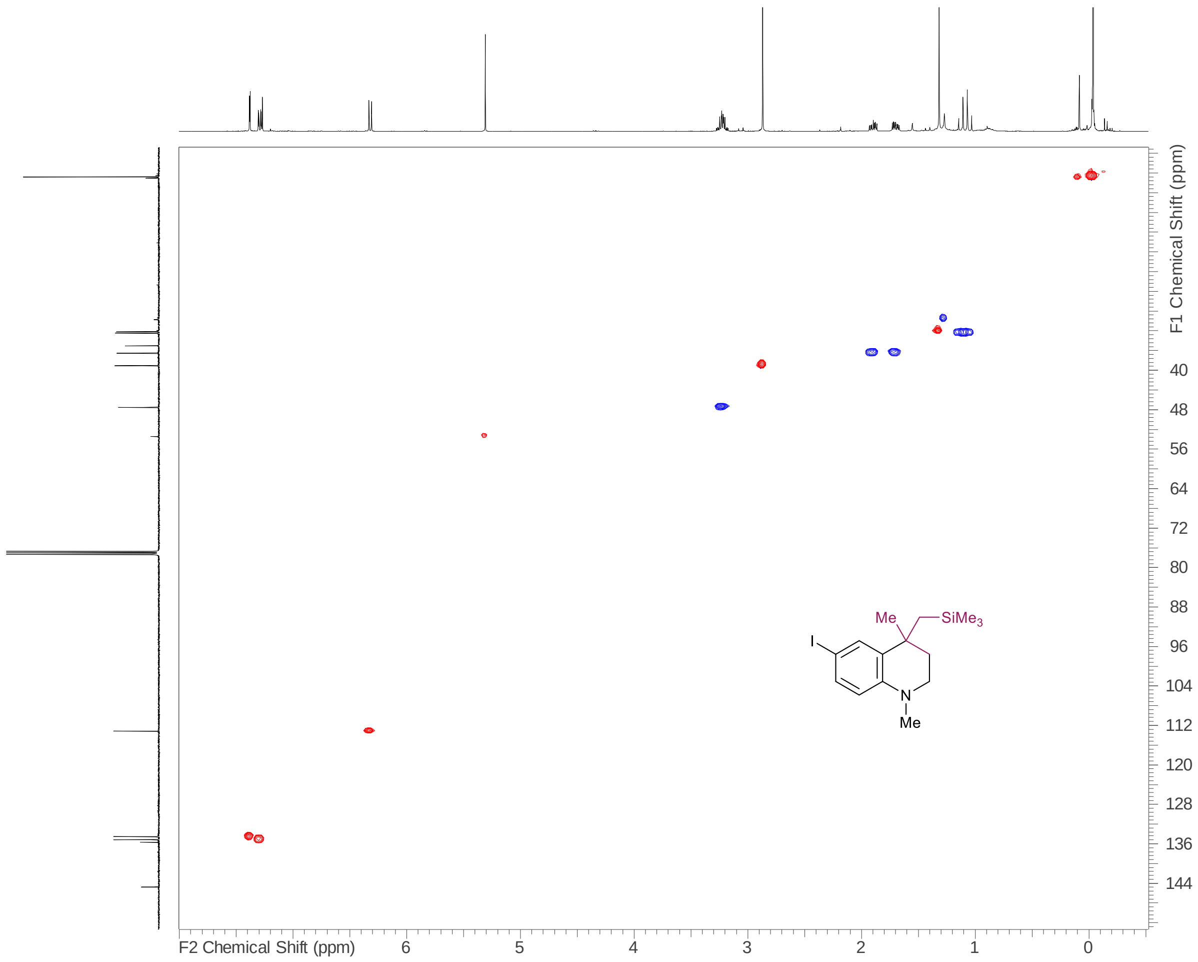

7-Bromo-1,4-dimethyl-4-((trimethylsilyl)methyl)-1,2,3,4-tetrahydroquinoline, 3m

^{1}H NMR (500 MHz, $CDCl_3$)

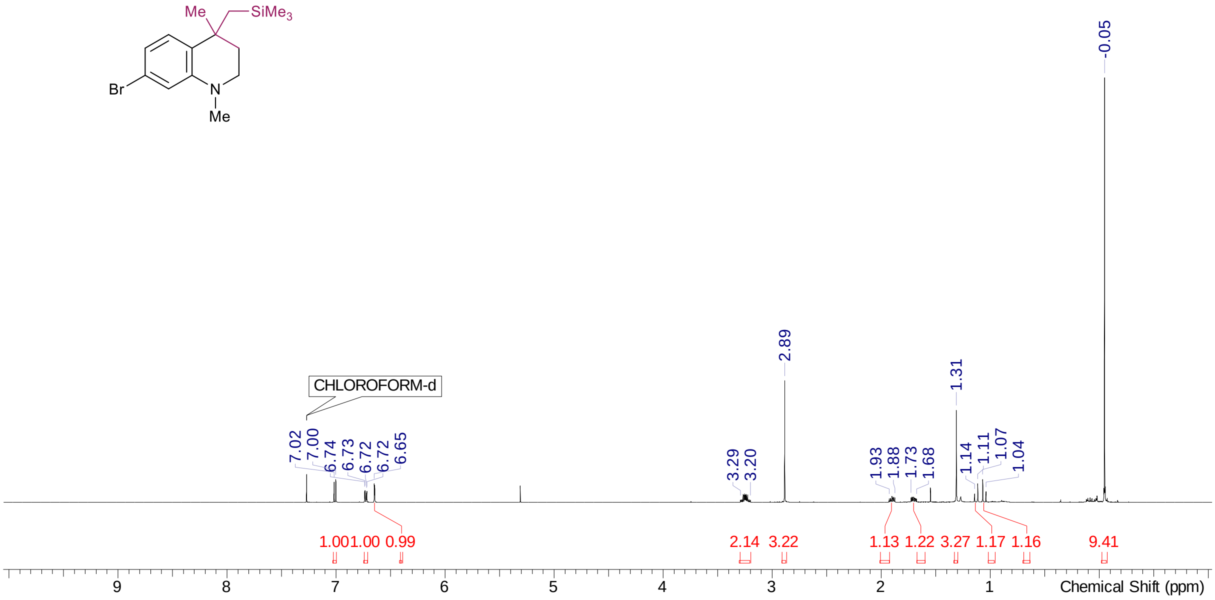

$^{13}C\{^{1}H\}$ NMR ($CDCl_3$, 126 MHz)

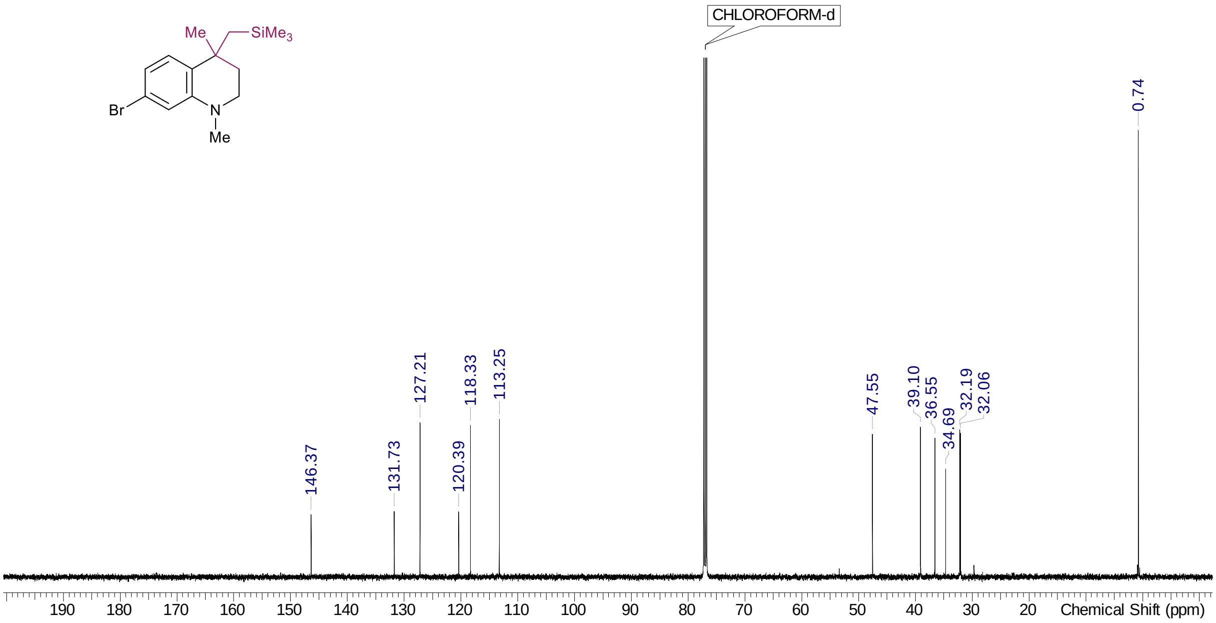

^{1}H, ^{13}C{^{1}H}-HSQC NMR ($CDCl_3$, 500, 126 MHz)

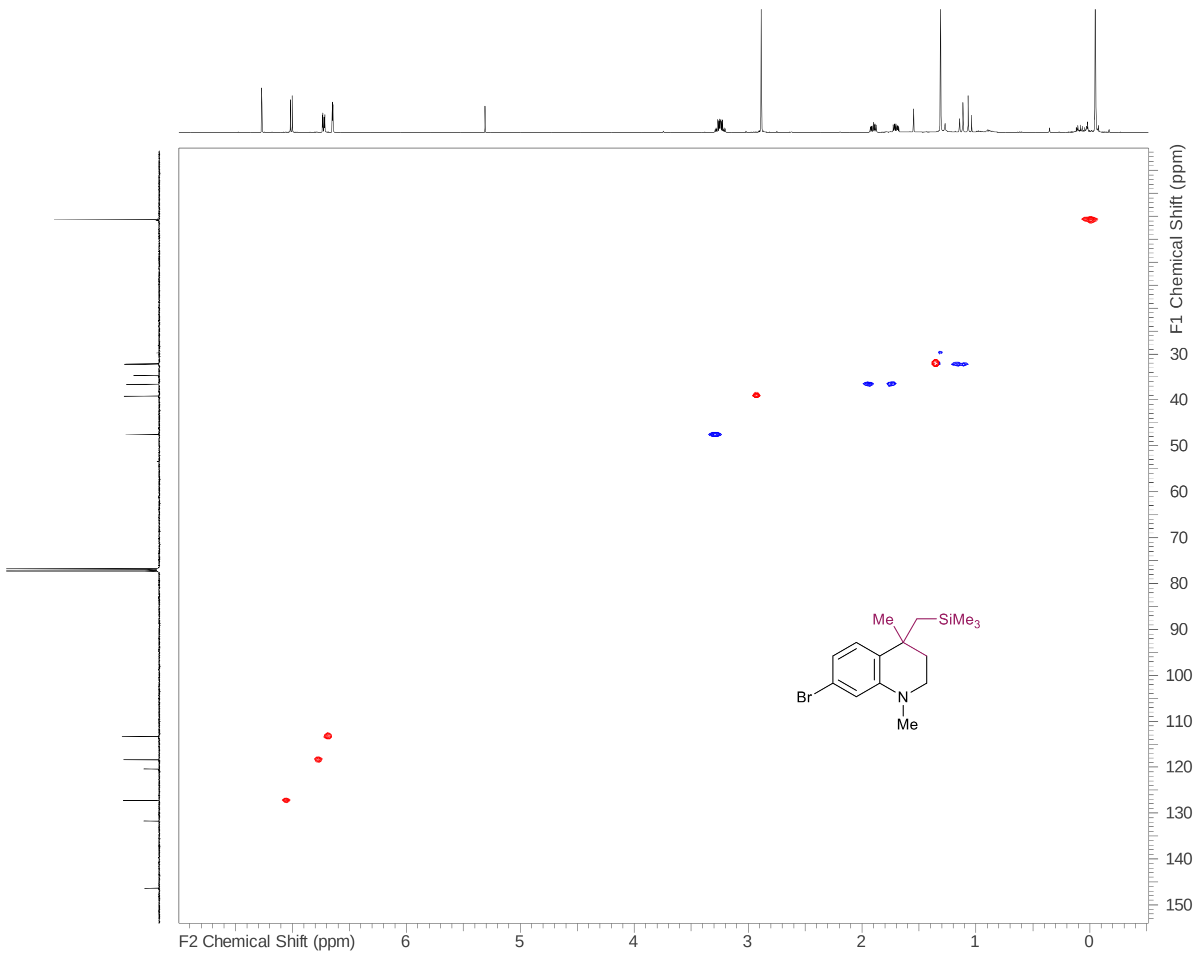

1-Benzyl-4-methyl-2-phenyl-4-((trimethylsilyl)methyl)-1,2,3,4-tetrahydroquinoline, 3n 1:1.5 *dr*

^{1}H NMR (400 MHz, $CDCl_3$)

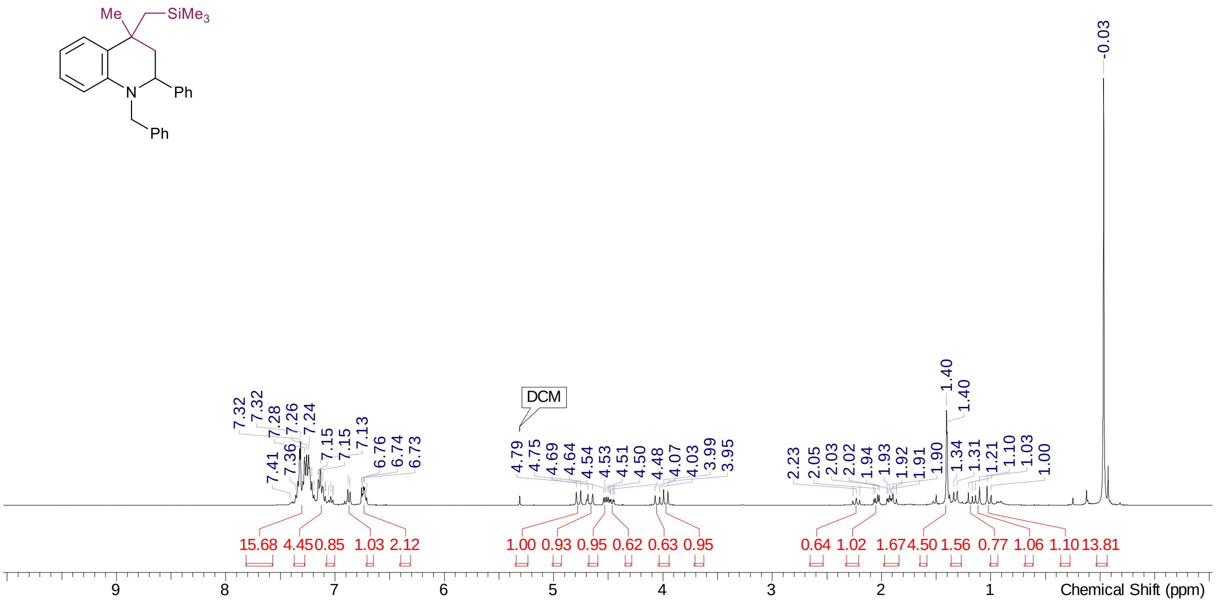

^{13}C{^{1}H} NMR ($CDCl_3$, 101 MHz)

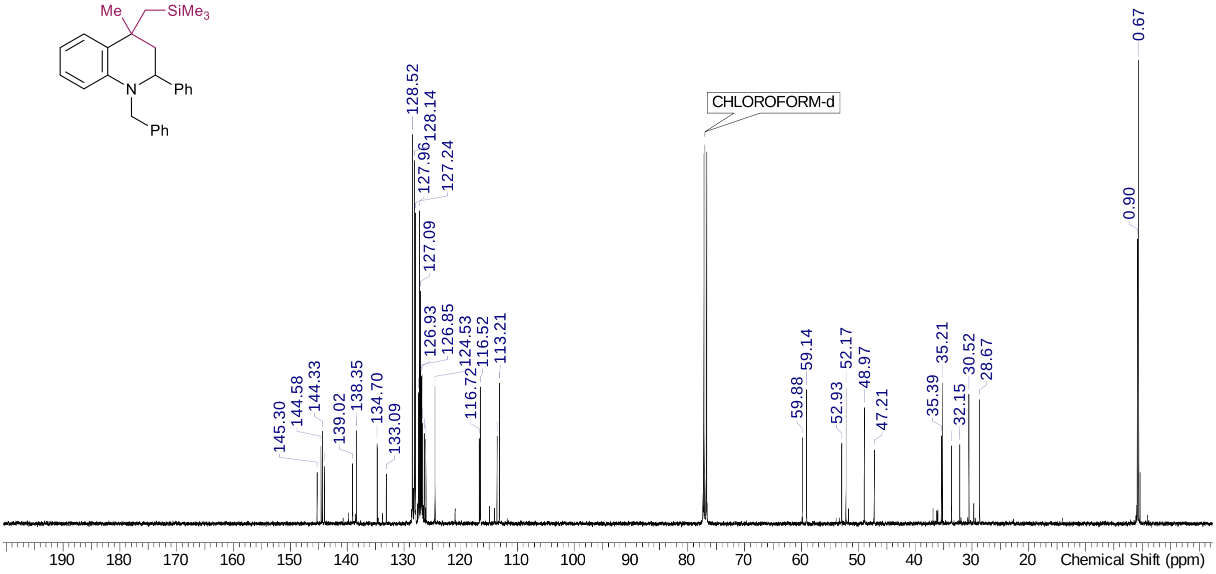

^{1}H, $^{13}C\{^{1}H\}$-HSQC NMR ($CDCl_3$, 400, 101 MHz)

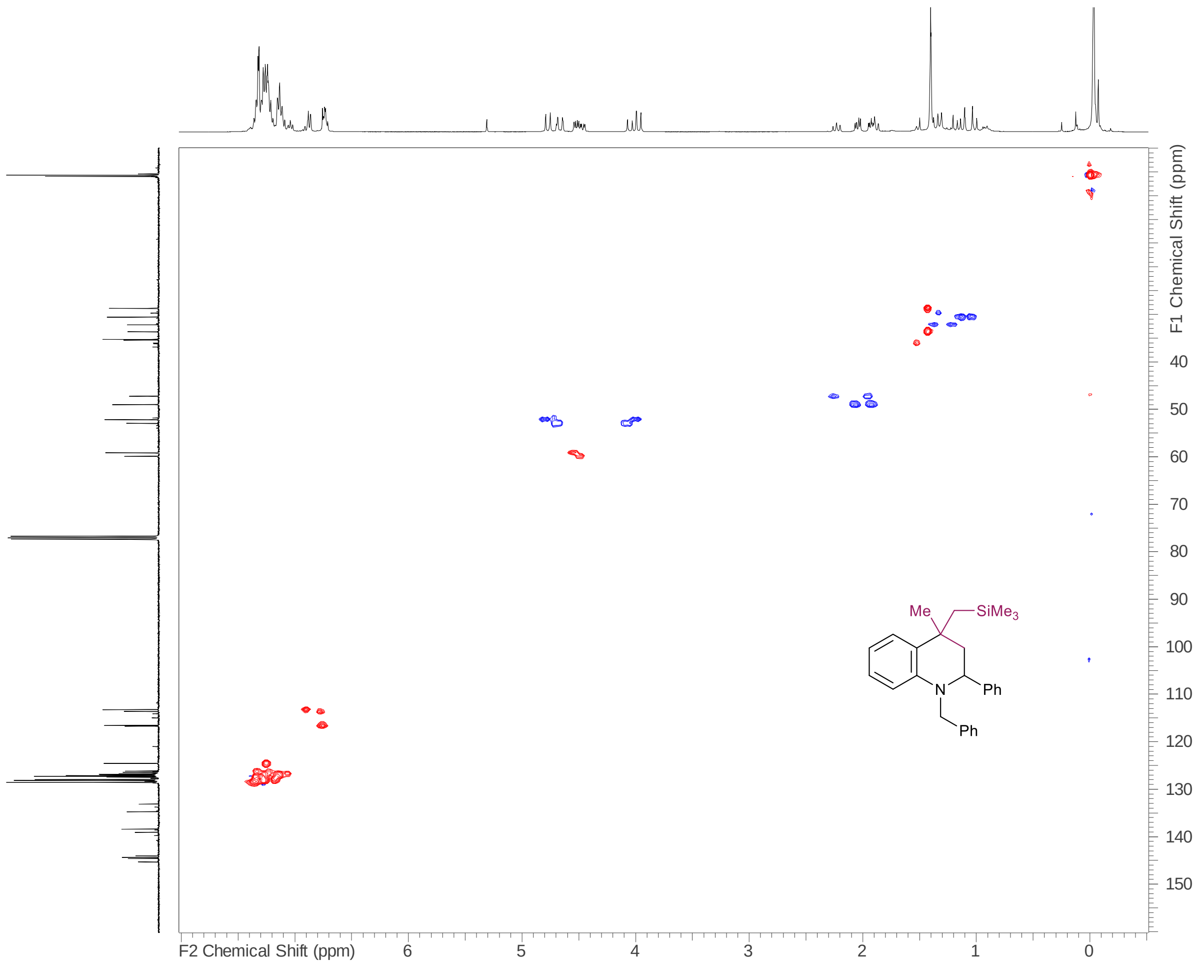

1-Benzyl-4,6-dimethyl-2-phenyl-4-((trimethylsilyl)methyl)-1,2,3,4-tetrahydroquinoline 3o

1:1.3 *dr*

^{1}H NMR (400 MHz, $CDCl_3$)

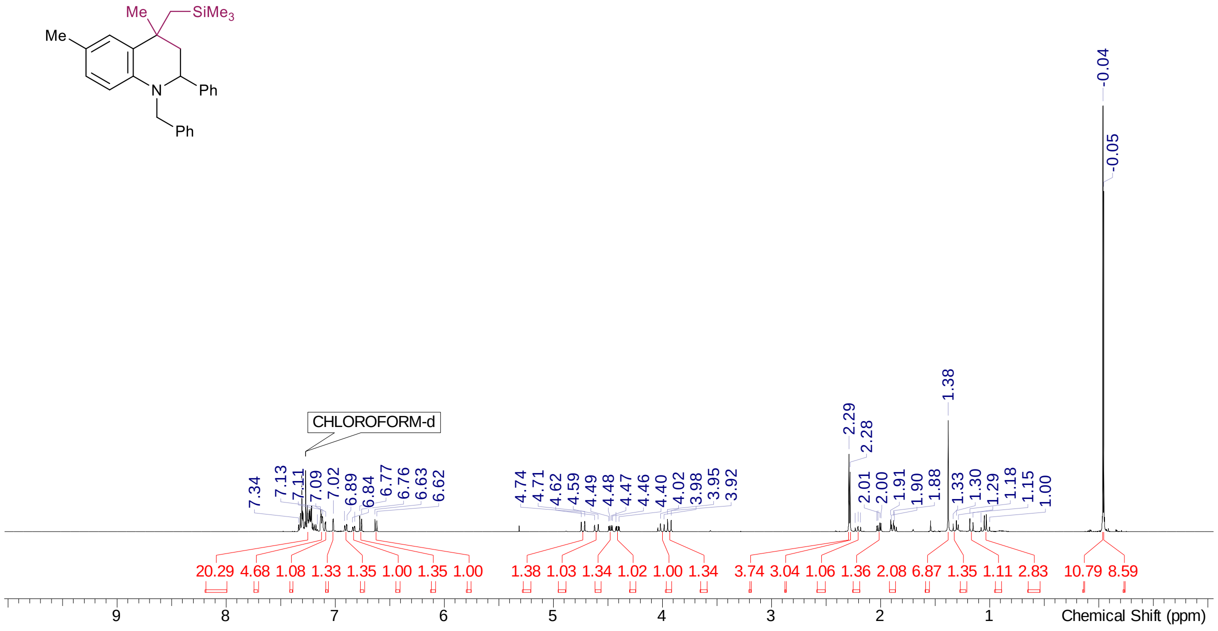

$^{13}C\{^{1}H\}$ NMR ($CDCl_3$, 101 MHz)

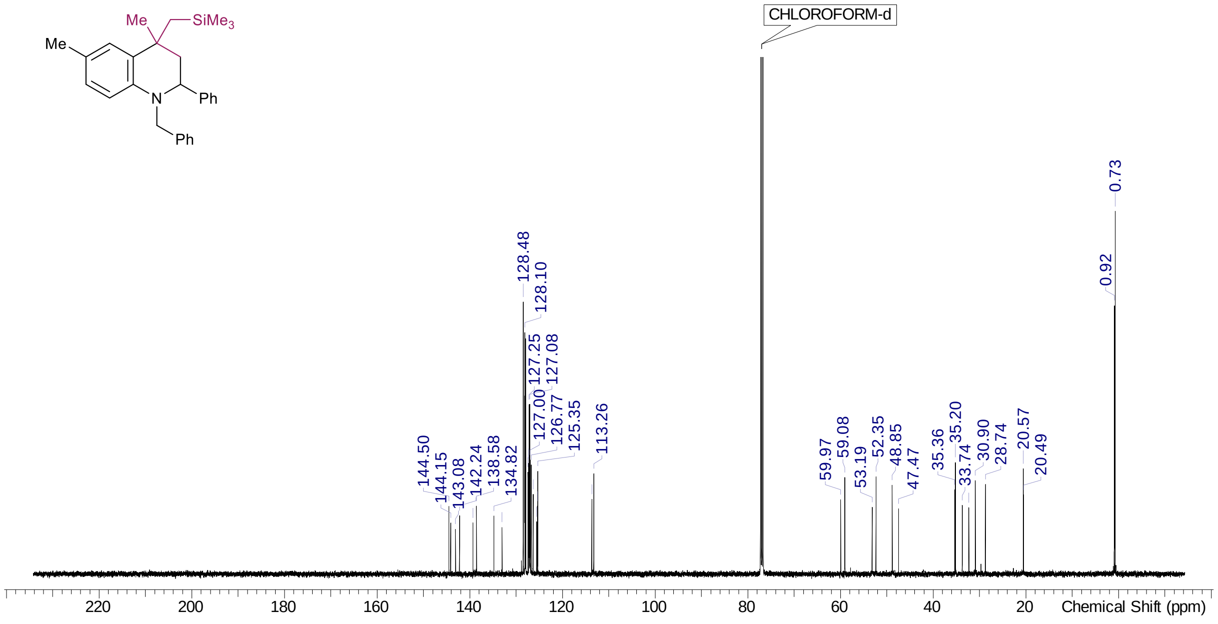

^{1}H, $^{13}C\{^{1}H\}$-HSQC NMR ($CDCl_3$, 400, 101 MHz)

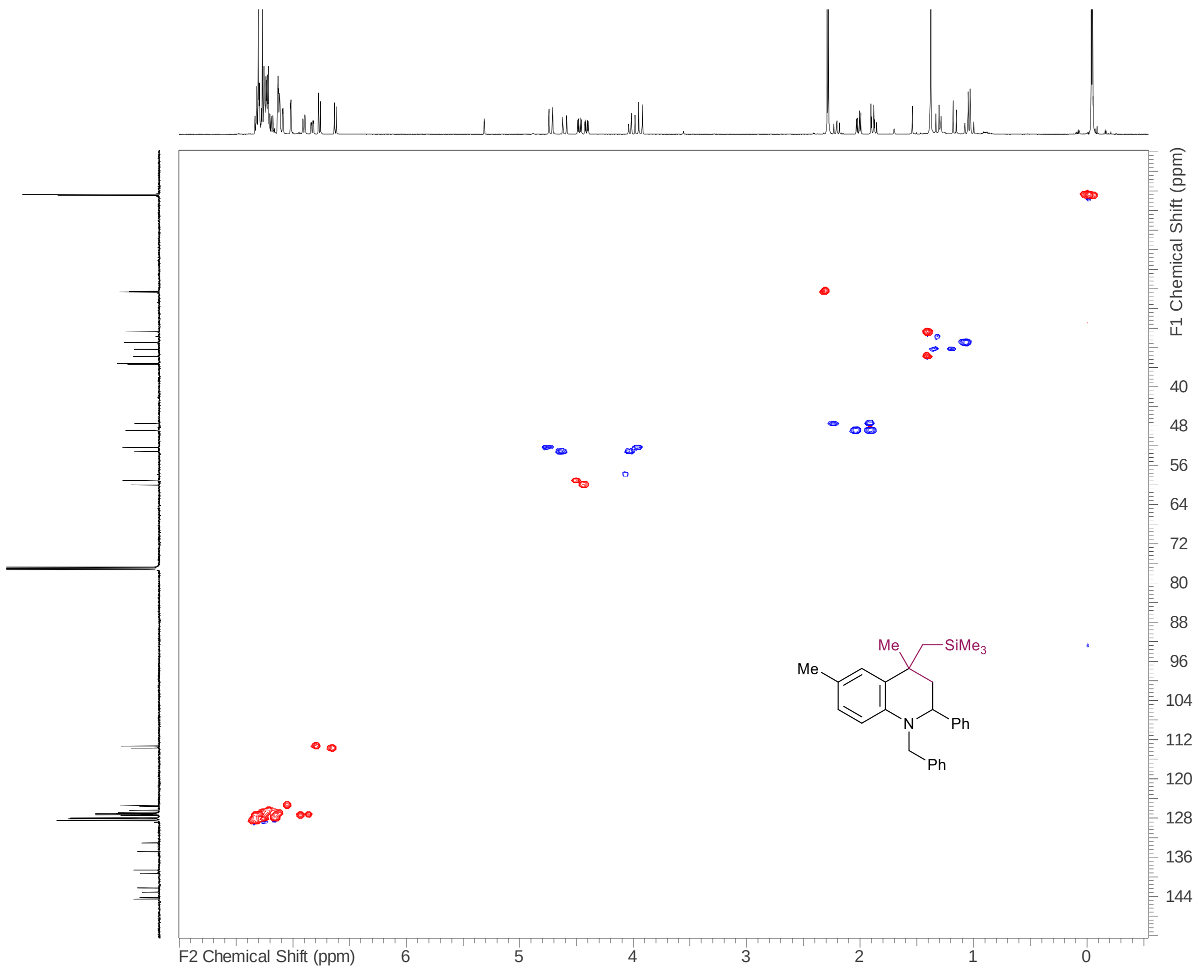

1-Benzyl-4-methyl-2-phenyl-4-((trimethylsilyl)methyl)-1,2,3,4-tetrahydrobenzo[h]quinoline, 3p

1:1.4 *dr*

^{1}H NMR (400 MHz, $CDCl_3$)

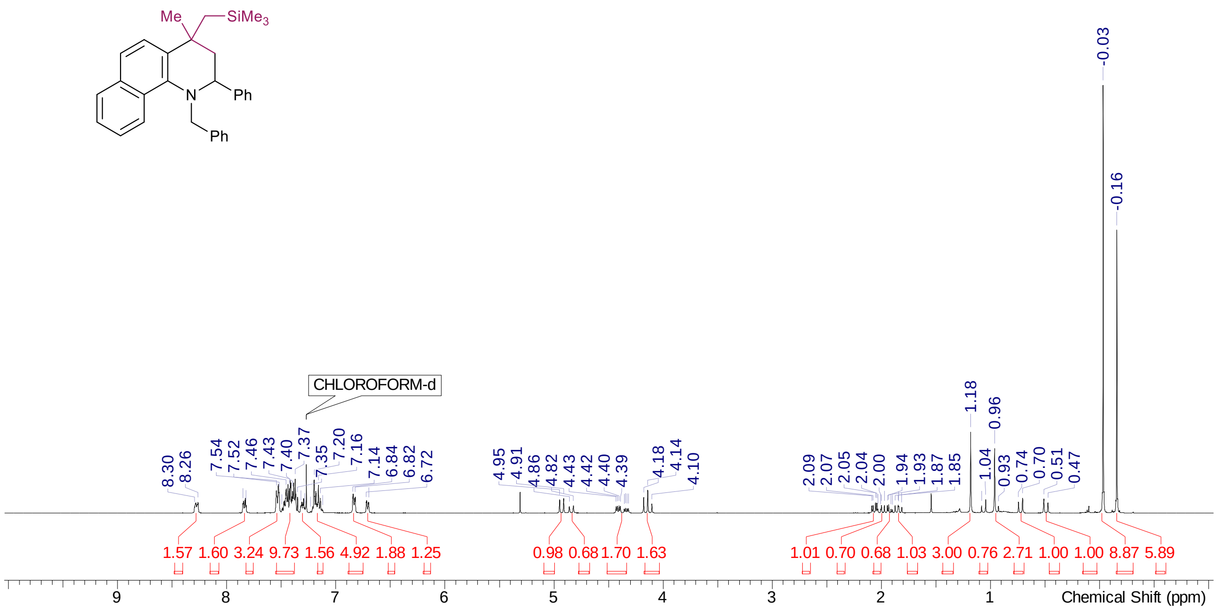

^{13}C{^{1}H} NMR ($CDCl_3$, 101 MHz)

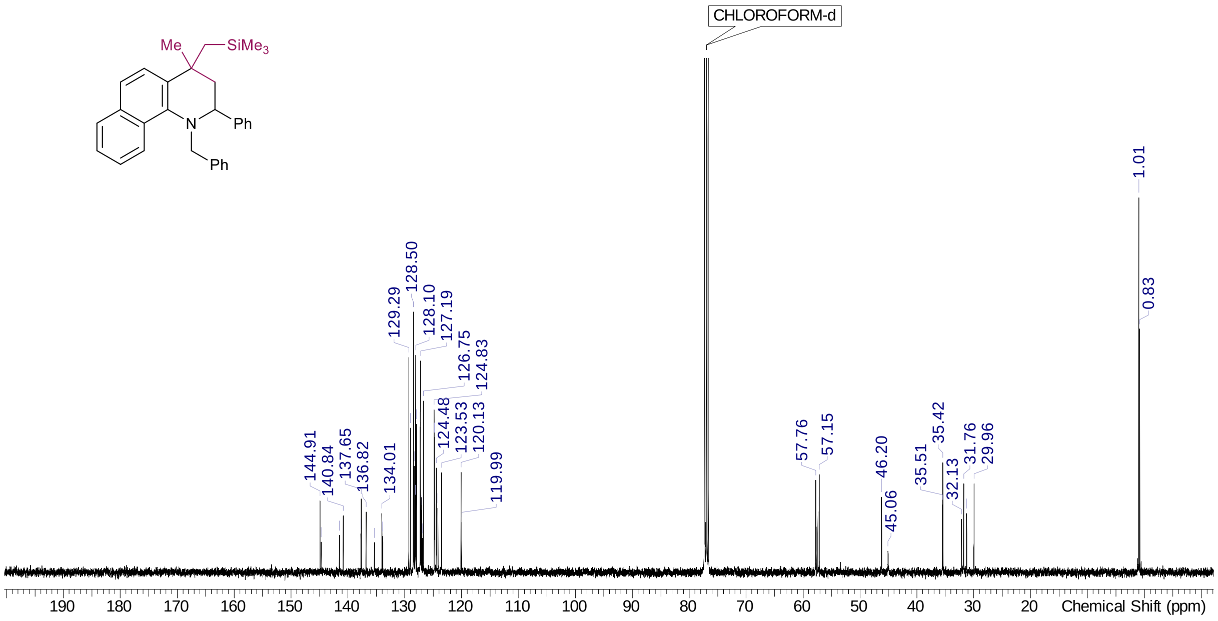

^{1}H, $^{13}C\{^{1}H\}$-HSQC NMR ($CDCl_3$, 400, 101 MHz)

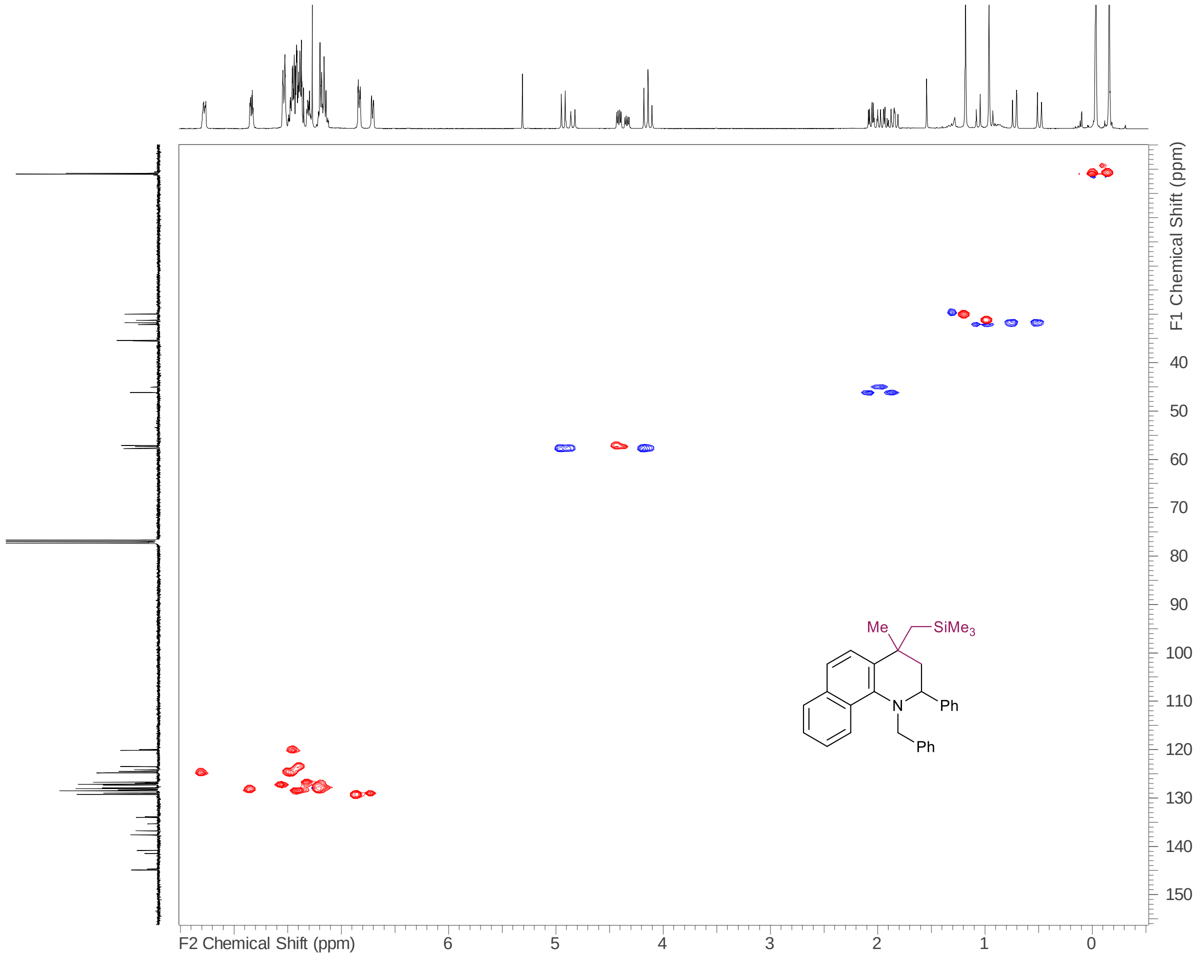

1-Benzyl-4-methyl-4-((trimethylsilyl)methyl)-1,2,3,4-tetrahydroquinoline, 3q

^{1}H NMR (400 MHz, $CDCl_3$)

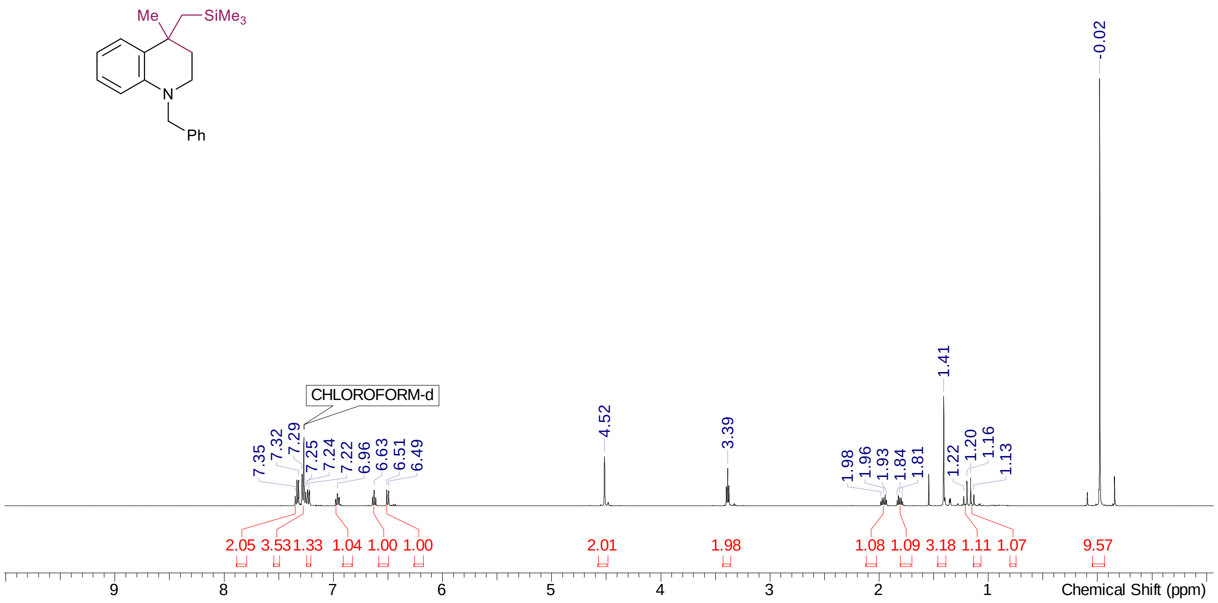

$^{13}C\{^{1}H\}$ NMR ($CDCl_3$, 101 MHz)

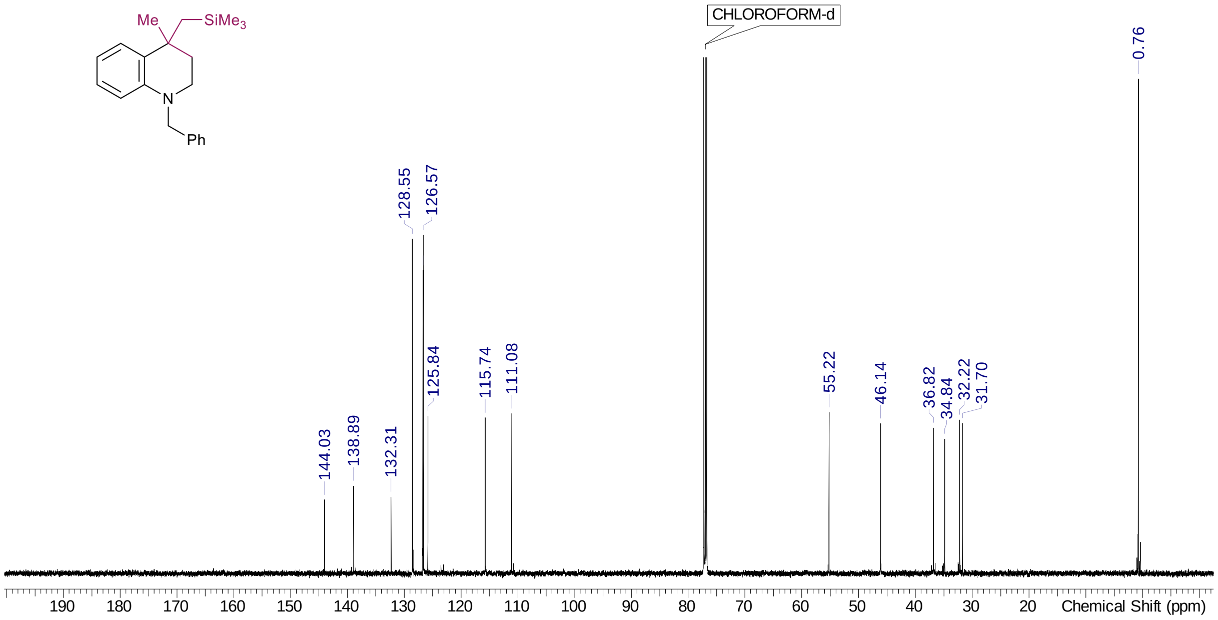

^{1}H, ^{13}C{^{1}H}-HSQC NMR ($CDCl_3$, 400, 101 MHz)

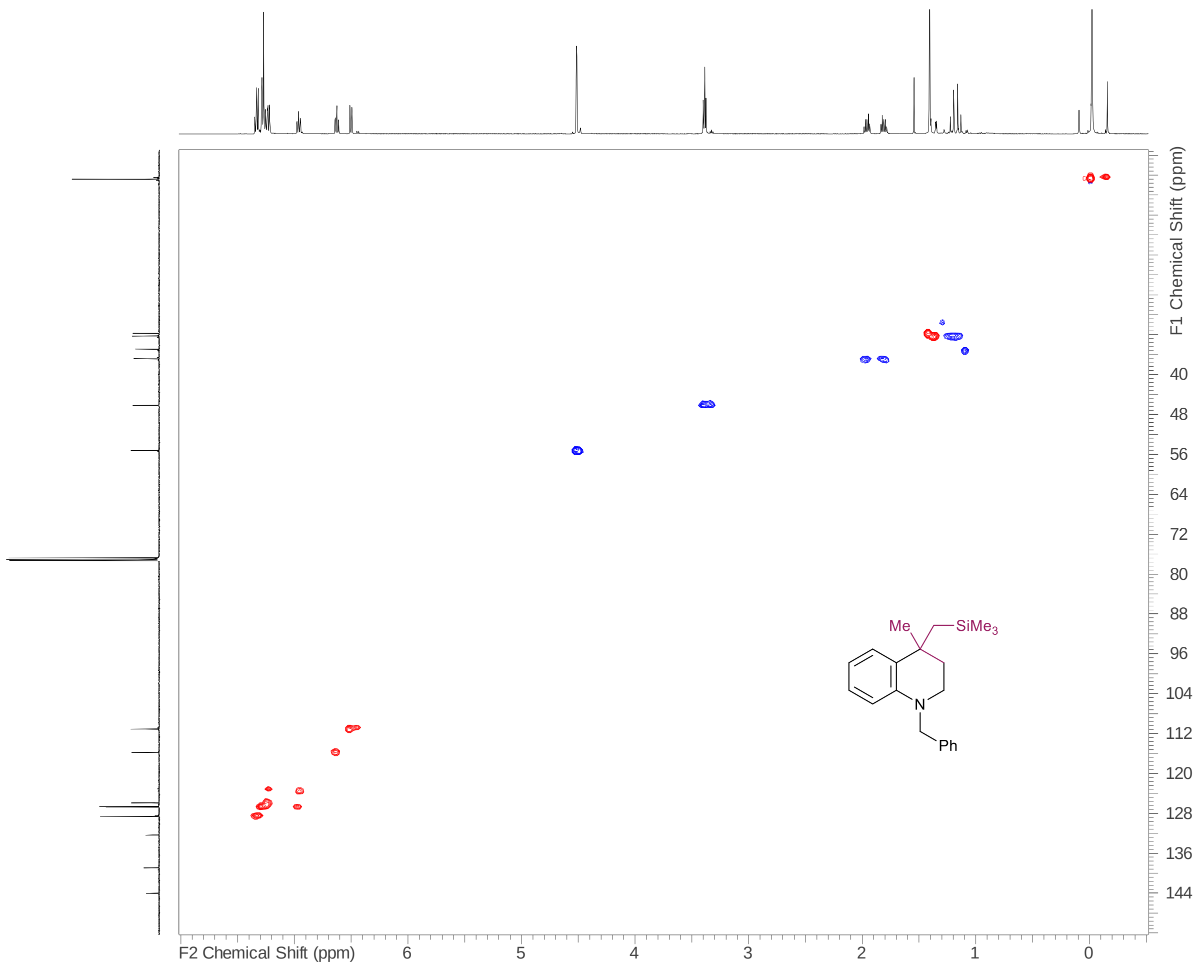

1-Benzyl-4,6-dimethyl-4-((trimethylsilyl)methyl)-1,2,3,4-tetrahydroquinoline, 3r

^{1}H NMR (400 MHz, $CDCl_3$)

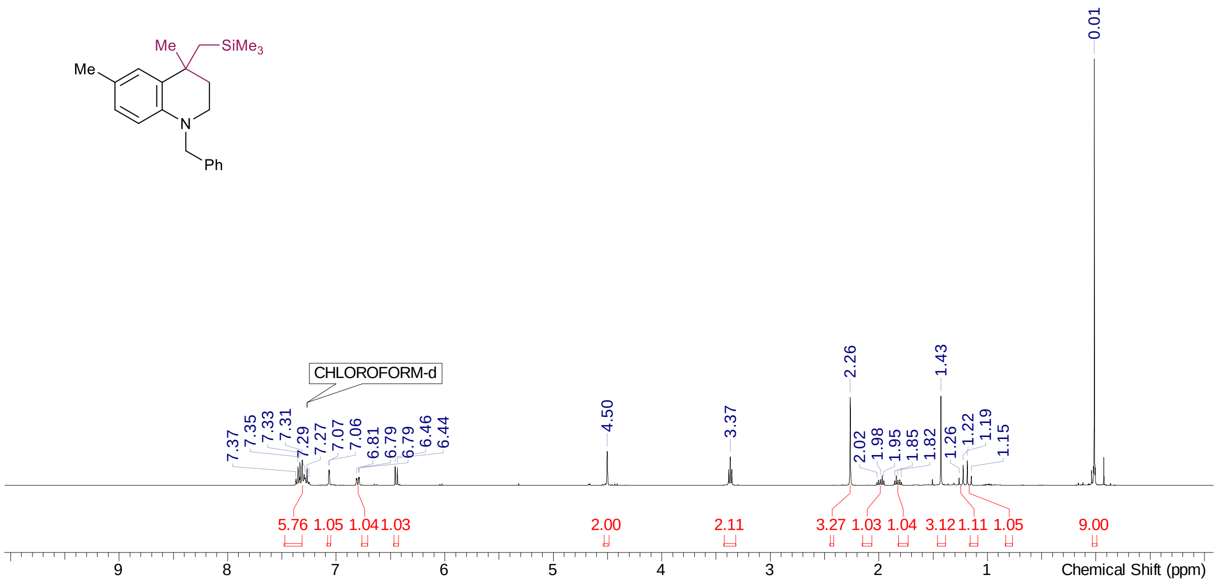

^{13}C{^{1}H} NMR ($CDCl_3$, 101 MHz)

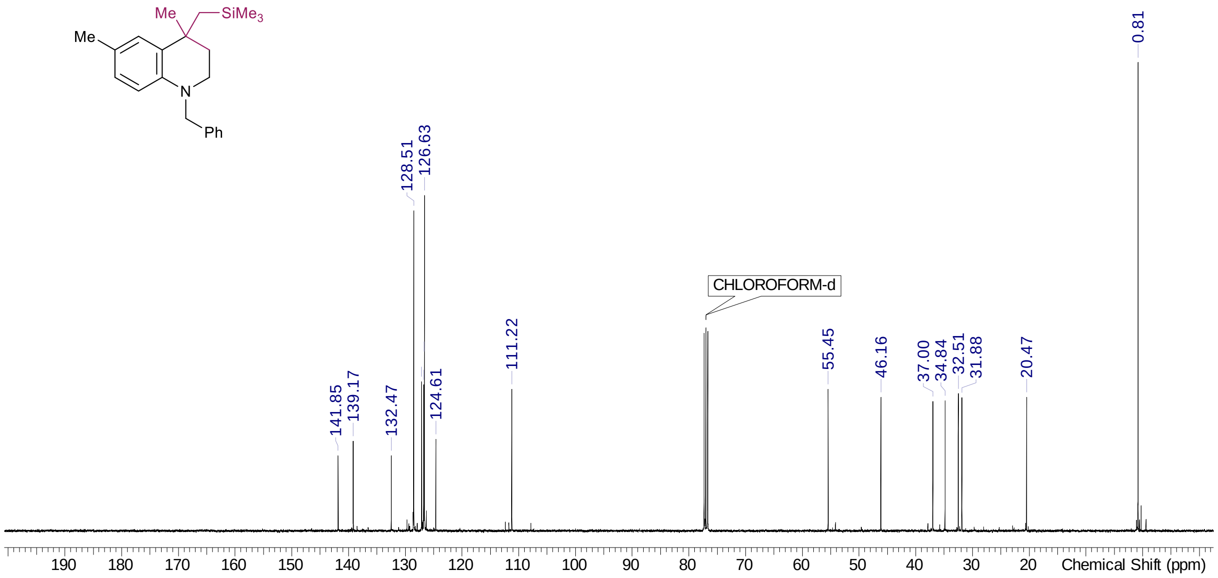

1-Benzhydryl-4-methyl-4-((trimethylsilyl)methyl)-1,2,3,4-tetrahydroquinoline, 3s

^{1}H NMR (400 MHz, $CDCl_3$)

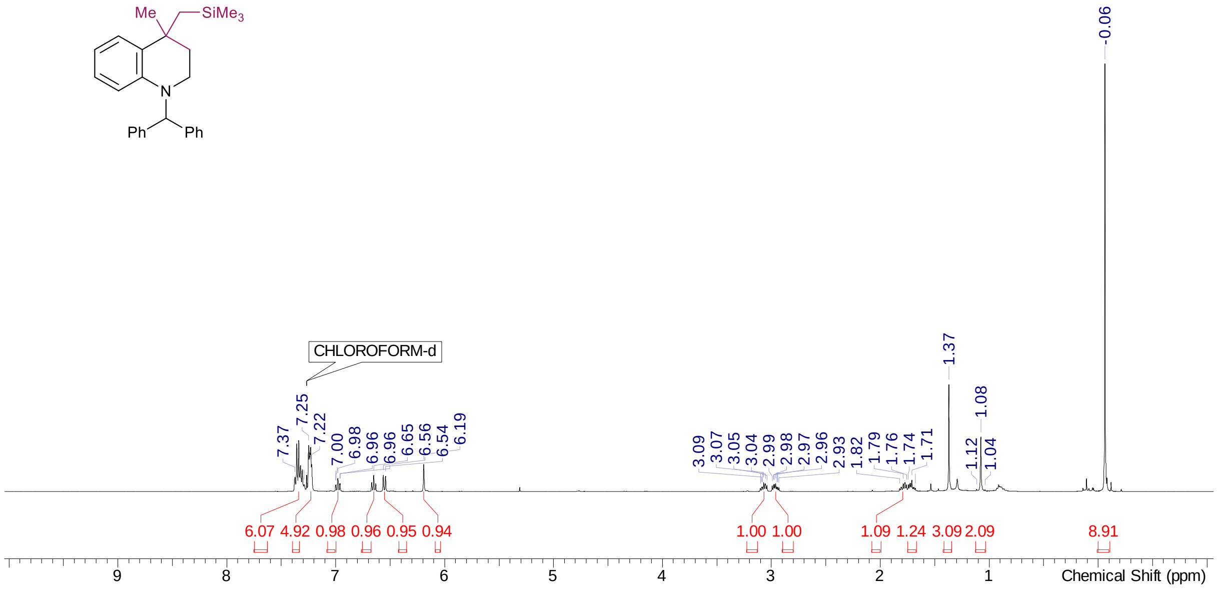

^{13}C{^{1}H} NMR ($CDCl_3$, 101 MHz)

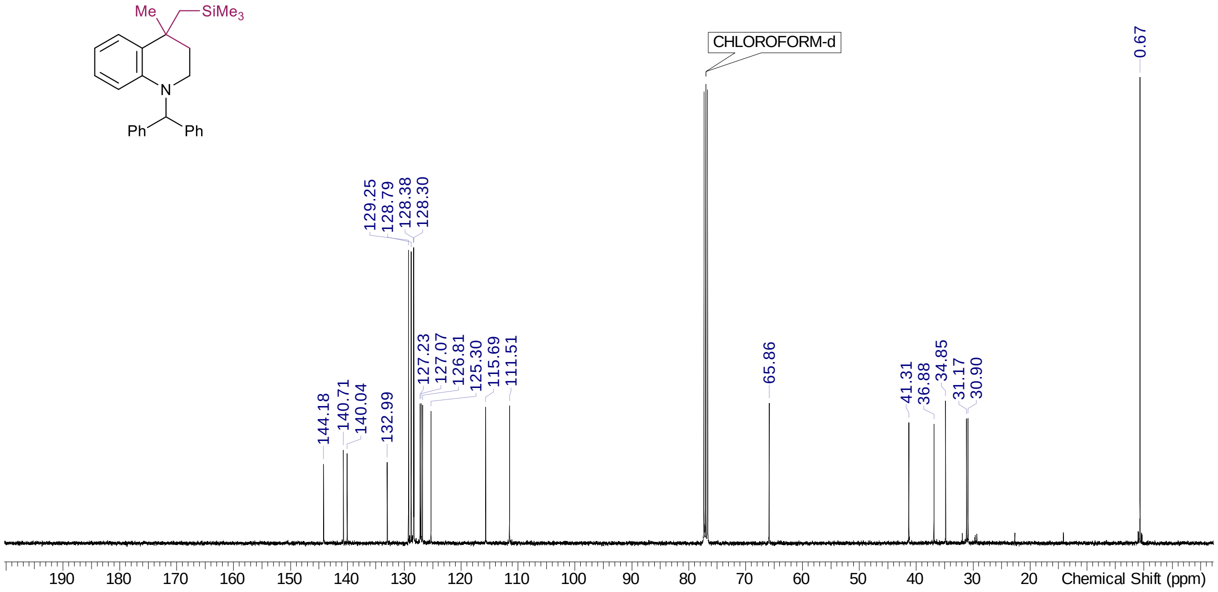

^{1}H, ^{13}C{^{1}H}-HSQC NMR ($CDCl_3$, 400, 101 MHz)

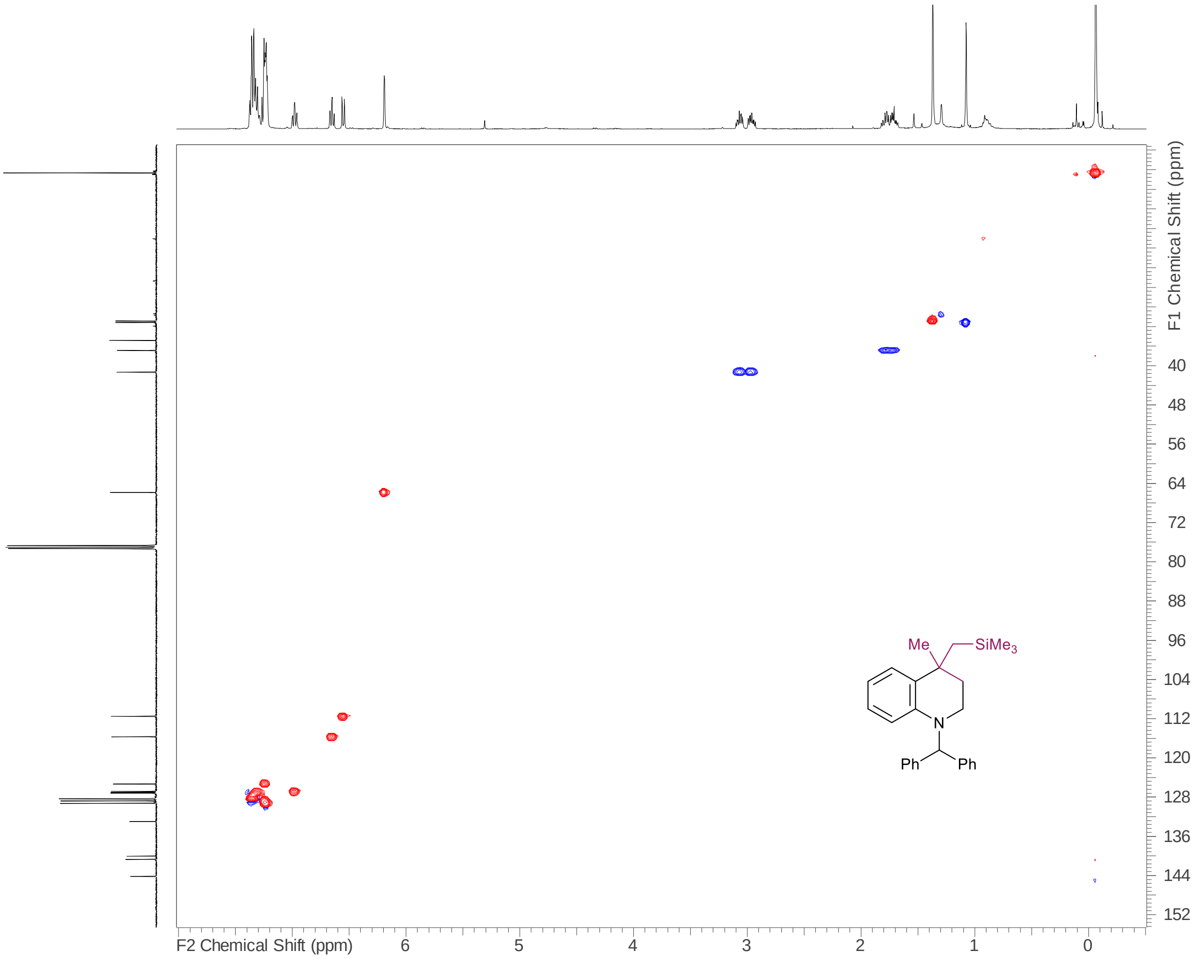

1-Benzhydryl-4,6-dimethyl-4-((trimethylsilyl)methyl)-1,2,3,4-tetrahydroquinoline, 3t

^{1}H NMR (400 MHz, $CDCl_3$)

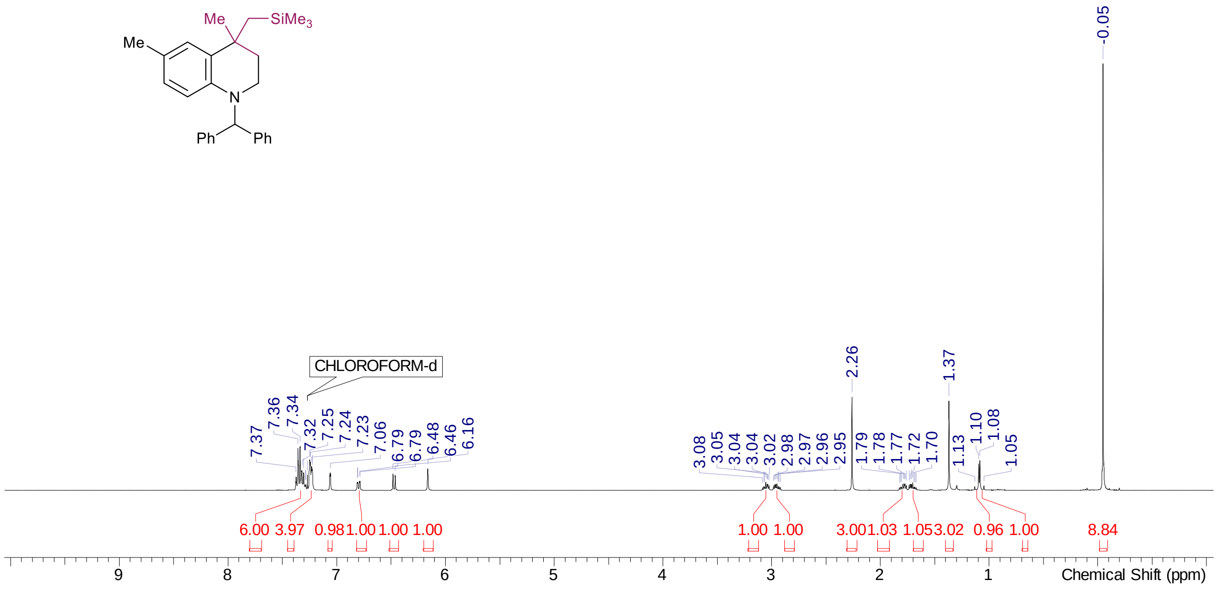

$^{13}C\{^{1}H\}$ NMR ($CDCl_3$, 101 MHz)

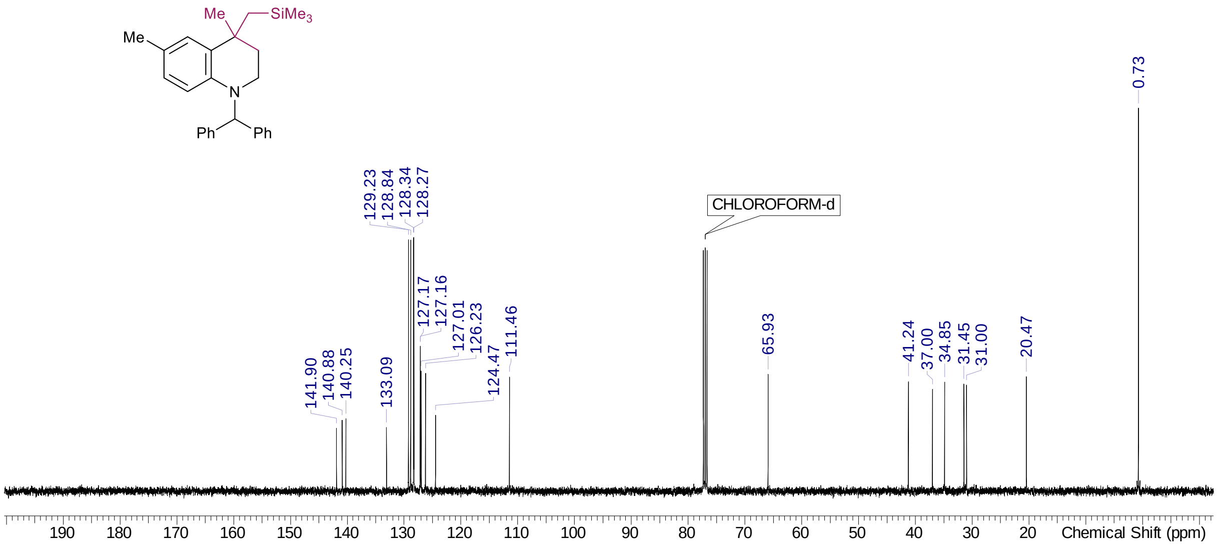

^{1}H, $^{13}C\{^{1}H\}$-HSQC NMR ($CDCl_3$, 400, 101 MHz)

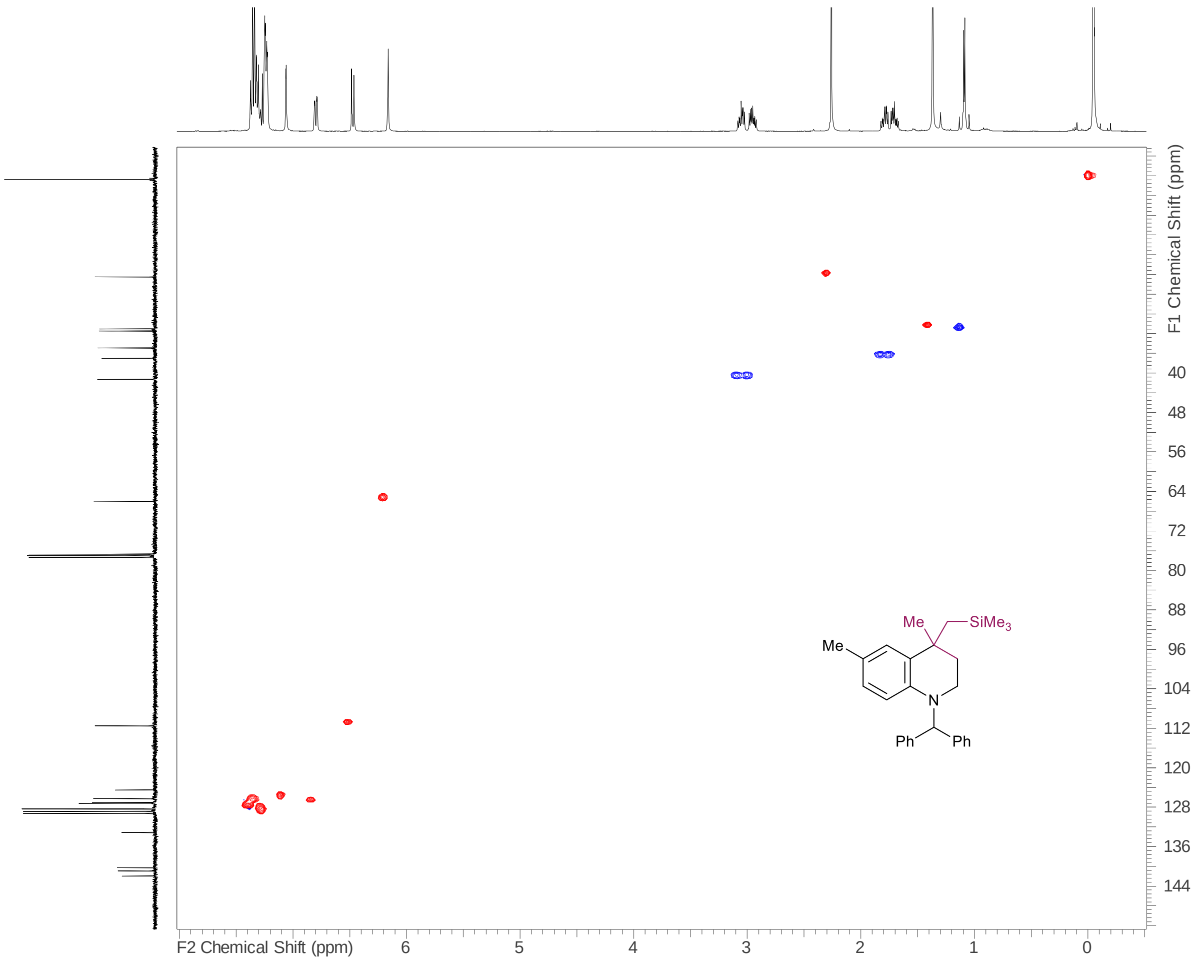

1,6-Dibenzhydryl-4-methyl-4-((trimethylsilyl)methyl)-1,2,3,4-tetrahydroquinoline, 3u

^{1}H NMR (400 MHz, $CDCl_3$)

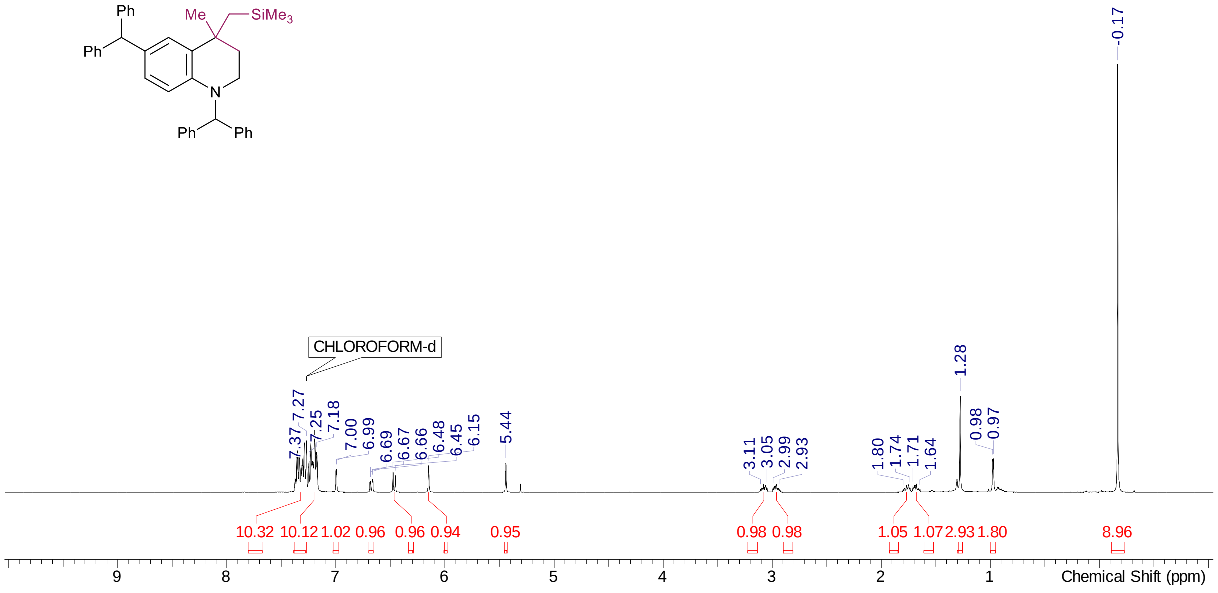

^{13}C{^{1}H} NMR ($CDCl_3$, 101 MHz)

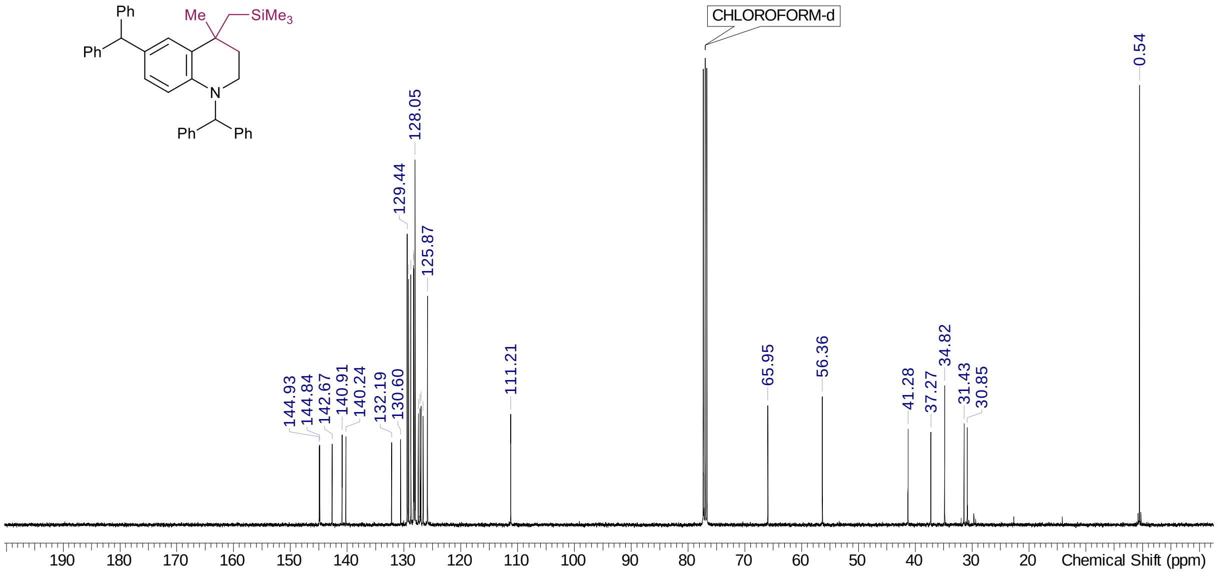

^{1}H, ^{13}C{^{1}H}-HSQC NMR ($CDCl_3$, 400, 101 MHz)

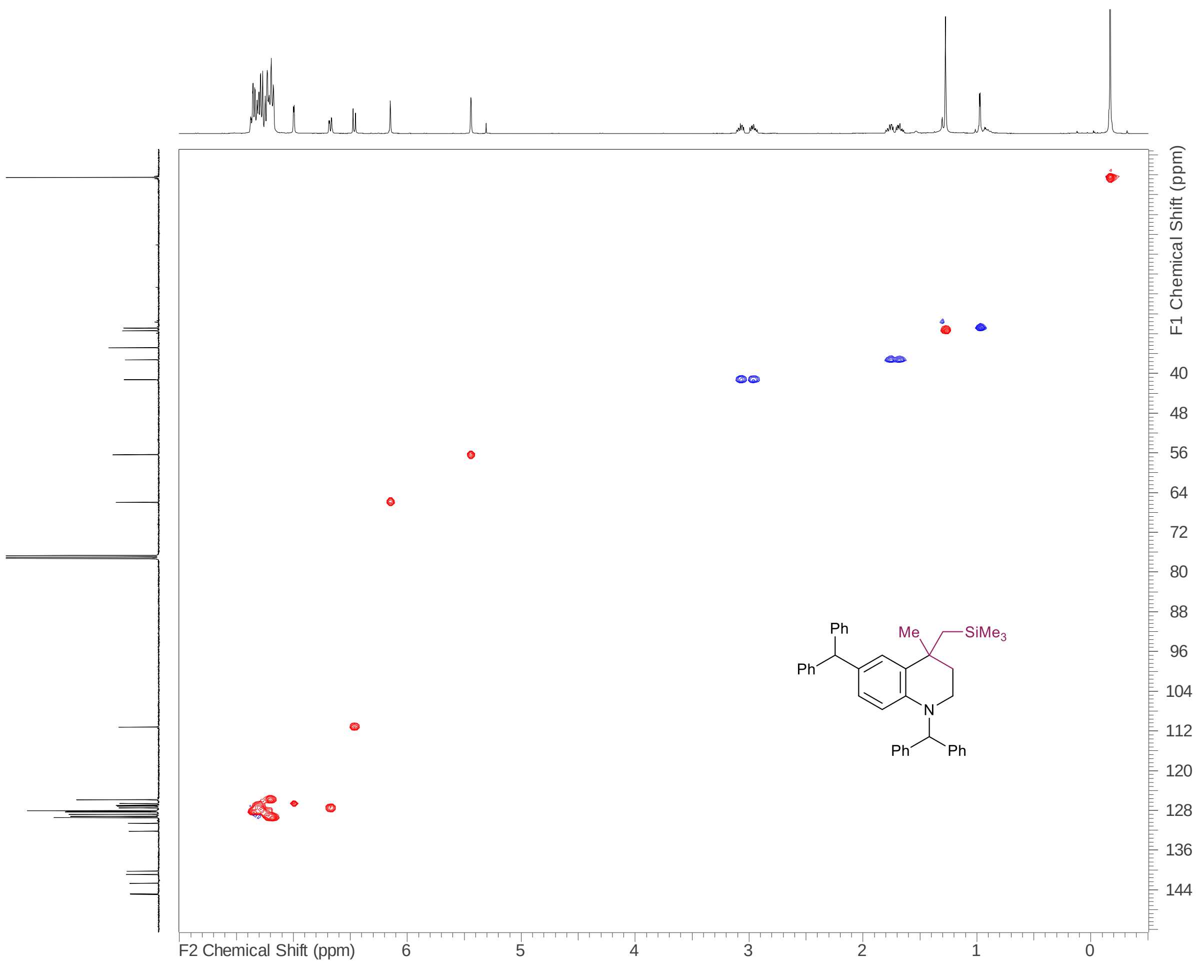

2,4,6-Trimethyl-1-(*p*-tolyl)-4-((trimethylsilyl)methyl)-1,2,3,4-tetrahydroquinoline, 3v

^{1}H NMR (400 MHz, $CDCl_3$)

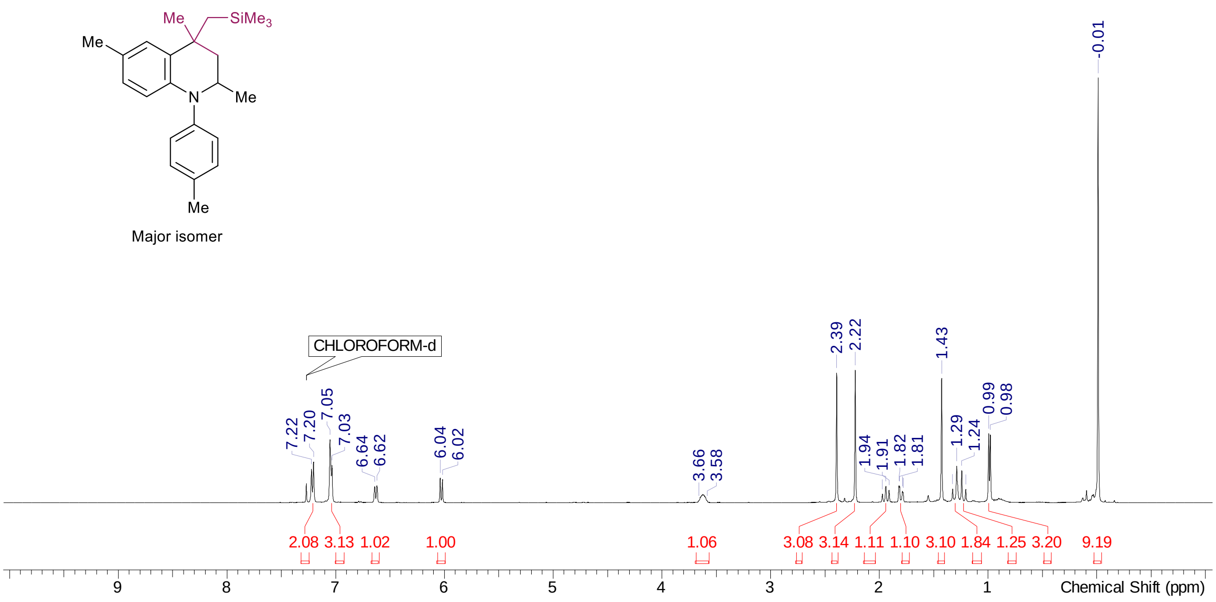

$^{13}C\{^{1}H\}$ NMR ($CDCl_3$, 101 MHz)

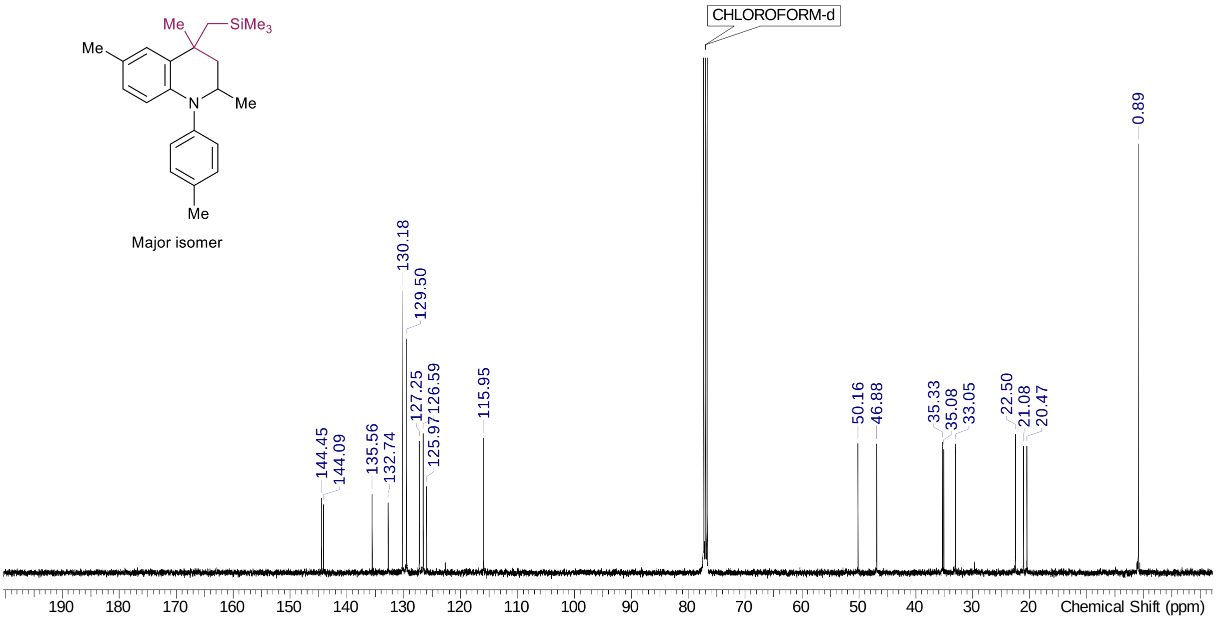

^{1}H, $^{13}C\{^{1}H\}$-HSQC NMR ($CDCl_3$, 400, 101 MHz)

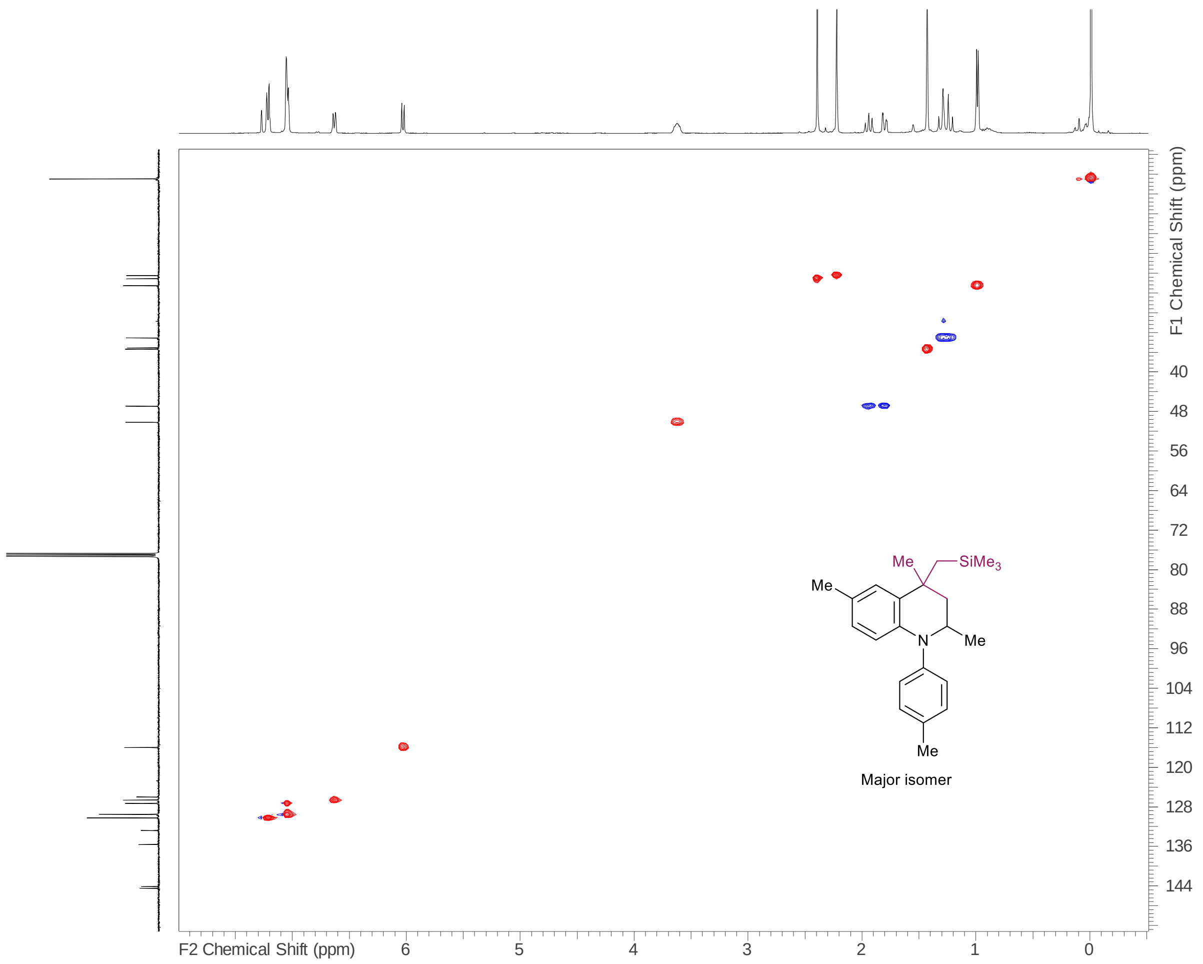

^{1}H NMR (400 MHz, $CDCl_3$)

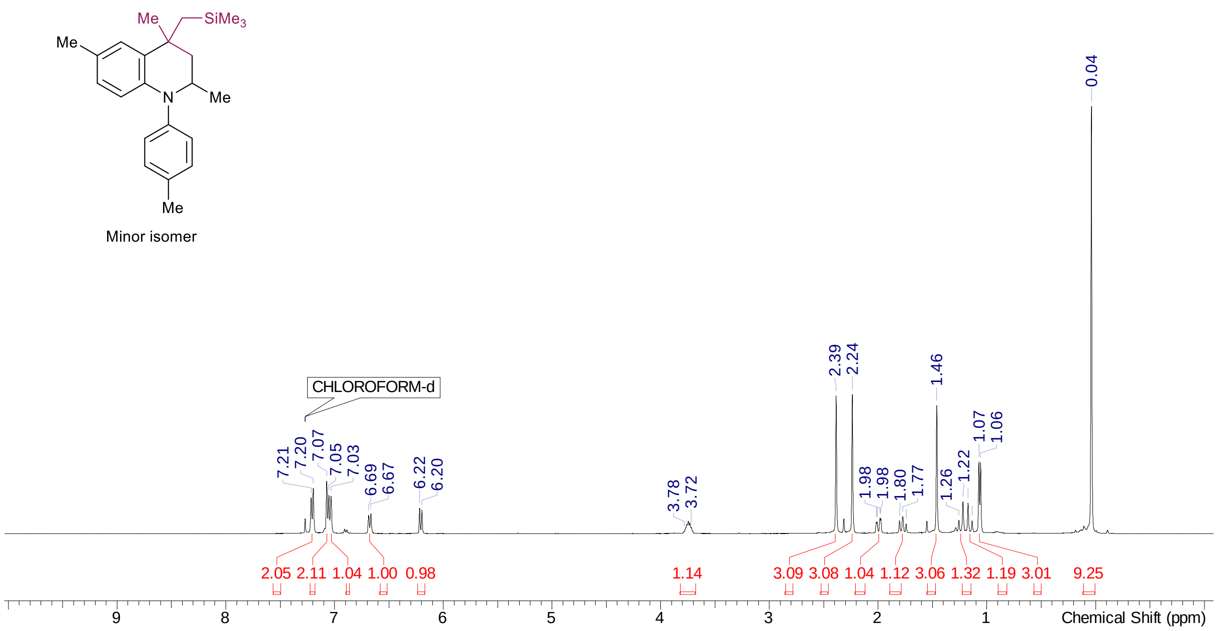

$^{13}C\{^{1}H\}$ NMR ($CDCl_3$, 101 MHz)

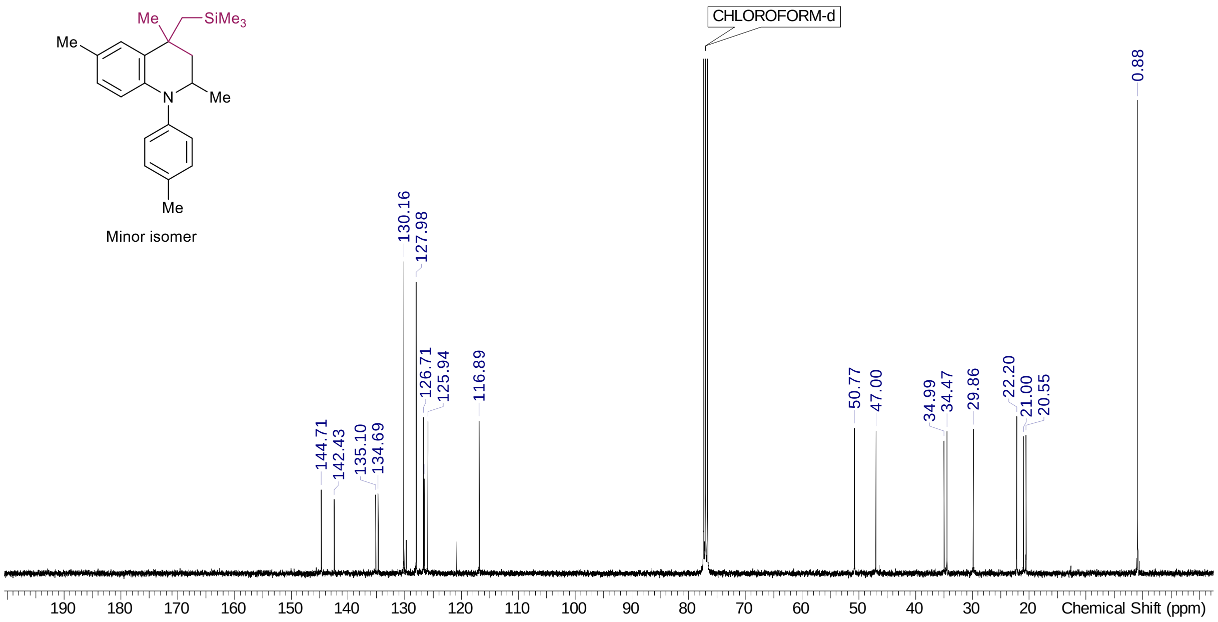

^{1}H, $^{13}C\{^{1}H\}$-HSQC NMR ($CDCl_3$, 400, 101 MHz)

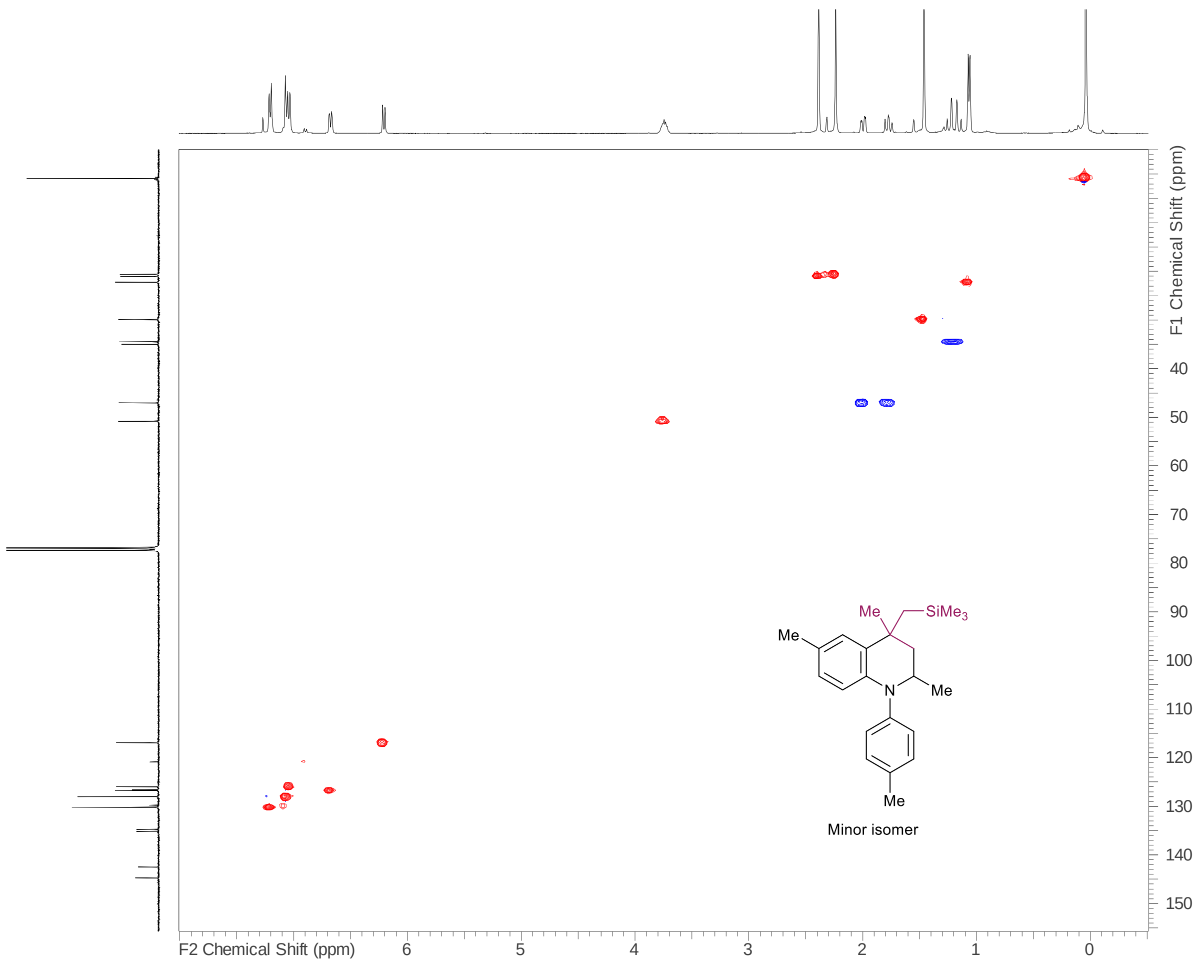

4,6-Dimethyl-2-propyl-1-(*p*-tolyl)-4-((trimethylsilyl)methyl)-1,2,3,4-tetrahydroquinoline, 3w

1:1.3 *dr*

^{1}H NMR (400 MHz, $CDCl_3$)

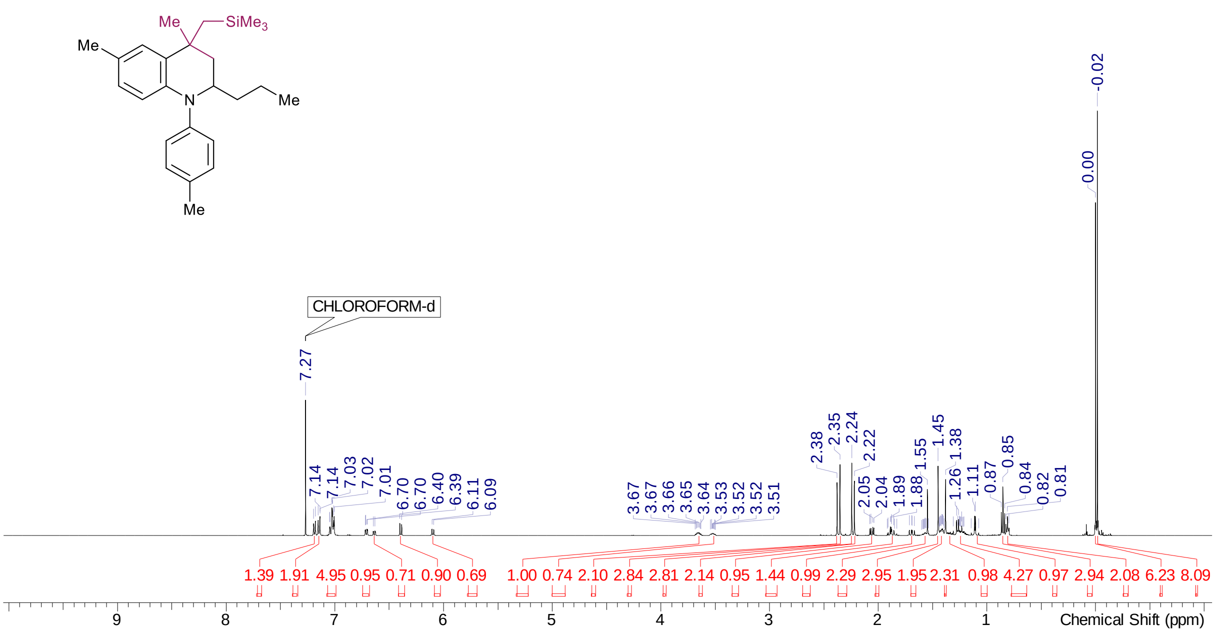

^{13}C{^{1}H} NMR ($CDCl_3$, 101 MHz)

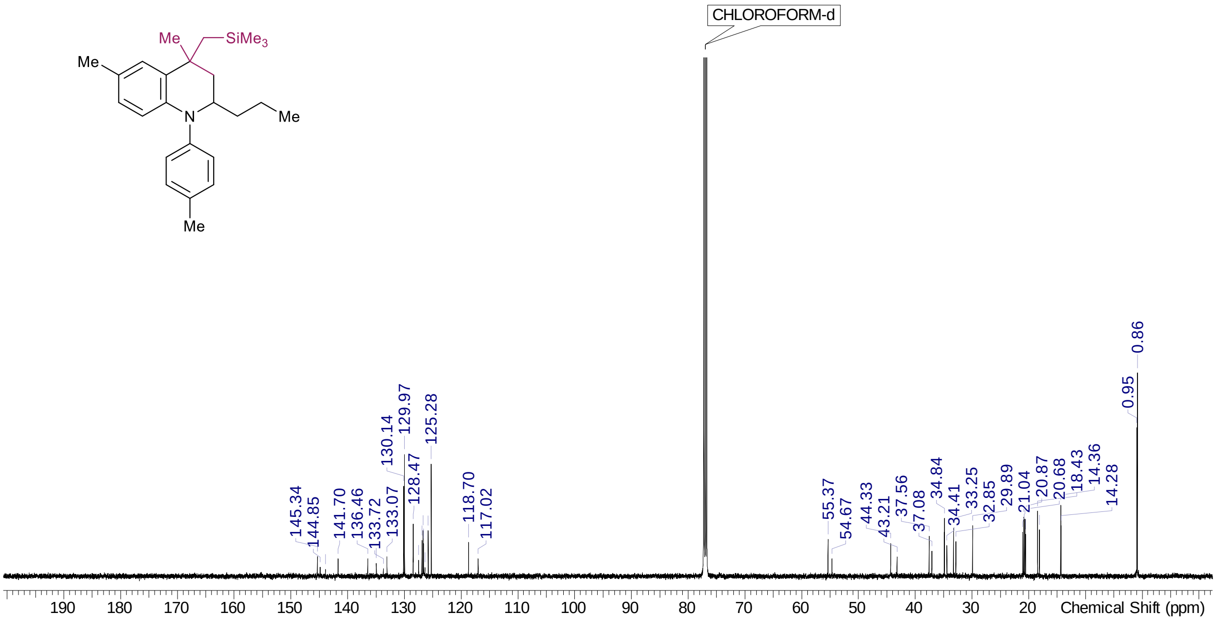

^{1}H, ^{13}C{^{1}H}-HSQC NMR (CDCl$_3$, 400, 101 MHz)

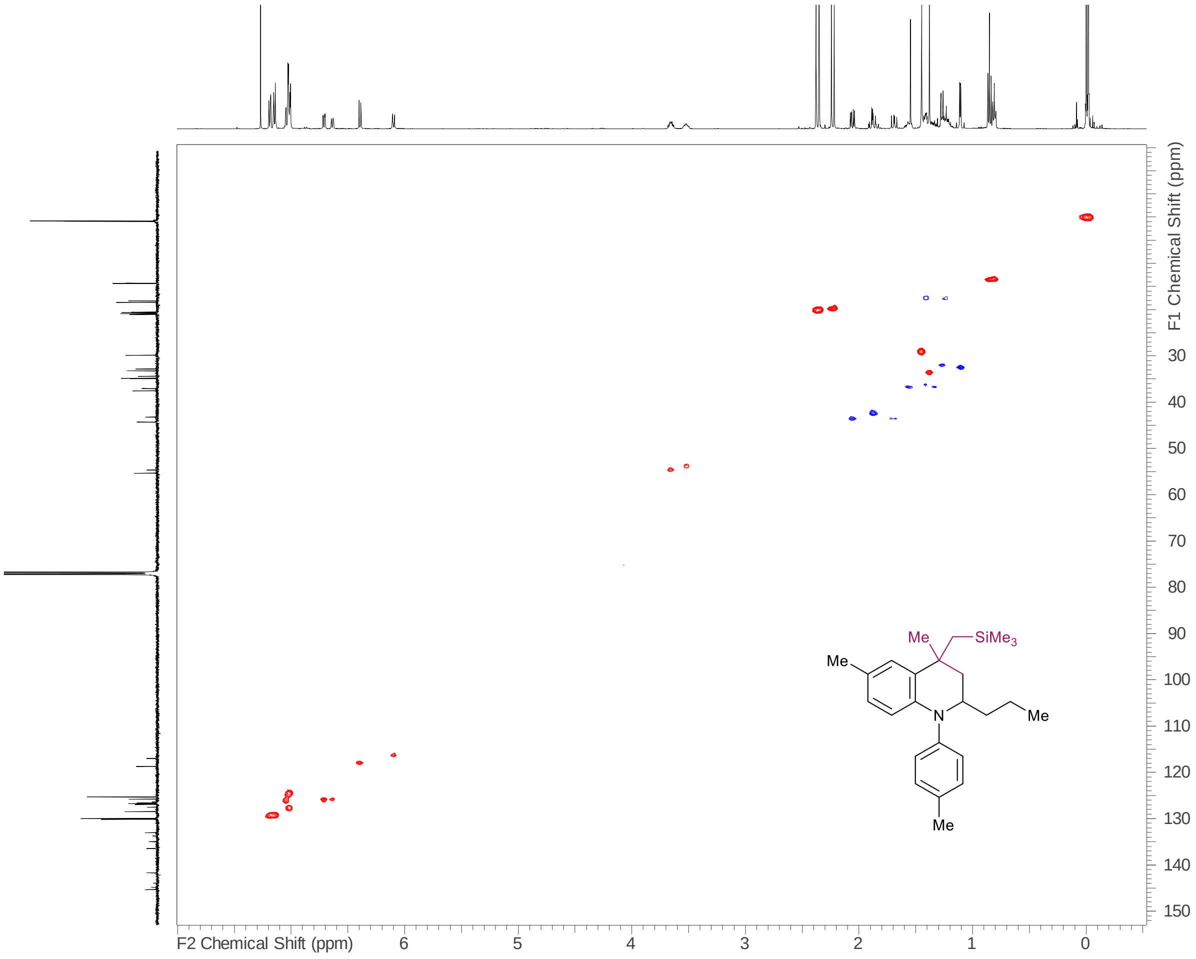

2-Cyclohexyl-4,6-dimethyl-1-(*p*-tolyl)-4-((trimethylsilyl)methyl)-1,2,3,4-tetrahydroquinoline, 3x

^{1}H NMR (400 MHz, $CDCl_3$)

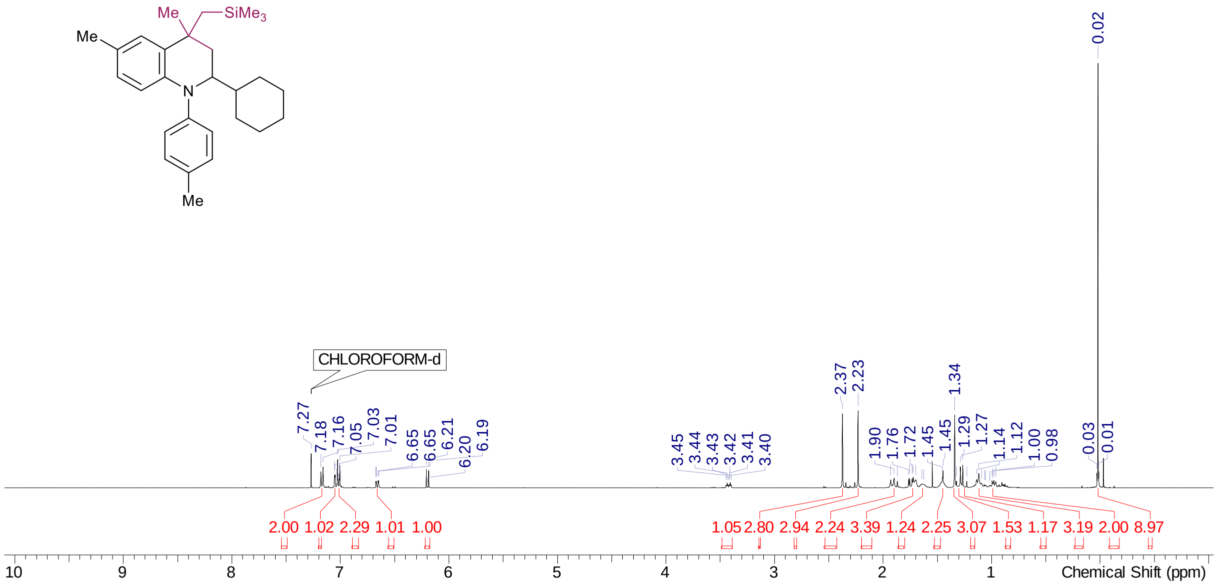

^{13}C{^{1}H} NMR ($CDCl_3$, 101 MHz)

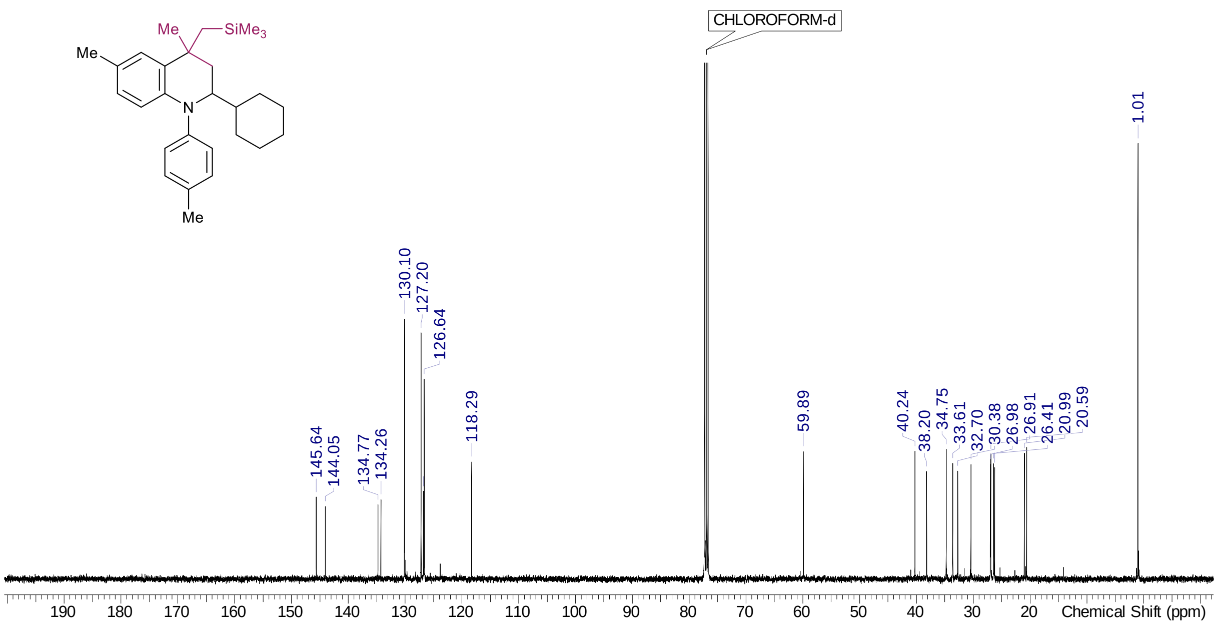

^{1}H, $^{13}C\{^{1}H\}$-HSQC NMR ($CDCl_3$, 400, 101 MHz)

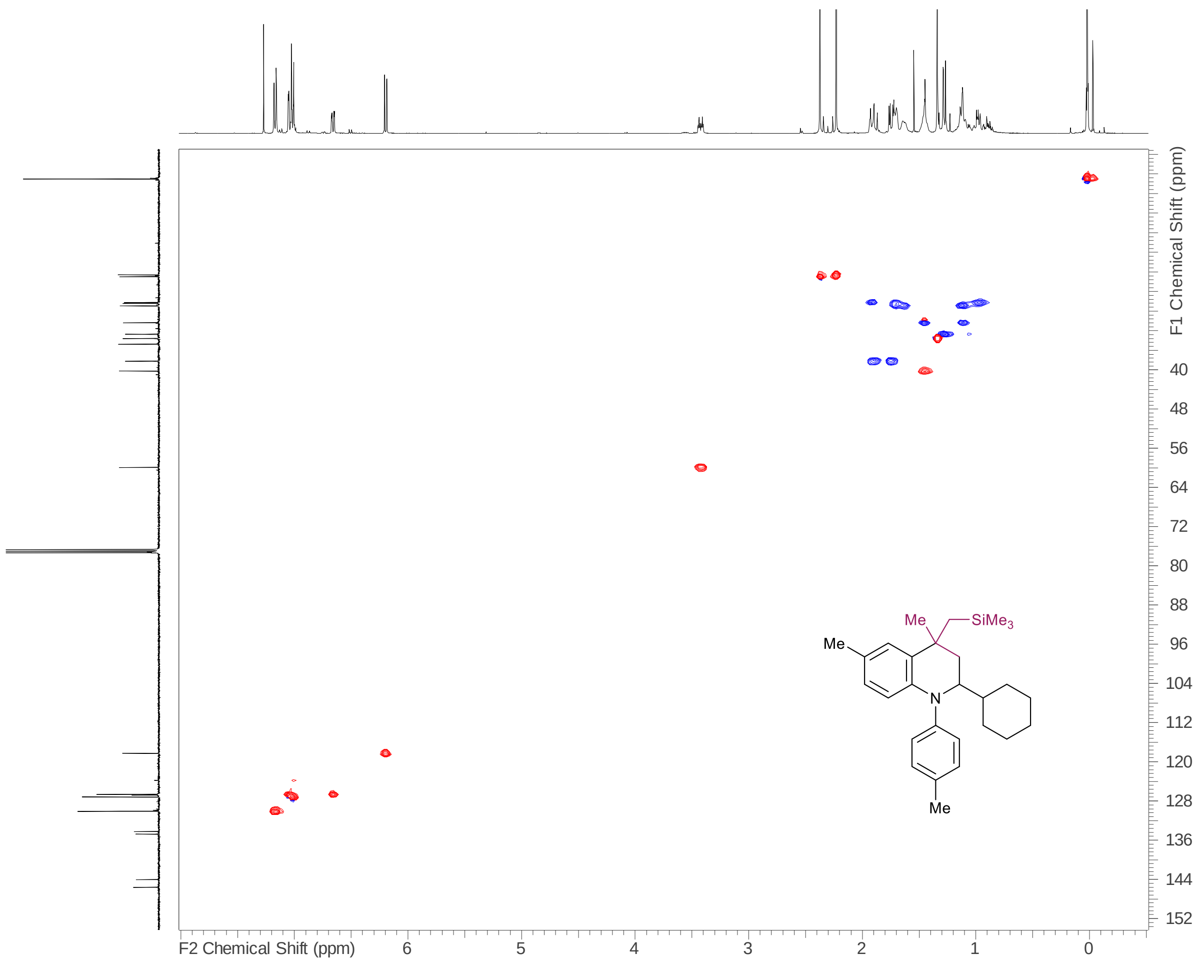

6,8-Dimethyl-6-((trimethylsilyl)methyl)-2,3,4,4a,5,6-hexahydro-1*H*-pyrido[1,2-*a*]quinoline, 3y

^{1}H NMR (400 MHz, $CDCl_3$)

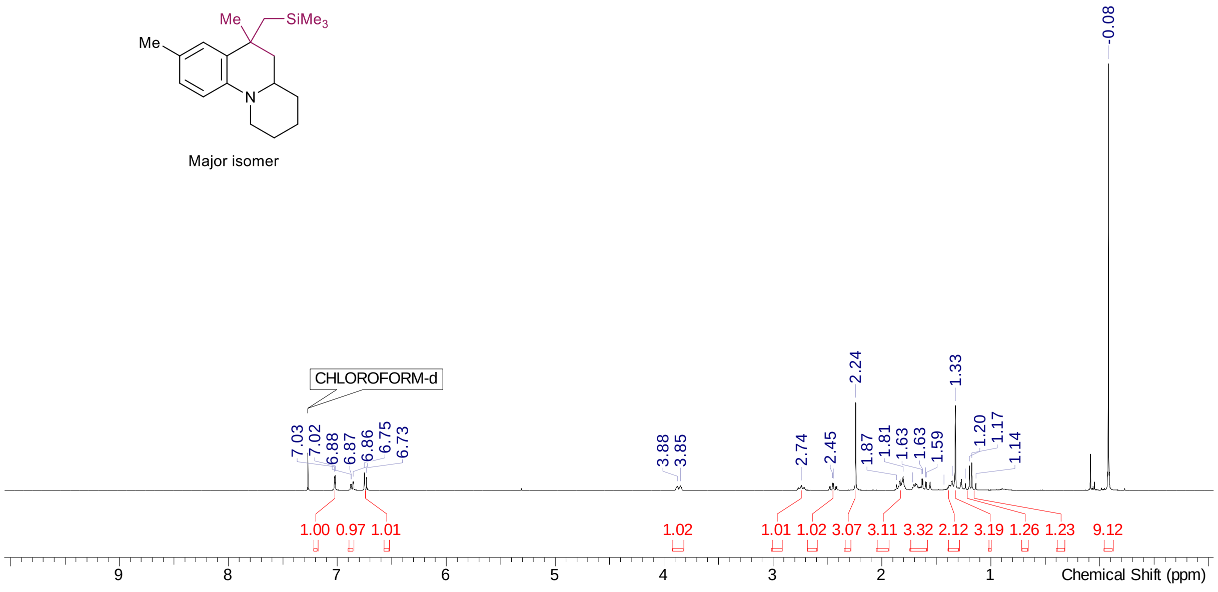

$^{13}C\{^{1}H\}$ NMR ($CDCl_3$, 101 MHz)

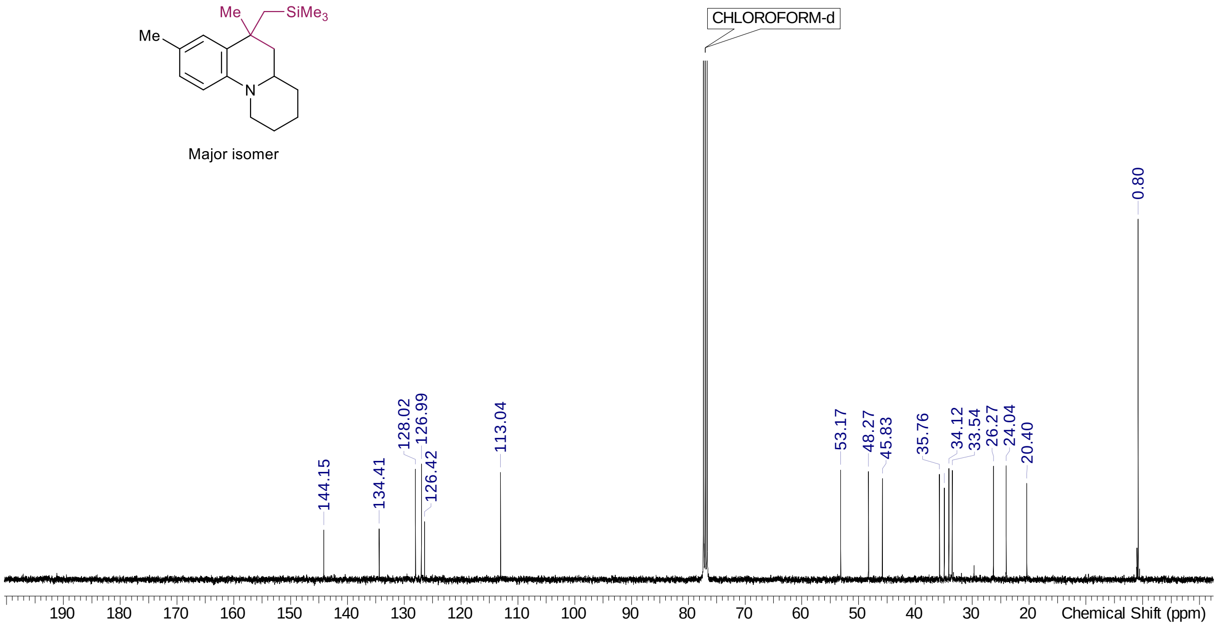

^1H, $^{13}C\{^1H\}$-HSQC NMR ($CDCl_3$, 400, 101 MHz)

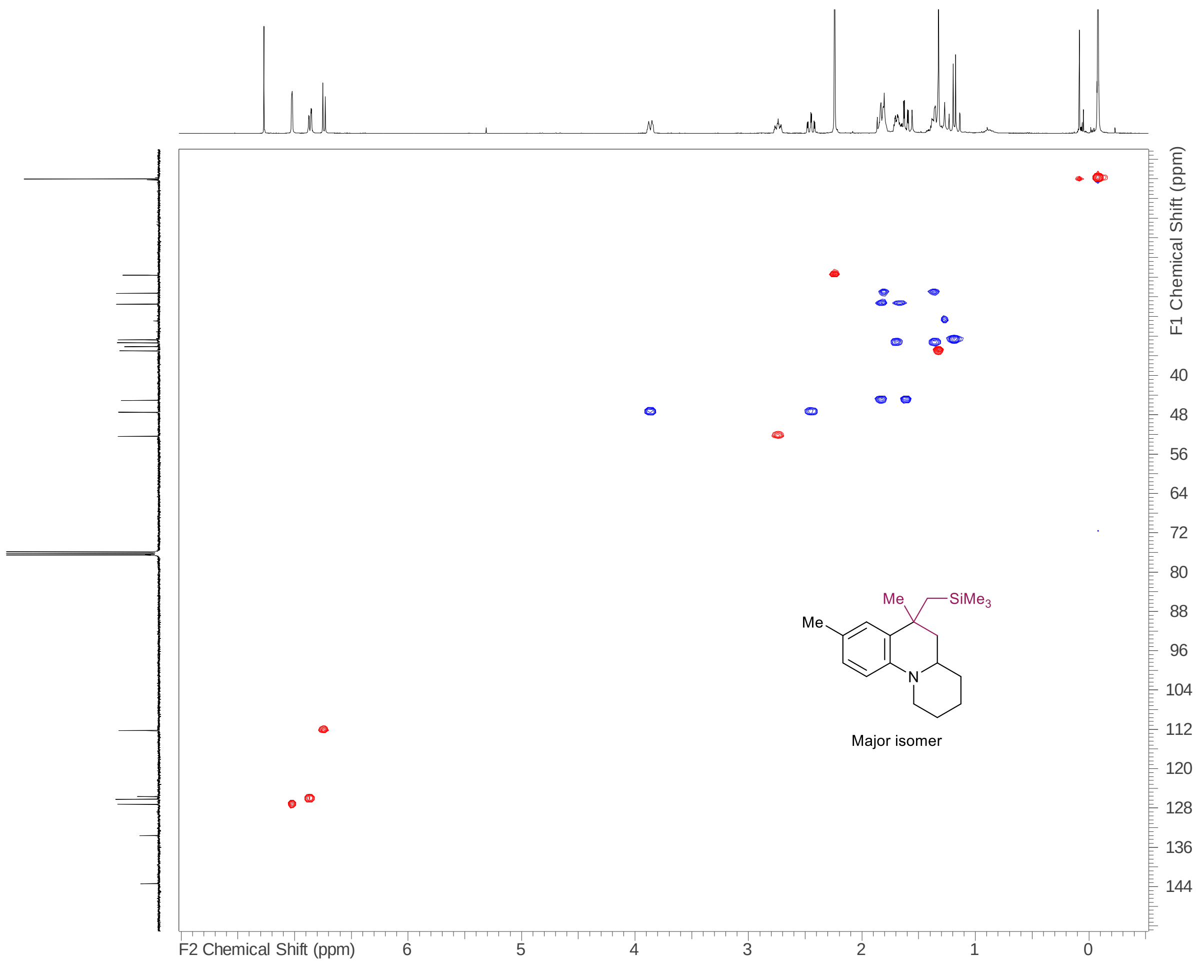

^1H NMR (400 MHz, $CDCl_3$)

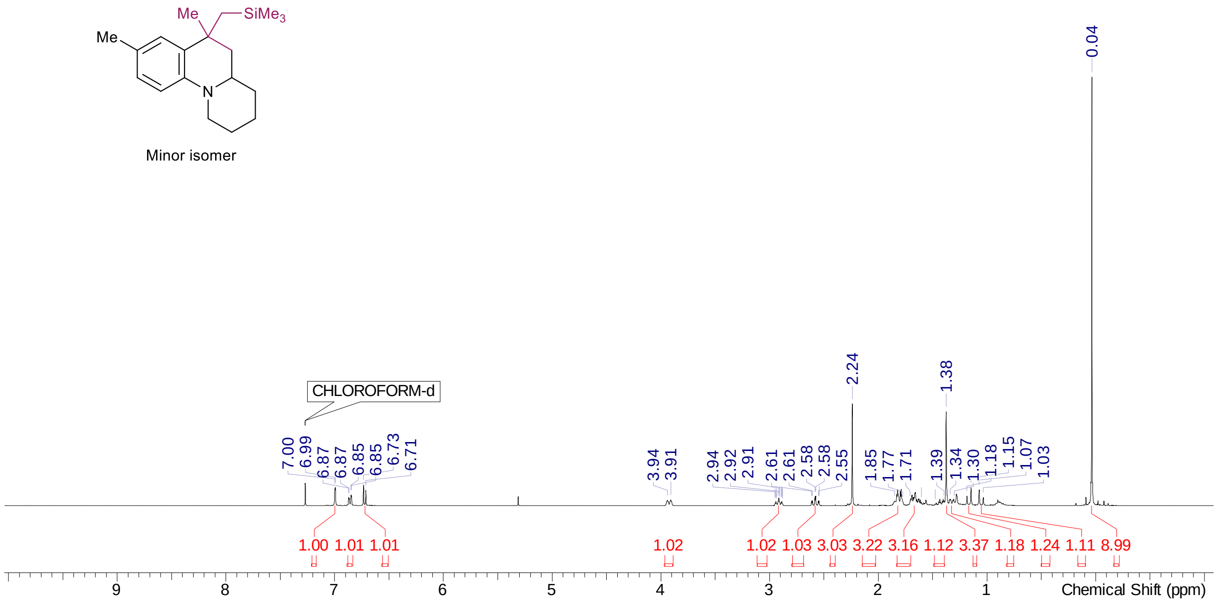

$^{13}C\{^1H\}$ NMR ($CDCl_3$, 101 MHz)

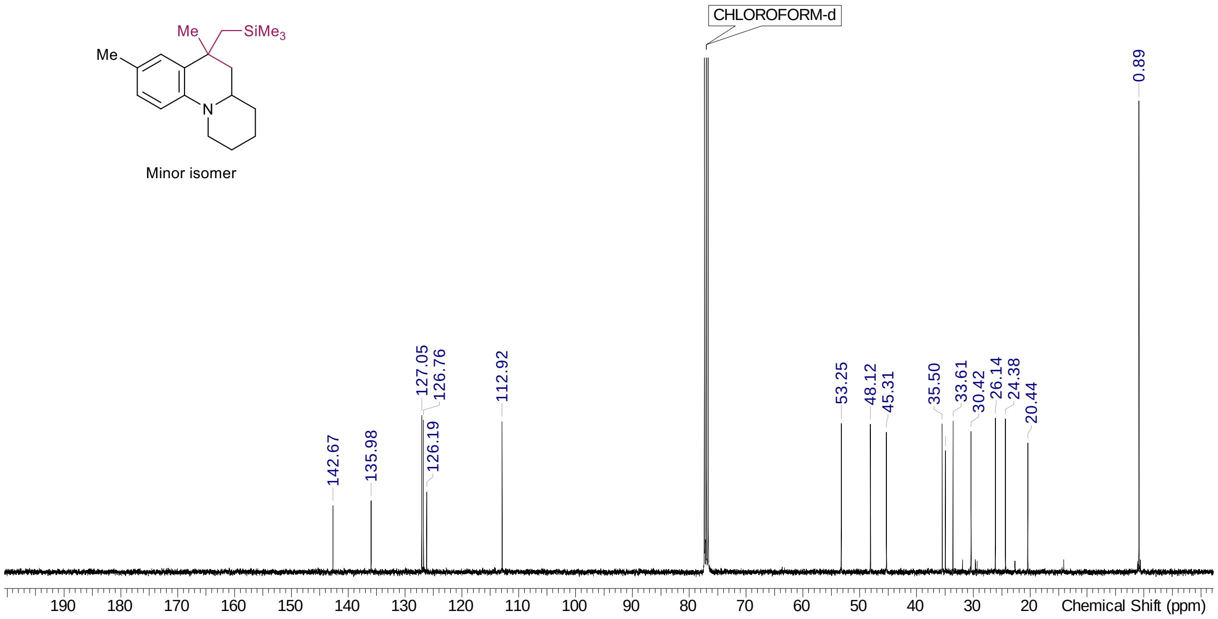

^{1}H, $^{13}C\{^{1}H\}$-HSQC NMR ($CDCl_3$, 400, 101 MHz)

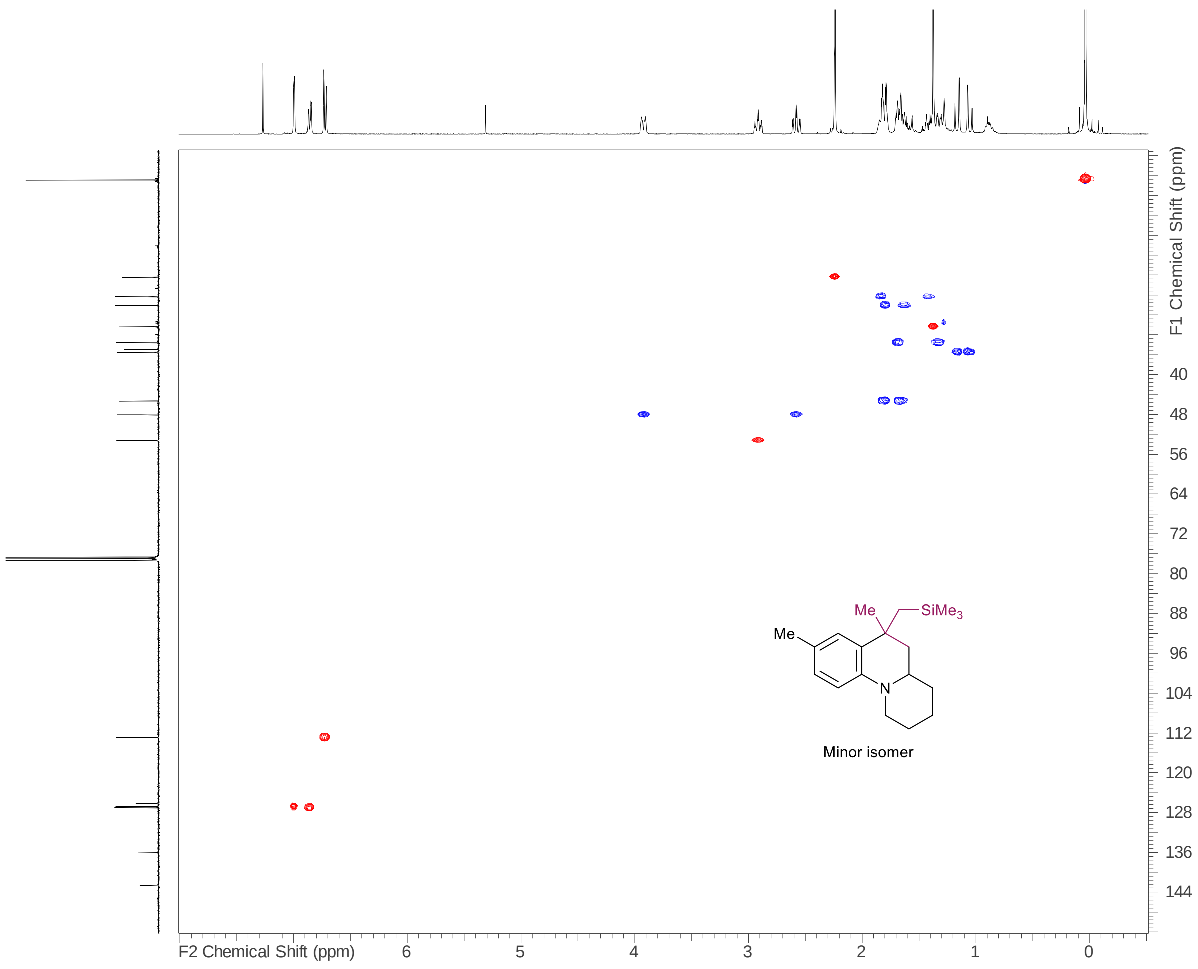

3,5-Dimethyl-5-((trimethylsilyl)methyl)-5,6,6a,7,8,9,10,11-octahydroazepino[1,2-*a*]quinoline, 3z

^{1}H NMR (400 MHz, $CDCl_3$)

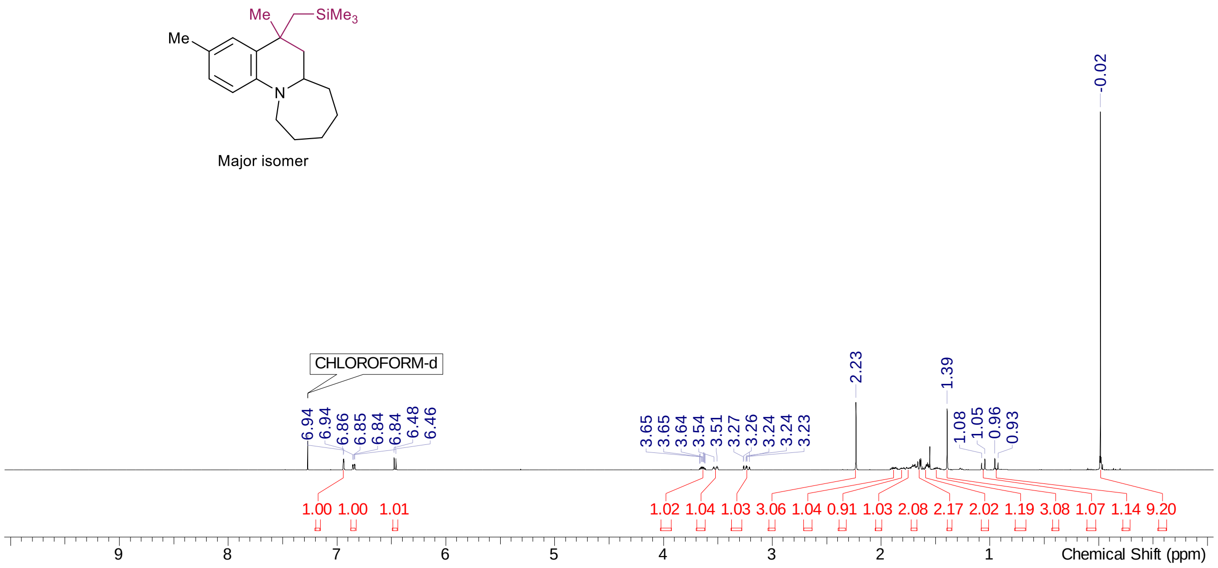

^{13}C{^{1}H} NMR ($CDCl_3$, 101 MHz)

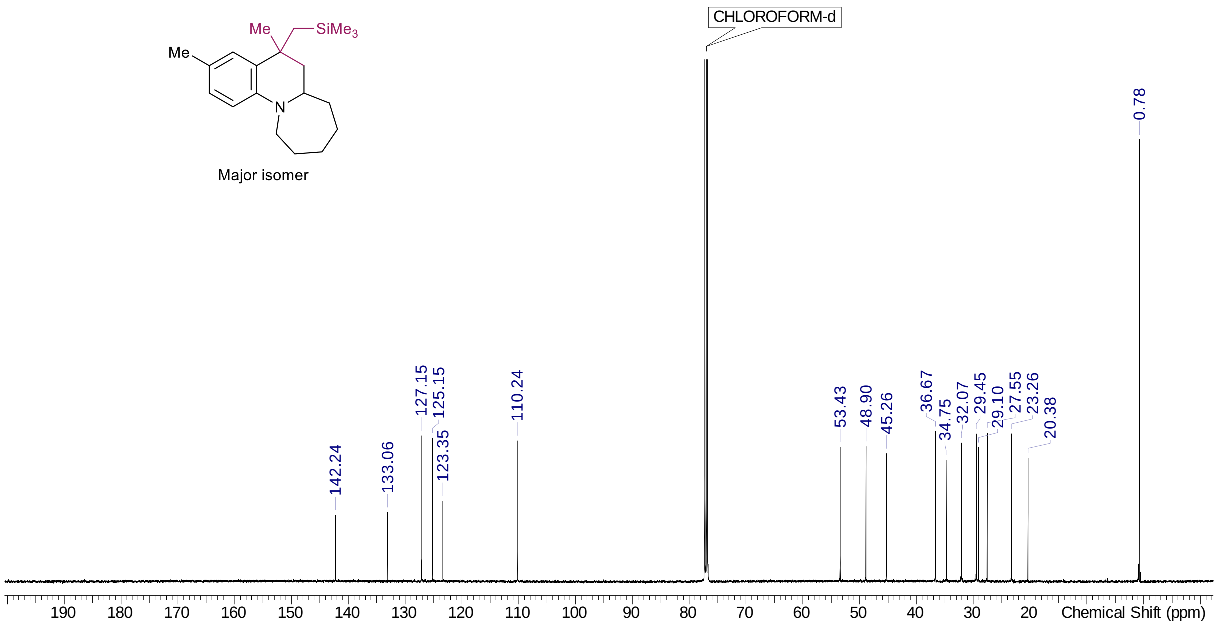

^{1}H, $^{13}C\{^{1}H\}$-HSQC NMR ($CDCl_3$, 400, 101 MHz)

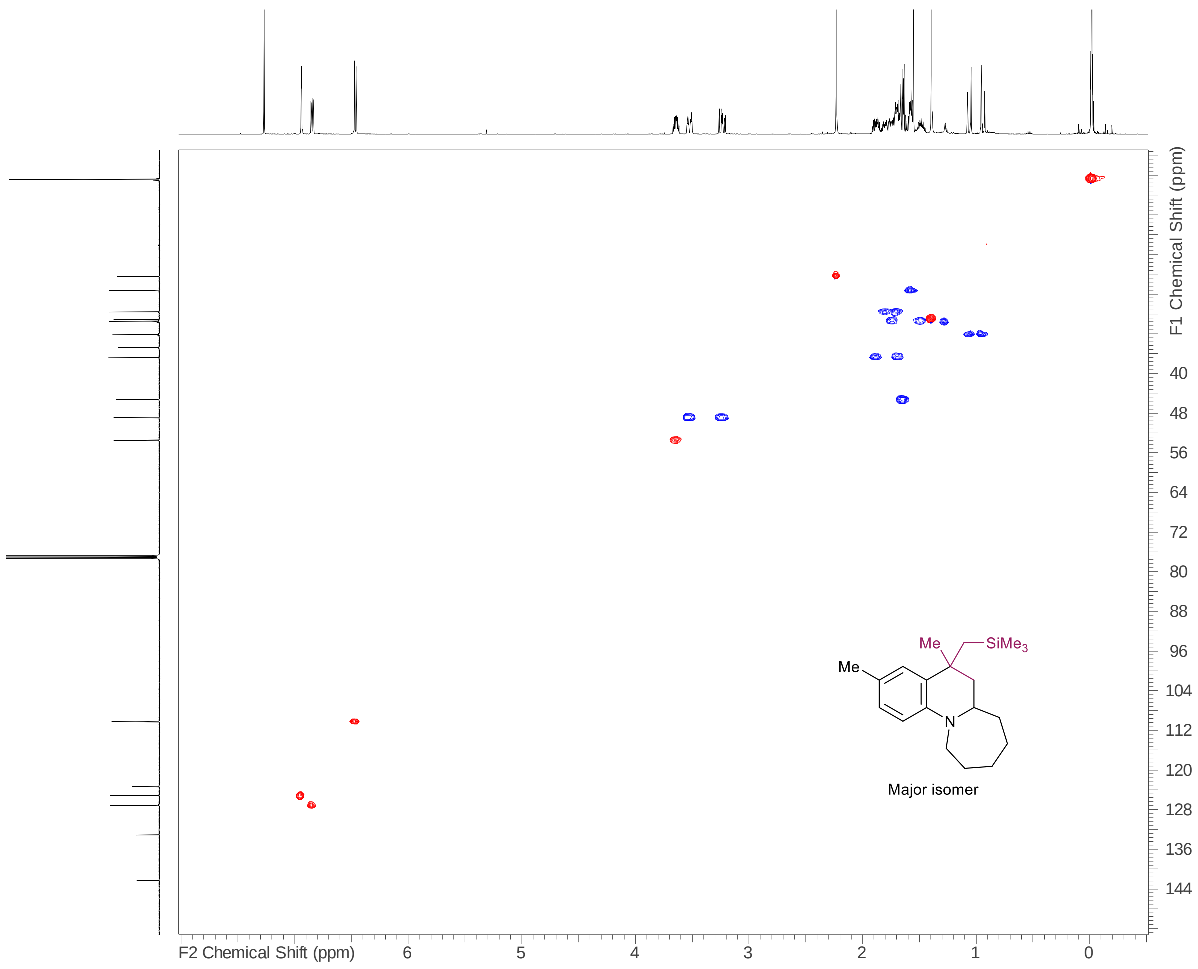

^{1}H NMR (400 MHz, $CDCl_3$)

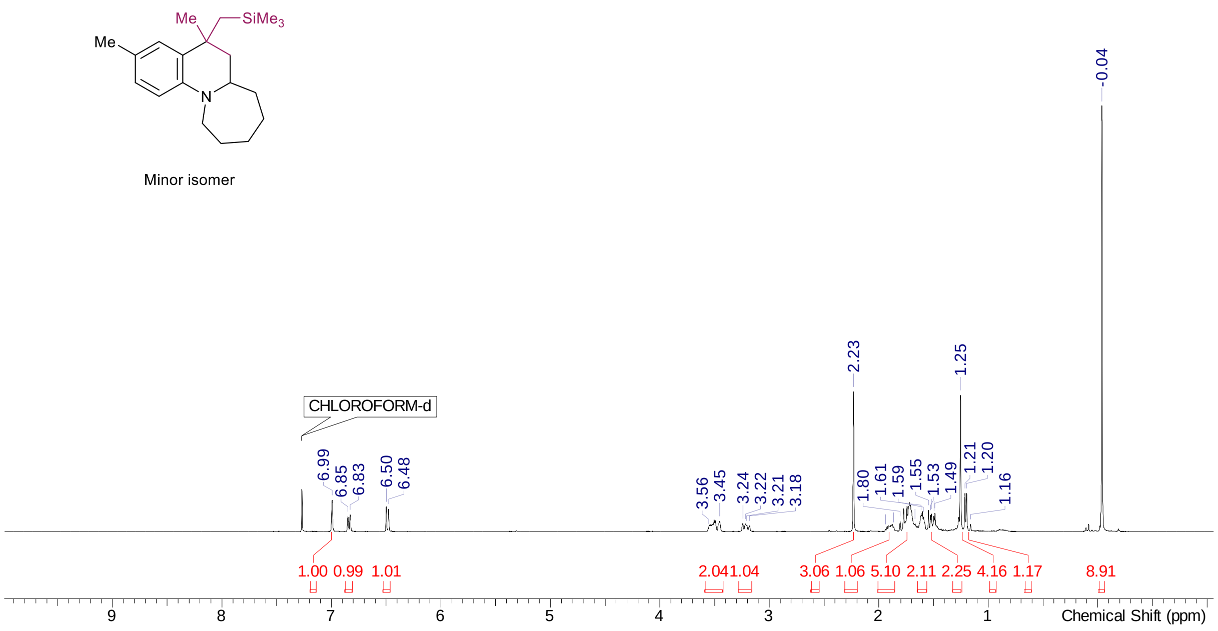

$^{13}C\{^{1}H\}$ NMR ($CDCl_3$, 101 MHz)

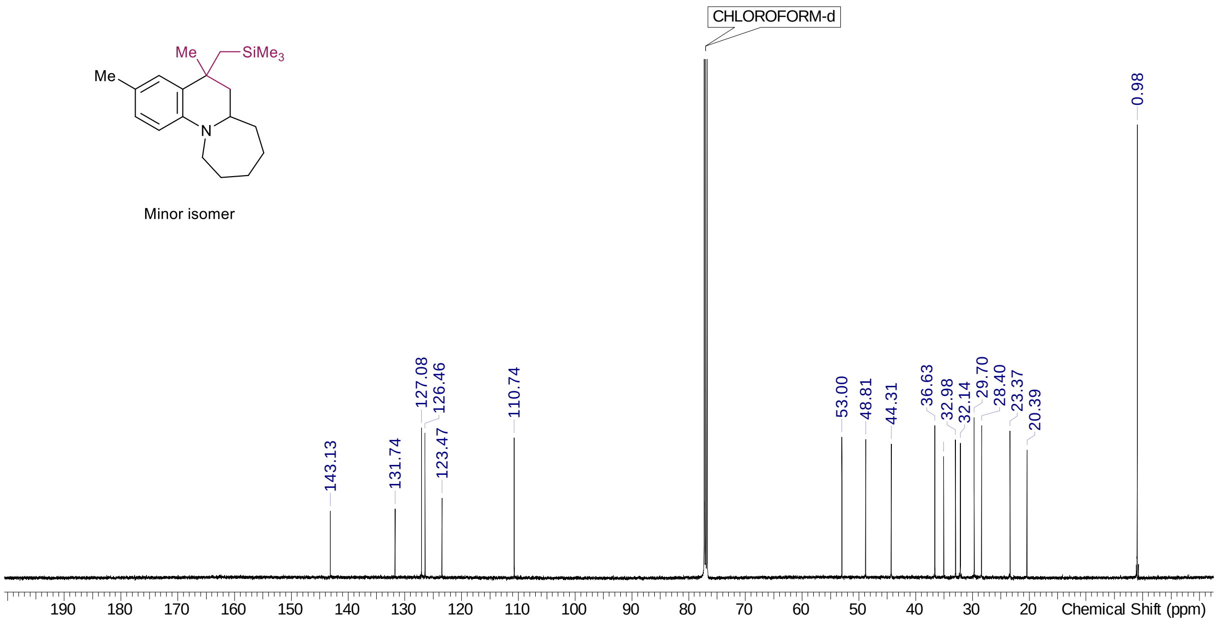

^{1}H, $^{13}C\{^{1}H\}$-HSQC NMR ($CDCl_3$, 400, 101 MHz)

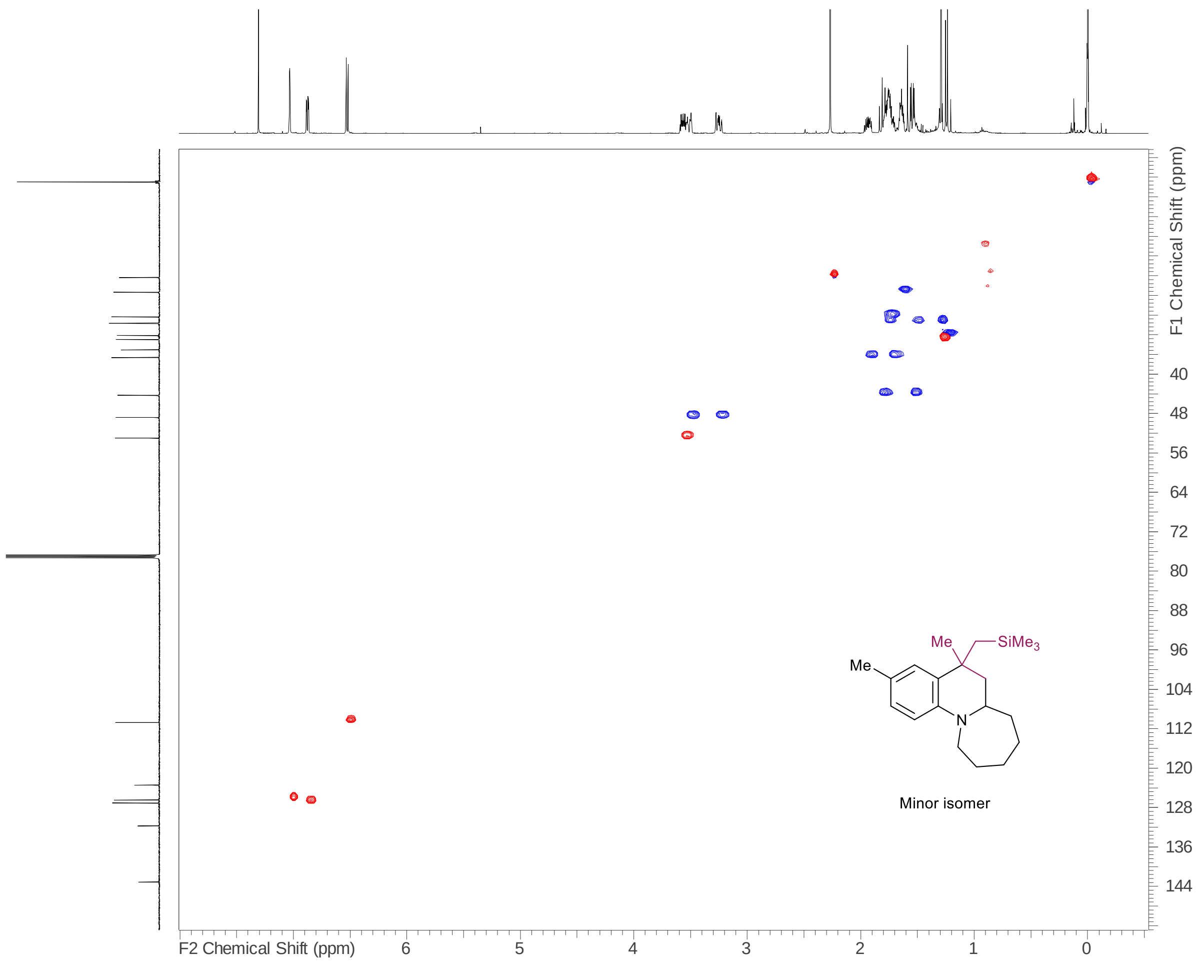

5,7-Dimethyl-5-((trimethylsilyl)methyl)-4,5-dihydropyrrolo[1,2-*a*]quinoline, 3aa

^{1}H NMR (400 MHz, $CDCl_3$)

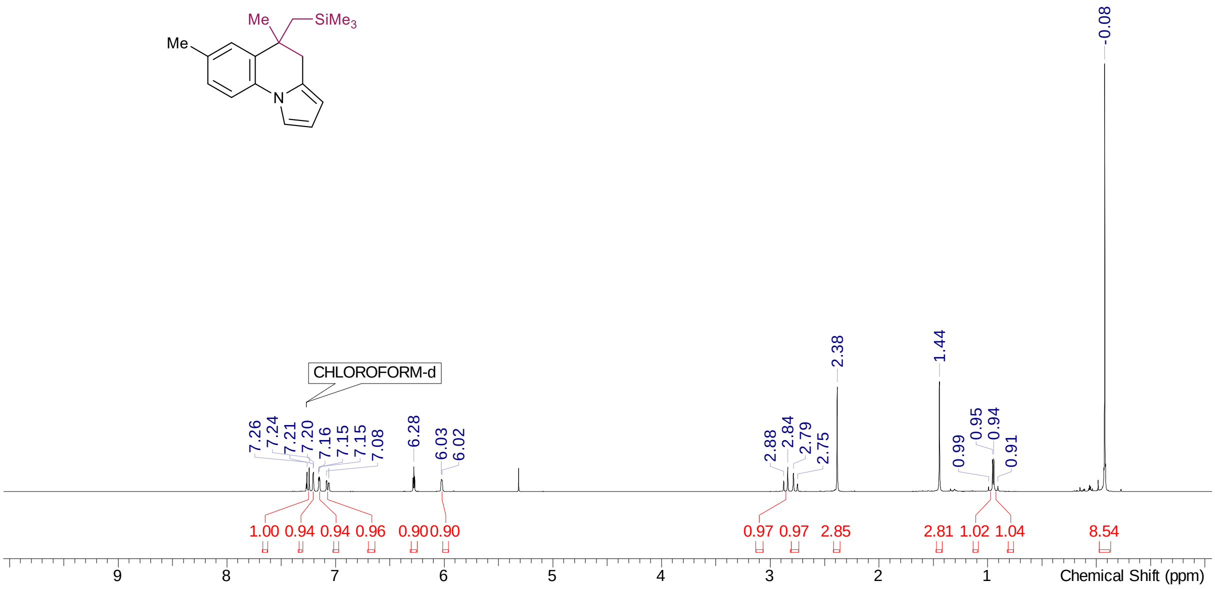

$^{13}C\{^{1}H\}$ NMR ($CDCl_3$, 101 MHz)

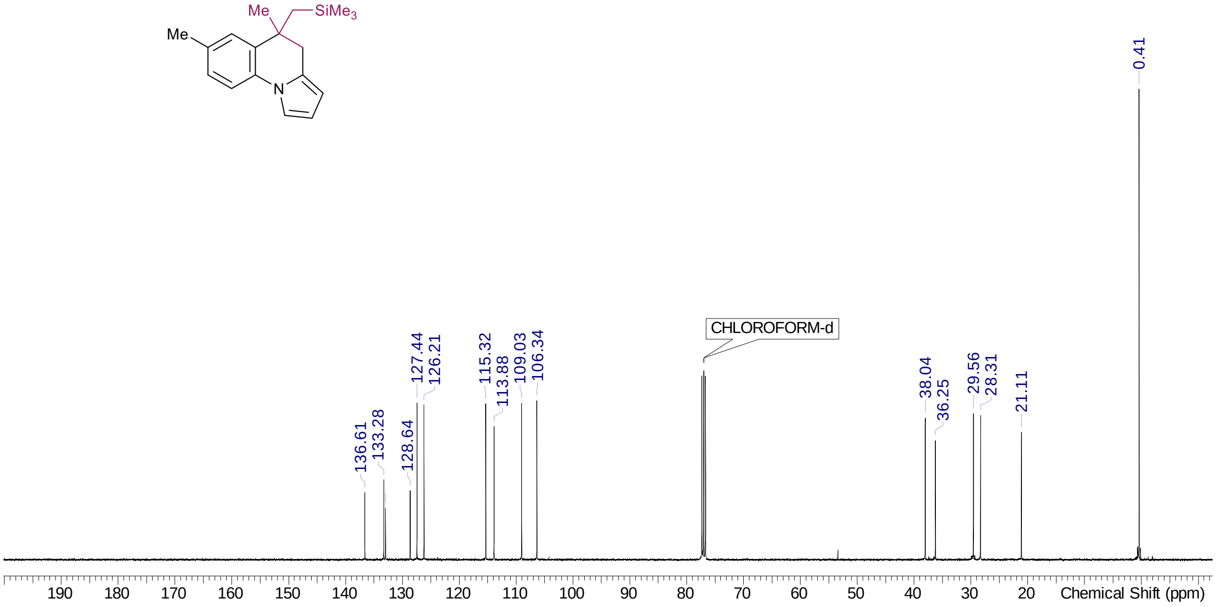

^{1}H, ^{13}C{^{1}H}-HSQC NMR ($CDCl_3$, 400, 101 MHz)

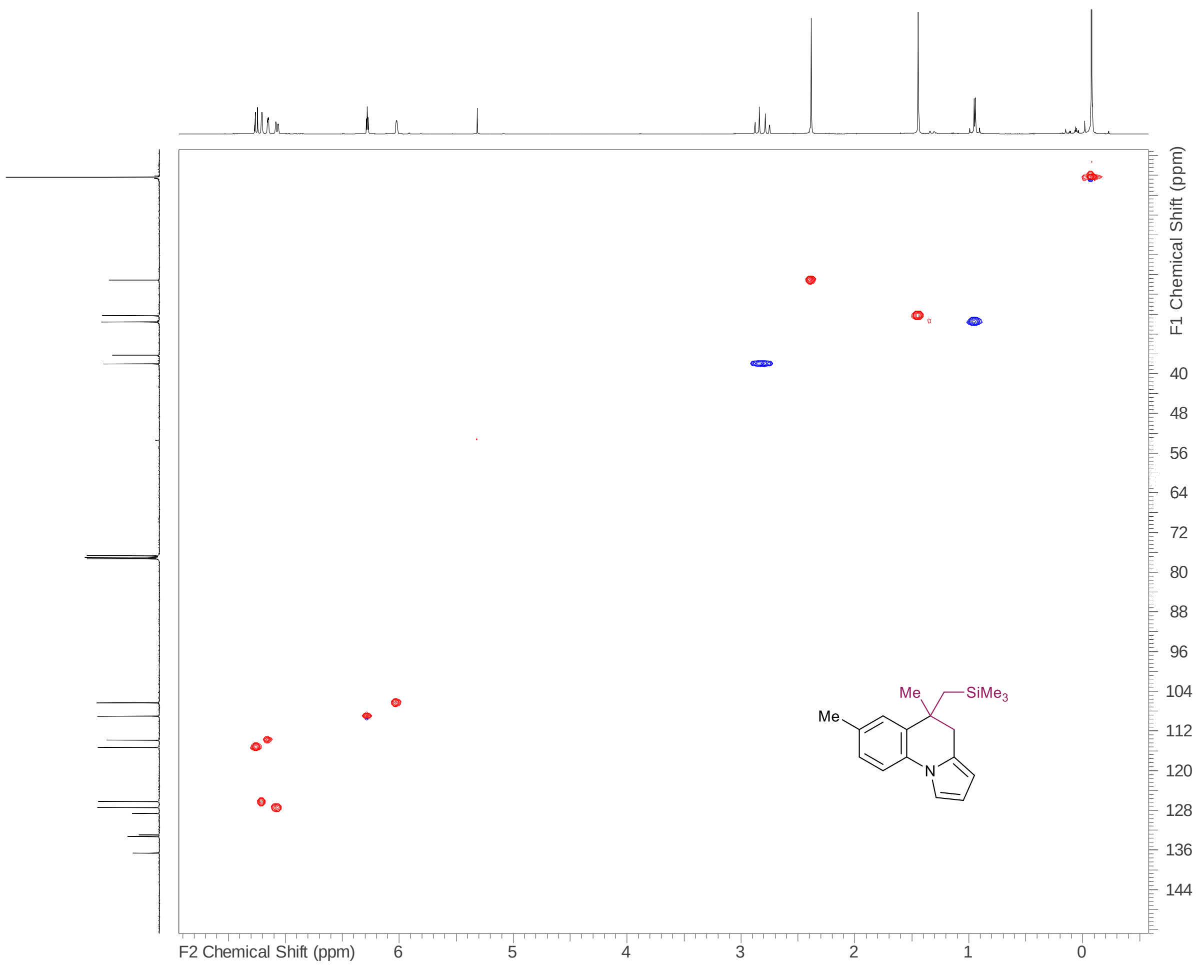

6-Methyl-4-phenyl-1-(*p*-tolyl)-4-((trimethylsilyl)methyl)-1,2,3,4-tetrahydroquinoline, 3ab

^{1}H NMR (400 MHz, $CDCl_3$)

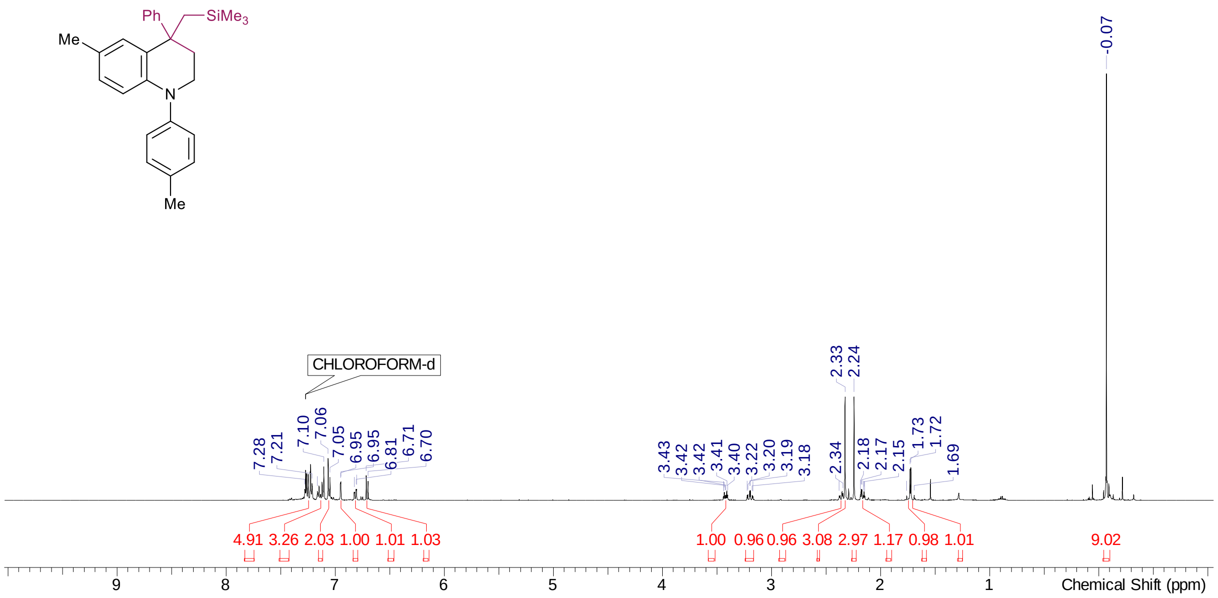

$^{13}C\{^{1}H\}$ NMR ($CDCl_3$, 101 MHz)

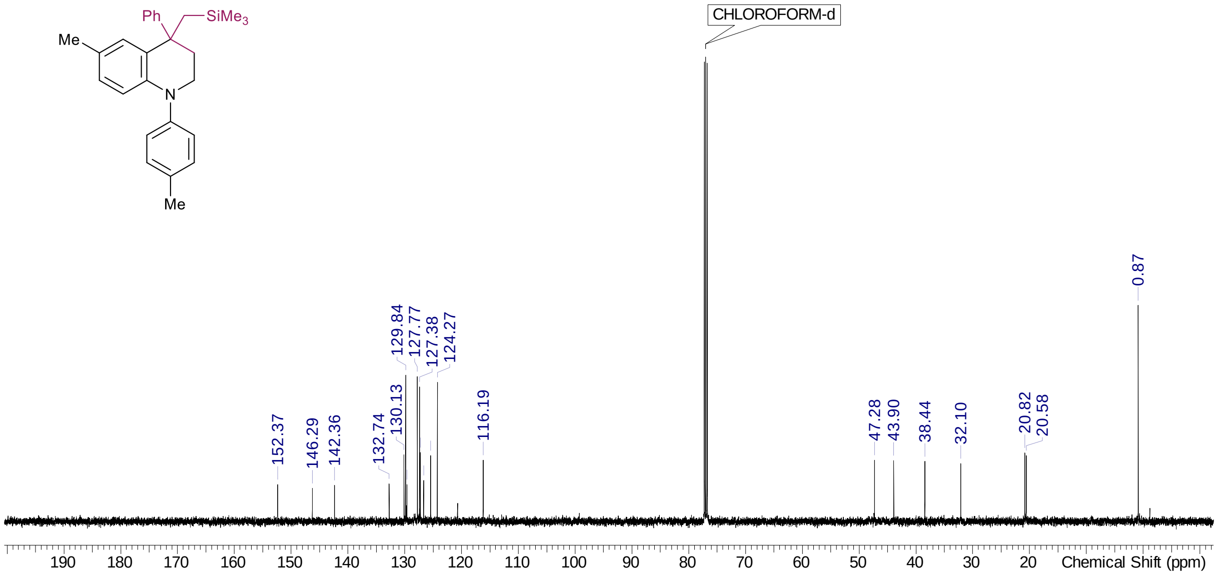

^1H, $^{13}C\{^1H\}$-HSQC NMR ($CDCl_3$, 400, 101 MHz)

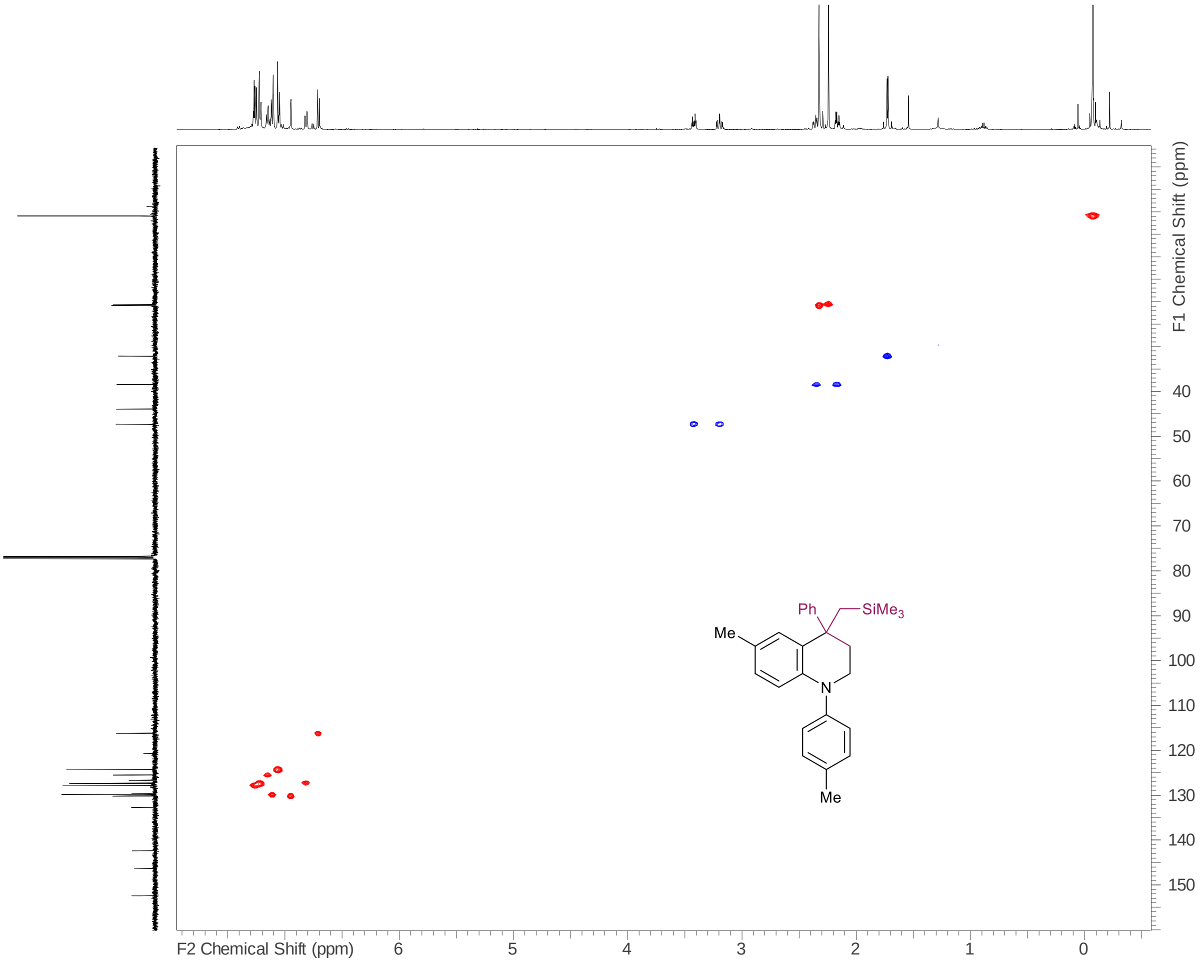

4-((Dimethyl(phenyl)silyl)methyl)-4,6-dimethyl-1-(*p*-tolyl)-1,2,3,4-tetrahydroquinoline, 3ac

Note: spectra contain 10% amine **1a**

^{1}H NMR (500 MHz, $CDCl_3$)

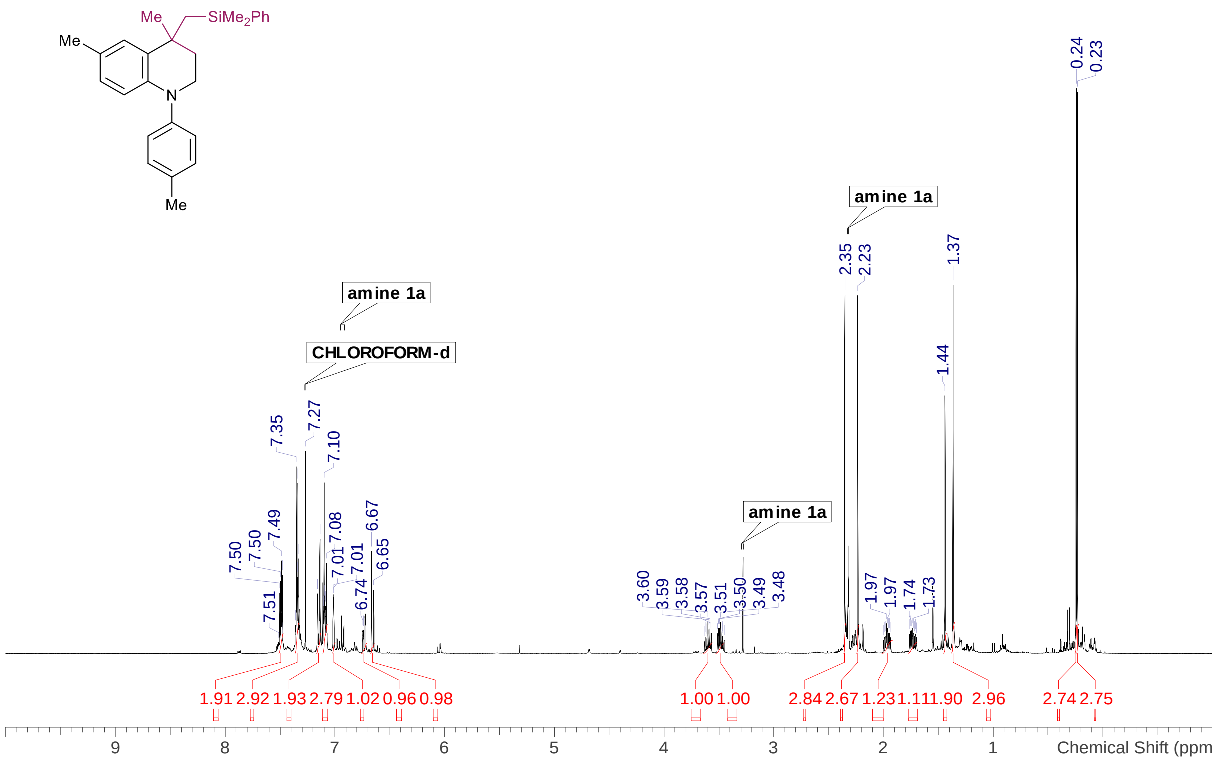

$^{13}C\{^1H\}$ NMR ($CDCl_3$, 126 MHz)

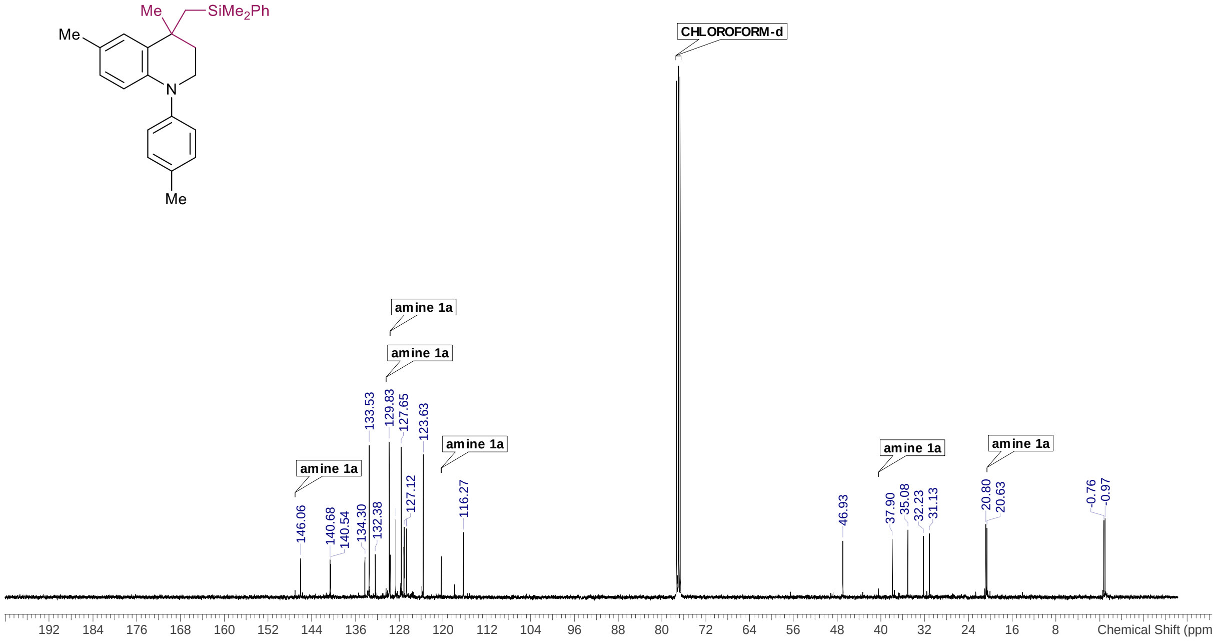

4-((Dimethyl(phenyl)silyl)methyl)-4,6-dimethyl-1-(*p*-tolyl)-1,4-dihydroquinoline, 3ac'

^{1}H NMR (500 MHz, $CDCl_3$)

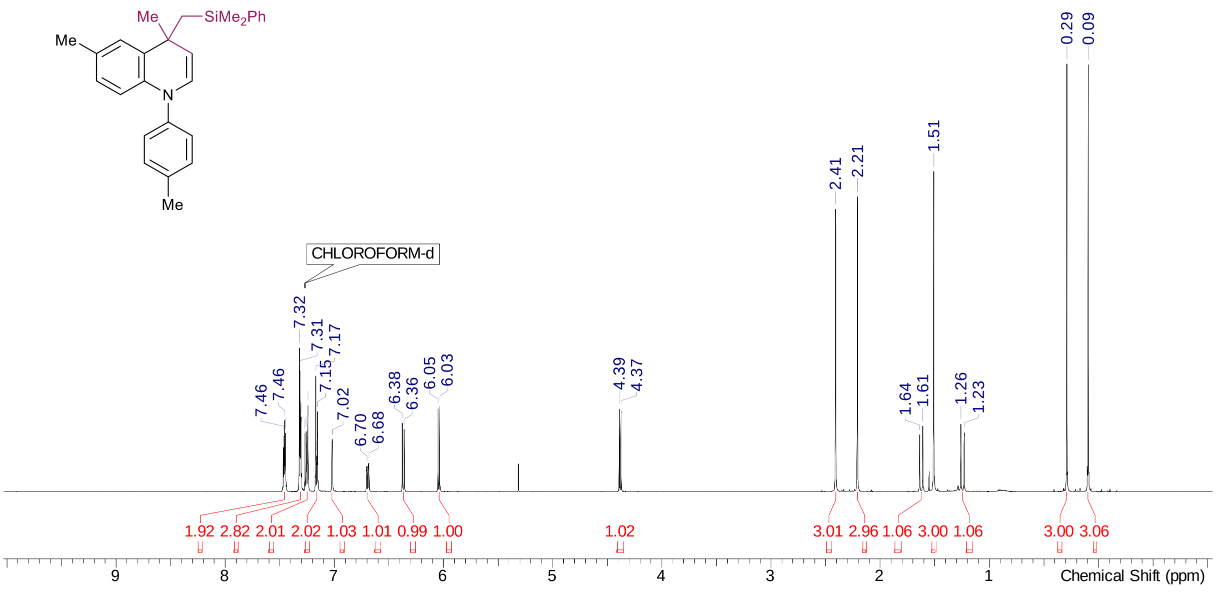

$^{13}C\{^{1}H\}$ NMR ($CDCl_3$, 126 MHz)

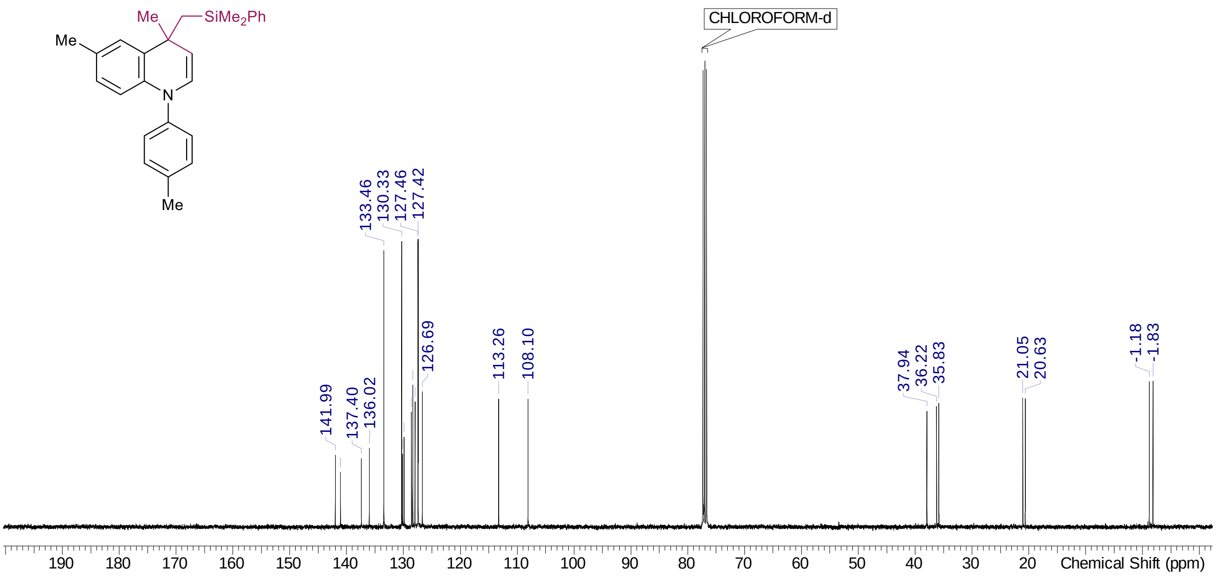

^{1}H, $^{13}C\{^{1}H\}$-HSQC NMR ($CDCl_3$, 500, 126 MHz)

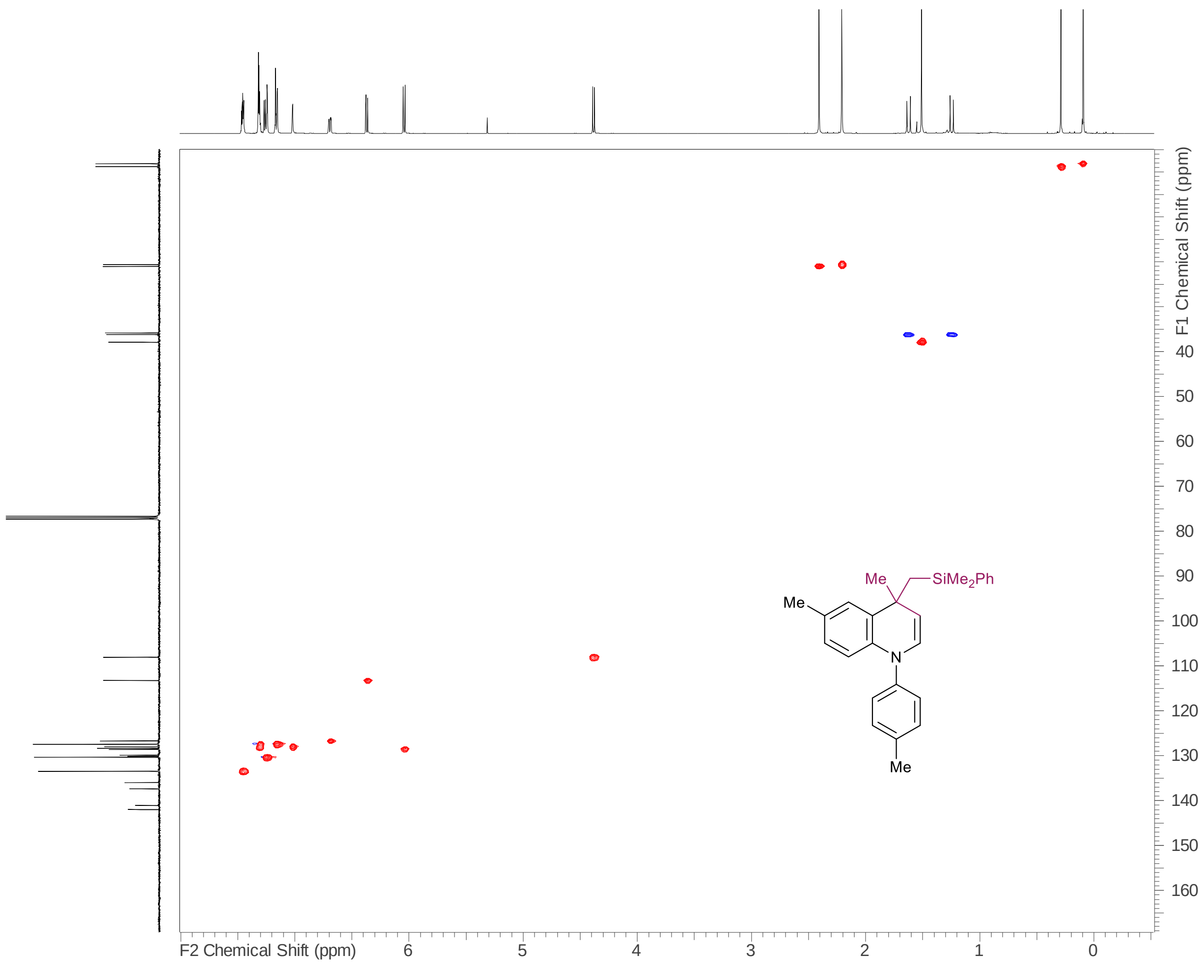

6-Methyl-4,4-diphenyl-1-(*p*-tolyl)-1,2,3,4-tetrahydroquinoline, 3ad

^{1}H NMR (500 MHz, $CDCl_3$)

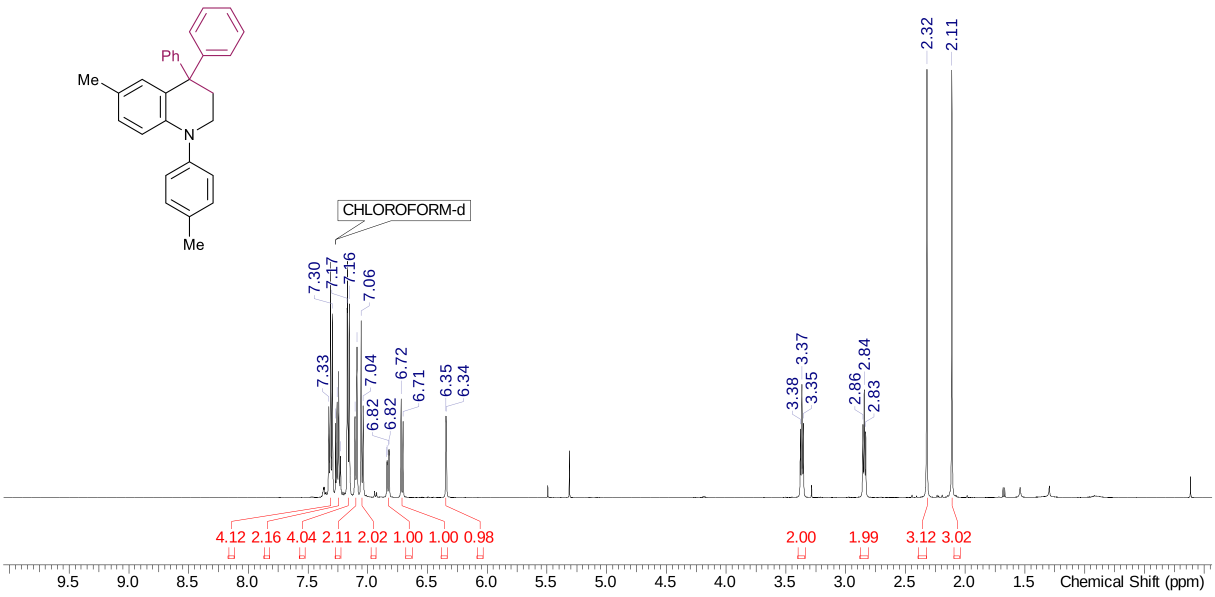

$^{13}C\{^{1}H\}$ NMR ($CDCl_3$, 126 MHz)

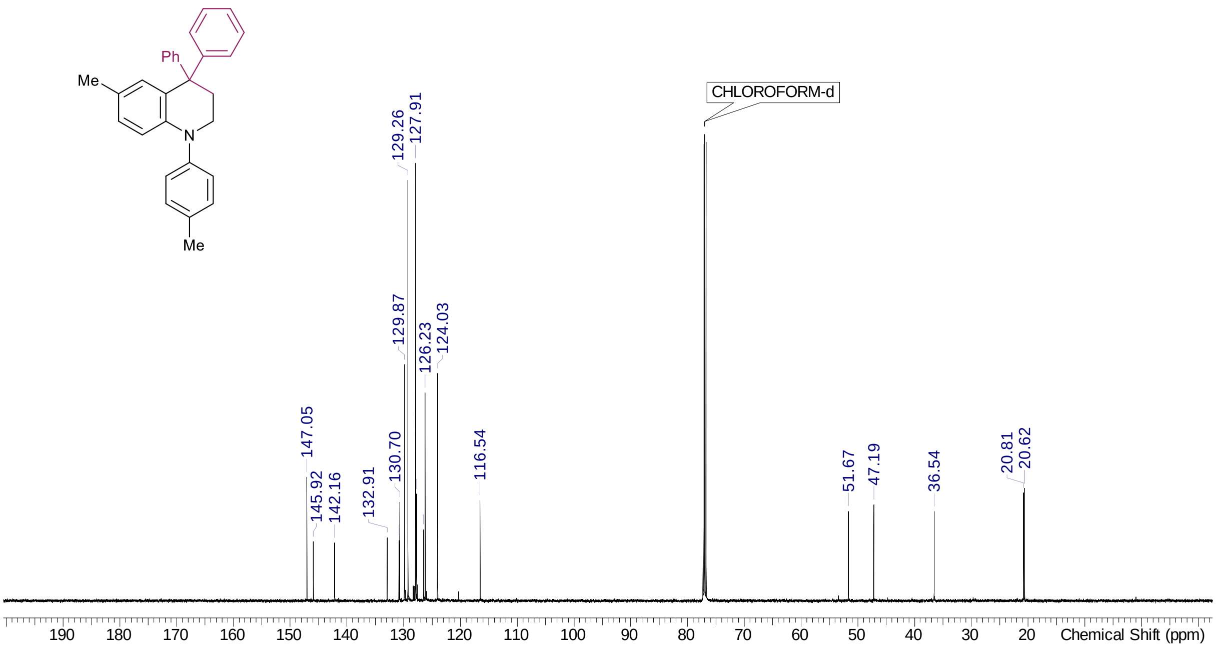

^{1}H, ^{13}C{^{1}H}-HSQC NMR ($CDCl_3$, 500, 126 MHz)

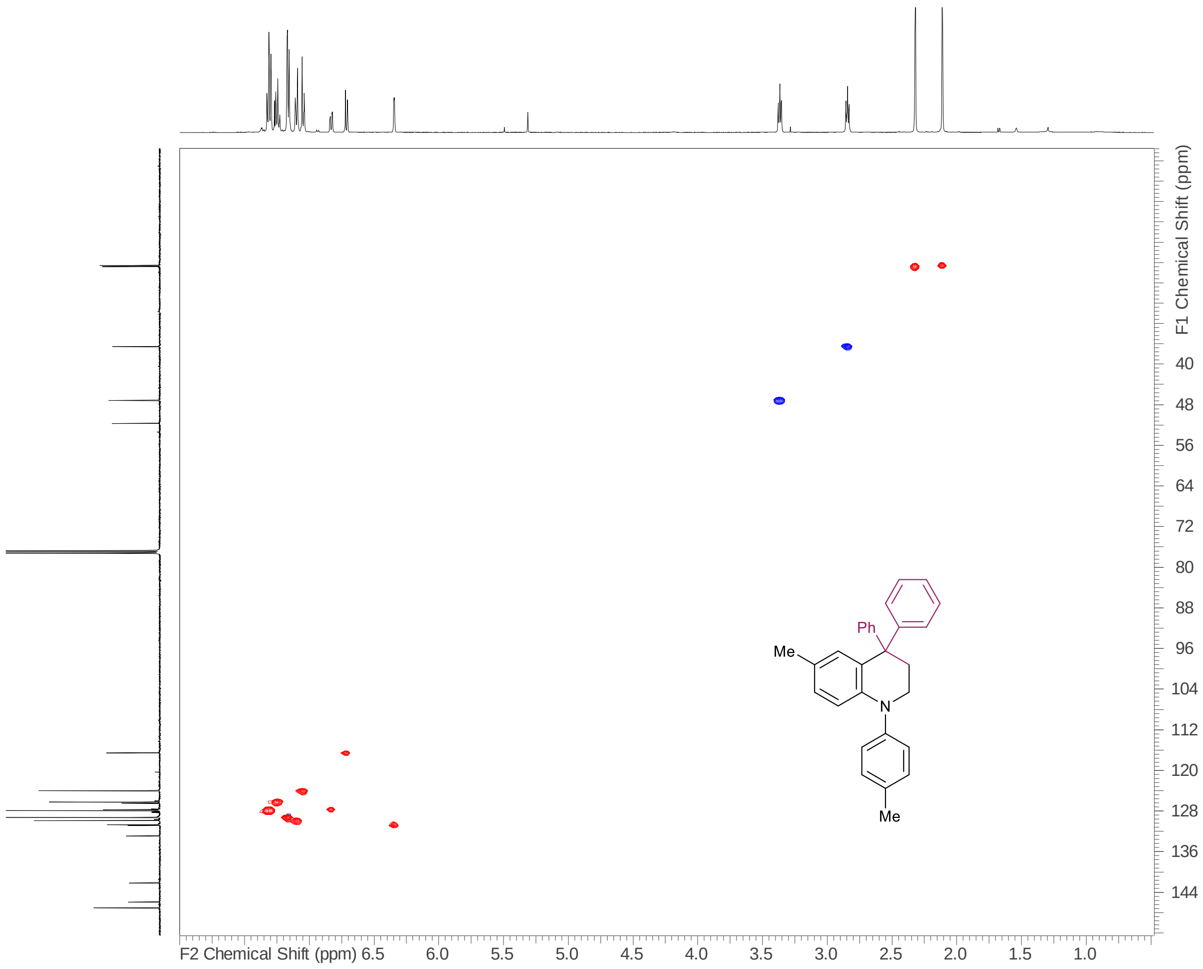

6-Methyl-4,4-diphenyl-1-(*p*-tolyl)-1,4-dihydroquinoline, 3ad'

^{1}H NMR (400 MHz, $CDCl_3$)

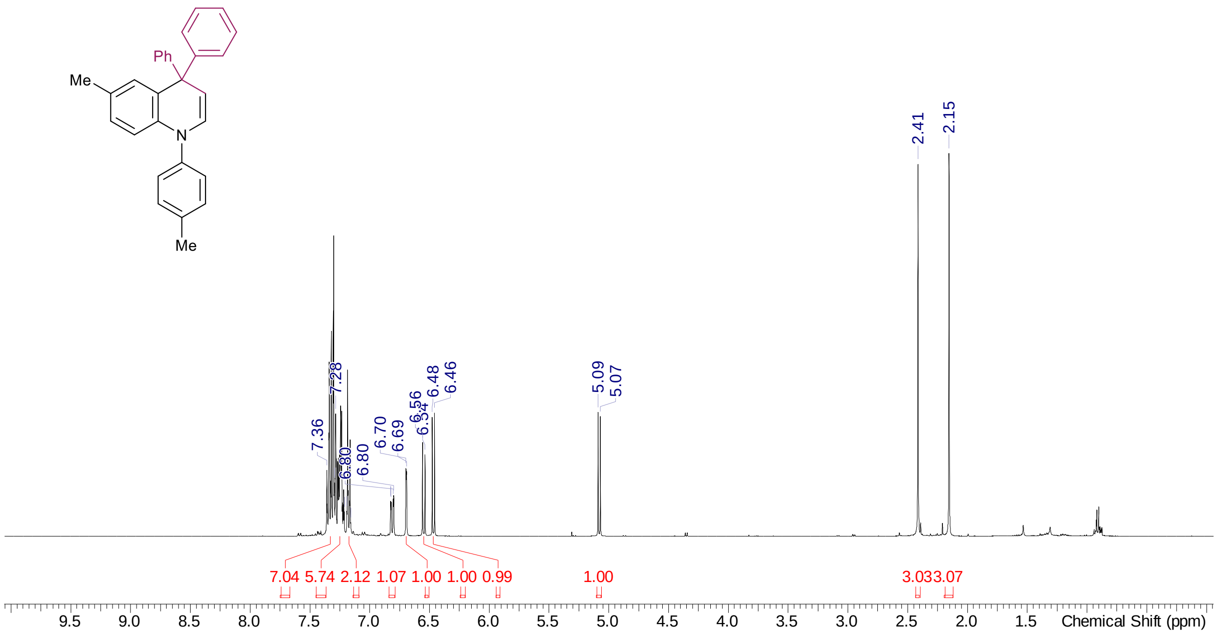

$^{13}C\{^{1}H\}$ NMR ($CDCl_3$, 101 MHz)

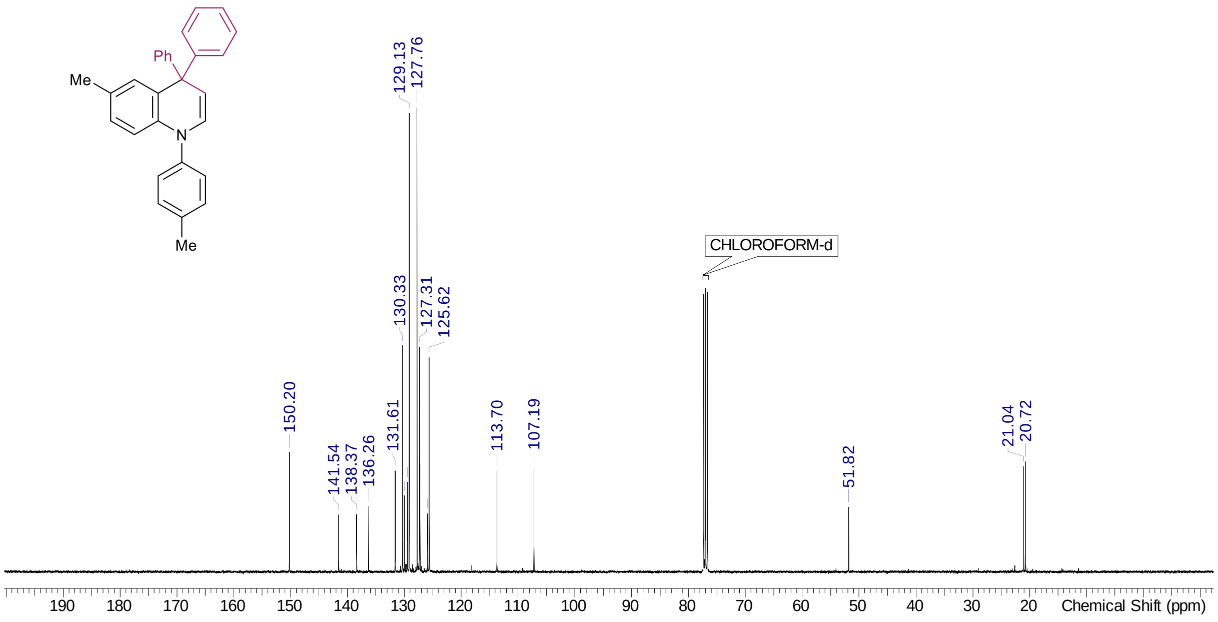

^{1}H, $^{13}C\{^{1}H\}$-HSQC NMR ($CDCl_3$, 400, 101 MHz)

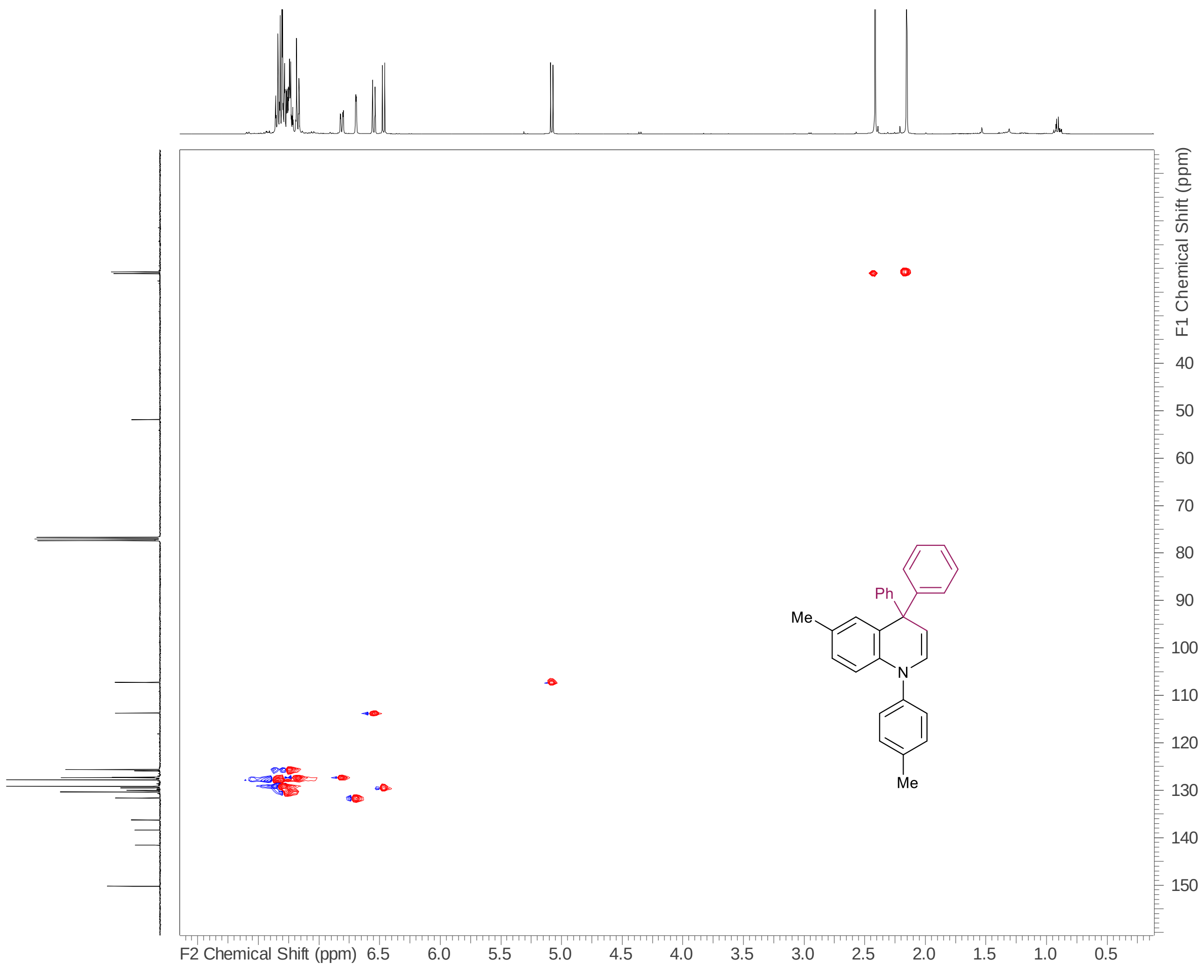

4-(4-Fluorophenyl)-6-methyl-4-phenyl-1-(*p*-tolyl)-1,2,3,4-tetrahydroquinoline, 3ae

^{1}H NMR (400 MHz, $CDCl_3$)

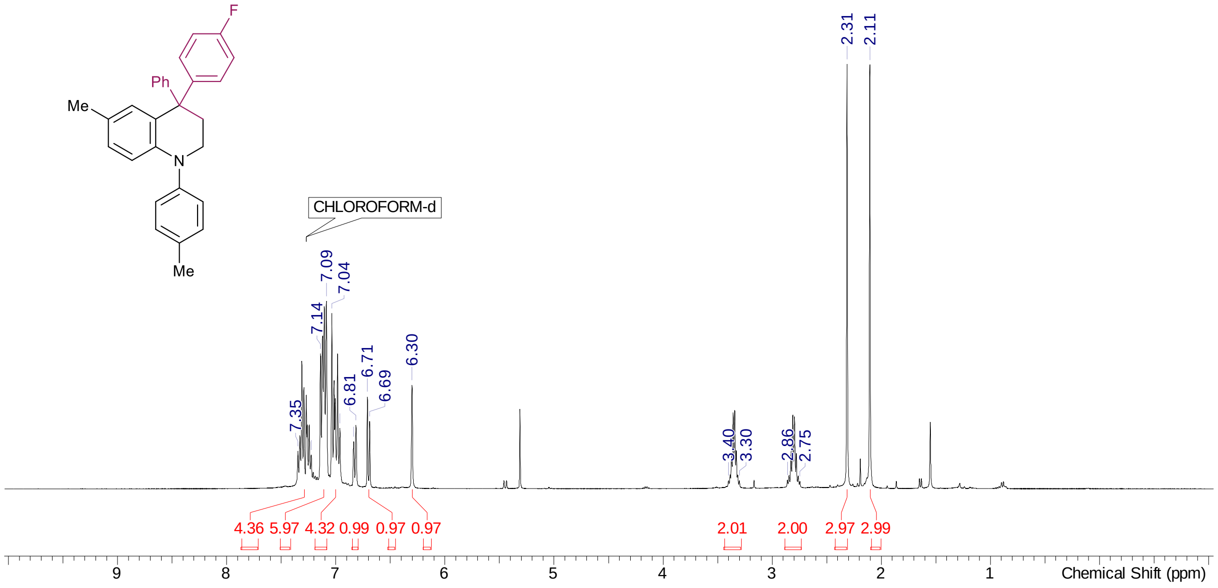

^{13}C{^{1}H} NMR ($CDCl_3$, 101 MHz)

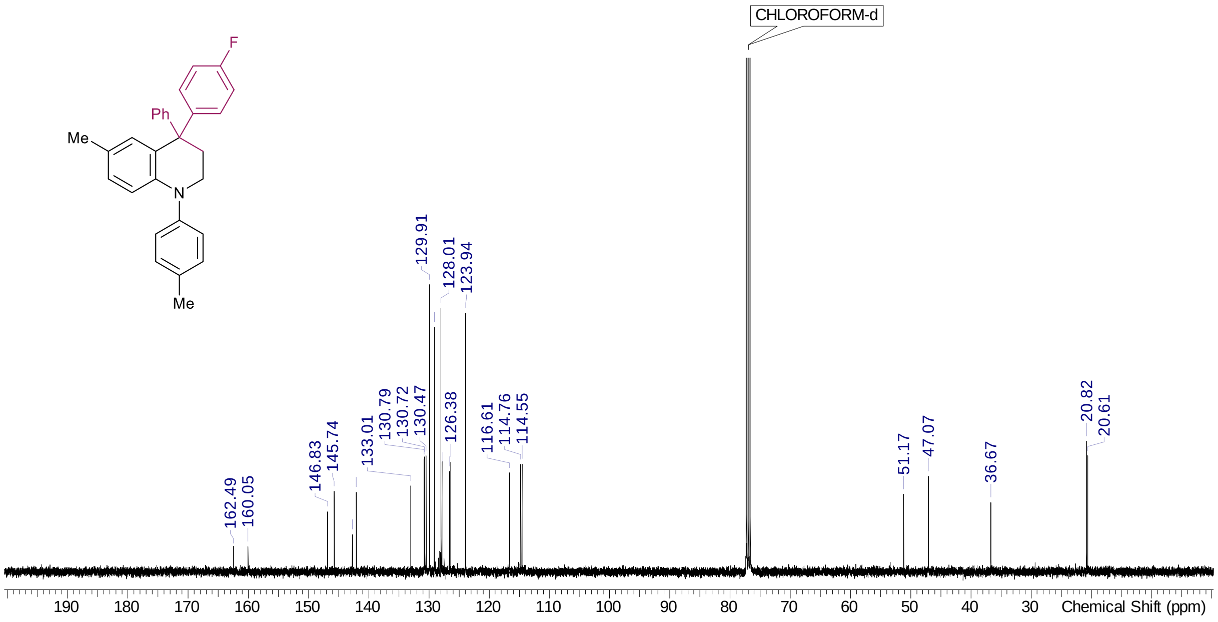

^{1}H, $^{13}C\{^{1}H\}$-HSQC NMR ($CDCl_3$, 400, 101 MHz)

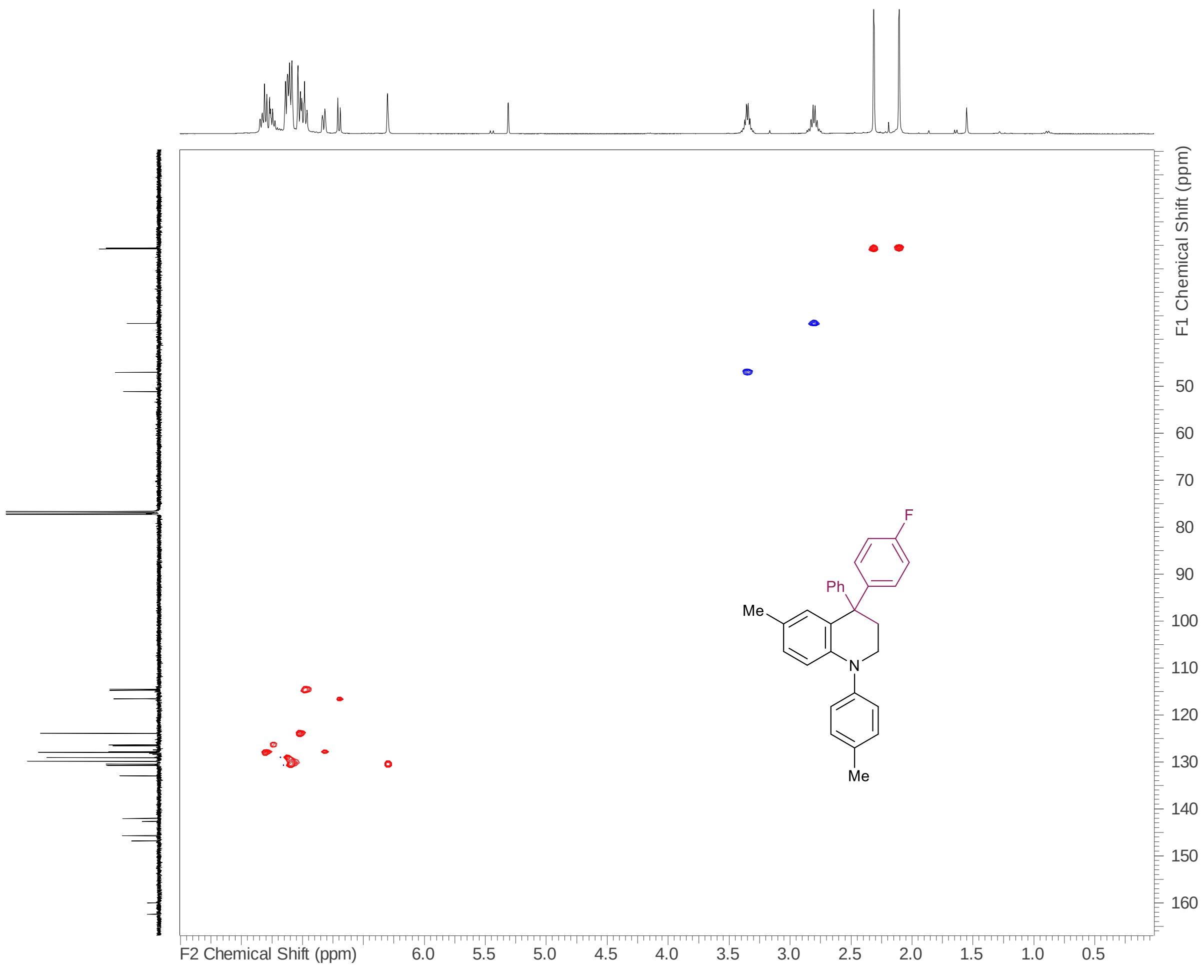

4-(4-Fluorophenyl)-6-methyl-4-phenyl-1-(*p*-tolyl)-1,4-dihydroquinoline, 3ae'

^{1}H NMR (400 MHz, $CDCl_3$)

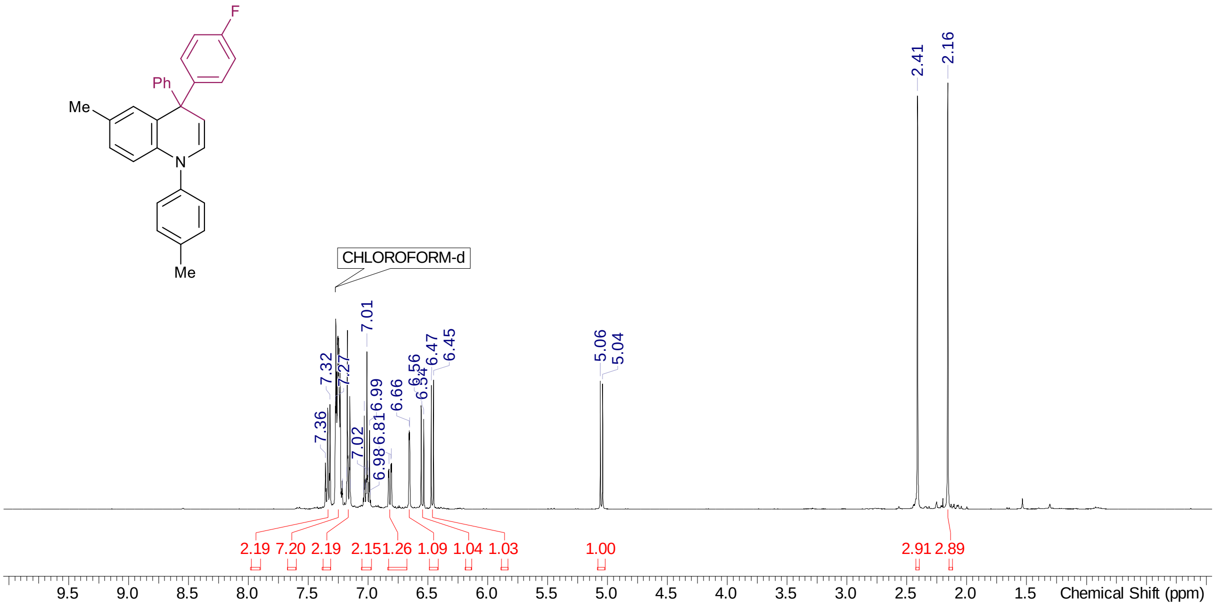

^{13}C{^{1}H} NMR ($CDCl_3$, 101 MHz)

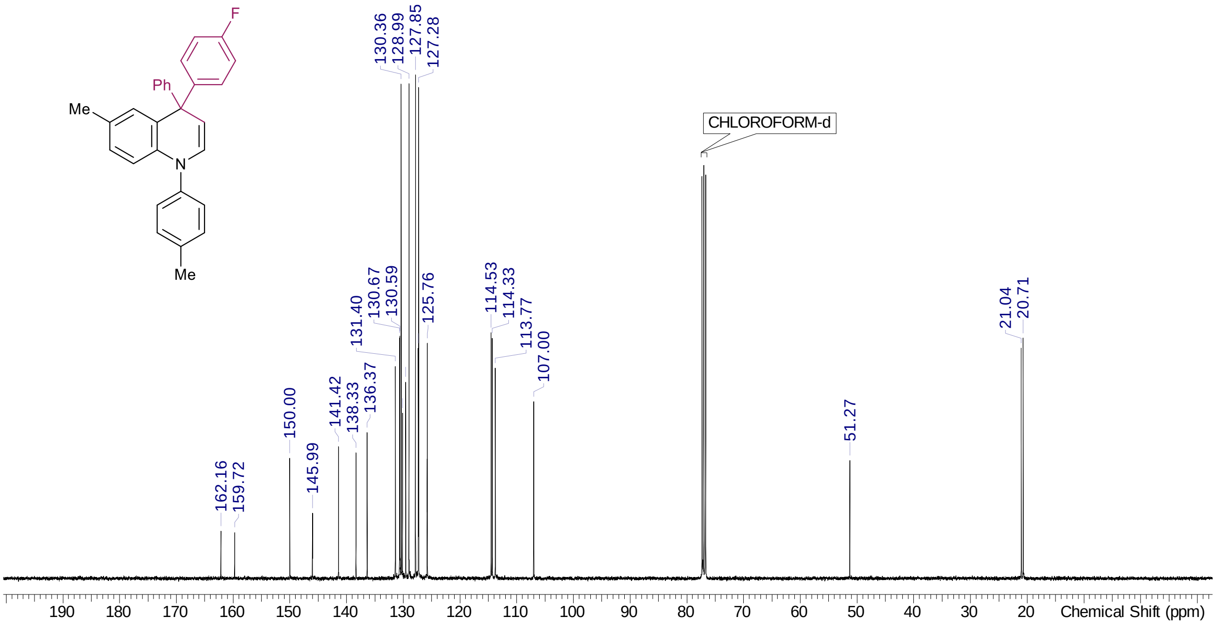

^{1}H, $^{13}C\{^{1}H\}$-HSQC NMR ($CDCl_3$, 400, 101 MHz)

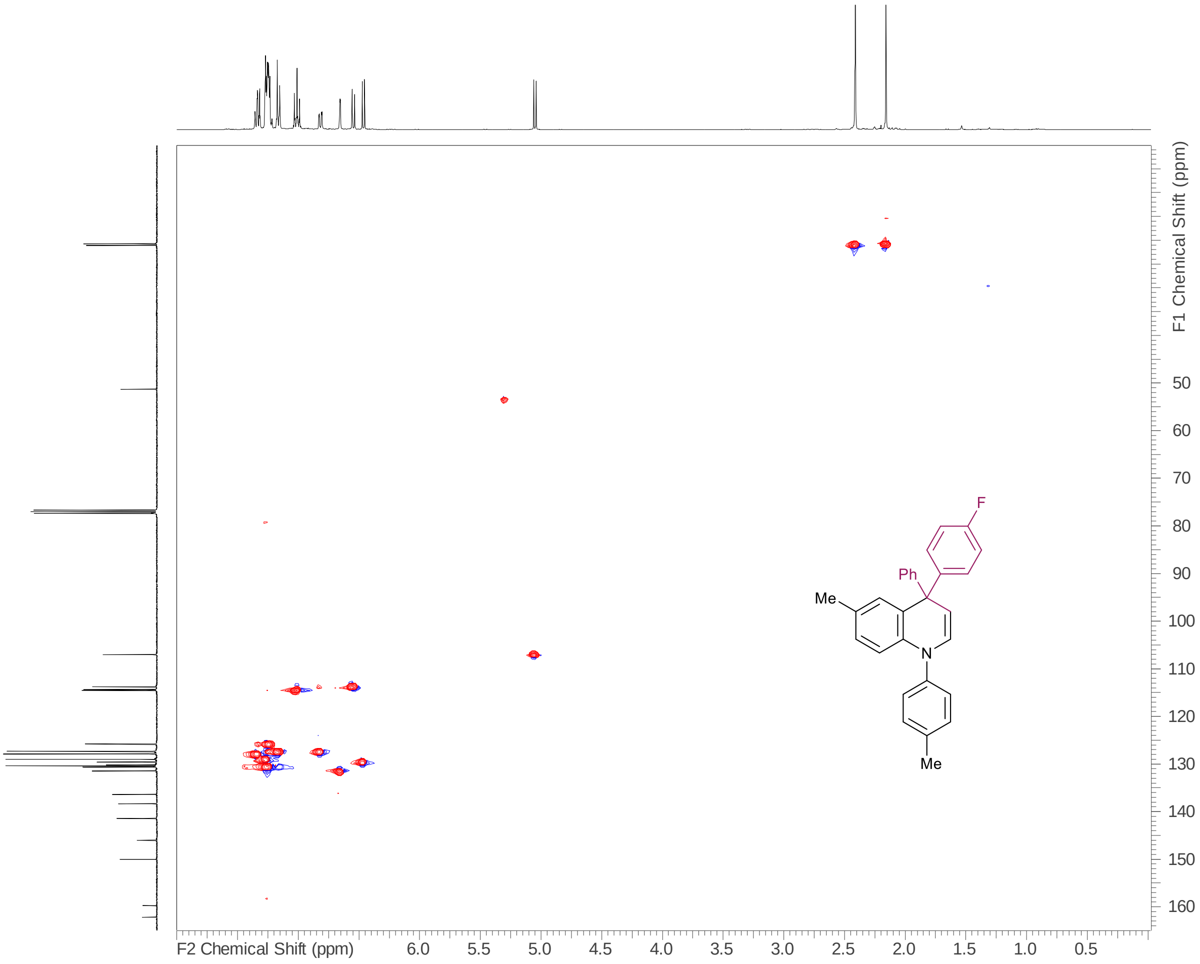

4-(4-Bromophenyl)-6-methyl-4-phenyl-1-(*p*-tolyl)-1,2,3,4-tetrahydroquinoline, 3af

^{1}H NMR (400 MHz, CDCl$_3$)

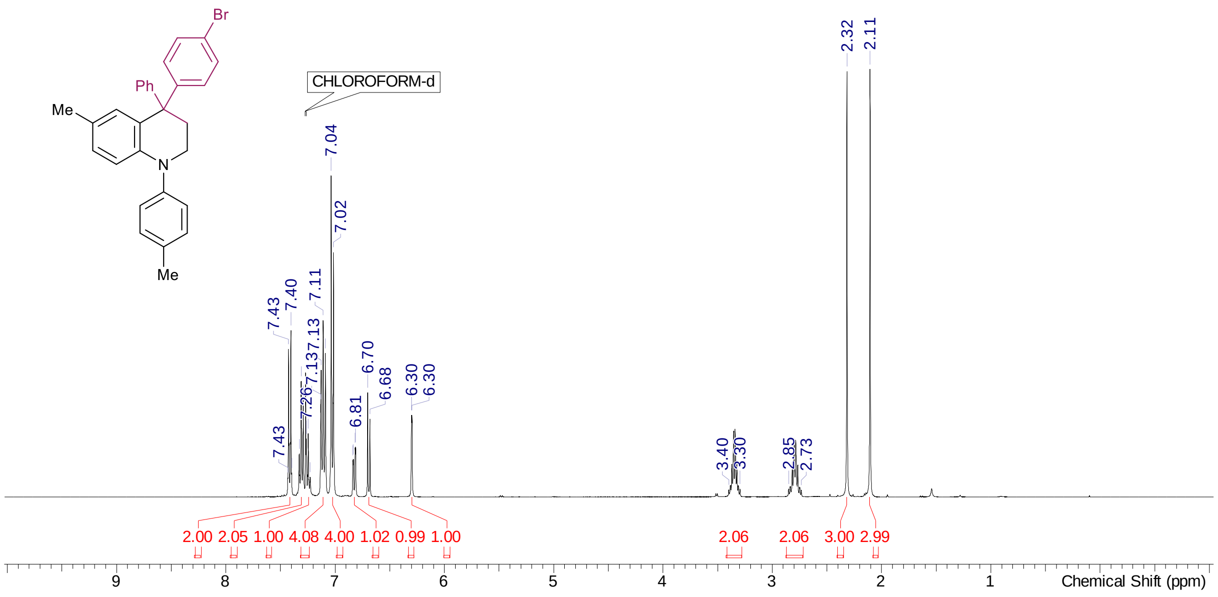

^{13}C{^{1}H} NMR (CDCl$_3$, 101 MHz)

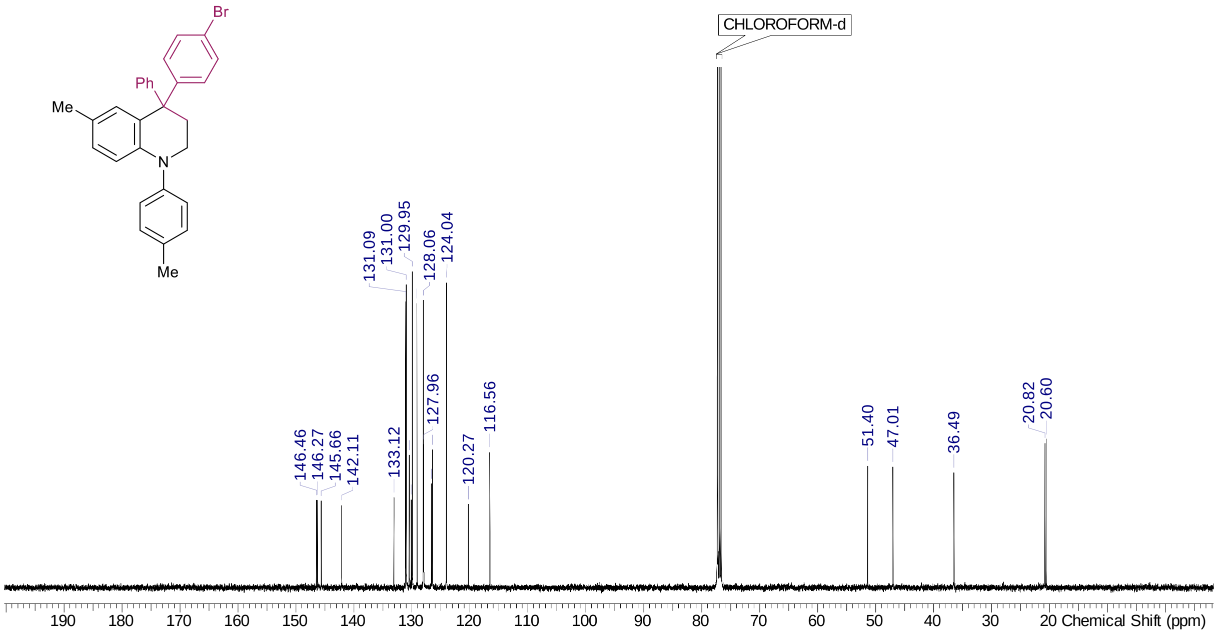

^{1}H, $^{13}C\{^{1}H\}$-HSQC NMR ($CDCl_3$, 400, 101 MHz)

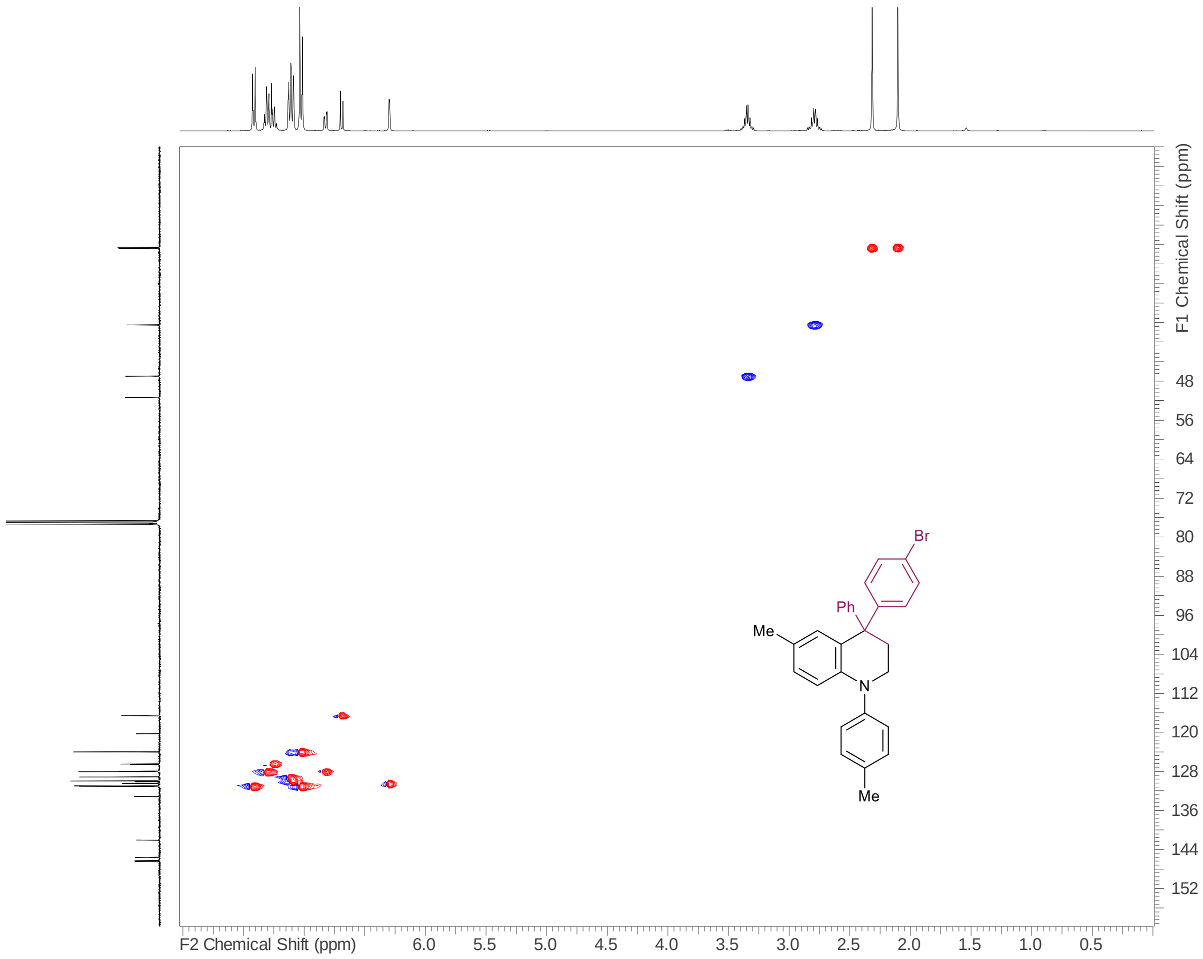

4-(4-Bromophenyl)-6-methyl-4-phenyl-1-(*p*-tolyl)-1,4-dihydroquinoline, 3af'

^{1}H NMR (400 MHz, $CDCl_3$)

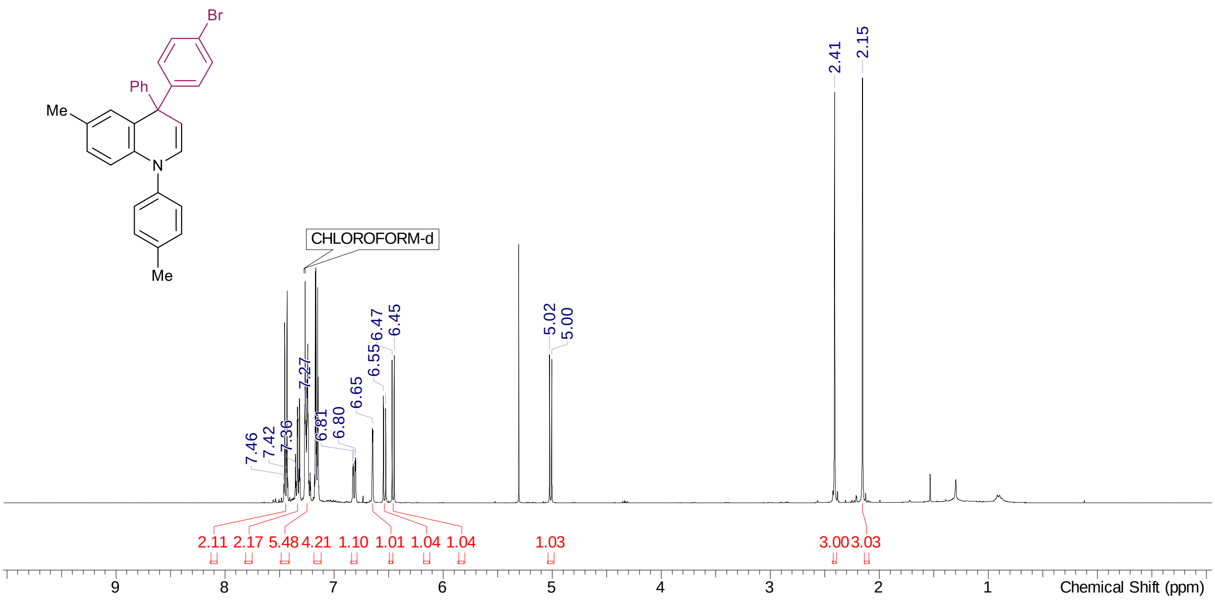

^{13}C{^{1}H} NMR ($CDCl_3$, 101 MHz)

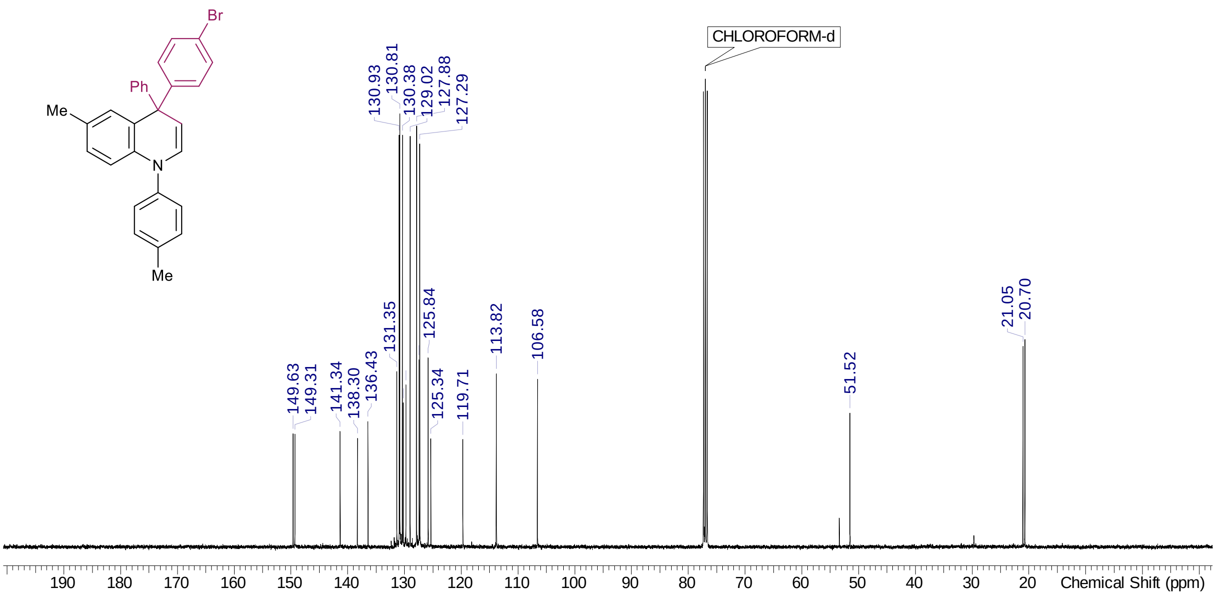

^{1}H, $^{13}C\{^{1}H\}$-HSQC NMR ($CDCl_3$, 400, 101 MHz)

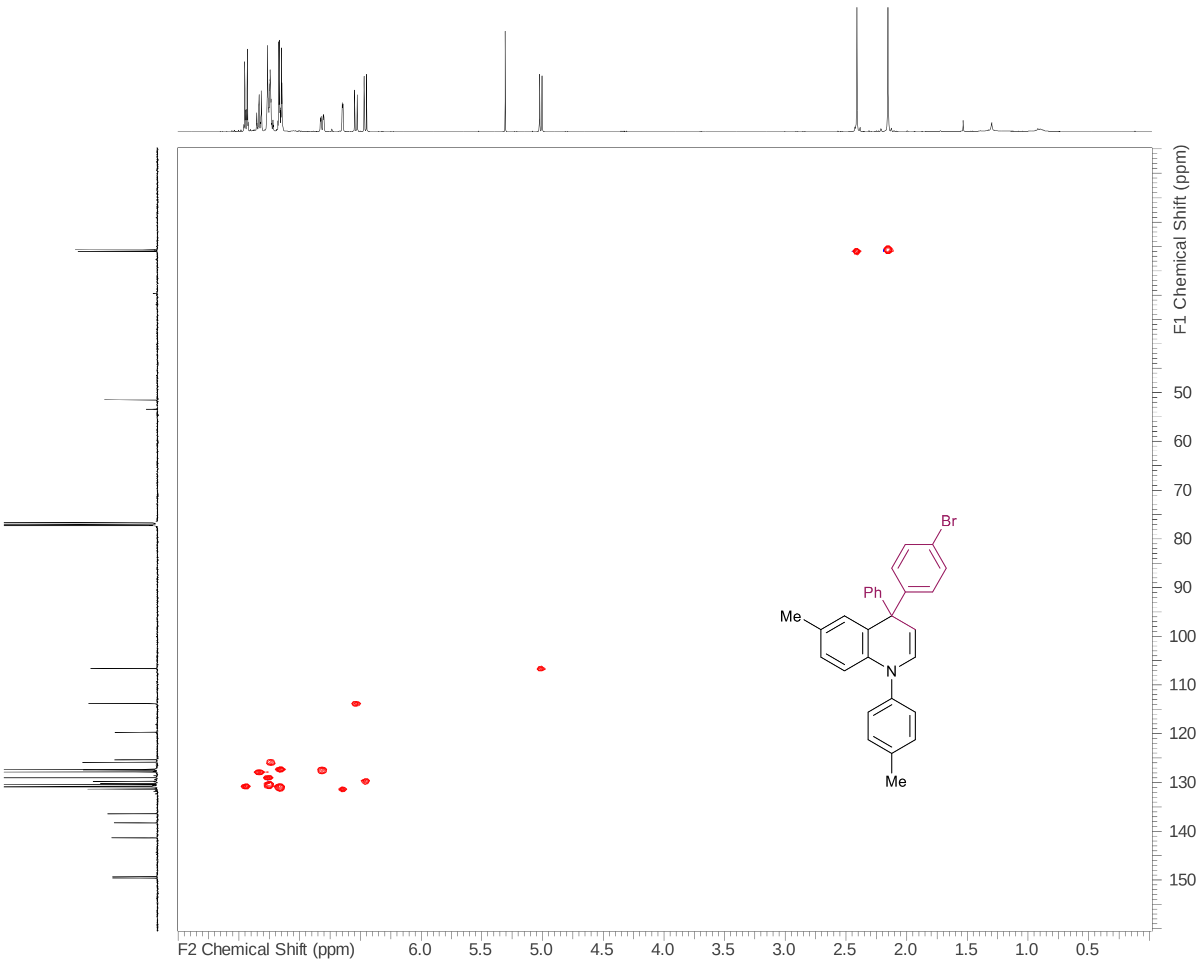

4-(4-Methoxyphenyl)-6-methyl-4-phenyl-1-(*p*-tolyl)-1,2,3,4-tetrahydroquinoline, 3ag

^{1}H NMR (500 MHz, $CDCl_3$)

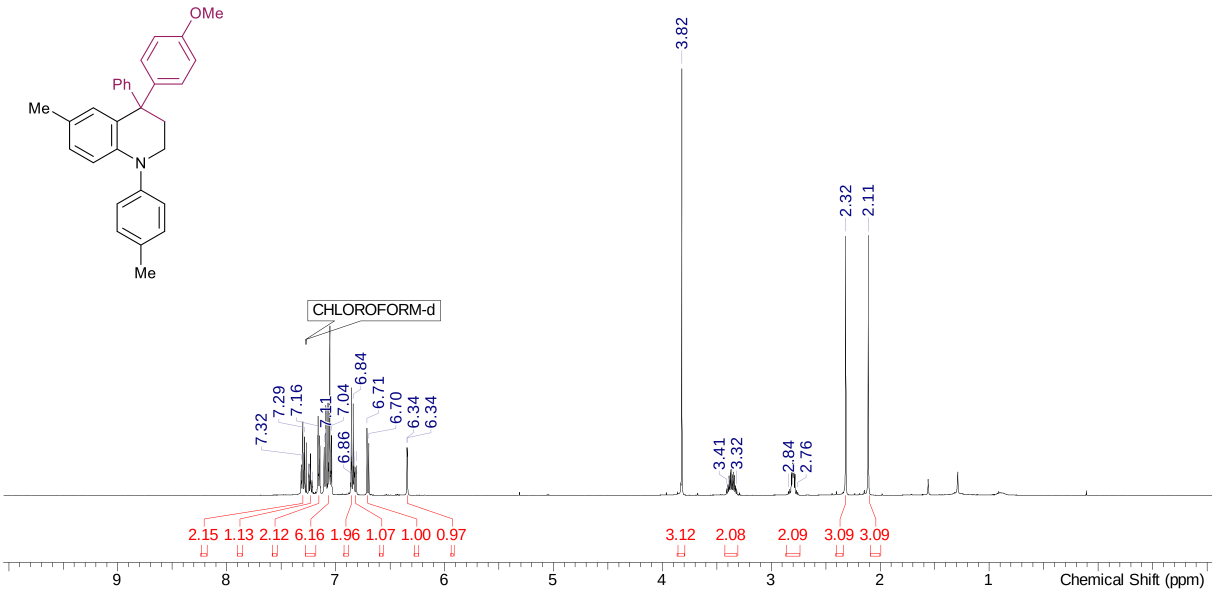

$^{13}C\{^{1}H\}$ NMR ($CDCl_3$, 126 MHz)

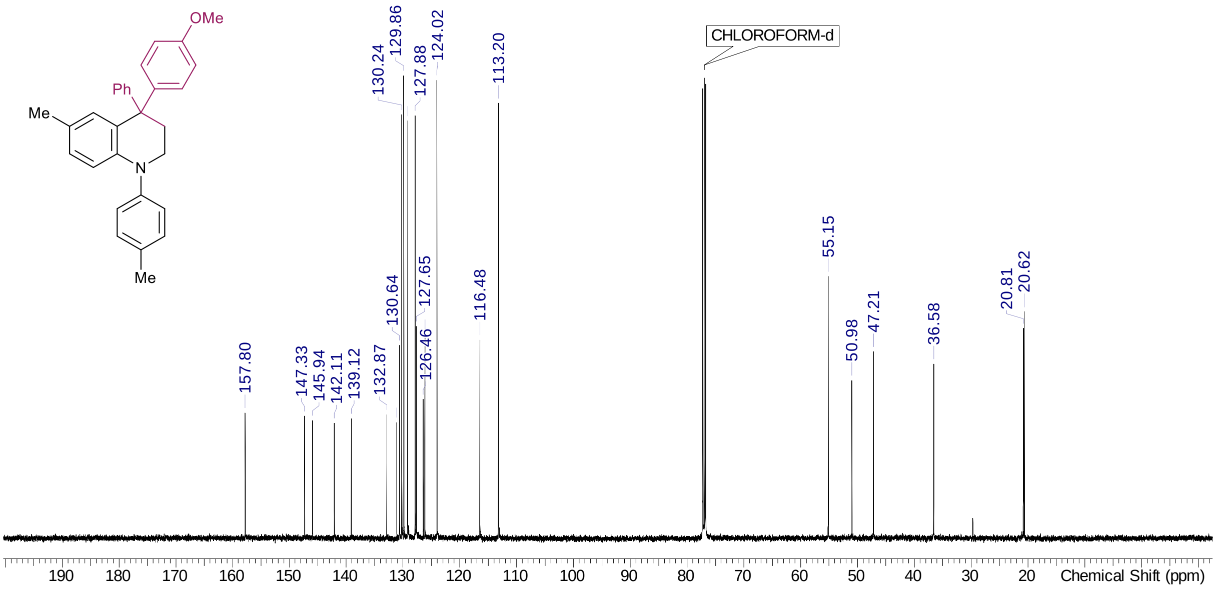

^{1}H, $^{13}C\{^{1}H\}$-HSQC NMR ($CDCl_3$, 500, 126 MHz)

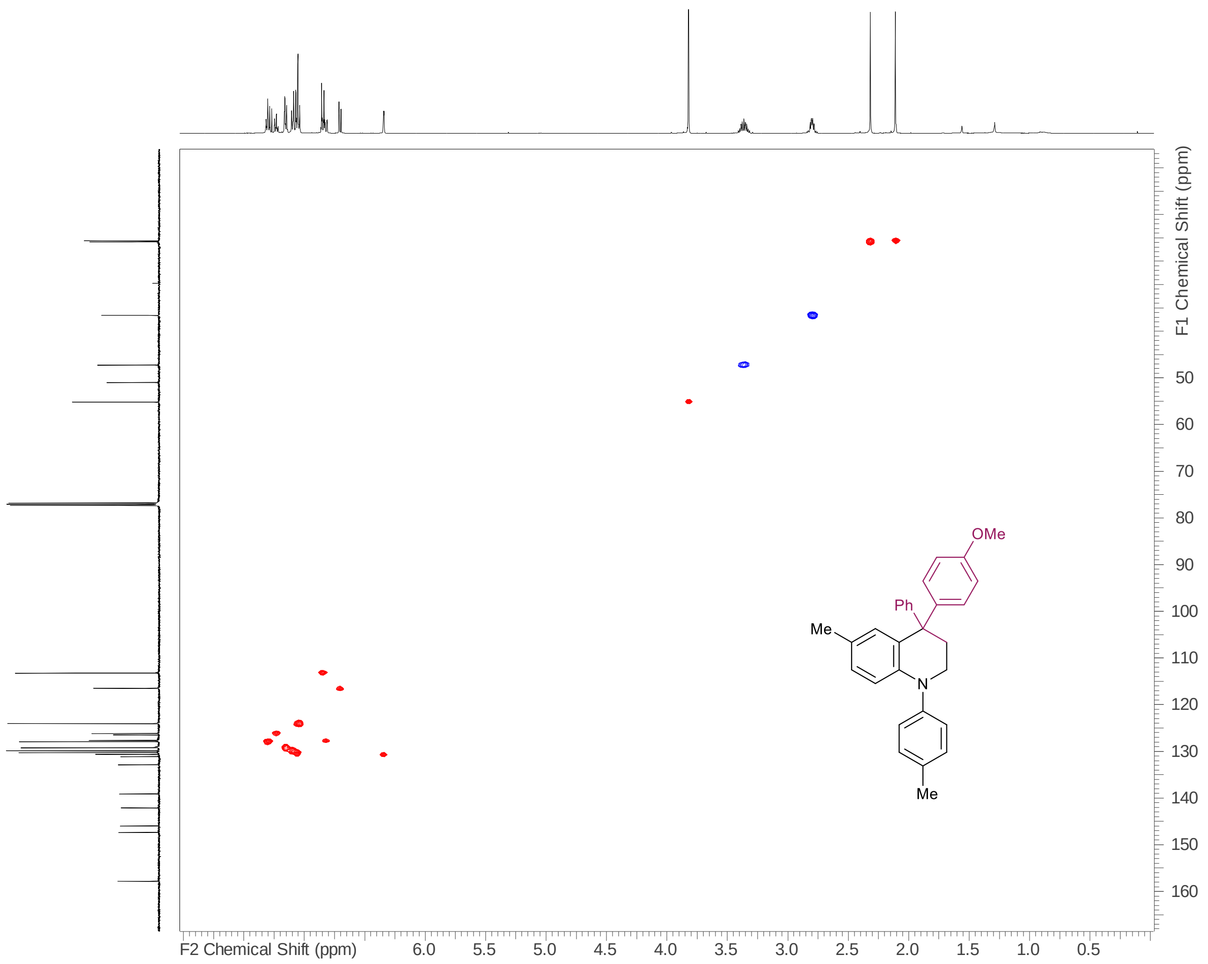

4-(4-Methoxyphenyl)-6-methyl-4-phenyl-1-(*p*-tolyl)-1,4-dihydroquinoline, 3ag'

^{1}H NMR (400 MHz, $CDCl_3$)

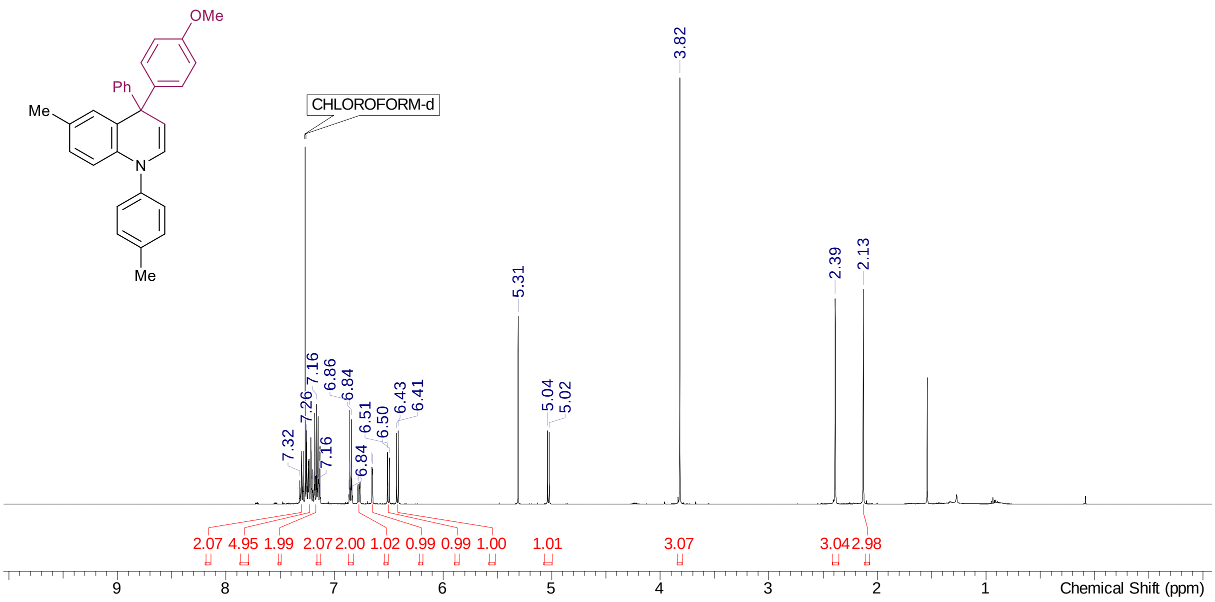

$^{13}C\{^{1}H\}$ NMR ($CDCl_3$, 101 MHz)

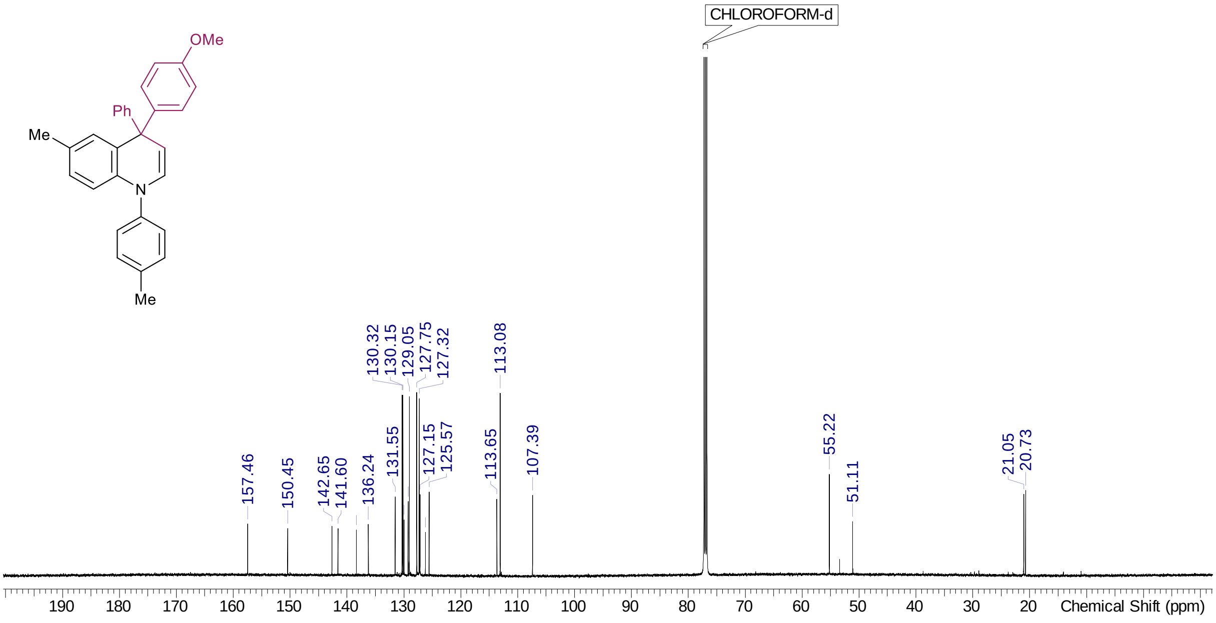

^{1}H, $^{13}C\{^{1}H\}$-HSQC NMR ($CDCl_3$, 400, 101 MHz)

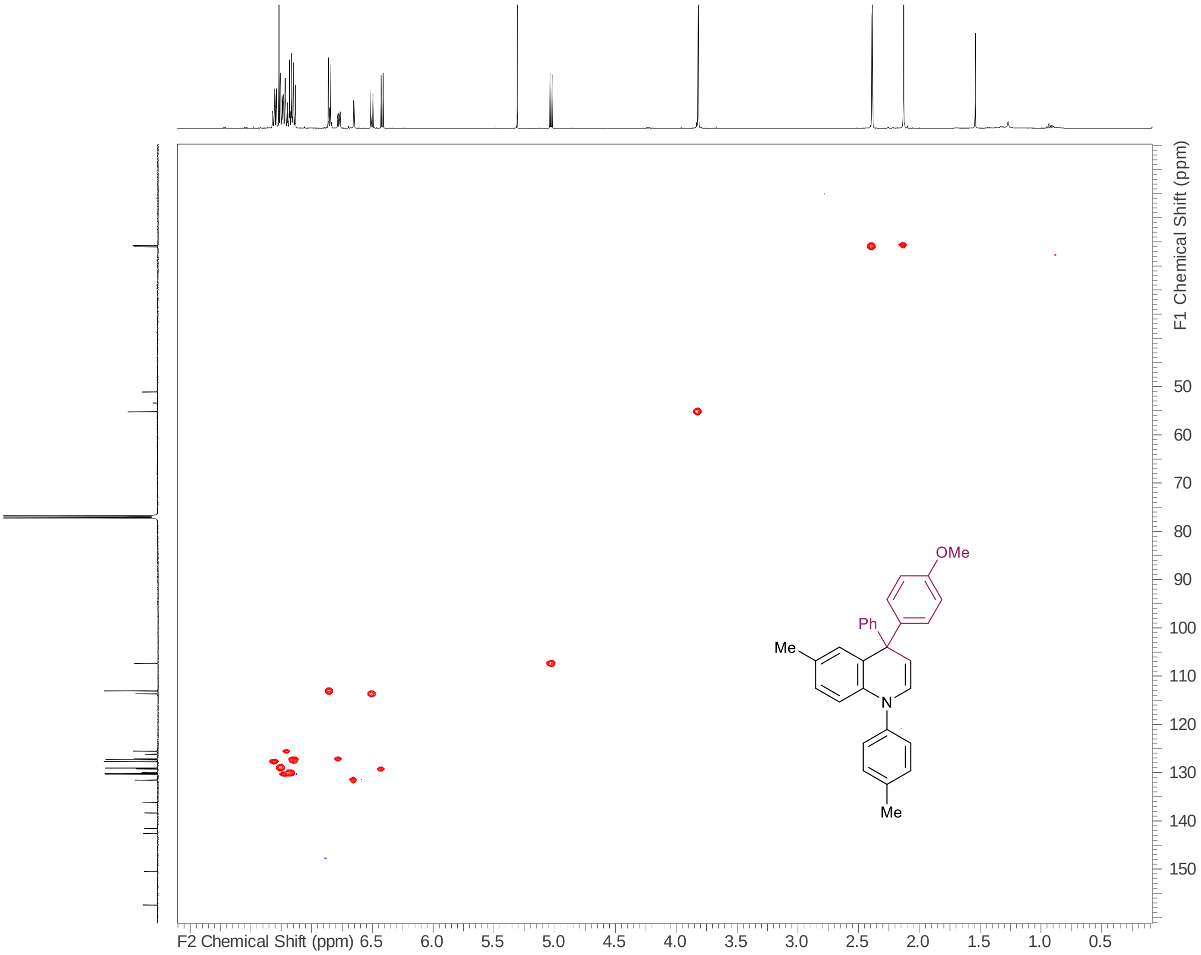

6-Methyl-4-phenyl-4-(4-(4,4,5,5-tetramethyl-1,3,2-dioxaborolan-2-yl)phenyl)-1-(p-tolyl)-1,2,3,4-tetrahydroquinoline, 3ah

^{1}H NMR (400 MHz, $CDCl_3$)

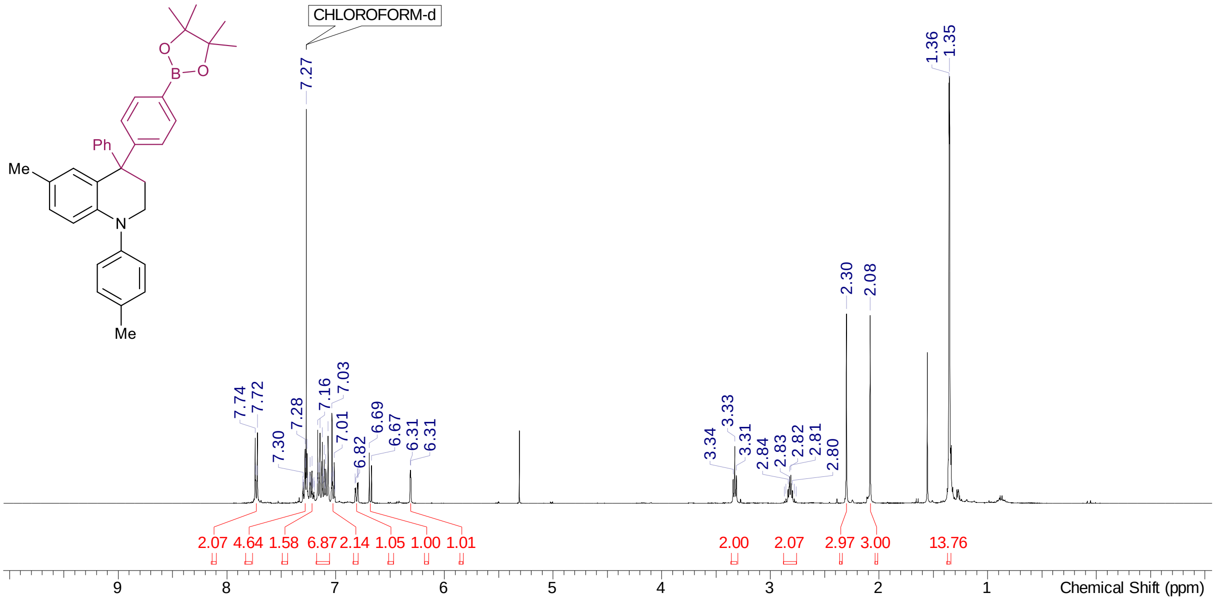

$^{13}C\{^1H\}$ NMR ($CDCl_3$, 101 MHz)

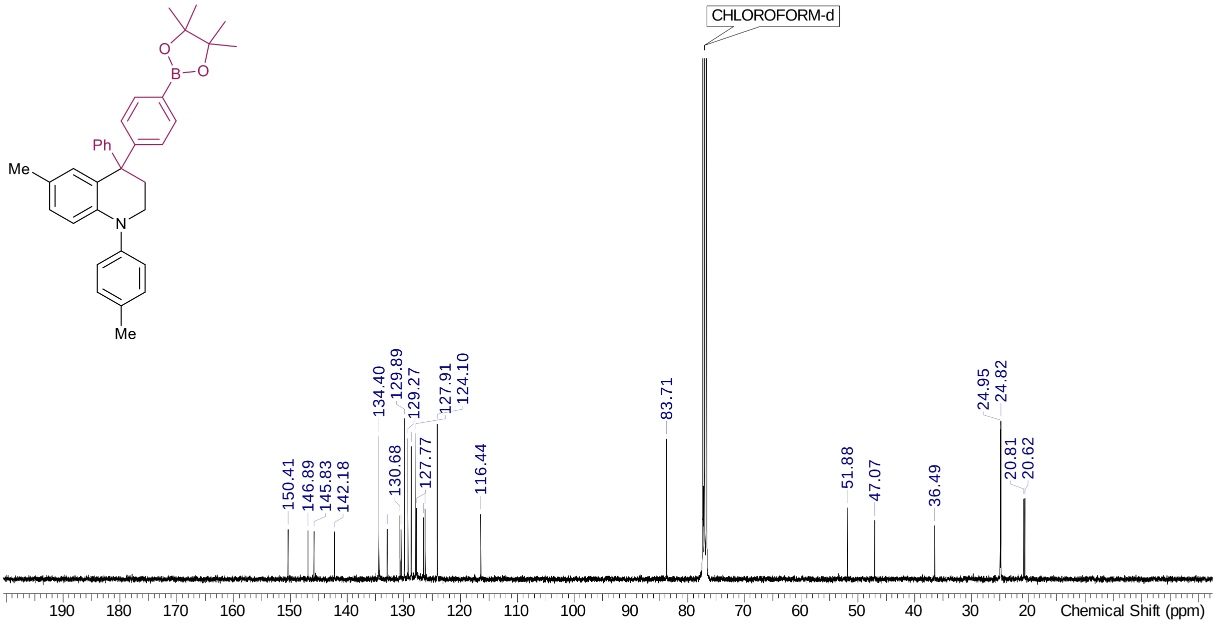

^{1}H, ^{13}C{^{1}H}-HSQC NMR ($CDCl_3$, 400, 101 MHz)

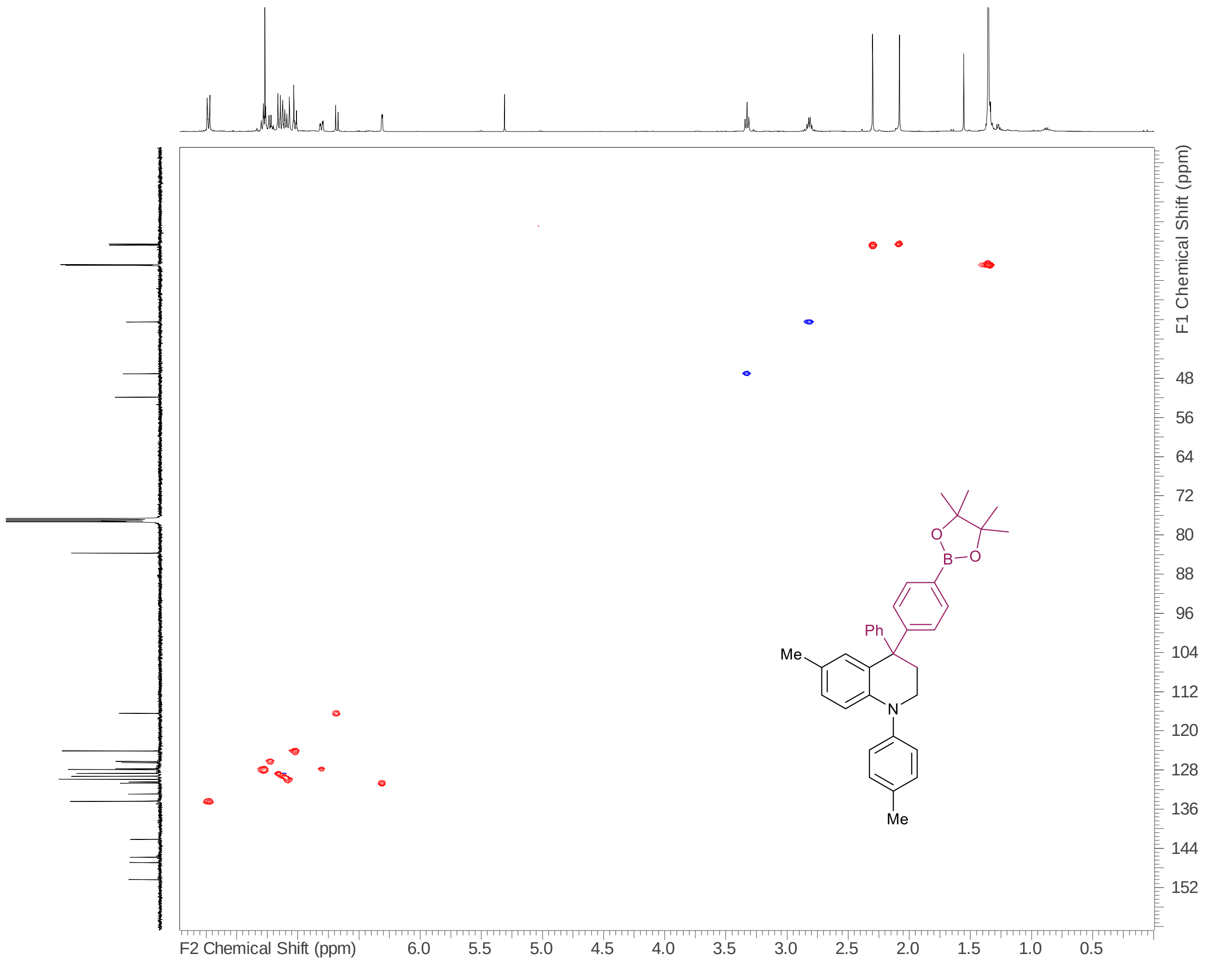

4-(3-Bromophenyl)-6-methyl-4-phenyl-1-(*p*-tolyl)-1,2,3,4-tetrahydroquinoline, 3ai

^{1}H NMR (400 MHz, $CDCl_3$)

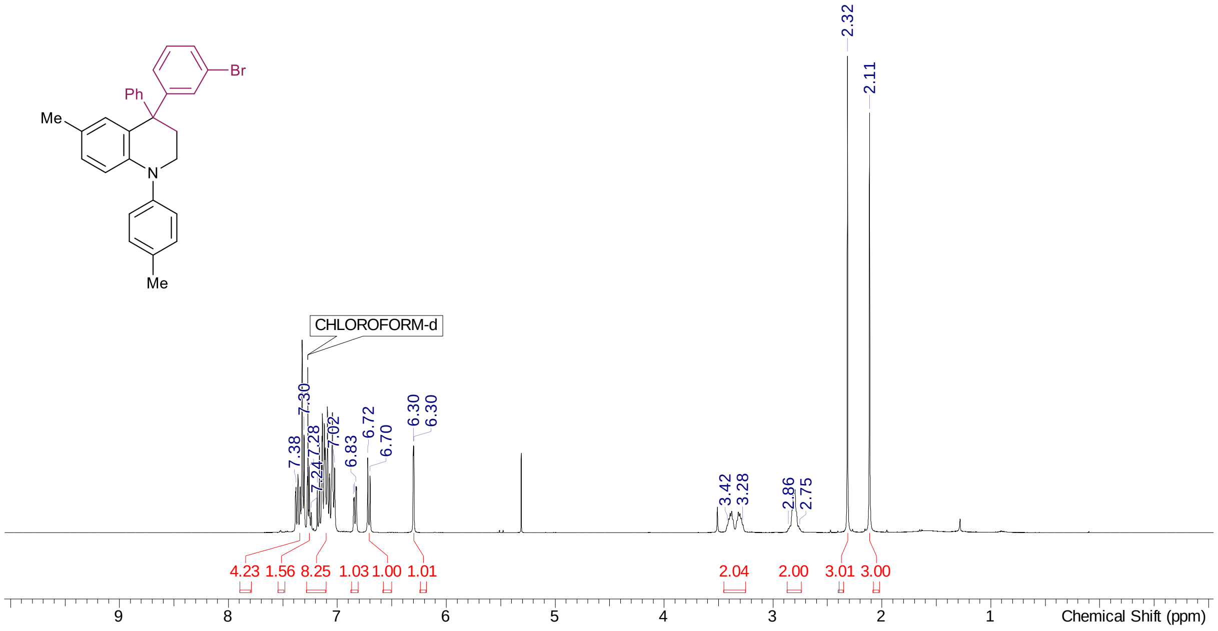

^{13}C{^{1}H} NMR ($CDCl_3$, 101 MHz)

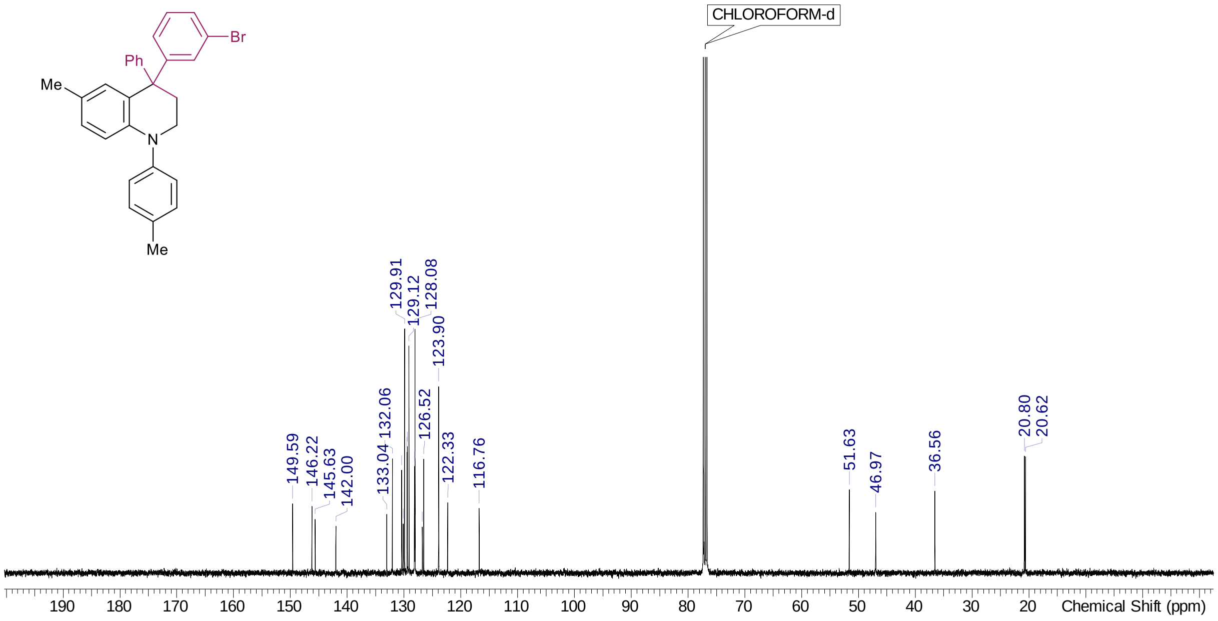

^{1}H, $^{13}C\{^{1}H\}$-HSQC NMR ($CDCl_3$, 400, 101 MHz)

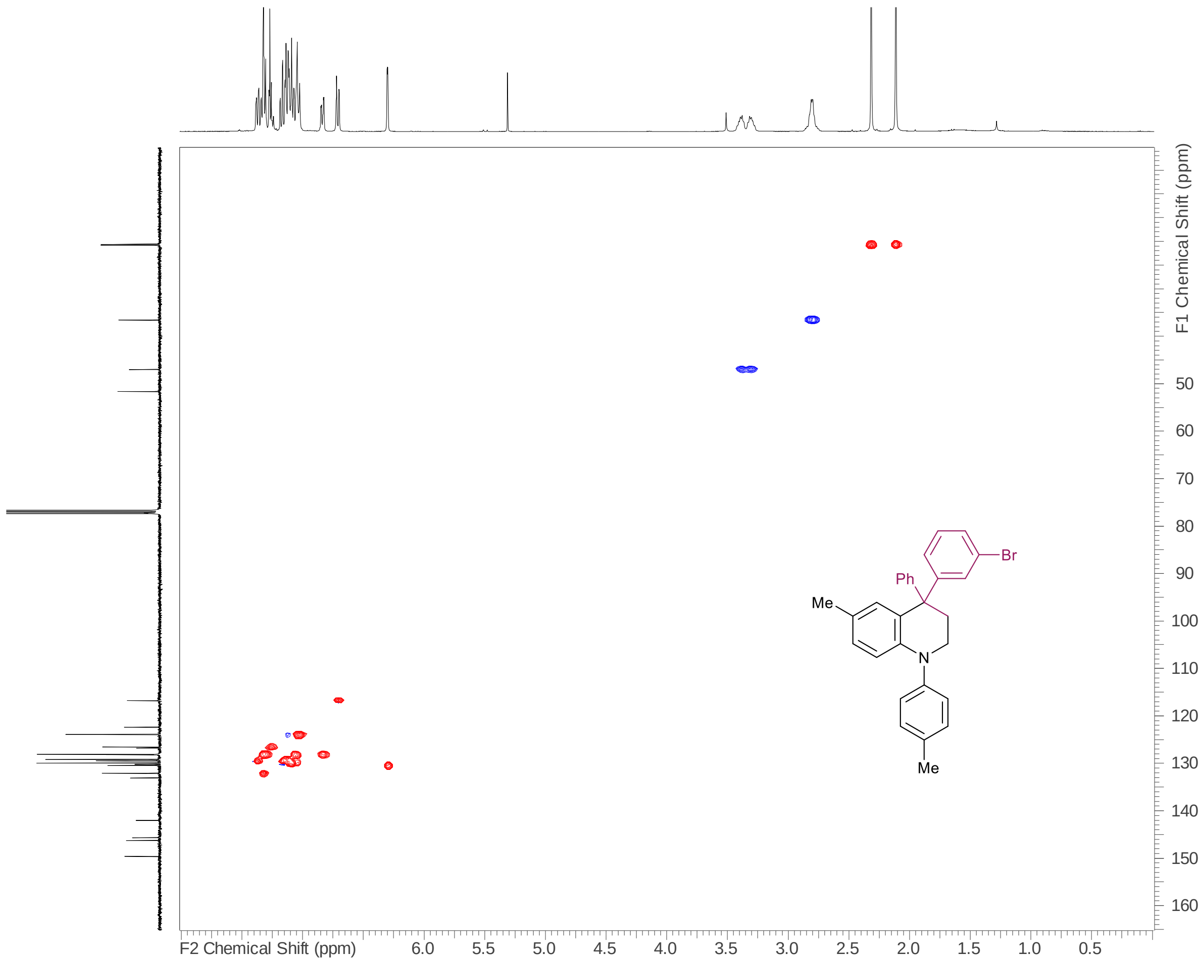

4-(3-Bromophenyl)-6-methyl-4-phenyl-1-(*p*-tolyl)-1,4-dihydroquinoline, 3ai'

^{1}H NMR (400 MHz, $CDCl_3$)

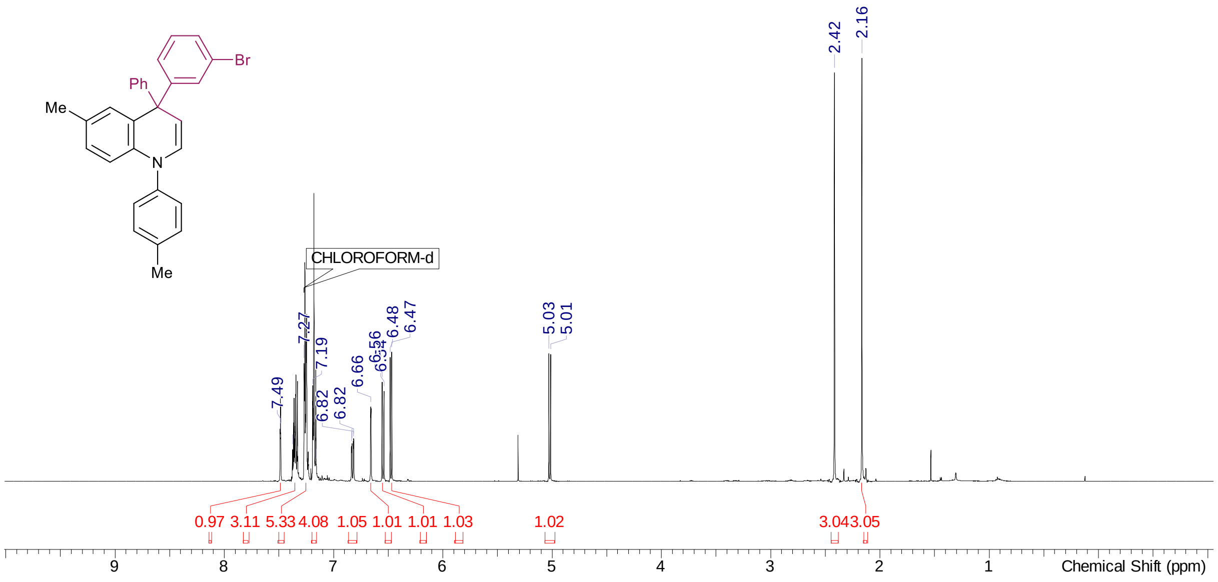

^{13}C{^{1}H} NMR ($CDCl_3$, 101 MHz)

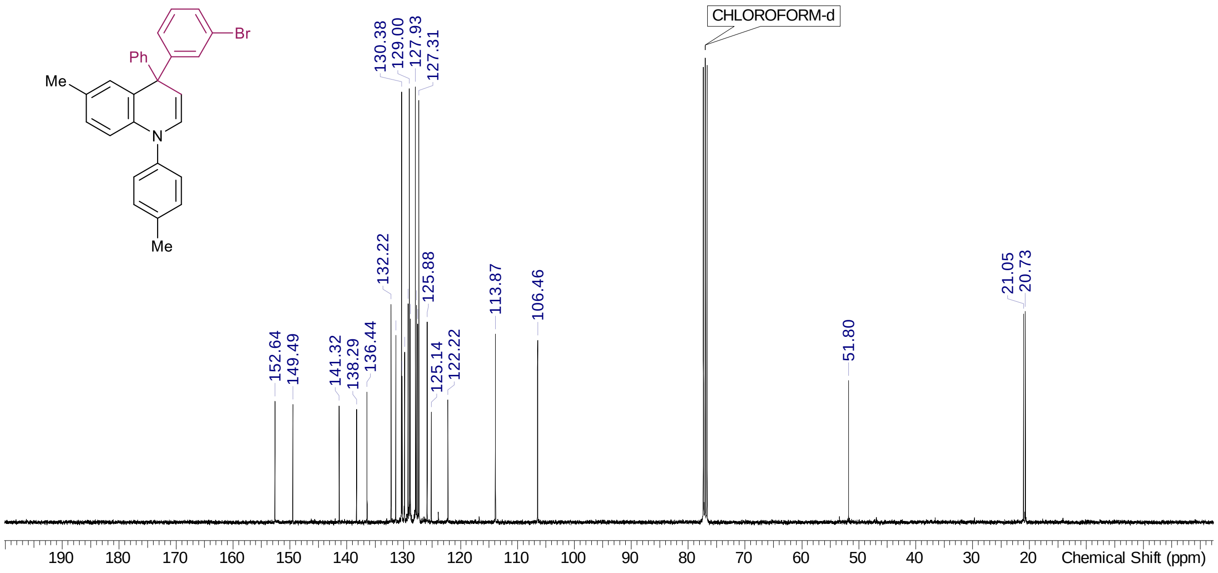

^1H, $^{13}C\{^1H\}$-HSQC NMR ($CDCl_3$, 400, 101 MHz)

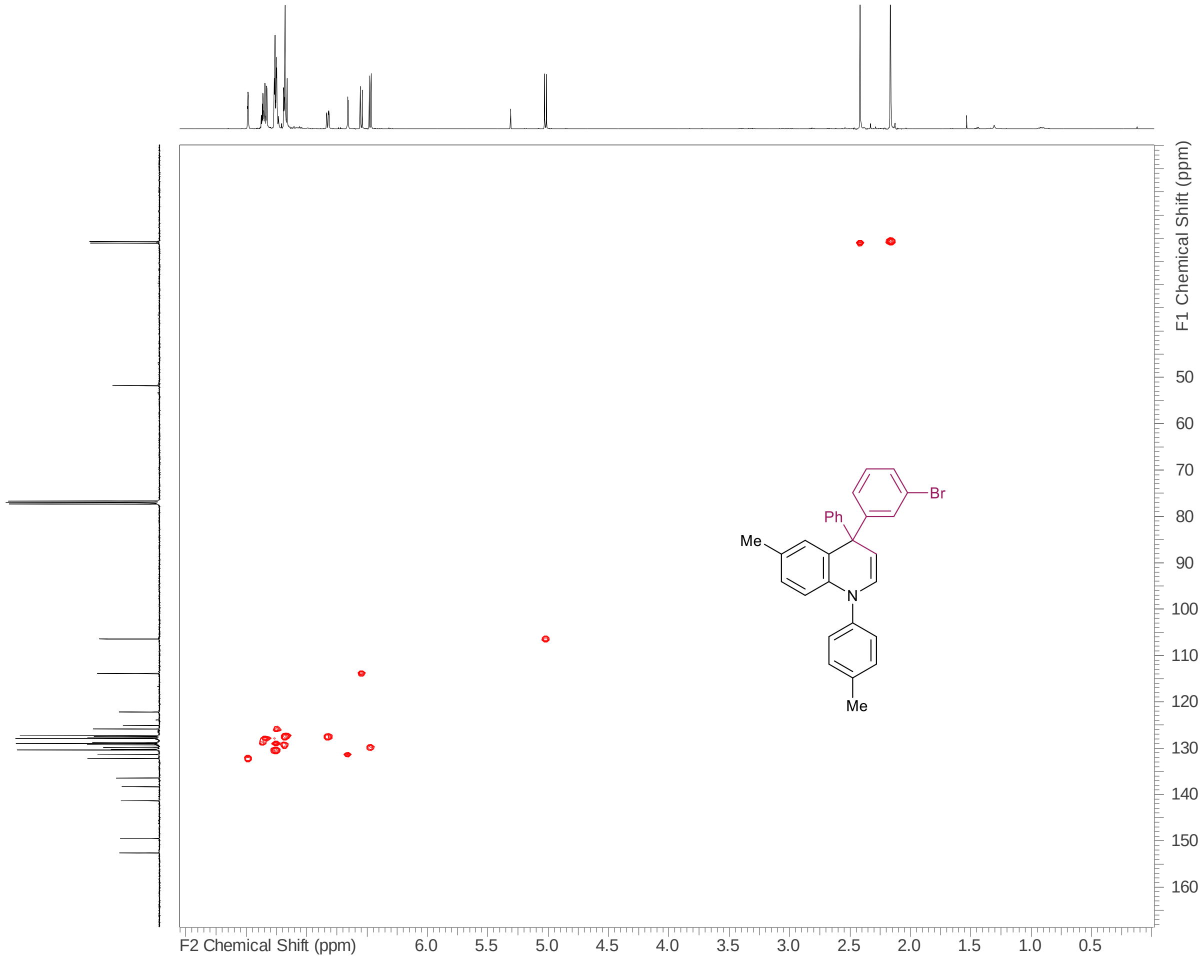

4-(4-Methoxyphenyl)-4,6-dimethyl-1-(*p*-tolyl)-1,2,3,4-tetrahydroquinoline, 3aj

^{1}H NMR (500 MHz, $CDCl_3$)

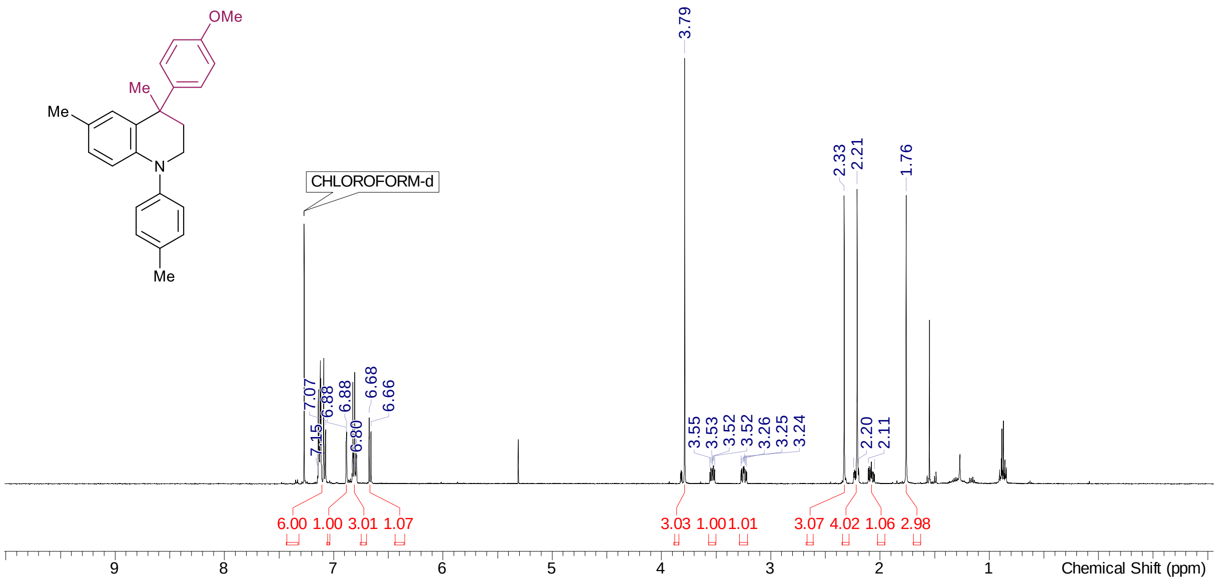

^{13}C{^{1}H} NMR ($CDCl_3$, 126 MHz)

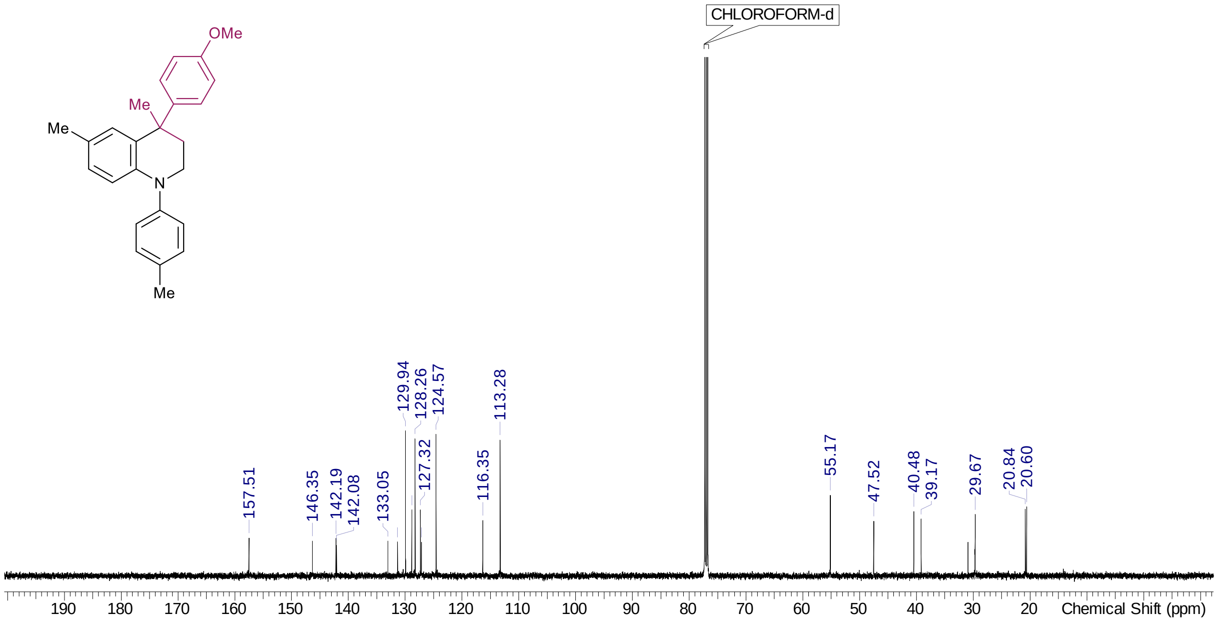

^{1}H, $^{13}C\{^{1}H\}$-HSQC NMR ($CDCl_3$, 500, 126 MHz)

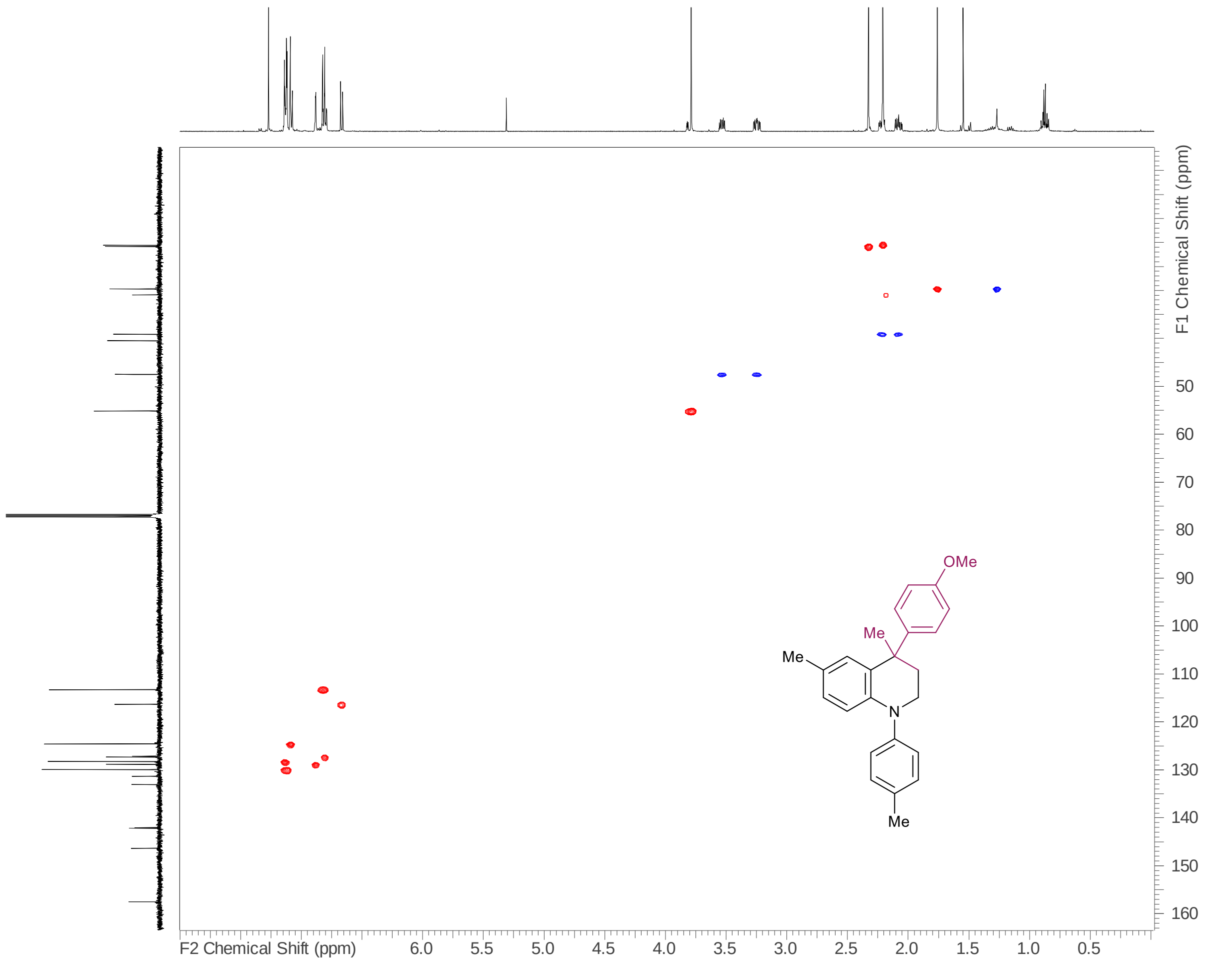

6-Methyl-1-(*p*-tolyl)-2,3-dihydro-1*H*-spiro[quinoline-4,9'-thioxanthene], 3ak

^{1}H NMR (400 MHz, $CDCl_3$)

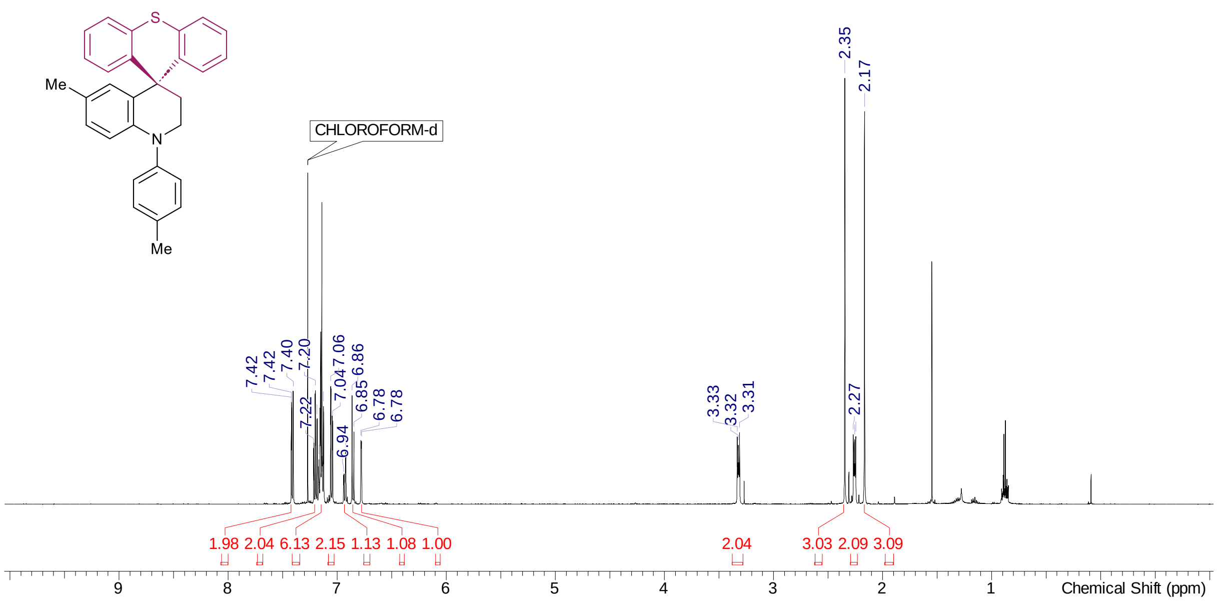

^{13}C{^{1}H} NMR ($CDCl_3$, 101 MHz)

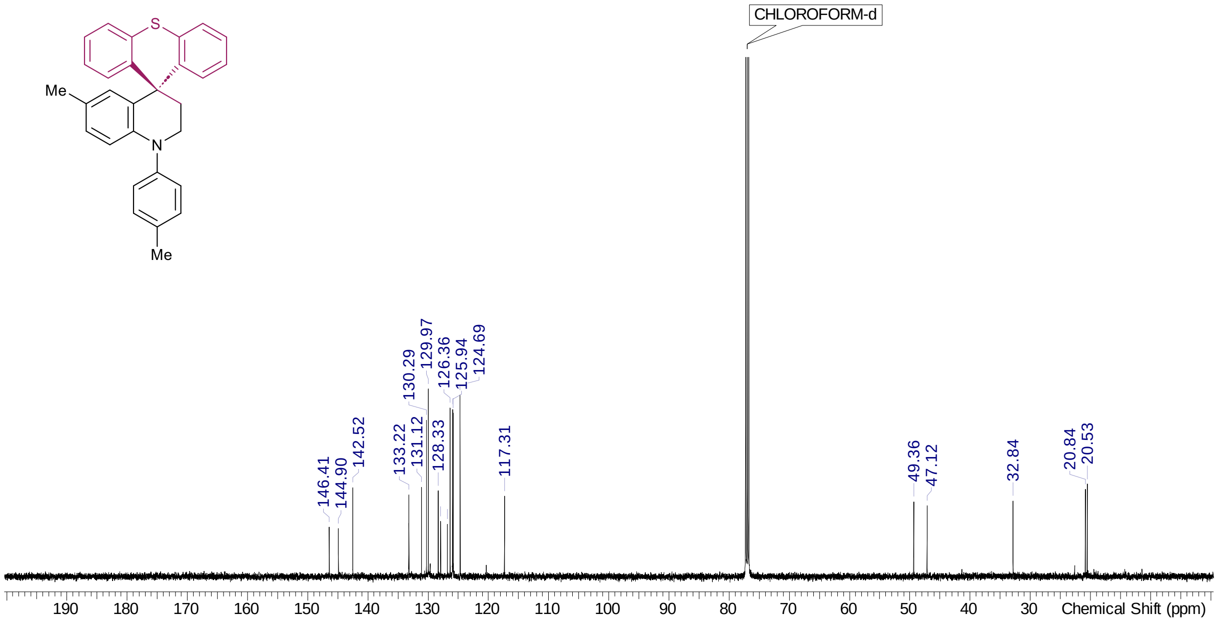

6-Methyl-1-(*p*-tolyl)-2,3-dihydro-1*H*-spiro[quinoline-4,9'-xanthene], 3al

^{1}H NMR (400 MHz, $CDCl_3$)

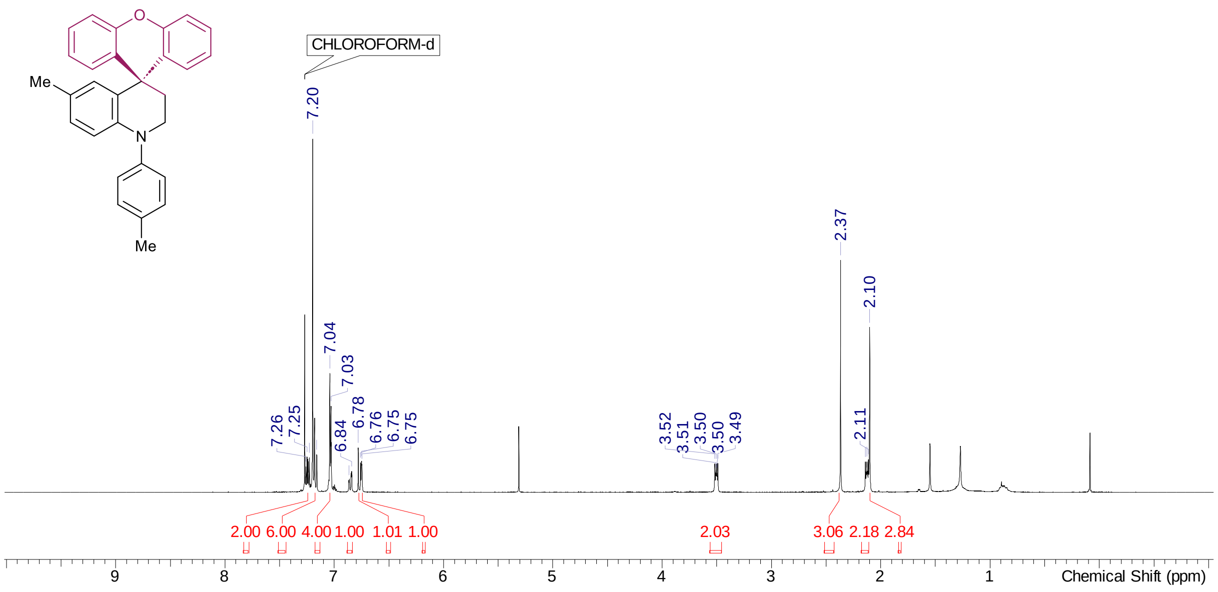

^{13}C{^{1}H} NMR ($CDCl_3$, 101 MHz)

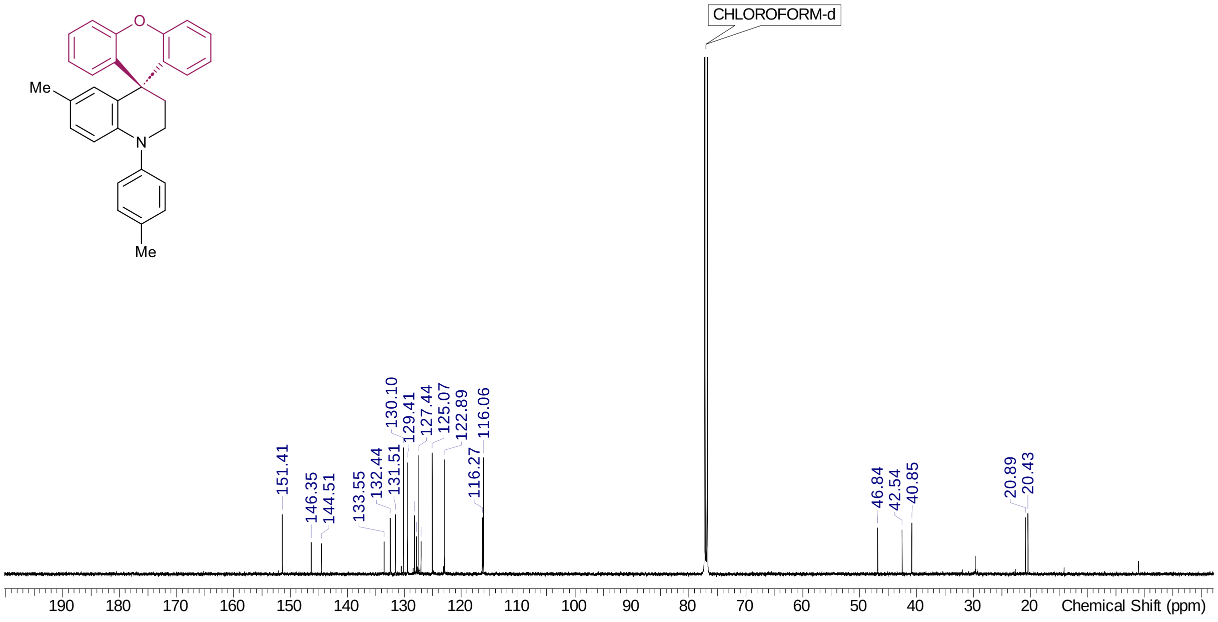

^{1}H, ^{13}C{^{1}H}-HSQC NMR ($CDCl_3$, 400, 101 MHz)

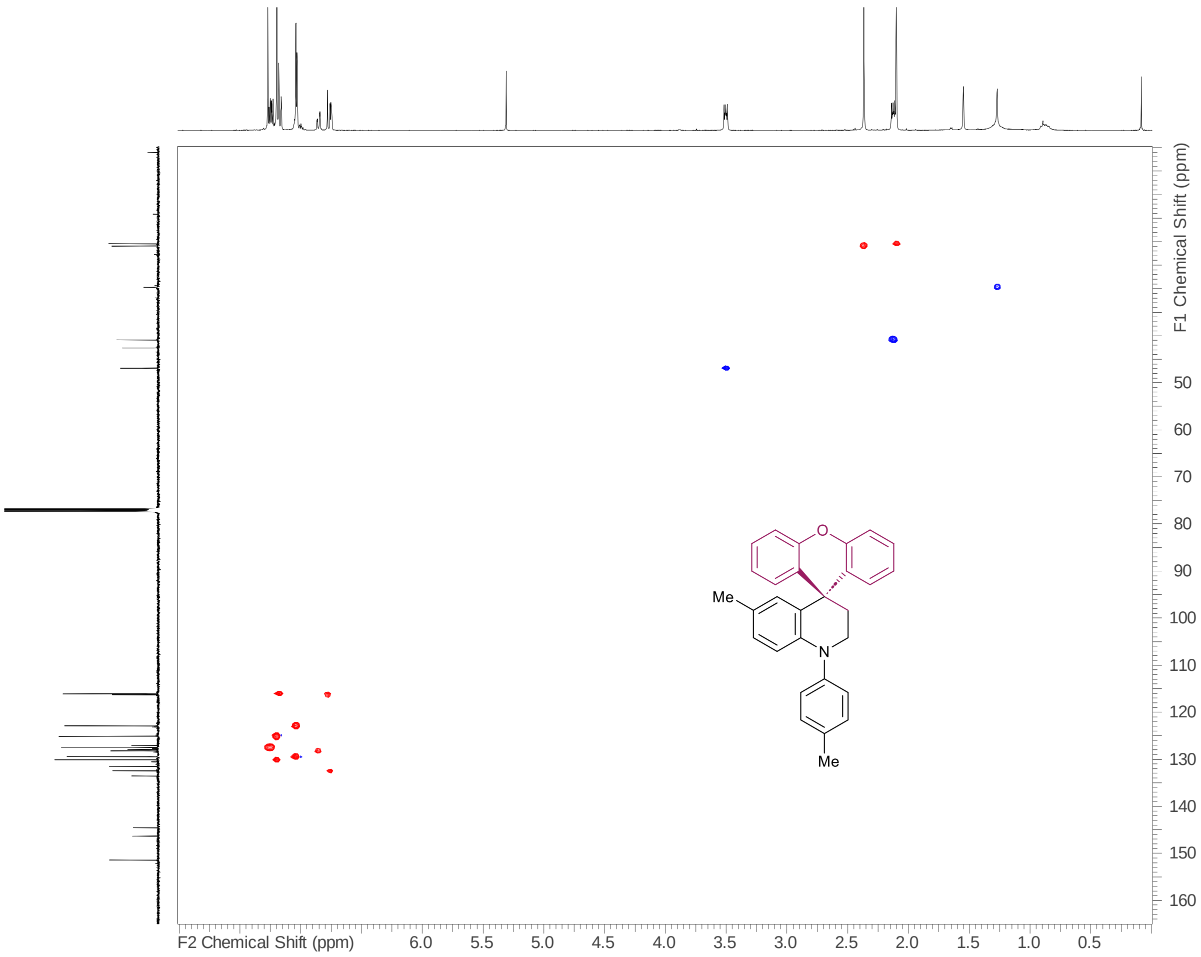

6'-Methyl-1'-(*p*-tolyl)-2',3,3',4-tetrahydro-1'*H*,2*H*-spiro[naphthalene-1,4'-quinoline], 3am

^{1}H NMR (400 MHz, $CDCl_3$)

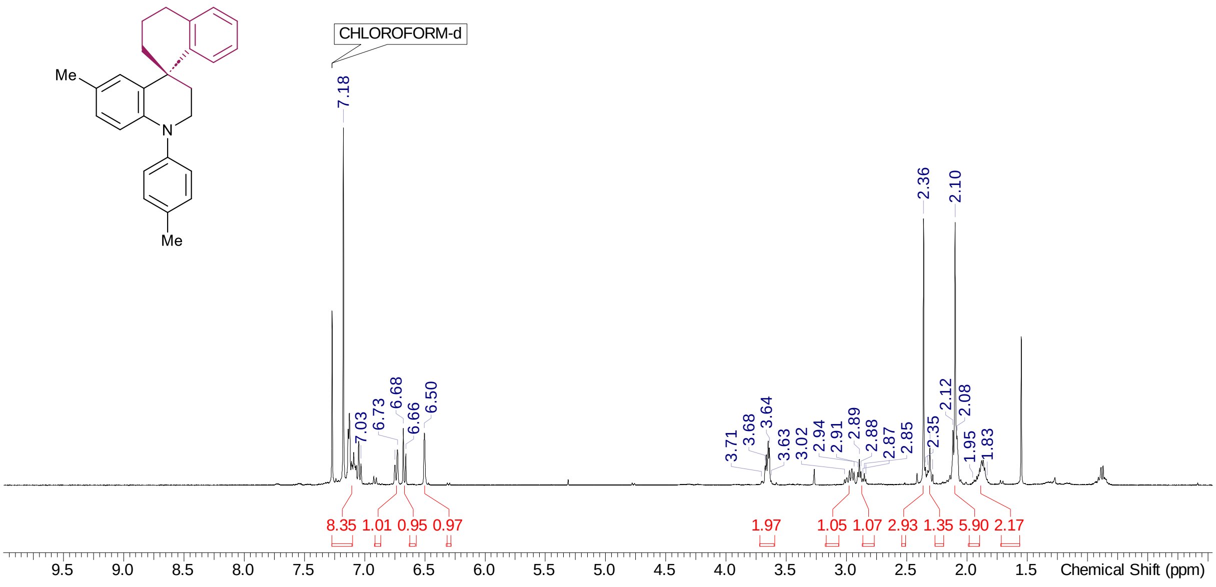

$^{13}C\{^1H\}$ NMR ($CDCl_3$, 101 MHz)

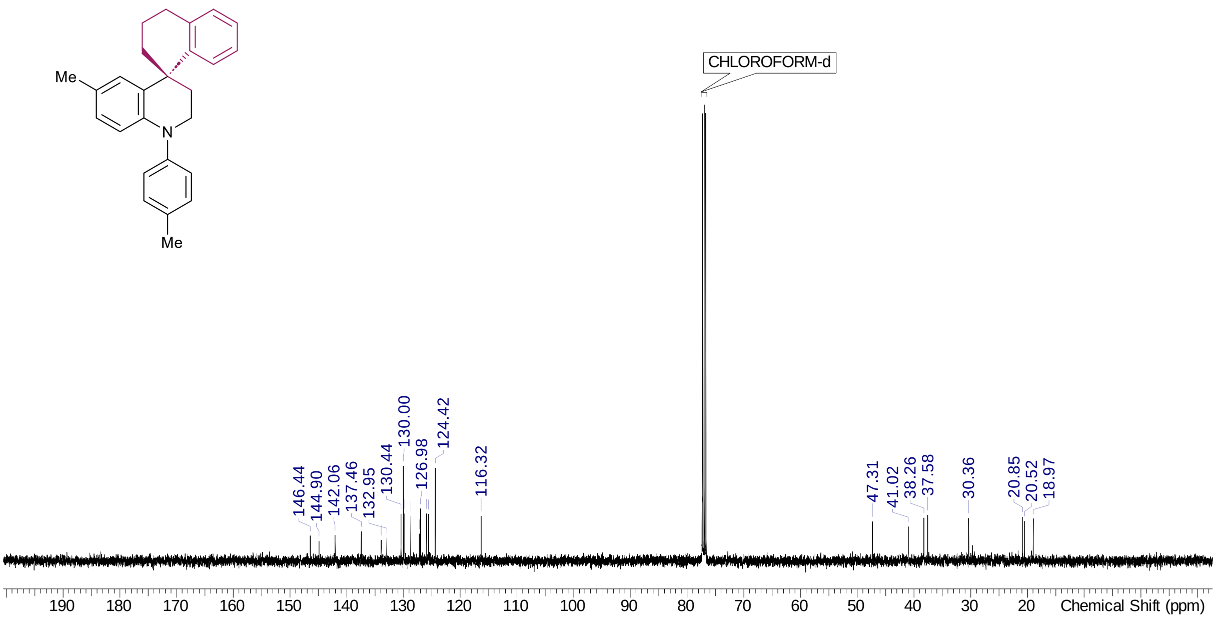

^{1}H, $^{13}C\{^{1}H\}$-HSQC NMR ($CDCl_3$, 400, 101 MHz)

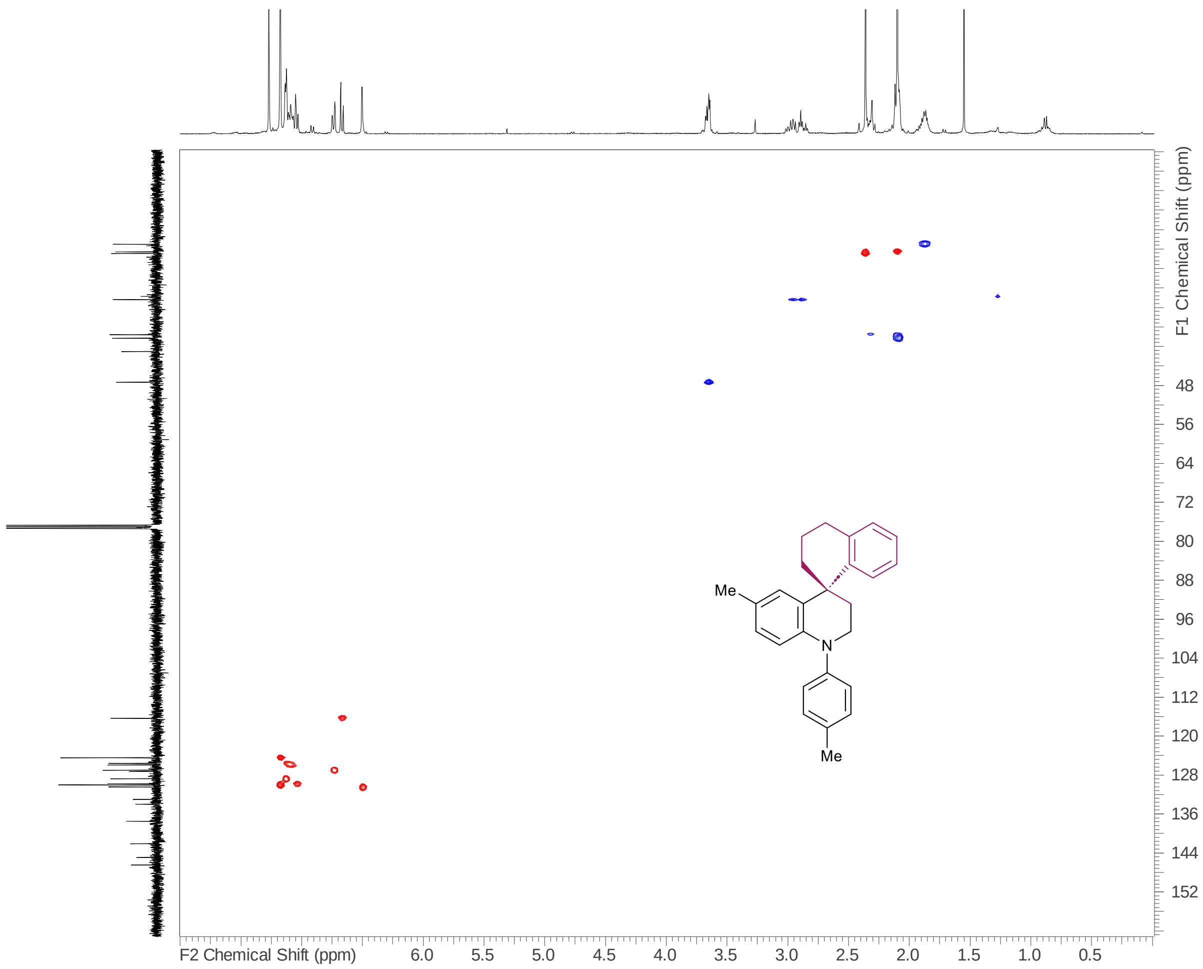

6'-Methyl-1'-(*p*-tolyl)-3,4-dihydro-1'*H*,2*H*-spiro[naphthalene-1,4'-quinoline], 3am'

^{1}H NMR (500 MHz, $CDCl_3$)

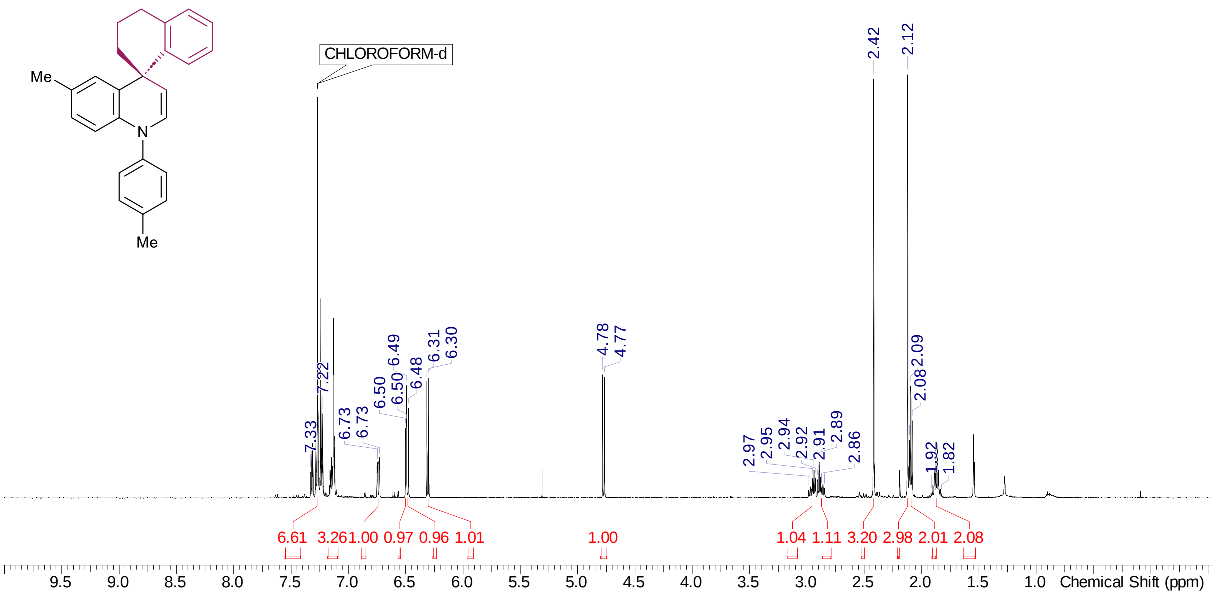

^{13}C{^{1}H} NMR ($CDCl_3$, 126 MHz)

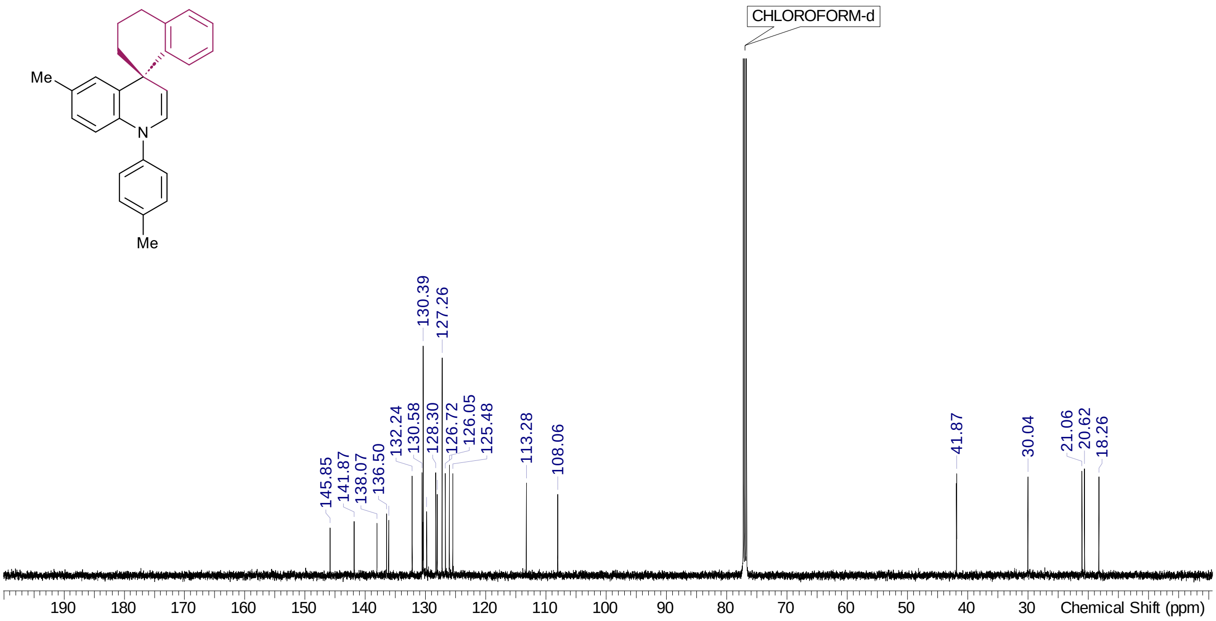

2,11b-Dimethyl-5-(*p*-tolyl)-6,6a,7,11b-tetrahydro-5*H*-indeno[2,1-c]quinoline, 3an

^{1}H NMR (500 MHz, $CDCl_3$)

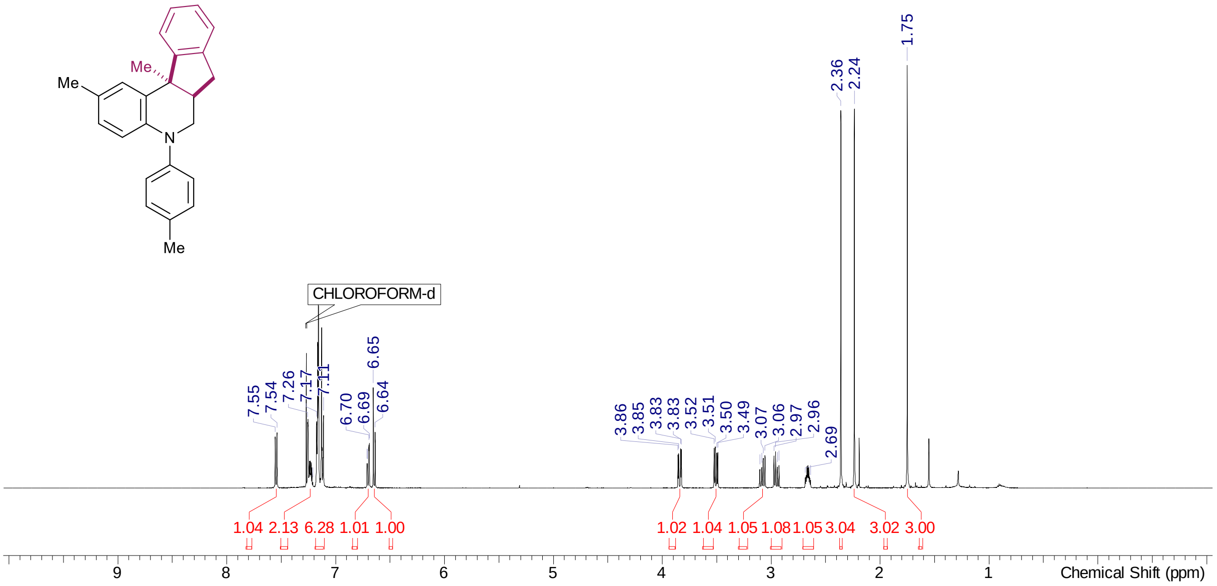

^{13}C{^{1}H} NMR ($CDCl_3$, 126 MHz)

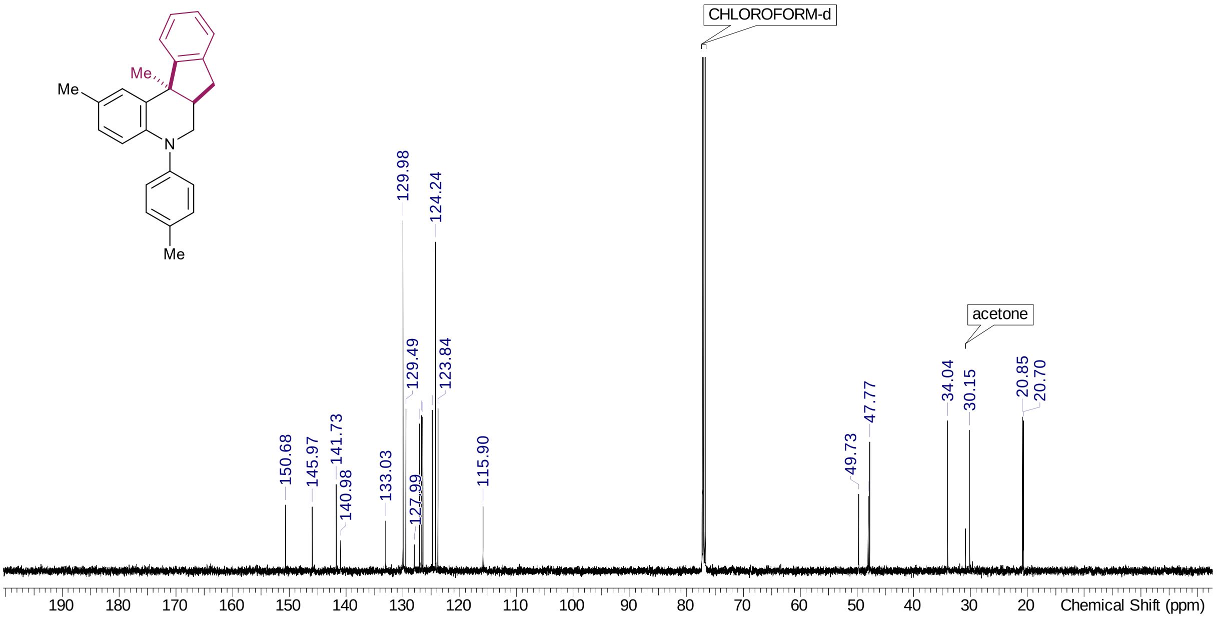

^{1}H, ^{13}C{^{1}H}-HSQC NMR (CDCl$_3$, 500, 126 MHz)

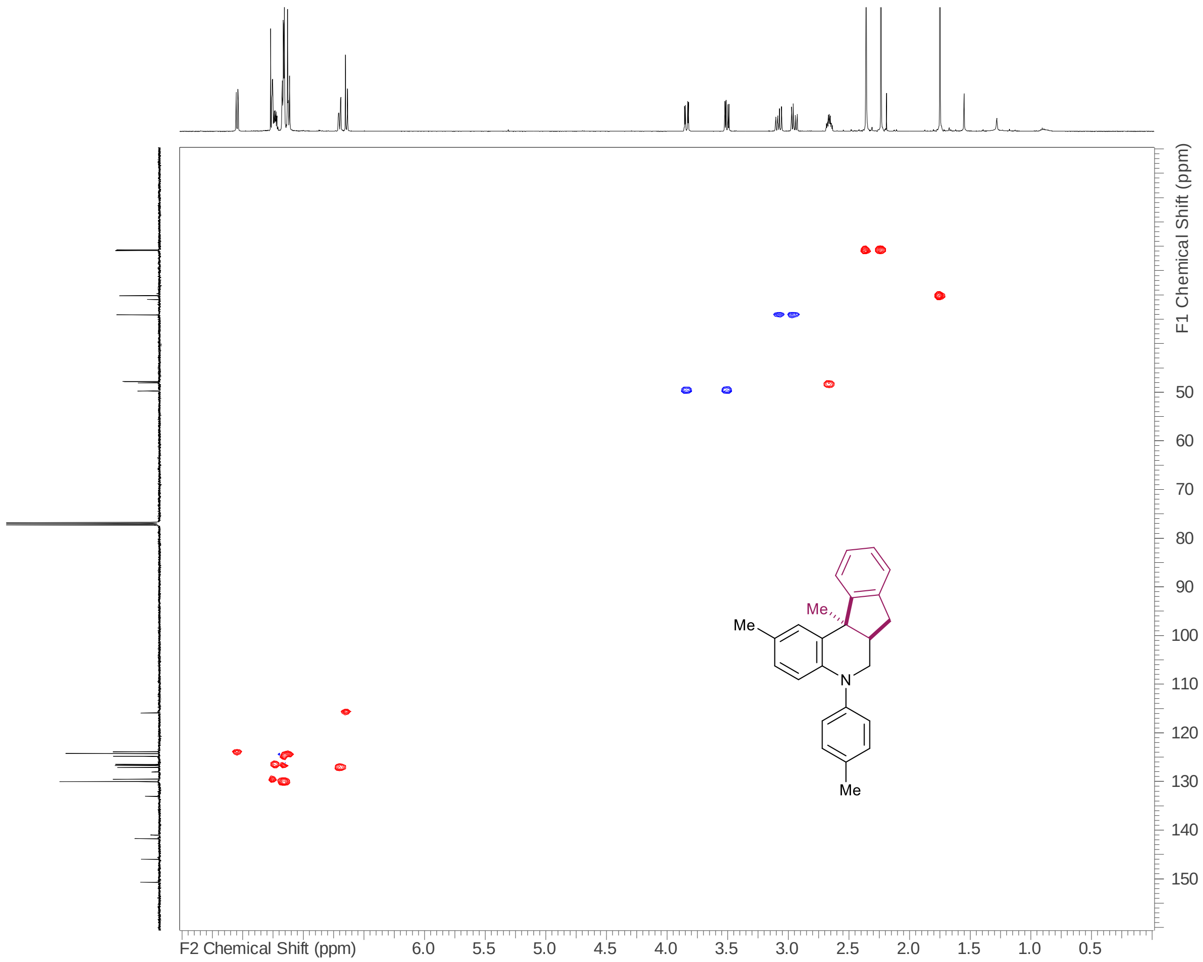

1-Benzhydryl-6-(4-chloro-3,5-dimethylphenoxy)-4-methyl-4-((trimethylsilyl)methyl)-1,2,3,4-tetrahydroquinoline, 3ao

^{1}H NMR (500 MHz, $CDCl_3$)

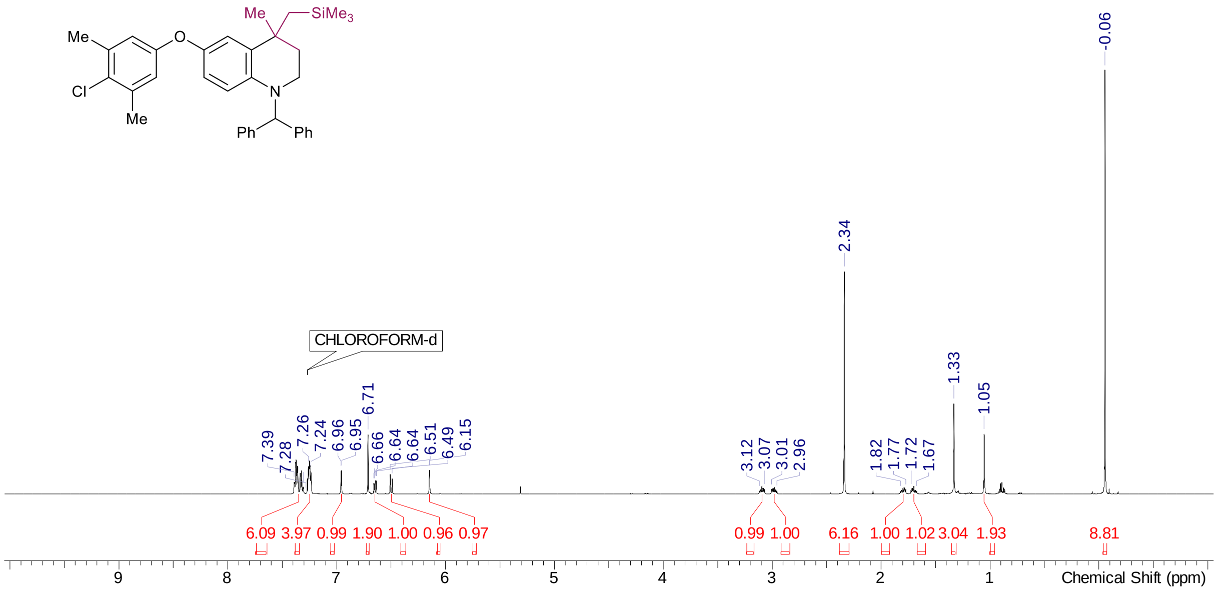

$^{13}C\{^{1}H\}$ NMR ($CDCl_3$, 126 MHz)

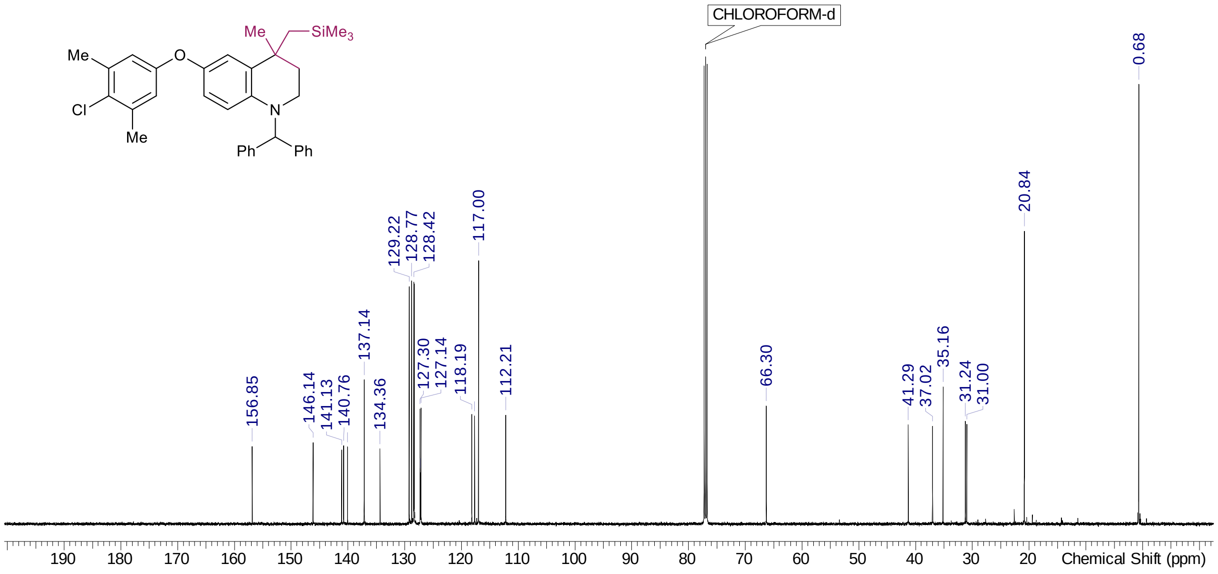

^{1}H, $^{13}C\{^{1}H\}$-HSQC NMR ($CDCl_3$, 500, 126 MHz)

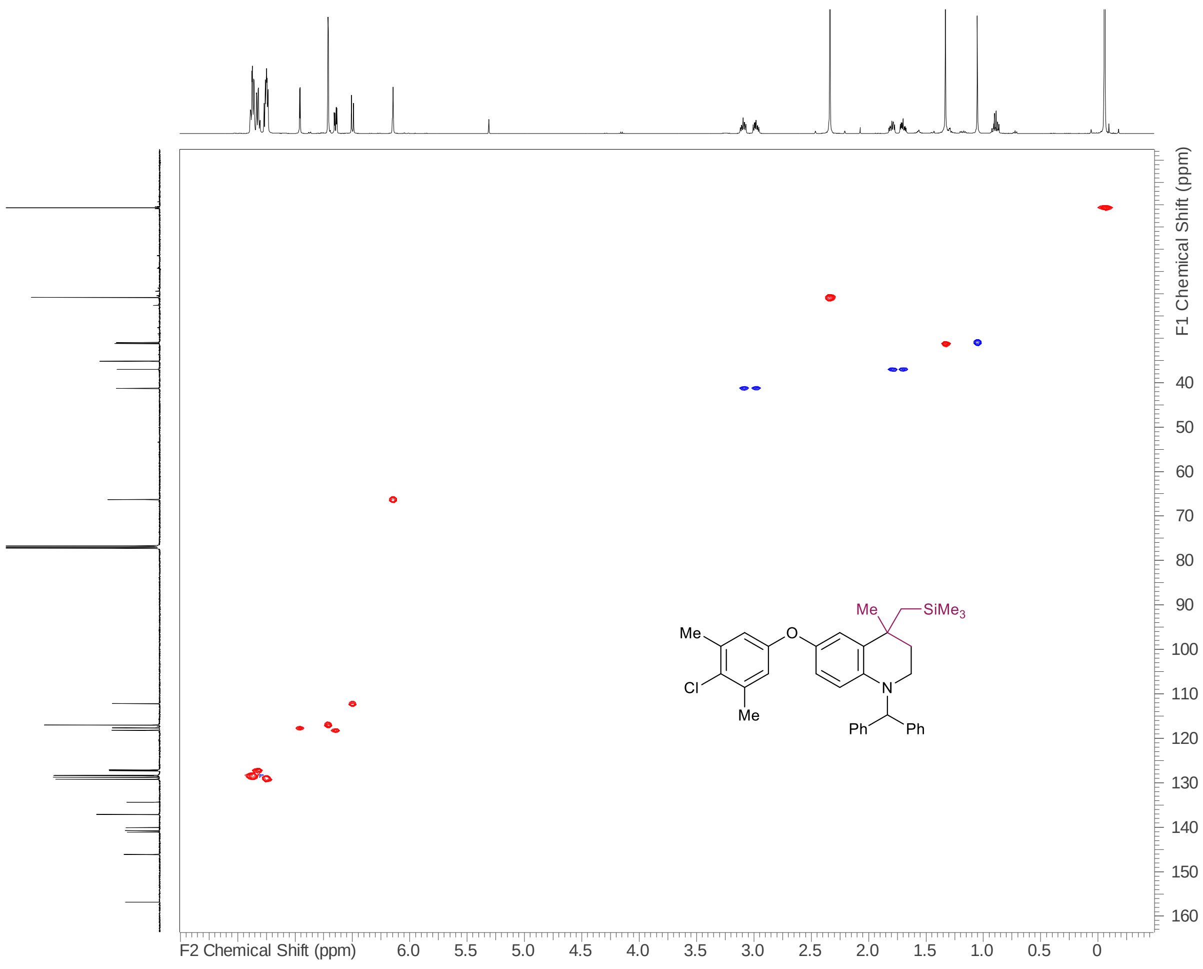

1-((4*S*)-4-(3,4-Dichlorophenyl)-1,2,3,4-tetrahydronaphthalen-1-yl)-4,6-dimethyl-4-((trimethylsilyl)methyl)-1,2,3,4-tetrahydroquinoline, 3ap 1:5 *dr*

^{1}H NMR (400 MHz, CDCl$_3$)

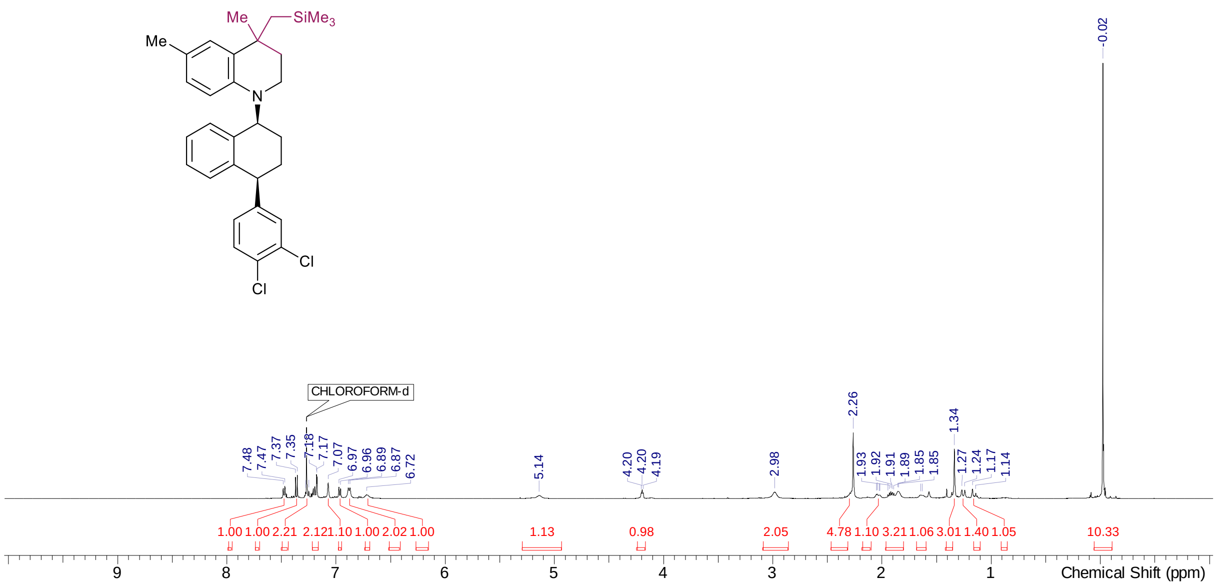

^{13}C{^{1}H} NMR (CDCl$_3$, 101 MHz)

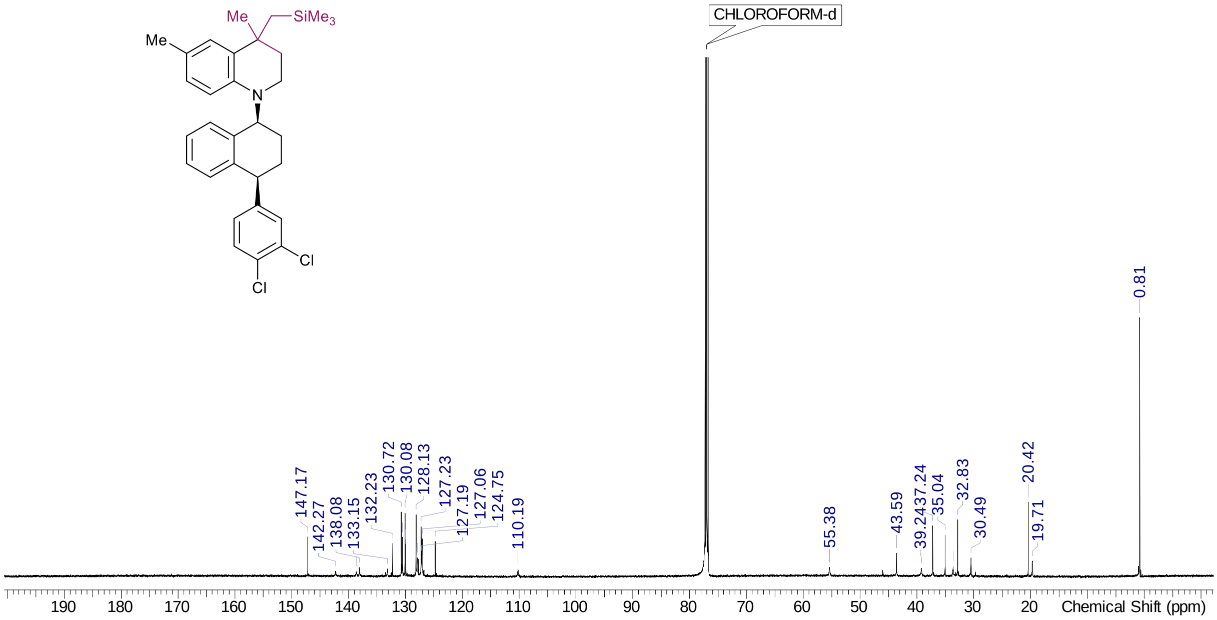

^{1}H, $^{13}C\{^{1}H\}$-HSQC NMR ($CDCl_3$, 400, 101 MHz)

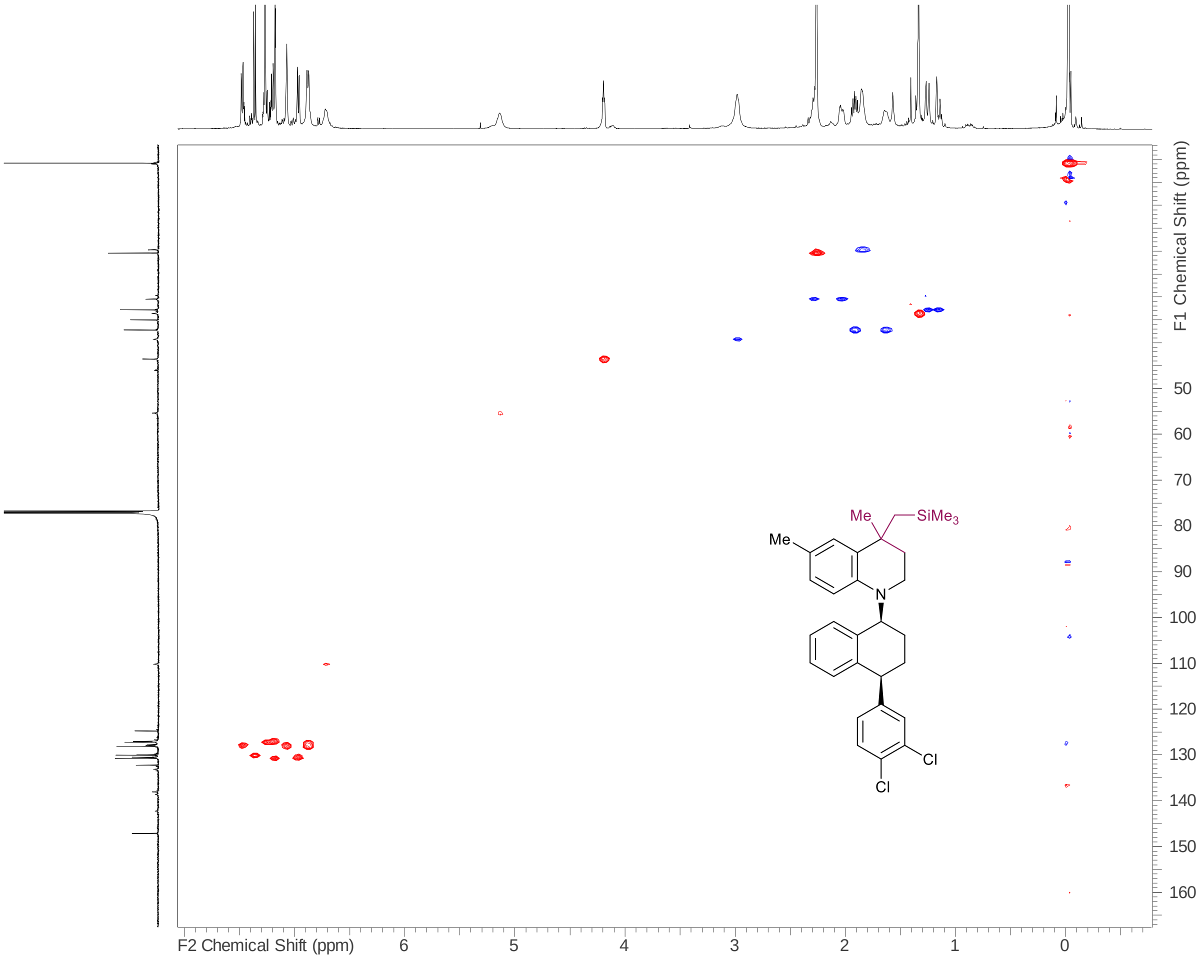

2-Chloro-*N*-methyl-*N*-(3-methyl-4-(trimethylsilyl)butyl)aniline, 4a

^{1}H NMR (400 MHz, $CDCl_3$)

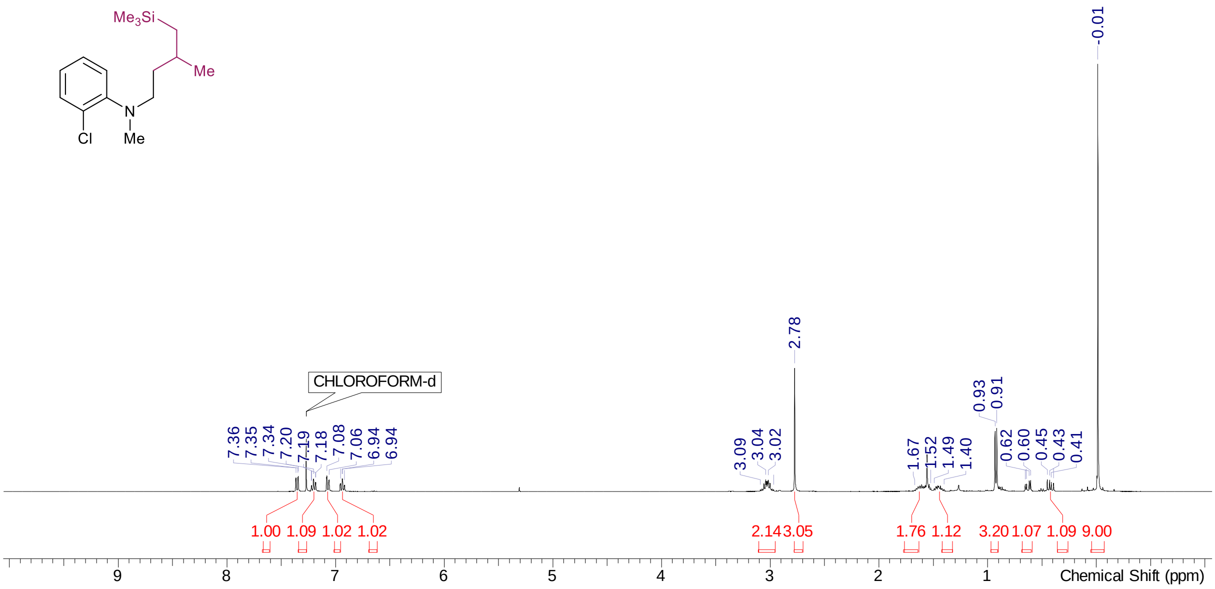

^{13}C{^{1}H} NMR ($CDCl_3$, 101 MHz)

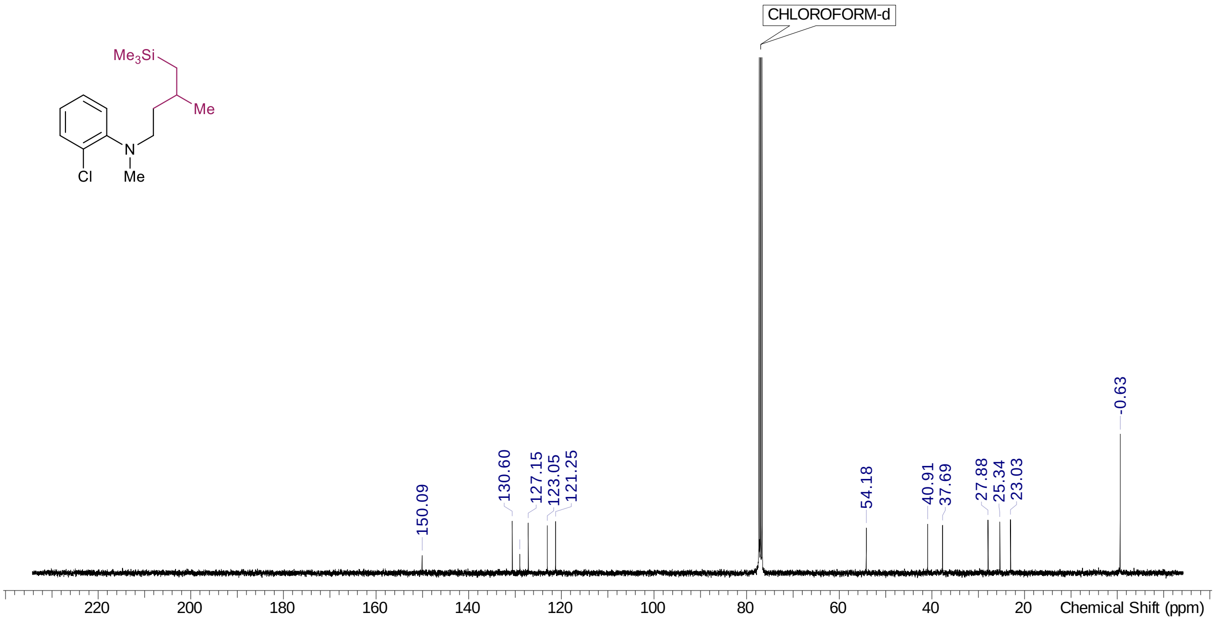

^{1}H, ^{13}C{^{1}H}-HSQC NMR ($CDCl_3$, 400, 101 MHz)

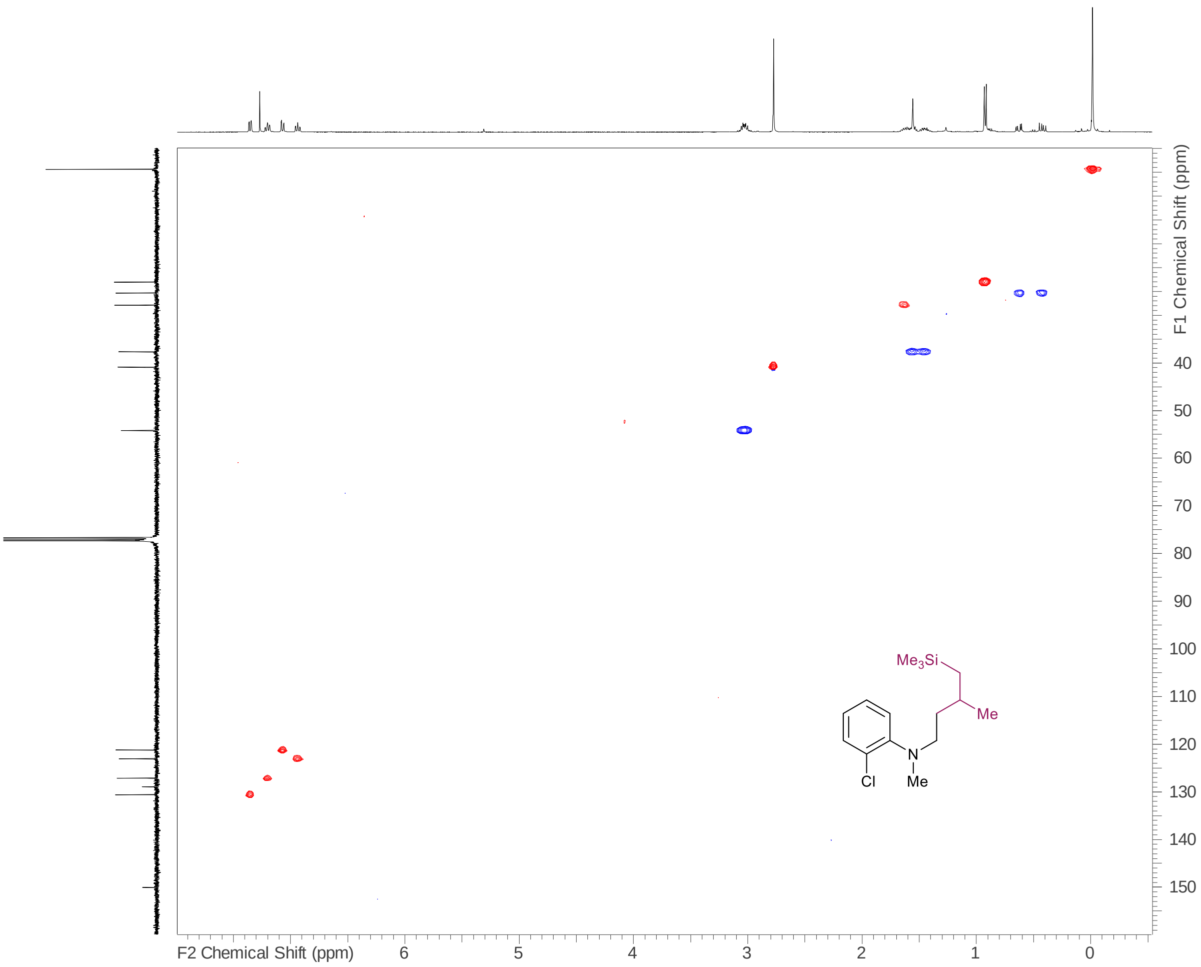

_N_-Methyl-_N_-(3-methyl-4-(trimethylsilyl)butyl)-2-phenoxyaniline, 4b

^{1}H NMR (400 MHz, $CDCl_3$)

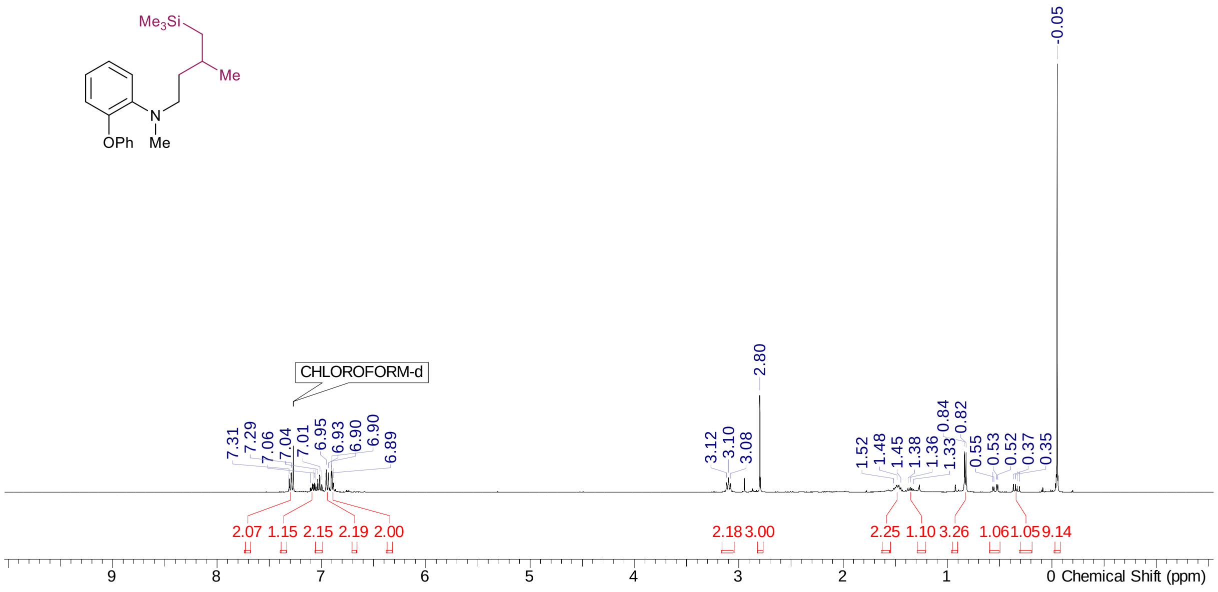

^{13}C{^{1}H} NMR ($CDCl_3$, 101 MHz)

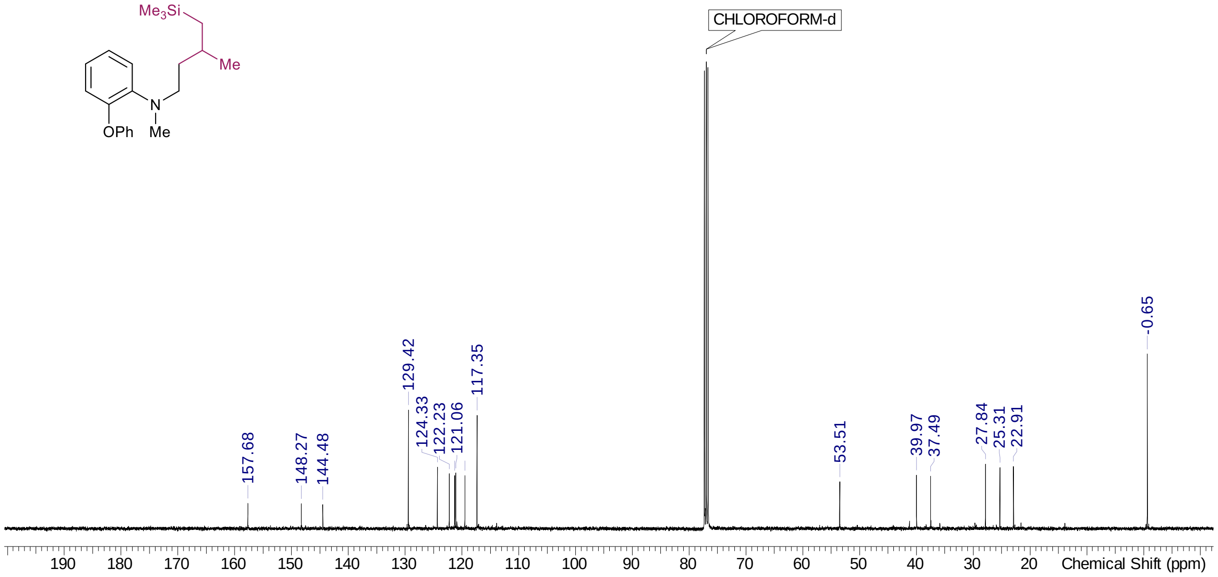

^{1}H, ^{13}C{^{1}H}-HSQC NMR ($CDCl_3$, 400, 101 MHz)

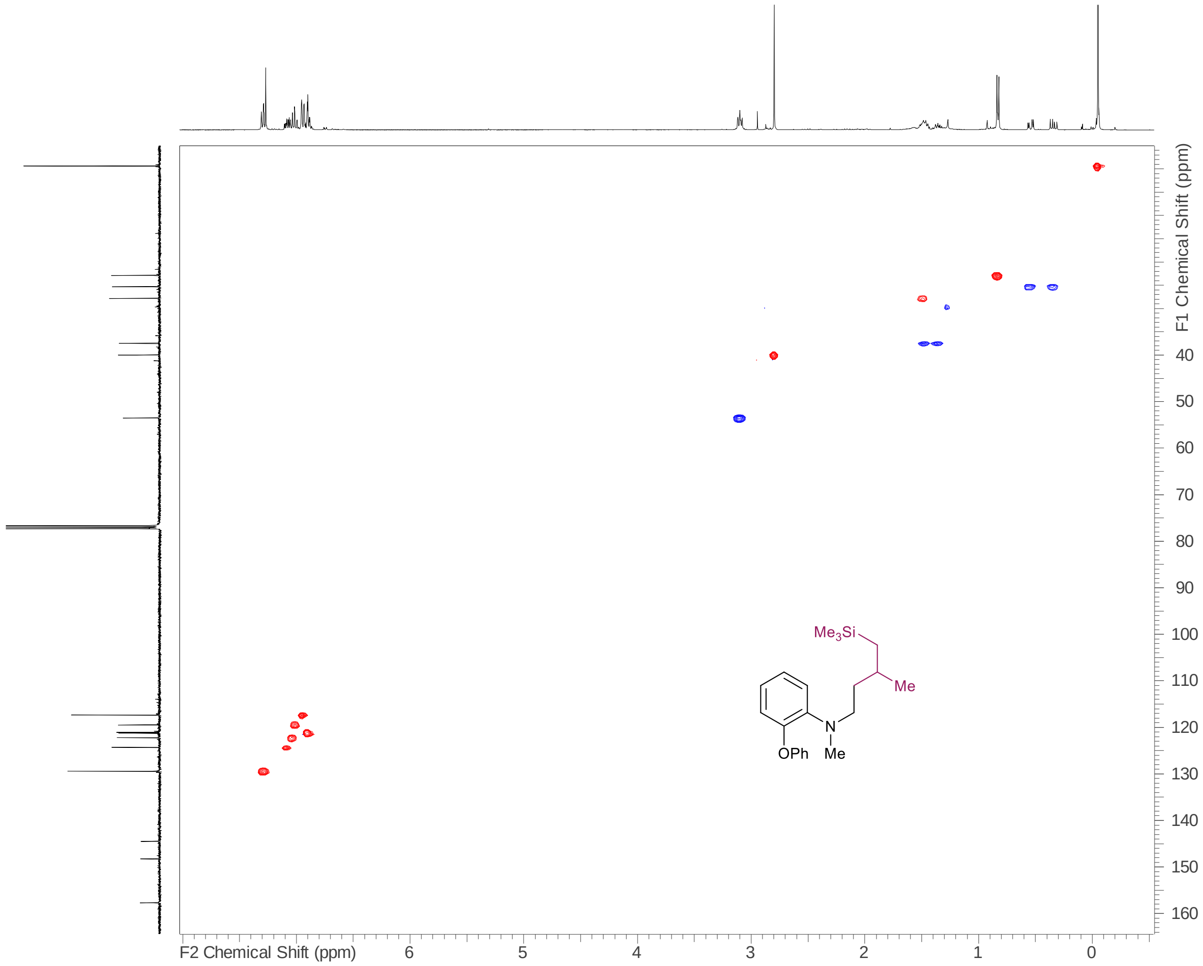

1-Benzyl-4-methyl-4-((trimethylsilyl)methyl)-1,2,3,4-tetrahydroquinoline-6-carbaldehyde, 9a

^{1}H NMR (400 MHz, $CDCl_3$)

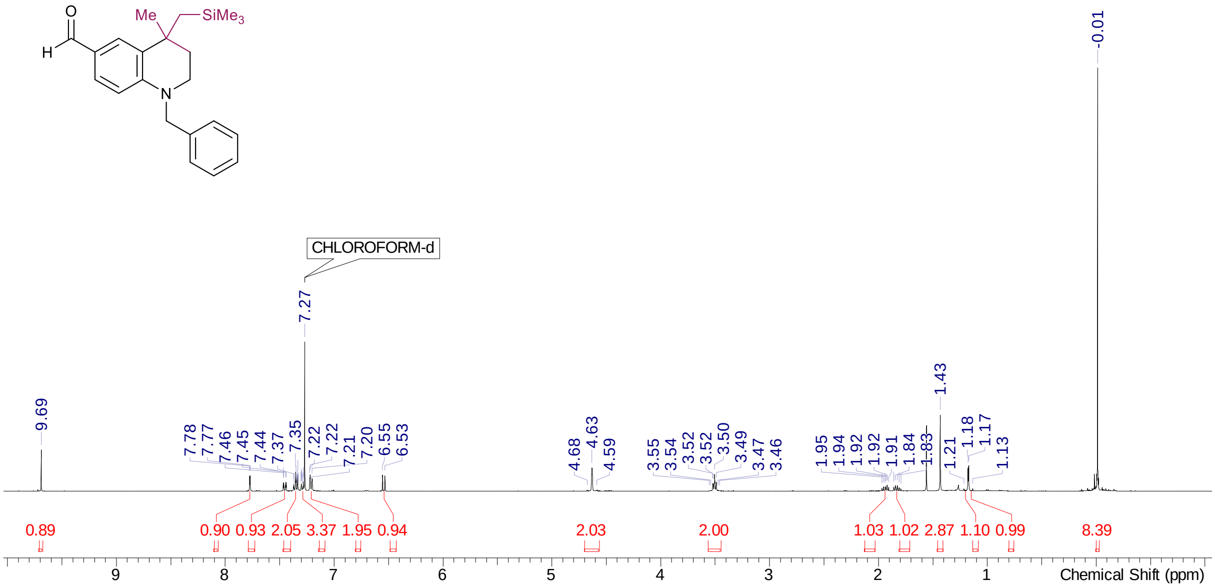

$^{13}C\{^{1}H\}$ NMR ($CDCl_3$, 101 MHz)

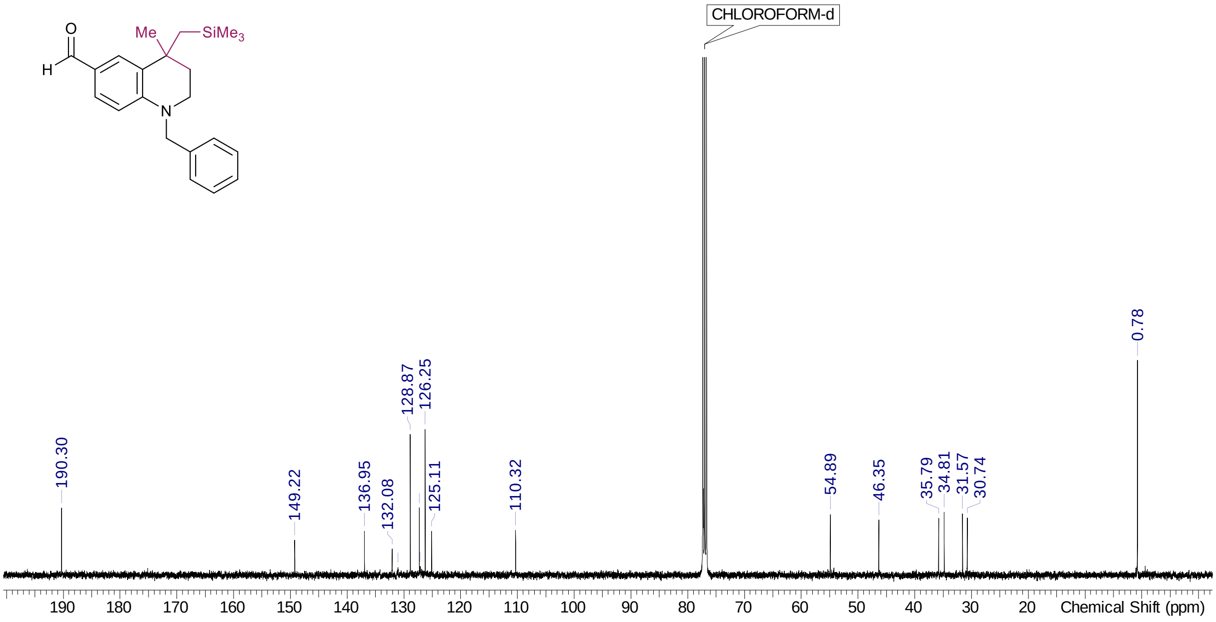

^{1}H, ^{13}C{^{1}H}-HSQC NMR ($CDCl_3$, 400, 101 MHz)

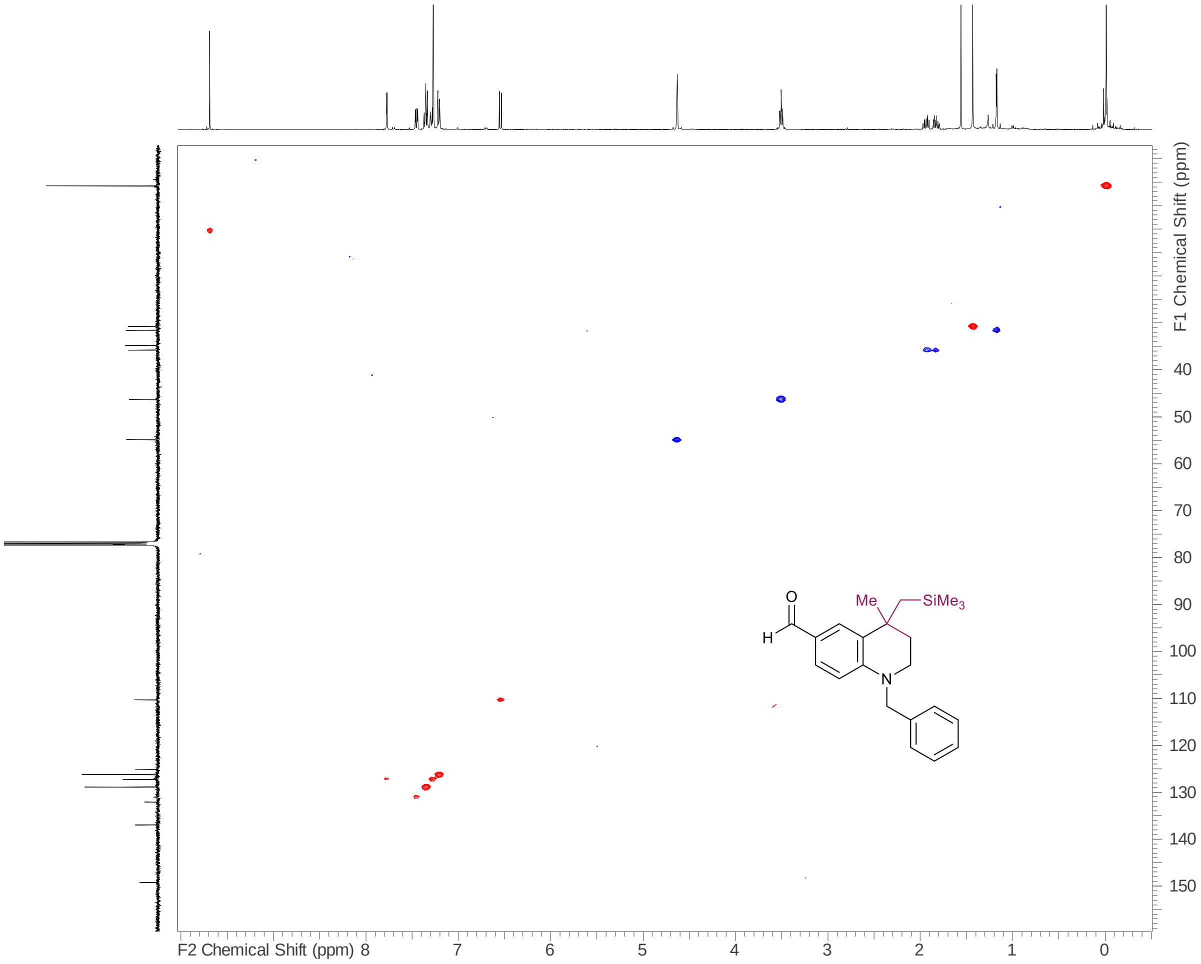

1-Benzhydryl-4-methyl-4-((trimethylsilyl)methyl)-1,2,3,4-tetrahydroquinoline-6-carbaldehyd, 9b

^{1}H NMR (400 MHz, $CDCl_3$)

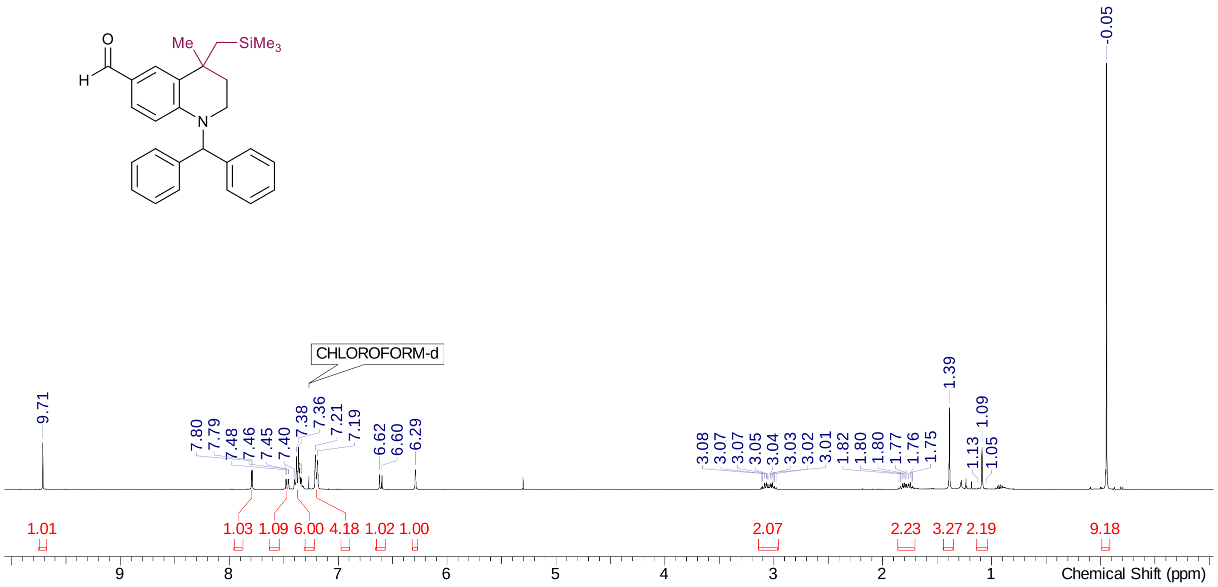

^{13}C{^{1}H} NMR ($CDCl_3$, 101 MHz)

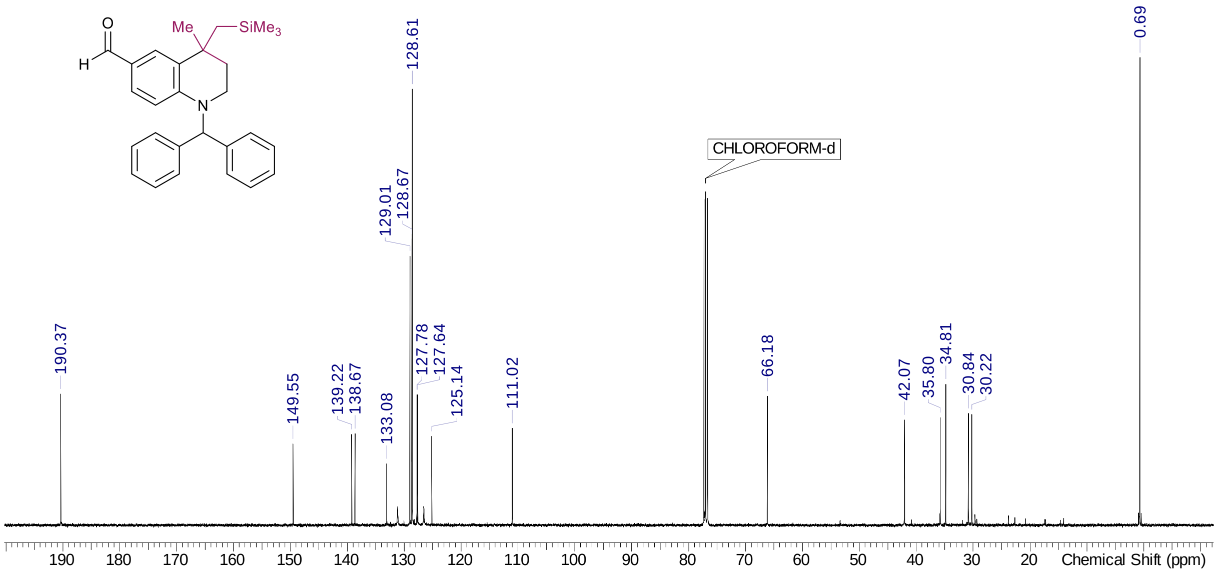

^{1}H, ^{13}C{^{1}H}-HSQC NMR ($CDCl_3$, 400, 101 MHz)

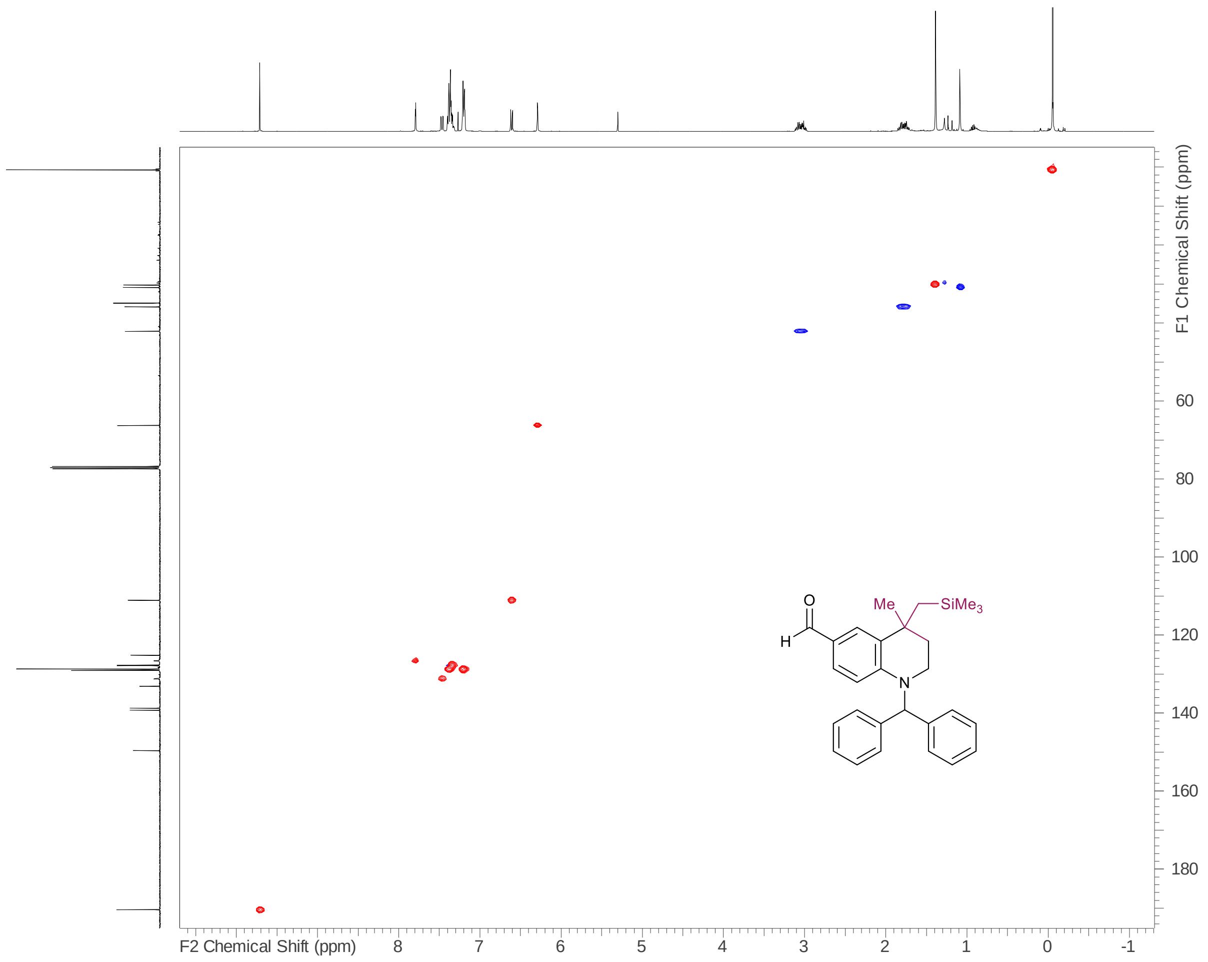

1-Benzhydryl-4,6-dimethyl-4-((trimethylsilyl)methyl)-1,4-dihydroquinolin, 10

^{1}H NMR (400 MHz, $CDCl_3$)

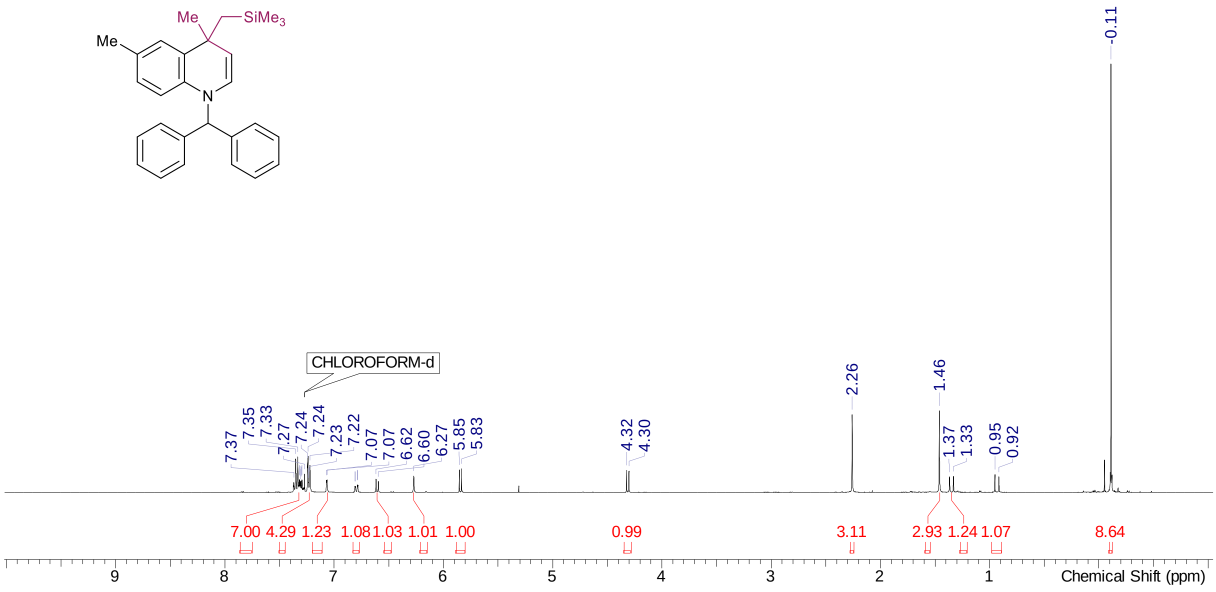

$^{13}C\{^1H\}$ NMR ($CDCl_3$, 101 MHz)

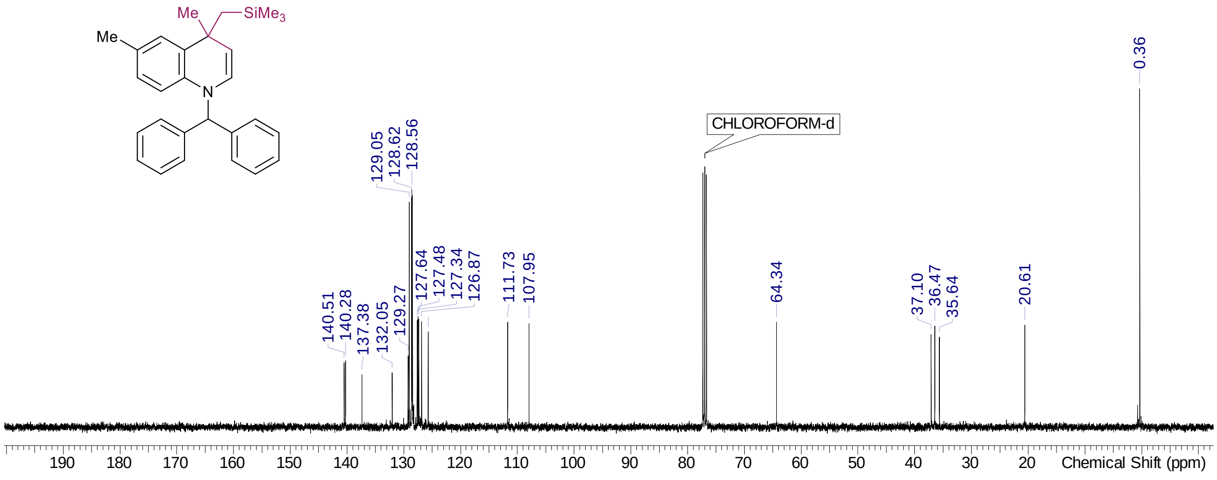

^1H, $^{13}C\{^1H\}$-HSQC NMR ($CDCl_3$, 400, 101 MHz)

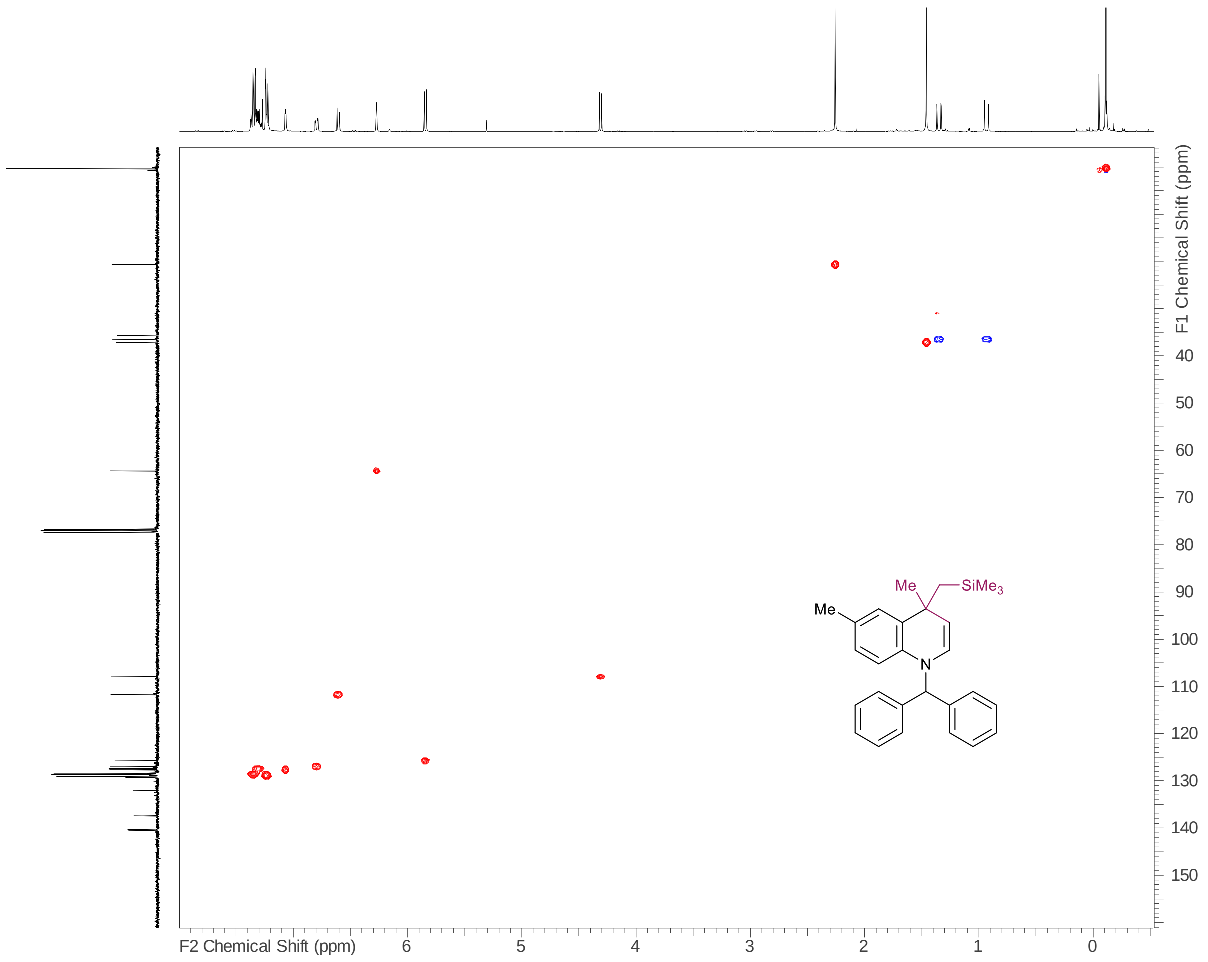

1-Benzyl-4,6-dimethyl-4-((trimethylsilyl)methyl)-3,4-dihydroquinolin-2(1*H*)-one, 11

^{1}H NMR (400 MHz, $CDCl_3$)

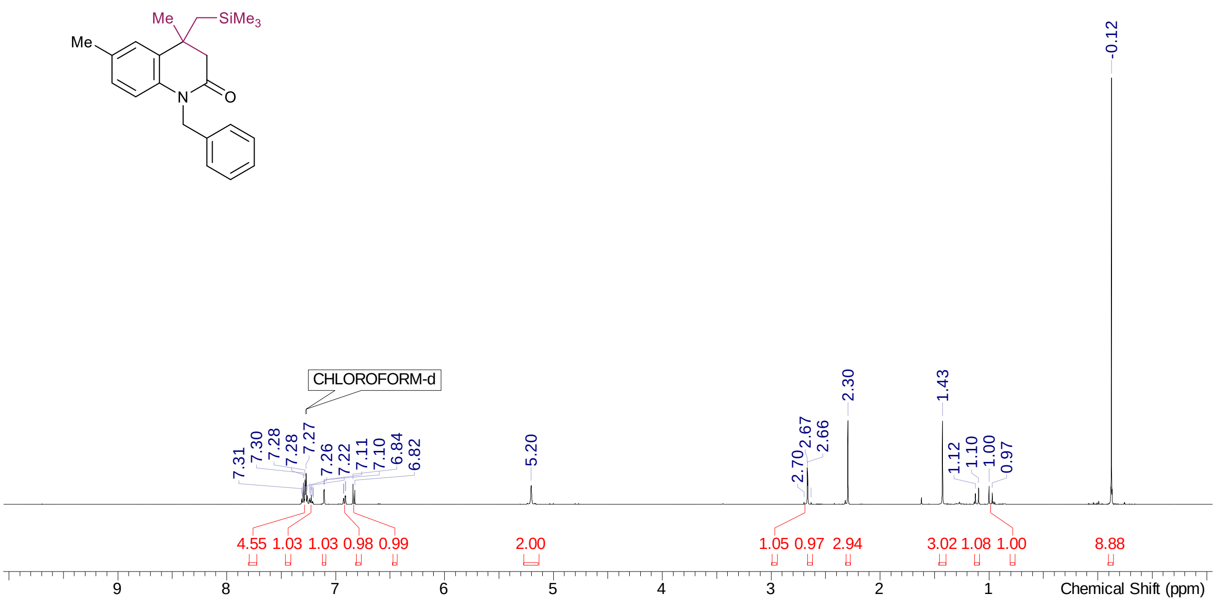

^{13}C{^{1}H} NMR ($CDCl_3$, 101 MHz)

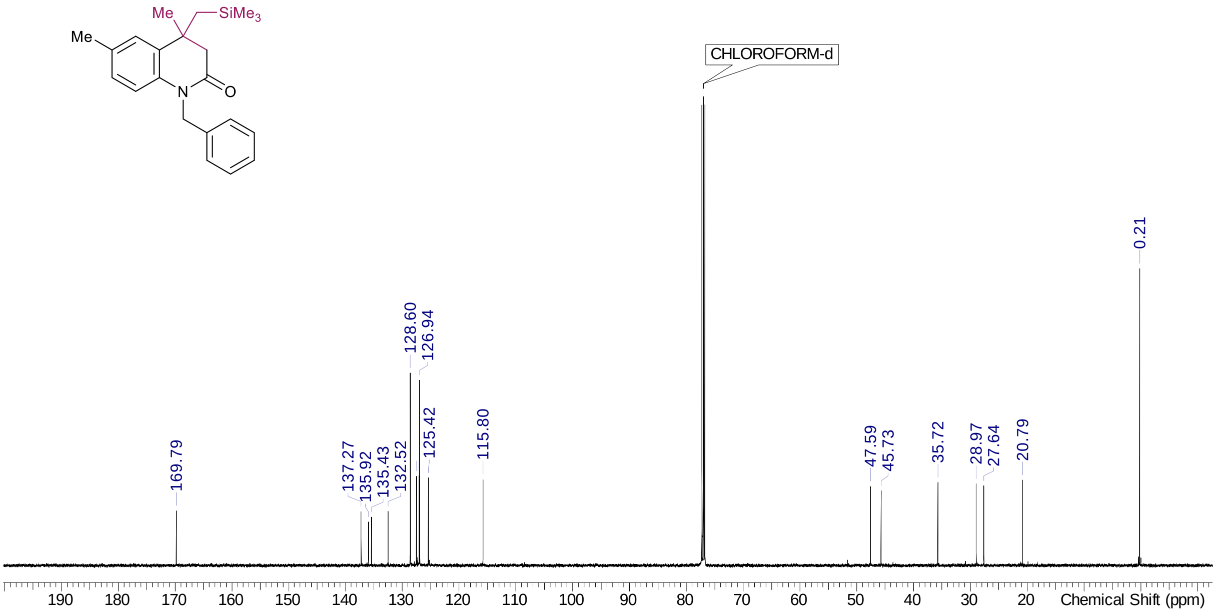

^{1}H, $^{13}C\{^{1}H\}$-HSQC NMR ($CDCl_3$, 400, 101 MHz)

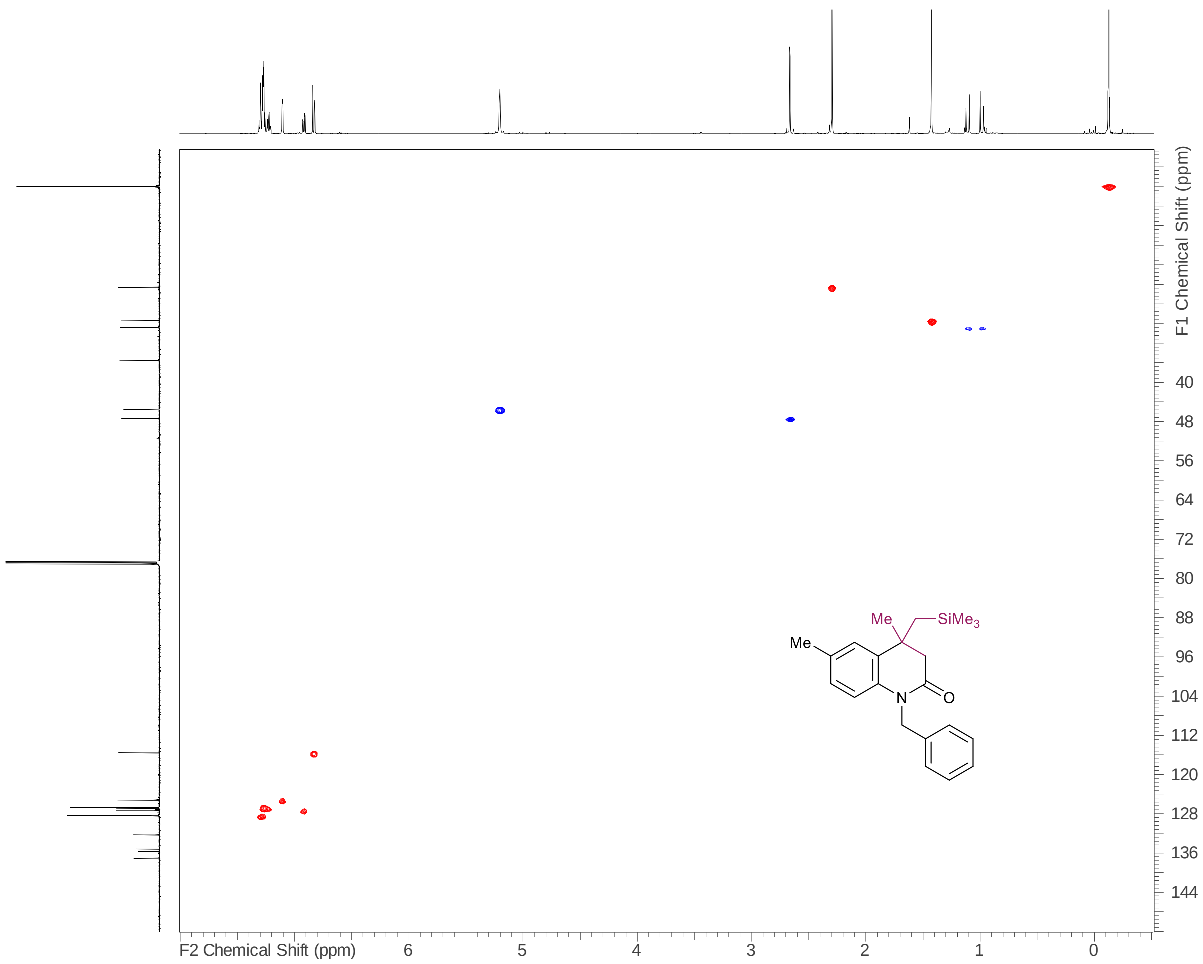

4,6-Dimethyl-4-((trimethylsilyl)methyl)-1,2,3,4-tetrahydroquinoline, 12

^{1}H NMR (400 MHz, $CDCl_3$)

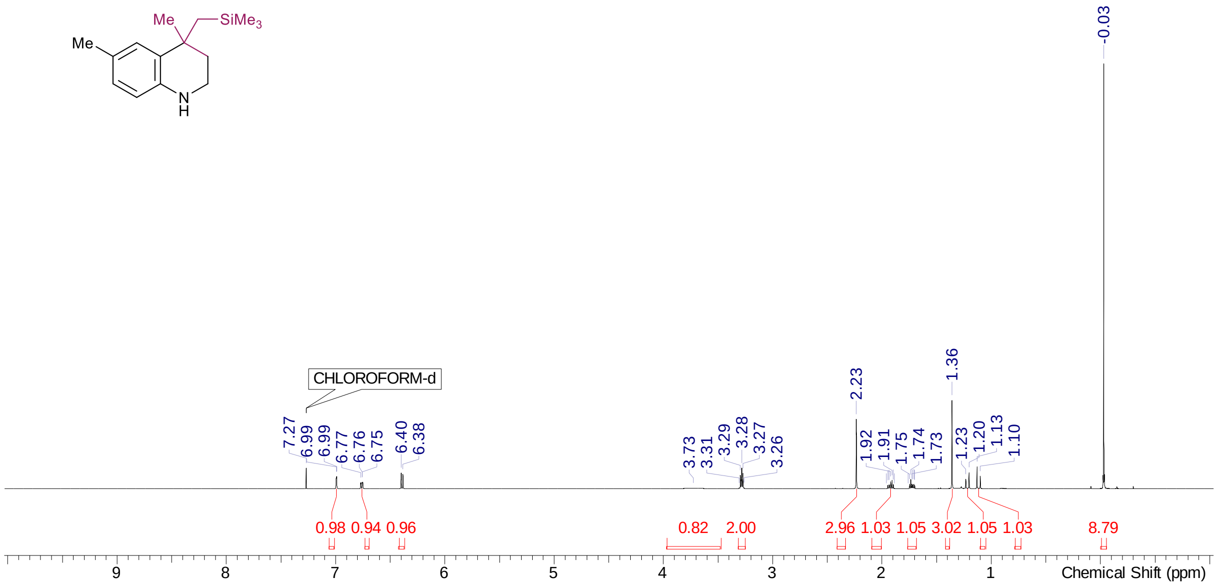

^{13}C{^{1}H} NMR ($CDCl_3$, 101 MHz)

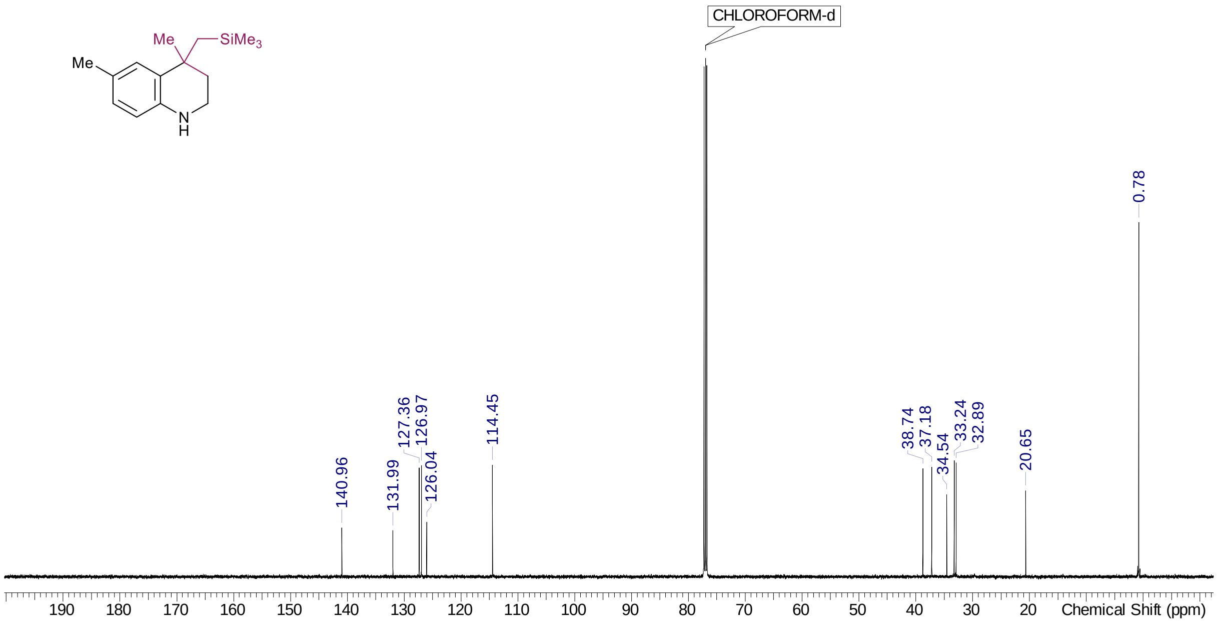

^{1}H, $^{13}C\{^{1}H\}$-HSQC NMR ($CDCl_3$, 400, 101 MHz)

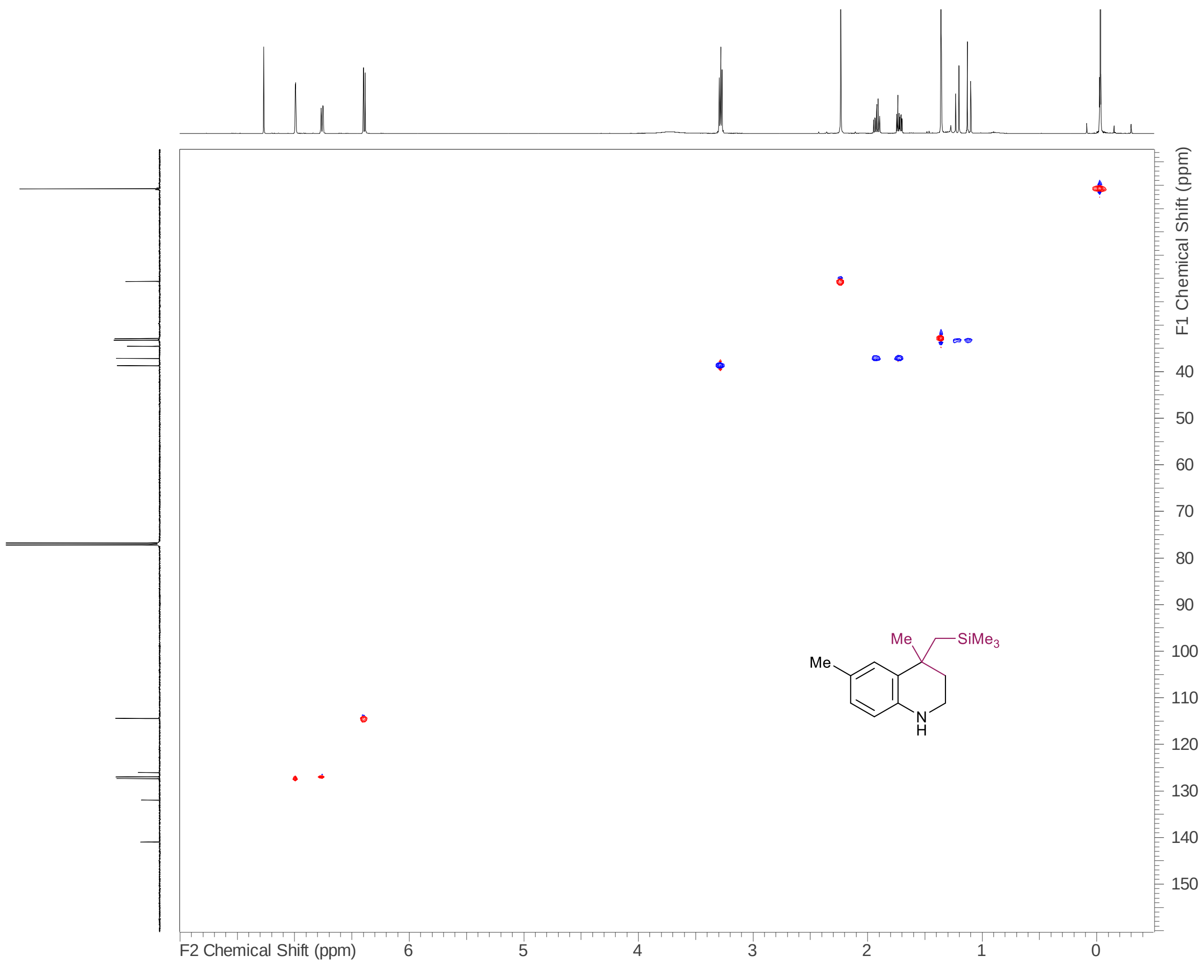

(4*RS*,4a*RS*,8a*RS*)-4,6-Dimethyl-4-((trimethylsilyl)methyl)decahydroquinoline, 13

^{1}H NMR (400 MHz, $CDCl_3$)

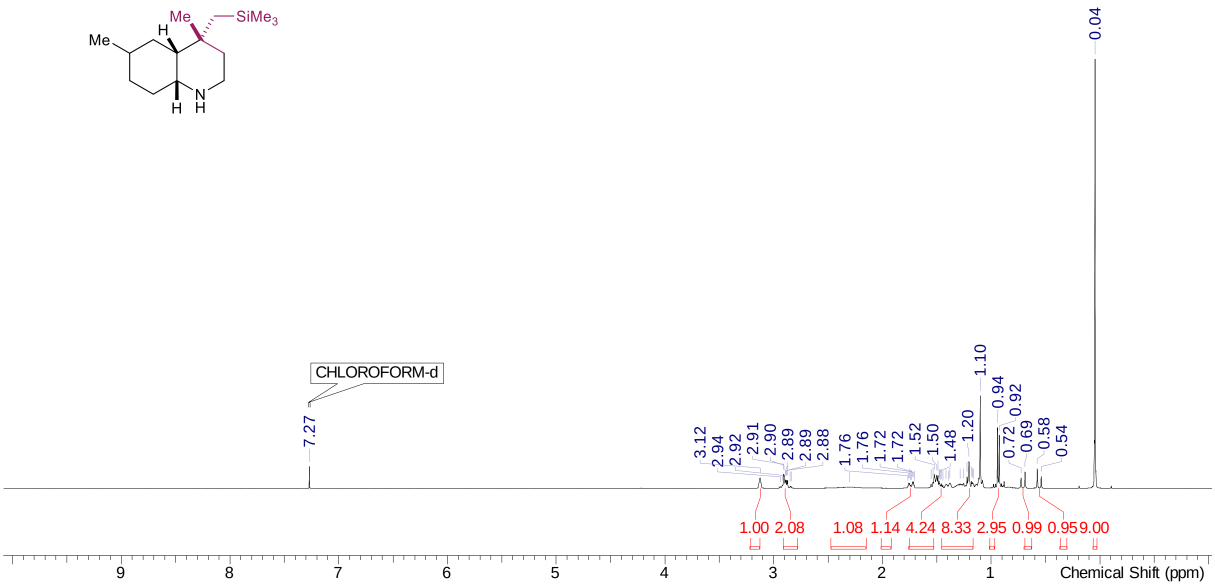

^{13}C{^{1}H} NMR ($CDCl_3$, 101 MHz)

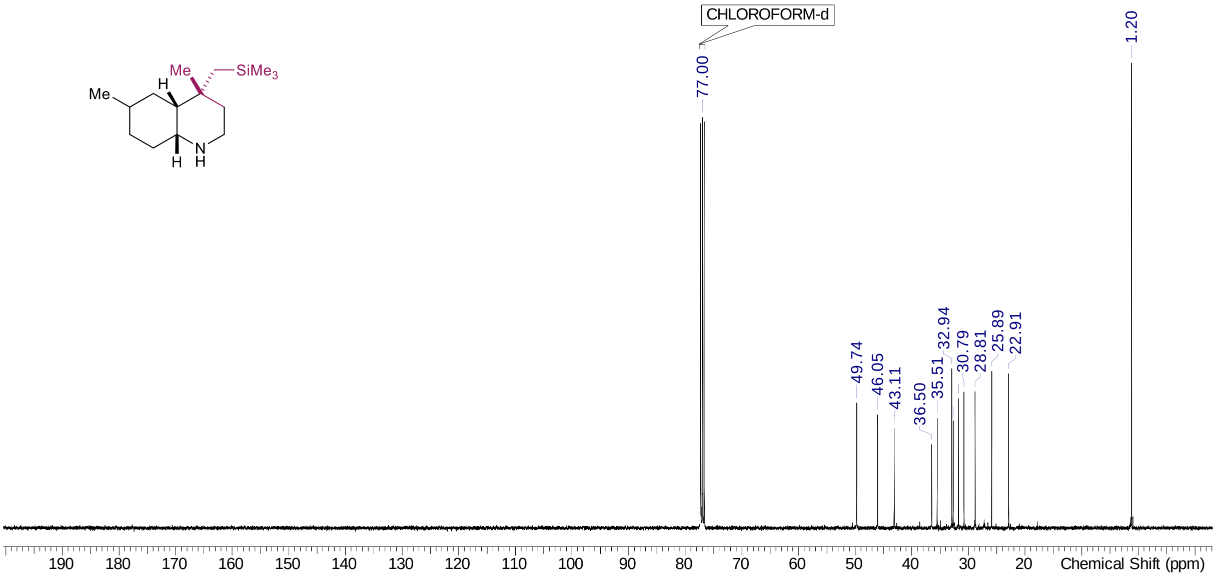

^{1}H, $^{13}C\{^{1}H\}$-HSQC NMR ($CDCl_3$, 400, 101 MHz)

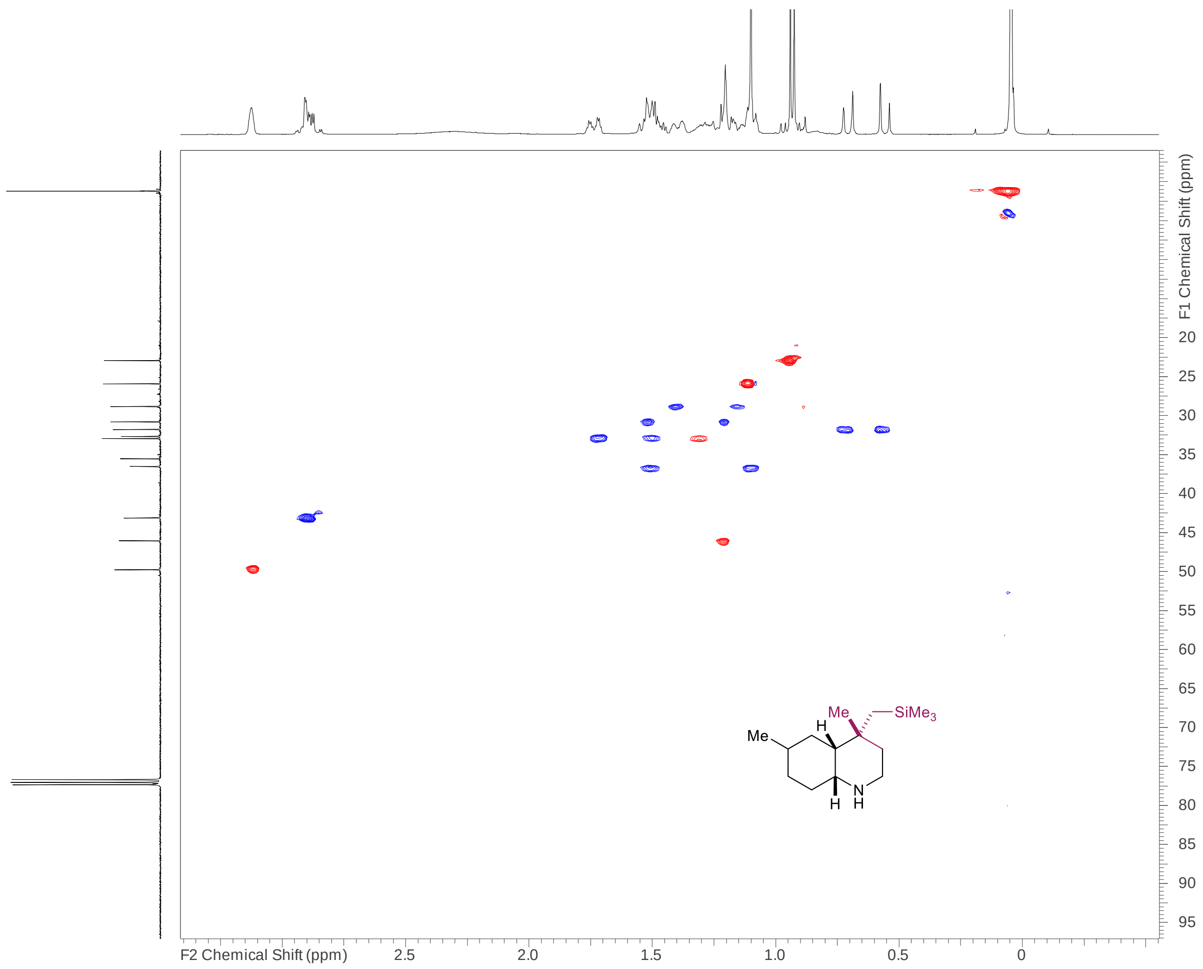

^{1}H, ^{1}H-NOESY NMR ($CDCl_3$, 400, 400 MHz)

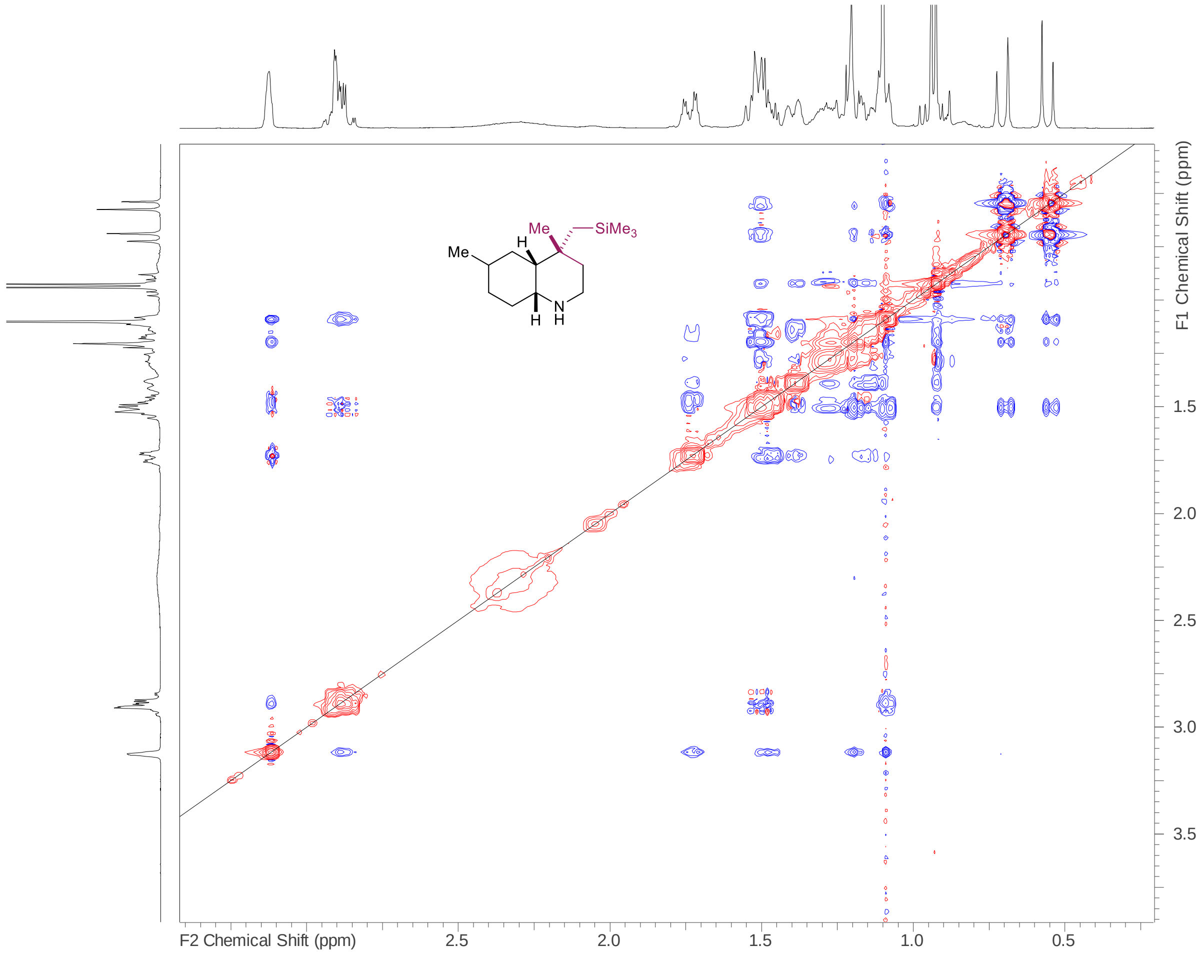

Supplement: Supplementary file 1 [file ol5c03023_si_001.pdf]
